# Differential interferon-α subtype induced immune signatures are associated with suppression of SARS-CoV-2 infection

**DOI:** 10.1073/pnas.2111600119

**Published:** 2022-02-07

**Authors:** Jonas Schuhenn, Toni Luise Meister, Daniel Todt, Thilo Bracht, Karin Schork, Jean-Noel Billaud, Carina Elsner, Natalie Heinen, Zehra Karakoese, Sibylle Haid, Sriram Kumar, Linda Brunotte, Martin Eisenacher, Yunyun Di, Jocelyne Lew, Darryl Falzarano, Jieliang Chen, Zhenghong Yuan, Thomas Pietschmann, Bettina Wiegmann, Hendrik Uebner, Christian Taube, Vu Thuy Khanh Le-Trilling, Mirko Trilling, Adalbert Krawczyk, Stephan Ludwig, Barbara Sitek, Eike Steinmann, Ulf Dittmer, Kerry J. Lavender, Kathrin Sutter, Stephanie Pfaender

**Affiliations:** ^a^Institute for Virology, University Hospital Essen, University Duisburg-Essen, 45122 Essen, Germany;; ^b^Molecular and Medical Virology, Ruhr-University Bochum, 44801 Bochum, Germany;; ^c^European Virus Bioinformatics Center (EVBC), 07743 Jena, Germany;; ^d^Medical Proteome Center, Ruhr-University Bochum, 44801 Bochum, Germany;; ^e^Department of Anesthesia, Intensive Care Medicine and Pain Therapy, University Hospital Knappschaftskrankenhaus Bochum, 44892 Bochum, Germany;; ^f^Center for Protein Diagnostics, Medical Proteome Analysis, Ruhr-University Bochum, 44801 Bochum, Germany;; ^g^Qiagen Digital Insights, Redwood City, CA 94063;; ^h^Department of Experimental Virology, Twincore, 30625 Hannover, Germany;; ^i^Institute of Virology Muenster, Westfaelische Wilhelms-University, 48149 Muenster, Germany;; ^j^Interdisciplinary Centre for Clinical Research, University of Muenster, 48149 Muenster, Germany;; ^k^Department of Biochemistry, Microbiology and Immunology, College of Medicine, University of Saskatchewan, Saskatoon, SK S7N 5E5, Canada;; ^l^Vaccine and Infectious Disease Organization–International Vaccine Centre, University of Saskatchewan, Saskatoon, SK S7N 5E3, Canada;; ^m^Key Laboratory of Medical Molecular Virology, School of Basic Medical Sciences, Shanghai Medical College, Fudan University, Shanghai 200433, China;; ^n^Cluster of Excellence RESIST, Hannover Medical School, 30625 Hannover, Germany;; ^o^German Center for Infection Research, Partner Site Hannover-Braunschweig, 30625 Hannover, Germany;; ^p^Department for Cardiothoracic, Transplantation and Vascular Surgery, Hannover Medical School, 30625 Hannover, Germany;; ^q^Department of Pulmonary Medicine, Experimental Pneumology, University Medical Center Essen - Ruhrlandklinik, 45239 Essen, Germany;; ^r^Department of Infectious Diseases, West German Centre of Infectious Diseases, University Hospital Essen, 45122 Essen, Germany

**Keywords:** SARS-CoV-2, type I IFNs, immunotherapy, antiviral

## Abstract

Type I interferons (IFN-I) exert pleiotropic biological effects during viral infections, balancing virus control versus immune-mediated pathologies, and have been successfully employed for the treatment of viral diseases. Humans express 12 IFN-alpha (α) subtypes, which activate downstream signaling cascades and result in distinct patterns of immune responses and differential antiviral responses. Inborn errors in IFN-I immunity and the presence of anti-IFN autoantibodies account for very severe courses of COVID-19; therefore, early administration of IFN-I may be protective against life-threatening disease. Here we comprehensively analyzed the antiviral activity of all IFNα subtypes against severe acute respiratory syndrome coronavirus 2 (SARS-CoV-2) to identify the underlying immune signatures and explore their therapeutic potential. Prophylaxis of primary human airway epithelial cells (hAEC) with different IFNα subtypes during SARS-CoV-2 infection uncovered distinct functional classes with high, intermediate, and low antiviral IFNs. In particular, IFNα5 showed superior antiviral activity against SARS-CoV-2 infection in vitro and in SARS-CoV-2–infected mice in vivo. Dose dependency studies further displayed additive effects upon coadministration with the broad antiviral drug remdesivir in cell culture. Transcriptomic analysis of IFN-treated hAEC revealed different transcriptional signatures, uncovering distinct, intersecting, and prototypical genes of individual IFNα subtypes. Global proteomic analyses systematically assessed the abundance of specific antiviral key effector molecules which are involved in IFN-I signaling pathways, negative regulation of viral processes, and immune effector processes for the potent antiviral IFNα5. Taken together, our data provide a systemic, multimodular definition of antiviral host responses mediated by defined IFN-I. This knowledge will support the development of novel therapeutic approaches against SARS-CoV-2.

## Significance

Type I interferons (IFN-I) exhibit various biological effects during viral infections, and they have been successfully used for clinical treatment of viral diseases. Humans express 12 IFNα subtypes, which strongly differ in their antiviral responses against different viruses. Here we analyzed the antiviral activity of all human IFNα subtypes against severe acute respiratory syndrome coronavirus 2 (SARS-CoV-2) to identify the underlying immune signatures and explore their therapeutic potential. Our data provide a systemic pattern of antiviral host effector responses mediated by high antiviral IFN-I, which could help to identify key cellular effectors targeted in novel therapeutic approaches against SARS-CoV-2 infection.

Without the capacity to produce or recognize interferons (IFN), mammalian hosts rapidly succumb in the case of viral infections. Accordingly, humans with loss-of-function mutations in the IFN signaling pathway even fail to control attenuated viruses. Therefore, IFNs are indispensable mediators of the first immediate intrinsic cellular defenses against invading pathogens, such as viruses. So far, three different types of IFNs, types I (IFN-I), IFN-II, and IFN-III, have been identified and classified based on their genetic, structural, and functional characteristics as well as receptor usages (1–3). IFN-I is among the first line of antiviral defense due to the ubiquitous expression of the surface receptor IFNAR consisting of two subunits, IFNAR1 and IFNAR2. In humans, the IFN-I family comprises IFNβ, IFNε, IFNκ, IFNω, and 12 IFNα subtypes. The latter code for the distinct human IFNα proteins: IFNα1, IFNα2, IFNα4, IFNα5, IFNα6, IFNα7, IFNα8, IFNα10, IFNα14, IFNα16, IFNα17, and IFNα21, encoded by 14 nonallelic genes, including one pseudogene and two genes that encode identical proteins (IFNα13 and IFNα1). The overall identity of the IFNα proteins ranges from 75 to 99% amino acid sequence identity (1, 4). Despite their binding to the same cellular receptor, their antiviral and antiproliferative potencies differ considerably (5–10). As a general event in terms of signal transduction, IFNα subtypes engage the IFNAR1/IFNAR2 receptor and initiate a signal transduction cascade resulting in the phosphorylation of receptor-associated Janus tyrosine kinases culminating in downstream signaling events including the activation of IFN-stimulated gene (ISG) factor 3 (ISGF3) consisting of phosphorylated STAT1 and STAT2 and the IFN regulatory factor 9. ISGF3 binding to the IFN-stimulated response elements (ISRE), in promotor regions of various genes, initiates the transcriptional activation of a large number of ISGs, which elicit direct antiviral, antiproliferative, and immunoregulatory properties (11). Why different IFNα proteins exhibit distinct effector functions is largely elusive (5, 6, 12). Different receptor affinities and/or interaction interfaces within the IFNAR have been discussed which may account for the observed variability in the biological activity (13, 14). Furthermore, the dose, the cell type, the timing, and the present cytokine milieu might further affect the IFN effector response (15). In the absence of specific antiviral drugs, treatment of patients with IFN-I is often considered as a first therapeutic response, given its successful clinical application against viral infections (16, 17). Recently, IFN-III (IFN-lambda, IFNλ) received significant attention and are currently explored in clinical trials (18). IFNλ binds to the IFN-III receptor, which is preferentially expressed on epithelial cells and certain myeloid cells (19), resulting in restricted cell signaling and compartmentalized activity. Especially at epithelial surface barriers, IFNλ mounts an effective local innate immune response, by conferring viral control and inducing immunity without generating systemic activation of the immune system which could trigger pathologic inflammatory responses. Signal transduction cascades of IFN-I and IFN-III are considered to be rather similar, resulting in overlapping ISG signatures; however, IFN-I signaling leads to a more rapid induction and decline of ISG expression (20).

The outbreak of novel viruses, as exemplified by the recent emergence of severe acute respiratory syndrome coronavirus 2 (SARS-CoV-2), causing the disease COVID-19, has emphasized the urgent need for fast and effective therapeutic strategies. Indeed, IFN-I treatment is currently being explored as an emergency treatment against COVID-19 in various clinical trials (21–23), and it was already shown that SARS-CoV-2 is sensitive to IFN-I (24) and ISGs (25). Given their large genome size, CoVs have evolved a variety of strategies circumventing the host innate immune reaction, including evasion strategies targeting IFN-I signaling (24, 26–28). Along those lines, recent studies showed significantly decreased IFN activity in COVID-19 patients who developed more severe disease (29), highlighting the importance of IFN in controlling viral infection. Against viruses, pegylated IFNα2 is approved and frequently administered in clinical settings. However, common side effects include the occurrence of flu-like symptoms, hematological toxicity, elevated transaminases, nausea, fatigue, and psychiatric sequelae, which often result from systemic activation of the immune system (30). Given the described distinct biological properties of IFNα subtypes, we comprehensively studied their antiviral effect against SARS-CoV-2 in comparison to another respiratory virus (influenza A virus [IAV]), and we aimed to explore SARS-CoV-2–specific immune signatures that could contribute to an efficient viral clearance. Accordingly, the aim of this study was twofold: 1) to identify underlying immune signatures crucial for controlling SARS-CoV-2 infection and 2) to explore the therapeutic potential of IFNα subtypes in SARS-CoV-2 infection.

## Results

### IFNα Subtypes Differentially Inhibit SARS-CoV-2.

**Fig. 1.**
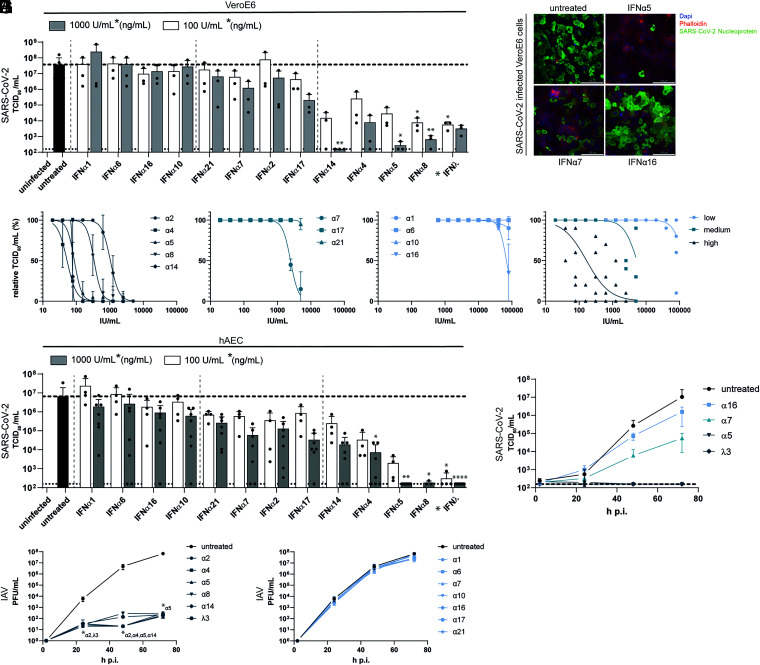
Treatment with IFNα subtypes reveals distinct antiviral effects against SARS-CoV-2. (*A*) Antiviral activity of IFNα subtypes (100 U/mL or 1,000 U/mL) and IFNλ3 (100 ng/mL or 1,000 ng/mL) against SARS-CoV-2 on VeroE6 cells (TCID_50_ per milliliter). (*B*) Representative immunofluorescence staining of IFN-treated SARS-CoV-2–infected VeroE6 cells. (Scale bar: 100 µm.) (*C*–*F*) IFNα subtypes were titrated against SARS-CoV-2 on VeroE6 cells by TCID_50_ assay, and the IFNs were grouped in high (*C*), medium (*D*), and low (*E*) antiviral patterns, and the mean values of each group are plotted in *F*. (*G* and *H*) Antiviral activity of IFNα subtypes and IFNλ3 in SARS-CoV-2–infected primary hAECs at 72 h p.i. (*G*) and kinetics of four selected IFNs (*H*). (*I* and *J*) Antiviral activity of IFNα subtypes and IFNλ3 in Influenza A/PR8-infected primary hAECs at different time points p.i. Mean values of high (*I*) and low/not (*J*) antiviral IFNs are shown. In *A* and *G*, each data point represents a biological replicate or an individual donor. In *A*, *C*–*F*, *I*, and *J*, mean values ± SEM are shown for *n* = 3. In *G* and *H*, *n* = 4 to 7. Statistical tests were performed for the individual IFN-treated groups against the untreated control group. *P < 0.05; **P < 0.01; ****P < 0.0001.

In order to determine the antiviral potencies of the 12 different IFNα subtypes against SARS-CoV-2, we pretreated VeroE6 cells with two doses (1,000 units per mL [U/mL] and 100 U/mL) for 16 h. This time point was chosen to include early and late ISGs into our analyses. We included IFNλ3 (1,000 ng/mL and 100 ng/mL) as a positive control, since its potent antiviral activity against SARS-CoV-2 and other respiratory pathogens has been documented (31, 32). Following treatment, cells were subsequently infected with SARS-CoV-2, and viral replication was quantified by determining infectious viruses (TCID_50_ per milliliter) and genome amplification. Interestingly, we observed a differential antiviral pattern for the individual subtypes, with IFNα5, α4, α8, α14, and IFNλ3 exhibiting the strongest antiviral effects with up to 10^5^-fold reduction in viral titers (Fig. 1*A* and *SI Appendix*, Fig. S1*A*). Immunofluorescence analysis of VeroE6 cells pretreated with IFNα5, IFNα7, and IFNα16 confirmed their different antiviral activities against SARS-CoV-2 (Fig. 1*B*). Analysis of ISG induction by low antiviral IFNα subtypes α1 and IFNα16 in comparison to IFNα5 revealed an induction of *OAS2A* and *IFITM3*, indicating that low antiviral subtypes are functionally active proteins (*SI Appendix*, Fig. S2*A*). To determine the inhibitory concentration 50 (IC_50_), we performed dose–response analyses covering concentrations from 19 U/mL to 80,000 U/mL for the pretreatment. SARS-CoV-2 replication was assessed by quantification of viral titers (Tissue Culture Infection Dose 50 [TCID_50_] per milliliter) and viral antigens applying a previously described in-cell enzyme-linked immunosorbent assay (icELISA) (33) (Table 1 and *SI Appendix*, Fig. S1 *B*–*D*). Corroborating previous results, a striking clustering of the antiviral subtypes according to their antiviral potency was observed, which allowed their separation into classes of low (IC_50_ > 5,000 U/mL), intermediate (IC_50_ = 2,000 U/mL to 5,000 U/mL) and high (IC_50_ < 2,000 U/mL) antiviral activities against SARS-CoV-2 (Fig. 1 *C*–*F* and *SI Appendix*, Fig. S1 *B*–*D* and Table 1). Since VeroE6 cells are derived from African green monkey, expressing the nonhuman primate instead of human IFN receptor, and also lack the capacity to produce IFN-I in a natural feed-forward loop (34), we further analyzed genuine target cells of SARS-CoV-2. We utilized well-differentiated primary human airway epithelial cells (hAEC), which closely resemble the in vivo physiology of the respiratory system, and differentiate into various cells types, resulting in ciliary movement and production of mucus (35, 36). After IFN pretreatment and subsequent infection with SARS-CoV-2, apical washes were monitored concerning viral replication kinetics at 33 °C (37). Cells were lysed at 72 h postinfection (p.i.), and viral progeny (Fig. 1 *G* and *H*) as well as viral *M* and *N* gene expression (*SI Appendix*, Fig. S1 *E*–*J*) were determined. Again, a distinct antiviral pattern became evident (Fig. 1*G*) defining IFN clusters of high (IFNα5, IFNα4, IFNα14, and IFNλ3), moderate (IFNα17, IFNα2, IFNα7, and IFNα21) and low antiviral activities (IFNα10, IFNα16, IFNα6, and IFNα1) (Fig. 1*H* and *SI Appendix*, Fig. S1 *G* and *J*). Prototypical ISG expression patterns, as analyzed by qRT-PCR, revealed subtype-specific gene expression signatures (*SI Appendix*, Fig. S2*B*). In order to address whether the observed antiviral activities were SARS-CoV-2 specific, we additionally tested IAV (IAV/PR8) in hAECs. Interestingly, pretreatment of hAECs with the IFN subtypes revealed differences compared to SARS-CoV-2. In general, antiviral responses could be clustered into strong antiviral activities for IFNα2, IFNα4, IFNα5, IFNα8, IFNα14, and IFNλ3 (Fig. 1*I*) and weak antiviral activities for IFNα1, IFNα6, IFNα7, IFNα10, IFNα16, IFNα17, and IFNα21 (Fig. 1*J*). Among the strong antiviral responses, we observed additional transient differences at 48 h p.i., with IFNα2, IFNα4, IFNα5, and IFNα14 being slightly superior to IFNα8 and IFNλ3 (Fig. 1*I*). These results clearly demonstrate that different IFNα subtypes mediate distinct biological and temporal activities.

**Table 1.** IC_50_ values of IFNα subtypes on VeroE6 cells obtained from endpoint dilution assay

IFNα subtype	IC_50_ (U/mL)
IFNα4	56.91
IFNα14	70.73
IFNα5	79.73
IFNα8	327.0
IFNα2	1,026
IFNα7	2,431
IFNα21	4,944
IFNα16	>5,000
IFNα1	>5,000
IFNα17	>5,000
IFNα6	>5,000
IFNα10	>5,000

### IFN Subtype–Specific Gene Expression Signatures.

**Fig. 2.**
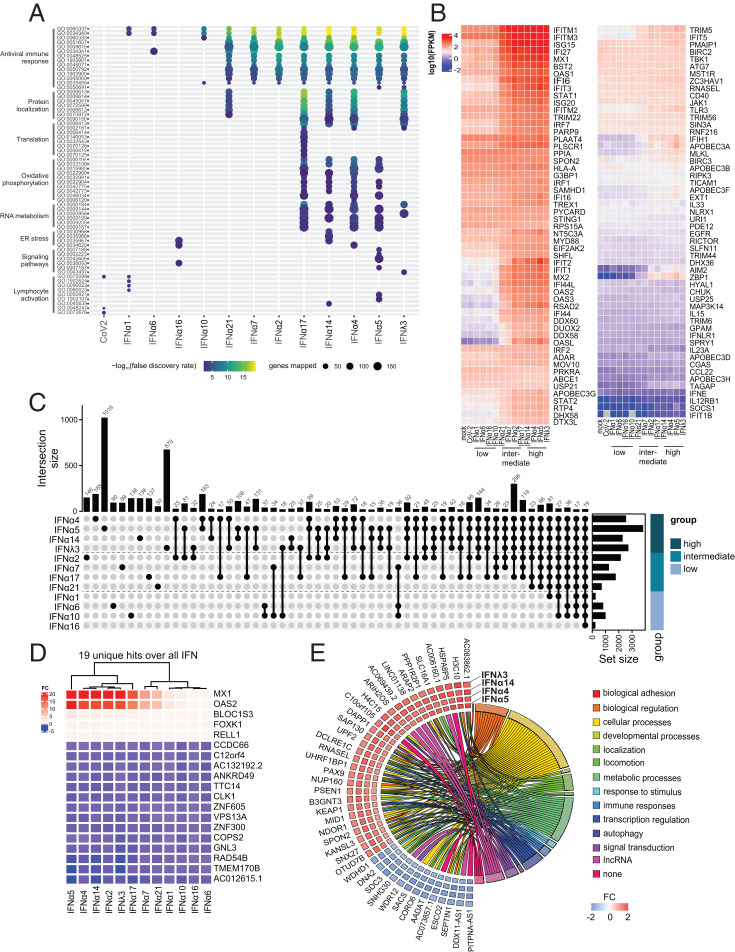
Transcriptomic analyses display IFN subtype–specific immune signatures. Transcriptomic analyses of IFN-treated (16 h posttreatment; 1,000 U/mL or 1,000 ng/mL) or SARS-CoV-2–infected (18 h p.i.) hAECs. (*A*) Biological processes induced by IFNs or SARS-CoV-2. (*B*) Heat maps displaying genes contained in antiviral response. (*C*) UpSet plots to summarize key DEGs. Numbers of individual or group-specific DEGs are shown as bars and numbers. The bottom right horizontal bar graph labeled Set Size shows the total number of DEGs per treatment. IFNs are plotted, according to their antiviral activity, in three groups (high, medium, and low). (*D*) Heatmap of the 19 basal DEGs expressed by all IFNs as identified in *C*. (*E*) Plot depicting fold changes (FC) of the identified 42 unique genes in the group displaying high antiviral activity and association of genes to functional categories. In *A*–*E*, *n* = 4.

Since we observed clear differences in the biological activities of different IFNα subtypes against SARS-CoV-2, we next aimed to identify their underlying immune signatures and mechanisms. To this end, primary hAECs were pretreated with the respective IFNs, and, 16 h poststimulation, cellular RNA was sequenced on an Illumina NovaSeq 6000, and differentially expressed genes (DEGs) were sent to Ingenuity Pathway Analysis (IPA; Qiagen) for biological analysis. In order to investigate cellular responses following viral infection, we included SARS-CoV-2–infected hAECs (18 h p.i.) in our analysis. Global transcriptomic analysis revealed unique DEGs, both up- and down-regulated upon IFN treatment (38, 39) for each IFN (*SI Appendix*, Fig. S3*A*) compared to mock-treated cells. Similar to the observed antiviral effects, a general clustering was apparent which showed similar expression patterns for low to intermediate antiviral subtypes (IFNα1, IFNα6, IFNα7, IFNα16, IFNα10, and IFNα21) and intermediate to high antiviral subtypes (IFNα2, IFNα17, IFNα14, IFNα4, IFNα5, and IFNλ3). Interestingly, we observed a clear difference in the numbers of significantly up- and down-regulated genes after treatment with IFNα subtypes compared to mock-treated cells, which positively correlated with antiviral activity (*SI Appendix*, Fig. S3*B*). Gene ontology (GO) pathway analysis revealed higher expression of genes mostly involved in antiviral immune response among the medium and high antiviral subtypes, as well as pathways which can be associated with protein localization, translation, oxidative phosphorylation, RNA metabolism, endoplasmic reticulum (ER) stress, signaling pathways, and lymphocyte activation (Fig. 2*A*). Strikingly, different IFNα subtypes displayed unique GO patterns, with IFNα17, in contrast to other subtypes, regulating genes involved in translation, whereas the treatment with IFNα5 resulted in the strongest regulation of genes associated with signaling pathways and lymphocyte activation among all IFNs (Fig. 2*A*). We next focused on genes associated with antiviral responses (Fig. 2*B*). A separation based on antiviral activity could be discerned with weak antiviral IFNα subtypes (IFNα1, IFNα6, IFNα16, and IFNα10) exhibiting comparatively lower expression values of specific ISGs, whereas medium to strong antiviral IFNα subtypes induced higher expression (Fig. 2*B*). We observed two clusters that differed between low and intermediate to high IFN subtypes, with *ISG15*, *IFI27*, *MX1*, and others showing generally lower expression values in the low antiviral IFN subtypes. Even more pronounced were expression changes of *IFIT2*, *IFIT1*, *MX2*, and others which resulted in a down-regulation for the low and an up-regulation for the intermediate to high antiviral IFN subtypes. As we aimed at identifying immune signatures that correlate with the antiviral activity against SARS-CoV-2 infection, we next evaluated DEGs with respect to distinct, intersecting, and common genes among and between subtypes (*SI Appendix*, Fig. S4*A*). We identified several DEGs for each subtype, with IFNα5 expressing the most unique genes (1,018 DEGs), followed by IFNλ3 (670 DEGs) (Fig. 2*C* and *SI Appendix*, Fig. S4*B*). A comparison between high, medium, and low antiviral subtypes revealed that 19 genes were commonly differentially expressed among all subtypes, including *MX1* and *OAS2* (Fig. 2*D*). The most striking differences could be observed for *MX1* and *OAS2*, whose expression levels clearly separated high, intermediate, and low antiviral IFN subtypes (Fig. 2*D*). Interestingly, a comparison between high antiviral IFNs (IFNα4, IFNα5, IFNα14, and IFNλ3) and all other IFNα subtypes identified 42 distinct DEGs that were exclusively up- or down-regulated in the high antiviral group, including *RNaseL* and genes associated with regulation of transcription, signal transduction, and metabolic processes, as well as long noncoding (lnc) RNAs (Fig. 2*E*). In conclusion, we could clearly demonstrate IFN subtype–specific immune signatures that could contribute to the observed differences in antiviral activity.

### Proteomic Analysis Highlights Key Cellular Factors.

**Fig. 3.**
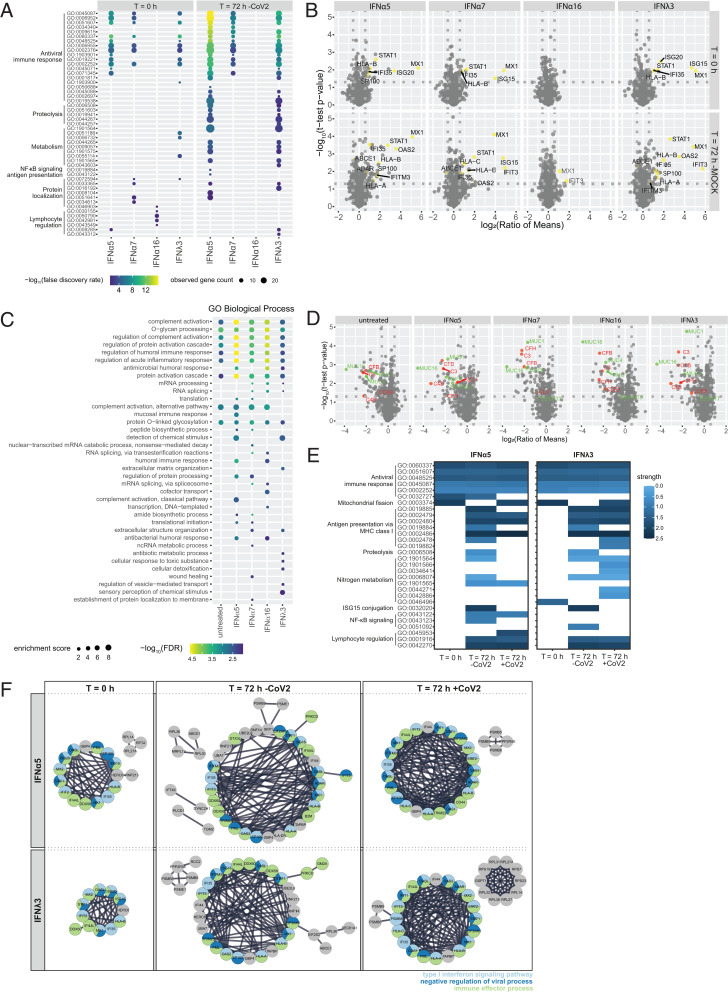
Proteomic analysis highlights key cellular mediators. Proteomic analysis of IFN-treated (1,000 U/mL or 1,000 ng/mL) and/or SARS-CoV-2–infected hAECs. (*A*) Biological processes induced by IFNs 16 h posttreatment (t = 0 h) or 88 h posttreatment (t = 72 h). (*B*) Volcano plots of IFN-treated hAECs at different time points posttreatment. Detected ISGs are colored yellow. (*C*) Biological processes induced by IFNs 88 h posttreatment in the presence of SARS-CoV-2 (t = 72 h); mRNA, messenger RNA; ncRNA, noncoding RNA. (*D*) Volcano plots of IFN-treated SARS-CoV-2–infected hAEC. Detected proteins are colored due to their biological function: red, complement activation; green, O-glycan processing. (*E*) Heatmaps of differentially activated biological processes by highly antiviral IFNα5 and IFNλ3 compared to untreated controls at different time points posttreatment in the presence and absence of SARS-CoV-2. (*F*) STRING analysis of proteins increased in IFN-treated and/or SARS-CoV-2–infected hAECs and identified abundant protein–protein interactions. Proteins are shown as circles and colors indicating biological processes. In *A*–*F*, *n* = 4.

Our transcriptomic analysis revealed IFNα subtype–specific distinct, intersecting, and common expression patterns of DEGs that most likely contribute to the differential biological activity against SARS-CoV-2. To further uncover relevant cellular effector proteins for the antiviral activity against SARS-CoV-2, we additionally performed proteomic analysis on hAECs pretreated with IFNs. Since we had observed the strongest antiviral activity for IFNα5 and IFNλ3, we decided to further investigate their specific proteomic profile in direct comparison with IFNα7, which exhibited a moderate antiviral effect, and IFNα16, displaying a weak effect against SARS-CoV-2 infection, in order to identify key antiviral pathways, crucial in controlling coronavirus infection. To this end, primary hAECs were pretreated with selected IFNs for 16 h. In addition to the early time point (t = 0 h), where we aim to identify key cellular factors that are expressed before viral infection, we included a late time point, 72 h posttreatment both in the presence (t = 72 h [CoV-2]) and absence of viral infection (t = 72 h [mock]), to investigate potential antiviral mechanisms and potential intervention by viral effectors (*SI Appendix*, Fig. S5*A*). Principal component analysis revealed a clustering according to donor and/or infection and time points (*SI Appendix*, Fig. S5 *B*–*D*). In addition to host cell proteins, various viral peptides were identified, which correlate to viral titers depending on the respective donor (*SI Appendix*, Table S1 and Fig. S5*E*). For all donors, no SARS-CoV-2 peptides could be detected following treatment with IFNα5 and IFNλ3. Pretreatment of cells with IFN subtypes resulted in up- or down-regulation of a variety of proteins compared to untreated hAECs, depending on the IFN stimulation (*SI Appendix*, Fig. S6 *A*–*C*). In order to perform statistical analysis, we considered proteins that were measured in a minimum of three of four donors; however, on/off analysis (defined as full absence of a protein in one group of a pairwise comparison) revealed additional proteins which might be of interest (*SI Appendix*, Fig. S6 *D*–*F* and Table S2). GO analysis of proteins differentially abundant between untreated and IFN-treated samples at each time point (untreated vs. IFN) identified enrichment of antiviral immune responses for all IFNs except IFNα16 (Fig. 3*A* and *SI Appendix*, Fig. S7*A*). For IFNα16, only proteins associated with lymphocyte regulation were induced, which likely do not contribute to SARS-CoV-2 restriction in cell culture but may be very important in vivo. At 72 h, pathways belonging to proteolysis, metabolism, and protein localization were additionally enriched after treatment with IFNα5 and IFNλ3. The most prominent up-regulated proteins, associated with IFN signaling (STAT1, MX1, ISG15, ISG20, IFI35, and others), were found to be on–off regulated and present only upon treatment with IFNα5, IFNα7, and IFNλ3. Additional ISGs, including IFIT3, OAS2, and IFITM3, were on–off regulated after 72 h and SARS-CoV-2 infection, except for IFNα16 treatment (Fig. 3*B* and *SI Appendix*, Fig. S7*B*). Interestingly, the comparison of samples in the presence or absence of SARS-CoV-2 (mock vs. CoV-2) showed a striking trend toward down-regulation of proteins upon SARS-CoV-2 infection. Enrichment of biological processes associated with complement activation and O-glycan processing (Fig. 3*C*) highlighted various complement factors (e.g., CFB, C4B, and C3) as well as various mucines (e.g., MUC1 and MUC16) by SARS-CoV-2, independent of IFN treatment and resulting viral titers (Fig. 3*D* and *SI Appendix*, Fig. S7 *C* and *E* and Table S3). In contrast, the strongest biological effects on antiviral immune responses after treatment with IFNα5 and IFNλ3, for example, IFN signaling as well as antigen presentation, nuclear factor κB signaling, or lymphocyte regulation, were not affected by viral infection. Interestingly, proteins belonging to other pathways, for example, antigen presentation by major histocompatibility complex (MHC) class I or proteolysis, seemed to be less abundantly represented under viral infection in the IFNα5-treated samples, a phenomenon which was not as prominent after treatment with IFNλ3 (Fig. 3*E* and *SI Appendix*, Fig. S7*D*). STRING (Search Tool for the Retrieval of Interacting Genes/Proteins) analysis (Fig. 3*F*) highlighted the presence of antiviral key effector molecules (e.g., ISG20, ISG15, IFI44L, IFIT2, IFIT3, IFI35, PML, and SP100), which are involved in IFN-I signaling pathways, negative regulation of viral processes, and immune effector processes among the most potent antiviral IFNs. In conclusion, we identified a variety of antiviral cellular effector molecules that correlate with antiviral activity and control of coronavirus infection.

### Therapeutic Potential of IFNα Subtypes.

**Fig. 4.**
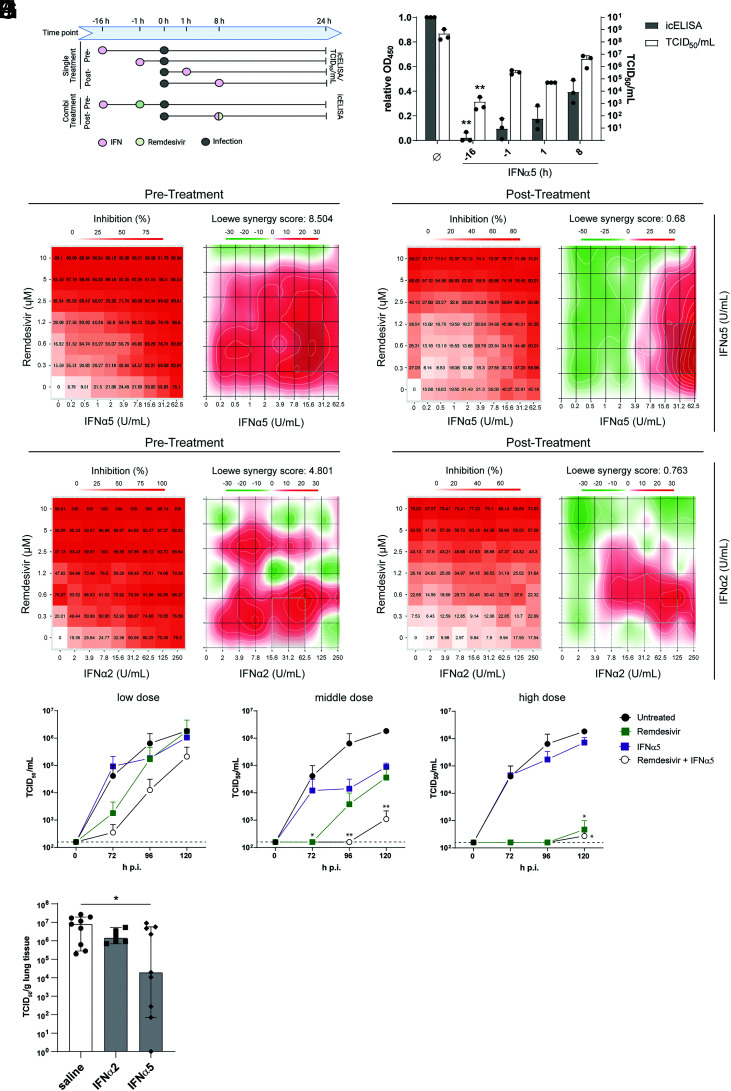
Therapeutic potential of highly antiviral IFNα subtypes. (*A*) Schematic depiction of treatment. (*B*) Pretreatments and posttreatments with IFNα5 of VeroE6 cells by icELISA (gray bars) and TCID_50_ assay (white bars). Each data point represents a biological replicate measured at an optical density at 450 nm (OD_450_). (*C*) Inhibition of SARS-CoV-2 infection by IFNα5 and analysis of drug combination experiments using SynergyFinder web application (72) 16 h before infection (Pre-Treatment). (*D*) Inhibition of SARS-CoV-2 infection and analysis of drug combination experiments using SynergyFinder web application 8 h p.i. (Post-Treatment). (*E*) Inhibition of SARS-CoV-2 infection by clinically approved IFNα2 and analysis of drug combination experiments using SynergyFinder web application 16 h before infection (Pre-Treatment). (*F*) Inhibition of SARS-CoV-2 infection and analysis of drug combination experiments using SynergyFinder web application 8 h p.i. (Post-Treatment). (*G*–*I*) Remdesivir and IFNα5 combinational treatment 8 h p.i. of hAECs with low doses (0.313 μM remdesivir, 0.2444 U/mL IFNα5; *G*), medium doses (0.63 μM remdesivir, 15.625 U/mL IFNα5; *H*) and high doses (2.5 μM remdesivir, 1.953 U/mL IFNα5; *I*). (*J*) Therapeutic effect of IFNα5 and IFNα2 in SARS-CoV-2–infected LoM. In *B*–*I*, *n* = 3. In *J*, n =9. *P < 0.05; **P < 0.01.

Currently, there are only a few approved specific antiviral drugs (e.g., monoclonal antibodies) (40, 41) for the treatment of COVID-19, which severely limit treatment options during severe clinical courses. Remdesivir, a nucleotide-analogous RNA-dependent RNA polymerase inhibitor originally developed as antiviral against Ebola virus, received an emergency use approval against COVID-19 and has been employed in clinics. Unfortunately, due to lack of evidence for recovery of critically ill patients, it is no longer recommended by the World Health Organization as a single treatment for COVID-19 (42). Therefore, alternative therapeutic approaches such as combination therapies are urgently needed. As we have observed the strongest antiviral effect in this study for IFNα5, we explored its therapeutic potential in comparison and in combination with remdesivir. Additionally, we included IFNα2 as the clinically approved IFNα in this analysis. In regard to patients viewed as an entity, prophylactic treatment with IFNs is not a clinical option. Nevertheless, a treatment initiated following diagnosis can still “prophylactically” condition and protect cells in the body against later infection events. To monitor the kinetics of the antiviral activity of IFNα subtypes, we treated cells either before infection (“pre”) or up to 8 h p.i. (“post”) and studied the antiviral activity by determining viral titers as TCID_50_ per milliliter and viral antigens by in-cell ELISA (icELISA) (Fig. 4 *A* and *B*). As expected, the strongest reduction in viral titers was observed upon pretreatment with IFNα5 as cells become alerted toward an antiviral state, and antiviral effectors can be transcribed or even translated prior to viral infection (Fig. 4*B*). Intriguingly, even after viral infection was established, treatment with IFNα5 was able to significantly reduce viral titers (Fig. 4*B*), which was also observed with the antiviral drug remdesivir (*SI Appendix*, Fig. S8*A*). Given the clear antiviral but incomplete inhibitory effect of both treatment modalities, we next studied a potential beneficial effect of IFNα5 or IFNα2 when coadministered with remdesivir (scheme in Fig. 4*A*). To this end, we analyzed the antiviral effect upon pretreatment as well as posttreatment of an established infection. To quantify the interaction between the antiviral drugs, the observed combination response was compared to the expected effect, using the Loewe additivity model, with δ-scores above 10 indicating synergistic effects. Combination therapies in VeroE6 cells revealed an additive antiviral activity, with over 90% viral inhibition upon pretreatment in the highest concentrations of both combinations tested and a Loewe synergistic score of 8.504 and 4.801 for IFNα5 and IFNα2, respectively (Fig. 4 *C* and *E*), without any cytotoxicity (*SI Appendix*, Fig. S8*B*). Likewise, posttreatment in combination with remdesivir resulted in a dose-dependent, additive viral inhibition with over 70% (Fig. 4 *D* and *F*) for both IFNα subtypes. To confirm these findings, we analyzed selected combinations of IFNα5 with remdesivir postinfection in hAEC. For this, we combined low doses (0.313 μM remdesivir, 0.2444 U/mL IFNα5), medium doses (0.63 μM remdesivir, 15.625 U/mL IFNα5), and high doses (2.5 μM remdesivir, 1.953 U/mL IFNα5), and observed, in all combinations, an additive therapeutic effect when coadministered 8 h p.i. (Fig. 4 *G*–*I*). To further strengthen the therapeutic potential of high antiviral IFNα subtypes, we performed therapeutic treatments with IFNα2 and IFNα5 in Rag2^−/−^γc^−/−^CD47^−/−^ triple-knockout (TKO) mice, which received human fetal lung transplants (humanized lung-only mice [LoM]). Treatment for 4 d with the highly antiviral IFNα5, starting 2 h postchallenge with SARS-CoV-2, significantly reduced viral titers in human lung organoids (Fig. 4*J*). Taken together, we provide evidence that coadministration of direct antiviral drugs together with potent IFNα subtypes clearly impaired viral replication and might provide an alternative therapeutic approach.

## Discussion

IFN-I serve as one of the first lines of defense and are induced almost immediately upon viral encounters. IFN-I foster intrinsic immunity, stimulate innate immunity, and recruit and orchestrate adaptive immunity. They can modulate the immune system in several ways, by exerting a wide range of biological activities including antiviral, antiproliferative, immunomodulatory, and regulatory activities. Importantly, impaired IFN-I activity are correlated with severe courses of COVID-19, highlighting their clinical importance (43). Accordingly, defectiveness to IFN-I significantly contributes to disease severity, and genetic polymorphisms decreasing IFN-I production are associated with more severe cases of COVID-19 (44–46). Furthermore, pegylated IFNα2a therapy in patients with inborn errors of IFN-I immunity prevented severe COVID-19 disease (47). In addition to the impaired IFN-I response triggered by SARS-CoV-2, recent studies have demonstrated the development of autoantibodies that can neutralize IFN-I (45, 48). To evade the antiviral effects of IFN-I, viruses have evolved various strategies to suppress IFN induction. SARS-CoV-2 codes for several proteins that have been implicated in IFN-I antagonism, thereby compromising host responses and favoring viral replication (49). Thus, early administration of IFN-I might be an effective treatment option for COVID-19 patients. The IFN-I family consists of multiple IFNα subtypes, which are highly conserved, and they all signal through the same ubiquitously expressed IFNAR1/IFNAR2. Activation of various downstream signaling cascades implicates that the IFNα subtypes share some overlapping functions, but also possess unique properties. Upon pretreatment of cells with 12 distinct IFNα subtypes, we observed cluster-specific antiviral patterns which were distinct between different viruses. These differential antiviral functions cannot be explained solely by the binding affinity to both receptor subunits, as IFNα5 and IFNα4 exhibit a median affinity to IFNAR1 and IFNAR2 in the range of 0.94 µM to 3 µM and 2.1 nM to 3.8 nM, respectively (13). Furthermore, the increased gene induction did not correlate with binding affinity to IFNAR1 or IFNAR2, as those IFNs with the highest binding affinity to IFNAR2 (IFNα10, IFNα17, IFNα6, IFNα14, and IFNα7) did not induce significantly higher numbers of DEGs. In IFN-treated gut biopsies of chronically HIV-infected patients, the numbers of induced genes by different IFN-I (IFNα1, IFNα2, IFNα5, IFNα8, IFNα14, and IFNβ) were not associated with binding affinity or ISRE activation (12). Importantly, it has been shown that the different IFN-I induced a specific pattern of genes, which are involved in various biological processes (12). Furthermore, these distinct IFN-induced ISG expression patterns clearly differ between subtypes in different cell types as well as in response to different viruses, indicating qualitative differences in IFNα subtype targeted antiviral responses (5, 12, 50). We observed distinct antiviral patterns, that could be clearly clustered into high, intermediate, and low antiviral effects against SARS-CoV-2. Interestingly, we identified 19 genes that were common between all groups, indicative of a basal IFN response. On top of that basal response, we identified several genes that were distinct, intersecting, or commonly differentially regulated between the high and/or medium group. Our dataset enabled us to identify expression patterns that can be correlated with antiviral activity against SARS-CoV-2. Foremost, antiviral immune responses were significantly dysregulated in the moderate and high antiviral groups. Nevertheless, several biological processes, for example, such as associated with protein localization, translation, or ER stress, displayed variable induction patterns depending on the IFNα subtype. Proteomic analysis confirmed expression of IFN effector molecules in high and moderate antiviral subtypes. We mostly identified factors involved in IFN-I signaling pathways, negative regulation of viral processes, and immune effector processes. These results clearly demonstrate unique and overarching properties of different IFNα subtypes. Another group recently reported that saturated concentrations (1,000 pg/mL) of IFNα subtypes against HIV-1 in vitro induced similar levels of 25 canonical ISGs (51). The authors concluded, from these 25 ISGs, that the overall difference between all subtypes is only quantitative, but not qualitative, implying that the transcription of 25 genes is fully sufficient to describe the whole interferome (52). We similarly observe a clear difference in the magnitude of differential regulated genes, that likely contributes to the observed antiviral patterns. Nevertheless, as demonstrated with IAV, these patterns do affect virus replication to a different extent, indicating that individual IFNα subtypes might have discriminative clinical effects in different viral infections. Due to its known antiviral activity and its clinical administration in chronic viral infections, IFN-I, specifically IFNα2 or IFNβ, were already used in a variety of different clinical trials in patients with mild or severe COVID-19. During SARS-CoV-2 infection, two phases can be observed: 1) an early phase with weak IFNα/IFNβ production and limited antiviral responses and 2) an excessive inflammatory immune response which can give rise to cytokine storms or acute respiratory distress syndrome. Therefore, a potential beneficial effect of IFN treatment must occur early during infection to not exacerbate hyperinflammation. Early subcutaneous administration of IFNβ in combination with lopinavir/ritonavir and ribavirin in patients with mild to moderate COVID-19 led to a significant reduction of symptoms, shortening the duration of viral shedding and hospital stay (23). Pulmonary administration of IFN-I might reduce systemic side effects, while increasing IFN-I concentrations in the infected epithelial cells. Inhaled or nebulized IFNα2b with arbidol or IFNβ-1b showed faster recovery from SARS-CoV-2 infection and decreased levels of inflammatory cytokines (21, 22). Furthermore, prophylactic intranasal application of IFNα2a/ IFNα2b in health care workers in China completely prevented new SARS-CoV-2 infections (53). A recent report from SARS-CoV-2 infection in golden hamsters demonstrated a systemic inflammation in distal organs like brain or intestine (54). The authors hypothesized that virus-derived molecular patterns and not infectious SARS-CoV-2 were disseminated to the periphery, leading to systemic inflammation and increased IFN signatures. These observations might further highlight the need to apply IFN-I via intranasal route or inhalation, as the IFN response in the periphery is already highly stimulated, and systemic administration would not further increase the antiviral host immune response. We clearly demonstrated the additive benefit of combining treatment of IFN-I with a direct acting antiviral, for example, remdesivir, as well as a direct therapeutic effect of high antiviral IFNα5 in humanized LoM. Taken together, most of the data so far support the administration of IFN-I early during infection to curb viral infection and lessen disease severity. Next to involvement of various cellular pathways, both on the transcriptomic and the proteomic levels, we identified unique signatures in primary hAEC after infection with SARS-CoV-2. Strikingly, despite reduced viral replication in the presence of highly antiviral IFNα subtypes, infection with SARS-CoV-2 resulted in a down-regulation of O-glycan processing. Mucus plays a vital role in protecting the respiratory tract from various factors, and serves as first line of defense against invading pathogens. Goblet cells secrete soluble mucus whose major components are heavily O-glycosylated mucin glycoproteins (55). Inflammatory conditions result in an increase of soluble and transmembrane mucins, and alteration of their glycosylation to boost mucosal defense (56, 57). Therefore, it is striking that we observed a consistent down-regulation of various mucins upon SARS-CoV-2 infection. Some recent studies have highlighted the highest level of expression of ACE2 and TMPRSS2, entry factors utilized by SARS-CoV-2, in the nasal goblet and ciliated cells in healthy individuals, cells which are also associated with high MUC1 and MUC5A expression levels (58, 59). Therefore, it is likely that these cells represent the initial infection route for the virus. It is tempting to speculate that virus infection of these cells triggers mucin down-regulation in order to impede cellular defense mechanisms. Interestingly, a significant proportion of COVID-19 patients present with dry cough, indicating that down-regulation of mucins could contribute to this clinical characteristic. In contrast, a recent study has described elevated MUC1 and MUC5AC protein levels in airway mucus of critically ill COVID-19 patients (60). However, the authors speculated that elevated mucin levels could originate from detached and disrupted epithelial cells. It will be interesting to further analyze the role of mucins and their glycans during COVID-19 pathogenesis and study the influence of viral replication on mucin expression. In conclusion, in this study, we provide a global characterization of the antiviral response of different IFNα subtypes on various levels and uncover immune signatures which are able to significantly reduce SARS-CoV-2 infection, as well as identifying unique features after virus infection of primary cell types. Our study contributes to an enhanced understanding of the molecular landscape controlling SARS-CoV-2 infection and could thereby pave the way toward novel therapeutic approaches upon identification of key cellular pathways and factors involved in SARS-CoV-2 infection.

## Methods

### Generation of Infectious SARS-CoV-2 Stocks.

The SARS-CoV-2 strain used in this study was isolated from patient material as described previously (61). For propagation, 2 × 10^6^ VeroE6 cells were seeded in a T75 flask and maintained in Dulbecco’s modified Eagle’s medium (DMEM) supplemented with 10% fetal bovine serum (FBS), L-glutamine, penicillin, and streptomycin. The next day, the cells were infected with isolated virus, and, after 3 d of incubation, the supernatant was harvested, and cell debris was removed by centrifugation. Aliquots of the supernatant were prepared and stored at −80 °C. Viral titers were determined by performing an end-point dilution assay or a plaque assay in order to calculate the TCID_50_ or the plaque-forming units (PFU), respectively. Virus stock was sequenced and assigned to B.1.1.10 according to the Pangolin database (62), accession number EPI_ISL_602518.

### Stimulation with Different Human IFNα Subtypes.

IFNα subtypes were produced and purified as previously described (63). Briefly, recombinant IFNs were expressed in *Escherichia coli* after M13 phage transduction. To harvest the proteins, the bacteria were pelleted, and the protein-containing inclusion bodies were denatured by sonication, dissolved in 6 M guanidine-hydrochloride, and refolded in arginine. The recombinant proteins were further purified by ion exchange chromatography and size exclusion chromatography; specificity and purity of the proteins were verified after each step via a sodium dodecyl sulfate (SDS) gel. By phase separation of the products with Triton X-114, the remaining endotoxin was removed from the solution. Endotoxin levels were tested using ToxinSensor (GenScript) and are below 0.25 EU/mL. The activity of each subtype was determined using the human ISRE-Luc reporter cell line, a retinal pigment epithelial cell line transfected with a plasmid containing the Firefly Luciferase gene, stably integrated under control of the ISRE. Following stimulation with IFNα, chemiluminescence can be detected and used to calculate the respective activity in units against commercially available IFNα (PBL Assay Science) (7). Protein concentrations (in milligrams per milliliter) and specific activities (in units per milliliter) are shown in Table 2.

**Table 2.** Concentration and specific activity of IFNα preparations used in this study

Human IFNα	Nanodrop 2000 Spectrophotometer, concentration (mg/mL)	RPE-ISRE-Luc reporter cells, specific activity (U/mg)
1	0.21	3.24 × 10^9^
2	1.11	7.06 × 10^7^
4	0.21	4.20 × 10^8^
5	1.79	1.12 × 10^7^
6	0.17	2.56 × 10^7^
7	0.12	2.42 × 10^7^
8	0.62	3.29 × 10^6^
10	0.17	3.42 × 10^8^
14	0.35	1.63 × 10^8^
16	0.32	4.67 × 10^6^
17	0.21	4.50 × 10^8^
21	0.19	2.32 × 10^8^

### End-Point Dilution Assay.

VeroE6 cells were seeded at a density of 10,000 cells per well in a 96-well plate and maintained in 200 µL of DMEM supplemented with 10% FBS, L-glutamine, and penicillin and streptomycin overnight. The next day, 22 µL of virus stock or apical washes of hAEC were added to the first row of the plate (six replicates). Then, the virus was diluted 1:10 by mixing the media and pipetting 22 µL to the next row repeatedly, followed by 72 h incubation in 37 °C in a 5% CO_2_ atmosphere. Thereafter, the supernatant was aspirated, and the cells were incubated in 100 µL of crystal violet solution (0.1% crystal violet [Roth] in phosphate-buffered saline [PBS], 10% ethanol, 0.37% formalin) for 5 min. Subsequently, the crystal violet solution was aspirated, cells were washed with PBS, and the number of wells with intact or damaged cell layer was determined. The TCID_50_ per milliliter was calculated by the Spearman & Kärber algorithm.

### IFN Titration Assay.

VeroE6 cells were seeded at a density of 10,000 cells per well in a 96-well plate and maintained in DMEM supplemented with 10% FBS, L-glutamine, penicillin, and streptomycin overnight. Then, the medium was aspirated, and serially diluted IFNα subtypes and IFNλ3 (R&D Systems) and virus with a final concentration of 350 PFU/mL were added to the cells in a total volume of 100 µL of cell culture media, followed by 72 h incubation in 37 °C in a 5% CO_2_ atmosphere. Thereafter, the supernatant was aspirated, and the cells were stained with 100 µL of crystal violet solution (0.1% crystal violet in PBS, 10% ethanol, 0.37% formalin) for 5 min. Subsequently, the crystal violet solution was aspirated, cells were washed with PBS, and the number of wells with intact or damaged cell layer was determined.

The IC_50_ was calculated using GraphPad Prism 6.

### icELISA.

The icELISA was performed based on the previously published protocol (33). Briefly, VeroE6 cells were seeded at a density of 20,000 cells per well in a 96-well plate and maintained in DMEM supplemented with 10% FBS, L-glutamine, penicillin, and streptomycin. At indicated time points, the medium was aspirated, and serially diluted IFNα subtypes or the indicated concentrations of remdesivir and virus with a final concentration of 350 PFU/mL were added to the cells in a total volume of 100 µL, followed by 24 h incubation in 37 °C in a 5% CO_2_ atmosphere. Thereafter, 100 µL of 8% ROTIHistofix (Roth) (equals 4% of total paraformaldehyde [PFA]) were added for a minimum of 2 h at room temperature to fix the cells and inactivate the virus.

Afterward, the plate was washed thrice with PBS. The PBS was aspirated, 200 µL of freshly prepared permeabilization buffer (PBS, 1% Triton X-100 [Roth]) were added to the cells, and the plate was incubated for 30 min at room temperature under constant shaking. Subsequently, the permeabilization buffer was aspirated, and 200 µL of blocking buffer (PBS, 3% FBS) were added for 1 h. Then, the blocking buffer was aspirated, and 50 µL of primary antibody solution (anti-SARS-CoV-2-NP [RRID: AB_2890255] 1:5,000 diluted in PBS + 1% FBS) was added to each well. The plate was incubated overnight at 4 °C. The next day, the primary antibody solution was aspirated, and the plate was washed thrice with wash buffer (PBS, 0.05% Tween 20 [Roth]). Thereafter, 50 µL of the secondary antibody solution (Peroxidase-AffiniPure Goat Anti-Mouse IgG [H+L] [RRID: AB_10015289] 1:2,000 in PBS, 1% FBS) was added to the wells, and the plate was incubated for 2 h at room temperature. After the incubation period, the wells were washed four times with 250 µL of wash buffer. Afterward, 100 µL of tetramethylbenzidine (TMB) substrate solution (BioLegend) were added, and the plate was incubated for about 20 min at room temperature in the dark. The reaction was stopped by addition of 100 µL of 2N H_2_SO_4_ (Roth). The absorbance was measured at 450 nm with a reference wavelength of 620 nm using Spark 10M multimode microplate reader (Tecan).

### Cell Viability Assay.

To exclude cytotoxic effects of the compounds used in our assays, a cell viability assay was performed using the Orangu Cell Counting Solution (CELL Guidance Systems) according to the manufacturer’s instructions. The cells were seeded and treated equally to the protocol that was used before without any viral infection. Afterward, 10 µL of Orangu Cell Counting Solution were added to each well, and the plate was incubated for 2 h. Then, the absorbance was measured at 450 nm with Spark 10M multimode microplate reader.

### Immunofluorescence.

VeroE6 cells were seeded and treated as described for the icELISA. After incubation with the primary antibody solution, 50 µL of secondary antibody solution (Goat IgG anti-Mouse IgG [H+L]-Alexa Fluor 488, MinX none 1:2,000 [RRID: AB_2338840], Phalloidin CF647 1:100 [Biotium] in PBS + 1% FBS) were added to each well, and the plate was incubated for 2 h at room temperature. Thereafter, the secondary antibody solution was aspirated, and the cells were counterstained for 20 min at room temperature with 50 µL of DAPI solution (0.1 µg/mL DAPI [Sigma-Aldrich] in PBS). Subsequently, the plate was washed thrice with PBS and microscopically analyzed using Leica THUNDER Imager 3D Cell Culture.

### Ethics Statement.

Fetal tissues for reconstitution of humanized mice were obtained through anonymous donations with informed written consent via Advanced Bioscience Resources under the University of Saskatchewan Research Ethics Board Bio ID-371. All animal studies were performed under University of Saskatchewan’s Animal Research Ethics Board protocols 20180079 and 20200016 and adhered to Canadian Council on Animal Care guidelines.

### Humanized TKO-LoM Mice.

LoM were generated as previously described (64, 65) except for the use of the C56BL/6 Rag2^−/−^γc^−/−^CD47^−/−^ TKO mouse as the immunocompromised recipient mouse strain. Briefly, two pieces (∼2 mm^3^ to 4 mm^3^) of 17- to 22-wk-gestation human fetal lung (Advanced Bioscience Resource) were implanted subcutaneously onto the backs of mice. Subcutaneous wounds were closed with surgical glue. Lung organoids were allowed to grow to ∼1 cm in diameter prior to use in experiments. Mice were generated from three donors.

### SARS-CoV-2 Infection and IFNα Treatment of TKO-LoM.

The B.1.1.7 (alpha) variant was isolated on Vero76 cells (ATCC) from a clinical specimen kindly provided by Graham Tipples and Kanti Pabbaraju at Alberta Health Services, Edmonton, AB, Canada. The virus was subsequently propagated, and a p.2 stock was generated, titrated on Vero76 cells by conventional TCID_50_ assay, and sequenced. The virus stock was clarified by centrifugation at 4,700 × *g* for 10 min and stored at −80 °C until thawed for infections. The TKO-LoM mice were infected by direct injection of SARS-COV-2 (1 × 10^5^ TCID_50_ in 50 µL) or vehicle control into each human lung organoid. Mice received daily intraperitoneal injections of 1.5 × 10^5^ U of IFNα2 or IFNα5 or vehicle control for 4 d starting 2 h postchallenge. Mice were killed on day 5, and lung organoids were harvested for infectious virus assay.

### Lung Organoid Viral Loads.

Lung organoids were weighed and placed in 1 mL of DMEM supplemented with 1% heat-inactivated FBS, 1× L-glutamine before being homogenized in a Tissuelyser II Homogenizer (Qiagen) at 30 Hz for 6 min. Tissue homogenates were clarified by centrifugation at 5,000 × *g* for 5 min and then serially diluted 10-fold in DMEM supplemented with 2% heat-inactivated FBS and 2× penicillin–streptomycin. Sample volumes of 50 µL were added to 96-well plates of 95% confluent Vero76 cells in triplicate and incubated at 37 °C with 5% CO_2_ before scoring for the presence of cytopathic effects.

### Infection of hAEC.

The hAEC were obtained from lung transplant donors postmortem (ethics of University Duisburg-Essen 18-8024-BO and 19-8717-BO) or from explanted lungs (ethics of Hannover Medical School 3346/2016). Selection criteria for donors are listed in the Eurotransplant guidelines. The hAECs from explanted lungs were cultured and differentiated as previously described (66). The hAEC from lung transplant donors postmortem were obtained by the following protocol: During the adaptation of the donor lung, a small tracheal ring was removed and stored in PBS supplemented with antibiotics (penicillin 100 U/mL, streptomycin 100 µg/mL, 10 µg/mL ciprofloxacin [Kabi]). The hAEC were isolated from the mucosa within 24 h after transplantation by enzymatic digestion (Protease XIV [Sigma-Aldrich]) and scraping. Cells were expanded for 7 d to 14 d in KSFM (keratinocyte-SF-medium [Gibco], supplemented with human epidermal growth factor [Gibco] [2.5 ng/mL], bovine pituitary extract [Gibco] [BPE 25 µg/mL, Gibco], isoproterenol [Sigma-Aldrich] [1 µM], penicillin, streptomycin, ciprofloxacin, and amphotericin B [PanBiotech] [2.5 µg/mL]) and, after trypsinization, stored in liquid nitrogen (10% dimethyl sulfoxide, 90% KSFM+BPE 0.3 mg/mL). All plastic surfaces during hAEC isolation and air–liquid interface (ALI) culture were coated with human fibronectin (PromoCell) (5 µg/mL), type I bovine collagen (Advanced BioMatrix) (PureCol 30 µg/mL), and bovine serum albumin (BSA) (10 µg/mL). For ALI cultures, cells were thawed, expanded in KSFM for 5 d to 7 d, and transferred to transwell inserts (PE Membrane, 12-well plates, 0.4-µm pore size, Corning). A monolayer of hAECs were grown submerged in S/D Media (1:1 mixture of DMEM [StemCell] and BEpiCM-b [ScienCell]), supplemented with penicillin and streptomycin, Hepes (Gibco) (12.5 mL/l, 1 M), 1× Bronchial Epithelial Cell Growth Supplement (ScienCell), and EC-23 (Tocris) (5 mM) until they reached confluency. Apical media was removed, and cell differentiation was induced under air exposure for 2 wk. Infection was started after cells were fully differentiated as measured by movement of cilia, secretion of mucus, and transepithelial electrical resistance (>1,000 Ω/cm^2^).

Fully differentiated hAECs were washed with Hanks’ balanced salt solution (HBSS) apically for 10 min before infection. For SARS experiments, the cells were infected apically with 30,000 PFU diluted in HBSS; for influenza, the cells were apically infected with IAV H1H1 strain A/Puerto Rico/34 (PR8) at 0.1 multiplicity of infection in 200 µL of HBSS. The cells were incubated with the inoculum for 1 h in 33 °C in a 5% CO_2_ atmosphere. Thereafter, the inoculum was aspirated, and the cells were washed thrice with 150 µL of HBSS for 10 min. The last wash was collected and stored at −80 °C as a 0-h sample. At the indicated time points, cells were washed apically for 10 min, and the washes were subjected to an end-point dilution assay or to a plaque titration assay as described for SARS-CoV-2 and influenza, respectively.

Treatment of hAECs was performed by adding the indicated amounts of IFNs or remdesivir directly to the cell culture medium on the basolateral side of the cells.

For the isolation of RNA, cells were lysed using Qiagen RLT buffer (Qiagen) supplemented with 1% β-mercaptoethanol (Sigma-Aldrich).

### Viral Messenger RNA Quantification.

Total RNA was purified from hAECs and VeroE6 cells using the RNeasy Mini Kit (Qiagen) according to manufacturer’s instructions with preceding DNase I digestion with the RNase-Free DNase Set (Qiagen).

To determine relative SARS-CoV-2 *M*- or *N*-gene expression, 500 ng of total RNA were reverse transcribed using the PrimeScript RT Master Mix (Takara). Promega’s GoTaq Probe qPCR Master Mix was used according to the manufacturer’s instructions, with gene-specific primers and probes (*SI Appendix*, Table S4). RT-qPCR was performed on a LightCycler 480 II (Roche) instrument, with the following conditions: Initial denaturation was 2 min at 95 °C and a ramp rate of 4.4 °C/s, followed by 40 cycles of denaturation for 15 s at 95 °C and a ramp rate of 4.4 °C/s and amplification for 60 s at 60 °C and a ramp rate of 2.2 °C/s. To assess *M*- and *N*-gene copy numbers, the *M-* and *N-*genes were partially cloned into pCR2.1 (ThermoFisher Scientific) or pMiniT 2.0 (NEB), respectively, and a 1:10 plasmid dilution series was used as a reference.

### IAV Plaque Assay.

MDCK-II cells were seeded in six-well plates, and cultured in DMEM supplemented with 5% FBS and 1% penicillin–streptomycin until 100% confluent. On the day of infection, 10-fold dilutions of apical washes were prepared in infection PBS (PBS supplemented with 1% penicillin–streptomycin, 0.01% CaCl_2_, 0.01% MgCl_2_, and 0.2% BSA). Cells were washed once with infection PBS, infected with 500 µL of diluted samples (virus inoculum), and incubated at 37 °C, 5% CO_2_ for 30 min. The inoculum was removed, and the infected monolayer was overlaid with plaque medium (prepared immediately before use by mixing 14.2% 10× MEM [Gibco], 0.3% NaHCO_3_, 0.014% DEAE-Dextran [Sigma-Aldrich], 1.4% 100× penicillin–streptomycin, 0.3% BSA, 0.9% agar, 0.01% MgCl_2_, 0.01% CaCl_2_, and 0.15 mg of TPCK-Trypsin [Sigma]). Plates were kept at room temperature until the agar solidified, and were incubated upside down at 37 °C, 5% CO_2_ for 72 h. Plaques were quantified in terms of infectious IAV particles, and were represented as plaque-forming units per milliliter.

### ISG Expression.

The 500,000 VeroE6 cells were seeded and stimulated with 1,000 U/mL of IFNα1, IFNα5, and IFNα16 for 16 h. Afterward, the cells were lysed using DNA/RNA Shield for RNA isolation.

RNA was isolated from cell lysates with Quick-RNA Miniprep Kit (Zymo Research) according to the manufacturer’s instruction.

Complementary DNA was synthesized from isolated RNA using HiScript II RT SuperMix for qPCR (Vazyme) according to the manufacturer’s instructions. ISG expression levels were quantified by qPCR with Luna Universal qPCR Master Mix and the respective primer pairs (*SI Appendix*, Table S5). Expression levels were normalized by 2^-ΔΔCT^ method (67) using GAPDH as reference gene.

### Proteomics Sample Preparation.

Cells were washed with ice-cold PBS and harvested in urea buffer (30 mM Tris HCl, 7 M Urea, 2 M Thiourea, 0.1% NaDOC, pH 8.5). Cells were centrifuged for 15 min at 16,100 × *g* and 4 °C, and the supernatant was further processed.

Tryptic digestion was performed on 20 µL of cell lysate. Disulfide bonds were reduced by adding a final 5 mM dithiothreitol for 15 min at 50 °C before thiols were alkylated by a final 15 mM IAA (iodoacetamide) for 15 min in the dark. Hydrophilic and hydrophobic Cytiva Sera-Mag Carboxyl-Magnet-Beads (GE Healthcare) were mixed 1:1, and 2 µL of beads (25 µg/µL) were added per sample. The samples were filled up to 70% ACN (acetonitrile) and incubated for 15 min to ensure protein binding to the beads. Subsequently, beads were washed two times with 70% EtOH followed by washing with 100% ACN. Beads were resuspended in 100 mM ammonium bicarbonate carbonate containing 0.2 µg of trypsin (SERVA) per sample and incubated overnight at 37 °C. The peptides were transferred into a new reaction tube, vacuum dried, and dissolved in 0.1% trifluoroacetic acid.

### Liquid Chromatography–Tandem Mass Spectrometry (LC-MS/MS Analysis).

Then 400 ng of tryptic peptides per sample were analyzed using an Ultimate 3000 RSLCnano HPLC (Dionex) coupled to a Q Exactive HF Orbitrap (Thermo Fisher Scientific). Samples were preconcentrated on a C18 trap column (Acclaim PepMap 100; 100 μm × 2 cm, 5 μm, 100 Å; Thermo Fisher Scientific) within 7 min at a flow rate of 30 μL/min with 0.1% trifluoric acid and subsequently transferred to a Nano Viper C18 analytical column (Acclaim PepMap RSLC; 75 μm × 50 cm, 2 μm, 100 Å; Thermo Fisher Scientific). Peptide separation was performed by a gradient from 5 to 30% solvent B over 120 min at 400 nL/min (solvent A: 0.1% formic acid; solvent B: 0.1% formic acid, 84% acetonitrile). Full-scan mass spectra were acquired in profile mode at a resolution of 70,000 at 400 *m/z* within a mass range of 350 *m/z* to 1,400 *m/z*. The 10 highest abundant peptide ions were fragmented by higher-energy collisional dissociation (normalized collision energy = 27), and MS/MS spectra were acquired at a resolution of 35,000.

### Proteomics Data Analysis.

Peptide identification and quantification were performed using MaxQuant (v.1.6.17) searching UniProtKB/SwissProt (2020_05, 563,552 entries) restricted to either *Homo sapiens* or *Homo sapiens* and SARS-CoV-2. Search parameters were default, label-free quantification was used for peak quantification, and normalization was enabled. Peptides were considered for quantification irrespective of modifications. Match between runs was enabled when the analysis was performed considering human proteins only. Statistical data analysis was conducted using R (v.3.6.2). Differences between the experimental groups were assessed using *t* tests (paired, two-sided), and proteins quantified in a minimum of three out of four donors per group with a minimum of two unique peptides, a *P* value ≤ 0.05, and a ratio of mean abundances ≥ 1.5 or ≤ 0.67 were considered statistically significant. Proteins that were quantified in one experimental group but not detected at all in an opposed group were defined as On-Offs between these groups. GO annotation and enrichment analyses were performed using STRING (v.11). Data visualization was done using R and Cytoscape (v.3.8.2).

### Transcriptomics.

Quality and integrity of total RNA was controlled on 5200 Fragment Analyzer System (Agilent Technologies)). The RNA sequencing library was generated from 50 ng of total RNA using NEBNext Single Cell/Low Input RNA Library to manufactureŕs protocols. The libraries were treated with Illumina Free Adapter Blocking and were sequenced on Illumina NovaSeq 6000 using NovaSeq 6000 S1 Reagent Kit (100 cycles, paired end run 2 × 50 bp) with an average of 3 × 10^7^ reads per RNA sample.

### Transcriptomic Analysis.

FASTQ files of RNA sequencing files were imported into the Array Studio software v10.2.5.9 (QIAGEN) package for further data analysis. All FASTQ files were aligned to the gene model Ensembl v96 and to the reference library Human B38 using the proprietary OmicSoft Aligner OSA (68). Differential gene expression of each condition was assessed using DESeq2 (69). DEGs were sent to IPA (https://digitalinsights.qiagen.com/products-overview/discovery-insights-portfolio/analysis-and-visualization/qiagen-ipa/) for biological analysis using the cutoffs *P* value < 0.05, fold change (fc) > |1.5|, and mean counts min > 5. IPA statistics is based on two outputs. A *P* value derived from a right-tailed Fisher’s exact test estimates the probability that the association between a function or pathway and a set of molecules might be due to random chance, but does not consider directional changes. This is, however, predicted for a disease and/or function, canonical pathway, or upstream regulator (activation or inhibition) by the activation z-score algorithm. The z score describes the number of SDs data lie above or below the mean. A z score > 2 was considered significantly increased, whereas a z score < −2 was considered significantly decreased (70). We performed an expression analysis to evaluate transcriptomic changes for canonical pathways in each of the comparison IFN vs. mock (70).

### Statistical Analysis.

Differences in transformed data were tested for significance using GraphPad Prism v8.4.2 for Windows (GraphPad). Statistically significant differences between the IFNα-treated groups and the untreated group were analyzed using nonparametric Kruskal–Wallis test with Dunn’s multiple comparison test. *P* values < 0.05 were considered significant.

## Supplementary Material

Supplementary File

## Data Availability

The authors declare that the data supporting the findings of this study are available within the article and *SI Appendix*. The mass spectrometry proteomics data have been deposited to the ProteomeXchange Consortium via the PRIDE partner repository with the dataset identifier PXD026079. The RNA sequencing data discussed in this publication have been deposited in National Center for Biotechnology Information’s Gene Expression Omnibus (GEO) (71) and are accessible through GEO Series accession number GSE189613.
