# Supplementary material for: Differential interferon-α subtype induced immune signatures are associated with suppression of SARS-CoV-2 infection
Source: Proc Natl Acad Sci U S A. 2022 Feb 7;119(8):e2111600119. doi: 10.1073/pnas.2111600119 (PMC8872780; doi:10.1073/pnas.2111600119)
Supplement: Supplementary File [file pnas.2111600119.sapp.pdf]

PNAS
www.pnas.org

Supplementary Information for

Differential interferon-α subtype induced immune signatures are associated with suppression of SARS-CoV-2 infection

Jonas Schuhenn, Toni Luise Meister, Daniel Todt, Thilo Bracht, Karin Schork, Jean-Noel Billaud, Carina Elsner, Natalie Heinen, Zehra Karakoese, Sibylle Haid Sriram Kumar, Linda Brunotte, Martin Eisenacher, Yunyun Di, Jocelyne Lew, Darryl Falzarano, Jieliang Chen, Zhenghong Yuan, Thomas Pietschmann, Bettina Wiegmann, Hendrik Beckert, Christian Taube, Vu Thuy Khanh Le-Trilling, Mirko Trilling, Adalbert Krawczyk, Stephan Ludwig, Barbara Sitek, Eike Steinmann, Ulf Dittmer, Kerry J. Lavender, Kathrin Sutter and Stephanie Pfaender

Corresponding authors:
Kerry J. Lavender
Email: kerry.lavender@usask.ca

Kathrin Sutter
Email: Kathrin.sutter@uni-due.de

Stephanie Pfaender
Email: Stephanie.pfaender@rub.de

This PDF file includes:

Figures S1 to S8
Legends for Figures S1 to S8
Tables S1 to S5

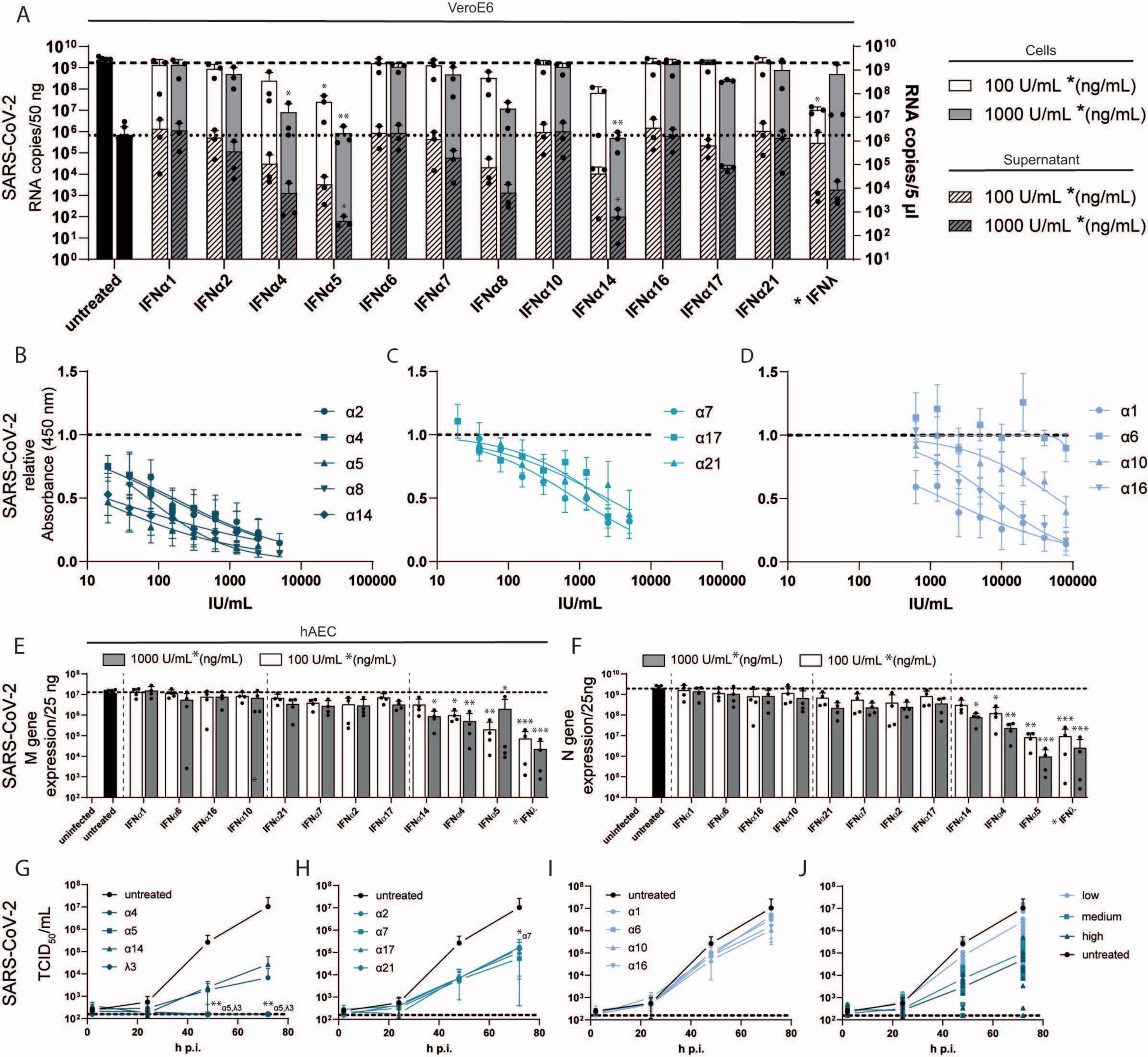
A
VeroE6
SARS-CoV-2
RNA copies/50 ng
RNA copies/5 µl
Cells
100 U/mL *(ng/mL)
1000 U/mL *(ng/mL)
Supernatant
100 U/mL *(ng/mL)
1000 U/mL *(ng/mL)
untreated
IFNα1
IFNα2
IFNα4
IFNα5
IFNα6
IFNα7
IFNα8
IFNα10
IFNα14
IFNα16
IFNα17
IFNα21
* IFNλ
B
C
D
SARS-CoV-2
relative
Absorbance (450 nm)
IU/mL
α2
α4
α5
α8
α14
α7
α17
α21
α1
α6
α10
α16
E
hAEC
1000 U/mL*(ng/mL)
100 U/mL *(ng/mL)
SARS-CoV-2
M gene
expression/25 ng
F
N gene
expression/25ng
uninfected
untreated
IFNα1
IFNα6
IFNα16
IFNα10
IFNα21
IFNα7
IFNα2
IFNα17
IFNα14
IFNα4
IFNα5
* IFNλ
G
H
I
J
SARS-CoV-2
TCID50/mL
h p.i.
untreated
α4
α5
α14
λ3
**α5,λ3
α2
α7
α17
α21
*α7
α1
α6
α10
α16
low
medium
high
untreated

Supplementary Fig. 1: Treatment with IFNα subtypes reveals distinct antiviral effects against SARS-CoV-2

(A) Antiviral activity of IFNα subtypes and IFNλ3 against SARS-CoV-2 were analysed in VeroE6 cells and cell supernatant by qRT-PCR. IFNα subtypes were titrated against SARS-CoV-2 on VeroE6 cells by ic-ELISA assay and the IFNs were grouped as mean values in high (B), medium (C) and low (D) antiviral pattern.

Antiviral activity of IFNα subtypes and IFNλ3 in SARS-CoV-2-infected primary hAECs at 72 h p.i. determined by qRT-PCR analysis of *M gene* (E) and *N gene* (F). Kinetics of the antiviral activity of IFNs by $TCID_{50}$ assay grouped into high (G), medium (H) and low (I) antiviral pattern and the mean values of each group are plotted in (J). Mean values of high (I) and low/not (J) antiviral IFNs are shown. Mean values ± SEM are shown. (A-D) n=3 (E-J) n=4.

* $p<0.05$; ** $p<0.01$; *** $p<0.001$.

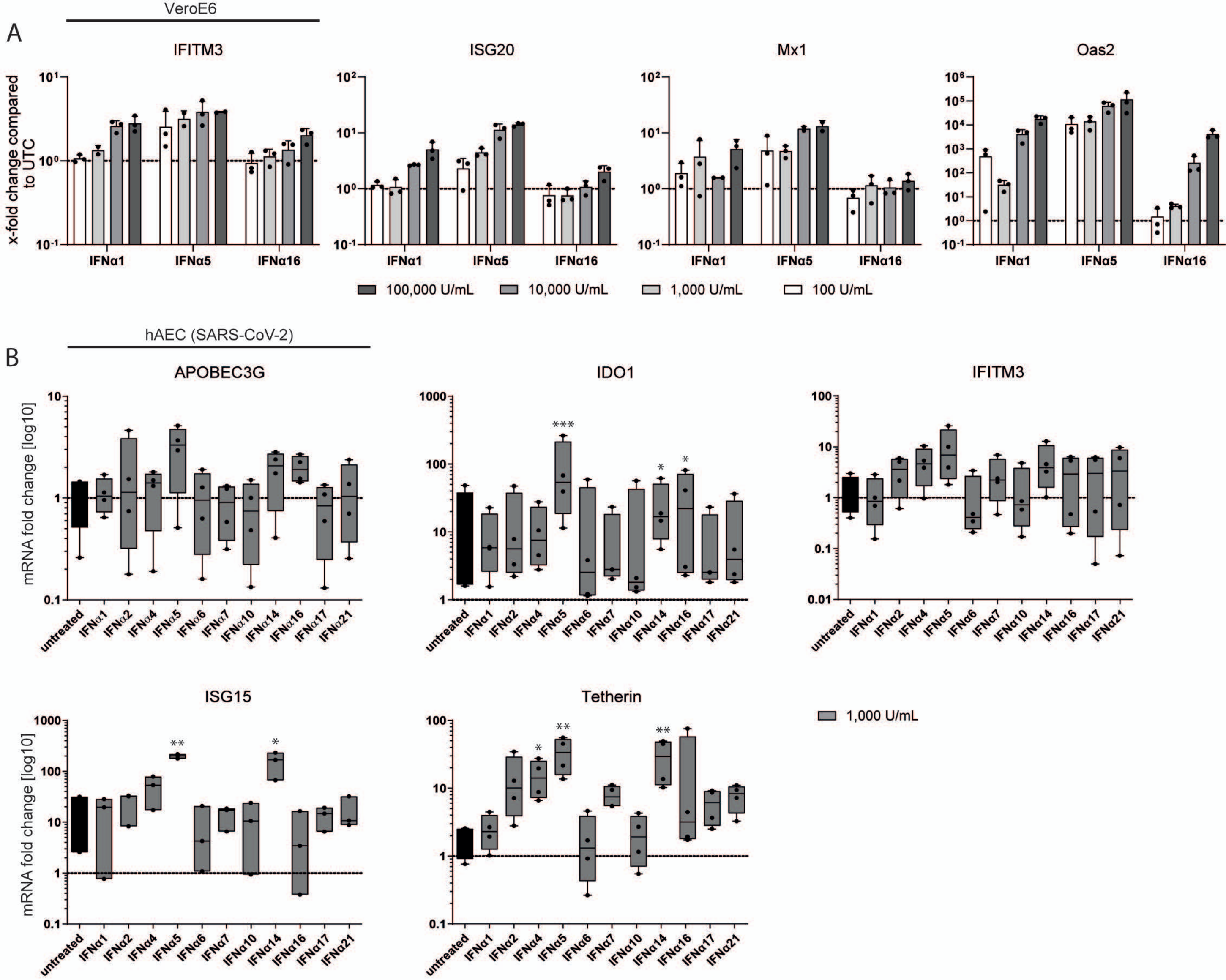

VeroE6
A
IFITM3
ISG20
Mx1
Oas2
x-fold change compared to UTC
IFNα1
IFNα5
IFNα16
100,000 U/mL
10,000 U/mL
1,000 U/mL
100 U/mL
hAEC (SARS-CoV-2)
B
APOBEC3G
IDO1
IFITM3
ISG15
Tetherin
mRNA fold change [log10]
untreated
IFNα1
IFNα2
IFNα4
IFNα5
IFNα6
IFNα7
IFNα10
IFNα14
IFNα16
IFNα17
IFNα21
1,000 U/mL

Supplementary. Fig. 2: ISG induction upon IFNα subtype stimulation

(A) mRNA expression of different ISGs in IFN-treated VeroE6 cells at 16 h p. stimulation determined by qRT-PCR analysis. (B) mRNA expression of different ISGs in IFN-treated SARS-CoV-2-infected primary hAECs at 72 h p.i. determined by qRT-PCR analysis. Mean values of mRNA expression is shown as fold change compared to untreated control. (A) n=3, (B) n=4. B: * $p<0.05$; ** $p<0.01$; *** $p<0.001$

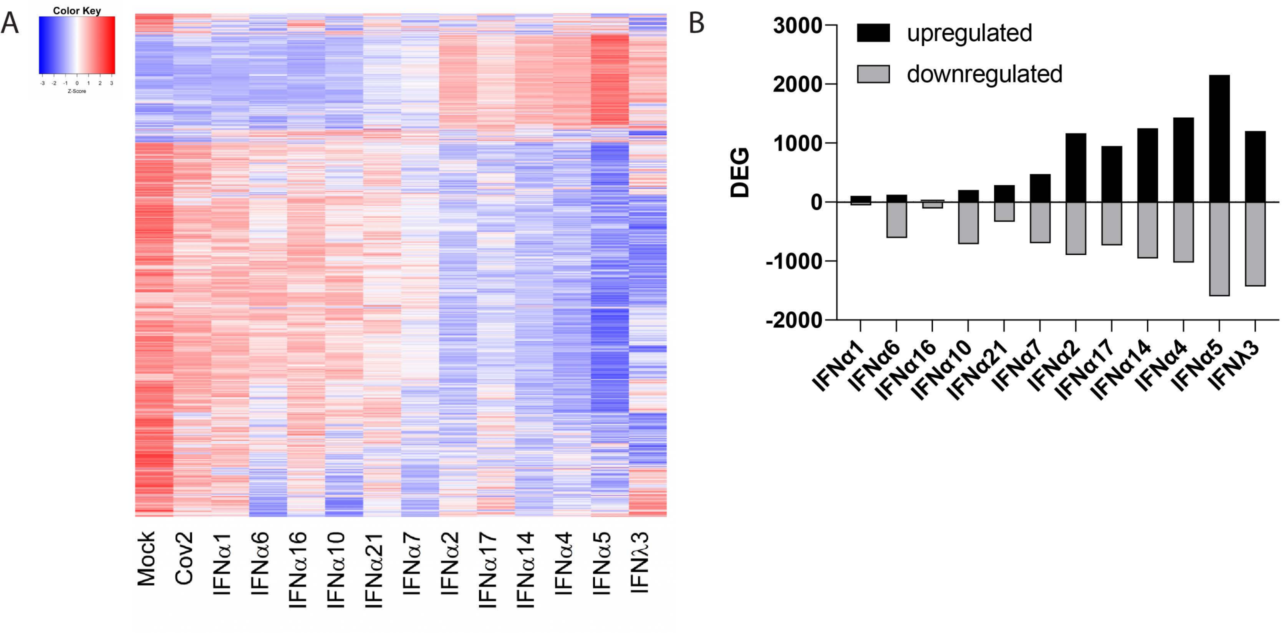
A
Color Key
Z-Score
-3 -2 -1 0 1 2 3
Mock
Cov2
IFNα1
IFNα6
IFNα16
IFNα10
IFNα21
IFNα7
IFNα2
IFNα17
IFNα14
IFNα4
IFNα5
IFNλ3
B
upregulated
downregulated
DEG
3000
2000
1000
0
-1000
-2000
IFNα1
IFNα6
IFNα16
IFNα10
IFNα21
IFNα7
IFNα2
IFNα17
IFNα14
IFNα4
IFNα5
IFNλ3

Supplementary Fig. 3: Transcriptomic analysis display IFNα subtype specific gene signatures

(A) Transcriptomic analyses of IFN-treated (16 hours post-treatment) or SARS-CoV-2-infected (18 hours post infection) hAECs. Heat maps displaying differentially expressed genes (DEG) from at least one comparison of an IFN vs. Mock. (B) Numbers of up- and downregulated DEGs of IFN-treated compared to untreated hAECs (4 donors) shown as bars.

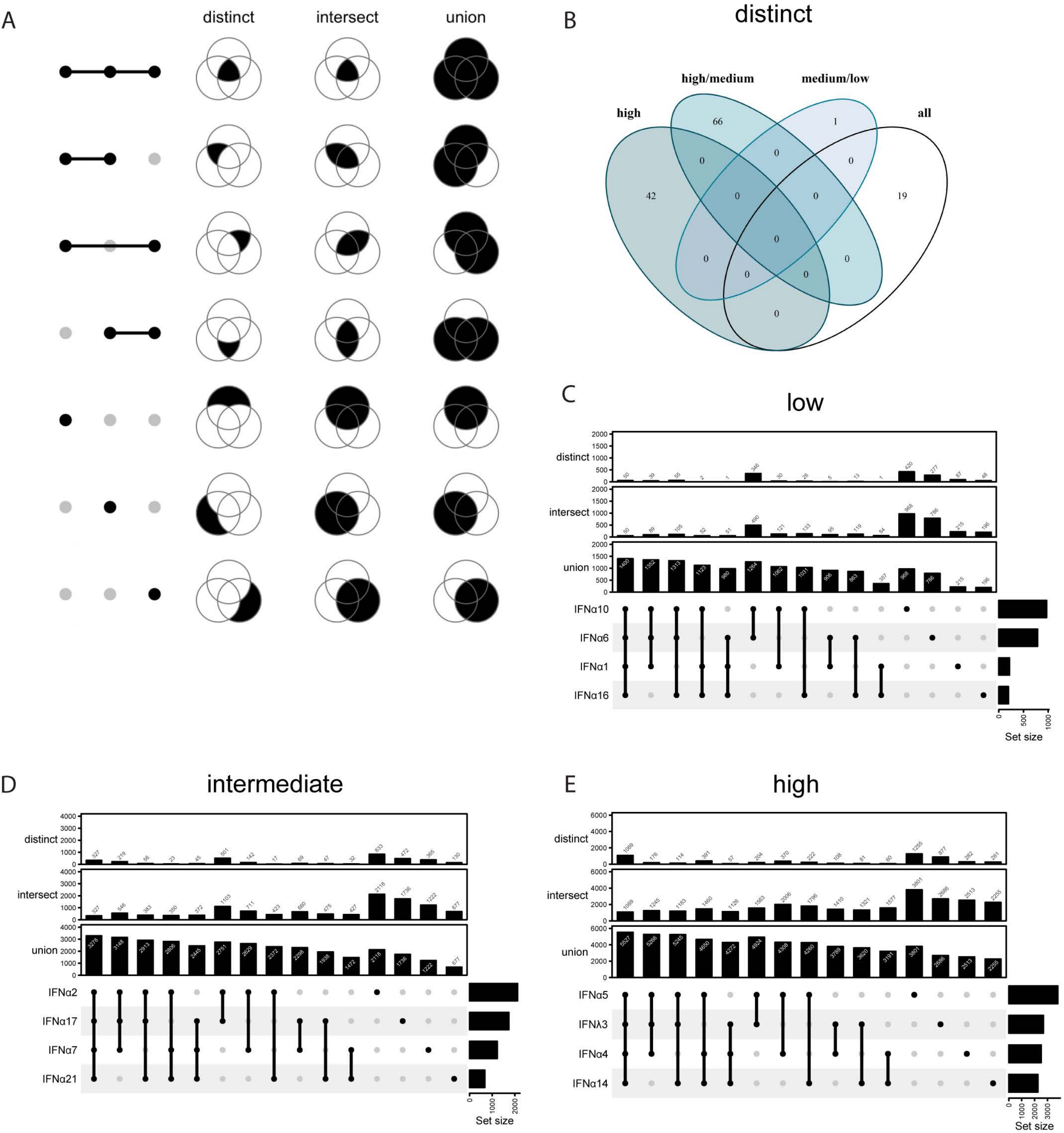
A
distinct
intersect
union
B
distinct
high
high/medium
medium/low
all
66
1
42
0
19
C
low
distinct
intersect
union
IFNα10
IFNα6
IFNα1
IFNα16
Set size
D
intermediate
distinct
intersect
union
IFNα2
IFNα17
IFNα7
IFNα21
Set size
E
high
distinct
intersect
union
IFNα5
IFNλ3
IFNα4
IFNα14
Set size

Supplementary Fig. 4: **Transcriptomic analysis reveal different patterns of distinct, intersect and union genes**

(A-E) UpSet plots to summarize distinct, intersect and union differentially expressed genes (DEG) of IFN-treated (16 hours post-treatment) hAECs (4 donors). (A) Schematic depiction of distinct, intersect and union DEGs. (B) Venn diagram of distinct DEGs expressed by all high, medium and low antiviral IFNs. (C) UpSet plots showing distinct, intersect and union DEGs of low (C), medium (D) and high (E) antiviral IFNs. Numbers of individually or group-specific DEGs are shown as bars and numbers. The bottom right horizontal bar graph labelled Set Size shows the total number of DEGs per treatment.

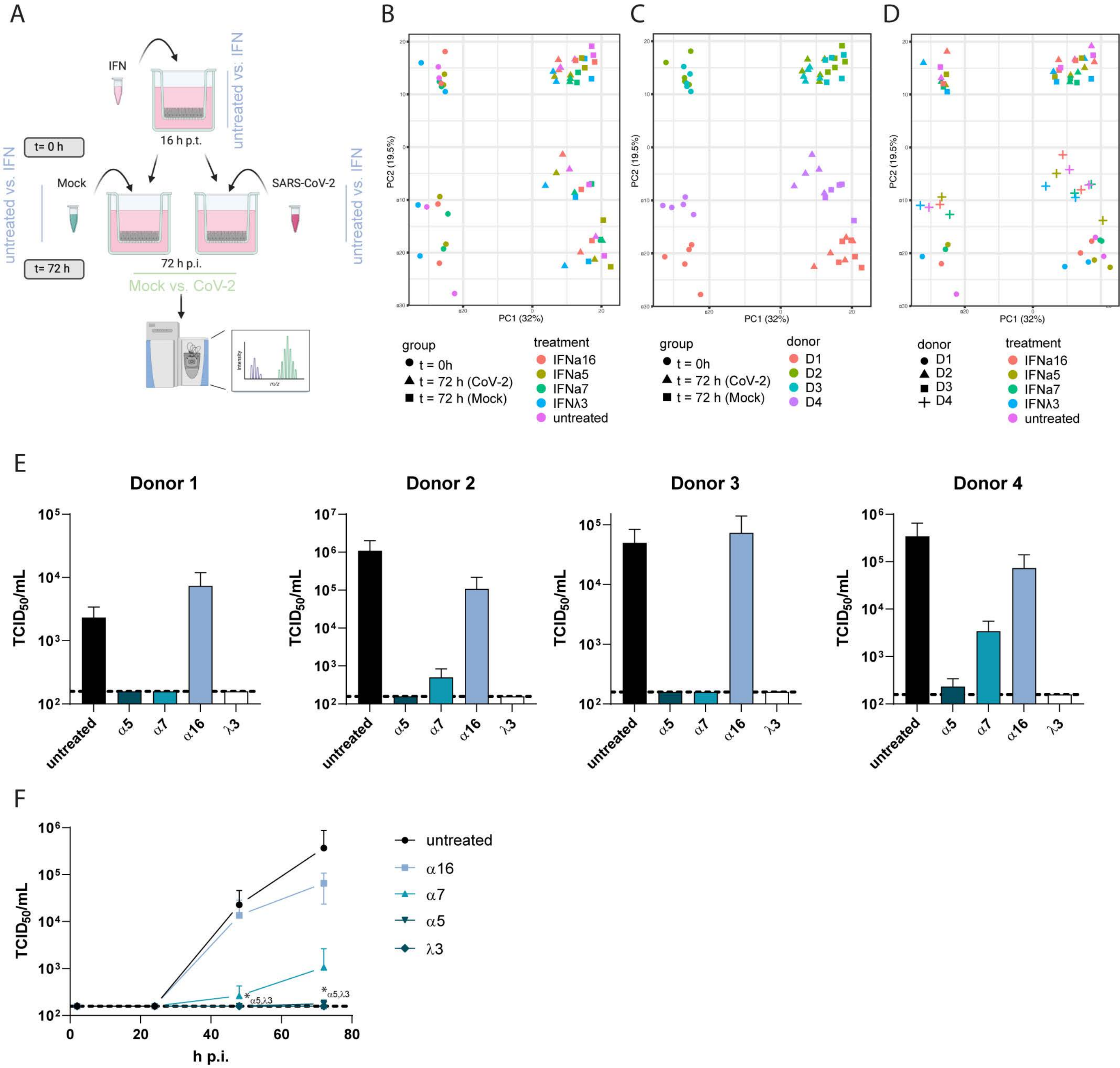

A
IFN
16 h p.t.
untreated vs. IFN
t= 0 h
Mock
SARS-CoV-2
t= 72 h
72 h p.i.
Mock vs. CoV-2
B
C
D
PC1 (32%)
PC2 (19.5%)
group
t = 0h
t = 72 h (CoV-2)
t = 72 h (Mock)
treatment
IFNa16
IFNa5
IFNa7
IFNλ3
untreated
donor
D1
D2
D3
D4
E
Donor 1
Donor 2
Donor 3
Donor 4
TCID50/mL
untreated
α5
α7
α16
λ3
F
untreated
α16
α7
α5
λ3
*α5,λ3
h p.i.

Supplementary Fig. 5: Proteomic analysis highlights key cellular mediators

(A-D) Proteomic analysis of IFN-treated and/or SARS-CoV-2-infected hAECs. (A) Schematic depiction (B-D) Principal component analysis (PCA) of hAEC proteomics. (B) The first two principal components (PCs) are plotted and shaped/coloured according to group and IFN-treatment (B); to group and individual donors (C) or to individual donors and IFN-treatment (D). PCA was performed using all proteins without missing values. Percentage of variation accounted for by each principal component is shown in brackets with the axis label. (E) Antiviral activity of IFNα subtypes and IFNλ3 in SARS-CoV-2-infected primary hAECs of 4 individual donors used for proteomic analysis at 72 h p.i. determined by $TCID_{50}$ assay. (F) Kinetics of the antiviral activity of selected IFNs by $TCID_{50}$ assay in SARS-CoV-2-infected primary hAECs of 4 individual donors used for proteomic analysis shown as mean values + SEM. D: 48h: *** $p<0.001$; **** $p<0.0001$

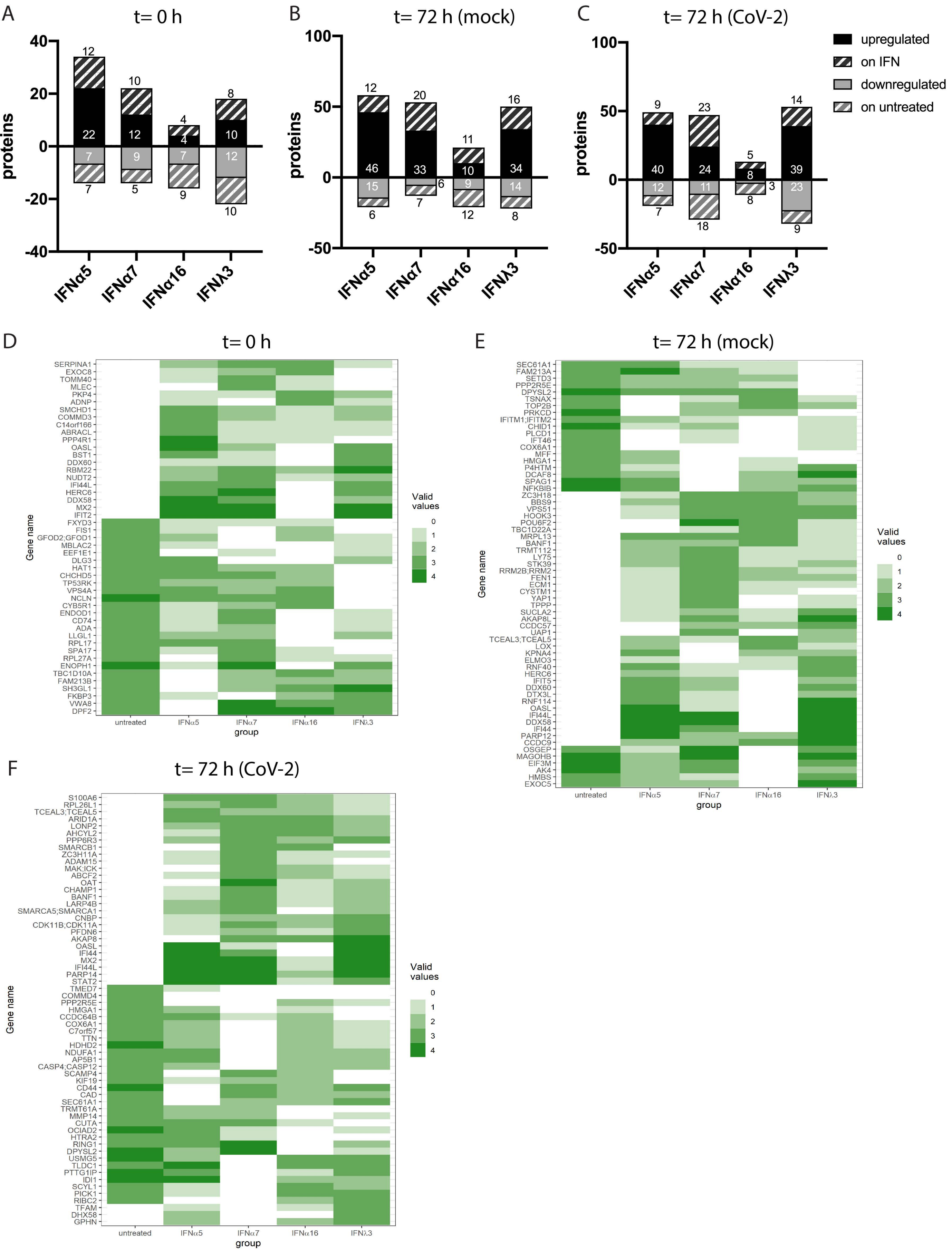

A
t= 0 h
proteins
40
20
0
-20
-40
12
22
7
7
10
12
9
5
4
4
7
9
8
10
12
10
IFNα5
IFNα7
IFNα16
IFNλ3
B
t= 72 h (mock)
proteins
100
50
0
-50
12
46
15
6
20
33
6
7
11
10
9
12
16
34
14
8
C
t= 72 h (CoV-2)
proteins
100
50
0
-50
9
40
12
7
23
24
11
18
5
8
3
8
14
39
23
9
upregulated
on IFN
downregulated
on untreated
D
t= 0 h
SERPINA1
EXOC8
TOMM40
MLEC
PKP4
ADNP
SMCHD1
COMMD3
C14orf166
ABRACL
PPP4R1
OASL
BST1
DDX60
RBM22
NUDT2
IFI44L
HERC6
DDX58
MX2
IFIT2
FXYD3
FIS1
GFOD2;GFOD1
MBLAC2
EEF1E1
DLG3
HAT1
CHCHD5
TP53RK
VPS4A
NCLN
CYB5R1
ENDOD1
CD74
ADA
LLGL1
RPL17
SPA17
RPL27A
ENOPH1
TBC1D10A
FAM213B
SH3GL1
FKBP3
VWA8
DPF2
Gene name
untreated
IFNα5
IFNα7
IFNα16
IFNλ3
group
Valid values
0
1
2
3
4
E
t= 72 h (mock)
SEC61A1
FAM213A
SETD3
PPP2R5E
DPYSL2
TSNAX
TOP2B
PRKCD
IFITM1;IFITM2
CHID1
PLCD1
IFT46
COX6A1
MFF
HMGA1
P4HTM
DCAF8
SPAG1
NFKBIB
ZC3H18
BBS9
VPS51
HOOK3
POU6F2
TBC1D22A
MRPL13
BANF1
TRMT112
LY75
STK39
RRM2B;RRM2
FEN1
ECM1
CYSTM1
YAP1
TPPP
SUCLA2
AKAP8L
CCDC57
UAP1
TCEAL3;TCEAL5
LOX
KPNA4
ELMO3
RNF40
HERC6
IFIT5
DDX60
DTX3L
RNF114
OASL
IFI44L
DDX58
IFI44
PARP12
CCDC9
OSGEP
MAGOHB
EIF3M
AK4
HMBS
EXOC5
Gene name
untreated
IFNα5
IFNα7
IFNα16
IFNλ3
group
Valid values
0
1
2
3
4
F
t= 72 h (CoV-2)
S100A6
RPL26L1
TCEAL3;TCEAL5
ARID1A
LONP2
AHCYL2
PPP6R3
SMARCB1
ZC3H11A
ADAM15
MAK;ICK
ABCF2
OAT
CHAMP1
BANF1
LARP4B
SMARCA5;SMARCA1
CNBP
CDK11B;CDK11A
PFDN6
AKAP8
OASL
IFI44
MX2
IFI44L
PARP14
STAT2
TMED7
COMMD4
PPP2R5E
HMGA1
CCDC64B
COX6A1
C7orf57
TTN
HDHD2
NDUFA1
AP5B1
CASP4;CASP12
SCAMP4
KIF19
CD44
CAD
SEC61A1
TRMT61A
MMP14
CUTA
OCIAD2
HTRA2
RING1
DPYSL2
USMG5
TLDC1
PTTG1IP
IDI1
SCYL1
PICK1
RIBC2
TFAM
DHX58
GPHN
Gene name
untreated
IFNα5
IFNα7
IFNα16
IFNλ3
group
Valid values
0
1
2
3
4

Supplementary Fig. 6: Proteomic analysis results in differential switched on/off proteins

Proteomic analysis of IFN-treated and/or SARS-CoV-2-infected hAECs. (A) Differentially regulated or induced (on IFN compared to untreated) proteins in IFN-stimulated hAECs at t=0h (A), at t=72h (mock) (B) or at t=72h (CoV-2) (C). Heatmaps of on-off regulated proteins in IFN-stimulated hAECs at t=0 h (D), at t=72 h (mock) (E) or at t=72 h (CoV-2) (F).

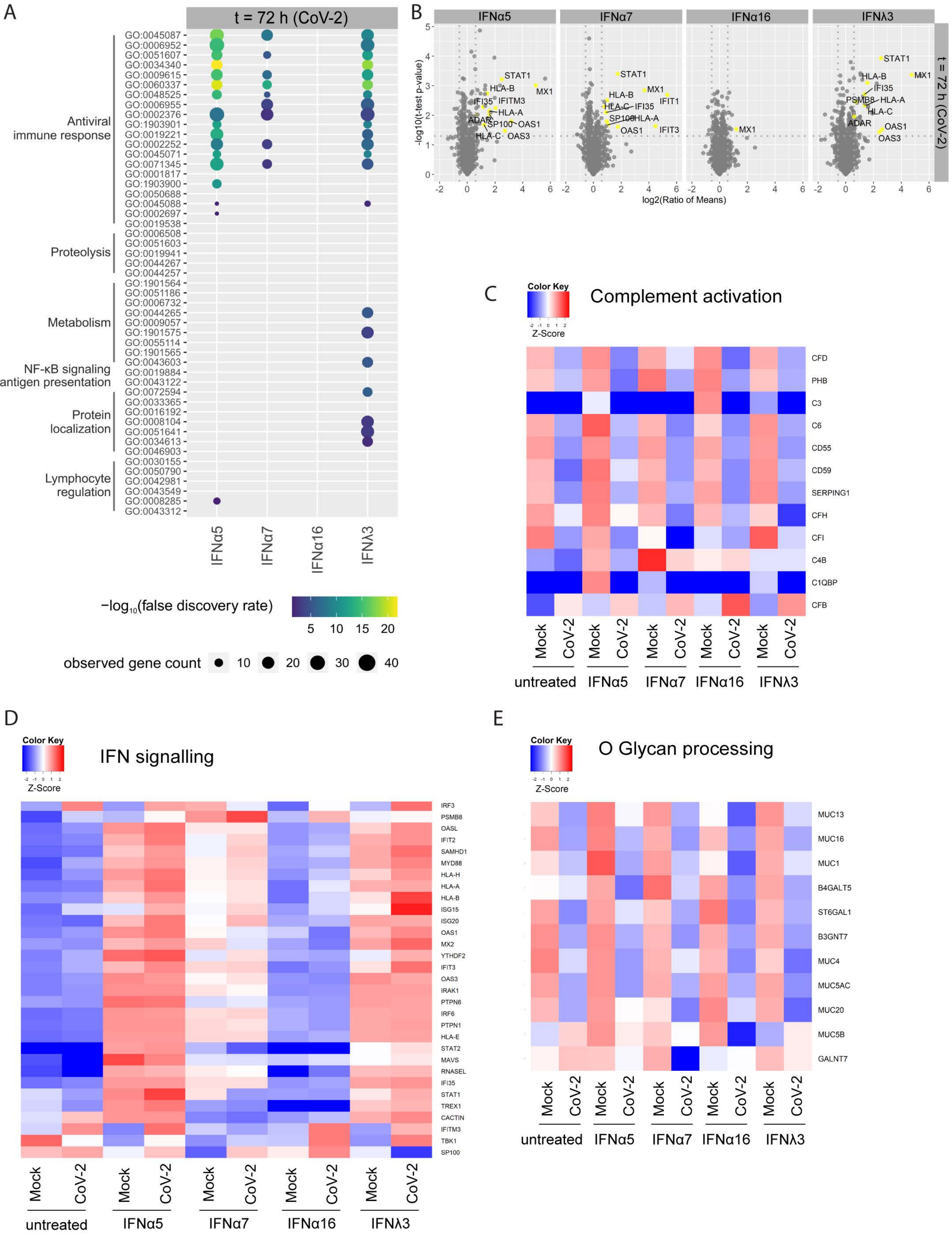
A
t = 72 h (CoV-2)
Antiviral immune response
Proteolysis
Metabolism
NF-κB signaling
antigen presentation
Protein localization
Lymphocyte regulation
GO:0045087
GO:0006952
GO:0051607
GO:0034340
GO:0009615
GO:0060337
GO:0048525
GO:0006955
GO:0002376
GO:1903901
GO:0019221
GO:0002252
GO:0045071
GO:0071345
GO:0001817
GO:1903900
GO:0050688
GO:0045088
GO:0002697
GO:0019538
GO:0006508
GO:0051603
GO:0019941
GO:0044267
GO:0044257
GO:1901564
GO:0051186
GO:0006732
GO:0044265
GO:0009057
GO:1901575
GO:0055114
GO:1901565
GO:0043603
GO:0019884
GO:0043122
GO:0072594
GO:0033365
GO:0016192
GO:0008104
GO:0051641
GO:0034613
GO:0046903
GO:0030155
GO:0050790
GO:0042981
GO:0043549
GO:0008285
GO:0043312
IFNα5
IFNα7
IFNα16
IFNλ3
−log10(false discovery rate)
5 10 15 20
observed gene count 10 20 30 40
B
IFNα5
IFNα7
IFNα16
IFNλ3
t = 72 h (CoV-2)
-log10(t-test p-value)
log2(Ratio of Means)
STAT1
MX1
HLA-B
IFI35
IFITM3
HLA-A
ADAR
SP100
OAS1
HLA-C
OAS3
IFIT1
IFIT3
PSMB8
C
Color Key
Z-Score
Complement activation
CFD
PHB
C3
C6
CD55
CD59
SERPING1
CFH
CFI
C4B
C1QBP
CFB
Mock
CoV-2
untreated
IFNα5
IFNα7
IFNα16
IFNλ3
D
Color Key
Z-Score
IFN signalling
IRF3
PSMB8
OASL
IFIT2
SAMHD1
MYD88
HLA-H
HLA-A
HLA-B
ISG15
ISG20
OAS1
MX2
YTHDF2
IFIT3
OAS3
IRAK1
PTPN6
IRF6
PTPN1
HLA-E
STAT2
MAVS
RNASEL
IFI35
STAT1
TREX1
CACTIN
IFITM3
TBK1
SP100
Mock
CoV-2
untreated
IFNα5
IFNα7
IFNα16
IFNλ3
E
Color Key
Z-Score
O Glycan processing
MUC13
MUC16
MUC1
B4GALT5
ST6GAL1
B3GNT7
MUC4
MUC5AC
MUC20
MUC5B
GALNT7
Mock
CoV-2
untreated
IFNα5
IFNα7
IFNα16
IFNλ3

Supplementary Fig. 7: IFN signature did not change upon SARS-CoV-2 infection

(A-E) Proteomic analysis of IFN-treated and/or SARS-CoV-2-infected hAECs. (A) Biological processes induced by IFNs in SARS-CoV-2-infected hAECs at 88 h p. treatment (t=72 h (CoV-2)). (B) Volcano plots of IFN-treated SARS-CoV-2-infected hAECs (t=72 h (CoV-2)) Detected ISGs are coloured yellow. Heatmaps displaying differentially expressed proteins which are associated with complement activation (C) IFN signalling (D) and O glycan processing (E). Comparisons of IFN-treated mock or SARS-CoV-2 infected hAECs at 72 h p.i. are depicted. n=4.

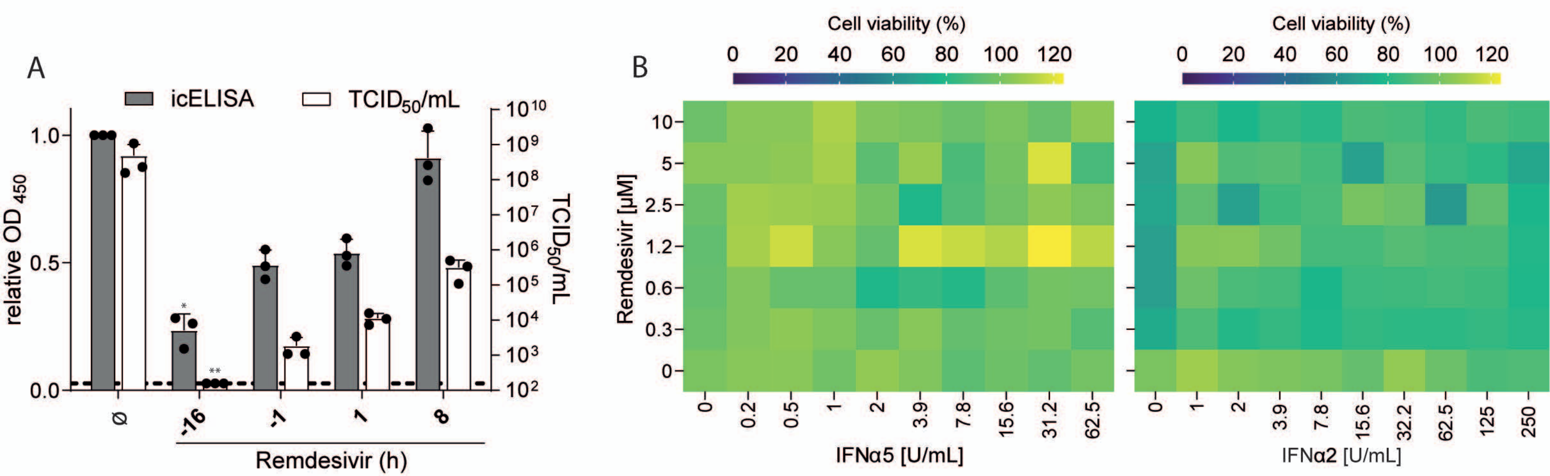
A
icELISA
TCID50/mL
relative OD 450
1.0
0.5
0.0
10^{10}
10^9
10^8
10^7
10^6
10^5
10^4
10^3
10^2
TCID50/mL
*
**
Ø
-16
-1
1
8
Remdesivir (h)
B
Cell viability (%)
0
20
40
60
80
100
120
Remdesivir [µM]
10
5
2.5
1.2
0.6
0.3
0
0
0.2
0.5
1
2
3.9
7.8
15.6
31.2
62.5
IFNα5 [U/mL]
Cell viability (%)
0
20
40
60
80
100
120
0
1
2
3.9
7.8
15.6
32.2
62.5
125
250
IFNα2 [U/mL]

Supplementary Fig. 8: Therapeutic potential of combination treatment

(A) Pre- and post-treatments of VeroE6 cells with remdesivir analysed by icELISA (black bars) and $TCID_{50}$ assay (white bars) shown as mean values + SEM. (B) Single and combined pre-treatments of IFNα5, IFNα2 and remdesivir in SARS-CoV-2 infected VeroE6 cells. Cell viability (%) normalised to untreated control (100%) is shown as heatmap. n=3. * $p<0.05$; ** $p<0.01$.

Supplementary Table 1 - SARS-CoV-2 Identifications

SARS-CoV2-Proteins and Peptides identified in T = 72 h +CoV2

Proteins

Donor 1					Donor 2					Donor 3					Donor 4										
untreated	IFNα5	IFNα7	IFNα16	IFNλ3	untreated	IFNα5	IFNα7	IFNα16	IFNλ3	untreated	IFNα5	IFNα7	IFNα16	IFNλ3	untreated	IFNα5	IFNα7	IFNα16	IFNλ3	Unique peptides	Sequence coverage [%]	Score	UniProt ID	Protein Names	Gene
0	0	0	0	0	0	0	0	0	0	0	0	0	0	0	9	0	0	0	0	8	6.7	45.809	P0DTC2	Spike glycoprotein	S
0	0	0	0	0	0	0	0	0	0	0	0	0	0	0	2	0	0	1	0	2	13.1	14.591	P0DTC5	Membrane protein	M
1	0	0	2	0	8	0	0	2	0	1	0	0	3	0	27	0	3	14	0	17	57	323.31	P0DTC9	Nucleoprotein	N

Peptides

Donor 1					Donor 2					Donor 3					Donor 4									
untreated	IFNα5	IFNα7	IFNα16	IFNλ3	untreated	IFNα5	IFNα7	IFNα16	IFNλ3	untreated	IFNα5	IFNα7	IFNα16	IFNλ3	untreated	IFNα5	IFNα7	IFNα16	IFNλ3	Protein Name	Missed cleavages	Score	UniProt ID	Sequence
0	0	0	0	0	0	0	0	0	0	0	0	0	0	0	1	0	0	0	0	Spike Protein S1	0	125.25	P0DTC2	FASVYAWNR
0	0	0	0	0	0	0	0	0	0	0	0	0	0	0	1	0	0	0	0	Spike Protein S1	0	135.87	P0DTC2	FLPFQQFGR
0	0	0	0	0	1	0	0	0	0	0	0	0	0	0	1	0	0	0	0	Spike Protein S1	0	96.034	P0DTC2	FQTLLALHR
0	0	0	0	0	0	0	0	0	0	0	0	0	0	0	2	0	0	1	0	Spike Protein S2	0	215.72	P0DTC2	LQSLQTYVTQQLIR
0	0	0	0	0	0	0	0	0	0	0	0	0	0	0	1	0	0	0	0	Spike Protein S2	0	93.551	P0DTC2	SFIEDLLFNK
0	0	0	0	0	0	0	0	0	0	0	0	0	0	0	1	0	0	0	0	Spike Protein S1	0	130.01	P0DTC2	TQLPPAYTNSFTR
0	0	0	0	0	0	0	0	0	0	0	0	0	0	0	1	0	0	0	0	Spike Protein S1	0	101.33	P0DTC2	VQPTESIVR
0	0	0	0	0	0	0	0	0	0	0	0	0	0	0	1	0	0	0	0	Spike Protein S1	0	207.34	P0DTC2	VYSTGSNVFQTR
0	0	0	0	0	0	0	0	0	0	0	0	0	0	0	1	0	0	0	0	Membrane protein	0	160.56	P0DTC5	LNTDHSSSSDNIALLVQ
0	0	0	0	0	0	0	0	0	0	0	0	0	0	0	1	0	0	1	0	Membrane protein	0	171.33	P0DTC5	VAGDSGFAAYSR
0	0	0	0	0	0	0	0	0	0	0	0	0	0	0	1	0	0	1	0	Nucleoprotein	0	168.08	P0DTC9	AYNVTQAFGR
0	0	0	0	0	1	0	0	0	0	0	0	0	0	0	1	0	0	0	0	Nucleoprotein	0	131.06	P0DTC9	DGIIWVATEGALNTPK
0	0	0	0	0	0	0	0	0	0	0	0	0	0	0	1	0	0	0	0	Nucleoprotein	1	63.893	P0DTC9	DHIGTRNPANNAAIVLQLPQGTTLPK
0	0	0	0	0	0	0	0	0	0	0	0	0	0	0	1	0	0	1	0	Nucleoprotein	0	357.53	P0DTC9	GPEQTQGNFGDQELIR
0	0	0	0	0	0	0	0	0	0	0	0	0	0	0	1	0	0	1	0	Nucleoprotein	0	266.97	P0DTC9	GQGVPINTNSSPDDQIGYYR
0	0	0	0	0	0	0	0	0	0	0	0	0	0	0	2	0	1	2	0	Nucleoprotein	1	334.6	P0DTC9	GQGVPINTNSSPDDQIGYYRR
0	0	0	0	0	0	0	0	0	0	0	0	0	0	0	1	0	0	0	0	Nucleoprotein	0	111.77	P0DTC9	IGMEVTPSGTWLTYTGAIK
1	0	0	1	0	1	0	0	1	0	1	0	0	1	0	2	0	1	2	0	Nucleoprotein	0	332.96	P0DTC9	ITFGGPSDSTGSNQNGER
0	0	0	0	0	0	0	0	0	0	0	0	0	0	0	1	0	0	0	0	Nucleoprotein	1	145.52	P0DTC9	ITFGGPSDSTGSNQNGERSGAR
0	0	0	0	0	1	0	0	0	0	0	0	0	0	0	1	0	0	0	0	Nucleoprotein	0	161.76	P0DTC9	MAGNGGDAALALLLLDR
0	0	0	0	0	0	0	0	0	0	0	0	0	0	0	2	0	0	1	0	Nucleoprotein	1	219.04	P0DTC9	MAGNGGDAALALLLLDRLNQLESK
0	0	0	0	0	2	0	0	0	0	0	0	0	0	0	2	0	0	2	0	Nucleoprotein	0	304.42	P0DTC9	NPANNAAIVLQLPQGTTLPK
0	0	0	0	0	0	0	0	0	0	0	0	0	0	0	2	0	0	1	0	Nucleoprotein	1	135.69	P0DTC9	QGTDYKHWPQIAQFAPSASAFFGMSR
0	0	0	0	0	1	0	0	0	0	0	0	0	0	0	2	0	0	0	0	Nucleoprotein	0	126.63	P0DTC9	QQTVTLLPAADLDDFSK
0	0	0	1	0	2	0	0	1	0	0	0	0	2	0	3	0	1	2	0	Nucleoprotein	1	363.83	P0DTC9	RGPEQTQGNFGDQELIR
0	0	0	0	0	0	0	0	0	0	0	0	0	0	0	1	0	0	0	0	Nucleoprotein	1	107.06	P0DTC9	RPQGLPNNTASWFTALTQHGK
0	0	0	0	0	0	0	0	0	0	0	0	0	0	0	1	0	0	0	0	Nucleoprotein	0	152.69	P0DTC9	WYFYYLGTGPEAGLPYGANK

Supplementary Table 2- Table of Contents - Significantly Differentially Abundant Proteins and On-Off Proteins

Filter criteria for significantly differentially abundant proteins: ≥ 2 unique peptides, p-value ≤ 0.05, ratio of means ≤ 0.67 or ≥ 1.5
Filter criteria for On-Off proteins: Quantified in minimum 3 of 4 samples of one experimental group but not the opposed group

Table No.	Content	
1	T = 0 h, IFNα5 / Untreated	**Significantly Differentially Abundant Proteins**
2	T = 0 h, IFNα7 / Untreated	
3	T = 0 h, IFNα16 / Untreated	
4	T = 0 h, IFNλ3 / Untreated	
5	T = 72 h (CoV-2), IFNα5 / Untreated	
6	T = 72 h (CoV-2), IFNα7 / Untreated	
7	T = 72 h (CoV-2), IFNα16 / Untreated	
8	T = 72 h (CoV-2), IFNλ3 / Untreated	
9	T = 72 h (mock) IFNα5 / Untreated	
10	T = 72 h (mock) IFNα7 / Untreated	
11	T = 72 h (mock) IFNα16 / Untreated	
12	T = 72 h (mock) IFNλ3 / Untreated	
13	T = 72 h, Untreated: CoV-2 / mock	
14	T = 72 h, IFNα5: CoV-2 / mock	
15	T = 72 h, IFNα7: CoV-2 / mock	
16	T = 72 h, IFNα16: CoV-2 / mock	
17	T = 72 h, IFNλ3: CoV-2 / mock	
18	T = 0 h, IFN / Untreated	**On-Off Proteins**
19	T = 72h, (mock) IFN / Untreated	
20	T = 72h, (CoV-2) IFN / Untreated	
21	T = 72 h, CoV-2 / mock	

T = 0 h, IFNα5 / Untreated

Uniprot ID	Protein Name	Gene Names	Number of Proteins	Peptides	Unique Peptides	p-Value	p_{FDR}-Value	Ratio of Means (IFNα5 / Untreated)
O14936	Peripheral plasma membrane protein CASK	CASK	1	5	5	0.020456521	0.84431694	0.542236659
O75822	Eukaryotic translation initiation factor 3 subunit J	EIF3J	1	4	4	0.024531672	0.84431694	1.91985968
P01011	Alpha-1-antichymotrypsin;Alpha-1-antichymotrypsin His-Pro-less	SERPINA3	1	6	6	0.027008694	0.890554595	2.785607202
P01889	HLA class I histocompatibility antigen, B-7 alpha chain	HLA-B	1	20	14	0.008017178	0.631261967	2.049845485
P04179	Superoxide dismutase [Mn], mitochondrial	SOD2	1	3	3	0.03648489	0.965633426	0.605294367
P05161	Ubiquitin-like protein ISG15	ISG15	1	8	8	0.000648287	0.428950212	93.57554337
P19835	Bile salt-activated lipase	CEL	1	11	11	0.004755993	0.631261967	1.525145936
P19971	Thymidine phosphorylase	TYMP	1	23	23	0.008805867	0.631261967	1.865603649
P20591	Interferon-induced GTP-binding protein Mx1;Interferon-induced GTP-binding protein Mx1, N-terminally processed	MX1	1	48	45	0.007809488	0.631261967	51.35881486
P22307	Non-specific lipid-transfer protein	SCP2	1	14	14	0.03789953	0.984775951	0.623100979
P23381	Tryptophan--tRNA ligase, cytoplasmic;T1-TrpRS;T2-TrpRS	WARS	1	28	28	0.012957478	0.730963468	2.988231399
P23497;Q9H930	Nuclear autoantigen Sp-100	SP100	2	10	10	0.020979763	0.84431694	1.702977673
P23786	Carnitine O-palmitoyltransferase 2, mitochondrial	CPT2	1	9	9	0.005284026	0.631261967	0.612087895
P29590	Protein PML	PML	1	33	33	0.023796771	0.84431694	1.531604171
P42224	Signal transducer and activator of transcription 1-alpha/beta	STAT1	1	35	35	0.002537616	0.503716758	3.07857135
P50914	60S ribosomal protein L14	RPL14	1	6	6	0.030617007	0.902257482	0.489846341
P80217	Interferon-induced 35 kDa protein	IFI35	1	10	10	0.013294489	0.730963468	1.967553753
Q01804	OTU domain-containing protein 4	OTUD4	1	2	2	0.036071231	0.965633426	0.660048181
Q53GL7	Poly [ADP-ribose] polymerase 10	PARP10	1	17	17	0.001297938	0.502711544	2.969708897
Q63HN8	E3 ubiquitin-protein ligase RNF213	RNF213	1	28	28	0.007089235	0.631261967	1.701118454
Q7Z2W4	Zinc finger CCCH-type antiviral protein 1	ZC3HAV1	1	10	9	0.015836805	0.748477576	1.506922458
Q969L2	Protein MAL2	MAL2	1	2	2	0.027504833	0.890554595	0.654500339
Q96AZ6	Interferon-stimulated gene 20 kDa protein	ISG20	1	6	6	0.011464435	0.730963468	10.40730141
Q96PP9;Q8N8V2	Guanylate-binding protein 4	GBP4	2	6	4	0.008904451	0.631261967	7.09646995
Q9BQE5	Apolipoprotein L2	APOL2	1	4	4	0.048692475	0.984775951	1.818077829
Q9H6T3	RNA polymerase II-associated protein 3	RPAP3	1	3	3	0.013732675	0.730963468	1.627748837
Q9NUQ6	SPATS2-like protein	SPATS2L	1	7	7	0.029536604	0.896758819	2.506981089
Q9NZT2	Opioid growth factor receptor	OGFR	1	10	10	0.011434157	0.730963468	1.514649802
Q9Y547	Intraflagellar transport protein 25 homolog	HSPB11	1	4	4	0.000261679	0.259716203	1.584021184

T = 0 h, IFNα7 / Untreated

Uniprot ID	Protein Name	Gene Names	Number of Proteins	Peptides	Unique Peptides	p-Value	p_{FDR}-Value	Ratio of Means (IFNα7 / Untreated)
O00410	Importin-5	IPO5	1	14	13	0.011047308	0.840858667	1.621904254
O75190;Q8NHS0;Q8WWF6;Q7Z6W7	DnaJ homolog subfamily B member 6	DNAJB6	4	7	6	0.005558028	0.803984849	0.618819474
O75882	Attractin	ATRN	1	8	8	0.032813423	0.920368351	1.501833728
P00387	NADH-cytochrome b5 reductase 3;NADH-cytochrome b5 reductase 3 membrane-bound form;NADH-cytochrome b5 reductase 3 soluble form	CYB5R3	1	9	9	0.020970918	0.845256394	0.583174242
P01889	HLA class I histocompatibility antigen, B-7 alpha chain	HLA-B	1	20	14	0.013591114	0.840858667	1.679458267
P05161	Ubiquitin-like protein ISG15	ISG15	1	8	8	0.030162483	0.920368351	15.66435228
P08237	ATP-dependent 6-phosphofructokinase, muscle type	PFKM	1	6	4	0.032934536	0.920368351	0.620291377
P13987	CD59 glycoprotein	CD59	1	4	4	0.011584884	0.840858667	2.212132671
P20591	Interferon-induced GTP-binding protein Mx1;Interferon-induced GTP-binding protein Mx1, N-terminally processed	MX1	1	48	45	0.010808667	0.840858667	27.92299594
P42224	Signal transducer and activator of transcription 1-alpha/beta	STAT1	1	35	35	0.009162757	0.840858667	2.371970655
P62995	Transformer-2 protein homolog beta	TRA2B	1	11	11	0.040746412	0.944882106	1.711326967
P80217	Interferon-induced 35 kDa protein	IFI35	1	10	10	0.011422429	0.840858667	1.618444791
Q53GL7	Poly [ADP-ribose] polymerase 10	PARP10	1	17	17	0.006319777	0.803984849	1.628792736
Q63HN8	E3 ubiquitin-protein ligase RNF213	RNF213	1	28	28	0.01112852	0.840858667	1.524826052
Q8N0W3	L-fucose kinase	FUK	1	5	5	0.017410091	0.840858667	0.591535642
Q92973	Transportin-1	TNPO1	1	12	8	0.026164845	0.896954874	0.543765238
Q93008;O00507	Probable ubiquitin carboxyl-terminal hydrolase FAF-X	USP9X	2	11	11	0.016082335	0.840858667	0.572809833
Q93077;Q7L7L0;P04908	Histone H2A type 1-C;Histone H2A type 3;Histone H2A type 1-B/E	HIST1H2AC;HIST3H2A;HIST1H2AB	3	8	2	0.048720946	0.944882106	0.440027011
Q96CV9	Optineurin	OPTN	1	5	5	0.002914805	0.648302369	1.889483476
Q9BT78	COP9 signalosome complex subunit 4	COPS4	1	9	9	0.006507057	0.803984849	0.616335508
Q9Y6I3	Epsin-1	EPN1	1	4	4	0.015876157	0.840858667	0.658594883

T = 0 h, IFNα16 / Untreated

Uniprot ID	Protein Name	Gene Names	Number of Proteins	Peptides	Unique Peptides	p-Value	p_{FDR}-Value	Ratio of Means (IFNα16 / Untreated)
Q71UI9;P0C0S5	Histone H2A.V;Histone H2A.Z	H2AFV;H2AFZ	2	5	3	0.013397682	0.763419391	2.885149603
P18510	Interleukin-1 receptor antagonist protein	IL1RN	1	5	5	0.046429593	0.960754525	0.630356021
P29992	Guanine nucleotide-binding protein subunit alpha-11	GNA11	1	10	4	0.017690874	0.885904519	1.742449141
P49593	Protein phosphatase 1F	PPM1F	1	5	5	0.004307646	0.579440325	0.649655333
P49959	Double-strand break repair protein MRE11A	MRE11A	1	7	7	0.029686516	0.944605204	0.606533458
Q15257	Serine/threonine-protein phosphatase 2A activator	PPP2R4	1	6	6	0.002087308	0.566644077	0.659362749
Q9Y6H1;Q5T1J5	Coiled-coil-helix-coiled-coil-helix domain-containing protein 2;Putative coiled-coil-helix-coiled-coil-helix domain-containing protein CHCHD2P9, mitochondrial	CHCHD2;CHCHD2P9	2	4	4	0.047589903	0.960754525	1.523655058
Q92743	Serine protease HTRA1	HTRA1	1	5	5	0.032781891	0.944605204	0.631404114
Q92934	Bcl2-associated agonist of cell death	BAD	1	7	7	0.010330244	0.706801765	1.579682649
Q92973	Transportin-1	TNPO1	1	12	8	0.030543812	0.944605204	0.544353394
Q9P2B2	Prostaglandin F2 receptor negative regulator	PTGFRN	1	6	6	0.004747079	0.579440325	0.6561842

T = 0 h, IFNλ3 / Untreated

Uniprot ID	Protein Name	Gene Names	Number of Proteins	Peptides	Unique Peptides	p-Value	p_{FDR}-Value	Ratio of Means (IFNλ3 / Untreated)
O60218	Aldo-keto reductase family 1 member B10	AKR1B10	1	9	7	0.008513172	0.788171565	0.592971555
P00387	NADH-cytochrome b5 reductase 3;NADH-cytochrome b5 reductase 3 membrane-bound form;NADH-cytochrome b5 reductase 3 soluble form	CYB5R3	1	9	9	0.047753359	0.920209345	0.562559919
P01889	HLA class I histocompatibility antigen, B-7 alpha chain	HLA-B	1	20	14	0.015174014	0.788171565	1.673608522
P05161	Ubiquitin-like protein ISG15	ISG15	1	8	8	0.007703968	0.788171565	25.59960899
P11177	Pyruvate dehydrogenase E1 component subunit beta, mitochondrial	PDHB	1	9	9	0.04917172	0.920209345	0.657947342
P13073	Cytochrome c oxidase subunit 4 isoform 1, mitochondrial	COX4I1	1	7	7	9.68211E-05	0.144592217	0.640600359
P14550	Alcohol dehydrogenase [NADP(+)]	AKR1A1	1	22	22	0.036341199	0.916364839	0.666338713
P20591	Interferon-induced GTP-binding protein Mx1;Interferon-induced GTP-binding protein Mx1, N-terminally processed	MX1	1	48	45	0.011446164	0.788171565	33.66804943
P40926	Malate dehydrogenase, mitochondrial	MDH2	1	15	15	0.001390873	0.506810306	0.646274878
P42224	Signal transducer and activator of transcription 1-alpha/beta	STAT1	1	35	35	0.010146187	0.788171565	2.623099718
P43155	Carnitine O-acetyltransferase	CRAT	1	3	3	0.045871609	0.920209345	0.435731703
P68431;P84243;Q71DI3;Q16695;Q6NXT2	Histone H3.1;Histone H3.3;Histone H3.2;Histone H3.1t;Histone H3.3C	HIST1H3A;H3F3A;HIST2H3A;HIST3H3;H3F3C	5	8	8	0.039068219	0.91824849	0.631374888
P78406	mRNA export factor	RAE1	1	6	6	0.002573102	0.577895937	0.598296535
P80217	Interferon-induced 35 kDa protein	IFI35	1	10	10	0.010962395	0.788171565	2.072933639
Q06830	Peroxiredoxin-1	PRDX1	1	24	21	0.010260691	0.788171565	0.666315637
Q53GL7	Poly [ADP-ribose] polymerase 10	PARP10	1	17	17	0.012791252	0.788171565	2.834795096
Q8IW45	ATP-dependent (S)-NAD(P)H-hydrate dehydratase	CARKD	1	5	5	0.017850244	0.788171565	1.537650653
Q8WUY1	Protein THEM6	THEM6	1	5	5	0.046230562	0.920209345	0.616201183
Q92925;Q96GM5;Q6STE5	SWI/SNF-related matrix-associated actin-dependent regulator of chromatin subfamily D member 2	SMARCD2	3	7	7	0.043896516	0.920209345	1.737795345
Q96AZ6	Interferon-stimulated gene 20 kDa protein	ISG20	1	6	6	0.003269987	0.577895937	3.291570345
Q96RQ3	Methylcrotonoyl-CoA carboxylase subunit alpha, mitochondrial	MCCC1	1	10	10	0.041920719	0.920209345	0.632200764
Q9NRC6	Spectrin beta chain, non-erythrocytic 5	SPTBN5	1	3	3	0.013066786	0.788171565	1.618321585

T = 72 h (CoV-2), IFNα5 / Untreated

Uniprot ID	Protein Name	Gene Names	Number of Proteins	Peptides	Unique Peptides	p-Value	p_{FDR}-Value	Ratio of Means (IFNα5 / Untreated)
O00182	Galectin-9	LGALS9	1	11	8	0.024469544	0.449265335	1.504917534
O00468	Agrin;Agrin N-terminal 110 kDa subunit;Agrin C-terminal 110 kDa subunit;Agrin C-terminal 90 kDa fragment;Agrin C-terminal 22 kDa	AGRN	1	34	34	0.00574231	0.263841253	1.551996998
O14879	Interferon-induced protein with tetratricopeptide repeats 3	IFIT3	1	25	25	0.017122469	0.383543299	80.63984248
P00973	2-5-oligoadenylate synthase 1	OAS1	1	9	9	0.016080942	0.379840471	9.028224832
P01889	HLA class I histocompatibility antigen, B-7 alpha chain	HLA-B	1	20	14	0.001823769	0.178265445	2.69898212
P04439	HLA class I histocompatibility antigen, A-3 alpha chain	HLA-A	1	20	13	0.007167454	0.293314288	3.018975491
P09914;Q5T764	Interferon-induced protein with tetratricopeptide repeats 1	IFIT1	2	25	24	0.00063248	0.134591684	128.771715
Q6FI13;P0C0S8;Q99878;Q9BTM1;Q16777;Q96KK5;P20671;P16104;Q96QV6	Histone H2A type 2-A;Histone H2A type 1;Histone H2A type 1-J;Histone H2A.J;Histone H2A type 2-C;Histone H2A type 1-H;Histone H2A type 1-D;Histone H2AX;Histone H2A type 1-A	HIST2H2AA3;HIST1H2AG;HIST1H2AJ;H2AFJ;HIST2H2AC;HIST1H2AH;HIST1H2AD;H2AFX;HIST1H2A A	9	8	2	0.018563216	0.405161292	0.620387474
P10321	HLA class I histocompatibility antigen, Cw-7 alpha chain	HLA-C	1	16	10	0.020986854	0.413518749	2.306302985
P13073	Cytochrome c oxidase subunit 4 isoform 1, mitochondrial	COX4I1	1	7	7	0.034152601	0.457086384	0.655134513
P19525	Interferon-induced, double-stranded RNA-activated protein kinase	EIF2AK2	1	13	13	0.012513862	0.337082246	2.727989372
P19971	Thymidine phosphorylase	TYMP	1	23	23	0.000989308	0.161942127	5.140175092
P20591	Interferon-induced GTP-binding protein Mx1;Interferon-induced GTP-binding protein Mx1, N-terminally processed	MX1	1	48	45	0.000969346	0.161942127	30.71400915
P21980;O43548	Protein-glutamine gamma-glutamyltransferase 2	TGM2	2	31	31	0.005046473	0.261924278	1.699879612
P23381	Tryptophan--tRNA ligase, cytoplasmic;T1-TrpRS;T2-TrpRS	WARS	1	28	28	0.000112939	0.060083622	3.87236415
P23497;Q9H930	Nuclear autoantigen Sp-100	SP100	2	10	10	0.009280017	0.32107976	3.016108827
P28065	Proteasome subunit beta type-9	PSMB9	1	10	10	0.001835203	0.178265445	1.92455023
P28072	Proteasome subunit beta type-6	PSMB6	1	7	7	0.001675991	0.178265445	0.623422384
P28074	Proteasome subunit beta type-5	PSMB5	1	9	9	0.00601856	0.263841253	0.60640131
P28838	Cytosol aminopeptidase	LAP3	1	32	32	0.001316233	0.178265445	2.419504104
P29466;Q5EG05	Caspase-1;Caspase-1 subunit p20;Caspase-1 subunit p10	CASP1	2	11	11	1.3557E-05	0.028849216	1.889806322
P29590	Protein PML	PML	1	33	33	0.001918108	0.178265445	2.419340576
P42224	Signal transducer and activator of transcription 1-alpha/beta	STAT1	1	35	35	0.000603523	0.134591684	5.609034351
P55265	Double-stranded RNA-specific adenosine deaminase	ADAR	1	19	19	0.009421842	0.32107976	1.603790329
P57076	UPF0769 protein C21orf59	C21orf59	1	5	5	0.048535775	0.487189286	0.645175066
P80217	Interferon-induced 35 kDa protein	IFI35	1	10	10	0.005256076	0.263841253	2.205278328
Q01628;C9JQL5	Interferon-induced transmembrane protein 3	IFITM3	2	4	2	0.005690281	0.263841253	4.124111782
Q08380	Galectin-3-binding protein	LGALS3BP	1	25	25	0.001414987	0.178265445	1.670650391
Q08AF3;Q7Z7L1;Q68D06	Schlafen family member 5	SLFN5	3	5	5	0.048374435	0.487189286	1.666078024
Q13501	Sequestosome-1	SQSTM1	1	10	10	0.021431445	0.414601048	1.550081846
Q16270	Insulin-like growth factor-binding protein 7	IGFBP7	1	6	6	0.046653372	0.478583607	1.506124483
Q53GL7	Poly [ADP-ribose] polymerase 10	PARP10	1	17	17	0.002561383	0.208679287	3.942489754
Q56VL3	OCIA domain-containing protein 2	OCIAD2	1	3	3	0.027483579	0.451259195	0.64755643
Q5EBM0	UMP-CMP kinase 2, mitochondrial	CMPK2	1	18	18	0.000578982	0.134591684	48.95897605
Q5R3I4	Tetratricopeptide repeat protein 38	TTC38	1	4	4	0.047315123	0.481734452	1.861750602
Q63HN8	E3 ubiquitin-protein ligase RNF213	RNF213	1	28	28	0.004791703	0.254918607	2.461481075
Q6P2E9	Enhancer of mRNA-decapping protein 4	EDC4	1	11	11	0.028321246	0.451259195	0.639258804
Q709C8	Vacuolar protein sorting-associated protein 13C	VPS13C	1	10	10	0.006075292	0.263841253	0.527160356
Q86TU7	Histone-lysine N-methyltransferase setd3	SETD3	1	3	3	0.008501741	0.320488686	0.662578579
Q86UW9	Probable E3 ubiquitin-protein ligase DTX2	DTX2	1	3	3	0.029729897	0.451259195	0.638491054

Q8IYM9	E3 ubiquitin-protein ligase TRIM22	TRIM22	1	5	5	0.027460273	0.451259195	1.58883453
Q92522	Histone H1x	H1FX	1	5	5	0.019324782	0.405161292	1.855994381
Q96PP9;Q8N8V2	Guanylate-binding protein 4	GBP4	2	6	4	0.04256742	0.47373118	3.09122546
Q99426	Tubulin-folding cofactor B	TBCB	1	6	6	0.011792292	0.329922649	0.578760977
Q99988	Growth/differentiation factor 15	GDF15	1	12	12	0.016797121	0.383543299	1.713689828
Q9BQE5	Apolipoprotein L2	APOL2	1	4	4	0.000981016	0.161942127	2.446201569
Q9NRD9	Dual oxidase 1	DUOX1	1	17	12	0.009505651	0.32107976	1.685148555
Q9NUL5	UPF0515 protein C19orf66	C19orf66	1	4	4	0.007977496	0.30865658	3.23880021
Q9NZT2	Opioid growth factor receptor	OGFR	1	10	10	0.002902712	0.212998982	1.648340236
Q9P2B2	Prostaglandin F2 receptor negative regulator	PTGFRN	1	6	6	0.013608096	0.353146684	0.65875054
Q9P2R3	Rabankyrin-5	ANKFY1	1	7	7	0.029797961	0.451259195	1.544197517
Q9Y6K5	2-5-oligoadenylate synthase 3	OAS3	1	15	15	0.033263426	0.453747253	6.538458042

T = 72 h (CoV-2), IFNα7 / Untreated

Uniprot ID	Protein Name	Gene Names	Number of Proteins	Peptides	Unique Peptides	p-Value	p_{FDR}-Value	Ratio of Means (IFNα7 / Untreated)
O00442	RNA 3-terminal phosphate cyclase	RTCA	1	8	8	0.022443609	0.542982077	0.665727058
O14879	Interferon-induced protein with tetratricopeptide repeats 3	IFIT3	1	25	25	0.023058269	0.542982077	22.21628095
O75976	Carboxypeptidase D	CPD	1	12	12	0.044558691	0.557156448	0.440709333
P00973	2-5-oligoadenylate synthase 1	OAS1	1	9	9	0.024420354	0.542982077	3.392710018
P01889	HLA class I histocompatibility antigen, B-7 alpha chain	HLA-B	1	20	14	0.003118051	0.456004853	1.959806303
P04439	HLA class I histocompatibility antigen, A-3 alpha chain	HLA-A	1	20	13	0.020852211	0.542982077	2.069803906
P06132	Uroporphyrinogen decarboxylase	UROD	1	12	12	0.001987664	0.3566244	0.590422048
P08195	4F2 cell-surface antigen heavy chain	SLC3A2	1	5	5	0.012810778	0.518590162	0.635344774
P08571	Monocyte differentiation antigen CD14;Monocyte differentiation antigen CD14, urinary form;Monocyte differentiation antigen CD14, membrane-bound form	CD14	1	7	7	0.025926764	0.542982077	0.541013723
P09543	2,3-cyclic-nucleotide 3-phosphodiesterase	CNP	1	9	9	0.041140183	0.557156448	1.576225431
P09914;Q5T764	Interferon-induced protein with tetratricopeptide repeats 1	IFIT1	2	25	24	0.002033013	0.3566244	40.32729178
Q71UI9;P0C0S5	Histone H2A.V;Histone H2A.Z	H2AFV;H2AFZ	2	5	3	0.047388155	0.564221141	0.544830472
P10321	HLA class I histocompatibility antigen, Cw-7 alpha chain	HLA-C	1	16	10	0.005044577	0.482537071	1.71330411
P19525	Interferon-induced, double-stranded RNA-activated protein kinase	EIF2AK2	1	13	13	0.049535457	0.57608915	1.92550923
P19971	Thymidine phosphorylase	TYMP	1	23	23	0.004306092	0.477069675	2.523566614
P20591	Interferon-induced GTP-binding protein Mx1;Interferon-induced GTP-binding protein Mx1, N-terminally processed	MX1	1	48	45	0.001425725	0.3566244	12.82408058
P23497;Q9H930	Nuclear autoantigen Sp-100	SP100	2	10	10	0.015904545	0.523110439	1.944950358
P28838	Cytosol aminopeptidase	LAP3	1	32	32	0.0035137	0.456004853	1.708728519
P31949	Protein S100-A11;Protein S100-A11, N-terminally processed	S100A11	1	2	2	0.033171365	0.557156448	1.797374223
P42224	Signal transducer and activator of transcription 1-alpha/beta	STAT1	1	35	35	0.000397629	0.279003085	3.355776488
P42330;P17516	Aldo-keto reductase family 1 member C3	AKR1C3	2	18	10	0.010646468	0.511729016	0.652257494
P46776	60S ribosomal protein L27a	RPL27A	1	6	6	0.019971953	0.542982077	0.575253297
P51970	NADH dehydrogenase [ubiquinone] 1 alpha subcomplex subunit 8	NDUFA8	1	6	6	0.029079218	0.542982077	1.702708602
P80217	Interferon-induced 35 kDa protein	IFI35	1	10	10	0.007965926	0.48293567	1.885947152
Q13564	NEDD8-activating enzyme E1 regulatory subunit	NAE1	1	8	8	0.035192796	0.557156448	0.629676649
Q13751	Laminin subunit beta-3	LAMB3	1	20	20	0.023075529	0.542982077	1.88431572
Q14244	Ensconsin	MAP7	1	8	8	0.044839125	0.557156448	0.446941301
Q5EBM0	UMP-CMP kinase 2, mitochondrial	CMPK2	1	18	18	0.011557129	0.518590162	7.22274518
Q6ZU80	Centrosomal protein of 128 kDa	CEP128	1	7	7	0.025008651	0.542982077	1.551110464
Q709C8	Vacuolar protein sorting-associated protein 13C	VPS13C	1	10	10	0.006805587	0.482537071	0.572940289
Q93052	Lipoma-preferred partner	LPP	1	12	12	0.025572279	0.542982077	1.5100862
Q96FV0	Leucine-rich repeat-containing protein 46	LRRC46	1	10	10	0.034142624	0.557156448	1.86195317
Q96PP9;Q8N8V2	Guanylate-binding protein 4	GBP4	2	6	4	0.03621397	0.557156448	1.54952868
Q9H2D6	TRIO and F-actin-binding protein	TRIOBP	1	14	14	0.04448977	0.557156448	1.638369602
Q9NUQ6	SPATS2-like protein	SPATS2L	1	7	7	0.034899838	0.557156448	2.07300735

T = 72 h (CoV-2), IFNα16 / Untreated

Uniprot ID	Protein Name	Gene Names	Number of Proteins	Peptides	Unique Peptides	p-Value	p_{FDR}-Value	Ratio of Means (IFNα16 / Untreated)
P14927	Cytochrome b-c1 complex subunit 7	UQCRB	1	3	3	0.02934394	0.811415117	1.793643054
P20591	Interferon-induced GTP-binding protein Mx1;Interferon-induced GTP-binding protein Mx1, N-terminally processed	MX1	1	48	45	0.029345217	0.811415117	2.311083046
P20674	Cytochrome c oxidase subunit 5A, mitochondrial	COX5A	1	5	5	0.009735971	0.811415117	1.851845411
P42025	Beta-centractin	ACTR1B	1	13	8	0.0081513	0.811415117	1.615411667
P50452	Serpin B8	SERPINB8	1	8	5	0.00534587	0.760895449	0.557923858
P60660;P14649	Myosin light polypeptide 6	MYL6	2	10	10	0.008009926	0.811415117	1.546819146
Q03154	Aminoacylase-1	ACY1	1	13	13	0.028752305	0.811415117	1.510005125
Q15185	Prostaglandin E synthase 3	PTGES3	1	5	5	0.044217652	0.859855344	0.565302942
Q8NFU3	Thiosulfate sulfurtransferase/rhodanese-like domain-containing protein	TSTD1	1	4	4	0.001700912	0.705097724	1.614396852
Q99538	Legumain	LGMN	1	6	6	0.032148762	0.814371479	0.592627023
Q9NUL5	UPF0515 protein C19orf66	C19orf66	1	4	4	0.026042375	0.811415117	1.640652499

T = 72 h (CoV-2), IFNλ3 / Untreated

Uniprot ID	Protein Name	Gene Names	Number of Proteins	Peptides	Unique Peptides	p-Value	p_{FDR}-Value	Ratio of Means (IFNλ3 / Untreated)
O00410	Importin-5	IPO5	1	14	13	0.001333139	0.177724081	0.631156478
O14545	TRAF-type zinc finger domain-containing protein 1	TRAFD1	1	7	7	0.003753399	0.268399721	1.766544974
O14879	Interferon-induced protein with tetratricopeptide repeats 3	IFIT3	1	25	25	0.004765196	0.286166561	66.33182125
O14949	Cytochrome b-c1 complex subunit 8	UQCRQ	1	4	4	0.031993212	0.416128485	0.639477656
O15533	Tapasin	TAPBP	1	6	6	0.002301183	0.233734409	2.350964746
O75964	ATP synthase subunit g, mitochondrial	ATP5L	1	3	3	0.030151265	0.408504901	0.527350572
O75976	Carboxypeptidase D	CPD	1	12	12	0.016671507	0.370420035	0.664097651
P00973	2-5-oligoadenylate synthase 1	OAS1	1	9	9	0.029726215	0.408504901	6.102939967
P01889	HLA class I histocompatibility antigen, B-7 alpha chain	HLA-B	1	20	14	0.000788971	0.126797117	2.945686097
P04439	HLA class I histocompatibility antigen, A-3 alpha chain	HLA-A	1	20	13	0.004573026	0.286166561	2.74414151
P09543	2,3-cyclic-nucleotide 3-phosphodiesterase	CNP	1	9	9	0.011168667	0.335531924	2.032743666
P09914;Q5T764	Interferon-induced protein with tetratricopeptide repeats 1	IFIT1	2	25	24	0.002430838	0.235680771	121.7540751
P10321	HLA class I histocompatibility antigen, Cw-7 alpha chain	HLA-C	1	16	10	0.004609711	0.286166561	2.562732535
P10412;Q02539;P22492	Histone H1.4	HIST1H1E	3	8	2	0.00693294	0.326888592	0.573191171
P15170	Eukaryotic peptide chain release factor GTP-binding subunit ERF3A	GSPT1	1	8	4	0.0403098	0.446902486	0.646700795
P19525	Interferon-induced, double-stranded RNA-activated protein kinase	EIF2AK2	1	13	13	0.004829815	0.286166561	3.05057632
P19971	Thymidine phosphorylase	TYMP	1	23	23	0.000832236	0.126797117	4.677156685
P20591	Interferon-induced GTP-binding protein Mx1;Interferon-induced GTP-binding protein Mx1, N-terminally processed	MX1	1	48	45	0.000430761	0.121167248	26.99318237
P21980;O43548	Protein-glutamine gamma-glutamyltransferase 2	TGM2	2	31	31	0.00321105	0.268399721	1.779006147
P23381	Tryptophan--tRNA ligase, cytoplasmic;T1-TrpRS;T2-TrpRS	WARS	1	28	28	0.005305135	0.298984601	3.033754683
P28062	Proteasome subunit beta type-8	PSMB8	1	14	14	0.003466958	0.268399721	1.640758712
P28065	Proteasome subunit beta type-9	PSMB9	1	10	10	0.000380534	0.121167248	2.072520042
P28072	Proteasome subunit beta type-6	PSMB6	1	7	7	0.029642595	0.408504901	0.54716141
P28838	Cytosol aminopeptidase	LAP3	1	32	32	0.00043518	0.121167248	2.117949432
P29590	Protein PML	PML	1	33	33	0.000635912	0.126797117	2.27617433
P29966	Myristoylated alanine-rich C-kinase substrate	MARCKS	1	3	3	0.026821221	0.403019414	1.513989241
P41208	Centrin-2	CETN2	1	7	7	0.011685043	0.3461694	2.003334622
P41226	Ubiquitin-like modifier-activating enzyme 7	UBA7	1	16	16	0.027908405	0.403019414	1.555849204
P42025	Beta-centractin	ACTR1B	1	13	8	0.047505753	0.457777502	1.816497142
P42224	Signal transducer and activator of transcription 1-alpha/beta	STAT1	1	35	35	0.000116807	0.121167248	5.838913935
P46776	60S ribosomal protein L27a	RPL27A	1	6	6	0.045515184	0.453307332	0.481333429
P50336	Protoporphyrinogen oxidase	PPOX	1	17	17	0.007259629	0.326888592	0.627614984
P50914	60S ribosomal protein L14	RPL14	1	6	6	0.042003849	0.446902486	0.618232463
P52789	Hexokinase-2	HK2	1	10	7	0.012858117	0.360873194	1.702751102
P53365	Arfaptin-2	ARFIP2	1	6	6	0.033113254	0.418374313	1.504325026
P55265	Double-stranded RNA-specific adenosine deaminase	ADAR	1	19	19	0.011111341	0.335531924	1.521303399
P61353	60S ribosomal protein L27	RPL27	1	5	5	0.02429826	0.403019414	0.646004933
P62081	40S ribosomal protein S7	RPS7	1	6	6	0.023489496	0.403019414	0.563244798
P62266	40S ribosomal protein S23	RPS23	1	3	3	0.048410272	0.458929375	0.624228009
P62269	40S ribosomal protein S18	RPS18	1	16	16	0.012207906	0.348665401	0.501292753
P62304	Small nuclear ribonucleoprotein E	SNRPE	1	4	4	0.040292414	0.446902486	0.569222481
P62805	Histone H4	HIST1H4A	1	13	13	0.031123453	0.414914535	0.528904214
P62899	60S ribosomal protein L31	RPL31	1	6	6	0.033872374	0.422513295	0.607839207

P62910	60S ribosomal protein L32	RPL32	1	6	6	0.00947205	0.335531924	0.352056823
P80217	Interferon-induced 35 kDa protein	IFI35	1	10	10	0.001908651	0.221922487	2.562452473
Q08380	Galectin-3-binding protein	LGALS3BP	1	25	25	0.00055999	0.126797117	1.508351675
Q12907	Vesicular integral-membrane protein VIP36	LMAN2	1	8	8	0.029714801	0.408504901	0.656213156
Q53GL7	Poly [ADP-ribose] polymerase 10	PARP10	1	17	17	0.007855263	0.326888592	3.64578244
Q5EBM0	UMP-CMP kinase 2, mitochondrial	CMPK2	1	18	18	0.000449581	0.121167248	42.14286883
Q63HN8	E3 ubiquitin-protein ligase RNF213	RNF213	1	28	28	0.01465361	0.367139981	2.066678035
Q709C8	Vacuolar protein sorting-associated protein 13C	VPS13C	1	10	10	0.019715843	0.385486612	0.500852146
Q8IY67	Ribonucleoprotein PTB-binding 1	RAVER1	1	6	6	0.01481034	0.367139981	1.571025697
Q92820	Gamma-glutamyl hydrolase	GGH	1	8	8	0.02693994	0.403019414	0.600970424
Q9BQE5	Apolipoprotein L2	APOL2	1	4	4	0.004026625	0.268399721	2.610022316
Q9BUP3	Oxidoreductase HTATIP2	HTATIP2	1	6	6	0.022313009	0.399946617	1.573780658
Q9NTZ6	RNA-binding protein 12	RBM12	1	9	9	0.041197099	0.446902486	1.541168447
Q9NUL5	UPF0515 protein C19orf66	C19orf66	1	4	4	0.008701656	0.33494246	3.78596436
Q9NUQ6	SPATS2-like protein	SPATS2L	1	7	7	0.019834379	0.385486612	2.145604594
Q9Y277	Voltage-dependent anion-selective channel protein 3	VDAC3	1	12	12	0.02644514	0.403019414	0.667387171
Q9Y3U8	60S ribosomal protein L36	RPL36	1	7	7	0.041224877	0.446902486	0.604278306
Q9Y4F1	FERM, RhoGEF and pleckstrin domain-containing protein 1	FARP1	1	6	6	0.044104458	0.450968623	2.029789541
Q9Y6K5	2-5-oligoadenylate synthase 3	OAS3	1	15	15	0.036763625	0.438082752	5.392341932

T = 72 h (mock) IFNα5 / Untreated

Uniprot ID	Protein Name	Gene Names	Number of Proteins	Peptides	Unique Peptides	p-Value	p_{FDR}-Value	Ratio of Means (IFNα5 / Untreated)
O00468	Agrin;Agrin N-terminal 110 kDa subunit;Agrin C-terminal 110 kDa subunit;Agrin C-terminal 90 kDa fragment;Agrin C-terminal 22 kDa fragment	AGRN	1	34	34	0.0029584	0.24160194	1.514028072
O14545	TRAF-type zinc finger domain-containing protein 1	TRAFD1	1	7	7	0.011016903	0.373099156	1.649960951
O14879	Interferon-induced protein with tetratricopeptide repeats 3	IFIT3	1	25	25	0.001137239	0.14832759	94.98826837
O14929	Histone acetyltransferase type B catalytic subunit	HAT1	1	4	4	0.048857382	0.469805802	0.529713792
O14933	Ubiquitin/ISG15-conjugating enzyme E2 L6	UBE2L6	1	6	6	0.028295828	0.402624958	4.24173436
O15533	Tapasin	TAPBP	1	6	6	0.019584805	0.399231048	3.254425911
O95361;Q309B1	Tripartite motif-containing protein 16;Tripartite motif-containing protein 16-	TRIM16;TRIM16L	2	5	5	0.013402838	0.373099156	0.642035941
P00751	Complement factor B;Complement factor B Ba fragment;Complement factor	CFB	1	40	40	0.033051011	0.417504022	1.610129574
P01009	Alpha-1-antitrypsin;Short peptide from AAT	SERPINA1	1	3	3	0.020106274	0.399231048	0.591002118
P01889	HLA class I histocompatibility antigen, B-7 alpha chain	HLA-B	1	20	14	0.003380794	0.25621587	3.712940575
P01903;P01906	HLA class II histocompatibility antigen, DR alpha chain	HLA-DRA	2	8	8	0.005384354	0.294228008	1.962549487
P04439	HLA class I histocompatibility antigen, A-3 alpha chain	HLA-A	1	20	13	0.032136828	0.415819206	2.578384906
P05161	Ubiquitin-like protein ISG15	ISG15	1	8	8	0.000168719	0.067512497	150.1045229
P09543	2,3-cyclic-nucleotide 3-phosphodiesterase	CNP	1	9	9	0.013538471	0.373099156	2.099137886
P13928;Q5VT79	Annexin A8;Annexin A8-like protein 2	ANXA8;ANXA8L2	2	14	14	0.028853248	0.402624958	0.628449187
P15374	Ubiquitin carboxyl-terminal hydrolase isozyme L3	UCHL3	1	7	7	0.035994594	0.424613984	2.420964435
P18510	Interleukin-1 receptor antagonist protein	IL1RN	1	5	5	0.023076716	0.402624958	1.945917834
P19525	Interferon-induced, double-stranded RNA-activated protein kinase	EIF2AK2	1	13	13	0.014065463	0.374319408	3.130219025
P19971	Thymidine phosphorylase	TYMP	1	23	23	0.001188298	0.14832759	4.798128007
P20290	Transcription factor BTF3	BTF3	1	5	5	0.026427685	0.402624958	0.601866257
P20591	Interferon-induced GTP-binding protein Mx1;Interferon-induced GTP-binding protein Mx1, N-terminally processed	MX1	1	48	45	0.000107477	0.067512497	34.79396037
P21926	CD9 antigen	CD9	1	4	4	0.010859529	0.373099156	0.522442165
P21980;O43548	Protein-glutamine gamma-glutamyltransferase 2	TGM2	2	31	31	0.008012986	0.361777794	1.762293622
P23381	Tryptophan--tRNA ligase, cytoplasmic;T1-TrpRS;T2-TrpRS	WARS	1	28	28	0.004165832	0.267875641	3.590316076
P23497;Q9H930	Nuclear autoantigen Sp-100	SP100	2	10	10	0.01191578	0.373099156	3.41043107
P28065	Proteasome subunit beta type-9	PSMB9	1	10	10	0.003734742	0.264170755	1.790827266
P28838	Cytosol aminopeptidase	LAP3	1	32	32	0.000300331	0.067512497	2.370355916
P29466;Q5EG05	Caspase-1;Caspase-1 subunit p20;Caspase-1 subunit p10	CASP1	2	11	11	0.000136916	0.067512497	1.518221704
P29590	Protein PML	PML	1	33	33	0.000350658	0.067512497	2.304786949
P29728	2-5-oligoadenylate synthase 2	OAS2	1	11	11	0.000510349	0.077354383	11.73869233
P41226	Ubiquitin-like modifier-activating enzyme 7	UBA7	1	16	16	0.035029771	0.424613984	1.766693504
P42224	Signal transducer and activator of transcription 1-alpha/beta	STAT1	1	35	35	0.000320769	0.067512497	6.773181717
P55210	Caspase-7;Caspase-7 subunit p20;Caspase-7 subunit p11	CASP7	1	11	11	0.007694071	0.35749574	1.536928085
P55265	Double-stranded RNA-specific adenosine deaminase	ADAR	1	19	19	0.01233365	0.373099156	1.618786851
P61221	ATP-binding cassette sub-family E member 1	ABCE1	1	3	3	0.00230223	0.226388443	0.669070197
P61769	Beta-2-microglobulin;Beta-2-microglobulin form pI 5.3	B2M	1	5	5	0.012039825	0.373099156	7.343941062
P62304	Small nuclear ribonucleoprotein E	SNRPE	1	4	4	0.037486982	0.427674062	0.558013041
P62888	60S ribosomal protein L30	RPL30	1	4	4	0.016666795	0.391704397	0.618016745
P63208	S-phase kinase-associated protein 1	SKP1	1	7	7	0.030058781	0.402624958	1.965306808
P80217	Interferon-induced 35 kDa protein	IFI35	1	10	10	0.000293898	0.067512497	2.024309699
P80303	Nucleobindin-2;Nesfatin-1	NUCB2	1	9	9	0.012980883	0.373099156	2.445874953
P80723	Brain acid soluble protein 1	BASP1	1	9	9	0.000476206	0.077354383	2.29909654
P98179	RNA-binding protein 3	RBM3	1	6	6	0.020936374	0.399231048	0.654543966
Q01628;C9JQL5	Interferon-induced transmembrane protein 3	IFITM3	2	4	2	0.016368467	0.390268393	3.399937342
Q06323	Proteasome activator complex subunit 1	PSME1	1	20	20	0.027799744	0.402624958	1.939490106

Q07021	Complement component 1 Q subcomponent-binding protein, mitochondrial	C1QBP	1	5	5	0.03605625	0.424613984	1.561643543
Q08380	Galectin-3-binding protein	LGALS3BP	1	25	25	0.016797879	0.391704397	1.538156916
Q13501	Sequestosome-1	SQSTM1	1	10	10	0.012979625	0.373099156	1.539844143
Q14258	E3 ubiquitin/ISG15 ligase TRIM25	TRIM25	1	12	12	0.031295462	0.408593049	1.944859119
Q53GL7	Poly [ADP-ribose] polymerase 10	PARP10	1	17	17	0.000381786	0.067512497	4.291931252
Q63HN8	E3 ubiquitin-protein ligase RNF213	RNF213	1	28	28	0.012327371	0.373099156	2.264909377
Q8N142	Adenylosuccinate synthetase isozyme 1	ADSSL1	1	13	11	0.021324813	0.399231048	0.638814251
Q8NCM8	Cytoplasmic dynein 2 heavy chain 1	DYNC2H1	1	40	40	0.004041879	0.267875641	0.614245806
Q8WXW3	Progesterone-induced-blocking factor 1	PIBF1	1	7	7	0.005407584	0.294228008	1.887868159
Q96PP9;Q8N8V2	Guanylate-binding protein 4	GBP4	2	6	4	0.013878116	0.374319408	3.368361714
Q9BQE5	Apolipoprotein L2	APOL2	1	4	4	0.014548205	0.374319408	2.623198141
Q9BSJ8	Extended synaptotagmin-1	ESYT1	1	13	13	0.0423694	0.454080135	0.572471367
Q9BXJ9	N-alpha-acetyltransferase 15, NatA auxiliary subunit	NAA15	1	6	5	0.024059543	0.402624958	0.663148835
Q9GZN4	Brain-specific serine protease 4	PRSS22	1	5	5	0.037400006	0.427674062	1.631047308
Q9NUL5	UPF0515 protein C19orf66	C19orf66	1	4	4	0.009041755	0.373099156	3.359611417
Q9Y3U8	60S ribosomal protein L36	RPL36	1	7	7	0.033046387	0.417504022	0.661807843

T = 72 h (mock) IFNα7 / Untreated

Uniprot ID	Protein Name	Gene Names	Number of Proteins	Peptides	Unique Peptides	p-Value	p_{FDR}-Value	Ratio of Means (IFNα7 / Untreated)
O14879	Interferon-induced protein with tetratricopeptide repeats 3	IFIT3	1	25	25	0.006212869	0.34318543	30.55864395
P19105;O14950;P24844	Myosin regulatory light chain 12A;Myosin regulatory light chain 12B;Myosin regulatory light polypeptide 9	MYL12A;MYL12B;MYL9	3	9	9	0.028335172	0.459015154	2.733906722
P00167	Cytochrome b5	CYB5A	1	5	5	0.005115131	0.339356985	1.746762864
P01040	Cystatin-A;Cystatin-A, N-terminally processed	CSTA	1	4	4	0.029107059	0.459015154	3.354490039
P01889	HLA class I histocompatibility antigen, B-7 alpha chain	HLA-B	1	20	14	0.008577211	0.37665208	2.659376449
P02792	Ferritin light chain	FTL	1	9	9	0.02838246	0.459015154	1.883436989
P05161	Ubiquitin-like protein ISG15	ISG15	1	8	8	0.001726317	0.218236597	26.86775196
P06753	Tropomyosin alpha-3 chain	TPM3	1	18	9	0.039467533	0.499634955	1.566619905
P09669	Cytochrome c oxidase subunit 6C	COX6C	1	5	5	0.010268519	0.37665208	0.570913833
P10321	HLA class I histocompatibility antigen, Cw-7 alpha chain	HLA-C	1	16	10	0.003506342	0.275702388	1.988640605
P13284	Gamma-interferon-inducible lysosomal thiol reductase	IFI30	1	2	2	0.042887245	0.509047425	1.908110713
P18510	Interleukin-1 receptor antagonist protein	IL1RN	1	5	5	0.014521789	0.405654698	1.754121056
P19525	Interferon-induced, double-stranded RNA-activated protein kinase	EIF2AK2	1	13	13	0.018818799	0.450519213	2.595273281
P19971	Thymidine phosphorylase	TYMP	1	23	23	0.007274303	0.34318543	2.745510007
P20290	Transcription factor BTF3	BTF3	1	5	5	0.018608508	0.450519213	0.602768531
P20591	Interferon-induced GTP-binding protein Mx1;Interferon-induced GTP-binding protein Mx1, N-terminally processed	MX1	1	48	45	7.93148E-05	0.084192678	14.50347907
P28838	Cytosol aminopeptidase	LAP3	1	32	32	0.001683146	0.218236597	1.640370581
P29590	Protein PML	PML	1	33	33	0.001831936	0.218236597	1.650774645
P29728	2-5-oligoadenylate synthase 2	OAS2	1	11	11	0.041323552	0.50419483	4.172354637
P31946	14-3-3 protein beta/alpha;14-3-3 protein beta/alpha, N-terminally	YWHAB	1	20	12	0.032462313	0.460480149	0.609828055
P42224	Signal transducer and activator of transcription 1-alpha/beta	STAT1	1	35	35	0.001504222	0.218236597	3.912337027
P51970	NADH dehydrogenase [ubiquinone] 1 alpha subcomplex subunit 8	NDUFA8	1	6	6	0.043203332	0.509047425	2.133607727
P56715	Oxygen-regulated protein 1	RP1	1	7	7	0.022220522	0.45425557	1.620715219
P60660;P14649	Myosin light polypeptide 6	MYL6	2	10	10	0.027157971	0.459015154	1.606500007
P61221	ATP-binding cassette sub-family E member 1	ABCE1	1	3	3	0.011302956	0.376688624	0.661877177
P62888	60S ribosomal protein L30	RPL30	1	4	4	0.035571529	0.473561144	0.632494997
P80217	Interferon-induced 35 kDa protein	IFI35	1	10	10	0.012065392	0.376688624	1.616822015
P80303	Nucleobindin-2;Nesfatin-1	NUCB2	1	9	9	0.039537764	0.499634955	4.221224617
Q06323	Proteasome activator complex subunit 1	PSME1	1	20	20	0.001391249	0.218236597	1.839533568
Q53GL7	Poly [ADP-ribose] polymerase 10	PARP10	1	17	17	0.047059876	0.520354778	1.74564149
Q8IU85;Q14012	Calcium/calmodulin-dependent protein kinase type 1D	CAMK1D	2	5	5	0.026598844	0.459015154	1.548813565
Q8IYT3	Coiled-coil domain-containing protein 170	CCDC170	1	10	10	0.029473017	0.459015154	1.819387734
Q96NG3	Tetratricopeptide repeat protein 25	TTC25	1	6	6	0.020922743	0.45425557	1.55460126
Q96PP9;Q8N8V2	Guanylate-binding protein 4	GBP4	2	6	4	0.018422317	0.450519213	1.649079963
Q9BPU9	B9 domain-containing protein 2	B9D2	1	4	4	0.042352325	0.509047425	0.570672801
Q9H2W6	39S ribosomal protein L46, mitochondrial	MRPL46	1	2	2	0.044290427	0.513817354	1.550292713
Q9NUL5	UPF0515 protein C19orf66	C19orf66	1	4	4	0.001126517	0.217417709	1.738533016
Q9NUU7;Q9UMR2	ATP-dependent RNA helicase DDX19A;ATP-dependent RNA helicase	DDX19A;DDX19	2	8	8	0.047724276	0.521768328	1.688931401
Q9NVR5	Protein kintoun	DNAAF2	1	12	12	0.016947544	0.442441381	1.576432607

T = 72 h (mock) IFNα16 / Untreated

Uniprot ID	Protein Name	Gene Names	Number of Proteins	Peptides	Unique Peptides	p-Value	p_{FDR}-Value	Ratio of Means (IFNα16 / Untreated)
O14645	Axonemal dynein light intermediate polypeptide 1	DNALI1	1	5	5	0.002955559	0.407291732	1.535389837
O14879	Interferon-induced protein with tetratricopeptide repeats 3	IFIT3	1	25	25	0.034459827	0.485911567	3.339856978
O43837	Isocitrate dehydrogenase [NAD] subunit beta, mitochondrial	IDH3B	1	6	6	0.041844965	0.485911567	1.51321778
P20042	Eukaryotic translation initiation factor 2 subunit 2	EIF2S2	1	6	6	0.032602284	0.485911567	1.725862508
P20591	Interferon-induced GTP-binding protein Mx1;Interferon-induced GTP-binding protein Mx1, N-terminally processed	MX1	1	48	45	0.009471639	0.481372252	2.117747589
P56715	Oxygen-regulated protein 1	RP1	1	7	7	0.037800264	0.485911567	1.642714861
P62861	40S ribosomal protein S30	FAU	1	3	3	0.048417978	0.485911567	0.606939128
Q02978	Mitochondrial 2-oxoglutarate/malate carrier protein	SLC25A11	1	10	10	0.041333329	0.485911567	1.524163567
Q13418	Integrin-linked protein kinase	ILK	1	8	8	0.041612861	0.485911567	1.529839062
Q58FF8	Putative heat shock protein HSP 90-beta 2	HSP90AB2P	1	9	2	0.001957913	0.407291732	0.592146333
Q76M96	Coiled-coil domain-containing protein 80	CCDC80	1	5	5	0.004447545	0.407291732	0.603834897
Q8NE86	Calcium uniporter protein, mitochondrial	MCU	1	6	6	0.037745109	0.485911567	0.656257874
Q8WWI5	Choline transporter-like protein 1	SLC44A1	1	5	5	0.020859409	0.485911567	0.648451825
Q8WXW3	Progesterone-induced-blocking factor 1	PIBF1	1	7	7	0.049419286	0.485911567	2.899737656
Q92688	Acidic leucine-rich nuclear phosphoprotein 32 family member B	ANP32B	1	6	3	0.046594338	0.485911567	0.577465361
Q9H7Z7	Prostaglandin E synthase 2;Prostaglandin E synthase 2 truncated form	PTGES2	1	6	6	0.031905541	0.485911567	0.620081626
Q9UHQ9	NADH-cytochrome b5 reductase 1	CYB5R1	1	8	8	0.045412639	0.485911567	0.650789107
Q9UIF3	Tektin-2	TEKT2	1	12	12	0.024939549	0.485911567	1.521361507
Q9Y224	UPF0568 protein C14orf166	C14orf166	1	9	9	0.026152117	0.485911567	0.660655287

T = 72 h (mock) IFNλ3 / Untreated

Uniprot ID	Protein Name	Gene Names	Number of Proteins	Peptides	Unique Peptides	p-Value	p_{FDR}-Value	Ratio of Means (IFNλ3 / Untreated)
O00468	Agrin;Agrin N-terminal 110 kDa subunit;Agrin C-terminal 110 kDa subunit;Agrin C-terminal 90 kDa fragment;Agrin C-terminal 22 kDa	AGRN	1	34	34	0.012652915	0.374315396	1.509708646
O14879	Interferon-induced protein with tetratricopeptide repeats 3	IFIT3	1	25	25	0.007056448	0.34309969	54.11247446
O14929	Histone acetyltransferase type B catalytic subunit	HAT1	1	4	4	0.016637707	0.398183321	0.663021058
O14933	Ubiquitin/ISG15-conjugating enzyme E2 L6	UBE2L6	1	6	6	0.011625243	0.353739542	3.129789383
O15533	Tapasin	TAPBP	1	6	6	0.009262127	0.353739542	2.484583764
O95433	Activator of 90 kDa heat shock protein ATPase homolog 1	AHSA1	1	9	9	0.02699992	0.418657986	1.526092652
P01889	HLA class I histocompatibility antigen, B-7 alpha chain	HLA-B	1	20	14	0.002266075	0.209858279	3.893287128
P04439	HLA class I histocompatibility antigen, A-3 alpha chain	HLA-A	1	20	13	0.022556962	0.418657986	2.625944466
P05109	Protein S100-A8;Protein S100-A8, N-terminally processed	S100A8	1	8	8	0.021450428	0.418023822	0.642770609
P05161	Ubiquitin-like protein ISG15	ISG15	1	8	8	0.000911702	0.15509589	109.6748655
P08590;P05976	Myosin light chain 3;Myosin light chain 1/3, skeletal muscle isoform	MYL3;MYL1	2	2	2	0.005694641	0.319206533	1.530683234
P13473	Lysosome-associated membrane glycoprotein 2	LAMP2	1	5	5	0.038632964	0.424752924	1.649221141
P15328	Folate receptor alpha	FOLR1	1	7	7	0.027466813	0.418657986	2.174605222
P17900	Ganglioside GM2 activator;Ganglioside GM2 activator isoform short	GM2A	1	3	3	0.023148482	0.418657986	1.504113886
P18510	Interleukin-1 receptor antagonist protein	IL1RN	1	5	5	0.009517084	0.353739542	1.894183621
P19525	Interferon-induced, double-stranded RNA-activated protein kinase	EIF2AK2	1	13	13	0.011572849	0.353739542	3.09337808
P19971	Thymidine phosphorylase	TYMP	1	23	23	0.004023007	0.273776161	4.574044666
P20042	Eukaryotic translation initiation factor 2 subunit 2	EIF2S2	1	6	6	0.045863016	0.446091173	1.830273723
P20591	Interferon-induced GTP-binding protein Mx1;Interferon-induced GTP-binding protein Mx1, N-terminally processed	MX1	1	48	45	0.000390342	0.153729497	28.69350368
P21926	CD9 antigen	CD9	1	4	4	0.014201304	0.384451623	0.530199842
P21980;O43548	Protein-glutamine gamma-glutamyltransferase 2	TGM2	2	31	31	0.011134114	0.353739542	1.8524377
P23381	Tryptophan--tRNA ligase, cytoplasmic;T1-TrpRS;T2-TrpRS	WARS	1	28	28	0.003924113	0.273776161	2.59065283
P23497;Q9H930	Nuclear autoantigen Sp-100	SP100	2	10	10	0.013092881	0.379919316	2.640400308
P28065	Proteasome subunit beta type-9	PSMB9	1	10	10	0.000172154	0.142914	1.707761882
P28072	Proteasome subunit beta type-6	PSMB6	1	7	7	0.010144366	0.353739542	0.635263515
P28838	Cytosol aminopeptidase	LAP3	1	32	32	0.001648569	0.191242366	2.130355149
P29590	Protein PML	PML	1	33	33	0.000505214	0.153729497	2.066053803
P29728	2-5-oligoadenylate synthase 2	OAS2	1	11	11	0.001414438	0.191242366	11.17966877
P41226	Ubiquitin-like modifier-activating enzyme 7	UBA7	1	16	16	0.007248585	0.34309969	1.552859569
P42224	Signal transducer and activator of transcription 1-alpha/beta	STAT1	1	35	35	0.000141431	0.142914	6.100260474
P48449	Lanosterol synthase	LSS	1	6	6	0.034205145	0.42330866	0.65795201
P61221	ATP-binding cassette sub-family E member 1	ABCE1	1	3	3	0.004718172	0.279158537	0.66563832
P62304	Small nuclear ribonucleoprotein E	SNRPE	1	4	4	0.033458027	0.42330866	0.561909406
P80217	Interferon-induced 35 kDa protein	IFI35	1	10	10	0.008553997	0.353739542	2.233171118
Q01628;C9JQL5	Interferon-induced transmembrane protein 3	IFITM3	2	4	2	0.046284577	0.446091173	1.627209665
Q06323	Proteasome activator complex subunit 1	PSME1	1	20	20	0.042163124	0.436280293	1.950282169
Q13835	Plakophilin-1	PKP1	1	11	11	0.043129891	0.437460323	0.486357855
Q14258	E3 ubiquitin/ISG15 ligase TRIM25	TRIM25	1	12	12	0.016396497	0.398183321	1.512841908
Q53GL7	Poly [ADP-ribose] polymerase 10	PARP10	1	17	17	0.027003635	0.418657986	3.428850031
Q63HN8	E3 ubiquitin-protein ligase RNF213	RNF213	1	28	28	0.006962252	0.34309969	2.016794316
Q709C8	Vacuolar protein sorting-associated protein 13C	VPS13C	1	10	10	0.035061739	0.42330866	0.571119183
Q96FV0	Leucine-rich repeat-containing protein 46	LRRC46	1	10	10	0.019211055	0.405144041	0.616940375
Q96PP9;Q8N8V2	Guanylate-binding protein 4	GBP4	2	6	4	0.021508197	0.418023822	2.261329541

Q9BQE5	Apolipoprotein L2	APOL2	1	4	4	0.000946595	0.15509589	2.553647181
Q9NRX4	14 kDa phosphohistidine phosphatase	PHPT1	1	4	4	0.012502374	0.374315396	0.610964135
Q9P258	Protein RCC2	RCC2	1	21	21	0.008773323	0.353739542	0.665604626
Q9UBQ7	Glyoxylate reductase/hydroxypyruvate reductase	GRHPR	1	10	10	0.004651892	0.279158537	0.655753211
Q9Y3U8	60S ribosomal protein L36	RPL36	1	7	7	0.036002204	0.424752924	0.636614651

T = 72 h, Untreated: CoV-2 / mock

Uniprot ID	Protein Name	Gene Names	Number of Proteins	Peptides	Unique Peptides	p-Value	p_{FDR}-Value	Ratio of Means (Untreated: CoV-2 / mock)
O00115	Deoxyribonuclease-2-alpha	DNASE2	1	5	5	1.21552E-05	0.025878526	0.639459183
O00391	Sulfhydryl oxidase 1	QSOX1	1	17	17	0.000745799	0.132805329	0.218535338
O00468	Agrin;Agrin N-terminal 110 kDa subunit;Agrin C-terminal 110 kDa subunit;Agrin C-terminal 90 kDa fragment;Agrin C-terminal 22	AGRN	1	34	34	0.032178843	0.337481561	0.487312691
O00560	Syntenin-1	SDCBP	1	8	8	0.042225878	0.358084664	0.514196138
O14976	Cyclin-G-associated kinase	GAK	1	6	6	0.018924005	0.29039315	1.811366737
O43490	Prominin-1	PROM1	1	27	27	0.00126288	0.143131287	0.129958059
O43653	Prostate stem cell antigen	PSCA	1	4	4	0.0441009	0.358084664	0.230569553
O43920	NADH dehydrogenase [ubiquinone] iron-sulfur protein 5	NDUFS5	1	5	5	0.02186853	0.302933694	1.699742177
O60635	Tetraspanin-1	TSPAN1	1	3	3	0.00022668	0.132805329	0.321445585
O75915	PRA1 family protein 3	ARL6IP5	1	3	3	0.008616345	0.229302483	1.850588844
O95436	Sodium-dependent phosphate transport protein 2B	SLC34A2	1	12	12	0.014870196	0.280165026	0.446893802
P00450;CON__ENSEMBL:ENSBTAP00000031900	Ceruloplasmin	CP	2	31	31	0.005267688	0.190083185	0.360068258
P00751	Complement factor B;Complement factor B Ba	CFB	1	40	40	0.002030715	0.147211642	0.241071833
P01024;CON__Q2UVX4	Complement C3;Complement C3 beta chain;C3-beta-c;Complement C3 alpha chain;C3a anaphylatoxin;Acylation stimulating protein;Complement C3b alpha chain;Complement C3c alpha chain fragment 1;Complement C3dg	C3	2	100	100	0.002582414	0.148593491	0.202003045
P01033	Metalloproteinase inhibitor 1	TIMP1	1	7	7	0.003902972	0.172375303	0.371612233
P01034	Cystatin-C	CST3	1	4	4	0.008036183	0.220784206	0.235054301
P01833	Polymeric immunoglobulin receptor;Secretory component	PIGR	1	42	42	0.002118885	0.147211642	0.185626117
P03973	Antileukoproteinase	SLPI	1	5	5	0.008932177	0.231909821	0.219711683
P04844	Dolichyl-diphosphooligosaccharide--protein glycosyltransferase	RPN2	1	8	8	0.002989257	0.163182781	1.793000905
P07919;A0A096LP55	Cytochrome b-c1 complex subunit 6, mitochondrial	UQCRH	2	5	5	0.025327477	0.302933694	2.754802722
P08195	4F2 cell-surface antigen heavy chain	SLC3A2	1	5	5	0.020546748	0.292436854	1.634079964
P08571	Monocyte differentiation antigen CD14;Monocyte differentiation antigen CD14, urinary form;Monocyte differentiation antigen	CD14	1	7	7	0.013774375	0.264640176	0.305238448
P09668	Pro-cathepsin H;Cathepsin H mini chain;Cathepsin H;Cathepsin H	CTSH	1	11	11	0.001652287	0.147211642	0.648788216
P12273	Prolactin-inducible protein	PIP	1	6	6	0.015903561	0.282748161	0.327424214
P13987	CD59 glycoprotein	CD59	1	4	4	0.036599335	0.339620278	0.482693024
P14780	Matrix metalloproteinase-9;67 kDa matrix metalloproteinase-	MMP9	1	13	13	0.024564876	0.302933694	0.28164335
P15941	Mucin-1;Mucin-1 subunit alpha;Mucin-1 subunit beta	MUC1	1	9	9	0.0021482	0.147211642	0.304914696
P20042	Eukaryotic translation initiation factor 2 subunit 2	EIF2S2	1	6	6	0.046437959	0.359380406	1.749709984
P20061	Transcobalamin-1	TCN1	1	8	8	0.041948828	0.358084664	0.514675452
P25685;Q9UDY4	DnaJ homolog subfamily B member 1	DNAJB1	2	13	13	0.010595555	0.242558463	1.540868516
P27105	Erythrocyte band 7 integral membrane protein	STOM	1	15	15	0.024936472	0.302933694	0.544895515
P27701	CD82 antigen	CD82	1	4	4	0.032841172	0.337714828	0.488273223
P31949	Protein S100-A11;Protein S100-A11, N-terminally processed	S100A11	1	2	2	0.036700746	0.339620278	0.607715561
P43353;P48448	Aldehyde dehydrogenase family 3 member B1	ALDH3B1	2	15	14	0.023166916	0.302933694	0.526189677
P49788	Retinoic acid receptor responder protein 1	RARRES1	1	8	8	0.048456862	0.363255844	0.574980624
P54707	Potassium-transporting ATPase alpha chain 2	ATP12A	1	31	27	0.006565002	0.206992477	0.577939665

P54727	UV excision repair protein RAD23 homolog B	RAD23B	1	7	7	0.043640081	0.358084664	1.713821319
P55058	Phospholipid transfer protein	PLTP	1	11	11	0.02018703	0.29039315	0.425869883
P60903	Protein S100-A10	S100A10	1	5	5	0.035729566	0.339135786	0.552206392
P61916	Epididymal secretory protein E1	NPC2	1	2	2	0.005171072	0.189814	0.632088248
P63092;Q5JWF2;P38405;P19087;A8MTJ3;P11488	Guanine nucleotide-binding protein G(s) subunit alpha isoforms short;Guanine nucleotide-binding protein G(s) subunit alpha isoforms XLas	GNAS	6	9	8	0.015864924	0.282748161	0.586014583
P80188	Neutrophil gelatinase-associated lipocalin	LCN2	1	16	16	0.000538344	0.132805329	0.326826597
Q03169	Tumor necrosis factor alpha-induced protein 2	TNFAIP2	1	15	15	0.0037648	0.172375303	1.776038258
Q07666	KH domain-containing, RNA-binding, signal transduction-	KHDRBS1	1	11	11	0.044234977	0.358084664	1.589647974
Q08380	Galectin-3-binding protein	LGALS3BP	1	25	25	0.00235469	0.147211642	0.467354093
Q08431	Lactadherin;Lactadherin short form;Medin	MFGE8	1	5	5	0.005132616	0.189814	0.624489326
Q12805	EGF-containing fibulin-like extracellular matrix protein 1	EFEMP1	1	14	14	0.019265442	0.29039315	0.131075047
Q13724	Mannosyl-oligosaccharide glucosidase	MOGS	1	12	12	0.039607582	0.348450785	1.603024649
Q13751	Laminin subunit beta-3	LAMB3	1	20	20	0.018285564	0.29039315	0.395197968
Q16270	Insulin-like growth factor-binding protein 7	IGFBP7	1	6	6	0.004644226	0.183102895	0.166752721
Q16348	Solute carrier family 15 member 2	SLC15A2	1	4	4	0.038518017	0.348450785	0.508412617
Q16787	Laminin subunit alpha-3	LAMA3	1	8	8	0.039917267	0.349727827	0.51421513
Q53GD3	Choline transporter-like protein 4	SLC44A4	1	5	5	0.007860996	0.220784206	0.25077206
Q6NVY1	3-hydroxyisobutyryl-CoA hydrolase, mitochondrial	HIBCH	1	6	6	0.022032558	0.302933694	0.615791531
Q6UXY8	Transmembrane channel-like protein 5	TMC5	1	19	19	0.002184324	0.147211642	0.299694792
Q7Z404	Transmembrane channel-like protein 4	TMC4	1	16	16	0.001435693	0.147211642	0.211189174
Q8IWA5	Choline transporter-like protein 2	SLC44A2	1	5	5	0.006125582	0.200636385	0.592188274
Q8NFJ5	Retinoic acid-induced protein 3	GPRC5A	1	5	5	0.028005598	0.31772678	0.343282107
Q8TDL5	BPI fold-containing family B member 1	BPIFB1	1	30	30	0.006034713	0.200636385	0.203135623
Q8WXI7	Mucin-16	MUC16	1	39	39	0.001806616	0.147211642	0.069632828
Q99102	Mucin-4;Mucin-4 alpha chain;Mucin-4 beta chain	MUC4	1	37	37	0.00424091	0.18057795	0.251637985
Q99828	Calcium and integrin-binding protein 1	CIB1	1	10	10	0.016801031	0.282748161	0.452608989
Q9BW30	Tubulin polymerization-promoting protein family member 3	TPPP3	1	13	13	0.002420107	0.147211642	0.638953157
Q9H223	EH domain-containing protein 4	EHD4	1	21	17	0.027599497	0.317497664	0.665099286
Q9NP55	BPI fold-containing family A member 1	BPIFA1	1	11	11	0.002211361	0.147211642	0.275526535
Q9NQ84	G-protein coupled receptor family C group 5 member C	GPRC5C	1	5	5	0.010342026	0.241957948	0.253627269
Q9P035	Very-long-chain (3R)-3-hydroxyacyl-CoA dehydratase 3	HACD3	1	7	7	0.024848697	0.302933694	1.928953096
Q9UGM3	Deleted in malignant brain tumors 1 protein	DMBT1	1	14	14	0.001802563	0.147211642	0.336818366
Q9UMX0	Ubiquilin-1	UBQLN1	1	10	5	0.01178661	0.253471652	1.51299425
Q9Y376	Calcium-binding protein 39	CAB39	1	8	7	0.04613586	0.359380406	0.458012712

T = 72 h, IFNα5: CoV-2 / mock

Uniprot ID	Protein Name	Gene Names	Number of Proteins	Peptides	Unique Peptides	p-Value	p_{FDR}-Value	Ratio of Means (IFNα5: CoV-2 / mock)
K7EJ46	Small integral membrane protein 22	SMIM22	1	2	2	0.002740305	0.288025456	0.313748798
O00391	Sulfhydryl oxidase 1	QSOX1	1	17	17	0.003106764	0.288025456	0.285362167
O00468	Agrin;Agrin N-terminal 110 kDa subunit;Agrin C-terminal 110 kDa subunit;Agrin C-terminal 90 kDa fragment;Agrin C-terminal	AGRN	1	34	34	0.012509333	0.330619128	0.49953356
O00515	Ladinin-1	LAD1	1	13	13	0.006604967	0.317105083	1.772154136
O14976	Cyclin-G-associated kinase	GAK	1	6	6	0.016189725	0.330619128	0.487838312
O15020	Spectrin beta chain, non-erythrocytic 2	SPTBN2	1	27	22	0.029470343	0.395690981	1.855365315
O15160	DNA-directed RNA polymerases I and III subunit RPAC1	POLR1C	1	5	5	0.003000672	0.288025456	1.547469605
O43490	Prominin-1	PROM1	1	27	27	0.029120434	0.395690981	0.08098288
O60635	Tetraspanin-1	TSPAN1	1	3	3	0.031832399	0.411672988	0.290533431
O95436	Sodium-dependent phosphate transport protein 2B	SLC34A2	1	12	12	0.048698953	0.435226874	0.4226145
P00450;CON__ENSEMBL:ENSBTAP00000031900	Ceruloplasmin	CP	2	31	31	0.009649749	0.330619128	0.299197042
P00751	Complement factor B;Complement factor B Ba	CFB	1	40	40	0.000609792	0.257088415	0.204153325
P01024;CON__Q2UVX4	Complement C3;Complement C3 beta chain;C3-beta-c;Complement C3 alpha chain;C3a anaphylatoxin;Acylation stimulating protein;Complement C3b alpha chain;Complement C3c alpha chain fragment 1;Complement C3dg	C3	2	100	100	0.001639101	0.287935406	0.22096803
P01034	Cystatin-C	CST3	1	4	4	0.015234586	0.330619128	0.283507231
P01833	Polymeric immunoglobulin receptor;Secretory component	PIGR	1	42	42	0.007669066	0.330619128	0.171836143
P01903;P01906	HLA class II histocompatibility antigen, DR alpha chain	HLA-DRA	2	8	8	0.033173992	0.412427077	0.491683859
P03973	Antileukoproteinase	SLPI	1	5	5	0.000832742	0.287935406	0.188560468
P06396;REV__Q6T	Gelsolin	GSN	2	42	20	0.004070283	0.288025456	0.638374388
P07339	Cathepsin D;Cathepsin D light chain;Cathepsin D heavy chain	CTSD	1	23	23	0.045170335	0.428914715	0.600004208
P07947;P06241	Tyrosine-protein kinase Yes	YES1	2	7	3	9.57765E-05	0.201896958	0.568069253
P08195	4F2 cell-surface antigen heavy chain	SLC3A2	1	5	5	0.003841787	0.288025456	1.938323986
P08571	Monocyte differentiation antigen CD14;Monocyte differentiation antigen CD14, urinary form;Monocyte	CD14	1	7	7	0.005234699	0.299513101	0.328448273
P08603;Q03591;Q02985	Complement factor H	CFH	3	23	23	0.046552893	0.43230616	0.244679603
P09668	Pro-cathepsin H;Cathepsin H mini chain;Cathepsin H;Cathepsin	CTSH	1	11	11	0.018279034	0.340992948	0.52960497
P10909	Clusterin;Clusterin beta chain;Clusterin alpha chain	CLU	1	22	22	0.008921609	0.330619128	0.536591915
P14780	Matrix metalloproteinase-9;67 kDa matrix metalloproteinase-	MMP9	1	13	13	0.039496354	0.412427077	0.249655237
P15907	Beta-galactoside alpha-2,6-sialyltransferase 1	ST6GAL1	1	4	4	0.009507056	0.330619128	0.400605866
P15941	Mucin-1;Mucin-1 subunit alpha;Mucin-1 subunit beta	MUC1	1	9	9	0.000506554	0.257088415	0.293298754
P18077	60S ribosomal protein L35a	RPL35A	1	5	5	0.023157284	0.369786587	1.693101745
P18827	Syndecan-1	SDC1	1	3	3	0.038695331	0.412427077	1.72351264
P24821	Tenascin	TNC	1	47	47	0.041759111	0.412427077	0.657422988
P25774	Cathepsin S	CTSS	1	14	14	0.009369469	0.330619128	0.617425931

P27105	Erythrocyte band 7 integral membrane protein	STOM	1	15	15	0.028173236	0.393305833	0.47112012
P35637	RNA-binding protein FUS	FUS	1	8	6	0.032414703	0.412427077	0.648651198
P42704	Leucine-rich PPR motif-containing protein, mitochondrial	LRPPRC	1	19	19	0.001932763	0.288025456	1.50755873
P43353;P48448	Aldehyde dehydrogenase family 3 member B1	ALDH3B1	2	15	14	0.029682634	0.396018937	0.536039802
P46060	Ran GTPase-activating protein 1	RANGAP1	1	13	13	0.029412188	0.395690981	1.525787958
P49788	Retinoic acid receptor responder protein 1	RARRES1	1	8	8	0.019159964	0.347218672	0.586359088
P53365	Arfaptin-2	ARFIP2	1	6	6	0.016600491	0.330619128	0.662182816
P54707	Potassium-transporting ATPase alpha chain 2	ATP12A	1	31	27	0.019780647	0.353369527	0.429528194
P55789	FAD-linked sulfhydryl oxidase ALR	GFER	1	4	4	0.034126477	0.412427077	1.691181794
P80188	Neutrophil gelatinase-associated lipocalin	LCN2	1	16	16	0.019177146	0.347218672	0.344891056
P80303	Nucleobindin-2;Nesfatin-1	NUCB2	1	9	9	0.003062405	0.288025456	0.226652242
P98175	RNA-binding protein 10	RBM10	1	5	5	0.01173493	0.330619128	1.532042693
Q03169	Tumor necrosis factor alpha-induced protein 2	TNFAIP2	1	15	15	0.002560438	0.288025456	2.061186873
Q08380	Galectin-3-binding protein	LGALS3BP	1	25	25	0.000481962	0.257088415	0.507610953
Q08AF3;Q7Z7L1;Q68D06	Schlafen family member 5	SLFN5	3	5	5	0.034884193	0.412427077	1.665004103
Q12805	EGF-containing fibulin-like extracellular matrix protein 1	EFEMP1	1	14	14	0.004099034	0.288025456	0.155713191
Q15833	Syntaxin-binding protein 2	STXBP2	1	18	18	0.020709411	0.35783147	0.66101332
Q16270	Insulin-like growth factor-binding protein 7	IGFBP7	1	6	6	0.007351815	0.330619128	0.238777144
Q53GD3	Choline transporter-like protein 4	SLC44A4	1	5	5	0.023589373	0.369786587	0.27608049
Q56VL3	OCIA domain-containing protein 2	OCIAD2	1	3	3	0.046829842	0.432970642	0.62038494
Q6NVY1	3-hydroxyisobutyryl-CoA hydrolase, mitochondrial	HIBCH	1	6	6	0.040082045	0.412427077	0.652110209
Q6UN15	Pre-mRNA 3-end-processing factor FIP1	FIP1L1	1	8	8	0.041854003	0.412427077	1.655961011
Q6UXY8	Transmembrane channel-like protein 5	TMC5	1	19	19	0.00361148	0.288025456	0.296974978
Q6ZNW5	GDP-D-glucose phosphorylase 1	GDPGP1	1	8	8	0.009231319	0.330619128	1.529930546
Q7Z404	Transmembrane channel-like protein 4	TMC4	1	16	16	0.023612737	0.369786587	0.216349934
Q7Z4L5	Tetratricopeptide repeat protein 21B	TTC21B	1	4	4	0.01256979	0.330619128	1.522324087
Q8IXT5	RNA-binding protein 12B	RBM12B	1	7	7	0.011441265	0.330619128	1.512561907
Q8N1D5	Uncharacterized protein C1orf158	C1orf158	1	5	5	0.022817704	0.369786587	0.535646423
Q8TDL5	BPI fold-containing family B member 1	BPIFB1	1	30	30	0.010335807	0.330619128	0.212823814
Q8WXI7	Mucin-16	MUC16	1	39	39	0.001534542	0.287935406	0.043738364
Q99102	Mucin-4;Mucin-4 alpha chain;Mucin-4 beta chain	MUC4	1	37	37	0.004648166	0.296919234	0.264260464
Q99538	Legumain	LGMN	1	6	6	0.017845554	0.33657013	0.613255599
Q99828	Calcium and integrin-binding protein 1	CIB1	1	10	10	0.047437664	0.434776507	0.499098535
Q9BX68	Histidine triad nucleotide-binding protein 2, mitochondrial	HINT2	1	3	3	0.013469002	0.330619128	0.432764661
Q9H008	Phospholysine phosphohistidine inorganic pyrophosphate	LHPP	1	4	4	0.045496373	0.42903768	0.573800815
Q9H977	WD repeat-containing protein 54	WDR54	1	7	7	0.024003102	0.369786587	1.561904496
Q9NP55	BPI fold-containing family A member 1	BPIFA1	1	11	11	0.038290118	0.412427077	0.235493704
Q9NR30	Nucleolar RNA helicase 2	DDX21	1	5	4	0.036721591	0.412427077	1.557630357
Q9NUQ8	ATP-binding cassette sub-family F member 3	ABCF3	1	10	10	0.02304964	0.369786587	1.652738066
Q9UGM3	Deleted in malignant brain tumors 1 protein	DMBT1	1	14	14	0.043468197	0.41650436	0.279556305
Q9UK41	Vacuolar protein sorting-associated protein 28 homolog	VPS28	1	10	10	0.005257109	0.299513101	0.651182972
Q9ULC4	Malignant T-cell-amplified sequence 1	MCTS1	1	4	4	0.037626883	0.412427077	1.587109717
Q9UN37	Vacuolar protein sorting-associated protein 4A	VPS4A	1	7	5	0.020673904	0.35783147	1.683005381
Q9Y376	Calcium-binding protein 39	CAB39	1	8	7	0.006015902	0.309305406	0.414821548

Q9Y3D6	Mitochondrial fission 1 protein	FIS1	1	4	4	0.025796051	0.382944199	0.648692464
Q9Y6E0;O00506	Serine/threonine-protein kinase 24;Serine/threonine-protein kinase 24 36 kDa subunit;Serine/threonine-protein kinase 24 12	STK24	2	11	6	0.030166551	0.399943965	0.637551109

T = 72 h, IFNα7: CoV-2 / mock

Uniprot ID	Protein Name	Gene Names	Number of Proteins	Peptides	Unique Peptides	p-Value	p_{FDR}-Value	Ratio of Means (IFNα7: CoV-2 / mock)
K7EJ46	Small integral membrane protein 22	SMIM22	1	2	2	0.013233528	0.378280486	0.584188577
O00391	Sulfhydryl oxidase 1	QSOX1	1	17	17	0.003156094	0.236205432	0.220184745
O00468	Agrin;Agrin N-terminal 110 kDa subunit;Agrin C-terminal 110 kDa subunit;Agrin C-terminal 90 kDa fragment;Agrin C-terminal 22 kDa	AGRN	1	34	34	0.004010734	0.238959236	0.530878147
O00560	Syntenin-1	SDCBP	1	8	8	0.020716204	0.450471745	0.413778252
O43490	Prominin-1	PROM1	1	27	27	0.000548348	0.192812914	0.159392851
O43795	Unconventional myosin-Ib	MYO1B	1	22	19	0.005148847	0.261242706	0.663516738
O60635	Tetraspanin-1	TSPAN1	1	3	3	0.008777888	0.346401483	0.259532191
O95436	Sodium-dependent phosphate transport protein 2B	SLC34A2	1	12	12	0.005932053	0.287300093	0.479756762
P00450;CON__ENSEMBL:ENSBTAP00000031900	Ceruloplasmin	CP	2	31	31	0.002660453	0.234975264	0.353611139
P00751	Complement factor B;Complement factor B Ba fragment;Complement	CFB	1	40	40	0.001313498	0.222290474	0.247291629
P01024;CON__Q2UVX4	Complement C3;Complement C3 beta chain;C3-beta-c;Complement C3 alpha chain;C3a anaphylatoxin;Acylation stimulating protein;Complement C3b alpha chain;Complement C3c alpha chain fragment 1;Complement C3dg fragment;Complement C3g	C3	2	100	100	0.000274094	0.192812914	0.210756052
P01033	Metalloproteinase inhibitor 1	TIMP1	1	7	7	0.008498643	0.346401483	0.370665393
P01833	Polymeric immunoglobulin receptor;Secretory component	PIGR	1	42	42	0.024106707	0.467012652	0.263377571
P03973	Antileukoproteinase	SLPI	1	5	5	0.030877818	0.476816161	0.16870288
P04843	Dolichyl-diphosphooligosaccharide--protein glycosyltransferase	RPN1	1	18	18	0.029437554	0.470398191	1.68167501
P06396;REV__Q6TDU7	Gelsolin	GSN	2	42	20	0.001241682	0.222290474	0.617990051
P06731;P31997;P40198	Carcinoembryonic antigen-related cell adhesion molecule 5	CEACAM5	3	13	11	0.034461014	0.497134445	0.548029653
P07948;P08631	Tyrosine-protein kinase Lyn	LYN	2	14	12	0.020238872	0.444629244	0.653169788
P08603;Q03591;Q02985	Complement factor H	CFH	3	23	23	0.000174155	0.185561961	0.213588158
P10909	Clusterin;Clusterin beta chain;Clusterin alpha chain	CLU	1	22	22	0.028724951	0.470398191	0.557364502
P12273	Prolactin-inducible protein	PIP	1	6	6	0.018255698	0.425136407	0.297486888
P14780	Matrix metalloproteinase-9;67 kDa matrix metalloproteinase-9;82	MMP9	1	13	13	0.023755456	0.467012652	0.270133942
P15941	Mucin-1;Mucin-1 subunit alpha;Mucin-1 subunit beta	MUC1	1	9	9	6.50027E-05	0.138520753	0.310128779
P20061	Transcobalamin-1	TCN1	1	8	8	0.004148987	0.238959236	0.378539432
P27105	Erythrocyte band 7 integral membrane protein	STOM	1	15	15	0.027835841	0.467572955	0.546884498
P27701	CD82 antigen	CD82	1	4	4	0.001577834	0.222290474	0.411711915
P29992	Guanine nucleotide-binding protein subunit alpha-11	GNA11	1	10	4	0.025742083	0.467572955	0.400964719
P35241	Radixin	RDX	1	21	6	0.008611535	0.346401483	0.489248528
P43353;P48448	Aldehyde dehydrogenase family 3 member B1	ALDH3B1	2	15	14	0.011869246	0.366570492	0.59049712
P49788	Retinoic acid receptor responder protein 1	RARRES1	1	8	8	0.023325166	0.464541393	0.534136643
P50148	Guanine nucleotide-binding protein G(q) subunit alpha	GNAQ	1	10	5	0.047539739	0.551336863	0.570758944
P54707	Potassium-transporting ATPase alpha chain 2	ATP12A	1	31	27	0.002343964	0.234975264	0.523855102
P80188	Neutrophil gelatinase-associated lipocalin	LCN2	1	16	16	0.001379596	0.222290474	0.313769046

P98160	Basement membrane-specific heparan sulfate proteoglycan core	HSPG2	1	40	40	0.032689798	0.496884637	0.44074221
Q02386	Zinc finger protein 45	ZNF45	1	2	2	0.009716211	0.353581747	0.261123097
Q03169	Tumor necrosis factor alpha-induced protein 2	TNFAIP2	1	15	15	0.011629664	0.364453142	1.638050764
Q08380	Galectin-3-binding protein	LGALS3BP	1	25	25	0.001536404	0.222290474	0.508017784
Q12805	EGF-containing fibulin-like extracellular matrix protein 1	EFEMP1	1	14	14	0.003574789	0.238959236	0.166529381
Q16270	Insulin-like growth factor-binding protein 7	IGFBP7	1	6	6	0.004431083	0.239246986	0.167699053
Q53GD3	Choline transporter-like protein 4	SLC44A4	1	5	5	0.007498647	0.34344822	0.295696649
Q6UXY8	Transmembrane channel-like protein 5	TMC5	1	19	19	0.004490793	0.239246986	0.382145396
Q7Z404	Transmembrane channel-like protein 4	TMC4	1	16	16	0.00063336	0.192812914	0.196775267
Q8TDL5	BPI fold-containing family B member 1	BPIFB1	1	30	30	0.004146615	0.238959236	0.224153742
Q8WXI7	Mucin-16	MUC16	1	39	39	0.001311534	0.222290474	0.08219263
Q92522	Histone H1x	H1FX	1	5	5	0.015838202	0.399373336	1.633754479
Q99102	Mucin-4;Mucin-4 alpha chain;Mucin-4 beta chain	MUC4	1	37	37	0.001506161	0.222290474	0.29091275
Q99816	Tumor susceptibility gene 101 protein	TSG101	1	10	10	0.014466414	0.399373336	0.637191251
Q9NP55	BPI fold-containing family A member 1	BPIFA1	1	11	11	0.003689341	0.238959236	0.238277118
Q9Y2A9	N-acetyllactosaminide beta-1,3-N-acetylglucosaminyltransferase 3	B3GNT3	1	6	6	0.04882048	0.551336863	0.651503596
Q9Y376	Calcium-binding protein 39	CAB39	1	8	7	0.042143996	0.532092202	0.551416886
Q9Y6R7	IgGFc-binding protein	FCGBP	1	34	34	0.025551026	0.467572955	0.146573974

T = 72 h, IFNα16: CoV-2 / mock

Uniprot ID	Protein Name	Gene Names	Number of Proteins	Peptides	Unique Peptides	p-Value	p_{FDR}-Value	Ratio of Means (IFNα16: CoV-2 / mock)
B0FP48;E5RIL1	Uroplakin-3b-like protein	UPK3BL	2	5	5	0.033337394	0.389460499	0.631772121
O00391	Sulfhydryl oxidase 1	QSOX1	1	17	17	0.002296359	0.162354374	0.209702132
O00468	Agrin;Agrin N-terminal 110 kDa subunit;Agrin C-terminal 110 kDa subunit;Agrin C-terminal 90 kDa fragment;Agrin	AGRN	1	34	34	0.01717762	0.335368745	0.436733979
O00560	Syntenin-1	SDCBP	1	8	8	0.034458534	0.389460499	0.41415854
O43286;Q9UBX8	Beta-1,4-galactosyltransferase 5	B4GALT5	2	7	7	0.042793183	0.401258054	0.645814963
O43490	Prominin-1	PROM1	1	27	27	0.006692268	0.249884497	0.126295658
O43653	Prostate stem cell antigen	PSCA	1	4	4	0.008527582	0.262369724	0.192342597
O60635	Tetraspanin-1	TSPAN1	1	3	3	0.01899516	0.335368745	0.283412222
O60936	Nucleolar protein 3	NOL3	1	5	5	0.010597543	0.288904507	2.12840721
O95436	Sodium-dependent phosphate transport protein 2B	SLC34A2	1	12	12	0.025981164	0.381306818	0.484590914
P00387	NADH-cytochrome b5 reductase 3;NADH-cytochrome b5 reductase 3 membrane-bound form;NADH-cytochrome	CYB5R3	1	9	9	0.023714714	0.361952309	1.567607794
P00450;CON__ENSEMBL:ENSBTAP00000031900	Ceruloplasmin	CP	2	31	31	0.023186352	0.359937405	0.325317694
P00751	Complement factor B;Complement factor B Ba	CFB	1	40	40	0.000245893	0.129807789	0.197023795
P00918	Carbonic anhydrase 2	CA2	1	5	5	0.02777449	0.381706815	1.508541789
P01024;CON__Q2UVX4	Complement C3;Complement C3 beta chain;C3-beta-c;Complement C3 alpha chain;C3a anaphylatoxin;Acylation stimulating protein;Complement C3b alpha chain;Complement C3c alpha chain fragment	C3	2	100	100	0.002200698	0.162354374	0.233942999
P01033	Metalloproteinase inhibitor 1	TIMP1	1	7	7	0.010622249	0.288904507	0.249356257
P01034	Cystatin-C	CST3	1	4	4	0.001643659	0.156222299	0.34130657
P01833	Polymeric immunoglobulin receptor;Secretory	PIGR	1	42	42	0.000900054	0.156222299	0.155584434
P03973	Antileukoproteinase	SLPI	1	5	5	0.014963481	0.309949751	0.204699144
P07602	Prosaposin;Saposin-A;Saposin-B-Val;Saposin-B;Saposin-	PSAP	1	11	11	0.027210772	0.381706815	0.436991153
P08243	Asparagine synthetase [glutamine-hydrolyzing]	ASNS	1	3	3	0.031571023	0.387740113	1.911575103
P08603;Q03591;Q02985	Complement factor H	CFH	3	23	23	0.006305837	0.249884497	0.191430627
P09622	Dihydrolipoyl dehydrogenase, mitochondrial	DLD	1	12	12	0.041140445	0.39553897	0.606955811
P09668	Pro-cathepsin H;Cathepsin H mini chain;Cathepsin	CTSH	1	11	11	0.03265253	0.389460499	0.588823443
P13987	CD59 glycoprotein	CD59	1	4	4	0.020564549	0.341593447	0.27689209
P15941	Mucin-1;Mucin-1 subunit alpha;Mucin-1 subunit beta	MUC1	1	9	9	0.002331873	0.162354374	0.330929466
P18077	60S ribosomal protein L35a	RPL35A	1	5	5	0.041426607	0.39553897	1.566009197
P18827	Syndecan-1	SDC1	1	3	3	0.01883918	0.335368745	1.68454312
P20061	Transcobalamin-1	TCN1	1	8	8	0.024661818	0.368341869	0.314363486
P20674	Cytochrome c oxidase subunit 5A, mitochondrial	COX5A	1	5	5	0.027055505	0.381706815	1.705333643
P27105	Erythrocyte band 7 integral membrane protein	STOM	1	15	15	0.027290651	0.381706815	0.458062603

P27701	CD82 antigen	CD82	1	4	4	0.001373097	0.156222299	0.318562608
P41212	Transcription factor ETV6	ETV6	1	6	6	8.55466E-05	0.089439014	1.681342984
P43353;P48448	Aldehyde dehydrogenase family 3 member B1	ALDH3B1	2	15	14	0.046732226	0.410575989	0.525406255
P46976	Glycogenin-1	GYG1	1	6	6	0.005282889	0.230135847	1.681295864
P51148	Ras-related protein Rab-5C	RAB5C	1	7	4	0.023238426	0.359937405	0.652369418
P51687	Sulfite oxidase, mitochondrial	SUOX	1	6	6	0.028066497	0.381706815	1.560984272
P51857	3-oxo-5-beta-steroid 4-dehydrogenase	AKR1D1	1	2	2	0.038405647	0.395431978	1.71777836
P52943	Cysteine-rich protein 2	CRIP2	1	3	3	0.006090028	0.249691139	0.621354065
P54707	Potassium-transporting ATPase alpha chain 2	ATP12A	1	31	27	0.018925162	0.335368745	0.49850936
P55058	Phospholipid transfer protein	PLTP	1	11	11	0.045839085	0.406142065	0.621195832
P55957	BH3-interacting domain death agonist;BH3-interacting domain death agonist p15;BH3-interacting domain death	BID	1	7	7	0.010284848	0.288904507	1.900101141
P61916	Epididymal secretory protein E1	NPC2	1	2	2	0.000886916	0.156222299	0.487487805
P62753	40S ribosomal protein S6	RPS6	1	9	9	0.011070867	0.288904507	1.617600758
P62861	40S ribosomal protein S30	FAU	1	3	3	0.01169932	0.288904507	2.310289122
P62873;P16520	Guanine nucleotide-binding protein G(I)/G(S)/G(T)	GNB1	2	21	13	0.040280843	0.39553897	0.600605374
P62879	Guanine nucleotide-binding protein G(I)/G(S)/G(T)	GNB2	1	22	13	0.012849411	0.288904507	0.620130646
P63167	Dynein light chain 1, cytoplasmic	DYNLL1	1	5	2	0.007651762	0.256332711	0.543341803
P80188	Neutrophil gelatinase-associated lipocalin	LCN2	1	16	16	0.006816759	0.250067438	0.329969015
P98160	Basement membrane-specific heparan sulfate	HSPG2	1	40	40	0.007422035	0.25441762	0.401492956
Q03169	Tumor necrosis factor alpha-induced protein 2	TNFAIP2	1	15	15	0.001777318	0.16158136	1.909309407
Q08380	Galectin-3-binding protein	LGALS3BP	1	25	25	0.001585858	0.156222299	0.523509991
Q08AF3;Q7Z7L1;Q68D06	Schlafen family member 5	SLFN5	3	5	5	0.049657167	0.413677833	1.539539382
Q12805	EGF-containing fibulin-like extracellular matrix protein 1	EFEMP1	1	14	14	0.002462952	0.162354374	0.132825436
Q13557	Calcium/calmodulin-dependent protein kinase type II	CAMK2D	1	12	7	0.044475787	0.406142065	0.658822411
Q13561	Dynactin subunit 2	DCTN2	1	12	12	0.004701784	0.217604496	1.770010852
Q15428	Splicing factor 3A subunit 2	SF3A2	1	5	5	0.044524991	0.406142065	1.872995119
Q16270	Insulin-like growth factor-binding protein 7	IGFBP7	1	6	6	0.002588254	0.164001207	0.218205392
Q53GD3	Choline transporter-like protein 4	SLC44A4	1	5	5	0.001364897	0.156222299	0.311037996
Q5T601	Probable G-protein coupled receptor 110	GPR110	1	4	4	0.01750817	0.335368745	0.537086046
Q6UXY8	Transmembrane channel-like protein 5	TMC5	1	19	19	0.000495091	0.147890813	0.39353385
Q7Z404	Transmembrane channel-like protein 4	TMC4	1	16	16	0.014094423	0.299911535	0.272668046
Q8IWA5	Choline transporter-like protein 2	SLC44A2	1	5	5	0.04096796	0.39553897	0.585698035
Q8NFJ5	Retinoic acid-induced protein 3	GPRC5A	1	5	5	0.001280462	0.156222299	0.271327341
Q8TDL5	BPI fold-containing family B member 1	BPIFB1	1	30	30	0.003362334	0.190510668	0.219695516
Q8WXH0;Q8NF9	Nesprin-2	SYNE2	2	6	6	0.02837955	0.381706815	0.600304236
Q8WXI7	Mucin-16	MUC16	1	39	39	6.11696E-05	0.089439014	0.067913747
Q99102	Mucin-4;Mucin-4 alpha chain;Mucin-4 beta chain	MUC4	1	37	37	0.000758543	0.156222299	0.291310352
Q99519	Sialidase-1	NEU1	1	11	11	0.048114346	0.413677833	0.618781335
Q99538	Legumain	LGMN	1	6	6	0.003320032	0.190510668	0.491169799
Q9BS26	Endoplasmic reticulum resident protein 44	ERP44	1	13	13	0.00248462	0.162354374	1.5252669
Q9H299	SH3 domain-binding glutamic acid-rich-like protein 3	SH3BGRL3	1	2	2	0.006581037	0.249884497	1.82341213

Q9NP55	BPI fold-containing family A member 1	BPIFA1	1	11	11	0.026398061	0.381306818	0.257128053
Q9NTK5	Obg-like ATPase 1	OLA1	1	6	6	0.017049738	0.335368745	1.565132875
Q9NUQ8	ATP-binding cassette sub-family F member 3	ABCF3	1	10	10	0.049345467	0.413677833	1.637955486
Q9P0J0	NADH dehydrogenase [ubiquinone] 1 alpha subcomplex	NDUFA13	1	5	5	0.042497246	0.400278111	1.508101665
Q9UMX0	Ubiquilin-1	UBQLN1	1	10	5	0.040812118	0.39553897	1.701204581
Q9Y6R7	IgGFc-binding protein	FCGBP	1	34	34	0.048755741	0.413677833	0.262098735

T = 72 h, IFNλ3: CoV-2 / mock

Uniprot ID	Protein Name	Gene Names	Number of Proteins	Peptides	Unique Peptides	p-Value	p_{FDR}-Value	Ratio of Means (IFNλ3: CoV-2 / mock)
O00391	Sulfhydryl oxidase 1	QSOX1	1	17	17	0.003496243	0.131048781	0.26728898
O00468	Agrin;Agrin N-terminal 110 kDa subunit;Agrin C-terminal 110 kDa subunit;Agrin C-terminal 90 kDa fragment;Agrin C-	AGRN	1	34	34	0.02374311	0.209876382	0.459290282
O00560	Syntenin-1	SDCBP	1	8	8	0.043883847	0.268137742	0.652539949
O43490	Prominin-1	PROM1	1	27	27	0.002535774	0.114268328	0.120934083
O43795	Unconventional myosin-Ib	MYO1B	1	22	19	0.047737639	0.274318439	0.667414587
O60341	Lysine-specific histone demethylase 1A	KDM1A	1	6	6	0.010807136	0.174121205	1.586422554
O60635	Tetraspanin-1	TSPAN1	1	3	3	0.019413081	0.201895613	0.383322046
O75607	Nucleoplasmin-3	NPM3	1	5	5	0.029743069	0.230588737	1.606131099
O75947	ATP synthase subunit d, mitochondrial	ATP5H	1	7	7	0.042336921	0.266117253	0.652025612
O75964	ATP synthase subunit g, mitochondrial	ATP5L	1	3	3	0.008005651	0.164010485	0.556408153
O95436	Sodium-dependent phosphate transport protein 2B	SLC34A2	1	12	12	0.02401449	0.209876382	0.433844785
O95486	Protein transport protein Sec24A	SEC24A	1	7	7	0.000127383	0.045921446	1.583070072
P00450;CON__ENSEMBL:ENSBTAP00000031900	Ceruloplasmin	CP	2	31	31	0.014350894	0.192801138	0.344853319
P00751	Complement factor B;Complement factor B Ba	CFB	1	40	40	0.001089766	0.108375899	0.190663991
P01024;CON__Q2UVX4	Complement C3;Complement C3 beta chain;C3-beta-c;Complement C3 alpha chain;C3a anaphylatoxin;Acylation stimulating protein;Complement C3b alpha chain;Complement C3c alpha chain fragment	C3	2	100	100	0.000211386	0.061268307	0.196671547
P01033	Metalloproteinase inhibitor 1	TIMP1	1	7	7	0.019449607	0.201895613	0.355146404
P01034	Cystatin-C	CST3	1	4	4	0.001155171	0.108375899	0.323796686
P01833	Polymeric immunoglobulin receptor;Secretory component	PIGR	1	42	42	0.001073589	0.108375899	0.194373387
P03973	Antileukoproteinase	SLPI	1	5	5	0.020471745	0.20194832	0.188706567
P06396;REV__Q6TDU7	Gelsolin	GSN	2	42	20	0.001119494	0.108375899	0.616343013
P06731;P31997;P40198	Carcinoembryonic antigen-related cell adhesion molecule	CEACAM5	3	13	11	0.049789408	0.276516168	0.645827888
P08195	4F2 cell-surface antigen heavy chain	SLC3A2	1	5	5	0.038826302	0.25604052	1.878135544
P08571	Monocyte differentiation antigen CD14;Monocyte differentiation antigen CD14, urinary form;Monocyte	CD14	1	7	7	0.019062908	0.201895613	0.297424804
P08603;Q03591;Q02985	Complement factor H	CFH	3	23	23	0.009184631	0.165552967	0.223756399
P09668	Pro-cathepsin H;Cathepsin H mini chain;Cathepsin	CTSH	1	11	11	0.0019823	0.108375899	0.629499588
P12830	Cadherin-1;E-Cad/CTF1;E-Cad/CTF2;E-Cad/CTF3	CDH1	1	11	11	0.005291985	0.144893198	1.513129633
P13987	CD59 glycoprotein	CD59	1	4	4	0.033209063	0.238641871	0.448699071
P15328	Folate receptor alpha	FOLR1	1	7	7	0.004081243	0.131935503	0.393525129
P15941	Mucin-1;Mucin-1 subunit alpha;Mucin-1 subunit beta	MUC1	1	9	9	1.68405E-05	0.036039453	0.32964792
P18827	Syndecan-1	SDC1	1	3	3	0.008966799	0.164010485	1.785104941
P20042	Eukaryotic translation initiation factor 2 subunit 2	EIF2S2	1	6	6	0.032862587	0.238641871	1.56780452
P20061	Transcobalamin-1	TCN1	1	8	8	0.02503345	0.215660912	0.645866756

P25774	Cathepsin S	CTSS	1	14	14	0.003392573	0.131048781	0.617954052
P27105	Erythrocyte band 7 integral membrane protein	STOM	1	15	15	0.015165823	0.196195789	0.542129024
P29034	Protein S100-A2	S100A2	1	4	4	0.031292454	0.23501937	0.577221298
P29279	Connective tissue growth factor	CTGF	1	6	6	0.033007805	0.238641871	0.61261754
P29992	Guanine nucleotide-binding protein subunit alpha-11	GNA11	1	10	4	0.02126105	0.202051327	0.52337909
P31949	Protein S100-A11;Protein S100-A11, N-terminally	S100A11	1	2	2	0.004431872	0.135016054	0.599249233
P35241	Radixin	RDX	1	21	6	0.031968495	0.237621495	0.612473985
P43353;P48448	Aldehyde dehydrogenase family 3 member B1	ALDH3B1	2	15	14	0.008355679	0.164010485	0.555998353
P46060	Ran GTPase-activating protein 1	RANGAP1	1	13	13	0.03768638	0.253942806	1.630038009
P53634	Dipeptidyl peptidase 1;Dipeptidyl peptidase 1 exclusion domain chain;Dipeptidyl peptidase 1 heavy	CTSC	1	19	19	0.005913988	0.154119946	0.662544881
P53999	Activated RNA polymerase II transcriptional coactivator	SUB1	1	5	5	0.005070471	0.142434147	1.502609686
P54707	Potassium-transporting ATPase alpha chain 2	ATP12A	1	31	27	0.00800499	0.164010485	0.590163731
P60903	Protein S100-A10	S100A10	1	5	5	0.005152549	0.142884134	0.485567543
P60953	Cell division control protein 42 homolog	CDC42	1	4	4	0.009023231	0.164010485	0.529200758
P61956;P55854;Q6EEV6	Small ubiquitin-related modifier 2;Small ubiquitin-related modifier 3;Small ubiquitin-related modifier 4	SUMO2;SUMO3;SUMO4	3	2	2	0.007835603	0.164010485	0.565832172
P62191	26S protease regulatory subunit 4	PSMC1	1	14	14	0.001011327	0.108375899	1.624784795
P62805	Histone H4	HIST1H4A	1	13	13	0.017988467	0.201895613	0.611721857
P62910	60S ribosomal protein L32	RPL32	1	6	6	0.018388965	0.201895613	0.55903016
P63000;P60763;P15153	Ras-related C3 botulinum toxin substrate 1;Ras-related C3	RAC1;RAC3	3	6	6	0.02628647	0.220378432	0.5963184
P80188	Neutrophil gelatinase-associated lipocalin	LCN2	1	16	16	0.002052453	0.108375899	0.288985284
Q08380	Galectin-3-binding protein	LGALS3BP	1	25	25	0.000467709	0.101165453	0.498709362
Q08752	Peptidyl-prolyl cis-trans isomerase D	PPID	1	4	4	0.001966495	0.108375899	0.65054818
Q12805	EGF-containing fibulin-like extracellular matrix protein 1	EFEMP1	1	14	14	0.004164366	0.132463578	0.129058934
Q13418	Integrin-linked protein kinase	ILK	1	8	8	0.006728671	0.157917902	1.572616495
Q16270	Insulin-like growth factor-binding protein 7	IGFBP7	1	6	6	0.014320189	0.192801138	0.206925415
Q16719	Kynureninase	KYNU	1	5	5	0.006585825	0.157917902	1.762251766
Q3YEC7	Rab-like protein 6	RABL6	1	11	11	0.002032753	0.108375899	1.559725616
Q53GD3	Choline transporter-like protein 4	SLC44A4	1	5	5	0.001106842	0.108375899	0.288574556
Q5T601	Probable G-protein coupled receptor 110	GPR110	1	4	4	0.015027783	0.196195789	0.523570413
Q6DKJ4	Nucleoredoxin	NXN	1	4	4	0.017025301	0.199605065	1.640254822
Q6IBS0	Twinfilin-2	TWF2	1	8	8	0.024480658	0.211806649	1.543769365
Q6UXY8	Transmembrane channel-like protein 5	TMC5	1	19	19	0.006887589	0.157917902	0.340414283
Q7Z404	Transmembrane channel-like protein 4	TMC4	1	16	16	0.000420282	0.101007784	0.280411655
Q8N5I2	Arrestin domain-containing protein 1	ARRDC1	1	3	3	0.019021784	0.201895613	0.464598766
Q8NFF5	FAD synthase;Molybdenum cofactor biosynthesis protein-	FLAD1	1	8	8	0.037569605	0.253942806	1.701348593
Q8NFJ5	Retinoic acid-induced protein 3	GPRC5A	1	5	5	0.006496463	0.157917902	0.359515972
Q8NFL0	UDP-GlcNAc:betaGal beta-1,3-N-	B3GNT7	1	5	5	0.043466561	0.26690788	0.661168791
Q8TBC5	Zinc finger and SCAN domain-containing protein 18	ZSCAN18	1	4	4	3.44244E-05	0.036039453	1.535610759
Q8TDL5	BPI fold-containing family B member 1	BPIFB1	1	30	30	0.003840384	0.131048781	0.233356316
Q8WXI7	Mucin-16	MUC16	1	39	39	0.000943314	0.108375899	0.049681869
Q92597	Protein NDRG1	NDRG1	1	10	10	0.049664549	0.276516168	1.534222128
Q92973	Transportin-1	TNPO1	1	12	8	0.018567438	0.201895613	0.633168412
Q96DI7	U5 small nuclear ribonucleoprotein 40 kDa protein	SNRNP40	1	10	10	0.01573791	0.197913363	1.598785622

Q96FF7	Uncharacterized protein LOC113230		1	4	4	0.030972062	0.233423591	1.895319273
Q96PP9;Q8N8V2	Guanylate-binding protein 4	GBP4	2	6	4	0.039541312	0.258052466	1.562043128
Q96QR8	Transcriptional activator protein Pur-beta	PURB	1	7	6	0.000226605	0.061268307	1.706249797
Q99102	Mucin-4;Mucin-4 alpha chain;Mucin-4 beta chain	MUC4	1	37	37	0.003145406	0.130921329	0.290813604
Q99828	Calcium and integrin-binding protein 1	CIB1	1	10	10	0.004086768	0.131935503	0.288487297
Q9NP55	BPI fold-containing family A member 1	BPIFA1	1	11	11	0.012876078	0.187628547	0.356669062
Q9UII2	ATPase inhibitor, mitochondrial	ATPIF1	1	5	5	0.01336903	0.188479261	0.170653923
Q9Y2A7	Nck-associated protein 1	NCKAP1	1	12	12	0.047324908	0.274318439	1.582510051
Q9Y4W6	AFG3-like protein 2	AFG3L2	1	5	5	0.008869189	0.164010485	1.540030356

T = 0 h, IFN / Untreated

					Number of Valid Values								
**Uniprot ID**	**Protein Name**	**Gene Names**	**Number of Proteins**	**Unique Peptides**	**Untreated**	**IFNα5**	**IFNα7**	**IFNα16**	**IFNλ3**	**"On"-Group Untreated / IFNα5**	**"On"-Group Untreated / IFNα7**	**"On"-Group Untreated / IFNα16**	**"On"-Group Untreated / IFNλ3**
P09913	Interferon-induced protein with tetratricopeptide	IFIT2	1	21	0	4	4	0	4	IFNα5	IFNα7		IFNλ3
P20592	Interferon-induced GTP-binding protein Mx2	MX2	1	13	0	4	4	0	4	IFNα5	IFNα7		IFNλ3
Q53G44	Interferon-induced protein 44-like	IFI44L	1	7	0	3	3	0	3	IFNα5	IFNα7		IFNλ3
Q8IVU3	Probable E3 ubiquitin-protein ligase HERC6	HERC6	1	2	0	3	4	0	3	IFNα5	IFNα7		IFNλ3
O95786	Probable ATP-dependent RNA helicase DDX58	DDX58	1	11	0	4	3	0	2	IFNα5	IFNα7		
Q10588	ADP-ribosyl cyclase/cyclic ADP-ribose hydrolase 2	BST1	1	3	0	3	1	0	3	IFNα5			IFNλ3
Q15646	2-5-oligoadenylate synthase-like protein	OASL	1	7	0	4	2	0	3	IFNα5			IFNλ3
A6NHR9	Structural maintenance of chromosomes flexible hinge domain-containing protein 1	SMCHD1	1	6	0	3	2	1	2	IFNα5			
Q8TF05	Serine/threonine-protein phosphatase 4 regulatory	PPP4R1	1	4	0	4	1	1	0	IFNα5			
Q9P1F3	Costars family protein ABRACL	ABRACL	1	2	0	3	1	1	1	IFNα5			
Q9UBI1	COMM domain-containing protein 3	COMMD3	1	2	0	3	2	1	2	IFNα5			
Q9Y224	UPF0568 protein C14orf166	C14orf166	1	9	0	3	1	1	1	IFNα5			
O43324	Eukaryotic translation elongation factor 1 epsilon-1	EEF1E1	1	5	3	0	1	0	1	Untreated		Untreated	
A3KMH1	von Willebrand factor A domain-containing protein 8	VWA8	1	5	3	0	4	3	3	Untreated			
P46776	60S ribosomal protein L27a	RPL27A	1	6	3	0	3	1	1	Untreated			
Q8TBF2	Prostamide/prostaglandin F synthase	FAM213B	1	3	3	0	2	2	2	Untreated			
Q92785	Zinc finger protein ubi-d4	DPF2	1	3	3	0	4	4	3	Untreated			
Q99961;Q99962	Endophilin-A2	SH3GL1	2	5	3	0	2	3	4	Untreated			
Q9BXI6	TBC1 domain family member 10A	TBC1D10A	1	3	3	0	2	3	2	Untreated			
P01009	Alpha-1-antitrypsin;Short peptide from AAT	SERPINA1	1	3	0	2	3	3	1		IFNα7	IFNα16	
Q9NW64	Pre-mRNA-splicing factor RBM22	RBM22	1	1	0	2	3	2	4		IFNα7		IFNλ3
O96008	Mitochondrial import receptor subunit TOM40	TOMM40	1	6	0	1	3	1	0		IFNα7		
P50583	Bis(5-nucleosyl)-tetraphosphatase [asymmetrical]	NUDT2	1	5	0	2	3	1	2		IFNα7		
Q14165	Malectin	MLEC	1	3	0	0	3	1	0		IFNα7		
Q68D91	Metallo-beta-lactamase domain-containing protein 2	MBLAC2	1	2	3	1	0	0	1		Untreated	Untreated	
Q92796	Disks large homolog 3	DLG3	1	7	3	3	0	0	2		Untreated	Untreated	
Q9Y3D6	Mitochondrial fission 1 protein	FIS1	1	4	3	1	0	2	0		Untreated		Untreated
Q00688	Peptidyl-prolyl cis-trans isomerase FKBP3	FKBP3	1	7	3	1	0	2	3		Untreated		
Q3B7J2;Q9NXC2	Glucose-fructose oxidoreductase domain-containing protein 2;Glucose-fructose oxidoreductase domain-	GFOD2;GFOD1	2	2	3	2	0	2	1		Untreated		
Q8IYI6	Exocyst complex component 8	EXOC8	1	2	0	1	2	3	0			IFNα16	
Q99569	Plakophilin-4	PKP4	1	7	0	1	1	3	2			IFNα16	
Q9H2P0	Activity-dependent neuroprotector homeobox	ADNP	1	3	0	1	0	3	1			IFNα16	
P18621	60S ribosomal protein L17	RPL17	1	5	3	3	3	0	0			Untreated	Untreated
O94919	Endonuclease domain-containing 1 protein	ENDOD1	1	3	3	1	3	0	1			Untreated	
P00813	Adenosine deaminase	ADA	1	3	3	1	2	0	1			Untreated	
P04233	HLA class II histocompatibility antigen gamma chain	CD74	1	3	3	1	3	0	1			Untreated	
Q15334	Lethal(2) giant larvae protein homolog 1	LLGL1	1	3	3	2	2	0	2			Untreated	
Q9UHY7	Enolase-phosphatase E1	ENOPH1	1	6	4	1	4	0	3			Untreated	
Q8IY21	Probable ATP-dependent RNA helicase DDX60	DDX60	1	5	0	1	1	0	3				IFNλ3
O14929	Histone acetyltransferase type B catalytic subunit	HAT1	1	4	3	3	2	2	0				Untreated
Q14802	FXYD domain-containing ion transport regulator 3	FXYD3	1	1	3	1	1	1	0				Untreated
Q15506	Sperm surface protein Sp17	SPA17	1	6	3	1	3	1	0				Untreated
Q969V3	Nicalin	NCLN	1	8	4	3	3	3	0				Untreated

Q96S44	TP53-regulating kinase	TP53RK	1	1	3	2	2	2	0				Untreated
Q9BSY4	Coiled-coil-helix-coiled-coil-helix domain-containing	CHCHD5	1	5	3	3	3	2	0				Untreated
Q9UHQ9	NADH-cytochrome b5 reductase 1	CYB5R1	1	8	3	1	2	3	0				Untreated
Q9UN37	Vacuolar protein sorting-associated protein 4A	VPS4A	1	5	3	3	2	3	0				Untreated

T = 72h, (mock) IFN / Untreated

Uniprot ID	Protein Name	Gene Names	Number of Proteins	Unique Peptides	Number of Valid Values: Untreated	IFNα5	IFNα7	IFNα16	IFNλ3	"On"-Group Untreated / IFNα5	"On"-Group Untreated / IFNα7	"On"-Group Untreated / IFNα16	"On"-Group Untreated / IFNλ3
Q9BYD1	39S ribosomal protein L13,	MRPL13	1	6	0	3	3	3	1	IFNα5	IFNα7	IFNα16	
O95786	Probable ATP-dependent RNA helicase	DDX58	1	11	0	4	4	0	4	IFNα5	IFNα7		IFNλ3
Q53G44	Interferon-induced protein 44-like	IFI44L	1	7	0	4	4	0	4	IFNα5	IFNα7		IFNλ3
Q8TCB0	Interferon-induced protein 44	IFI44	1	6	0	4	3	0	4	IFNα5	IFNα7		IFNλ3
O75150;Q5VTR2	E3 ubiquitin-protein ligase BRE1B	RNF40	2	3	0	3	1	1	3	IFNα5			IFNλ3
Q15646	2-5-oligoadenylate synthase-like protein	OASL	1	7	0	4	1	0	4	IFNα5			IFNλ3
Q8IY21	Probable ATP-dependent RNA helicase	DDX60	1	5	0	3	2	0	3	IFNα5			IFNλ3
Q9H0J9	Poly [ADP-ribose] polymerase 12	PARP12	1	4	0	4	2	2	4	IFNα5			IFNλ3
Q9Y508	E3 ubiquitin-protein ligase RNF114	RNF114	1	3	0	3	1	0	4	IFNα5			IFNλ3
O00629	Importin subunit alpha-3	KPNA4	1	6	0	3	0	2	2	IFNα5			
Q13325	Interferon-induced protein with	IFIT5	1	2	0	3	2	0	2	IFNα5			
Q8TDB6	E3 ubiquitin-protein ligase DTX3L	DTX3L	1	5	0	3	1	0	2	IFNα5			
P12074	Cytochrome c oxidase subunit 6A1,	COX6A1	1	1	3	0	0	0	1	Untreated	Untreated	Untreated	
P51178	1-phosphatidylinositol 4,5-bisphosphate	PLCD1	1	6	3	0	1	0	1	Untreated		Untreated	
Q9NQC8	Intraflagellar transport protein 46	IFT46	1	2	3	0	1	0	1	Untreated		Untreated	
Q05655	Protein kinase C delta type;Protein kinase C delta type regulatory	PRKCD	1	4	4	0	2	2	0	Untreated			Untreated
Q02880;P11388	DNA topoisomerase 2-beta	TOP2B	2	7	3	0	2	3	2	Untreated			
Q99598	Translin-associated protein X	TSNAX	1	5	3	0	1	3	1	Untreated			
Q86VS8	Protein Hook homolog 3	HOOK3	1	5	0	1	3	3	3		IFNα7	IFNα16	IFNλ3
Q9UID3	Vacuolar protein sorting-associated	VPS51	1	5	0	1	3	3	3		IFNα7	IFNα16	IFNλ3
P78424	POU domain, class 6, transcription factor	POU6F2	1	2	0	0	4	3	1		IFNα7	IFNα16	
Q3SYG4	Protein PTHB1	BBS9	1	5	0	2	3	3	2		IFNα7	IFNα16	
Q86VM9	Zinc finger CCCH domain-containing	ZC3H18	1	5	0	1	3	3	2		IFNα7	IFNα16	
Q9ULX6	A-kinase anchor protein 8-like	AKAP8L	1	3	0	1	3	1	4		IFNα7		IFNλ3
O60449	Lymphocyte antigen 75	LY75	1	2	0	2	3	1	1		IFNα7		
O94811	Tubulin polymerization-promoting	TPPP	1	3	0	1	3	0	1		IFNα7		
P39748	Flap endonuclease 1	FEN1	1	2	0	1	3	2	2		IFNα7		
P46937	Transcriptional coactivator YAP1	YAP1	1	5	0	1	3	0	1		IFNα7		
Q16222	UDP-N-acetylhexosamine pyrophosphorylase;UDP-N-acetylgalactosamine	UAP1	1	3	0	0	3	0	2		IFNα7		
Q16610	Extracellular matrix protein 1	ECM1	1	5	0	1	3	1	1		IFNα7		
Q7LG56;P31350	Ribonucleoside-diphosphate reductase subunit M2 B;Ribonucleoside-	RRM2B;RRM2	2	2	0	1	3	2	1		IFNα7		
Q9H1C7	Cysteine-rich and transmembrane	CYSTM1	1	1	0	1	3	1	0		IFNα7		
Q9UEW8	STE20/SPS1-related proline-alanine-rich	STK39	1	5	0	2	3	1	2		IFNα7		
Q9UI30	Multifunctional methyltransferase	TRMT112	1	3	0	2	3	1	1		IFNα7		
Q9GZY8	Mitochondrial fission factor	MFF	1	5	3	2	0	0	0		Untreated	Untreated	Untreated
P17096	High mobility group protein HMG-	HMGA1	1	3	3	2	0	1	0		Untreated		Untreated

Q07617	Sperm-associated antigen 1	SPAG1	1	3	4	3	0	2	2		Untreated		
Q15653	NF-kappa-B inhibitor beta	NFKBIB	1	5	4	3	0	2	3		Untreated		
Q5TAQ9	DDB1- and CUL4-associated factor 8	DCAF8	1	2	3	2	0	2	4		Untreated		
Q9NXG6	Transmembrane prolyl 4-hydroxylase	P4HTM	1	3	3	1	0	1	3		Untreated		
Q9Y3X0	Coiled-coil domain-containing protein 9	CCDC9	1	6	0	2	2	3	4			IFNα16	IFNλ3
O75531	Barrier-to-autointegration factor;Barrier-to-autointegration factor, N-terminally	BANF1	1	4	0	2	2	3	1			IFNα16	
P28300	Protein-lysine 6-oxidase	LOX	1	2	0	2	0	3	1			IFNα16	
Q8WUA7	TBC1 domain family member 22A	TBC1D22A	1	2	0	0	1	3	1			IFNα16	
Q969E4;Q5H9L2;Q6IPX3	Transcription elongation factor A protein-like 3;Transcription elongation	TCEAL3;TCEAL5	3	5	0	2	1	3	2			IFNα16	
O00471	Exocyst complex component 5	EXOC5	1	3	3	2	2	0	4			Untreated	
P08397	Porphobilinogen deaminase	HMBS	1	4	3	2	1	0	3			Untreated	
P13164;Q01629	Interferon-induced transmembrane protein 1;Interferon-induced	IFITM1;IFITM2	2	1	3	2	1	0	1			Untreated	
P27144	Adenylate kinase 4, mitochondrial	AK4	1	4	4	2	3	0	2			Untreated	
Q7L2H7	Eukaryotic translation initiation factor 3	EIF3M	1	4	4	2	3	0	3			Untreated	
Q96A72	Protein mago nashi homolog 2	MAGOHB	1	1	4	2	4	0	4			Untreated	
Q9BWS9	Chitinase domain-containing protein 1	CHID1	1	4	4	1	2	0	1			Untreated	
Q9NPF4	Probable tRNA N6-adenosine	OSGEP	1	7	3	1	4	0	3			Untreated	
Q2TAC2	Coiled-coil domain-containing protein	CCDC57	1	4	0	0	2	2	3				IFNλ3
Q8IVU3	Probable E3 ubiquitin-protein ligase	HERC6	1	2	0	2	1	0	3				IFNλ3
Q96BJ8	Engulfment and cell motility protein 3	ELMO3	1	4	0	1	0	1	3				IFNλ3
Q9P2R7	Succinyl-CoA ligase [ADP-forming]	SUCLA2	1	3	0	1	2	1	3				IFNλ3
P61619	Protein transport protein Sec61 subunit	SEC61A1	1	1	3	3	1	1	0				Untreated
Q16537	Serine/threonine-protein phosphatase 2A 56 kDa regulatory subunit epsilon	PPP2R5E	1	1	3	2	2	1	0				Untreated
Q16555	Dihydropyrimidinase-related protein 2	DPYSL2	1	5	4	3	3	3	0				Untreated
Q86TU7	Histone-lysine N-methyltransferase	SETD3	1	3	3	2	2	2	0				Untreated
Q9BRX8	Redox-regulatory protein FAM213A	FAM213A	1	5	3	4	2	1	0				Untreated

T = 72h, (CoV-2) IFN / Untreated

					Number of Valid Values								
Uniprot ID	Protein Name	Gene Names	Number of Proteins	Unique Peptides	Untreated	IFNα5	IFNα7	IFNα16	IFNλ3	"On"-Group Untreated / IFNα5	"On"-Group Untreated / IFNα7	"On"-Group Untreated / IFNα16	"On"-Group Untreated / IFNλ3
O14497	AT-rich interactive domain-containing	ARID1A	1	3	0	3	3	3	2	IFNα5	IFNα7	IFNα16	
P20592	Interferon-induced GTP-binding protein Mx2	MX2	1	13	0	4	4	1	4	IFNα5	IFNα7		IFNλ3
P52630	Signal transducer and activator of	STAT2	1	5	0	4	4	1	3	IFNα5	IFNα7		IFNλ3
Q460N5	Poly [ADP-ribose] polymerase 14	PARP14	1	4	0	4	4	2	4	IFNα5	IFNα7		IFNλ3
Q53G44	Interferon-induced protein 44-like	IFI44L	1	7	0	4	4	1	4	IFNα5	IFNα7		IFNλ3
Q8TCB0	Interferon-induced protein 44	IFI44	1	6	0	4	3	0	4	IFNα5	IFNα7		IFNλ3
P06703	Protein S100-A6	S100A6	1	2	0	3	3	2	1	IFNα5	IFNα7		
Q15646	2-5-oligoadenylate synthase-like protein	OASL	1	7	0	4	1	0	4	IFNα5			IFNλ3
Q969E4;Q5H9L2;Q6IPX3	Transcription elongation factor A protein-like 3;Transcription elongation factor A protein-	TCEAL3;TCEAL5	3	5	0	3	2	2	1	IFNα5			
Q9H0A8	COMM domain-containing protein 4	COMMD4	1	2	3	0	0	0	0	untreated	untreated	untreated	untreated
Q16537	Serine/threonine-protein phosphatase 2A 56 kDa regulatory subunit epsilon isoform	PPP2R5E	1	1	3	0	0	2	1	untreated	untreated		
Q9H4K1	RIB43A-like with coiled-coils protein 2	RIBC2	1	6	3	0	0	2	3	untreated	untreated		
Q969E2	Secretory carrier-associated membrane	SCAMP4	1	2	3	0	3	2	0	untreated			untreated
P16070	CD44 antigen	CD44	1	2	4	0	3	2	3	untreated			
P27708	CAD protein;Glutamine-dependent carbamoyl-phosphate synthase;Aspartate	CAD	1	6	3	0	3	2	2	untreated			
P61619	Protein transport protein Sec61 subunit	SEC61A1	1	1	3	0	2	2	3	untreated			
O43823	A-kinase anchor protein 8	AKAP8	1	4	0	0	3	3	4		IFNα7	IFNα16	IFNλ3
Q12824	SWI/SNF-related matrix-associated actin-dependent regulator of chromatin subfamily	SMARCB1	1	4	0	0	3	3	0		IFNα7	IFNα16	
Q86WA8	Lon protease homolog 2, peroxisomal	LONP2	1	2	0	2	3	3	2		IFNα7	IFNα16	
Q96HN2	Putative adenosylhomocysteinase 3	AHCYL2	1	3	0	1	3	3	2		IFNα7	IFNα16	
P21127;Q9UQ88	Cyclin-dependent kinase 11B;Cyclin-	CDK11B;CDK11A	2	7	0	1	3	2	3		IFNα7		IFNλ3
Q5H9R7	Serine/threonine-protein phosphatase 6	PPP6R3	1	4	0	2	3	2	3		IFNα7		IFNλ3
O60264;P28370	SWI/SNF-related matrix-associated actin-dependent regulator of chromatin subfamily A member 5;Probable global transcription	SMARCA5;SMARCA1	2	4	0	2	3	0	2		IFNα7		
O75152;A0A1B0GTU1	Zinc finger CCCH domain-containing protein 11A	ZC3H11A	2	2	0	1	3	1	1		IFNα7		
O75531	Barrier-to-autointegration factor;Barrier-to-autointegration factor, N-terminally	BANF1	1	4	0	1	3	1	2		IFNα7		
P04181	Ornithine aminotransferase, mitochondrial;Ornithine aminotransferase, hepatic form;Ornithine aminotransferase,	OAT	1	5	0	0	4	1	2		IFNα7		
P20794;Q9UPZ9	Serine/threonine-protein kinase MAK;Serine/threonine-protein kinase ICK	MAK;ICK	2	3	0	0	3	2	1		IFNα7		
Q13444	Disintegrin and metalloproteinase domain-	ADAM15	1	3	0	1	3	1	0		IFNα7		
Q92615	La-related protein 4B	LARP4B	1	5	0	2	3	1	2		IFNα7		
Q96JM3	Chromosome alignment-maintaining	CHAMP1	1	2	0	1	3	1	2		IFNα7		
Q9UG63	ATP-binding cassette sub-family F member 2	ABCF2	1	2	0	1	3	2	1		IFNα7		
Q9UNX3	60S ribosomal protein L26-like 1	RPL26L1	1	2	0	2	3	2	1		IFNα7		

Q9Y3B3	Transmembrane emp24 domain-containing	TMED7	1	2	3	1	0	0	0		untreated	untreated	untreated
P17096	High mobility group protein HMG-I/HMG-Y	HMGA1	1	3	3	2	0	1	0		untreated		untreated
O15239	NADH dehydrogenase [ubiquinone] 1 alpha	NDUFA1	1	2	3	3	0	2	2		untreated		
P12074	Cytochrome c oxidase subunit 6A1,	COX6A1	1	1	3	2	0	2	1		untreated		
P49662;Q6UXS9	Caspase-4;Caspase-4 subunit 1;Caspase-4 subunit 2;Inactive caspase-12	CASP4;CASP12	2	2	3	2	0	2	2		untreated		
P53801	Pituitary tumor-transforming gene 1 protein-	PTTG1IP	1	1	4	3	0	1	3		untreated		
Q13907	Isopentenyl-diphosphate Delta-isomerase 1	IDI1	1	4	4	4	0	2	2		untreated		
Q2VPB7	AP-5 complex subunit beta-1	AP5B1	1	3	3	3	0	2	2		untreated		
Q6P9B6	TLD domain-containing protein 1	TLDC1	1	3	3	4	0	3	3		untreated		
Q8NEG2	Uncharacterized protein C7orf57	C7orf57	1	3	3	2	0	2	1		untreated		
Q8WZ42	Titin	TTN	1	4	3	2	0	2	1		untreated		
Q96IX5	Up-regulated during skeletal muscle growth	USMG5	1	2	4	2	0	3	3		untreated		
Q96KG9	N-terminal kinase-like protein	SCYL1	1	2	3	1	0	3	2		untreated		
Q9H0R4	Haloacid dehalogenase-like hydrolase	HDHD2	1	3	4	2	0	2	1		untreated		
Q9NRD5	PRKCA-binding protein	PICK1	1	2	3	1	0	3	3		untreated		
O43464;P83110	Serine protease HTRA2, mitochondrial	HTRA2	2	6	3	3	1	0	0			untreated	untreated
Q96FX7	tRNA (adenine(58)-N(1))-methyltransferase catalytic subunit TRMT61A	TRMT61A	1	3	3	2	2	0	0			untreated	untreated
P50281	Matrix metalloproteinase-14	MMP14	1	3	3	2	2	0	1			untreated	
Q06587	E3 ubiquitin-protein ligase RING1	RING1	1	3	3	3	4	0	2			untreated	
Q16555	Dihydropyrimidinase-related protein 2	DPYSL2	1	5	4	2	4	0	1			untreated	
Q56VL3	OCIA domain-containing protein 2	OCIAD2	1	3	4	3	1	0	1			untreated	
O15212	Prefoldin subunit 6	PFDN6	1	2	0	1	2	1	3				IFNλ3
P62633	Cellular nucleic acid-binding protein	CNBP	1	2	0	1	2	2	3				IFNλ3
Q00059	Transcription factor A, mitochondrial	TFAM	1	2	0	1	0	1	3				IFNλ3
Q96C10	Probable ATP-dependent RNA helicase	DHX58	1	3	0	2	0	0	3				IFNλ3
Q9NQX3	Gephyrin;Molybdopterin adenylyltransferase;Molybdopterin	GPHN	1	4	0	2	0	2	3				IFNλ3
A1A5D9	Bicaudal D-related protein 2	CCDC64B	1	9	3	3	1	2	0				untreated
O60888	Protein CutA	CUTA	1	3	3	3	3	1	0				untreated
Q2TAC6	Kinesin-like protein KIF19	KIF19	1	4	3	1	2	2	0				untreated

T = 72 h, CoV-2 / mock

					Number of Valid Values														
Uniprot ID	Protein Name	Gene Names	Number of Proteins	Unique Peptides	Untreated (CoV-2)	Untreated (mock)	IFNα5 (CoV-2)	IFNα5 (mock)	IFNα7 (CoV-2)	IFNα7 (mock)	IFNα16 (CoV-2)	IFNα16 (mock)	IFNλ3 (CoV-2)	IFNλ3 (mock)	"On"-Group Untreated: CoV-2 /	"On"-Group IFNα5: CoV-2 / mock	"On"-Group IFNα7: CoV-2 / mock	"On"-Group IFNα16: CoV-2 / mock	"On"-Group IFNλ3: CoV-2 / mock
Q96KG9	N-terminal kinase-like protein	SCYL1	1	2	3	0	1	0	0	1	3	0	2	0	CoV-2			CoV-2	
Q3SYG4	Protein PTHB1	BBS9	1	5	3	0	3	2	4	3	2	3	3	2	CoV-2				
Q86VM9	Zinc finger CCCH domain-containing protein	ZC3H18	1	5	3	0	3	1	4	3	3	3	3	2	CoV-2				
Q86VS8	Protein Hook homolog 3	HOOK3	1	5	3	0	3	1	3	3	3	3	3	3	CoV-2				
Q969E2	Secretory carrier-associated membrane	SCAMP4	1	2	3	0	0	1	3	2	2	1	0	0	CoV-2				
Q96EY1	DnaJ homolog subfamily A member 3,	DNAJA3	1	2	3	0	1	0	1	1	2	0	1	1	CoV-2				
Q96FX7	tRNA (adenine(58)-N(1))-methyltransferase catalytic subunit TRMT61A	TRMT61A	1	3	3	0	2	1	2	0	0	0	0	1	CoV-2				
Q9H6T0	Epithelial splicing regulatory protein 2	ESRP2	1	3	3	0	1	1	1	0	2	2	1	1	CoV-2				
P01009	Alpha-1-antitrypsin;Short peptide from AAT	SERPINA1	1	3	0	4	0	4	0	4	0	4	0	2	mock	mock	mock	mock	
O15230	Laminin subunit alpha-5	LAMA5	1	8	0	3	0	3	0	2	2	3	1	2	mock	mock			
Q96EY5	Multivesicular body subunit 12A	MVB12A	1	5	0	4	1	3	0	2	0	3	1	2	mock			mock	
Q9UBX7	Kallikrein-11;Kallikrein-11 inactive chain 1;Kallikrein-11 inactive chain 2	KLK11	1	3	0	4	0	2	0	2	0	3	0	2	mock			mock	
P33991	DNA replication licensing factor MCM4	MCM4	1	3	0	3	0	2	0	1	0	2	0	3	mock				mock
P01011	Alpha-1-antichymotrypsin;Alpha-1-	SERPINA3	1	6	0	3	1	3	1	2	1	2	1	1	mock				
P02751	Fibronectin;Anastellin;Ugl-Y1;Ugl-Y2;Ugl-Y3	FN1	1	13	0	3	0	2	1	2	1	1	1	2	mock				
P08118	Beta-microseminoprotein	MSMB	1	2	0	3	0	2	1	1	0	1	0	2	mock				
Q8NFJ9	Bardet-Biedl syndrome 1 protein	BBS1	1	4	0	3	2	2	2	4	2	4	2	3	mock				
Q16222	UDP-N-acetylhexosamine pyrophosphorylase;UDP-N-acetylgalactosamine pyrophosphorylase;UDP-	UAP1	1	3	1	0	3	0	0	3	3	0	1	2		CoV-2	mock	CoV-2	
P52434	DNA-directed RNA polymerases I, II, and III	POLR2H	1	4	2	2	3	0	0	2	1	1	3	0		CoV-2			CoV-2
O60888	Protein CutA	CUTA	1	3	3	2	3	0	3	2	1	2	0	2		CoV-2			
Q15650	Activating signal cointegrator 1	TRIP4	1	4	2	2	3	0	3	2	2	2	2	1		CoV-2			
Q16740	ATP-dependent Clp protease proteolytic	CLPP	1	1	4	2	3	0	3	3	2	1	3	1		CoV-2			
Q96PU5;P46934	E3 ubiquitin-protein ligase NEDD4-like	NEDD4L	2	9	3	2	3	0	2	1	2	0	3	3		CoV-2			
Q99567	Nuclear pore complex protein Nup88	NUP88	1	5	2	0	3	0	1	0	1	1	1	0		CoV-2			
Q9BRG2	SH2 domain-containing protein 3A	SH2D3A	1	9	2	0	3	0	2	1	0	1	1	0		CoV-2			
Q9BU89	Deoxyhypusine hydroxylase	DOHH	1	6	2	2	3	0	0	2	2	0	1	2		CoV-2			
Q9NPC4	Lactosylceramide 4-alpha-	A4GALT	1	5	1	3	0	3	0	3	1	4	4	2		mock	mock		
P05186	Alkaline phosphatase, tissue-nonspecific	ALPL	1	4	0	1	0	3	0	2	0	3	1	3		mock		mock	
P61619	Protein transport protein Sec61 subunit alpha	SEC61A1	1	1	3	3	0	3	2	1	2	1	3	0		mock			CoV-2
Q10472;Q8IUC8	Polypeptide N-acetylgalactosaminyltransferase 1;Polypeptide N-	GALNT1;GALNT13	2	3	0	2	0	3	0	0	0	1	0	3		mock			mock
A6NHR9	Structural maintenance of chromosomes flexible hinge domain-containing protein 1	SMCHD1	1	6	2	2	0	3	2	0	0	0	2	1		mock			
O43823	A-kinase anchor protein 8	AKAP8	1	4	0	1	0	3	3	1	3	2	4	3		mock			
O60240	Perilipin-1	PLIN1	1	2	2	2	0	3	1	1	2	4	2	2		mock			
P48378;P22670	DNA-binding protein RFX2	RFX2	2	3	1	1	0	3	1	0	0	0	1	0		mock			
Q14696	LDLR chaperone MESD	MESDC2	1	3	0	2	0	3	1	2	2	2	1	3		mock			
Q5VW32	BRO1 domain-containing protein BROX	BROX	1	3	1	3	0	4	2	4	1	2	1	2		mock			
Q9UBI6	Guanine nucleotide-binding protein G(I)/G(S)/G(O) subunit gamma-12	GNG12	1	3	1	2	0	3	4	2	2	3	4	3		mock			
O75152;A0A1B0GTU1	Zinc finger CCCH domain-containing protein 11A	ZC3H11A	2	2	0	0	1	1	3	0	1	1	1	1			CoV-2		
P21127;Q9UQ88	Cyclin-dependent kinase 11B;Cyclin-	CDK11B;CDK11	2	7	0	0	1	1	3	0	2	2	3	2			CoV-2		
P49903	Selenide, water dikinase 1	SEPHS1	1	6	1	0	2	1	3	0	3	1	2	1			CoV-2		
Q9UG63	ATP-binding cassette sub-family F member 2	ABCF2	1	2	0	0	1	0	3	0	2	1	1	0			CoV-2		
Q687X5	Metalloreductase STEAP4	STEAP4	1	5	1	4	1	3	0	3	0	4	0	3			mock	mock	mock
P07902	Galactose-1-phosphate uridylyltransferase	GALT	1	2	1	1	0	1	0	3	1	1	1	2			mock		
P43234	Cathepsin O	CTSO	1	4	1	4	1	4	0	4	1	4	0	2			mock		
Q96IX5	Up-regulated during skeletal muscle growth	USMG5	1	2	4	2	2	2	0	3	3	2	3	3			mock		

Q9H1C7	Cysteine-rich and transmembrane domain-	CYSTM1	1	1	1	0	0	1	0	3	0	1	0	0			mock		
Q9H4K1	RIB43A-like with coiled-coils protein 2	RIBC2	1	6	3	3	0	2	0	4	2	1	3	2			mock		
O00471	Exocyst complex component 5	EXOC5	1	3	3	3	4	2	1	2	3	0	2	4				CoV-2	
P17936	Insulin-like growth factor-binding protein 3	IGFBP3	1	4	3	1	3	1	2	1	3	0	2	1				CoV-2	
P27144	Adenylate kinase 4, mitochondrial	AK4	1	4	2	4	3	2	3	3	3	0	3	2				CoV-2	
P33527	Multidrug resistance-associated protein 1	ABCC1	1	3	3	2	2	0	2	1	3	0	1	0				CoV-2	
P51178	1-phosphatidylinositol 4,5-bisphosphate	PLCD1	1	6	3	3	1	0	1	1	3	0	1	1				CoV-2	
P82921	28S ribosomal protein S21, mitochondrial	MRPS21	1	3	2	2	2	2	0	1	3	0	1	1				CoV-2	
Q15031	Probable leucine--tRNA ligase, mitochondrial	LARS2	1	5	3	1	1	1	1	0	3	0	1	1				CoV-2	
Q5T653	39S ribosomal protein L2, mitochondrial	MRPL2	1	3	3	2	2	3	2	1	3	0	1	3				CoV-2	
Q96A72	Protein mago nashi homolog 2	MAGOHB	1	1	2	4	4	2	3	4	3	0	2	4				CoV-2	
Q96HN2	Putative adenosylhomocysteinase 3	AHCYL2	1	3	0	1	1	0	3	1	3	0	2	1				CoV-2	
Q96P16	Regulation of nuclear pre-mRNA domain-	RPRD1A	1	4	3	1	2	2	2	2	3	0	2	2				CoV-2	
Q9BPW9	Dehydrogenase/reductase SDR family	DHRS9	1	4	2	2	3	2	3	2	3	0	2	2				CoV-2	
Q9NPF4	Probable tRNA N6-adenosine	OSGEP	1	7	2	3	2	1	4	4	4	0	3	3				CoV-2	
Q9NRD5	PRKCA-binding protein	PICK1	1	2	3	1	1	2	0	1	3	0	3	1				CoV-2	
Q9UBP6	tRNA (guanine-N(7)-)-methyltransferase	METTL1	1	3	1	1	2	2	0	0	4	0	1	0				CoV-2	
Q9UHD2	Serine/threonine-protein kinase TBK1	TBK1	1	3	2	0	0	2	2	2	3	0	2	1				CoV-2	
Q9UPM9	B9 domain-containing protein 1	B9D1	1	2	1	1	0	0	1	1	3	0	2	0				CoV-2	
Q9Y3B7	39S ribosomal protein L11, mitochondrial	MRPL11	1	4	4	2	2	1	2	0	3	0	2	1				CoV-2	
O43447	Peptidyl-prolyl cis-trans isomerase H	PPIH	1	4	0	1	0	1	1	2	0	3	0	1				mock	
P19021	Peptidyl-glycine alpha-amidating monooxygenase;Peptidylglycine alpha-hydroxylating monooxygenase;Peptidyl-alpha-	PAM	1	4	1	2	0	2	0	1	0	3	0	0				mock	
P26583	High mobility group protein B2	HMGB2	1	2	2	3	1	1	2	3	0	4	3	2				mock	
P28300	Protein-lysine 6-oxidase	LOX	1	2	0	0	0	2	0	0	0	3	0	1				mock	
P78424	POU domain, class 6, transcription factor 2	POU6F2	1	2	0	0	0	0	1	4	0	3	1	1				mock	
Q06587	E3 ubiquitin-protein ligase RING1	RING1	1	3	3	2	3	3	4	3	0	3	2	4				mock	
Q16555	Dihydropyrimidinase-related protein 2	DPYSL2	1	5	4	4	2	3	4	3	0	3	1	0				mock	
Q56VL3	OCIA domain-containing protein 2	OCIAD2	1	3	4	4	3	3	1	2	0	4	1	1				mock	
Q9BUR4	Telomerase Cajal body protein 1	WRAP53	1	3	0	1	0	1	0	1	0	3	1	1				mock	
Q9P2H3	Intraflagellar transport protein 80 homolog	IFT80	1	6	2	2	1	1	1	1	0	3	1	3				mock	
Q9Y6D6;Q9Y6D5	Brefeldin A-inhibited guanine nucleotide-exchange protein 1;Brefeldin A-inhibited	ARFGEF1;ARFGEF2	2	4	0	1	1	0	2	0	0	3	0	2				mock	
P35613	Basigin	BSG	1	1	1	1	1	0	2	0	2	1	3	0					CoV-2
Q13868	Exosome complex component RRP4	EXOSC2	1	3	2	1	1	0	0	1	1	1	3	0					CoV-2
Q86TU7	Histone-lysine N-methyltransferase setd3	SETD3	1	3	4	3	3	2	2	2	3	2	3	0					CoV-2
Q96A35	39S ribosomal protein L24, mitochondrial	MRPL24	1	3	1	1	1	0	2	1	1	1	3	0					CoV-2
Q9BRX8	Redox-regulatory protein FAM213A	FAM213A	1	5	4	3	2	4	2	2	1	1	3	0					CoV-2
Q9GZY8	Mitochondrial fission factor	MFF	1	5	2	3	3	2	1	0	2	0	4	0					CoV-2
O75882	Attractin	ATRN	1	8	1	4	1	4	1	4	2	4	0	4					mock
P18065	Insulin-like growth factor-binding protein 2	IGFBP2	1	6	1	2	1	2	2	2	1	3	0	3					mock
Q14508	WAP four-disulfide core domain protein 2	WFDC2	1	2	1	1	1	1	0	2	1	3	0	3					mock
Q96DA0	Zymogen granule protein 16 homolog B	ZG16B	1	3	1	2	1	2	0	2	1	2	0	3					mock
Q9UID3	Vacuolar protein sorting-associated protein 51	VPS51	1	5	2	0	0	1	2	3	1	3	0	3					mock
Q9ULX6	A-kinase anchor protein 8-like	AKAP8L	1	3	2	0	1	1	2	3	2	1	0	4					mock

Spplementary Table 3_STRING Enriched GO-Biological Processes

Table No.	Content
1	T = 0 h, IFNα5 / Untreated
2	T = 0 h, IFNα7 / Untreated
3	T = 0 h, IFNα16 / Untreated
4	T = 0 h, IFNλ3 / Untreated
5	T = 72 h (CoV-2), IFNα5 / Untreated
6	T = 72 h (CoV-2), IFNα7 / Untreated
7	T = 72 h (CoV-2), IFNα16 / Untreated
8	T = 72 h (CoV-2), IFNλ3 / Untreated
9	T = 72 h (mock) IFNα5 / Untreated
10	T = 72 h (mock) IFNα7 / Untreated
11	T = 72 h (mock) IFNα16 / Untreated
12	T = 72 h (mock) IFNλ3 / Untreated
13	T = 72 h, Untreated: CoV-2 / mock
14	T = 72 h, IFNα5: CoV-2 / mock
15	T = 72 h, IFNα7: CoV-2 / mock
16	T = 72 h, IFNα16: CoV-2 / mock
17	T = 72 h, IFNλ3: CoV-2 / mock

T = 0 h, IFNα5 / Untreated

#Term ID	Term Description	Observed Gene Count	Background Gene Count	Strength	False Discovery Rate	Matching Proteins in Your Input (IDs)	Matching Proteins in Your Input (Labels)
GO:0060337	type I interferon signaling pathway	10	65	1.8	3.06E-12	9606.ENSP00000257570,9606.ENSP00000306565,9606.ENSP00000333657,9606.ENSP00000343023,9606.ENSP00000354394,9606.ENSP00000360891,9606.ENSP00000368699,9606.ENSP00000381601,9606.ENSP00000395590,9606.ENSP00000399168	OASL,ISG20,MX2,SP100,STAT1,IFIT2,ISG15,MX1,IFI35,HLA-B
GO:0051607	defense response to virus	11	181	1.39	3.58E-10	9606.ENSP00000242351,9606.ENSP00000257570,9606.ENSP00000268058,9606.ENSP00000306565,9606.ENSP00000333657,9606.ENSP00000354394,9606.ENSP00000359787,9606.ENSP00000360891,9606.ENSP00000368699,9606.ENSP00000369213,9606.ENSP00000381601	ZC3HAV1,OASL,PML,ISG20,MX2,STAT1,IFI44L,IFIT2,ISG15,DDX58,MX1
GO:0048525	negative regulation of viral process	9	93	1.6	8.06E-10	9606.ENSP00000242351,9606.ENSP00000257570,9606.ENSP00000268058,9606.ENSP00000306565,9606.ENSP00000325618,9606.ENSP00000343023,9606.ENSP00000354394,9606.ENSP00000368699,9606.ENSP00000381601	ZC3HAV1,OASL,PML,ISG20,PARP10,SP100,STAT1,ISG15,MX1
GO:0045087	innate immune response	15	676	0.96	1.25E-08	9606.ENSP00000242351,9606.ENSP00000257570,9606.ENSP00000268058,9606.ENSP00000306565,9606.ENSP00000333657,9606.ENSP00000343023,9606.ENSP00000354394,9606.ENSP00000359490,9606.ENSP00000360891,9606.ENSP00000368699,9606.ENSP00000369213,9606.ENSP00000381601,9606.ENSP00000395590,9606.ENSP00000399168,9606.ENSP00000409279	ZC3HAV1,OASL,PML,ISG20,MX2,SP100,STAT1,GBP4,IFIT2,ISG15,DDX58,MX1,IFI35,HLA-B,OTUD4
GO:0043901	negative regulation of multi-organism process	10	212	1.28	2.32E-08	9606.ENSP00000242351,9606.ENSP00000257570,9606.ENSP00000268058,9606.ENSP00000306565,9606.ENSP00000325618,9606.ENSP00000343023,9606.ENSP00000354394,9606.ENSP00000368699,9606.ENSP00000381601,9606.ENSP00000399168	ZC3HAV1,OASL,PML,ISG20,PARP10,SP100,STAT1,ISG15,MX1,HLA-B
GO:0098542	defense response to other organism	16	859	0.88	2.32E-08	9606.ENSP00000242351,9606.ENSP00000257570,9606.ENSP00000268058,9606.ENSP00000306565,9606.ENSP00000333657,9606.ENSP00000343023,9606.ENSP00000354394,9606.ENSP00000359490,9606.ENSP00000359787,9606.ENSP00000360891,9606.ENSP00000368699,9606.ENSP00000369213,9606.ENSP00000381601,9606.ENSP00000395590,9606.ENSP00000399168,9606.ENSP00000409279	ZC3HAV1,OASL,PML,ISG20,MX2,SP100,STAT1,GBP4,IFI44L,IFIT2,ISG15,DDX58,MX1,IFI35,HLA-B,OTUD4
GO:0006952	defense response	18	1234	0.77	4.69E-08	9606.ENSP00000242351,9606.ENSP00000249066,9606.ENSP00000257570,9606.ENSP00000268058,9606.ENSP00000306565,9606.ENSP00000333657,9606.ENSP00000343023,9606.ENSP00000354394,9606.ENSP00000359490,9606.ENSP00000359787,9606.ENSP00000360891,9606.ENSP00000368699,9606.ENSP00000369213,9606.ENSP00000376793,9606.ENSP00000381601,9606.ENSP00000395590,9606.ENSP00000399168,9606.ENSP00000409279	ZC3HAV1,APOL2,OASL,PML,ISG20,MX2,SP100,STAT1,GBP4,IFI44L,IFIT2,ISG15,DDX58,GIG25,MX1,IFI35,HLA-B,OTUD4

GO:0051707	response to other organism	17	1173	0.77	1.57E-07	9606.ENSP00000242351,9606.ENSP00000257570,9606.ENSP00000264346,9606.ENSP00000268058,9606.ENSP00000306565,9606.ENSP00000333657,9606.ENSP00000343023,9606.ENSP00000354394,9606.ENSP00000359490,9606.ENSP00000359787,9606.ENSP00000360891,9606.ENSP00000368699,9606.ENSP00000369213,9606.ENSP00000381601,9606.ENSP00000395590,9606.ENSP00000399168,9606.ENSP00000409279	ZC3HAV1,OASL,HERC6,PML,ISG20,MX2,SP100,STAT1,GBP4,IFI44L,IFIT2,ISG15,DDX58,MX1,IFI35,HLA-B,OTUD4
GO:1903901	negative regulation of viral life cycle	7	77	1.57	1.57E-07	9606.ENSP00000242351,9606.ENSP00000257570,9606.ENSP00000268058,9606.ENSP00000306565,9606.ENSP00000325618,9606.ENSP00000368699,9606.ENSP00000381601	ZC3HAV1,OASL,PML,ISG20,PARP10,ISG15,MX1
GO:0002252	immune effector process	15	927	0.82	3.17E-07	9606.ENSP00000242351,9606.ENSP00000257570,9606.ENSP00000265016,9606.ENSP00000268058,9606.ENSP00000306565,9606.ENSP00000333657,9606.ENSP00000354394,9606.ENSP00000359787,9606.ENSP00000360891,9606.ENSP00000366032,9606.ENSP00000368699,9606.ENSP00000369213,9606.ENSP00000376793,9606.ENSP00000381601,9606.ENSP00000399168	ZC3HAV1,OASL,BST1,PML,ISG20,MX2,STAT1,IFI44L,IFIT2,COMMD3,ISG15,DDX58,GIG25,MX1,HLA-B
GO:0045071	negative regulation of viral genome replication	6	52	1.67	4.65E-07	9606.ENSP00000242351,9606.ENSP00000257570,9606.ENSP00000306565,9606.ENSP00000325618,9606.ENSP00000368699,9606.ENSP00000381601	ZC3HAV1,OASL,ISG20,PARP10,ISG15,MX1
GO:0006955	immune response	18	1560	0.67	9.85E-07	9606.ENSP00000242351,9606.ENSP00000257570,9606.ENSP00000265016,9606.ENSP00000268058,9606.ENSP00000306565,9606.ENSP00000333657,9606.ENSP00000343023,9606.ENSP00000354394,9606.ENSP00000359490,9606.ENSP00000360891,9606.ENSP00000366032,9606.ENSP00000368699,9606.ENSP00000369213,9606.ENSP00000376793,9606.ENSP00000381601,9606.ENSP00000395590,9606.ENSP00000399168,9606.ENSP00000409279	ZC3HAV1,OASL,BST1,PML,ISG20,MX2,SP100,STAT1,GBP4,IFIT2,COMMD3,ISG15,DDX58,GIG25,MX1,IFI35,HLA-B,OTUD4
GO:0019221	cytokine-mediated signaling pathway	12	655	0.87	3.48E-06	9606.ENSP00000257570,9606.ENSP00000268058,9606.ENSP00000306565,9606.ENSP00000333657,9606.ENSP00000343023,9606.ENSP00000354394,9606.ENSP00000360891,9606.ENSP00000368699,9606.ENSP00000381601,9606.ENSP00000395590,9606.ENSP00000399168,9606.ENSP00000446252	OASL,PML,ISG20,MX2,SP100,STAT1,IFIT2,ISG15,MX1,IFI35,HLA-B,SOD2
GO:0043900	regulation of multi-organism process	12	653	0.87	3.48E-06	9606.ENSP00000242351,9606.ENSP00000257570,9606.ENSP00000268058,9606.ENSP00000306565,9606.ENSP00000325618,9606.ENSP00000343023,9606.ENSP00000354394,9606.ENSP00000368699,9606.ENSP00000369213,9606.ENSP00000381601,9606.ENSP00000399168,9606.ENSP00000409279	ZC3HAV1,OASL,PML,ISG20,PARP10,SP100,STAT1,ISG15,DDX58,MX1,HLA-B,OTUD4
GO:0002376	immune system process	20	2370	0.54	1.63E-05	9606.ENSP00000242351,9606.ENSP00000257570,9606.ENSP00000264346,9606.ENSP00000265016,9606.ENSP00000268058,9606.ENSP00000306565,9606.ENSP00000333657,9606.ENSP00000343023,9606.ENSP00000354394,9606.ENSP00000359490,9606.ENSP00000359787,9606.ENSP00000360891,9606.ENSP00000366032,9606.ENSP00000368699,9606.ENSP00000369213,9606.ENSP00000376793,9606.ENSP00000381601,9606.ENSP00000395590,9606.ENSP00000399168,9606.ENSP00000409279	ZC3HAV1,OASL,HERC6,BST1,PML,ISG20,MX2,SP100,STAT1,GBP4,IFI44L,IFIT2,COMMD3,ISG15,DDX58,GIG25,MX1,IFI35,HLA-B,OTUD4

GO:0071345	cellular response to cytokine stimulus	13	953	0.75	2.10E-05	9606.ENSP00000257570,9606.ENSP00000268058,9606.ENSP00000306565,9606.ENSP00000333657,9606.ENSP00000343023,9606.ENSP00000354394,9606.ENSP00000359490,9606.ENSP00000360891,9606.ENSP00000368699,9606.ENSP00000381601,9606.ENSP00000395590,9606.ENSP00000399168,9606.ENSP00000446252	OASL,PML,ISG20,MX2,SP100,STAT1,GBP4,IFIT2,ISG15,MX1,IFI35,HLA-B,SOD2
GO:0060333	interferon-gamma-mediated signaling pathway	5	69	1.47	5.46E-05	9606.ENSP00000257570,9606.ENSP00000268058,9606.ENSP00000343023,9606.ENSP00000354394,9606.ENSP00000399168	OASL,PML,SP100,STAT1,HLA-B
GO:0009605	response to external stimulus	18	2152	0.53	8.33E-05	9606.ENSP00000242351,9606.ENSP00000257570,9606.ENSP00000264346,9606.ENSP00000268058,9606.ENSP00000306565,9606.ENSP00000333657,9606.ENSP00000343023,9606.ENSP00000354394,9606.ENSP00000359490,9606.ENSP00000359787,9606.ENSP00000360891,9606.ENSP00000368699,9606.ENSP00000369213,9606.ENSP00000379038,9606.ENSP00000381601,9606.ENSP00000395590,9606.ENSP00000399168,9606.ENSP00000409279	ZC3HAV1,OASL,HERC6,PML,ISG20,MX2,SP100,STAT1,GBP4,IFI44L,IFIT2,ISG15,DDX58,TYMP,MX1,IFI35,HLA-B,OTUD4
GO:0071346	cellular response to interferon-gamma	6	155	1.2	0.00013	9606.ENSP00000257570,9606.ENSP00000268058,9606.ENSP00000343023,9606.ENSP00000354394,9606.ENSP00000359490,9606.ENSP00000399168	OASL,PML,SP100,STAT1,GBP4,HLA-B
GO:0051704	multi-organism process	19	2514	0.49	0.00016	9606.ENSP00000242351,9606.ENSP00000249066,9606.ENSP00000257570,9606.ENSP00000261700,9606.ENSP00000264346,9606.ENSP00000268058,9606.ENSP00000306565,9606.ENSP00000333657,9606.ENSP00000343023,9606.ENSP00000354394,9606.ENSP00000359490,9606.ENSP00000359787,9606.ENSP00000360891,9606.ENSP00000368699,9606.ENSP00000369213,9606.ENSP00000381601,9606.ENSP00000395590,9606.ENSP00000399168,9606.ENSP00000409279	ZC3HAV1,APOL2,OASL,C14orf166,HERC6,PML,ISG20,MX2,SP100,STAT1,GBP4,IFI44L,IFIT2,ISG15,DDX58,MX1,IFI35,HLA-B,OTUD4
GO:0032727	positive regulation of interferon-alpha production	3	23	1.73	0.0015	9606.ENSP00000242351,9606.ENSP00000354394,9606.ENSP00000369213	ZC3HAV1,STAT1,DDX58
GO:0050688	regulation of defense response to virus	4	71	1.36	0.0015	9606.ENSP00000242351,9606.ENSP00000268058,9606.ENSP00000354394,9606.ENSP00000369213	ZC3HAV1,PML,STAT1,DDX58
GO:0006950	response to stress	20	3267	0.4	0.0017	9606.ENSP00000242351,9606.ENSP00000249066,9606.ENSP00000257570,9606.ENSP00000268058,9606.ENSP00000306565,9606.ENSP00000326603,9606.ENSP00000333657,9606.ENSP00000343023,9606.ENSP00000354394,9606.ENSP00000359490,9606.ENSP00000359787,9606.ENSP00000360891,9606.ENSP00000368699,9606.ENSP00000369213,9606.ENSP00000376793,9606.ENSP00000381601,9606.ENSP00000395590,9606.ENSP00000399168,9606.ENSP00000409279,9606.ENSP00000446252	ZC3HAV1,APOL2,OASL,PML,ISG20,SMCHD1,MX2,SP100,STAT1,GBP4,IFI44L,IFIT2,ISG15,DDX58,GIG25,MX1,IFI35,HLA-B,OTUD4,SOD2
GO:0002230	positive regulation of defense response to virus by host	3	26	1.67	0.002	9606.ENSP00000268058,9606.ENSP00000354394,9606.ENSP00000369213	PML,STAT1,DDX58
GO:0032479	regulation of type I interferon production	4	112	1.16	0.0071	9606.ENSP00000242351,9606.ENSP00000354394,9606.ENSP00000368699,9606.ENSP00000369213	ZC3HAV1,STAT1,ISG15,DDX58
GO:0003374	dynamin family protein polymerization involved in mitochondrial fission	2	7	2.07	0.0078	9606.ENSP00000333657,9606.ENSP00000381601	MX2,MX1

GO:0016032	viral process	7	571	0.7	0.0172	9606.ENSP00000261700,9606.ENSP00000268058,9606.ENSP00000343023,9606.ENSP00000354394,9606.ENSP00000368699,9606.ENSP00000369213,9606.ENSP00000399168	C14orf166,PML,SP100,STAT1,ISG15,DDX58,HLA-B
GO:0002831	regulation of response to biotic stimulus	6	426	0.76	0.021	9606.ENSP00000242351,9606.ENSP00000268058,9606.ENSP00000354394,9606.ENSP00000369213,9606.ENSP00000399168,9606.ENSP00000409279	ZC3HAV1,PML,STAT1,DDX58,HLA-B,OTUD4
GO:0071310	cellular response to organic substance	14	2219	0.41	0.0217	9606.ENSP00000257570,9606.ENSP00000268058,9606.ENSP00000306565,9606.ENSP00000333657,9606.ENSP00000343023,9606.ENSP00000354394,9606.ENSP00000359490,9606.ENSP00000360891,9606.ENSP00000368699,9606.ENSP00000369213,9606.ENSP00000381601,9606.ENSP00000395590,9606.ENSP00000399168,9606.ENSP00000446252	OASL,PML,ISG20,MX2,SP100,STAT1,GBP4,IFIT2,ISG15,DDX58,MX1,IFI35,HLA-B,SOD2
GO:1901342	regulation of vasculature development	5	305	0.82	0.0298	9606.ENSP00000268058,9606.ENSP00000343023,9606.ENSP00000347495,9606.ENSP00000354394,9606.ENSP00000446252	PML,SP100,WARS,STAT1,SOD2
GO:0048384	retinoic acid receptor signaling pathway	2	17	1.68	0.0304	9606.ENSP00000268058,9606.ENSP00000343023	PML,SP100
GO:0080134	regulation of response to stress	10	1299	0.5	0.0321	9606.ENSP00000242351,9606.ENSP00000265016,9606.ENSP00000268058,9606.ENSP00000354394,9606.ENSP00000367408,9606.ENSP00000369038,9606.ENSP00000369213,9606.ENSP00000399168,9606.ENSP00000409279,9606.ENSP00000446252	ZC3HAV1,BST1,PML,STAT1,CASK,EEF1E1,DDX58,HLA-B,OTUD4,SOD2
GO:0000266	mitochondrial fission	2	19	1.63	0.035	9606.ENSP00000333657,9606.ENSP00000381601	MX2,MX1
GO:0008285	negative regulation of cell population proliferation	7	669	0.63	0.035	9606.ENSP00000268058,9606.ENSP00000325618,9606.ENSP00000347495,9606.ENSP00000354394,9606.ENSP00000367408,9606.ENSP00000369038,9606.ENSP00000446252	PML,PARP10,WARS,STAT1,CASK,EEF1E1,SOD2
GO:0009062	fatty acid catabolic process	3	84	1.16	0.0356	9606.ENSP00000360541,9606.ENSP00000360569,9606.ENSP00000361151	CPT2,SCP2,CEL
GO:0031347	regulation of defense response	7	676	0.63	0.0356	9606.ENSP00000242351,9606.ENSP00000265016,9606.ENSP00000268058,9606.ENSP00000354394,9606.ENSP00000369213,9606.ENSP00000399168,9606.ENSP00000409279	ZC3HAV1,BST1,PML,STAT1,DDX58,HLA-B,OTUD4
GO:0050896	response to stimulus	30	7824	0.19	0.0371	9606.ENSP00000194214,9606.ENSP00000242351,9606.ENSP00000249066,9606.ENSP00000257570,9606.ENSP00000264346,9606.ENSP00000265016,9606.ENSP00000268058,9606.ENSP00000269886,9606.ENSP00000290291,9606.ENSP00000306565,9606.ENSP00000326603,9606.ENSP00000333657,9606.ENSP00000343023,9606.ENSP00000354394,9606.ENSP00000359490,9606.ENSP00000359787,9606.ENSP00000360891,9606.ENSP00000366032,9606.ENSP00000368699,9606.ENSP00000369213,9606.ENSP00000376793,9606.ENSP00000379038,9606.ENSP00000381601,9606.ENSP00000383402,9606.ENSP00000395590,9606.ENSP00000399168,9606.ENSP00000409279,9606.ENSP00000413218,9606.ENSP00000436901,9606.ENSP00000446252	HSPB11,ZC3HAV1,APOL2,OASL,HERC6,BST1,PML,SH3GL1,OGFR,ISG20,SMCHD1,MX2,SP100,STAT1,GBP4,IFI44L,IFIT2,COMMD3,ISG15,DDX58,GIG25,TYMP,MX1,PPP4R1,IFI35,HLA-B,OTUD4,FAM213B,DPF2,SOD2

GO:0070887	cellular response to chemical stimulus	15	2672	0.36	0.0371	9606.ENSP00000257570,9606.ENSP00000268058,9606.ENSP00000306565,9606.ENSP00000333657,9606.ENSP00000343023,9606.ENSP00000354394,9606.ENSP00000359490,9606.ENSP00000360891,9606.ENSP00000368699,9606.ENSP00000369213,9606.ENSP00000381601,9606.ENSP00000395590,9606.ENSP00000399168,9606.ENSP00000413218,9606.ENSP00000446252	OASL,PML,ISG20,MX2,SP100,STAT1,GBP4,IFIT2,ISG15,DDX58,MX1,IFI35,HLA-B,FAM213B,SOD2
GO:0035455	response to interferon-alpha	2	22	1.57	0.0409	9606.ENSP00000333657,9606.ENSP00000360891	MX2,IFIT2
GO:0051457	maintenance of protein location in nucleus	2	22	1.57	0.0409	9606.ENSP00000268058,9606.ENSP00000343023	PML,SP100
GO:0002697	regulation of immune effector process	5	362	0.75	0.0485	9606.ENSP00000242351,9606.ENSP00000268058,9606.ENSP00000354394,9606.ENSP00000369213,9606.ENSP00000399168	ZC3HAV1,PML,STAT1,DDX58,HLA-B
GO:0006412	translation	5	362	0.75	0.0485	9606.ENSP00000261868,9606.ENSP00000346015,9606.ENSP00000347495,9606.ENSP00000369038,9606.ENSP00000379506	EIF3J,RPL27A,WARS,EEF1E1,RPL14
GO:0007166	cell surface receptor signaling pathway	13	2198	0.38	0.0485	9606.ENSP00000194214,9606.ENSP00000257570,9606.ENSP00000268058,9606.ENSP00000306565,9606.ENSP00000333657,9606.ENSP00000343023,9606.ENSP00000354394,9606.ENSP00000360891,9606.ENSP00000368699,9606.ENSP00000381601,9606.ENSP00000395590,9606.ENSP00000399168,9606.ENSP00000446252	HSPB11,OASL,PML,ISG20,MX2,SP100,STAT1,IFIT2,ISG15,MX1,IFI35,HLA-B,SOD2
GO:0045088	regulation of innate immune response	5	361	0.75	0.0485	9606.ENSP00000242351,9606.ENSP00000354394,9606.ENSP00000369213,9606.ENSP00000399168,9606.ENSP00000409279	ZC3HAV1,STAT1,DDX58,HLA-B,OTUD4

T = 0 h, IFNα7 / Untreated

#Term ID	Term Description	Observed Gene Count	Background Gene Count	Strength	False Discovery Rate	Matching Proteins in Your Input (IDs)	Matching Proteins in Your Input (Labels)
GO:0060337	type I interferon signaling pathway	7	65	1.79	4.09E-08	9606.ENSP00000333657,9606.ENSP00000354394,9606.ENSP00000360891,9606.ENSP00000368699,9606.ENSP00000381601,9606.ENSP00000395590,9606.ENSP00000399168	MX2,STAT1,IFIT2,ISG15,MX1,IFI35,HLA-B
GO:0002252	immune effector process	12	927	0.87	8.63E-06	9606.ENSP00000228506,9606.ENSP00000333657,9606.ENSP00000354394,9606.ENSP00000354468,9606.ENSP00000359787,9606.ENSP00000360891,9606.ENSP00000368699,9606.ENSP00000369213,9606.ENSP00000379191,9606.ENSP00000381601,9606.ENSP00000399168,9606.ENSP00000416066	MLEC,MX2,STAT1,CYB5R3,IFI44L,IFIT2,ISG15,DDX58,CD59,MX1,HLA-B,SERPINA1
GO:0051607	defense response to virus	7	181	1.35	8.63E-06	9606.ENSP00000333657,9606.ENSP00000354394,9606.ENSP00000359787,9606.ENSP00000360891,9606.ENSP00000368699,9606.ENSP00000369213,9606.ENSP00000381601	MX2,STAT1,IFI44L,IFIT2,ISG15,DDX58,MX1
GO:0006952	defense response	12	1234	0.75	1.10E-04	9606.ENSP00000262919,9606.ENSP00000333657,9606.ENSP00000354394,9606.ENSP00000359787,9606.ENSP00000360891,9606.ENSP00000368022,9606.ENSP00000368699,9606.ENSP00000369213,9606.ENSP00000381601,9606.ENSP00000395590,9606.ENSP00000399168,9606.ENSP00000416066	ATRN,MX2,STAT1,IFI44L,IFIT2,OPTN,ISG15,DDX58,MX1,IFI35,HLA-B,SERPINA1
GO:0006955	immune response	13	1560	0.68	1.60E-04	9606.ENSP00000228506,9606.ENSP00000333657,9606.ENSP00000354394,9606.ENSP00000354468,9606.ENSP00000360891,9606.ENSP00000368022,9606.ENSP00000368699,9606.ENSP00000369213,9606.ENSP00000379191,9606.ENSP00000381601,9606.ENSP00000395590,9606.ENSP00000399168,9606.ENSP00000416066	MLEC,MX2,STAT1,CYB5R3,IFIT2,OPTN,ISG15,DDX58,CD59,MX1,IFI35,HLA-B,SERPINA1
GO:0098542	defense response to other organism	10	859	0.83	1.90E-04	9606.ENSP00000333657,9606.ENSP00000354394,9606.ENSP00000359787,9606.ENSP00000360891,9606.ENSP00000368022,9606.ENSP00000368699,9606.ENSP00000369213,9606.ENSP00000381601,9606.ENSP00000395590,9606.ENSP00000399168	MX2,STAT1,IFI44L,IFIT2,OPTN,ISG15,DDX58,MX1,IFI35,HLA-B
GO:0045087	innate immune response	9	676	0.88	2.20E-04	9606.ENSP00000333657,9606.ENSP00000354394,9606.ENSP00000360891,9606.ENSP00000368022,9606.ENSP00000368699,9606.ENSP00000369213,9606.ENSP00000381601,9606.ENSP00000395590,9606.ENSP00000399168	MX2,STAT1,IFIT2,OPTN,ISG15,DDX58,MX1,IFI35,HLA-B
GO:0002376	immune system process	15	2370	0.56	3.30E-04	9606.ENSP00000228506,9606.ENSP00000264346,9606.ENSP00000333657,9606.ENSP00000354394,9606.ENSP00000354468,9606.ENSP00000359787,9606.ENSP00000360891,9606.ENSP00000368022,9606.ENSP00000368699,9606.ENSP00000369213,9606.ENSP00000379191,9606.ENSP00000381601,9606.ENSP00000395590,9606.ENSP00000399168,9606.ENSP00000416066	MLEC,HERC6,MX2,STAT1,CYB5R3,IFI44L,IFIT2,OPTN,ISG15,DDX58,CD59,MX1,IFI35,HLA-B,SERPINA1
GO:0051707	response to other organism	11	1173	0.73	3.30E-04	9606.ENSP00000264346,9606.ENSP00000333657,9606.ENSP00000354394,9606.ENSP00000359787,9606.ENSP00000360891,9606.ENSP00000368022,9606.ENSP00000368699,9606.ENSP00000369213,9606.ENSP00000381601,9606.ENSP00000395590,9606.ENSP00000399168	HERC6,MX2,STAT1,IFI44L,IFIT2,OPTN,ISG15,DDX58,MX1,IFI35,HLA-B
GO:0000266	mitochondrial fission	3	19	1.96	5.90E-04	9606.ENSP00000223136,9606.ENSP00000333657,9606.ENSP00000381601	FIS1,MX2,MX1
GO:0006950	response to stress	17	3267	0.48	5.90E-04	9606.ENSP00000223136,9606.ENSP00000262919,9606.ENSP00000264389,9606.ENSP00000333657,9606.ENSP00000354394,9606.ENSP00000359787,9606.ENSP00000360891,9606.ENSP00000368022,9606.ENSP00000368455,9606.ENSP00000368699,9606.ENSP00000369213,9606.ENSP00000379191,9606.ENSP00000381601,9606.ENSP00000395590,9606.ENSP00000399168,9606.ENSP00000416066,9606.ENSP00000416959	FIS1,ATRN,COPS4,MX2,STAT1,IFI44L,IFIT2,OPTN,NUDT2,ISG15,DDX58,CD59,MX1,IFI35,HLA-B,SERPINA1,TRA2B
GO:0051704	multi-organism process	15	2514	0.54	5.90E-04	9606.ENSP00000261574,9606.ENSP00000264346,9606.ENSP00000316357,9606.ENSP00000333657,9606.ENSP00000336712,9606.ENSP00000354394,9606.ENSP00000359787,9606.ENSP00000360891,9606.ENSP00000368022,9606.ENSP00000368699,9606.ENSP00000369213,9606.ENSP00000381601,9606.ENSP00000395590,9606.ENSP00000399168,9606.ENSP00000406209	IPO5,HERC6,USP9X,MX2,TNPO1,STAT1,IFI44L,IFIT2,OPTN,ISG15,DDX58,MX1,IFI35,HLA-B,EPN1
GO:0070887	cellular response to chemical stimulus	15	2672	0.51	1.00E-03	9606.ENSP00000199814,9606.ENSP00000223136,9606.ENSP00000261574,9606.ENSP00000316357,9606.ENSP00000333657,9606.ENSP00000354394,9606.ENSP00000360891,9606.ENSP00000368022,9606.ENSP00000368455,9606.ENSP00000368699,9606.ENSP00000369213,9606.ENSP00000381601,9606.ENSP00000395590,9606.ENSP00000399168,9606.ENSP00000416959	RBM22,FIS1,IPO5,USP9X,MX2,STAT1,IFIT2,OPTN,NUDT2,ISG15,DDX58,MX1,IFI35,HLA-B,TRA2B
GO:0048525	negative regulation of viral process	4	93	1.39	1.50E-03	9606.ENSP00000325618,9606.ENSP00000354394,9606.ENSP00000368699,9606.ENSP00000381601	PARP10,STAT1,ISG15,MX1

GO:0043901	negative regulation of multi-organism process	5	212	1.13	2.10E-03	9606.ENSP00000325618,9606.ENSP00000354394,9606.ENSP00000368699,9606.ENSP00000381601,9606.ENSP00000399168	PARP10,STAT1,ISG15,MX1,HLA-B
GO:0071310	cellular response to organic substance	13	2219	0.53	2.80E-03	9606.ENSP00000223136,9606.ENSP00000261574,9606.ENSP00000316357,9606.ENSP00000333657,9606.ENSP00000354394,9606.ENSP00000360891,9606.ENSP00000368022,9606.ENSP00000368699,9606.ENSP00000369213,9606.ENSP00000381601,9606.ENSP00000395590,9606.ENSP00000399168,9606.ENSP00000416959	FIS1,IPO5,USP9X,MX2,STAT1,IFIT2,OPTN,ISG15,DDX58,MX1,IFI35,HLA-B,TRA2B
GO:0072594	establishment of protein localization to organelle	6	396	0.94	3.30E-03	9606.ENSP00000199814,9606.ENSP00000223136,9606.ENSP00000261574,9606.ENSP00000316357,9606.ENSP00000336712,9606.ENSP00000410339	RBM22,FIS1,IPO5,USP9X,TNPO1,TOMM40
GO:0050896	response to stimulus	25	7824	0.26	4.10E-03	9606.ENSP00000199814,9606.ENSP00000216330,9606.ENSP00000223136,9606.ENSP00000228506,9606.ENSP00000261574,9606.ENSP00000262919,9606.ENSP00000264346,9606.ENSP00000264389,9606.ENSP00000316357,9606.ENSP00000333657,9606.ENSP00000354394,9606.ENSP00000354468,9606.ENSP00000359787,9606.ENSP00000360891,9606.ENSP00000368022,9606.ENSP00000368455,9606.ENSP00000368699,9606.ENSP00000369213,9606.ENSP00000379191,9606.ENSP00000381601,9606.ENSP00000395590,9606.ENSP00000399168,9606.ENSP00000406209,9606.ENSP00000416066,9606.ENSP00000416959	RBM22,FKBP3,FIS1,MLEC,IPO5,ATRN,HERC6,COPS4,USP9X,MX2,STAT1,CYB5R3,IFI44L,IFIT2,OPTN,NUDT2,ISG15,DDX58,CD59,MX1,IFI35,HLA-B,EPN1,SERPINA1,TRA2B
GO:0003374	dynamin family protein polymerization involved in mitochondrial fission	2	7	2.22	5.30E-03	9606.ENSP00000333657,9606.ENSP00000381601	MX2,MX1
GO:0006610	ribosomal protein import into nucleus	2	8	2.16	5.30E-03	9606.ENSP00000261574,9606.ENSP00000336712	IPO5,TNPO1
GO:0033365	protein localization to organelle	7	649	0.79	5.30E-03	9606.ENSP00000199814,9606.ENSP00000223136,9606.ENSP00000261574,9606.ENSP00000316357,9606.ENSP00000336712,9606.ENSP00000368022,9606.ENSP00000410339	RBM22,FIS1,IPO5,USP9X,TNPO1,OPTN,TOMM40
GO:0043900	regulation of multi-organism process	7	653	0.79	5.30E-03	9606.ENSP00000325618,9606.ENSP00000354394,9606.ENSP00000368022,9606.ENSP00000368699,9606.ENSP00000369213,9606.ENSP00000381601,9606.ENSP00000399168	PARP10,STAT1,OPTN,ISG15,DDX58,MX1,HLA-B
GO:0045071	negative regulation of viral genome replication	3	52	1.52	5.30E-03	9606.ENSP00000325618,9606.ENSP00000368699,9606.ENSP00000381601	PARP10,ISG15,MX1
GO:0017038	protein import	4	150	1.19	5.60E-03	9606.ENSP00000199814,9606.ENSP00000261574,9606.ENSP00000336712,9606.ENSP00000410339	RBM22,IPO5,TNPO1,TOMM40
GO:0009605	response to external stimulus	12	2152	0.51	6.50E-03	9606.ENSP00000223136,9606.ENSP00000264346,9606.ENSP00000333657,9606.ENSP00000354394,9606.ENSP00000359787,9606.ENSP00000360891,9606.ENSP00000368022,9606.ENSP00000368699,9606.ENSP00000369213,9606.ENSP00000381601,9606.ENSP00000395590,9606.ENSP00000399168	FIS1,HERC6,MX2,STAT1,IFI44L,IFIT2,OPTN,ISG15,DDX58,MX1,IFI35,HLA-B
GO:0016032	viral process	6	571	0.78	1.41E-02	9606.ENSP00000261574,9606.ENSP00000336712,9606.ENSP00000354394,9606.ENSP00000368699,9606.ENSP00000369213,9606.ENSP00000399168	IPO5,TNPO1,STAT1,ISG15,DDX58,HLA-B
GO:0051222	positive regulation of protein transport	5	365	0.9	1.41E-02	9606.ENSP00000199814,9606.ENSP00000223136,9606.ENSP00000261574,9606.ENSP00000345771,9606.ENSP00000369213	RBM22,FIS1,IPO5,PFKM,DDX58
GO:0006607	NLS-bearing protein import into nucleus	2	21	1.74	2.11E-02	9606.ENSP00000261574,9606.ENSP00000336712	IPO5,TNPO1
GO:0006606	protein import into nucleus	3	101	1.23	2.20E-02	9606.ENSP00000199814,9606.ENSP00000261574,9606.ENSP00000336712	RBM22,IPO5,TNPO1
GO:0035455	response to interferon-alpha	2	22	1.72	2.20E-02	9606.ENSP00000333657,9606.ENSP00000360891	MX2,IFIT2
GO:0007005	mitochondrion organization	5	424	0.83	2.21E-02	9606.ENSP00000223136,9606.ENSP00000333657,9606.ENSP00000360891,9606.ENSP00000381601,9606.ENSP00000410339	FIS1,MX2,IFIT2,MX1,TOMM40
GO:0032727	positive regulation of interferon-alpha production	2	23	1.7	2.23E-02	9606.ENSP00000354394,9606.ENSP00000369213	STAT1,DDX58
GO:0065003	protein-containing complex assembly	9	1514	0.53	2.28E-02	9606.ENSP00000199814,9606.ENSP00000223136,9606.ENSP00000333657,9606.ENSP00000345771,9606.ENSP00000379191,9606.ENSP00000381601,9606.ENSP00000416066,9606.ENSP00000416959,9606.ENSP00000464087	RBM22,FIS1,MX2,PFKM,CD59,MX1,SERPINA1,TRA2B,RNF213
GO:0002230	positive regulation of defense response to virus by host	2	26	1.65	2.58E-02	9606.ENSP00000354394,9606.ENSP00000369213	STAT1,DDX58
GO:0032479	regulation of type I interferon production	3	112	1.19	2.58E-02	9606.ENSP00000354394,9606.ENSP00000368699,9606.ENSP00000369213	STAT1,ISG15,DDX58
GO:0046822	regulation of nucleocytoplasmic transport	3	113	1.18	2.58E-02	9606.ENSP00000199814,9606.ENSP00000261574,9606.ENSP00000333657	RBM22,IPO5,MX2
GO:0046835	carbohydrate phosphorylation	2	26	1.65	2.58E-02	9606.ENSP00000288078,9606.ENSP00000345771	FUK,PFKM

GO:0051716	cellular response to stimulus	20	6212	0.27	2.58E-02	9606.ENSP00000199814,9606.ENSP00000216330,9606.ENSP00000223136,9606.ENSP00000261574,9606.ENSP00000262919,9606.ENSP00000264389,9606.ENSP00000316357,9606.ENSP00000333657,9606.ENSP00000354394,9606.ENSP00000360891,9606.ENSP00000368022,9606.ENSP00000368455,9606.ENSP00000368699,9606.ENSP00000369213,9606.ENSP00000379191,9606.ENSP00000381601,9606.ENSP00000395590,9606.ENSP00000399168,9606.ENSP00000406209,9606.ENSP00000416959	RBM22,FKBP3,FIS1,IPO5,ATRN,COPS4,USP9X,MX2,STAT1,IFIT2,OPTN,NUDT2,ISG15,DDX58,CD59,MX1,IFI35,HLA-B,EPN1,TRA2B
GO:0045648	positive regulation of erythrocyte differentiation	2	29	1.6	2.93E-02	9606.ENSP00000354394,9606.ENSP00000368699	STAT1,ISG15
GO:0033120	positive regulation of RNA splicing	2	31	1.57	3.04E-02	9606.ENSP00000199814,9606.ENSP00000416959	RBM22,TRA2B
GO:0043312	neutrophil degranulation	5	485	0.77	3.04E-02	9606.ENSP00000228506,9606.ENSP00000354468,9606.ENSP00000379191,9606.ENSP00000399168,9606.ENSP00000416066	MLEC,CYB5R3,CD59,HLA-B,SERPINA1
GO:0061024	membrane organization	6	729	0.68	3.04E-02	9606.ENSP00000223136,9606.ENSP00000333657,9606.ENSP00000379191,9606.ENSP00000381601,9606.ENSP00000406209,9606.ENSP00000416066	FIS1,MX2,CD59,MX1,EPN1,SERPINA1
GO:0090316	positive regulation of intracellular protein transport	3	126	1.14	3.04E-02	9606.ENSP00000199814,9606.ENSP00000223136,9606.ENSP00000261574	RBM22,FIS1,IPO5
GO:0009165	nucleotide biosynthetic process	4	291	0.9	3.12E-02	9606.ENSP00000288078,9606.ENSP00000325618,9606.ENSP00000345771,9606.ENSP00000363480	FUK,PARP10,PFKM,DLG3
GO:0034613	cellular protein localization	8	1367	0.53	3.35E-02	9606.ENSP00000199814,9606.ENSP00000223136,9606.ENSP00000261574,9606.ENSP00000316357,9606.ENSP00000336712,9606.ENSP00000363480,9606.ENSP00000368022,9606.ENSP00000410339	RBM22,FIS1,IPO5,USP9X,TNPO1,DLG3,OPTN,TOMM40
GO:0006810	transport	15	4130	0.32	3.54E-02	9606.ENSP00000199814,9606.ENSP00000223136,9606.ENSP00000228506,9606.ENSP00000261574,9606.ENSP00000316357,9606.ENSP00000333657,9606.ENSP00000336712,9606.ENSP00000354468,9606.ENSP00000363480,9606.ENSP00000368022,9606.ENSP00000379191,9606.ENSP00000399168,9606.ENSP00000406209,9606.ENSP00000410339,9606.ENSP00000416066	RBM22,FIS1,MLEC,IPO5,USP9X,MX2,TNPO1,CYB5R3,DLG3,OPTN,CD59,HLA-B,EPN1,TOMM40,SERPINA1
GO:0015031	protein transport	8	1391	0.52	3.57E-02	9606.ENSP00000199814,9606.ENSP00000223136,9606.ENSP00000261574,9606.ENSP00000316357,9606.ENSP00000333657,9606.ENSP00000336712,9606.ENSP00000368022,9606.ENSP00000410339	RBM22,FIS1,IPO5,USP9X,MX2,TNPO1,OPTN,TOMM40
GO:0031324	negative regulation of cellular metabolic process	11	2463	0.41	3.57E-02	9606.ENSP00000261574,9606.ENSP00000262177,9606.ENSP00000316357,9606.ENSP00000325618,9606.ENSP00000354394,9606.ENSP00000363480,9606.ENSP00000368022,9606.ENSP00000368699,9606.ENSP00000379191,9606.ENSP00000416066,9606.ENSP00000416959	IPO5,DNAJB6,USP9X,PARP10,STAT1,DLG3,OPTN,ISG15,CD59,SERPINA1,TRA2B
GO:0046907	intracellular transport	8	1390	0.52	3.57E-02	9606.ENSP00000199814,9606.ENSP00000223136,9606.ENSP00000261574,9606.ENSP00000316357,9606.ENSP00000336712,9606.ENSP00000379191,9606.ENSP00000410339,9606.ENSP00000416066	RBM22,FIS1,IPO5,USP9X,TNPO1,CD59,TOMM40,SERPINA1
GO:0016043	cellular component organization	17	5163	0.28	3.91E-02	9606.ENSP00000199814,9606.ENSP00000223136,9606.ENSP00000262177,9606.ENSP00000264389,9606.ENSP00000316357,9606.ENSP00000333657,9606.ENSP00000336712,9606.ENSP00000345771,9606.ENSP00000360891,9606.ENSP00000368022,9606.ENSP00000379191,9606.ENSP00000381601,9606.ENSP00000406209,9606.ENSP00000410339,9606.ENSP00000416066,9606.ENSP00000416959,9606.ENSP00000464087	RBM22,FIS1,DNAJB6,COPS4,USP9X,MX2,TNPO1,PFKM,IFIT2,OPTN,CD59,MX1,EPN1,TOMM40,SERPINA1,TRA2B,RNF213
GO:0032386	regulation of intracellular transport	4	335	0.84	3.91E-02	9606.ENSP00000199814,9606.ENSP00000223136,9606.ENSP00000261574,9606.ENSP00000333657	RBM22,FIS1,IPO5,MX2
GO:0032480	negative regulation of type I interferon production	2	43	1.43	3.91E-02	9606.ENSP00000368699,9606.ENSP00000369213	ISG15,DDX58
GO:0042307	positive regulation of protein import into nucleus	2	42	1.44	3.91E-02	9606.ENSP00000199814,9606.ENSP00000261574	RBM22,IPO5
GO:0043124	negative regulation of I-kappaB kinase/NF-kappaB signaling	2	42	1.44	3.91E-02	9606.ENSP00000354394,9606.ENSP00000368022	STAT1,OPTN
GO:0006886	intracellular protein transport	6	836	0.62	4.00E-02	9606.ENSP00000199814,9606.ENSP00000223136,9606.ENSP00000261574,9606.ENSP00000316357,9606.ENSP00000336712,9606.ENSP00000410339	RBM22,FIS1,IPO5,USP9X,TNPO1,TOMM40
GO:0051641	cellular localization	10	2180	0.42	4.03E-02	9606.ENSP00000199814,9606.ENSP00000223136,9606.ENSP00000261574,9606.ENSP00000316357,9606.ENSP00000336712,9606.ENSP00000363480,9606.ENSP00000368022,9606.ENSP00000379191,9606.ENSP00000410339,9606.ENSP00000416066	RBM22,FIS1,IPO5,USP9X,TNPO1,DLG3,OPTN,CD59,TOMM40,SERPINA1
GO:0042593	glucose homeostasis	3	169	1.01	4.08E-02	9606.ENSP00000223136,9606.ENSP00000345771,9606.ENSP00000416959	FIS1,PFKM,TRA2B
GO:0007166	cell surface receptor signaling pathway	10	2198	0.42	4.10E-02	9606.ENSP00000316357,9606.ENSP00000333657,9606.ENSP00000354394,9606.ENSP00000360891,9606.ENSP00000368699,9606.ENSP00000379191,9606.ENSP00000381601,9606.ENSP00000395590,9606.ENSP00000399168,9606.ENSP00000406209	USP9X,MX2,STAT1,IFIT2,ISG15,CD59,MX1,IFI35,HLA-B,EPN1

GO:0061025	membrane fusion	3	170	1.01	4.10E-02	9606.ENSP00000223136,9606.ENSP00000333657,9606.ENSP00000381601	FIS1,MX2,MX1
GO:0002697	regulation of immune effector process	4	362	0.8	4.25E-02	9606.ENSP00000354394,9606.ENSP00000369213,9606.ENSP00000379191,9606.ENSP00000399168	STAT1,DDX58,CD59,HLA-B
GO:0009435	NAD biosynthetic process	2	51	1.35	4.50E-02	9606.ENSP00000325618,9606.ENSP00000345771	PARP10,PFKM
GO:0006979	response to oxidative stress	4	373	0.79	4.63E-02	9606.ENSP00000262919,9606.ENSP00000354394,9606.ENSP00000368455,9606.ENSP00000416959	ATRN,STAT1,NUDT2,TRA2B
GO:0009987	cellular process	32	14652	0.1	4.72E-02	9606.ENSP00000199814,9606.ENSP00000216330,9606.ENSP00000223136,9606.ENSP00000228506,9606.ENSP00000261574,9606.ENSP00000262177,9606.ENSP00000262919,9606.ENSP00000264346,9606.ENSP00000264389,9606.ENSP00000288078,9606.ENSP00000316357,9606.ENSP00000325618,9606.ENSP00000333657,9606.ENSP00000336712,9606.ENSP00000345771,9606.ENSP00000354394,9606.ENSP00000354468,9606.ENSP00000360891,9606.ENSP00000363480,9606.ENSP00000368022,9606.ENSP00000368455,9606.ENSP00000368699,9606.ENSP00000369213,9606.ENSP00000379191,9606.ENSP00000381601,9606.ENSP00000395590,9606.ENSP00000399168,9606.ENSP00000406209,9606.ENSP00000410339,9606.ENSP00000416066,9606.ENSP00000416959,9606.ENSP00000464087	RBM22,FKBP3,FIS1,MLEC,IPO5,DNAJB6,ATRN,HERC6,COPS4,FUK,USP9X,PARP10,MX2,TNPO1,PFKM,STAT1,CYB5R3,IFIT2,DLG3,OPTN,NUDT2,ISG15,DDX58,CD59,MX1,IFI35,HLA-B,EPN1,TOMM40,SERPINA1,TRA2B,RNF213
GO:0032880	regulation of protein localization	6	901	0.58	4.75E-02	9606.ENSP00000199814,9606.ENSP00000223136,9606.ENSP00000261574,9606.ENSP00000262177,9606.ENSP00000345771,9606.ENSP00000369213	RBM22,FIS1,IPO5,DNAJB6,PFKM,DDX58

T = 0 h, IFNα16 / Untreated

#Term ID	Term Description	Observed Gene Count	Background Gene Count	Strength	False Discovery Rate	Matching Proteins in Your Input (IDs)	Matching Proteins in Your Input (Labels)
GO:0002906	negative regulation of mature B cell apoptotic process	2	4	2.65	1.36E-02	9606.ENSP00000009530,9606.ENSP00000361965	CD74,ADA
GO:0043085	positive regulation of catalytic activity	9	1381	0.76	1.36E-02	9606.ENSP00000009530,9606.ENSP00000263212,9606.ENSP00000321537,9606.ENSP00000325863,9606.ENSP00000363480,9606.ENSP00000374409,9606.ENSP00000377036,9606.ENSP00000378040,9606.ENSP00000379346	CD74,PPM1F,LLGL1,MRE11A,DLG3,PKP4,PPP2R4,BAD,ADNP
GO:0030155	regulation of cell adhesion	6	623	0.93	1.85E-02	9606.ENSP00000009530,9606.ENSP00000259206,9606.ENSP00000263212,9606.ENSP00000361965,9606.ENSP00000374409,9606.ENSP00000378040	CD74,IL1RN,PPM1F,ADA,PKP4,BAD
GO:0050790	regulation of catalytic activity	10	2249	0.6	1.85E-02	9606.ENSP00000009530,9606.ENSP00000263212,9606.ENSP00000321537,9606.ENSP00000325863,9606.ENSP00000363480,9606.ENSP00000374409,9606.ENSP00000377036,9606.ENSP00000378040,9606.ENSP00000379346,9606.ENSP00000416066	CD74,PPM1F,LLGL1,MRE11A,DLG3,PKP4,PPP2R4,BAD,ADNP,SERPINA1
GO:0045582	positive regulation of T cell differentiation	3	78	1.53	2.03E-02	9606.ENSP00000009530,9606.ENSP00000361965,9606.ENSP00000378040	CD74,ADA,BAD
GO:0050871	positive regulation of B cell activation	3	80	1.52	2.03E-02	9606.ENSP00000009530,9606.ENSP00000361965,9606.ENSP00000378040	CD74,ADA,BAD
GO:0042981	regulation of apoptotic process	8	1501	0.68	2.10E-02	9606.ENSP00000009530,9606.ENSP00000263212,9606.ENSP00000325863,9606.ENSP00000361965,9606.ENSP00000369038,9606.ENSP00000377036,9606.ENSP00000378040,9606.ENSP00000379346	CD74,PPM1F,MRE11A,ADA,EEF1E1,PPP2R4,BAD,ADNP
GO:0033674	positive regulation of kinase activity	5	553	0.91	2.74E-02	9606.ENSP00000009530,9606.ENSP00000325863,9606.ENSP00000363480,9606.ENSP00000378040,9606.ENSP00000379346	CD74,MRE11A,DLG3,BAD,ADNP
GO:0043549	regulation of kinase activity	6	849	0.8	2.74E-02	9606.ENSP00000009530,9606.ENSP00000263212,9606.ENSP00000325863,9606.ENSP00000363480,9606.ENSP00000378040,9606.ENSP00000379346	CD74,PPM1F,MRE11A,DLG3,BAD,ADNP
GO:0051336	regulation of hydrolase activity	7	1238	0.7	2.74E-02	9606.ENSP00000263212,9606.ENSP00000321537,9606.ENSP00000363480,9606.ENSP00000374409,9606.ENSP00000377036,9606.ENSP00000378040,9606.ENSP00000416066	PPM1F,LLGL1,DLG3,PKP4,PPP2R4,BAD,SERPINA1
GO:0065009	regulation of molecular function	11	3322	0.47	2.87E-02	9606.ENSP00000009530,9606.ENSP00000259206,9606.ENSP00000263212,9606.ENSP00000321537,9606.ENSP00000325863,9606.ENSP00000363480,9606.ENSP00000374409,9606.ENSP00000377036,9606.ENSP00000378040,9606.ENSP00000379346,9606.ENSP00000416066	CD74,IL1RN,PPM1F,LLGL1,MRE11A,DLG3,PKP4,PPP2R4,BAD,ADNP,SERPINA1
GO:0043516	regulation of DNA damage response, signal transduction by p53 class mediator	2	31	1.76	3.72E-02	9606.ENSP00000009530,9606.ENSP00000369038	CD74,EEF1E1
GO:0022407	regulation of cell-cell adhesion	4	366	0.99	3.87E-02	9606.ENSP00000009530,9606.ENSP00000259206,9606.ENSP00000361965,9606.ENSP00000378040	CD74,IL1RN,ADA,BAD
GO:0043094	cellular metabolic compound salvage	2	36	1.69	3.98E-02	9606.ENSP00000273920,9606.ENSP00000361965	ENOPH1,ADA
GO:0045785	positive regulation of cell adhesion	4	375	0.98	3.98E-02	9606.ENSP00000009530,9606.ENSP00000263212,9606.ENSP00000361965,9606.ENSP00000378040	CD74,PPM1F,ADA,BAD
GO:0048519	negative regulation of biological process	13	4953	0.37	3.98E-02	9606.ENSP00000009530,9606.ENSP00000259206,9606.ENSP00000263212,9606.ENSP00000325863,9606.ENSP00000357980,9606.ENSP00000361965,9606.ENSP00000363480,9606.ENSP00000369038,9606.ENSP00000377036,9606.ENSP00000378040,9606.ENSP00000379346,9606.ENSP00000397798,9606.ENSP00000416066	CD74,IL1RN,PPM1F,MRE11A,HTRA1,ADA,DLG3,EEF1E1,PPP2R4,BAD,ADNP,RPL17,SERPINA1

GO:0045937	positive regulation of phosphate metabolic process	6	1052	0.71	4.01E-02	9606.ENSP00000009530,9606.ENSP00000325863,9606.ENSP00000363480,9606.ENSP00000377036,9606.ENSP00000378040,9606.ENSP00000379346	CD74,MRE11A,DLG3,PPP2R4,BAD,ADNP
GO:0032270	positive regulation of cellular protein metabolic process	7	1496	0.62	4.10E-02	9606.ENSP00000009530,9606.ENSP00000263212,9606.ENSP00000325863,9606.ENSP00000363480,9606.ENSP00000377036,9606.ENSP00000378040,9606.ENSP00000379346	CD74,PPM1F,MRE11A,DLG3,PPP2R4,BAD,ADNP
GO:0046903	secretion	6	1070	0.7	4.10E-02	9606.ENSP00000259206,9606.ENSP00000278505,9606.ENSP00000321537,9606.ENSP00000355605,9606.ENSP00000361965,9606.ENSP00000416066	IL1RN,ENDOD1,LLGL1,EXOC8,ADA,SERPINA1
GO:0030890	positive regulation of B cell proliferation	2	42	1.63	4.32E-02	9606.ENSP00000009530,9606.ENSP00000361965	CD74,ADA
GO:0050731	positive regulation of peptidyl-tyrosine phosphorylation	3	180	1.17	4.32E-02	9606.ENSP00000009530,9606.ENSP00000363480,9606.ENSP00000379346	CD74,DLG3,ADNP
GO:0006953	acute-phase response	2	45	1.6	4.50E-02	9606.ENSP00000259206,9606.ENSP00000416066	IL1RN,SERPINA1
GO:0044092	negative regulation of molecular function	6	1119	0.68	4.50E-02	9606.ENSP00000259206,9606.ENSP00000263212,9606.ENSP00000363480,9606.ENSP00000377036,9606.ENSP00000379346,9606.ENSP00000416066	IL1RN,PPM1F,DLG3,PPP2R4,ADNP,SERPINA1
GO:0048523	negative regulation of cellular process	12	4454	0.38	4.50E-02	9606.ENSP00000009530,9606.ENSP00000259206,9606.ENSP00000263212,9606.ENSP00000325863,9606.ENSP00000357980,9606.ENSP00000361965,9606.ENSP00000363480,9606.ENSP00000369038,9606.ENSP00000377036,9606.ENSP00000378040,9606.ENSP00000379346,9606.ENSP00000416066	CD74,IL1RN,PPM1F,MRE11A,HTRA1,ADA,DLG3,EEF1E1,PPP2R4,BAD,ADNP,SERPINA1
GO:0051345	positive regulation of hydrolase activity	5	742	0.78	4.50E-02	9606.ENSP00000263212,9606.ENSP00000321537,9606.ENSP00000374409,9606.ENSP00000377036,9606.ENSP00000378040	PPM1F,LLGL1,PKP4,PPP2R4,BAD

T = 0 h, IFNλ3 / Untreated

#Term ID	Term Description	Observed Gene Count	Background Gene Count	Strength	False Discovery Rate	Matching Proteins in Your Input (IDs)	Matching Proteins in Your Input (Labels)
GO:0060337	type I interferon signaling pathway	9	65	1.83	3.01E-11	9606.ENSP00000257570,9606.ENSP00000306565,9606.ENSP00000333657,9606.ENSP00000354394,9606.ENSP00000360891,9606.ENSP00000368699,9606.ENSP00000381601,9606.ENSP00000395590,9606.ENSP00000399168	OASL,ISG20,MX2,STAT1,IFIT2,ISG15,MX1,IFI35,HLA-B
GO:0051607	defense response to virus	9	181	1.39	4.23E-08	9606.ENSP00000257570,9606.ENSP00000306565,9606.ENSP00000333657,9606.ENSP00000354394,9606.ENSP00000359787,9606.ENSP00000360891,9606.ENSP00000368699,9606.ENSP00000377344,9606.ENSP00000381601	OASL,ISG20,MX2,STAT1,IFI44L,IFIT2,ISG15,DDX60,MX1
GO:0002252	immune effector process	13	927	0.84	5.02E-06	9606.ENSP00000257570,9606.ENSP00000262746,9606.ENSP00000265016,9606.ENSP00000306565,9606.ENSP00000333657,9606.ENSP00000354394,9606.ENSP00000354468,9606.ENSP00000359787,9606.ENSP00000360891,9606.ENSP00000368699,9606.ENSP00000377344,9606.ENSP00000381601,9606.ENSP00000399168	OASL,PRDX1,BST1,ISG20,MX2,STAT1,CYB5R3,IFI44L,IFIT2,ISG15,DDX60,MX1,HLA-B
GO:0051186	cofactor metabolic process	10	467	1.02	5.35E-06	9606.ENSP00000262746,9606.ENSP00000265016,9606.ENSP00000265594,9606.ENSP00000307241,9606.ENSP00000311984,9606.ENSP00000325618,9606.ENSP00000327070,9606.ENSP00000352584,9606.ENSP00000354468,9606.ENSP00000361140	PRDX1,BST1,MCCC1,PDHB,CARKD,PARP10,MDH2,AKR1B10,CYB5R3,AKR1A1
GO:0048525	negative regulation of viral process	6	93	1.5	7.63E-06	9606.ENSP00000257570,9606.ENSP00000306565,9606.ENSP00000325618,9606.ENSP00000354394,9606.ENSP00000368699,9606.ENSP00000381601	OASL,ISG20,PARP10,STAT1,ISG15,MX1
GO:0050792	regulation of viral process	7	174	1.29	1.05E-05	9606.ENSP00000254950,9606.ENSP00000257570,9606.ENSP00000306565,9606.ENSP00000325618,9606.ENSP00000354394,9606.ENSP00000368699,9606.ENSP00000381601	VPS4A,OASL,ISG20,PARP10,STAT1,ISG15,MX1
GO:0045087	innate immune response	11	676	0.9	1.06E-05	9606.ENSP00000257570,9606.ENSP00000262746,9606.ENSP00000306565,9606.ENSP00000333657,9606.ENSP00000354394,9606.ENSP00000360891,9606.ENSP00000368699,9606.ENSP00000377344,9606.ENSP00000381601,9606.ENSP00000395590,9606.ENSP00000399168	OASL,PRDX1,ISG20,MX2,STAT1,IFIT2,ISG15,DDX60,MX1,IFI35,HLA-B
GO:0045071	negative regulation of viral genome replication	5	52	1.67	1.13E-05	9606.ENSP00000257570,9606.ENSP00000306565,9606.ENSP00000325618,9606.ENSP00000368699,9606.ENSP00000381601	OASL,ISG20,PARP10,ISG15,MX1
GO:0098542	defense response to other organism	12	859	0.83	1.13E-05	9606.ENSP00000257570,9606.ENSP00000262746,9606.ENSP00000306565,9606.ENSP00000333657,9606.ENSP00000354394,9606.ENSP00000359787,9606.ENSP00000360891,9606.ENSP00000368699,9606.ENSP00000377344,9606.ENSP00000381601,9606.ENSP00000395590,9606.ENSP00000399168	OASL,PRDX1,ISG20,MX2,STAT1,IFI44L,IFIT2,ISG15,DDX60,MX1,IFI35,HLA-B
GO:0006732	coenzyme metabolic process	8	297	1.12	1.54E-05	9606.ENSP00000265016,9606.ENSP00000265594,9606.ENSP00000307241,9606.ENSP00000311984,9606.ENSP00000325618,9606.ENSP00000327070,9606.ENSP00000354468,9606.ENSP00000361140	BST1,MCCC1,PDHB,CARKD,PARP10,MDH2,CYB5R3,AKR1A1
GO:0043901	negative regulation of multi-organism process	7	212	1.21	2.30E-05	9606.ENSP00000257570,9606.ENSP00000306565,9606.ENSP00000325618,9606.ENSP00000354394,9606.ENSP00000368699,9606.ENSP00000381601,9606.ENSP00000399168	OASL,ISG20,PARP10,STAT1,ISG15,MX1,HLA-B

GO:0009605	response to external stimulus	17	2152	0.59	2.83E-05	9606.ENSP00000223136,9606.ENSP00000257570,9606.ENSP00000262746,9606.ENSP00000264108,9606.ENSP00000264346,9606.ENSP00000306565,9606.ENSP00000317790,9606.ENSP00000333657,9606.ENSP00000354394,9606.ENSP00000359787,9606.ENSP00000360891,9606.ENSP00000368699,9606.ENSP00000377344,9606.ENSP00000381601,9606.ENSP00000395590,9606.ENSP00000399168,9606.ENSP00000457513	FIS1,OASL,PRDX1,HAT1,HERC6,ISG20,SPTBN5,MX2,STAT1,IFI44L,IFIT2,ISG15,DDX60,MX1,IFI35,HLA-B,COX4I1
GO:0051707	response to other organism	13	1173	0.73	2.83E-05	9606.ENSP00000257570,9606.ENSP00000262746,9606.ENSP00000264346,9606.ENSP00000306565,9606.ENSP00000333657,9606.ENSP00000354394,9606.ENSP00000359787,9606.ENSP00000360891,9606.ENSP00000368699,9606.ENSP00000377344,9606.ENSP00000381601,9606.ENSP00000395590,9606.ENSP00000399168	OASL,PRDX1,HERC6,ISG20,MX2,STAT1,IFI44L,IFIT2,ISG15,DDX60,MX1,IFI35,HLA-B
GO:1903900	regulation of viral life cycle	6	135	1.34	2.83E-05	9606.ENSP00000254950,9606.ENSP00000257570,9606.ENSP00000306565,9606.ENSP00000325618,9606.ENSP00000368699,9606.ENSP00000381601	VPS4A,OASL,ISG20,PARP10,ISG15,MX1
GO:0019221	cytokine-mediated signaling pathway	10	655	0.87	3.89E-05	9606.ENSP00000257570,9606.ENSP00000306565,9606.ENSP00000333657,9606.ENSP00000354394,9606.ENSP00000360891,9606.ENSP00000368699,9606.ENSP00000381601,9606.ENSP00000395590,9606.ENSP00000399168,9606.ENSP00000480826	OASL,ISG20,MX2,STAT1,IFIT2,ISG15,MX1,IFI35,HLA-B,HIST1H3A
GO:0046496	nicotinamide nucleotide metabolic process	5	107	1.36	1.70E-04	9606.ENSP00000265016,9606.ENSP00000307241,9606.ENSP00000311984,9606.ENSP00000325618,9606.ENSP00000327070	BST1,PDHB,CARKD,PARP10,MDH2
GO:0043900	regulation of multi-organism process	9	653	0.83	2.70E-04	9606.ENSP00000254950,9606.ENSP00000257570,9606.ENSP00000306565,9606.ENSP00000325618,9606.ENSP00000354394,9606.ENSP00000368699,9606.ENSP00000377344,9606.ENSP00000381601,9606.ENSP00000399168	VPS4A,OASL,ISG20,PARP10,STAT1,ISG15,DDX60,MX1,HLA-B
GO:0006733	oxidoreduction coenzyme metabolic process	5	124	1.29	2.80E-04	9606.ENSP00000265016,9606.ENSP00000307241,9606.ENSP00000311984,9606.ENSP00000325618,9606.ENSP00000327070	BST1,PDHB,CARKD,PARP10,MDH2
GO:0006955	immune response	13	1560	0.61	3.60E-04	9606.ENSP00000257570,9606.ENSP00000262746,9606.ENSP00000265016,9606.ENSP00000306565,9606.ENSP00000333657,9606.ENSP00000354394,9606.ENSP00000354468,9606.ENSP00000360891,9606.ENSP00000368699,9606.ENSP00000377344,9606.ENSP00000381601,9606.ENSP00000395590,9606.ENSP00000399168	OASL,PRDX1,BST1,ISG20,MX2,STAT1,CYB5R3,IFIT2,ISG15,DDX60,MX1,IFI35,HLA-B
GO:0000266	mitochondrial fission	3	19	1.89	4.90E-04	9606.ENSP00000223136,9606.ENSP00000333657,9606.ENSP00000381601	FIS1,MX2,MX1
GO:0019674	NAD metabolic process	4	66	1.47	5.00E-04	9606.ENSP00000265016,9606.ENSP00000311984,9606.ENSP00000325618,9606.ENSP00000327070	BST1,CARKD,PARP10,MDH2
GO:0055114	oxidation-reduction process	10	932	0.72	5.50E-04	9606.ENSP00000262746,9606.ENSP00000307241,9606.ENSP00000315013,9606.ENSP00000325655,9606.ENSP00000327070,9606.ENSP00000352584,9606.ENSP00000354468,9606.ENSP00000356218,9606.ENSP00000361140,9606.ENSP00000457513	PRDX1,PDHB,CRAT,CHCHD5,MDH2,AKR1B10,CYB5R3,CYB5R1,AKR1A1,COX4I1
GO:0009060	aerobic respiration	4	71	1.44	6.10E-04	9606.ENSP00000307241,9606.ENSP00000325655,9606.ENSP00000327070,9606.ENSP00000457513	PDHB,CHCHD5,MDH2,COX4I1
GO:0051704	multi-organism process	16	2514	0.49	6.10E-04	9606.ENSP00000254950,9606.ENSP00000257570,9606.ENSP00000262746,9606.ENSP00000264346,9606.ENSP00000306565,9606.ENSP00000333657,9606.ENSP00000354394,9606.ENSP00000359787,9606.ENSP00000360891,9606.ENSP00000368699,9606.ENSP00000377344,9606.ENSP00000379182,9606.ENSP00000381601,9606.ENSP00000395590,9606.ENSP00000399168,9606.ENSP00000432305	VPS4A,OASL,PRDX1,HERC6,ISG20,MX2,STAT1,IFI44L,IFIT2,ISG15,DDX60,RAE1,MX1,IFI35,HLA-B,SPA17

GO:0002376	immune system process	15	2370	0.49	1.20E-03	9606.ENSP00000257570,9606.ENSP00000262746,9606.ENSP00000264346,9606.ENSP00000265016,9606.ENSP00000306565,9606.ENSP00000333657,9606.ENSP00000354394,9606.ENSP00000354468,9606.ENSP00000359787,9606.ENSP00000360891,9606.ENSP00000368699,9606.ENSP00000377344,9606.ENSP00000381601,9606.ENSP00000395590,9606.ENSP00000399168	OASL,PRDX1,HERC6,BST1,ISG20,MX2,STAT1,CYB5R3,IFI44L,IFIT2,ISG15,DDX60,MX1,IFI35,HLA-B
GO:0016999	antibiotic metabolic process	4	124	1.2	4.40E-03	9606.ENSP00000262746,9606.ENSP00000307241,9606.ENSP00000327070,9606.ENSP00000352584	PRDX1,PDHB,MDH2,AKR1B10
GO:0003374	dynamin family protein polymerization involved in mitochondrial fission	2	7	2.15	4.70E-03	9606.ENSP00000333657,9606.ENSP00000381601	MX2,MX1
GO:0072594	establishment of protein localization to organelle	6	396	0.87	4.70E-03	9606.ENSP00000199814,9606.ENSP00000223136,9606.ENSP00000254950,9606.ENSP00000315013,9606.ENSP00000379182,9606.ENSP00000397798	RBM22,FIS1,VPS4A,CRAT,RAE1,RPL17
GO:0019852	L-ascorbic acid metabolic process	2	11	1.95	9.10E-03	9606.ENSP00000354468,9606.ENSP00000361140	CYB5R3,AKR1A1
GO:0009108	coenzyme biosynthetic process	4	167	1.07	1.12E-02	9606.ENSP00000307241,9606.ENSP00000311984,9606.ENSP00000325618,9606.ENSP00000361140	PDHB,CARKD,PARP10,AKR1A1
GO:0060333	interferon-gamma-mediated signaling pathway	3	69	1.33	1.16E-02	9606.ENSP00000257570,9606.ENSP00000354394,9606.ENSP00000399168	OASL,STAT1,HLA-B
GO:0061025	membrane fusion	4	170	1.06	1.16E-02	9606.ENSP00000223136,9606.ENSP00000254950,9606.ENSP00000333657,9606.ENSP00000381601	FIS1,VPS4A,MX2,MX1
GO:0034356	NAD biosynthesis via nicotinamide riboside salvage pathway	2	15	1.81	1.42E-02	9606.ENSP00000311984,9606.ENSP00000325618	CARKD,PARP10
GO:0070887	cellular response to chemical stimulus	14	2672	0.41	1.43E-02	9606.ENSP00000199814,9606.ENSP00000223136,9606.ENSP00000257570,9606.ENSP00000262746,9606.ENSP00000306565,9606.ENSP00000333657,9606.ENSP00000354394,9606.ENSP00000360891,9606.ENSP00000368699,9606.ENSP00000379182,9606.ENSP00000381601,9606.ENSP00000395590,9606.ENSP00000399168,9606.ENSP00000480826	RBM22,FIS1,OASL,PRDX1,ISG20,MX2,STAT1,IFIT2,ISG15,RAE1,MX1,IFI35,HLA-B,HIST1H3A
GO:0006767	water-soluble vitamin metabolic process	3	81	1.26	1.68E-02	9606.ENSP00000265594,9606.ENSP00000354468,9606.ENSP00000361140	MCCC1,CYB5R3,AKR1A1
GO:0044281	small molecule metabolic process	11	1779	0.48	1.68E-02	9606.ENSP00000265016,9606.ENSP00000265594,9606.ENSP00000307241,9606.ENSP00000311984,9606.ENSP00000315013,9606.ENSP00000325618,9606.ENSP00000327070,9606.ENSP00000352584,9606.ENSP00000354468,9606.ENSP00000361140,9606.ENSP00000457513	BST1,MCCC1,PDHB,CARKD,CRAT,PARP10,MDH2,AKR1B10,CYB5R3,AKR1A1,COX4I1
GO:0005996	monosaccharide metabolic process	4	198	0.99	1.81E-02	9606.ENSP00000307241,9606.ENSP00000327070,9606.ENSP00000354468,9606.ENSP00000361140	PDHB,MDH2,CYB5R3,AKR1A1
GO:0009117	nucleotide metabolic process	6	576	0.71	2.49E-02	9606.ENSP00000265016,9606.ENSP00000307241,9606.ENSP00000311984,9606.ENSP00000325618,9606.ENSP00000327070,9606.ENSP00000457513	BST1,PDHB,CARKD,PARP10,MDH2,COX4I1
GO:0035455	response to interferon-alpha	2	22	1.65	2.49E-02	9606.ENSP00000333657,9606.ENSP00000360891	MX2,IFIT2
GO:0051188	cofactor biosynthetic process	4	218	0.95	2.49E-02	9606.ENSP00000307241,9606.ENSP00000311984,9606.ENSP00000325618,9606.ENSP00000361140	PDHB,CARKD,PARP10,AKR1A1
GO:0071310	cellular response to organic substance	12	2219	0.42	2.62E-02	9606.ENSP00000223136,9606.ENSP00000257570,9606.ENSP00000306565,9606.ENSP00000333657,9606.ENSP00000354394,9606.ENSP00000360891,9606.ENSP00000368699,9606.ENSP00000379182,9606.ENSP00000381601,9606.ENSP00000395590,9606.ENSP00000399168,9606.ENSP00000480826	FIS1,OASL,ISG20,MX2,STAT1,IFIT2,ISG15,RAE1,MX1,IFI35,HLA-B,HIST1H3A
GO:0006091	generation of precursor metabolites and energy	5	400	0.79	2.85E-02	9606.ENSP00000307241,9606.ENSP00000325655,9606.ENSP00000327070,9606.ENSP00000361140,9606.ENSP00000457513	PDHB,CHCHD5,MDH2,AKR1A1,COX4I1

GO:0044248	cellular catabolic process	10	1646	0.47	3.05E-02	9606.ENSP00000223136,9606.ENSP00000254950,9606.ENSP00000262746,9606.ENSP00000265594,9606.ENSP00000306565,9606.ENSP00000315013,9606.ENSP00000352584,9606.ENSP00000361140,9606.ENSP00000368699,9606.ENSP00000397798	FIS1,VPS4A,PRDX1,MCCC1,ISG20,CRAT,AKR1B10,AKR1A1,ISG15,RPL17
GO:0017144	drug metabolic process	6	622	0.67	3.32E-02	9606.ENSP00000262746,9606.ENSP00000265594,9606.ENSP00000307241,9606.ENSP00000327070,9606.ENSP00000352584,9606.ENSP00000457513	PRDX1,MCCC1,PDHB,MDH2,AKR1B10,COX4I1
GO:0006006	glucose metabolic process	3	113	1.11	3.47E-02	9606.ENSP00000307241,9606.ENSP00000327070,9606.ENSP00000361140	PDHB,MDH2,AKR1A1
GO:0045648	positive regulation of erythrocyte differentiation	2	29	1.53	3.65E-02	9606.ENSP00000354394,9606.ENSP00000368699	STAT1,ISG15
GO:0042221	response to chemical	17	4153	0.3	3.86E-02	9606.ENSP00000199814,9606.ENSP00000223136,9606.ENSP00000257570,9606.ENSP00000262746,9606.ENSP00000264108,9606.ENSP00000306565,9606.ENSP00000317790,9606.ENSP00000333657,9606.ENSP00000354394,9606.ENSP00000360891,9606.ENSP00000368699,9606.ENSP00000379182,9606.ENSP00000381601,9606.ENSP00000395590,9606.ENSP00000399168,9606.ENSP00000457513,9606.ENSP00000480826	RBM22,FIS1,OASL,PRDX1,HAT1,ISG20,SPTBN5,MX2,STAT1,IFIT2,ISG15,RAE1,MX1,IFI35,HLA-B,COX4I1,HIST1H3A
GO:0006099	tricarboxylic acid cycle	2	32	1.49	4.13E-02	9606.ENSP00000307241,9606.ENSP00000327070	PDHB,MDH2
GO:0006335	DNA replication-dependent nucleosome assembly	2	32	1.49	4.13E-02	9606.ENSP00000264108,9606.ENSP00000480826	HAT1,HIST1H3A
GO:0043933	protein-containing complex subunit organization	10	1770	0.44	4.44E-02	9606.ENSP00000199814,9606.ENSP00000223136,9606.ENSP00000254950,9606.ENSP00000264108,9606.ENSP00000265594,9606.ENSP00000317790,9606.ENSP00000333657,9606.ENSP00000381601,9606.ENSP00000392617,9606.ENSP00000480826	RBM22,FIS1,VPS4A,HAT1,MCCC1,SPTBN5,MX2,MX1,SMARCD2,HIST1H3A
GO:0006101	citrate metabolic process	2	36	1.43	4.79E-02	9606.ENSP00000307241,9606.ENSP00000327070	PDHB,MDH2

T = 72 h (CoV-2), IFNα5 / Untreated

#Term ID	Term Description	Observed Gene Count	Background Gene Count	Strength	False Discovery Rate	Matching Proteins in Your Input (IDs)	Matching Proteins in Your Input (Labels)
GO:0034340	response to type I interferon	17	70	1.84	1.33E-22	9606.ENSP00000228928,9606.ENSP00000253110,9606.ENSP00000257570,9606.ENSP00000315768,9606.ENSP00000333657,9606.ENSP00000343023,9606.ENSP00000354394,9606.ENSP00000357459,9606.ENSP00000360869,9606.ENSP00000360883,9606.ENSP00000365402,9606.ENSP00000379873,9606.ENSP00000381601,9606.ENSP00000382707,9606.ENSP00000388001,9606.ENSP00000395590,9606.ENSP00000399168	OAS3,C19orf66,OASL,STAT2,MX2,SP100,STAT1,ADAR,IFIT1,IFIT3,HLA-C,HLA-A,MX1,IFITM3,OAS1,IFI35,HLA-B
GO:0060337	type I interferon signaling pathway	16	65	1.85	1.72E-21	9606.ENSP00000228928,9606.ENSP00000257570,9606.ENSP00000315768,9606.ENSP00000333657,9606.ENSP00000343023,9606.ENSP00000354394,9606.ENSP00000357459,9606.ENSP00000360869,9606.ENSP00000360883,9606.ENSP00000365402,9606.ENSP00000379873,9606.ENSP00000381601,9606.ENSP00000382707,9606.ENSP00000388001,9606.ENSP00000395590,9606.ENSP00000399168	OAS3,OASL,STAT2,MX2,SP100,STAT1,ADAR,IFIT1,IFIT3,HLA-C,HLA-A,MX1,IFITM3,OAS1,IFI35,HLA-B
GO:0045087	innate immune response	26	676	1.04	7.78E-18	9606.ENSP00000228928,9606.ENSP00000233057,9606.ENSP00000243253,9606.ENSP00000253110,9606.ENSP00000257570,9606.ENSP00000268058,9606.ENSP00000315768,9606.ENSP00000333657,9606.ENSP00000343023,9606.ENSP00000354394,9606.ENSP00000357459,9606.ENSP00000359490,9606.ENSP00000360869,9606.ENSP00000360883,9606.ENSP00000365402,9606.ENSP00000369299,9606.ENSP00000378856,9606.ENSP00000379873,9606.ENSP00000381601,9606.ENSP00000382707,9606.ENSP00000388001,9606.ENSP00000395590,9606.ENSP00000398632,9606.ENSP00000399168,9606.ENSP00000418194,9606.ENSP00000433138	OAS3,EIF2AK2,SEC61A1,C19orf66,OASL,PML,STAT2,MX2,SP100,STAT1,ADAR,GBP4,IFIT1,IFIT3,HLA-C,TRIM22,LGALS9,HLA-A,MX1,IFITM3,OAS1,IFI35,CD44,HLA-B,PARP14,CASP1
GO:0043901	negative regulation of multi-organism process	18	212	1.39	2.53E-17	9606.ENSP00000228928,9606.ENSP00000233057,9606.ENSP00000253110,9606.ENSP00000257570,9606.ENSP00000268058,9606.ENSP00000325618,9606.ENSP00000343023,9606.ENSP00000354394,9606.ENSP00000357459,9606.ENSP00000360869,9606.ENSP00000374455,9606.ENSP00000378856,9606.ENSP00000379873,9606.ENSP00000381601,9606.ENSP00000382707,9606.ENSP00000388001,9606.ENSP00000399168,9606.ENSP00000418194	OAS3,EIF2AK2,C19orf66,OASL,PML,PARP10,SP100,STAT1,ADAR,IFIT1,SQSTM1,LGALS9,HLA-A,MX1,IFITM3,OAS1,HLA-B,PARP14
GO:0034341	response to interferon-gamma	17	176	1.44	3.35E-17	9606.ENSP00000228928,9606.ENSP00000243253,9606.ENSP00000253110,9606.ENSP00000257570,9606.ENSP00000268058,9606.ENSP00000343023,9606.ENSP00000354394,9606.ENSP00000359490,9606.ENSP00000365402,9606.ENSP00000369299,9606.ENSP00000378856,9606.ENSP00000379873,9606.ENSP00000382707,9606.ENSP00000388001,9606.ENSP00000398632,9606.ENSP00000399168,9606.ENSP00000433138	OAS3,SEC61A1,C19orf66,OASL,PML,SP100,STAT1,GBP4,HLA-C,TRIM22,LGALS9,HLA-A,IFITM3,OAS1,CD44,HLA-B,CASP1

GO:0098542	defense response to other organism	27	859	0.96	1.00E-16	9606.ENSP00000228928,9606.ENSP00000233057,9606.ENSP00000243253,9606.ENSP00000253110,9606.ENSP00000257570,9606.ENSP00000268058,9606.ENSP00000315768,9606.ENSP00000333657,9606.ENSP00000343023,9606.ENSP00000354394,9606.ENSP00000357459,9606.ENSP00000359490,9606.ENSP00000359787,9606.ENSP00000360869,9606.ENSP00000360883,9606.ENSP00000365402,9606.ENSP00000369299,9606.ENSP00000378856,9606.ENSP00000379873,9606.ENSP00000381601,9606.ENSP00000382707,9606.ENSP00000388001,9606.ENSP00000395590,9606.ENSP00000398632,9606.ENSP00000399168,9606.ENSP00000418194,9606.ENSP00000433138	OAS3,EIF2AK2,SEC61A1,C19orf66,OASL,PML,STAT2,MX2,SP100,STAT1,ADAR,GBP4,IFI44L,IFIT1,IFIT3,HLA-C,TRIM22,LGALS9,HLA-A,MX1,IFITM3,OAS1,IFI35,CD44,HLA-B,PARP14,CASP1
GO:0009615	response to virus	18	270	1.28	9.06E-16	9606.ENSP00000228928,9606.ENSP00000233057,9606.ENSP00000253110,9606.ENSP00000257570,9606.ENSP00000268058,9606.ENSP00000315768,9606.ENSP00000333657,9606.ENSP00000354394,9606.ENSP00000357459,9606.ENSP00000359783,9606.ENSP00000359787,9606.ENSP00000360869,9606.ENSP00000360883,9606.ENSP00000369299,9606.ENSP00000378856,9606.ENSP00000381601,9606.ENSP00000382707,9606.ENSP00000388001	OAS3,EIF2AK2,C19orf66,OASL,PML,STAT2,MX2,STAT1,ADAR,IFI44,IFI44L,IFIT1,IFIT3,TRIM22,LGALS9,MX1,IFITM3,OAS1
GO:0051607	defense response to virus	16	181	1.41	1.12E-15	9606.ENSP00000228928,9606.ENSP00000233057,9606.ENSP00000253110,9606.ENSP00000257570,9606.ENSP00000268058,9606.ENSP00000315768,9606.ENSP00000333657,9606.ENSP00000354394,9606.ENSP00000357459,9606.ENSP00000359787,9606.ENSP00000360869,9606.ENSP00000360883,9606.ENSP00000369299,9606.ENSP00000381601,9606.ENSP00000382707,9606.ENSP00000388001	OAS3,EIF2AK2,C19orf66,OASL,PML,STAT2,MX2,STAT1,ADAR,IFI44L,IFIT1,IFIT3,TRIM22,MX1,IFITM3,OAS1
GO:0051707	response to other organism	29	1173	0.85	1.23E-15	9606.ENSP00000228928,9606.ENSP00000233057,9606.ENSP00000243253,9606.ENSP00000253110,9606.ENSP00000256722,9606.ENSP00000257570,9606.ENSP00000268058,9606.ENSP00000315768,9606.ENSP00000333657,9606.ENSP00000343023,9606.ENSP00000354394,9606.ENSP00000357459,9606.ENSP00000359490,9606.ENSP00000359783,9606.ENSP00000359787,9606.ENSP00000360869,9606.ENSP00000360883,9606.ENSP00000365402,9606.ENSP00000369299,9606.ENSP00000378856,9606.ENSP00000379873,9606.ENSP00000381601,9606.ENSP00000382707,9606.ENSP00000388001,9606.ENSP00000395590,9606.ENSP00000398632,9606.ENSP00000399168,9606.ENSP00000418194,9606.ENSP00000433138	OAS3,EIF2AK2,SEC61A1,C19orf66,CMPK2,OASL,PML,STAT2,MX2,SP100,STAT1,ADAR,GBP4,IFI44,IFI44L,IFIT1,IFIT3,HLA-C,TRIM22,LGALS9,HLA-A,MX1,IFITM3,OAS1,IFI35,CD44,HLA-B,PARP14,CASP1
GO:0006952	defense response	29	1234	0.83	3.55E-15	9606.ENSP00000228928,9606.ENSP00000233057,9606.ENSP00000243253,9606.ENSP00000249066,9606.ENSP00000253110,9606.ENSP00000257570,9606.ENSP00000262776,9606.ENSP00000268058,9606.ENSP00000315768,9606.ENSP00000333657,9606.ENSP00000343023,9606.ENSP00000354394,9606.ENSP00000357459,9606.ENSP00000359490,9606.ENSP00000359787,9606.ENSP00000360869,9606.ENSP00000360883,9606.ENSP00000365402,9606.ENSP00000369299,9606.ENSP00000378856,9606.ENSP00000379873,9606.ENSP00000381601,9606.ENSP00000382707,9606.ENSP00000388001,9606.ENSP00000395590,9606.ENSP00000398632,9606.ENSP00000399168,9606.ENSP00000418194,9606.ENSP00000433138	OAS3,EIF2AK2,SEC61A1,APOL2,C19orf66,OASL,LGALS3BP,PML,STAT2,MX2,SP100,STAT1,ADAR,GBP4,IFI44L,IFIT1,IFIT3,HLA-C,TRIM22,LGALS9,HLA-A,MX1,IFITM3,OAS1,IFI35,CD44,HLA-B,PARP14,CASP1

GO:0034097	response to cytokine	27	1035	0.88	4.92E-15	9606.ENSP00000228928,9606.ENSP00000233057,9606.ENSP00000243253,9606.ENSP00000253110,9606.ENSP00000257570,9606.ENSP00000268058,9606.ENSP00000315768,9606.ENSP00000317997,9606.ENSP00000333657,9606.ENSP00000343023,9606.ENSP00000354394,9606.ENSP00000357459,9606.ENSP00000359490,9606.ENSP00000360869,9606.ENSP00000360883,9606.ENSP00000365402,9606.ENSP00000369299,9606.ENSP00000374455,9606.ENSP00000378856,9606.ENSP00000379873,9606.ENSP00000381601,9606.ENSP00000382707,9606.ENSP00000388001,9606.ENSP00000395590,9606.ENSP00000398632,9606.ENSP00000399168,9606.ENSP00000433138	OAS3,EIF2AK2,SEC61A1,C19orf66,OASL,PML,STAT2,DUOX1,MX2,SP100,STAT1,ADAR,GBP4,IFIT1,IFIT3,HLA-C,TRIM22,SQSTM1,LGALS9,HLA-A,MX1,IFITM3,OAS1,IFI35,CD44,HLA-B,CASP1
GO:0048525	negative regulation of viral process	13	93	1.6	4.92E-15	9606.ENSP00000228928,9606.ENSP00000233057,9606.ENSP00000253110,9606.ENSP00000257570,9606.ENSP00000268058,9606.ENSP00000325618,9606.ENSP00000343023,9606.ENSP00000354394,9606.ENSP00000357459,9606.ENSP00000360869,9606.ENSP00000381601,9606.ENSP00000382707,9606.ENSP00000388001	OAS3,EIF2AK2,C19orf66,OASL,PML,PARP10,SP100,STAT1,ADAR,IFIT1,MX1,IFITM3,OAS1
GO:0019221	cytokine-mediated signaling pathway	22	655	0.99	5.13E-14	9606.ENSP00000228928,9606.ENSP00000257570,9606.ENSP00000268058,9606.ENSP00000315768,9606.ENSP00000317997,9606.ENSP00000333657,9606.ENSP00000343023,9606.ENSP00000354394,9606.ENSP00000357459,9606.ENSP00000360869,9606.ENSP00000360883,9606.ENSP00000365402,9606.ENSP00000369299,9606.ENSP00000374455,9606.ENSP00000379873,9606.ENSP00000381601,9606.ENSP00000382707,9606.ENSP00000388001,9606.ENSP00000395590,9606.ENSP00000398632,9606.ENSP00000399168,9606.ENSP00000433138	OAS3,OASL,PML,STAT2,DUOX1,MX2,SP100,STAT1,ADAR,IFIT1,IFIT3,HLA-C,TRIM22,SQSTM1,HLA-A,MX1,IFITM3,OAS1,IFI35,CD44,HLA-B,CASP1
GO:0071346	cellular response to interferon-gamma	14	155	1.41	6.52E-14	9606.ENSP00000228928,9606.ENSP00000257570,9606.ENSP00000268058,9606.ENSP00000343023,9606.ENSP00000354394,9606.ENSP00000359490,9606.ENSP00000365402,9606.ENSP00000369299,9606.ENSP00000378856,9606.ENSP00000379873,9606.ENSP00000388001,9606.ENSP00000398632,9606.ENSP00000399168,9606.ENSP00000433138	OAS3,OASL,PML,SP100,STAT1,GBP4,HLA-C,TRIM22,LGALS9,HLA-A,OAS1,CD44,HLA-B,CASP1
GO:0043903	regulation of symbiosis, encompassing mutualism through parasitism	15	203	1.33	8.22E-14	9606.ENSP00000228928,9606.ENSP00000233057,9606.ENSP00000253110,9606.ENSP00000257570,9606.ENSP00000268058,9606.ENSP00000325618,9606.ENSP00000343023,9606.ENSP00000354394,9606.ENSP00000357459,9606.ENSP00000360869,9606.ENSP00000374455,9606.ENSP00000378856,9606.ENSP00000381601,9606.ENSP00000382707,9606.ENSP00000388001	OAS3,EIF2AK2,C19orf66,OASL,PML,PARP10,SP100,STAT1,ADAR,IFIT1,SQSTM1,LGALS9,MX1,IFITM3,OAS1
GO:0050792	regulation of viral process	14	174	1.36	2.61E-13	9606.ENSP00000228928,9606.ENSP00000233057,9606.ENSP00000253110,9606.ENSP00000257570,9606.ENSP00000268058,9606.ENSP00000325618,9606.ENSP00000343023,9606.ENSP00000354394,9606.ENSP00000357459,9606.ENSP00000360869,9606.ENSP00000378856,9606.ENSP00000381601,9606.ENSP00000382707,9606.ENSP00000388001	OAS3,EIF2AK2,C19orf66,OASL,PML,PARP10,SP100,STAT1,ADAR,IFIT1,LGALS9,MX1,IFITM3,OAS1
GO:0060333	interferon-gamma-mediated signaling pathway	11	69	1.66	3.03E-13	9606.ENSP00000228928,9606.ENSP00000257570,9606.ENSP00000268058,9606.ENSP00000343023,9606.ENSP00000354394,9606.ENSP00000365402,9606.ENSP00000369299,9606.ENSP00000379873,9606.ENSP00000388001,9606.ENSP00000398632,9606.ENSP00000399168	OAS3,OASL,PML,SP100,STAT1,HLA-C,TRIM22,HLA-A,OAS1,CD44,HLA-B

GO:0051704	multi-organism process	36	2514	0.61	3.17E-13	9606.ENSP00000228928,9606.ENSP00000233057,9606.ENSP00000243253,9606.ENSP00000249066,9606.ENSP00000253110,9606.ENSP00000256722,9606.ENSP00000257570,9606.ENSP00000264705,9606.ENSP00000268058,9606.ENSP00000270586,9606.ENSP00000295666,9606.ENSP00000315768,9606.ENSP00000320485,9606.ENSP00000333657,9606.ENSP00000343023,9606.ENSP00000354394,9606.ENSP00000355325,9606.ENSP00000357459,9606.ENSP00000359490,9606.ENSP00000359783,9606.ENSP00000359787,9606.ENSP00000360869,9606.ENSP00000360883,9606.ENSP00000363993,9606.ENSP00000365402,9606.ENSP00000369299,9606.ENSP00000378856,9606.ENSP00000379873,9606.ENSP00000381601,9606.ENSP00000382707,9606.ENSP00000388001,9606.ENSP00000395590,9606.ENSP00000398632,9606.ENSP00000399168,9606.ENSP00000418194,9606.ENSP00000433138	OAS3,EIF2AK2,SEC61A1,APOL2,C19orf66,CMPK2,OASL,CAD,PML,PSMB6,IGFBP7,STAT2,ARID1A,MX2,SP100,STAT1,PSMB5,ADAR,GBP4,IFI44,IFI44L,IFIT1,IFIT3,PSMB9,HLA-C,TRIM22,LGALS9,HLA-A,MX1,IFITM3,OAS1,IFI35,CD44,HLA-B,PARP14,CASP1
GO:0071345	cellular response to cytokine stimulus	24	953	0.86	6.42E-13	9606.ENSP00000228928,9606.ENSP00000257570,9606.ENSP00000268058,9606.ENSP00000315768,9606.ENSP00000317997,9606.ENSP00000333657,9606.ENSP00000343023,9606.ENSP00000354394,9606.ENSP00000357459,9606.ENSP00000359490,9606.ENSP00000360869,9606.ENSP00000360883,9606.ENSP00000365402,9606.ENSP00000369299,9606.ENSP00000374455,9606.ENSP00000378856,9606.ENSP00000379873,9606.ENSP00000381601,9606.ENSP00000382707,9606.ENSP00000388001,9606.ENSP00000395590,9606.ENSP00000398632,9606.ENSP00000399168,9606.ENSP00000433138	OAS3,OASL,PML,STAT2,DUOX1,MX2,SP100,STAT1,ADAR,GBP4,IFIT1,IFIT3,HLA-C,TRIM22,SQSTM1,LGALS9,HLA-A,MX1,IFITM3,OAS1,IFI35,CD44,HLA-B,CASP1
GO:1903901	negative regulation of viral life cycle	11	77	1.61	7.88E-13	9606.ENSP00000228928,9606.ENSP00000233057,9606.ENSP00000253110,9606.ENSP00000257570,9606.ENSP00000268058,9606.ENSP00000325618,9606.ENSP00000357459,9606.ENSP00000360869,9606.ENSP00000381601,9606.ENSP00000382707,9606.ENSP00000388001	OAS3,EIF2AK2,C19orf66,OASL,PML,PARP10,ADAR,IFIT1,MX1,IFITM3,OAS1
GO:0045071	negative regulation of viral genome replication	10	52	1.74	9.16E-13	9606.ENSP00000228928,9606.ENSP00000233057,9606.ENSP00000253110,9606.ENSP00000257570,9606.ENSP00000325618,9606.ENSP00000357459,9606.ENSP00000360869,9606.ENSP00000381601,9606.ENSP00000382707,9606.ENSP00000388001	OAS3,EIF2AK2,C19orf66,OASL,PARP10,ADAR,IFIT1,MX1,IFITM3,OAS1
GO:0009605	response to external stimulus	33	2152	0.64	1.12E-12	9606.ENSP00000228928,9606.ENSP00000233057,9606.ENSP00000243253,9606.ENSP00000252809,9606.ENSP00000253110,9606.ENSP00000256722,9606.ENSP00000257570,9606.ENSP00000264705,9606.ENSP00000268058,9606.ENSP00000315768,9606.ENSP00000333657,9606.ENSP00000343023,9606.ENSP00000354394,9606.ENSP00000357459,9606.ENSP00000359490,9606.ENSP00000359783,9606.ENSP00000359787,9606.ENSP00000360869,9606.ENSP00000360883,9606.ENSP00000365402,9606.ENSP00000369299,9606.ENSP00000378856,9606.ENSP00000379038,9606.ENSP00000379873,9606.ENSP00000381601,9606.ENSP00000382707,9606.ENSP00000388001,9606.ENSP00000395590,9606.ENSP00000398632,9606.ENSP00000399168,9606.ENSP00000418194,9606.ENSP00000433138,9606.ENSP00000457513	OAS3,EIF2AK2,SEC61A1,GDF15,C19orf66,CMPK2,OASL,CAD,PML,STAT2,MX2,SP100,STAT1,ADAR,GBP4,IFI44,IFI44L,IFIT1,IFIT3,HLA-C,TRIM22,LGALS9,TYMP,HLA-A,MX1,IFITM3,OAS1,IFI35,CD44,HLA-B,PARP14,CASP1,COX4I1
GO:1903900	regulation of viral life cycle	12	135	1.41	7.29E-12	9606.ENSP00000228928,9606.ENSP00000233057,9606.ENSP00000253110,9606.ENSP00000257570,9606.ENSP00000268058,9606.ENSP00000325618,9606.ENSP00000357459,9606.ENSP00000360869,9606.ENSP00000378856,9606.ENSP00000381601,9606.ENSP00000382707,9606.ENSP00000388001	OAS3,EIF2AK2,C19orf66,OASL,PML,PARP10,ADAR,IFIT1,LGALS9,MX1,IFITM3,OAS1

GO:0071310	cellular response to organic substance	31	2219	0.6	1.15E-10	9606.ENSP00000228928,9606.ENSP00000233057,9606.ENSP00000252809,9606.ENSP00000256722,9606.ENSP00000257570,9606.ENSP00000264705,9606.ENSP00000268058,9606.ENSP00000295666,9606.ENSP00000315768,9606.ENSP00000317997,9606.ENSP00000320485,9606.ENSP00000333657,9606.ENSP00000343023,9606.ENSP00000354394,9606.ENSP00000357459,9606.ENSP00000359490,9606.ENSP00000360869,9606.ENSP00000360883,9606.ENSP00000365402,9606.ENSP00000368678,9606.ENSP00000369299,9606.ENSP00000374455,9606.ENSP00000378856,9606.ENSP00000379873,9606.ENSP00000381601,9606.ENSP00000382707,9606.ENSP00000388001,9606.ENSP00000395590,9606.ENSP00000398632,9606.ENSP00000399168,9606.ENSP00000433138	OAS3,EIF2AK2,GDF15,CMPK2,OASL,CAD,PML,IGFBP7,STAT2,DUOX1,ARID1A,MX2,SP100,STAT1,ADAR,GBP4,IFIT1,IFIT3,HLA-C,AGRN,TRIM22,SQSTM1,LGALS9,HLA-A,MX1,IFITM3,OAS1,IFI35,CD44,HLA-B,CASP1
GO:0010033	response to organic substance	33	2815	0.53	1.63E-09	9606.ENSP00000228928,9606.ENSP00000233057,9606.ENSP00000243253,9606.ENSP00000252809,9606.ENSP00000253110,9606.ENSP00000256722,9606.ENSP00000257570,9606.ENSP00000264705,9606.ENSP00000268058,9606.ENSP00000295666,9606.ENSP00000315768,9606.ENSP00000317997,9606.ENSP00000320485,9606.ENSP00000333657,9606.ENSP00000343023,9606.ENSP00000354394,9606.ENSP00000357459,9606.ENSP00000359490,9606.ENSP00000360869,9606.ENSP00000360883,9606.ENSP00000365402,9606.ENSP00000368678,9606.ENSP00000369299,9606.ENSP00000374455,9606.ENSP00000378856,9606.ENSP00000379873,9606.ENSP00000381601,9606.ENSP00000382707,9606.ENSP00000388001,9606.ENSP00000395590,9606.ENSP00000398632,9606.ENSP00000399168,9606.ENSP00000433138	OAS3,EIF2AK2,SEC61A1,GDF15,C19orf66,CMPK2,OASL,CAD,PML,IGFBP7,STAT2,DUOX1,ARID1A,MX2,SP100,STAT1,ADAR,GBP4,IFIT1,IFIT3,HLA-C,AGRN,TRIM22,SQSTM1,LGALS9,HLA-A,MX1,IFITM3,OAS1,IFI35,CD44,HLA-B,CASP1
GO:0002252	immune effector process	20	927	0.79	2.05E-09	9606.ENSP00000228928,9606.ENSP00000233057,9606.ENSP00000253110,9606.ENSP00000257570,9606.ENSP00000268058,9606.ENSP00000315768,9606.ENSP00000333657,9606.ENSP00000354394,9606.ENSP00000357459,9606.ENSP00000359787,9606.ENSP00000360869,9606.ENSP00000360883,9606.ENSP00000365402,9606.ENSP00000369299,9606.ENSP00000379873,9606.ENSP00000381601,9606.ENSP00000382707,9606.ENSP00000388001,9606.ENSP00000398632,9606.ENSP00000399168	OAS3,EIF2AK2,C19orf66,OASL,PML,STAT2,MX2,STAT1,ADAR,IFI44L,IFIT1,IFIT3,HLA-C,TRIM22,HLA-A,MX1,IFITM3,OAS1,CD44,HLA-B
GO:0006950	response to stress	34	3267	0.48	1.61E-08	9606.ENSP00000228928,9606.ENSP00000233057,9606.ENSP00000243253,9606.ENSP00000249066,9606.ENSP00000253110,9606.ENSP00000257570,9606.ENSP00000262776,9606.ENSP00000264705,9606.ENSP00000268058,9606.ENSP00000295666,9606.ENSP00000315768,9606.ENSP00000317997,9606.ENSP00000333657,9606.ENSP00000343023,9606.ENSP00000354394,9606.ENSP00000355325,9606.ENSP00000357459,9606.ENSP00000359490,9606.ENSP00000359787,9606.ENSP00000360869,9606.ENSP00000360883,9606.ENSP00000365402,9606.ENSP00000369299,9606.ENSP00000374455,9606.ENSP00000378856,9606.ENSP00000379873,9606.ENSP00000381601,9606.ENSP00000382707,9606.ENSP00000388001,9606.ENSP00000395590,9606.ENSP00000398632,9606.ENSP00000399168,9606.ENSP00000418194,9606.ENSP00000433138	OAS3,EIF2AK2,SEC61A1,APOL2,C19orf66,OASL,LGALS3BP,CAD,PML,IGFBP7,STAT2,DUOX1,MX2,SP100,STAT1,PSMB5,ADAR,GBP4,IFI44L,IFIT1,IFIT3,HLA-C,TRIM22,SQSTM1,LGALS9,HLA-A,MX1,IFITM3,OAS1,IFI35,CD44,HLA-B,PARP14,CASP1

GO:0002376	immune system process	29	2370	0.55	1.70E-08	9606.ENSP00000228928,9606.ENSP00000233057,9606.ENSP00000243253,9606.ENSP00000253110,9606.ENSP00000257570,9606.ENSP00000268058,9606.ENSP00000315768,9606.ENSP00000333657,9606.ENSP00000343023,9606.ENSP00000354394,9606.ENSP00000357459,9606.ENSP00000359490,9606.ENSP00000359787,9606.ENSP00000360869,9606.ENSP00000360883,9606.ENSP00000363993,9606.ENSP00000365402,9606.ENSP00000369299,9606.ENSP00000374455,9606.ENSP00000378856,9606.ENSP00000379873,9606.ENSP00000381601,9606.ENSP00000382707,9606.ENSP00000388001,9606.ENSP00000395590,9606.ENSP00000398632,9606.ENSP00000399168,9606.ENSP00000418194,9606.ENSP00000433138	OAS3,EIF2AK2,SEC61A1,C19orf66,OASL,PML,STAT2,MX2,SP100,STAT1,ADAR,GBP4,IFI44L,IFIT1,IFIT3,PSMB9,HLA-C,TRIM22,SQSTM1,LGALS9,HLA-A,MX1,IFITM3,OAS1,IFI35,CD44,HLA-B,PARP14,CASP1
GO:0035455	response to interferon-alpha	6	22	1.89	2.57E-08	9606.ENSP00000233057,9606.ENSP00000333657,9606.ENSP00000357459,9606.ENSP00000360883,9606.ENSP00000382707,9606.ENSP00000388001	EIF2AK2,MX2,ADAR,IFIT3,IFITM3,OAS1
GO:0042221	response to chemical	35	4153	0.38	2.10E-06	9606.ENSP00000228928,9606.ENSP00000233057,9606.ENSP00000243253,9606.ENSP00000252809,9606.ENSP00000253110,9606.ENSP00000256722,9606.ENSP00000257570,9606.ENSP00000264705,9606.ENSP00000268058,9606.ENSP00000295666,9606.ENSP00000315768,9606.ENSP00000317997,9606.ENSP00000320485,9606.ENSP00000333657,9606.ENSP00000343023,9606.ENSP00000354394,9606.ENSP00000357459,9606.ENSP00000359490,9606.ENSP00000360869,9606.ENSP00000360883,9606.ENSP00000365402,9606.ENSP00000368678,9606.ENSP00000369299,9606.ENSP00000374455,9606.ENSP00000378856,9606.ENSP00000379038,9606.ENSP00000379873,9606.ENSP00000381601,9606.ENSP00000382707,9606.ENSP00000388001,9606.ENSP00000395590,9606.ENSP00000398632,9606.ENSP00000399168,9606.ENSP00000433138,9606.ENSP00000457513	OAS3,EIF2AK2,SEC61A1,GDF15,C19orf66,CMPK2,OASL,CAD,PML,IGFBP7,STAT2,DUOX1,ARID1A,MX2,SP100,STAT1,ADAR,GBP4,IFIT1,IFIT3,HLA-C,AGRN,TRIM22,SQSTM1,LGALS9,TYMP,HLA-A,MX1,IFITM3,OAS1,IFI35,CD44,HLA-B,CASP1,COX4I1
GO:0016032	viral process	13	571	0.82	4.36E-06	9606.ENSP00000233057,9606.ENSP00000268058,9606.ENSP00000270586,9606.ENSP00000315768,9606.ENSP00000343023,9606.ENSP00000354394,9606.ENSP00000355325,9606.ENSP00000360869,9606.ENSP00000363993,9606.ENSP00000365402,9606.ENSP00000369299,9606.ENSP00000379873,9606.ENSP00000399168	EIF2AK2,PML,PSMB6,STAT2,SP100,STAT1,PSMB5,IFIT1,PSMB9,HLA-C,TRIM22,HLA-A,HLA-B
GO:0007166	cell surface receptor signaling pathway	24	2198	0.5	8.67E-06	9606.ENSP00000228928,9606.ENSP00000252809,9606.ENSP00000257570,9606.ENSP00000268058,9606.ENSP00000315768,9606.ENSP00000317997,9606.ENSP00000322885,9606.ENSP00000333657,9606.ENSP00000343023,9606.ENSP00000354394,9606.ENSP00000357459,9606.ENSP00000360869,9606.ENSP00000360883,9606.ENSP00000365402,9606.ENSP00000369299,9606.ENSP00000374455,9606.ENSP00000379873,9606.ENSP00000381601,9606.ENSP00000382707,9606.ENSP00000388001,9606.ENSP00000395590,9606.ENSP00000398632,9606.ENSP00000399168,9606.ENSP00000433138	OAS3,GDF15,OASL,PML,STAT2,DUOX1,DTX2,MX2,SP100,STAT1,ADAR,IFIT1,IFIT3,HLA-C,TRIM22,SQSTM1,HLA-A,MX1,IFITM3,OAS1,IFI35,CD44,HLA-B,CASP1
GO:0002832	negative regulation of response to biotic stimulus	6	85	1.31	3.37E-05	9606.ENSP00000357459,9606.ENSP00000360869,9606.ENSP00000378856,9606.ENSP00000379873,9606.ENSP00000399168,9606.ENSP00000418194	ADAR,IFIT1,LGALS9,HLA-A,HLA-B,PARP14
GO:0002486	antigen processing and presentation of endogenous peptide antigen via MHC class I via ER pathway, TAP-independent	3	3	2.46	3.56E-05	9606.ENSP00000365402,9606.ENSP00000379873,9606.ENSP00000399168	HLA-C,HLA-A,HLA-B
GO:0045824	negative regulation of innate immune response	5	49	1.47	5.12E-05	9606.ENSP00000357459,9606.ENSP00000378856,9606.ENSP00000379873,9606.ENSP00000399168,9606.ENSP00000418194	ADAR,LGALS9,HLA-A,HLA-B,PARP14

GO:0050896	response to stimulus	46	7824	0.23	1.60E-04	9606.ENSP00000228928,9606.ENSP00000233057,9606.ENSP00000243253,9606.ENSP00000249066,9606.ENSP00000252809,9606.ENSP00000253110,9606.ENSP00000256722,9606.ENSP00000257570,9606.ENSP00000262776,9606.ENSP00000264705,9606.ENSP00000268058,9606.ENSP00000290291,9606.ENSP00000295666,9606.ENSP00000315768,9606.ENSP00000317997,9606.ENSP00000320485,9606.ENSP00000322885,9606.ENSP00000333657,9606.ENSP00000337641,9606.ENSP00000343023,9606.ENSP00000354394,9606.ENSP00000355325,9606.ENSP00000355330,9606.ENSP00000357459,9606.ENSP00000357709,9606.ENSP00000359490,9606.ENSP00000359783,9606.ENSP00000359787,9606.ENSP00000360869,9606.ENSP00000360883,9606.ENSP00000365402,9606.ENSP00000368678,9606.ENSP00000369299,9606.ENSP00000374455,9606.ENSP00000378856,9606.ENSP00000379038,9606.ENSP00000379873,9606.ENSP00000381601,9606.ENSP00000382707,9606.ENSP00000388001,9606.ENSP00000395590,9606.ENSP00000398632,9606.ENSP00000399168,9606.ENSP00000418194,9606.ENSP00000433138,9606.ENSP00000457513	OAS3,EIF2AK2,SEC61A1,APOL2,GDF15,C19orf66,CMPK2,OASL,LGALS3BP,CAD,PML,OGFR,IGFBP7,STAT2,DUOX1,ARID1A,DTX2,MX2,PPP2R5E,SP100,STAT1,PSMB5,TGM2,ADAR,S100A6,GBP4,IFI44,IFI44L,IFIT1,IFIT3,HLA-C,AGRN,TRIM22,SQSTM1,LGALS9,TYMP,HLA-A,MX1,IFITM3,OAS1,IFI35,CD44,HLA-B,PARP14,CASP1,COX4I1
GO:0002480	antigen processing and presentation of exogenous peptide antigen via MHC class I, TAP-independent	3	8	2.03	2.60E-04	9606.ENSP00000365402,9606.ENSP00000379873,9606.ENSP00000399168	HLA-C,HLA-A,HLA-B
GO:0007165	signal transduction	33	4738	0.3	4.60E-04	9606.ENSP00000228928,9606.ENSP00000233057,9606.ENSP00000252809,9606.ENSP00000257570,9606.ENSP00000262776,9606.ENSP00000268058,9606.ENSP00000290291,9606.ENSP00000315768,9606.ENSP00000317997,9606.ENSP00000320485,9606.ENSP00000322885,9606.ENSP00000333657,9606.ENSP00000337641,9606.ENSP00000343023,9606.ENSP00000354394,9606.ENSP00000355330,9606.ENSP00000357459,9606.ENSP00000357709,9606.ENSP00000360869,9606.ENSP00000360883,9606.ENSP00000365402,9606.ENSP00000368678,9606.ENSP00000369299,9606.ENSP00000374455,9606.ENSP00000378856,9606.ENSP00000379873,9606.ENSP00000381601,9606.ENSP00000382707,9606.ENSP00000388001,9606.ENSP00000395590,9606.ENSP00000398632,9606.ENSP00000399168,9606.ENSP00000433138	OAS3,EIF2AK2,GDF15,OASL,LGALS3BP,PML,OGFR,STAT2,DUOX1,ARID1A,DTX2,MX2,PPP2R5E,SP100,STAT1,TGM2,ADAR,S100A6,IFIT1,IFIT3,HLA-C,AGRN,TRIM22,SQSTM1,LGALS9,HLA-A,MX1,IFITM3,OAS1,IFI35,CD44,HLA-B,CASP1
GO:0045953	negative regulation of natural killer cell mediated cytotoxicity	3	13	1.82	8.00E-04	9606.ENSP00000378856,9606.ENSP00000379873,9606.ENSP00000399168	LGALS9,HLA-A,HLA-B
GO:0031348	negative regulation of defense response	6	180	0.98	1.50E-03	9606.ENSP00000357459,9606.ENSP00000360869,9606.ENSP00000378856,9606.ENSP00000379873,9606.ENSP00000399168,9606.ENSP00000418194	ADAR,IFIT1,LGALS9,HLA-A,HLA-B,PARP14

GO:0051716	cellular response to stimulus	37	6212	0.23	3.10E-03	9606.ENSP00000228928,9606.ENSP00000233057,9606.ENSP00000252809,9606.ENSP00000256722,9606.ENSP00000257570,9606.ENSP00000262776,9606.ENSP00000264705,9606.ENSP00000268058,9606.ENSP00000290291,9606.ENSP00000295666,9606.ENSP00000315768,9606.ENSP00000317997,9606.ENSP00000320485,9606.ENSP00000322885,9606.ENSP00000333657,9606.ENSP00000337641,9606.ENSP00000343023,9606.ENSP00000354394,9606.ENSP00000355330,9606.ENSP00000357459,9606.ENSP00000357709,9606.ENSP00000359490,9606.ENSP00000360869,9606.ENSP00000360883,9606.ENSP00000365402,9606.ENSP00000368678,9606.ENSP00000369299,9606.ENSP00000374455,9606.ENSP00000378856,9606.ENSP00000379873,9606.ENSP00000381601,9606.ENSP00000382707,9606.ENSP00000388001,9606.ENSP00000395590,9606.ENSP00000398632,9606.ENSP00000399168,9606.ENSP00000433138	OAS3,EIF2AK2,GDF15,CMPK2,OASL,LGALS3BP,CAD,PML,OGFR,IGFBP7,STAT2,DUOX1,ARID1A,DTX2,MX2,PPP2R5E,SP100,STAT1,TGM2,ADAR,S100A6,GBP4,IFIT1,IFIT3,HLA-C,AGRN,TRIM22,SQSTM1,LGALS9,HLA-A,MX1,IFITM3,OAS1,IFI35,CD44,HLA-B,CASP1
GO:0031347	regulation of defense response	10	676	0.63	3.70E-03	9606.ENSP00000268058,9606.ENSP00000354394,9606.ENSP00000355330,9606.ENSP00000357459,9606.ENSP00000360869,9606.ENSP00000378856,9606.ENSP00000379873,9606.ENSP00000399168,9606.ENSP00000418194,9606.ENSP00000433138	PML,STAT1,TGM2,ADAR,IFIT1,LGALS9,HLA-A,HLA-B,PARP14,CASP1
GO:0002479	antigen processing and presentation of exogenous peptide antigen via MHC class I, TAP-dependent	3	25	1.54	3.80E-03	9606.ENSP00000365402,9606.ENSP00000379873,9606.ENSP00000399168	HLA-C,HLA-A,HLA-B
GO:0002831	regulation of response to biotic stimulus	8	426	0.73	3.80E-03	9606.ENSP00000268058,9606.ENSP00000354394,9606.ENSP00000357459,9606.ENSP00000360869,9606.ENSP00000378856,9606.ENSP00000379873,9606.ENSP00000399168,9606.ENSP00000418194	PML,STAT1,ADAR,IFIT1,LGALS9,HLA-A,HLA-B,PARP14
GO:0042270	protection from natural killer cell mediated cytotoxicity	2	5	2.06	7.20E-03	9606.ENSP00000379873,9606.ENSP00000399168	HLA-A,HLA-B
GO:0051414	response to cortisol	2	5	2.06	7.20E-03	9606.ENSP00000264705,9606.ENSP00000295666	CAD,IGFBP7
GO:0070212	protein poly-ADP-ribosylation	2	5	2.06	7.20E-03	9606.ENSP00000325618,9606.ENSP00000418194	PARP10,PARP14
GO:0043122	regulation of I-kappaB kinase/NF-kappaB signaling	5	167	0.94	9.00E-03	9606.ENSP00000354394,9606.ENSP00000355330,9606.ENSP00000369299,9606.ENSP00000374455,9606.ENSP00000433138	STAT1,TGM2,TRIM22,SQSTM1,CASP1
GO:0050706	regulation of interleukin-1 beta secretion	3	37	1.37	9.80E-03	9606.ENSP00000268058,9606.ENSP00000378856,9606.ENSP00000433138	PML,LGALS9,CASP1
GO:0003374	dynamin family protein polymerization involved in mitochondrial fission	2	7	1.91	1.09E-02	9606.ENSP00000333657,9606.ENSP00000381601	MX2,MX1
GO:0050691	regulation of defense response to virus by host	3	39	1.35	1.09E-02	9606.ENSP00000268058,9606.ENSP00000354394,9606.ENSP00000360869	PML,STAT1,IFIT1
GO:0060700	regulation of ribonuclease activity	2	7	1.91	1.09E-02	9606.ENSP00000228928,9606.ENSP00000388001	OAS3,OAS1
GO:0007565	female pregnancy	5	180	0.9	1.12E-02	9606.ENSP00000249066,9606.ENSP00000264705,9606.ENSP00000295666,9606.ENSP00000320485,9606.ENSP00000378856	APOL2,CAD,IGFBP7,ARID1A,LGALS9
GO:0080134	regulation of response to stress	13	1299	0.46	1.19E-02	9606.ENSP00000233057,9606.ENSP00000261517,9606.ENSP00000268058,9606.ENSP00000354394,9606.ENSP00000355330,9606.ENSP00000357459,9606.ENSP00000360869,9606.ENSP00000378856,9606.ENSP00000379873,9606.ENSP00000398632,9606.ENSP00000399168,9606.ENSP00000418194,9606.ENSP00000433138	EIF2AK2,VPS13C,PML,STAT1,TGM2,ADAR,IFIT1,LGALS9,HLA-A,CD44,HLA-B,PARP14,CASP1
GO:0046719	regulation by virus of viral protein levels in host cell	2	8	1.86	1.24E-02	9606.ENSP00000354394,9606.ENSP00000360869	STAT1,IFIT1
GO:0002698	negative regulation of immune effector process	4	113	1.01	1.68E-02	9606.ENSP00000360869,9606.ENSP00000378856,9606.ENSP00000379873,9606.ENSP00000399168	IFIT1,LGALS9,HLA-A,HLA-B

GO:0032102	negative regulation of response to external stimulus	6	310	0.75	1.77E-02	9606.ENSP00000357459,9606.ENSP00000360869,9606.ENSP00000378856,9606.ENSP00000379873,9606.ENSP00000399168,9606.ENSP00000418194	ADAR,IFIT1,LGALS9,HLA-A,HLA-B,PARP14
GO:2001198	regulation of dendritic cell differentiation	2	11	1.72	2.00E-02	9606.ENSP00000378856,9606.ENSP00000399168	LGALS9,HLA-B
GO:0072528	pyrimidine-containing compound biosynthetic process	3	54	1.2	2.21E-02	9606.ENSP00000256722,9606.ENSP00000264705,9606.ENSP00000379038	CMPK2,CAD,TYMP
GO:0035457	cellular response to interferon-alpha	2	12	1.68	2.25E-02	9606.ENSP00000360883,9606.ENSP00000388001	IFIT3,OAS1
GO:1903902	positive regulation of viral life cycle	3	59	1.17	2.70E-02	9606.ENSP00000357459,9606.ENSP00000360869,9606.ENSP00000378856	ADAR,IFIT1,LGALS9
GO:0048519	negative regulation of biological process	29	4953	0.23	2.82E-02	9606.ENSP00000228928,9606.ENSP00000233057,9606.ENSP00000252809,9606.ENSP00000253110,9606.ENSP00000257570,9606.ENSP00000261517,9606.ENSP00000268058,9606.ENSP00000295666,9606.ENSP00000320485,9606.ENSP00000325618,9606.ENSP00000343023,9606.ENSP00000347495,9606.ENSP00000351811,9606.ENSP00000354394,9606.ENSP00000357459,9606.ENSP00000360869,9606.ENSP00000360883,9606.ENSP00000369299,9606.ENSP00000374455,9606.ENSP00000378856,9606.ENSP00000379873,9606.ENSP00000381601,9606.ENSP00000382707,9606.ENSP00000388001,9606.ENSP00000398632,9606.ENSP00000399168,9606.ENSP00000418194,9606.ENSP00000464087,9606.ENSP00000475814	OAS3,EIF2AK2,GDF15,C19orf66,OASL,VPS13C,PML,IGFBP7,ARID1A,PARP10,SP100,WARS,EDC4,STAT1,ADAR,IFIT1,IFIT3,TRIM22,SQSTM1,LGALS9,HLA-A,MX1,IFITM3,OAS1,CD44,HLA-B,PARP14,RNF213,HIST2H2AA3
GO:0016045	detection of bacterium	2	15	1.58	3.13E-02	9606.ENSP00000379873,9606.ENSP00000399168	HLA-A,HLA-B
GO:0002697	regulation of immune effector process	6	362	0.68	3.36E-02	9606.ENSP00000268058,9606.ENSP00000354394,9606.ENSP00000360869,9606.ENSP00000378856,9606.ENSP00000379873,9606.ENSP00000399168	PML,STAT1,IFIT1,LGALS9,HLA-A,HLA-B
GO:0032101	regulation of response to external stimulus	10	955	0.48	3.36E-02	9606.ENSP00000268058,9606.ENSP00000354394,9606.ENSP00000355330,9606.ENSP00000357459,9606.ENSP00000360869,9606.ENSP00000378856,9606.ENSP00000379873,9606.ENSP00000399168,9606.ENSP00000418194,9606.ENSP00000433138	PML,STAT1,TGM2,ADAR,IFIT1,LGALS9,HLA-A,HLA-B,PARP14,CASP1
GO:0045088	regulation of innate immune response	6	361	0.68	3.36E-02	9606.ENSP00000354394,9606.ENSP00000357459,9606.ENSP00000378856,9606.ENSP00000379873,9606.ENSP00000399168,9606.ENSP00000418194	STAT1,ADAR,LGALS9,HLA-A,HLA-B,PARP14
GO:2000116	regulation of cysteine-type endopeptidase activity	5	243	0.77	3.36E-02	9606.ENSP00000268058,9606.ENSP00000363993,9606.ENSP00000378856,9606.ENSP00000398632,9606.ENSP00000433138	PML,PSMB9,LGALS9,CD44,CASP1
GO:0001916	positive regulation of T cell mediated cytotoxicity	2	17	1.53	3.68E-02	9606.ENSP00000379873,9606.ENSP00000399168	HLA-A,HLA-B
GO:0048384	retinoic acid receptor signaling pathway	2	17	1.53	3.68E-02	9606.ENSP00000268058,9606.ENSP00000343023	PML,SP100
GO:0001959	regulation of cytokine-mediated signaling pathway	4	151	0.88	3.82E-02	9606.ENSP00000354394,9606.ENSP00000357459,9606.ENSP00000418194,9606.ENSP00000433138	STAT1,ADAR,PARP14,CASP1
GO:0014074	response to purine-containing compound	4	151	0.88	3.82E-02	9606.ENSP00000264705,9606.ENSP00000317997,9606.ENSP00000354394,9606.ENSP00000433138	CAD,DUOX1,STAT1,CASP1
GO:0006206	pyrimidine nucleobase metabolic process	2	18	1.5	3.89E-02	9606.ENSP00000264705,9606.ENSP00000379038	CAD,TYMP
GO:0043555	regulation of translation in response to stress	2	18	1.5	3.89E-02	9606.ENSP00000233057,9606.ENSP00000268058	EIF2AK2,PML
GO:0046641	positive regulation of alpha-beta T cell proliferation	2	18	1.5	3.89E-02	9606.ENSP00000378856,9606.ENSP00000379873	LGALS9,HLA-A

GO:0008285	negative regulation of cell population proliferation	8	669	0.54	3.98E-02	9606.ENSP00000233057,9606.ENSP00000268058,9606.ENSP00000295666,9606.ENSP00000325618,9606.ENSP00000347495,9606.ENSP00000354394,9606.ENSP00000360883,9606.ENSP00000378856	EIF2AK2,PML,IGFBP7,PARP10,WARS,STAT1,IFIT3,LGALS9
GO:0000266	mitochondrial fission	2	19	1.48	4.06E-02	9606.ENSP00000333657,9606.ENSP00000381601	MX2,MX1
GO:0009148	pyrimidine nucleoside triphosphate biosynthetic process	2	19	1.48	4.06E-02	9606.ENSP00000256722,9606.ENSP00000264705	CMPK2,CAD

T = 72 h (CoV-2), IFNα7 / Untreated

#Term ID	Term Description	Observed Gene Count	Background Gene Count	Strength	False Discovery Rate	Matching Proteins in Your Input (IDs)	Matching Proteins in Your Input (Labels)
GO:0060337	type I interferon signaling pathway	12	65	1.71	1.03E-13	9606.ENSP00000315768,9606.ENSP00000333657,9606.ENSP00000343023,9606.ENSP00000354394,9606.ENSP00000360869,9606.ENSP00000360883,9606.ENSP00000365402,9606.ENSP00000379873,9606.ENSP00000381601,9606.ENSP00000388001,9606.ENSP00000395590,9606.ENSP00000399168	STAT2,MX2,SP100,STAT1,IFIT1,IFIT3,HLA-C,HLA-A,MX1,OAS1,IFI35,HLA-B
GO:0051707	response to other organism	24	1173	0.75	8.26E-10	9606.ENSP00000233057,9606.ENSP00000256722,9606.ENSP00000269701,9606.ENSP00000304236,9606.ENSP00000310275,9606.ENSP00000315768,9606.ENSP00000333657,9606.ENSP00000343023,9606.ENSP00000349436,9606.ENSP00000354394,9606.ENSP00000359490,9606.ENSP00000359783,9606.ENSP00000359787,9606.ENSP00000360869,9606.ENSP00000360883,9606.ENSP00000365402,9606.ENSP00000377470,9606.ENSP00000379873,9606.ENSP00000381601,9606.ENSP00000388001,9606.ENSP00000395590,9606.ENSP00000399168,9606.ENSP00000399888,9606.ENSP00000418194	EIF2AK2,CMPK2,AKAP8,CD14,BANF1,STAT2,MX2,SP100,ADAM15,STAT1,GBP4,IFI44,IFI44L,IFIT1,IFIT3,HLA-C,CNP,HLA-A,MX1,OAS1,IFI35,HLA-B,HMGA1,PARP14
GO:0045087	innate immune response	18	676	0.87	8.22E-09	9606.ENSP00000233057,9606.ENSP00000269701,9606.ENSP00000304236,9606.ENSP00000315768,9606.ENSP00000333657,9606.ENSP00000343023,9606.ENSP00000349436,9606.ENSP00000354394,9606.ENSP00000359490,9606.ENSP00000360869,9606.ENSP00000360883,9606.ENSP00000365402,9606.ENSP00000379873,9606.ENSP00000381601,9606.ENSP00000388001,9606.ENSP00000395590,9606.ENSP00000399168,9606.ENSP00000418194	EIF2AK2,AKAP8,CD14,STAT2,MX2,SP100,ADAM15,STAT1,GBP4,IFIT1,IFIT3,HLA-C,HLA-A,MX1,OAS1,IFI35,HLA-B,PARP14
GO:0098542	defense response to other organism	19	859	0.79	4.05E-08	9606.ENSP00000233057,9606.ENSP00000269701,9606.ENSP00000304236,9606.ENSP00000315768,9606.ENSP00000333657,9606.ENSP00000343023,9606.ENSP00000349436,9606.ENSP00000354394,9606.ENSP00000359490,9606.ENSP00000359787,9606.ENSP00000360869,9606.ENSP00000360883,9606.ENSP00000365402,9606.ENSP00000379873,9606.ENSP00000381601,9606.ENSP00000388001,9606.ENSP00000395590,9606.ENSP00000399168,9606.ENSP00000418194	EIF2AK2,AKAP8,CD14,STAT2,MX2,SP100,ADAM15,STAT1,GBP4,IFI44L,IFIT1,IFIT3,HLA-C,HLA-A,MX1,OAS1,IFI35,HLA-B,PARP14
GO:0009615	response to virus	12	270	1.09	7.32E-08	9606.ENSP00000233057,9606.ENSP00000310275,9606.ENSP00000315768,9606.ENSP00000333657,9606.ENSP00000354394,9606.ENSP00000359783,9606.ENSP00000359787,9606.ENSP00000360869,9606.ENSP00000360883,9606.ENSP00000381601,9606.ENSP00000388001,9606.ENSP00000399888	EIF2AK2,BANF1,STAT2,MX2,STAT1,IFI44,IFI44L,IFIT1,IFIT3,MX1,OAS1,HMGA1

GO:0009605	response to external stimulus	28	2152	0.55	1.09E-07	9606.ENSP00000233057,9606.ENSP00000256722,9606.ENSP00000269701,9606.ENSP00000304236,9606.ENSP00000310275,9606.ENSP00000315768,9606.ENSP00000333657,9606.ENSP00000343023,9606.ENSP00000349436,9606.ENSP00000354394,9606.ENSP00000359490,9606.ENSP00000359783,9606.ENSP00000359787,9606.ENSP00000360869,9606.ENSP00000360883,9606.ENSP00000365402,9606.ENSP00000369927,9606.ENSP00000377470,9606.ENSP00000379038,9606.ENSP00000379873,9606.ENSP00000381601,9606.ENSP00000385205,9606.ENSP00000388001,9606.ENSP00000395590,9606.ENSP00000399168,9606.ENSP00000399888,9606.ENSP00000418194,9606.ENSP00000467141	EIF2AK2,CMPK2,AKAP8,CD14,BANF1,STAT2,MX2,SP100,ADAM15,STAT1,GBP4,IFI44,IFI44L,IFIT1,IFIT3,HLA-C,AKR1C3,CNP,TYMP,HLA-A,MX1,PICK1,OAS1,IFI35,HLA-B,HMGA1,PARP14,TTN
GO:0043901	negative regulation of multi-organism process	10	212	1.11	1.15E-06	9606.ENSP00000233057,9606.ENSP00000310275,9606.ENSP00000343023,9606.ENSP00000354394,9606.ENSP00000360869,9606.ENSP00000379873,9606.ENSP00000381601,9606.ENSP00000388001,9606.ENSP00000399168,9606.ENSP00000418194	EIF2AK2,BANF1,SP100,STAT1,IFIT1,HLA-A,MX1,OAS1,HLA-B,PARP14
GO:0051607	defense response to virus	9	181	1.14	4.09E-06	9606.ENSP00000233057,9606.ENSP00000315768,9606.ENSP00000333657,9606.ENSP00000354394,9606.ENSP00000359787,9606.ENSP00000360869,9606.ENSP00000360883,9606.ENSP00000381601,9606.ENSP00000388001	EIF2AK2,STAT2,MX2,STAT1,IFI44L,IFIT1,IFIT3,MX1,OAS1
GO:0048525	negative regulation of viral process	7	93	1.32	9.38E-06	9606.ENSP00000233057,9606.ENSP00000310275,9606.ENSP00000343023,9606.ENSP00000354394,9606.ENSP00000360869,9606.ENSP00000381601,9606.ENSP00000388001	EIF2AK2,BANF1,SP100,STAT1,IFIT1,MX1,OAS1
GO:0051704	multi-organism process	27	2514	0.47	1.01E-05	9606.ENSP00000233057,9606.ENSP00000256722,9606.ENSP00000263121,9606.ENSP00000269701,9606.ENSP00000304236,9606.ENSP00000310275,9606.ENSP00000315768,9606.ENSP00000320485,9606.ENSP00000333657,9606.ENSP00000343023,9606.ENSP00000349436,9606.ENSP00000354394,9606.ENSP00000359490,9606.ENSP00000359783,9606.ENSP00000359787,9606.ENSP00000360869,9606.ENSP00000360883,9606.ENSP00000365402,9606.ENSP00000377470,9606.ENSP00000379873,9606.ENSP00000381601,9606.ENSP00000385205,9606.ENSP00000388001,9606.ENSP00000395590,9606.ENSP00000399168,9606.ENSP00000399888,9606.ENSP00000418194	EIF2AK2,CMPK2,SMARCB1,AKAP8,CD14,BANF1,STAT2,ARID1A,MX2,SP100,ADAM15,STAT1,GBP4,IFI44,IFI44L,IFIT1,IFIT3,HLA-C,CNP,HLA-A,MX1,PICK1,OAS1,IFI35,HLA-B,HMGA1,PARP14
GO:0050792	regulation of viral process	8	174	1.1	3.34E-05	9606.ENSP00000233057,9606.ENSP00000263121,9606.ENSP00000310275,9606.ENSP00000343023,9606.ENSP00000354394,9606.ENSP00000360869,9606.ENSP00000381601,9606.ENSP00000388001	EIF2AK2,SMARCB1,BANF1,SP100,STAT1,IFIT1,MX1,OAS1
GO:0060333	interferon-gamma-mediated signaling pathway	6	69	1.38	3.34E-05	9606.ENSP00000343023,9606.ENSP00000354394,9606.ENSP00000365402,9606.ENSP00000379873,9606.ENSP00000388001,9606.ENSP00000399168	SP100,STAT1,HLA-C,HLA-A,OAS1,HLA-B
GO:0002486	antigen processing and presentation of endogenous peptide antigen via MHC class I via ER pathway, TAP-independent	3	3	2.44	8.97E-05	9606.ENSP00000365402,9606.ENSP00000379873,9606.ENSP00000399168	HLA-C,HLA-A,HLA-B

GO:0006955	immune response	19	1560	0.53	1.70E-04	9606.ENSP00000233057,9606.ENSP00000269701,9606.ENSP00000271638,9606.ENSP00000304236,9606.ENSP00000315768,9606.ENSP00000333657,9606.ENSP00000343023,9606.ENSP00000349436,9606.ENSP00000354394,9606.ENSP00000359490,9606.ENSP00000360869,9606.ENSP00000360883,9606.ENSP00000365402,9606.ENSP00000379873,9606.ENSP00000381601,9606.ENSP00000388001,9606.ENSP00000395590,9606.ENSP00000399168,9606.ENSP00000418194	EIF2AK2,AKAP8,S100A11,CD14,STAT2,MX2,SP100,ADAM15,STAT1,GBP4,IFIT1,IFIT3,HLA-C,HLA-A,MX1,OAS1,IFI35,HLA-B,PARP14
GO:0035455	response to interferon-alpha	4	22	1.7	1.70E-04	9606.ENSP00000233057,9606.ENSP00000333657,9606.ENSP00000360883,9606.ENSP00000388001	EIF2AK2,MX2,IFIT3,OAS1
GO:0045071	negative regulation of viral genome replication	5	52	1.42	1.70E-04	9606.ENSP00000233057,9606.ENSP00000310275,9606.ENSP00000360869,9606.ENSP00000381601,9606.ENSP00000388001	EIF2AK2,BANF1,IFIT1,MX1,OAS1
GO:0071346	cellular response to interferon-gamma	7	155	1.1	1.70E-04	9606.ENSP00000343023,9606.ENSP00000354394,9606.ENSP00000359490,9606.ENSP00000365402,9606.ENSP00000379873,9606.ENSP00000388001,9606.ENSP00000399168	SP100,STAT1,GBP4,HLA-C,HLA-A,OAS1,HLA-B
GO:0034097	response to cytokine	15	1035	0.6	3.00E-04	9606.ENSP00000233057,9606.ENSP00000304236,9606.ENSP00000315768,9606.ENSP00000333657,9606.ENSP00000343023,9606.ENSP00000354394,9606.ENSP00000359490,9606.ENSP00000360869,9606.ENSP00000360883,9606.ENSP00000365402,9606.ENSP00000379873,9606.ENSP00000381601,9606.ENSP00000388001,9606.ENSP00000395590,9606.ENSP00000399168	EIF2AK2,CD14,STAT2,MX2,SP100,STAT1,GBP4,IFIT1,IFIT3,HLA-C,HLA-A,MX1,OAS1,IFI35,HLA-B
GO:0043900	regulation of multi-organism process	12	653	0.7	3.00E-04	9606.ENSP00000233057,9606.ENSP00000263121,9606.ENSP00000304236,9606.ENSP00000310275,9606.ENSP00000343023,9606.ENSP00000354394,9606.ENSP00000360869,9606.ENSP00000379873,9606.ENSP00000381601,9606.ENSP00000388001,9606.ENSP00000399168,9606.ENSP00000418194	EIF2AK2,SMARCB1,CD14,BANF1,SP100,STAT1,IFIT1,HLA-A,MX1,OAS1,HLA-B,PARP14
GO:0002252	immune effector process	14	927	0.62	3.50E-04	9606.ENSP00000233057,9606.ENSP00000271638,9606.ENSP00000304236,9606.ENSP00000315768,9606.ENSP00000333657,9606.ENSP00000354394,9606.ENSP00000359787,9606.ENSP00000360869,9606.ENSP00000360883,9606.ENSP00000365402,9606.ENSP00000379873,9606.ENSP00000381601,9606.ENSP00000388001,9606.ENSP00000399168	EIF2AK2,S100A11,CD14,STAT2,MX2,STAT1,IFI44L,IFIT1,IFIT3,HLA-C,HLA-A,MX1,OAS1,HLA-B
GO:0002480	antigen processing and presentation of exogenous peptide antigen via MHC class I, TAP-independent	3	8	2.01	4.30E-04	9606.ENSP00000365402,9606.ENSP00000379873,9606.ENSP00000399168	HLA-C,HLA-A,HLA-B
GO:0016032	viral process	11	571	0.73	4.30E-04	9606.ENSP00000233057,9606.ENSP00000263121,9606.ENSP00000310275,9606.ENSP00000315768,9606.ENSP00000343023,9606.ENSP00000354394,9606.ENSP00000360869,9606.ENSP00000365402,9606.ENSP00000379873,9606.ENSP00000399168,9606.ENSP00000399888	EIF2AK2,SMARCB1,BANF1,STAT2,SP100,STAT1,IFIT1,HLA-C,HLA-A,HLA-B,HMGA1

GO:0071345	cellular response to cytokine stimulus	13	953	0.58	1.80E-03	9606.ENSP00000315768,9606.ENSP00000333657,9606.ENSP00000343023,9606.ENSP00000354394,9606.ENSP00000359490,9606.ENSP00000360869,9606.ENSP00000360883,9606.ENSP00000365402,9606.ENSP00000379873,9606.ENSP00000381601,9606.ENSP00000388001,9606.ENSP00000395590,9606.ENSP00000399168	STAT2,MX2,SP100,STAT1,GBP4,IFIT1,IFIT3,HLA-C,HLA-A,MX1,OAS1,IFI35,HLA-B
GO:0006337	nucleosome disassembly	3	18	1.66	2.60E-03	9606.ENSP00000263121,9606.ENSP00000320485,9606.ENSP00000399888	SMARCB1,ARID1A,HMGA1
GO:0002376	immune system process	21	2370	0.39	3.10E-03	9606.ENSP00000233057,9606.ENSP00000269701,9606.ENSP00000271638,9606.ENSP00000304236,9606.ENSP00000315768,9606.ENSP00000333657,9606.ENSP00000343023,9606.ENSP00000349436,9606.ENSP00000354394,9606.ENSP00000359490,9606.ENSP00000359787,9606.ENSP00000360869,9606.ENSP00000360883,9606.ENSP00000365402,9606.ENSP00000367123,9606.ENSP00000379873,9606.ENSP00000381601,9606.ENSP00000388001,9606.ENSP00000395590,9606.ENSP00000399168,9606.ENSP00000418194	EIF2AK2,AKAP8,S100A11,CD14,STAT2,MX2,SP100,ADAM15,STAT1,GBP4,IFI44L,IFIT1,IFIT3,HLA-C,SLC3A2,HLA-A,MX1,OAS1,IFI35,HLA-B,PARP14
GO:0071310	cellular response to organic substance	20	2219	0.4	3.70E-03	9606.ENSP00000233057,9606.ENSP00000256722,9606.ENSP00000269701,9606.ENSP00000304236,9606.ENSP00000315768,9606.ENSP00000320485,9606.ENSP00000333657,9606.ENSP00000343023,9606.ENSP00000349436,9606.ENSP00000354394,9606.ENSP00000359490,9606.ENSP00000360869,9606.ENSP00000360883,9606.ENSP00000365402,9606.ENSP00000369927,9606.ENSP00000379873,9606.ENSP00000381601,9606.ENSP00000388001,9606.ENSP00000395590,9606.ENSP00000399168	EIF2AK2,CMPK2,AKAP8,CD14,STAT2,ARID1A,MX2,SP100,ADAM15,STAT1,GBP4,IFIT1,IFIT3,HLA-C,AKR1C3,HLA-A,MX1,OAS1,IFI35,HLA-B
GO:0010033	response to organic substance	23	2815	0.35	4.10E-03	9606.ENSP00000233057,9606.ENSP00000256722,9606.ENSP00000269701,9606.ENSP00000285737,9606.ENSP00000304236,9606.ENSP00000315768,9606.ENSP00000320485,9606.ENSP00000333657,9606.ENSP00000343023,9606.ENSP00000349436,9606.ENSP00000354394,9606.ENSP00000359490,9606.ENSP00000360869,9606.ENSP00000360883,9606.ENSP00000365402,9606.ENSP00000367123,9606.ENSP00000369927,9606.ENSP00000377470,9606.ENSP00000379873,9606.ENSP00000381601,9606.ENSP00000388001,9606.ENSP00000395590,9606.ENSP00000399168	EIF2AK2,CMPK2,AKAP8,LONP2,CD14,STAT2,ARID1A,MX2,SP100,ADAM15,STAT1,GBP4,IFIT1,IFIT3,HLA-C,SLC3A2,AKR1C3,CNP,HLA-A,MX1,OAS1,IFI35,HLA-B
GO:0002479	antigen processing and presentation of exogenous peptide antigen via MHC class I, TAP-dependent	3	25	1.52	5.40E-03	9606.ENSP00000365402,9606.ENSP00000379873,9606.ENSP00000399168	HLA-C,HLA-A,HLA-B
GO:0042270	protection from natural killer cell mediated cytotoxicity	2	5	2.04	9.80E-03	9606.ENSP00000379873,9606.ENSP00000399168	HLA-A,HLA-B
GO:0002832	negative regulation of response to biotic stimulus	4	85	1.11	1.09E-02	9606.ENSP00000360869,9606.ENSP00000379873,9606.ENSP00000399168,9606.ENSP00000418194	IFIT1,HLA-A,HLA-B,PARP14

GO:0006950	response to stress	24	3267	0.31	1.25E-02	9606.ENSP00000233057,9606.ENSP00000263121,9606.ENSP00000269701,9606.ENSP00000304236,9606.ENSP00000315768,9606.ENSP00000333657,9606.ENSP00000343023,9606.ENSP00000349436,9606.ENSP00000354394,9606.ENSP00000359490,9606.ENSP00000359787,9606.ENSP00000360869,9606.ENSP00000360883,9606.ENSP00000365402,9606.ENSP00000369927,9606.ENSP00000379873,9606.ENSP00000381601,9606.ENSP00000385205,9606.ENSP00000388001,9606.ENSP00000395590,9606.ENSP00000399168,9606.ENSP00000399888,9606.ENSP00000418194,9606.ENSP00000482335	EIF2AK2,SMARCB1,AKAP8,CD14,STAT2,MX2,SP100,ADAM15,STAT1,GBP4,IFI44L,IFIT1,IFIT3,HLA-C,AKR1C3,HLA-A,MX1,PICK1,OAS1,IFI35,HLA-B,HMGA1,PARP14,MAP7
GO:0070887	cellular response to chemical stimulus	21	2672	0.34	1.35E-02	9606.ENSP00000233057,9606.ENSP00000256722,9606.ENSP00000269701,9606.ENSP00000304236,9606.ENSP00000315768,9606.ENSP00000320485,9606.ENSP00000333657,9606.ENSP00000343023,9606.ENSP00000349436,9606.ENSP00000354394,9606.ENSP00000359490,9606.ENSP00000360869,9606.ENSP00000360883,9606.ENSP00000365402,9606.ENSP00000369927,9606.ENSP00000379873,9606.ENSP00000381601,9606.ENSP00000385205,9606.ENSP00000388001,9606.ENSP00000395590,9606.ENSP00000399168	EIF2AK2,CMPK2,AKAP8,CD14,STAT2,ARID1A,MX2,SP100,ADAM15,STAT1,GBP4,IFIT1,IFIT3,HLA-C,AKR1C3,HLA-A,MX1,PICK1,OAS1,IFI35,HLA-B
GO:0003374	dynamin family protein polymerization involved in mitochondrial fission	2	7	1.9	1.55E-02	9606.ENSP00000333657,9606.ENSP00000381601	MX2,MX1
GO:0046719	regulation by virus of viral protein levels in host cell	2	8	1.84	1.84E-02	9606.ENSP00000354394,9606.ENSP00000360869	STAT1,IFIT1
GO:0051234	establishment of localization	28	4248	0.26	1.84E-02	9606.ENSP00000229379,9606.ENSP00000261517,9606.ENSP00000269701,9606.ENSP00000270176,9606.ENSP00000271638,9606.ENSP00000285737,9606.ENSP00000304236,9606.ENSP00000310275,9606.ENSP00000320485,9606.ENSP00000328325,9606.ENSP00000333657,9606.ENSP00000346015,9606.ENSP00000354730,9606.ENSP00000357709,9606.ENSP00000360869,9606.ENSP00000365402,9606.ENSP00000367123,9606.ENSP00000369927,9606.ENSP00000377390,9606.ENSP00000377470,9606.ENSP00000379873,9606.ENSP00000385205,9606.ENSP00000399168,9606.ENSP00000399888,9606.ENSP00000405926,9606.ENSP00000438527,9606.ENSP00000454303,9606.ENSP00000467141	COX6A1,VPS13C,AKAP8,SCYL1,S100A11,LONP2,CD14,BANF1,ARID1A,PTTG1IP,MX2,RPL27A,CHAMP1,S100A6,IFIT1,HLA-C,SLC3A2,AKR1C3,PPP6R3,CNP,HLA-A,PICK1,HLA-B,HMGA1,TMED7,ZC3H11A,AP5B1,TTN
GO:0075713	establishment of integrated proviral latency	2	8	1.84	1.84E-02	9606.ENSP00000310275,9606.ENSP00000399888	BANF1,HMGA1

GO:0006810	transport	27	4130	0.26	2.57E-02	9606.ENSP00000229379,9606.ENSP00000261517,9606.ENSP00000269701,9606.ENSP00000270176,9606.ENSP00000271638,9606.ENSP00000285737,9606.ENSP00000304236,9606.ENSP00000310275,9606.ENSP00000320485,9606.ENSP00000328325,9606.ENSP00000333657,9606.ENSP00000346015,9606.ENSP00000357709,9606.ENSP00000360869,9606.ENSP00000365402,9606.ENSP00000367123,9606.ENSP00000369927,9606.ENSP00000377390,9606.ENSP00000377470,9606.ENSP00000379873,9606.ENSP00000385205,9606.ENSP00000399168,9606.ENSP00000399888,9606.ENSP00000405926,9606.ENSP00000438527,9606.ENSP00000454303,9606.ENSP00000467141	COX6A1,VPS13C,AKAP8,SCYL1,S100A11,LONP2,CD14,BANF1,ARID1A,PTTG1IP,MX2,RPL27A,S100A6,IFIT1,HLA-C,SLC3A2,AKR1C3,PPP6R3,CNP,HLA-A,PICK1,HLA-B,HMGA1,TMED7,ZC3H11A,AP5B1,TTN
GO:0045824	negative regulation of innate immune response	3	49	1.23	2.60E-02	9606.ENSP00000379873,9606.ENSP00000399168,9606.ENSP00000418194	HLA-A,HLA-B,PARP14
GO:0007005	mitochondrion organization	7	424	0.66	2.69E-02	9606.ENSP00000261517,9606.ENSP00000333657,9606.ENSP00000360492,9606.ENSP00000362873,9606.ENSP00000377470,9606.ENSP00000379038,9606.ENSP00000381601	VPS13C,MX2,NDUFA1,NDUFA8,CNP,TYMP,MX1
GO:0035457	cellular response to interferon-alpha	2	12	1.66	3.25E-02	9606.ENSP00000360883,9606.ENSP00000388001	IFIT3,OAS1
GO:0051179	localization	31	5233	0.21	4.20E-02	9606.ENSP00000229379,9606.ENSP00000261517,9606.ENSP00000269701,9606.ENSP00000270176,9606.ENSP00000271638,9606.ENSP00000285737,9606.ENSP00000304236,9606.ENSP00000310275,9606.ENSP00000320485,9606.ENSP00000328325,9606.ENSP00000333657,9606.ENSP00000343023,9606.ENSP00000346015,9606.ENSP00000354730,9606.ENSP00000357709,9606.ENSP00000360869,9606.ENSP00000365402,9606.ENSP00000367123,9606.ENSP00000369927,9606.ENSP00000377390,9606.ENSP00000377470,9606.ENSP00000379873,9606.ENSP00000385205,9606.ENSP00000399168,9606.ENSP00000399888,9606.ENSP00000405926,9606.ENSP00000438527,9606.ENSP00000451162,9606.ENSP00000454303,9606.ENSP00000467141,9606.ENSP00000482335	COX6A1,VPS13C,AKAP8,SCYL1,S100A11,LONP2,CD14,BANF1,ARID1A,PTTG1IP,MX2,SP100,RPL27A,CHAMP1,S100A6,IFIT1,HLA-C,SLC3A2,AKR1C3,PPP6R3,CNP,HLA-A,PICK1,HLA-B,HMGA1,TMED7,ZC3H11A,CEP128,AP5B1,TTN,MAP7
GO:0007076	mitotic chromosome condensation	2	15	1.57	4.41E-02	9606.ENSP00000269701,9606.ENSP00000467141	AKAP8,TTN
GO:0016045	detection of bacterium	2	15	1.57	4.41E-02	9606.ENSP00000379873,9606.ENSP00000399168	HLA-A,HLA-B

T = 72 h (CoV-2), IFNα16 / Untreated

#Term ID	Term Description	Observed Gene Count	Background Gene Count	Strength	False Discovery Rate	Matching Proteins in Your Input (IDs)	Matching Proteins in Your Input (Labels)
GO:0016485	protein processing	4	142	1.36	2.88E-02	9606.ENSP00000258080,9606.ENSP00000285737,9606.ENSP00000308208,9606.ENSP00000376911	HTRA2,LONP2,MMP14,LGMN

T = 72 h (CoV-2), IFNλ3 / Untreated

#Term ID	Term Description	Observed Gene Count	Background Gene Count	Strength	False Discovery Rate	Matching Proteins in Your Input (IDs)	Matching Proteins in Your Input (Labels)
GO:0034340	response to type I interferon	16	70	1.72	4.67E-19	9606.ENSP00000228928,9606.ENSP00000253110,9606.ENSP00000257570,9606.ENSP00000315768,9606.ENSP00000333657,9606.ENSP00000354394,9606.ENSP00000357459,9606.ENSP00000360869,9606.ENSP00000360883,9606.ENSP00000364016,9606.ENSP00000365402,9606.ENSP00000379873,9606.ENSP00000381601,9606.ENSP00000388001,9606.ENSP00000395590,9606.ENSP00000399168	OAS3,C19orf66,OASL,STAT2,MX2,STAT1,ADAR,IFIT1,IFIT3,PSMB8,HLA-C,HLA-A,MX1,OAS1,IFI35,HLA-B
GO:0060337	type I interferon signaling pathway	15	65	1.73	4.56E-18	9606.ENSP00000228928,9606.ENSP00000257570,9606.ENSP00000315768,9606.ENSP00000333657,9606.ENSP00000354394,9606.ENSP00000357459,9606.ENSP00000360869,9606.ENSP00000360883,9606.ENSP00000364016,9606.ENSP00000365402,9606.ENSP00000379873,9606.ENSP00000381601,9606.ENSP00000388001,9606.ENSP00000395590,9606.ENSP00000399168	OAS3,OASL,STAT2,MX2,STAT1,ADAR,IFIT1,IFIT3,PSMB8,HLA-C,HLA-A,MX1,OAS1,IFI35,HLA-B
GO:0043901	negative regulation of multi-organism process	16	212	1.24	1.12E-12	9606.ENSP00000228928,9606.ENSP00000233057,9606.ENSP00000251642,9606.ENSP00000253110,9606.ENSP00000257570,9606.ENSP00000257604,9606.ENSP00000268058,9606.ENSP00000325618,9606.ENSP00000354394,9606.ENSP00000357459,9606.ENSP00000360869,9606.ENSP00000379873,9606.ENSP00000381601,9606.ENSP00000388001,9606.ENSP00000399168,9606.ENSP00000418194	OAS3,EIF2AK2,DHX58,C19orf66,OASL,TRAFD1,PML,PARP10,STAT1,ADAR,IFIT1,HLA-A,MX1,OAS1,HLA-B,PARP14
GO:0009615	response to virus	17	270	1.16	1.86E-12	9606.ENSP00000228928,9606.ENSP00000233057,9606.ENSP00000251642,9606.ENSP00000253110,9606.ENSP00000257570,9606.ENSP00000268058,9606.ENSP00000315768,9606.ENSP00000333657,9606.ENSP00000354394,9606.ENSP00000357459,9606.ENSP00000359783,9606.ENSP00000359787,9606.ENSP00000360869,9606.ENSP00000360883,9606.ENSP00000381601,9606.ENSP00000388001,9606.ENSP00000399888	OAS3,EIF2AK2,DHX58,C19orf66,OASL,PML,STAT2,MX2,STAT1,ADAR,IFI44,IFI44L,IFIT1,IFIT3,MX1,OAS1,HMGA1
GO:0051607	defense response to virus	15	181	1.28	1.86E-12	9606.ENSP00000228928,9606.ENSP00000233057,9606.ENSP00000251642,9606.ENSP00000253110,9606.ENSP00000257570,9606.ENSP00000268058,9606.ENSP00000315768,9606.ENSP00000333657,9606.ENSP00000354394,9606.ENSP00000357459,9606.ENSP00000359787,9606.ENSP00000360869,9606.ENSP00000360883,9606.ENSP00000381601,9606.ENSP00000388001	OAS3,EIF2AK2,DHX58,C19orf66,OASL,PML,STAT2,MX2,STAT1,ADAR,IFI44L,IFIT1,IFIT3,MX1,OAS1
GO:0048525	negative regulation of viral process	11	93	1.43	2.12E-10	9606.ENSP00000228928,9606.ENSP00000233057,9606.ENSP00000253110,9606.ENSP00000257570,9606.ENSP00000268058,9606.ENSP00000325618,9606.ENSP00000354394,9606.ENSP00000357459,9606.ENSP00000360869,9606.ENSP00000381601,9606.ENSP00000388001	OAS3,EIF2AK2,C19orf66,OASL,PML,PARP10,STAT1,ADAR,IFIT1,MX1,OAS1

GO:0045087	innate immune response	21	676	0.85	3.06E-10	9606.ENSP00000228928,9606.ENSP00000233057,9606.ENSP00000251642,9606.ENSP00000253110,9606.ENSP00000257570,9606.ENSP00000268058,9606.ENSP00000269701,9606.ENSP00000315768,9606.ENSP00000333657,9606.ENSP00000354394,9606.ENSP00000357459,9606.ENSP00000360869,9606.ENSP00000360883,9606.ENSP00000364016,9606.ENSP00000365402,9606.ENSP00000379873,9606.ENSP00000381601,9606.ENSP00000388001,9606.ENSP00000395590,9606.ENSP00000399168,9606.ENSP00000418194	OAS3,EIF2AK2,DHX58,C19orf66,OASL,PML,AKAP8,STAT2,MX2,STAT1,ADAR,IFIT1,IFIT3,PSMB8,HLA-C,HLA-A,MX1,OAS1,IFI35,HLA-B,PARP14
GO:0051707	response to other organism	26	1173	0.71	6.32E-10	9606.ENSP00000228928,9606.ENSP00000233057,9606.ENSP00000251642,9606.ENSP00000253110,9606.ENSP00000256722,9606.ENSP00000257570,9606.ENSP00000268058,9606.ENSP00000269701,9606.ENSP00000315768,9606.ENSP00000333657,9606.ENSP00000354394,9606.ENSP00000357459,9606.ENSP00000359783,9606.ENSP00000359787,9606.ENSP00000360869,9606.ENSP00000360883,9606.ENSP00000364016,9606.ENSP00000365402,9606.ENSP00000377470,9606.ENSP00000379873,9606.ENSP00000381601,9606.ENSP00000388001,9606.ENSP00000395590,9606.ENSP00000399168,9606.ENSP00000399888,9606.ENSP00000418194	OAS3,EIF2AK2,DHX58,C19orf66,CMPK2,OASL,PML,AKAP8,STAT2,MX2,STAT1,ADAR,IFI44,IFI44L,IFIT1,IFIT3,PSMB8,HLA-C,CNP,HLA-A,MX1,OAS1,IFI35,HLA-B,HMGA1,PARP14
GO:1903901	negative regulation of viral life cycle	10	77	1.48	6.70E-10	9606.ENSP00000228928,9606.ENSP00000233057,9606.ENSP00000253110,9606.ENSP00000257570,9606.ENSP00000268058,9606.ENSP00000325618,9606.ENSP00000357459,9606.ENSP00000360869,9606.ENSP00000381601,9606.ENSP00000388001	OAS3,EIF2AK2,C19orf66,OASL,PML,PARP10,ADAR,IFIT1,MX1,OAS1
GO:0045071	negative regulation of viral genome replication	9	52	1.6	7.51E-10	9606.ENSP00000228928,9606.ENSP00000233057,9606.ENSP00000253110,9606.ENSP00000257570,9606.ENSP00000325618,9606.ENSP00000357459,9606.ENSP00000360869,9606.ENSP00000381601,9606.ENSP00000388001	OAS3,EIF2AK2,C19orf66,OASL,PARP10,ADAR,IFIT1,MX1,OAS1
GO:0098542	defense response to other organism	22	859	0.77	1.87E-09	9606.ENSP00000228928,9606.ENSP00000233057,9606.ENSP00000251642,9606.ENSP00000253110,9606.ENSP00000257570,9606.ENSP00000268058,9606.ENSP00000269701,9606.ENSP00000315768,9606.ENSP00000333657,9606.ENSP00000354394,9606.ENSP00000357459,9606.ENSP00000359787,9606.ENSP00000360869,9606.ENSP00000360883,9606.ENSP00000364016,9606.ENSP00000365402,9606.ENSP00000379873,9606.ENSP00000381601,9606.ENSP00000388001,9606.ENSP00000395590,9606.ENSP00000399168,9606.ENSP00000418194	OAS3,EIF2AK2,DHX58,C19orf66,OASL,PML,AKAP8,STAT2,MX2,STAT1,ADAR,IFI44L,IFIT1,IFIT3,PSMB8,HLA-C,HLA-A,MX1,OAS1,IFI35,HLA-B,PARP14
GO:0006952	defense response	25	1234	0.67	7.39E-09	9606.ENSP00000228928,9606.ENSP00000233057,9606.ENSP00000249066,9606.ENSP00000251642,9606.ENSP00000253110,9606.ENSP00000257570,9606.ENSP00000262776,9606.ENSP00000268058,9606.ENSP00000269701,9606.ENSP00000315768,9606.ENSP00000333657,9606.ENSP00000354394,9606.ENSP00000357459,9606.ENSP00000359787,9606.ENSP00000360869,9606.ENSP00000360883,9606.ENSP00000364016,9606.ENSP00000365402,9606.ENSP00000379873,9606.ENSP00000381601,9606.ENSP00000388001,9606.ENSP00000395590,9606.ENSP00000399168,9606.ENSP00000418194,9606.ENSP00000428845	OAS3,EIF2AK2,APOL2,DHX58,C19orf66,OASL,LGALS3BP,PML,AKAP8,STAT2,MX2,STAT1,ADAR,IFI44L,IFIT1,IFIT3,PSMB8,HLA-C,HLA-A,MX1,OAS1,IFI35,HLA-B,PARP14,VDAC3

GO:0000184	nuclear-transcribed mRNA catabolic process, nonsense-mediated decay	10	118	1.29	2.27E-08	9606.ENSP00000296674,9606.ENSP00000339095,9606.ENSP00000346015,9606.ENSP00000379506,9606.ENSP00000386717,9606.ENSP00000393241,9606.ENSP00000398131,9606.ENSP00000416429,9606.ENSP00000464342,9606.ENSP00000464813	RPS23,RPS7,RPL27A,RPL14,RPL31,RPS18,GSPT1,RPL32,RPL36,RPL27
GO:0006614	SRP-dependent cotranslational protein targeting to membrane	9	92	1.35	4.96E-08	9606.ENSP00000296674,9606.ENSP00000339095,9606.ENSP00000346015,9606.ENSP00000379506,9606.ENSP00000386717,9606.ENSP00000393241,9606.ENSP00000416429,9606.ENSP00000464342,9606.ENSP00000464813	RPS23,RPS7,RPL27A,RPL14,RPL31,RPS18,RPL32,RPL36,RPL27
GO:0060333	interferon-gamma-mediated signaling pathway	8	69	1.43	1.14E-07	9606.ENSP00000228928,9606.ENSP00000257570,9606.ENSP00000268058,9606.ENSP00000354394,9606.ENSP00000365402,9606.ENSP00000379873,9606.ENSP00000388001,9606.ENSP00000399168	OAS3,OASL,PML,STAT1,HLA-C,HLA-A,OAS1,HLA-B
GO:0002252	immune effector process	20	927	0.7	1.87E-07	9606.ENSP00000228928,9606.ENSP00000233057,9606.ENSP00000251642,9606.ENSP00000253110,9606.ENSP00000257570,9606.ENSP00000260118,9606.ENSP00000268058,9606.ENSP00000289228,9606.ENSP00000315768,9606.ENSP00000333657,9606.ENSP00000354394,9606.ENSP00000357459,9606.ENSP00000359787,9606.ENSP00000360869,9606.ENSP00000360883,9606.ENSP00000365402,9606.ENSP00000379873,9606.ENSP00000381601,9606.ENSP00000388001,9606.ENSP00000399168	OAS3,EIF2AK2,DHX58,C19orf66,OASL,GGH,PML,ACTR1B,STAT2,MX2,STAT1,ADAR,IFI44L,IFIT1,IFIT3,HLA-C,HLA-A,MX1,OAS1,HLA-B
GO:0034097	response to cytokine	21	1035	0.67	1.94E-07	9606.ENSP00000228928,9606.ENSP00000233057,9606.ENSP00000253110,9606.ENSP00000257570,9606.ENSP00000257604,9606.ENSP00000258080,9606.ENSP00000268058,9606.ENSP00000290573,9606.ENSP00000315768,9606.ENSP00000333657,9606.ENSP00000354394,9606.ENSP00000357459,9606.ENSP00000360869,9606.ENSP00000360883,9606.ENSP00000364016,9606.ENSP00000365402,9606.ENSP00000379873,9606.ENSP00000381601,9606.ENSP00000388001,9606.ENSP00000395590,9606.ENSP00000399168	OAS3,EIF2AK2,C19orf66,OASL,TRAFD1,HTRA2,PML,HK2,STAT2,MX2,STAT1,ADAR,IFIT1,IFIT3,PSMB8,HLA-C,HLA-A,MX1,OAS1,IFI35,HLA-B
GO:0051704	multi-organism process	32	2514	0.47	4.74E-07	9606.ENSP00000228928,9606.ENSP00000233057,9606.ENSP00000249066,9606.ENSP00000251642,9606.ENSP00000253110,9606.ENSP00000256722,9606.ENSP00000257570,9606.ENSP00000261574,9606.ENSP00000268058,9606.ENSP00000269701,9606.ENSP00000270586,9606.ENSP00000315768,9606.ENSP00000333657,9606.ENSP00000354394,9606.ENSP00000357459,9606.ENSP00000359300,9606.ENSP00000359783,9606.ENSP00000359787,9606.ENSP00000360869,9606.ENSP00000360883,9606.ENSP00000363993,9606.ENSP00000364016,9606.ENSP00000365402,9606.ENSP00000377470,9606.ENSP00000379873,9606.ENSP00000381601,9606.ENSP00000388001,9606.ENSP00000392985,9606.ENSP00000395590,9606.ENSP00000399168,9606.ENSP00000399888,9606.ENSP00000418194	OAS3,EIF2AK2,APOL2,DHX58,C19orf66,CMPK2,OASL,IPO5,PML,AKAP8,PSMB6,STAT2,MX2,STAT1,ADAR,CETN2,IFI44,IFI44L,IFIT1,IFIT3,PSMB9,PSMB8,HLA-C,CNP,HLA-A,MX1,OAS1,HTATIP2,IFI35,HLA-B,HMGA1,PARP14
GO:0002181	cytoplasmic translation	7	57	1.45	6.58E-07	9606.ENSP00000296674,9606.ENSP00000339095,9606.ENSP00000346015,9606.ENSP00000386717,9606.ENSP00000398131,9606.ENSP00000416429,9606.ENSP00000464342	RPS23,RPS7,RPL27A,RPL31,GSPT1,RPL32,RPL36
GO:0006413	translational initiation	9	142	1.16	1.01E-06	9606.ENSP00000296674,9606.ENSP00000339095,9606.ENSP00000346015,9606.ENSP00000379506,9606.ENSP00000386717,9606.ENSP00000393241,9606.ENSP00000416429,9606.ENSP00000464342,9606.ENSP00000464813	RPS23,RPS7,RPL27A,RPL14,RPL31,RPS18,RPL32,RPL36,RPL27

GO:0034655	nucleobase-containing compound catabolic process	13	394	0.88	1.09E-06	9606.ENSP00000290573,9606.ENSP00000296674,9606.ENSP00000339095,9606.ENSP00000346015,9606.ENSP00000377470,9606.ENSP00000379038,9606.ENSP00000379506,9606.ENSP00000386717,9606.ENSP00000393241,9606.ENSP00000398131,9606.ENSP00000416429,9606.ENSP00000464342,9606.ENSP00000464813	HK2,RPS23,RPS7,RPL27A,CNP,TYMP,RPL14,RPL31,RPS18,GSPT1,RPL32,RPL36,RPL27
GO:0072594	establishment of protein localization to organelle	13	396	0.88	1.12E-06	9606.ENSP00000261574,9606.ENSP00000268058,9606.ENSP00000290573,9606.ENSP00000296674,9606.ENSP00000339095,9606.ENSP00000346015,9606.ENSP00000357459,9606.ENSP00000379506,9606.ENSP00000386717,9606.ENSP00000393241,9606.ENSP00000416429,9606.ENSP00000464342,9606.ENSP00000464813	IPO5,PML,HK2,RPS23,RPS7,RPL27A,ADAR,RPL14,RPL31,RPS18,RPL32,RPL36,RPL27
GO:0019221	cytokine-mediated signaling pathway	16	655	0.75	1.23E-06	9606.ENSP00000228928,9606.ENSP00000257570,9606.ENSP00000268058,9606.ENSP00000315768,9606.ENSP00000333657,9606.ENSP00000354394,9606.ENSP00000357459,9606.ENSP00000360869,9606.ENSP00000360883,9606.ENSP00000364016,9606.ENSP00000365402,9606.ENSP00000379873,9606.ENSP00000381601,9606.ENSP00000388001,9606.ENSP00000395590,9606.ENSP00000399168	OAS3,OASL,PML,STAT2,MX2,STAT1,ADAR,IFIT1,IFIT3,PSMB8,HLA-C,HLA-A,MX1,OAS1,IFI35,HLA-B
GO:0016032	viral process	15	571	0.78	1.37E-06	9606.ENSP00000233057,9606.ENSP00000251642,9606.ENSP00000261574,9606.ENSP00000268058,9606.ENSP00000270586,9606.ENSP00000315768,9606.ENSP00000354394,9606.ENSP00000360869,9606.ENSP00000363993,9606.ENSP00000364016,9606.ENSP00000365402,9606.ENSP00000379873,9606.ENSP00000392985,9606.ENSP00000399168,9606.ENSP00000399888	EIF2AK2,DHX58,IPO5,PML,PSMB6,STAT2,STAT1,IFIT1,PSMB9,PSMB8,HLA-C,HLA-A,HTATIP2,HLA-B,HMGA1
GO:0006955	immune response	24	1560	0.55	1.57E-06	9606.ENSP00000228928,9606.ENSP00000233057,9606.ENSP00000251642,9606.ENSP00000253110,9606.ENSP00000257570,9606.ENSP00000260118,9606.ENSP00000268058,9606.ENSP00000269701,9606.ENSP00000289228,9606.ENSP00000315768,9606.ENSP00000333657,9606.ENSP00000354394,9606.ENSP00000357459,9606.ENSP00000360869,9606.ENSP00000360883,9606.ENSP00000364016,9606.ENSP00000365402,9606.ENSP00000379873,9606.ENSP00000381601,9606.ENSP00000388001,9606.ENSP00000395590,9606.ENSP00000399168,9606.ENSP00000404833,9606.ENSP00000418194	OAS3,EIF2AK2,DHX58,C19orf66,OASL,GGH,PML,AKAP8,ACTR1B,STAT2,MX2,STAT1,ADAR,IFIT1,IFIT3,PSMB8,HLA-C,HLA-A,MX1,OAS1,IFI35,HLA-B,TAPBP,PARP14
GO:0006518	peptide metabolic process	14	497	0.81	1.68E-06	9606.ENSP00000225719,9606.ENSP00000233057,9606.ENSP00000296674,9606.ENSP00000339095,9606.ENSP00000346015,9606.ENSP00000347495,9606.ENSP00000379506,9606.ENSP00000386717,9606.ENSP00000393241,9606.ENSP00000398131,9606.ENSP00000404833,9606.ENSP00000416429,9606.ENSP00000464342,9606.ENSP00000464813	CPD,EIF2AK2,RPS23,RPS7,RPL27A,WARS,RPL14,RPL31,RPS18,GSPT1,TAPBP,RPL32,RPL36,RPL27
GO:0006412	translation	12	362	0.88	3.09E-06	9606.ENSP00000233057,9606.ENSP00000296674,9606.ENSP00000339095,9606.ENSP00000346015,9606.ENSP00000347495,9606.ENSP00000379506,9606.ENSP00000386717,9606.ENSP00000393241,9606.ENSP00000398131,9606.ENSP00000416429,9606.ENSP00000464342,9606.ENSP00000464813	EIF2AK2,RPS23,RPS7,RPL27A,WARS,RPL14,RPL31,RPS18,GSPT1,RPL32,RPL36,RPL27
GO:0035455	response to interferon-alpha	5	22	1.72	4.01E-06	9606.ENSP00000233057,9606.ENSP00000333657,9606.ENSP00000357459,9606.ENSP00000360883,9606.ENSP00000388001	EIF2AK2,MX2,ADAR,IFIT3,OAS1

GO:0034341	response to interferon-gamma	9	176	1.07	4.11E-06	9606.ENSP00000228928,9606.ENSP00000253110,9606.ENSP00000257570,9606.ENSP00000268058,9606.ENSP00000354394,9606.ENSP00000365402,9606.ENSP00000379873,9606.ENSP00000388001,9606.ENSP00000399168	OAS3,C19orf66,OASL,PML,STAT1,HLA-C,HLA-A,OAS1,HLA-B
GO:0043603	cellular amide metabolic process	16	732	0.7	4.25E-06	9606.ENSP00000225719,9606.ENSP00000233057,9606.ENSP00000258080,9606.ENSP00000260118,9606.ENSP00000296674,9606.ENSP00000339095,9606.ENSP00000346015,9606.ENSP00000347495,9606.ENSP00000379506,9606.ENSP00000386717,9606.ENSP00000393241,9606.ENSP00000398131,9606.ENSP00000404833,9606.ENSP00000416429,9606.ENSP00000464342,9606.ENSP00000464813	CPD,EIF2AK2,HTRA2,GGH,RPS23,RPS7,RPL27A,WARS,RPL14,RPL31,RPS18,GSPT1,TAPBP,RPL32,RPL36,RPL27
GO:0044265	cellular macromolecule catabolic process	17	842	0.67	4.70E-06	9606.ENSP00000258080,9606.ENSP00000268058,9606.ENSP00000270586,9606.ENSP00000296674,9606.ENSP00000333266,9606.ENSP00000339095,9606.ENSP00000346015,9606.ENSP00000363993,9606.ENSP00000364016,9606.ENSP00000379506,9606.ENSP00000386717,9606.ENSP00000393241,9606.ENSP00000398131,9606.ENSP00000416429,9606.ENSP00000464087,9606.ENSP00000464342,9606.ENSP00000464813	HTRA2,PML,PSMB6,RPS23,UBA7,RPS7,RPL27A,PSMB9,PSMB8,RPL14,RPL31,RPS18,GSPT1,RPL32,RNF213,RPL36,RPL27
GO:0045824	negative regulation of innate immune response	6	49	1.45	4.78E-06	9606.ENSP00000251642,9606.ENSP00000257604,9606.ENSP00000357459,9606.ENSP00000379873,9606.ENSP00000399168,9606.ENSP00000418194	DHX58,TRAFD1,ADAR,HLA-A,HLA-B,PARP14
GO:0071345	cellular response to cytokine stimulus	18	953	0.64	4.82E-06	9606.ENSP00000228928,9606.ENSP00000257570,9606.ENSP00000258080,9606.ENSP00000268058,9606.ENSP00000290573,9606.ENSP00000315768,9606.ENSP00000333657,9606.ENSP00000354394,9606.ENSP00000357459,9606.ENSP00000360869,9606.ENSP00000360883,9606.ENSP00000364016,9606.ENSP00000365402,9606.ENSP00000379873,9606.ENSP00000381601,9606.ENSP00000388001,9606.ENSP00000395590,9606.ENSP00000399168	OAS3,OASL,HTRA2,PML,HK2,STAT2,MX2,STAT1,ADAR,IFIT1,IFIT3,PSMB8,HLA-C,HLA-A,MX1,OAS1,IFI35,HLA-B
GO:0002832	negative regulation of response to biotic stimulus	7	85	1.28	4.83E-06	9606.ENSP00000251642,9606.ENSP00000257604,9606.ENSP00000357459,9606.ENSP00000360869,9606.ENSP00000379873,9606.ENSP00000399168,9606.ENSP00000418194	DHX58,TRAFD1,ADAR,IFIT1,HLA-A,HLA-B,PARP14
GO:0019885	antigen processing and presentation of endogenous peptide antigen via MHC class I	4	9	2.01	7.22E-06	9606.ENSP00000365402,9606.ENSP00000379873,9606.ENSP00000399168,9606.ENSP00000404833	HLA-C,HLA-A,HLA-B,TAPBP
GO:0009605	response to external stimulus	27	2152	0.46	7.63E-06	9606.ENSP00000228928,9606.ENSP00000233057,9606.ENSP00000251642,9606.ENSP00000253110,9606.ENSP00000256722,9606.ENSP00000257570,9606.ENSP00000268058,9606.ENSP00000269701,9606.ENSP00000315768,9606.ENSP00000333657,9606.ENSP00000354394,9606.ENSP00000357459,9606.ENSP00000359783,9606.ENSP00000359787,9606.ENSP00000360869,9606.ENSP00000360883,9606.ENSP00000364016,9606.ENSP00000365402,9606.ENSP00000377470,9606.ENSP00000379038,9606.ENSP00000379873,9606.ENSP00000381601,9606.ENSP00000388001,9606.ENSP00000395590,9606.ENSP00000399168,9606.ENSP00000399888,9606.ENSP00000418194	OAS3,EIF2AK2,DHX58,C19orf66,CMPK2,OASL,PML,AKAP8,STAT2,MX2,STAT1,ADAR,IFI44,IFI44L,IFIT1,IFIT3,PSMB8,HLA-C,CNP,TYMP,HLA-A,MX1,OAS1,IFI35,HLA-B,HMGA1,PARP14

GO:0016071	mRNA metabolic process	14	667	0.68	3.45E-05	9606.ENSP00000293677,9606.ENSP00000296674,9606.ENSP00000339095,9606.ENSP00000346015,9606.ENSP00000357459,9606.ENSP00000374399,9606.ENSP00000379506,9606.ENSP00000386717,9606.ENSP00000393241,9606.ENSP00000398131,9606.ENSP00000400591,9606.ENSP00000416429,9606.ENSP00000464342,9606.ENSP00000464813	RAVER1,RPS23,RPS7,RPL27A,ADAR,TRMT61A,RPL14,RPL31,RPS18,GSPT1,SNRPE,RPL32,RPL36,RPL27
GO:1901566	organonitrogen compound biosynthetic process	20	1370	0.53	3.91E-05	9606.ENSP00000233057,9606.ENSP00000256722,9606.ENSP00000290573,9606.ENSP00000296674,9606.ENSP00000300688,9606.ENSP00000325618,9606.ENSP00000339095,9606.ENSP00000346015,9606.ENSP00000347495,9606.ENSP00000356978,9606.ENSP00000379038,9606.ENSP00000379506,9606.ENSP00000386717,9606.ENSP00000388001,9606.ENSP00000393241,9606.ENSP00000398131,9606.ENSP00000416429,9606.ENSP00000417901,9606.ENSP00000464342,9606.ENSP00000464813	EIF2AK2,CMPK2,HK2,RPS23,ATP5L,PARP10,RPS7,RPL27A,WARS,PPOX,TYMP,RPL14,RPL31,OAS1,RPS18,GSPT1,RPL32,GPHN,RPL36,RPL27
GO:0006605	protein targeting	10	318	0.86	4.10E-05	9606.ENSP00000268058,9606.ENSP00000296674,9606.ENSP00000339095,9606.ENSP00000346015,9606.ENSP00000379506,9606.ENSP00000386717,9606.ENSP00000393241,9606.ENSP00000416429,9606.ENSP00000464342,9606.ENSP00000464813	PML,RPS23,RPS7,RPL27A,RPL14,RPL31,RPS18,RPL32,RPL36,RPL27
GO:0002486	antigen processing and presentation of endogenous peptide antigen via MHC class I via ER pathway, TAP-independent	3	3	2.36	4.14E-05	9606.ENSP00000365402,9606.ENSP00000379873,9606.ENSP00000399168	HLA-C,HLA-A,HLA-B
GO:0046907	intracellular transport	20	1390	0.52	4.59E-05	9606.ENSP00000261517,9606.ENSP00000261574,9606.ENSP00000268058,9606.ENSP00000296674,9606.ENSP00000300688,9606.ENSP00000339095,9606.ENSP00000346015,9606.ENSP00000357459,9606.ENSP00000360869,9606.ENSP00000377390,9606.ENSP00000379506,9606.ENSP00000386717,9606.ENSP00000392985,9606.ENSP00000393241,9606.ENSP00000399888,9606.ENSP00000400591,9606.ENSP00000405926,9606.ENSP00000416429,9606.ENSP00000464342,9606.ENSP00000464813	VPS13C,IPO5,PML,RPS23,ATP5L,RPS7,RPL27A,ADAR,IFIT1,PPP6R3,RPL14,RPL31,HTATIP2,RPS18,HMGA1,SNRPE,TMED7,RPL32,RPL36,RPL27
GO:0034641	cellular nitrogen compound metabolic process	42	5126	0.28	1.10E-04	9606.ENSP00000225719,9606.ENSP00000228928,9606.ENSP00000233057,9606.ENSP00000256722,9606.ENSP00000258080,9606.ENSP00000260118,9606.ENSP00000267935,9606.ENSP00000268058,9606.ENSP00000269701,9606.ENSP00000290573,9606.ENSP00000293677,9606.ENSP00000296674,9606.ENSP00000300688,9606.ENSP00000315768,9606.ENSP00000325618,9606.ENSP00000339095,9606.ENSP00000346015,9606.ENSP00000347495,9606.ENSP00000354394,9606.ENSP00000356978,9606.ENSP00000357459,9606.ENSP00000359300,9606.ENSP00000367939,9606.ENSP00000374399,9606.ENSP00000377470,9606.ENSP00000379038,9606.ENSP00000379506,9606.ENSP00000386717,9606.ENSP00000388001,9606.ENSP00000393241,9606.ENSP00000398131,9606.ENSP00000399888,9606.ENSP00000400591,9606.ENSP00000404833,9606.ENSP00000410769,9606.ENSP00000416429,9606.ENSP00000417901,9606.ENSP00000418194,9606.ENSP00000420588,9606.ENSP00000464342,9606.ENSP00000464813,9606.ENSP00000479106	CPD,OAS3,EIF2AK2,CMPK2,HTRA2,GGH,COMMD4,PML,AKAP8,HK2,RAVER1,RPS23,ATP5L,STAT2,PARP10,RPS7,RPL27A,WARS,STAT1,PPOX,ADAR,CETN2,UQCRQ,TRMT61A,CNP,TYMP,RPL14,RPL31,OAS1,RPS18,GSPT1,HMGA1,SNRPE,TAPBP,CNBP,RPL32,GPHN,PARP14,TFAM,RPL36,RPL27,HIST1H4A

GO:0043933	protein-containing complex subunit organization	22	1770	0.46	1.20E-04	9606.ENSP00000258080,9606.ENSP00000268058,9606.ENSP00000296674,9606.ENSP00000307705,9606.ENSP00000333657,9606.ENSP00000355330,9606.ENSP00000357459,9606.ENSP00000359300,9606.ENSP00000374566,9606.ENSP00000377390,9606.ENSP00000378563,9606.ENSP00000379873,9606.ENSP00000381601,9606.ENSP00000388001,9606.ENSP00000398131,9606.ENSP00000399888,9606.ENSP00000400591,9606.ENSP00000404833,9606.ENSP00000420588,9606.ENSP00000464087,9606.ENSP00000478061,9606.ENSP00000479106	HTRA2,PML,RPS23,HIST1H1E,MX2,TGM2,ADAR,CETN2,KIF19,PPP6R3,PFDN6,HLA-A,MX1,OAS1,GSPT1,HMGA1,SNRPE,TAPBP,TFAM,RNF213,MARCKS,HIST1H4A
GO:0048519	negative regulation of biological process	41	4953	0.28	1.20E-04	9606.ENSP00000228928,9606.ENSP00000233057,9606.ENSP00000251642,9606.ENSP00000253110,9606.ENSP00000257570,9606.ENSP00000257604,9606.ENSP00000258080,9606.ENSP00000261517,9606.ENSP00000261574,9606.ENSP00000268058,9606.ENSP00000269701,9606.ENSP00000290573,9606.ENSP00000296674,9606.ENSP00000307705,9606.ENSP00000325618,9606.ENSP00000333266,9606.ENSP00000339095,9606.ENSP00000346015,9606.ENSP00000347495,9606.ENSP00000354394,9606.ENSP00000357459,9606.ENSP00000360869,9606.ENSP00000360883,9606.ENSP00000379506,9606.ENSP00000379873,9606.ENSP00000381601,9606ENSP00000386717,9606.ENSP00000388001,9606.ENSP00000392985,9606.ENSP00000393241,9606.ENSP00000398131,9606.ENSP00000399168,9606.ENSP00000399888,9606.ENSP00000410769,9606.ENSP00000416429,9606.ENSP00000418194,9606.ENSP00000464087,9606.ENSP00000464342,9606.ENSP00000464813,9606.ENSP00000471242,9606.ENSP00000479106	OAS3,EIF2AK2,DHX58,C19orf66,OASL,TRAFD1,HTRA2,VPS13C,IPO5,PML,AKAP8,HK2,RPS23,HIST1H1E,PARP10,UBA7,RPS7,RPL27A,WARS,STAT1,ADAR,IFIT1,IFIT3,RPL14,HLA-A,MX1,RPL31,OAS1,HTATIP2,RPS18,GSPT1,HLA-B,HMGA1,CNBP,RPL32,PARP14,RNF213,RPL36,RPL27,FARP1,HIST1H4A
GO:1901360	organic cyclic compound metabolic process	41	4963	0.28	1.20E-04	9606.ENSP00000228928,9606.ENSP00000233057,9606.ENSP00000249066,9606.ENSP00000256722,9606.ENSP00000258080,9606.ENSP00000260118,9606.ENSP00000267935,9606.ENSP00000268058,9606.ENSP00000269701,9606.ENSP00000290573,9606.ENSP00000293677,9606.ENSP00000296674,9606.ENSP00000300688,9606.ENSP00000315768,9606.ENSP00000325618,9606.ENSP00000339095,9606.ENSP00000346015,9606.ENSP00000347495,9606.ENSP00000354394,9606.ENSP00000356978,9606.ENSP00000357459,9606.ENSP00000359300,9606.ENSP00000367939,9606.ENSP00000374399,9606.ENSP00000377470,9606.ENSP00000379038,9606ENSP00000379506,9606.ENSP00000386717,9606.ENSP00000388001,9606.ENSP00000393241,9606.ENSP00000398131,9606.ENSP00000399888,9606.ENSP00000400591,9606.ENSP00000410769,9606.ENSP00000416429,9606.ENSP00000417901,9606.ENSP00000418194,9606.ENSP00000420588,9606.ENSP00000464342,9606.ENSP00000464813,9606.ENSP00000479106	OAS3,EIF2AK2,APOL2,CMPK2,HTRA2,GGH,COMMD4,PML,AKAP8,HK2,RAVER1,RPS23,ATP5L,STAT2,PARP10,RPS7,RPL27A,WARS,STAT1,PPOX,ADAR,CETN2,UQCRQ,TRMT61A,CNP,TYMP,RPL14,RPL31,OAS1,RPS18,GSPT1,HMGA1,SNRPE,CNBP,RPL32,GPHN,PARP14,TFAM,RPL36,RPL27,HIST1H4A

GO:0002376	immune system process	26	2370	0.4	1.30E-04	9606.ENSP00000228928,9606.ENSP00000233057,9606.ENSP00000251642,9606.ENSP00000253110,9606.ENSP00000257570,9606.ENSP00000260118,9606.ENSP00000268058,9606.ENSP00000269701,9606.ENSP00000289228,9606.ENSP00000315768,9606.ENSP00000333657,9606.ENSP00000354394,9606.ENSP00000357459,9606.ENSP00000359787,9606.ENSP00000360869,9606.ENSP00000360883,9606.ENSP00000363993,9606.ENSP00000364016,9606.ENSP00000365402,9606.ENSP00000379873,9606.ENSP00000381601,9606.ENSP00000388001,9606.ENSP00000395590,9606.ENSP00000399168,9606.ENSP00000404833,9606.ENSP00000418194	OAS3,EIF2AK2,DHX58,C19orf66,OASL,GGH,PML,AKAP8,ACTR1B,STAT2,MX2,STAT1,ADAR,IFI44L,IFIT1,IFIT3,PSMB9,PSMB8,HLA-C,HLA-A,MX1,OAS1,IFI35,HLA-B,TAPBP,PARP14
GO:0065003	protein-containing complex assembly	20	1514	0.48	1.50E-04	9606.ENSP00000258080,9606.ENSP00000268058,9606.ENSP00000296674,9606.ENSP00000307705,9606.ENSP00000333657,9606.ENSP00000355330,9606.ENSP00000357459,9606.ENSP00000359300,9606.ENSP00000377390,9606.ENSP00000378563,9606.ENSP00000379873,9606.ENSP00000381601,9606.ENSP00000388001,9606.ENSP00000399888,9606.ENSP00000400591,9606.ENSP00000404833,9606.ENSP00000420588,9606.ENSP00000464087,9606.ENSP00000478061,9606.ENSP00000479106	HTRA2,PML,RPS23,HIST1H1E,MX2,TGM2,ADAR,CETN2,PPP6R3,PFDN6,HLA-A,MX1,OAS1,HMGA1,SNRPE,TAPBP,TFAM,RNF213,MARCKS,HIST1H4A
GO:0002479	antigen processing and presentation of exogenous peptide antigen via MHC class I, TAP-dependent	4	25	1.57	1.60E-04	9606.ENSP00000365402,9606.ENSP00000379873,9606.ENSP00000399168,9606.ENSP00000404833	HLA-C,HLA-A,HLA-B,TAPBP
GO:0006810	transport	36	4130	0.3	2.00E-04	9606.ENSP00000249066,9606.ENSP00000260118,9606.ENSP00000261517,9606.ENSP00000261574,9606.ENSP00000262776,9606.ENSP00000268058,9606.ENSP00000269701,9606.ENSP00000289228,9606.ENSP00000290573,9606.ENSP00000296674,9606.ENSP00000300688,9606.ENSP00000303366,9606.ENSP00000316007,9606.ENSP00000333657,9606.ENSP00000339095,9606.ENSP00000346015,9606.ENSP00000355330,9606.ENSP00000357459,9606.ENSP00000360869,9606.ENSP00000365402,9606.ENSP00000377390,9606.ENSP00000377470,9606.ENSP00000379506,9606.ENSP00000379873,9606.ENSP00000386717,9606.ENSP00000392985,9606.ENSP00000393241,9606.ENSP00000399168,9606.ENSP00000399888,9606.ENSP00000400591,9606.ENSP00000404833,9606.ENSP00000405926,9606.ENSP00000416429,9606.ENSP00000428845,9606.ENSP00000464342,9606.ENSP00000464813	APOL2,GGH,VPS13C,IPO5,LGALS3BP,PML,AKAP8,ACTR1B,HK2,RPS23,ATP5L,LMAN2,SCAMP4,MX2,RPS7,RPL27A,TGM2,ADAR,IFIT1,HLA-C,PPP6R3,CNP,RPL14,HLA-A,RPL31,HTATIP2,RPS18,HLA-B,HMGA1,SNRPE,TAPBP,TMED7,RPL32,VDAC3,RPL36,RPL27

GO:0006950	response to stress	31	3267	0.34	2.20E-04	9606.ENSP00000228928,9606.ENSP00000233057,9606.ENSP00000249066,9606.ENSP00000251642,9606.ENSP00000253110,9606.ENSP00000257570,9606.ENSP00000258080,9606.ENSP00000262776,9606.ENSP00000268058,9606.ENSP00000269701,9606.ENSP00000290573,9606.ENSP00000315768,9606.ENSP00000333266,9606.ENSP00000333657,9606.ENSP00000354394,9606.ENSP00000357459,9606.ENSP00000359300,9606.ENSP00000359787,9606.ENSP00000360869,9606.ENSP00000360883,9606.ENSP00000364016,9606.ENSP00000365402,9606.ENSP00000379873,9606.ENSP00000381601,9606.ENSP00000388001,9606.ENSP00000395590,9606.ENSP00000399168,9606.ENSP00000399888,9606.ENSP00000418194,9606.ENSP00000428845,9606.ENSP00000479106	OAS3,EIF2AK2,APOL2,DHX58,C19orf66,OASL,HTRA2,LGALS3BP,PML,AKAP8,HK2,STAT2,UBA7,MX2,STAT1,ADAR,CETN2,IFI44L,IFIT1,IFIT3,PSMB8,HLA-C,HLA-A,MX1,OAS1,IFI35,HLA-B,HMGA1,PARP14,VDAC3,HIST1H4A
GO:0046483	heterocycle metabolic process	39	4716	0.28	2.20E-04	9606.ENSP00000228928,9606.ENSP00000233057,9606.ENSP00000256722,9606.ENSP00000260118,9606.ENSP00000267935,9606.ENSP00000268058,9606.ENSP00000269701,9606.ENSP00000290573,9606.ENSP00000293677,9606.ENSP00000296674,9606.ENSP00000300688,9606.ENSP00000315768,9606.ENSP00000325618,9606.ENSP00000339095,9606.ENSP00000346015,9606.ENSP00000347495,9606.ENSP00000354394,9606.ENSP00000356978,9606.ENSP00000357459,9606.ENSP00000359300,9606.ENSP00000367939,9606.ENSP00000374399,9606.ENSP00000377470,9606.ENSP00000379038,9606.ENSP00000379506,9606.ENSP00000386717,9606.ENSP00000388001,9606.ENSP00000393241,9606.ENSP00000398131,9606.ENSP00000399888,9606.ENSP00000400591,9606.ENSP00000410769,9606.ENSP00000416429,9606.ENSP00000417901,9606.ENSP00000418194,9606.ENSP00000420588,9606.ENSP00000464342,9606.ENSP00000464813,9606.ENSP00000479106	OAS3,EIF2AK2,CMPK2,GGH,COMMD4,PML,AKAP8,HK2,RAVER1,RPS23,ATP5L,STAT2,PARP10,RPS7,RPL27A,WARS,STAT1,PPOX,ADAR,CETN2,UQCRQ,TRMT61A,CNP,TYMP,RPL14,RPL31,OAS1,RPS18,GSPT1,HMGA1,SNRPE,CNBP,RPL32,GPHN,PARP14,TFAM,RPL36,RPL27,HIST1H4A
GO:0042886	amide transport	19	1440	0.48	2.50E-04	9606.ENSP00000261574,9606.ENSP00000268058,9606.ENSP00000269701,9606.ENSP00000296674,9606.ENSP00000303366,9606.ENSP00000316007,9606.ENSP00000333657,9606.ENSP00000339095,9606.ENSP00000346015,9606.ENSP00000357459,9606.ENSP00000360869,9606.ENSP00000379506,9606.ENSP00000386717,9606.ENSP00000393241,9606.ENSP00000404833,9606.ENSP00000405926,9606.ENSP00000416429,9606.ENSP00000464342,9606.ENSP00000464813	IPO5,PML,AKAP8,RPS23,LMAN2,SCAMP4,MX2,RPS7,RPL27A,ADAR,IFIT1,RPL14,RPL31,RPS18,TAPBP,TMED7,RPL32,RPL36,RPL27
GO:0002480	antigen processing and presentation of exogenous peptide antigen via MHC class I, TAP-independent	3	8	1.94	2.60E-04	9606.ENSP00000365402,9606.ENSP00000379873,9606.ENSP00000399168	HLA-C,HLA-A,HLA-B

GO:0006725	cellular aromatic compound metabolic process	39	4754	0.28	2.60E-04	9606.ENSP00000228928,9606.ENSP00000233057,9606.ENSP00000256722,9606.ENSP00000260118,9606.ENSP00000267935,9606.ENSP00000268058,9606.ENSP00000269701,9606.ENSP00000290573,9606.ENSP00000293677,9606.ENSP00000296674,9606.ENSP00000300688,9606.ENSP00000315768,9606.ENSP00000325618,9606.ENSP00000339095,9606.ENSP00000346015,9606.ENSP00000347495,9606.ENSP00000354394,9606.ENSP00000356978,9606.ENSP00000357459,9606.ENSP00000359300,9606.ENSP00000367939,9606.ENSP00000374399,9606.ENSP00000377470,9606.ENSP00000379038,9606.ENSP00000379506,9606.ENSP00000386717,9606.ENSP00000388001,9606.ENSP00000393241,9606.ENSP00000398131,9606.ENSP00000399888,9606.ENSP00000400591,9606.ENSP00000410769,9606.ENSP00000416429,9606.ENSP00000417901,9606.ENSP00000418194,9606.ENSP00000420588,9606.ENSP00000464342,9606.ENSP00000464813,9606.ENSP00000479106	OAS3,EIF2AK2,CMPK2,GGH,COMMD4,PML,AKAP8,HK2,RAVER1,RPS23,ATP5L,STAT2,PARP10,RPS7,RPL27A,WARS,STAT1,PPOX,ADAR,CETN2,UQCRQ,TRMT61A,CNP,TYMP,RPL14,RPL31,OAS1,RPS18,GSPT1,HMGA1,SNRPE,CNBP,RPL32,GPHN,PARP14,TFAM,RPL36,RPL27,HIST1H4A
GO:0072657	protein localization to membrane	10	405	0.75	2.60E-04	9606.ENSP00000296674,9606.ENSP00000339095,9606.ENSP00000346015,9606.ENSP00000379506,9606.ENSP00000386717,9606.ENSP00000393241,9606.ENSP00000416429,9606.ENSP00000417901,9606.ENSP00000464342,9606.ENSP00000464813	RPS23,RPS7,RPL27A,RPL14,RPL31,RPS18,RPL32,GPHN,RPL36,RPL27
GO:0045184	establishment of protein localization	19	1467	0.47	3.10E-04	9606.ENSP00000261574,9606.ENSP00000268058,9606.ENSP00000269701,9606.ENSP00000290573,9606.ENSP00000296674,9606.ENSP00000303366,9606.ENSP00000316007,9606.ENSP00000333657,9606.ENSP00000339095,9606.ENSP00000346015,9606.ENSP00000357459,9606.ENSP00000360869,9606.ENSP00000379506,9606.ENSP00000386717,9606.ENSP00000393241,9606.ENSP00000405926,9606.ENSP00000416429,9606.ENSP00000464342,9606.ENSP00000464813	IPO5,PML,AKAP8,HK2,RPS23,LMAN2,SCAMP4,MX2,RPS7,RPL27A,ADAR,IFIT1,RPL14,RPL31,RPS18,TMED7,RPL32,RPL36,RPL27
GO:1901575	organic substance catabolic process	20	1609	0.46	3.20E-04	9606.ENSP00000258080,9606.ENSP00000268058,9606.ENSP00000270586,9606.ENSP00000290573,9606.ENSP00000296674,9606.ENSP00000333266,9606.ENSP00000339095,9606.ENSP00000346015,9606.ENSP00000363993,9606.ENSP00000364016,9606.ENSP00000377470,9606.ENSP00000379038,9606.ENSP00000379506,9606.ENSP00000386717,9606.ENSP00000393241,9606.ENSP00000398131,9606.ENSP00000416429,9606.ENSP00000464087,9606.ENSP00000464342,9606.ENSP00000464813	HTRA2,PML,PSMB6,HK2,RPS23,UBA7,RPS7,RPL27A,PSMB9,PSMB8,CNP,TYMP,RPL14,RPL31,RPS18,GSPT1,RPL32,RNF213,RPL36,RPL27
GO:0031348	negative regulation of defense response	7	180	0.95	3.30E-04	9606.ENSP00000251642,9606.ENSP00000257604,9606.ENSP00000357459,9606.ENSP00000360869,9606.ENSP00000379873,9606.ENSP00000399168,9606.ENSP00000418194	DHX58,TRAFD1,ADAR,IFIT1,HLA-A,HLA-B,PARP14
GO:0044085	cellular component biogenesis	26	2556	0.37	4.10E-04	9606.ENSP00000258080,9606.ENSP00000268058,9606.ENSP00000296674,9606.ENSP00000307705,9606.ENSP00000333657,9606.ENSP00000339095,9606.ENSP00000355330,9606.ENSP00000357459,9606.ENSP00000359300,9606.ENSP00000377390,9606.ENSP00000378563,9606.ENSP00000379506,9606.ENSP00000379873,9606.ENSP00000381601,9606.ENSP00000388001,9606.ENSP00000393241,9606.ENSP00000399888,9606.ENSP00000400591,9606.ENSP00000404833,9606.ENSP00000420588,9606.ENSP00000464087,9606.ENSP00000464813,9606.ENSP00000471242,9606.ENSP00000478061,9606.ENSP00000479106,9606.ENSP00000484121	HTRA2,PML,RPS23,HIST1H1E,MX2,RPS7,TGM2,ADAR,CETN2,PPP6R3,PFDN6,RPL14,HLA-A,MX1,OAS1,RPS18,HMGA1,SNRPE,TAPBP,TFAM,RNF213,RPL27,FARP1,MARCKS,HIST1H4A,ARFIP2

GO:0044248	cellular catabolic process	20	1646	0.45	4.20E-04	9606.ENSP00000258080,9606.ENSP00000268058,9606.ENSP00000270586,9606.ENSP00000290573,9606.ENSP00000296674,9606.ENSP00000333266,9606.ENSP00000339095,9606.ENSP00000346015,9606.ENSP00000363993,9606.ENSP00000364016,9606.ENSP00000377470,9606.ENSP00000379038,9606.ENSP00000379506,9606.ENSP00000386717,9606.ENSP00000393241,9606.ENSP00000398131,9606.ENSP00000416429,9606.ENSP00000464087,9606.ENSP00000464342,9606.ENSP00000464813	HTRA2,PML,PSMB6,HK2,RPS23,UBA7,RPS7,RPL27A,PSMB9,PSMB8,CNP,TYMP,RPL14,RPL31,RPS18,GSPT1,RPL32,RNF213,RPL36,RPL27
GO:0010629	negative regulation of gene expression	20	1670	0.44	5.10E-04	9606.ENSP00000233057,9606.ENSP00000268058,9606.ENSP00000296674,9606.ENSP00000307705,9606.ENSP00000325618,9606.ENSP00000339095,9606.ENSP00000346015,9606.ENSP00000354394,9606.ENSP00000357459,9606.ENSP00000379506,9606.ENSP00000386717,9606.ENSP00000393241,9606.ENSP00000398131,9606.ENSP00000399888,9606.ENSP00000410769,9606.ENSP00000416429,9606.ENSP00000418194,9606.ENSP00000464342,9606.ENSP00000464813,9606.ENSP00000479106	EIF2AK2,PML,RPS23,HIST1H1E,PARP10,RPS7,RPL27A,STAT1,ADAR,RPL14,RPL31,RPS18,GSPT1,HMGA1,CNBP,RPL32,PARP14,RPL36,RPL27,HIST1H4A
GO:0015031	protein transport	18	1391	0.47	5.10E-04	9606.ENSP00000261574,9606.ENSP00000268058,9606.ENSP00000269701,9606.ENSP00000296674,9606.ENSP00000303366,9606.ENSP00000316007,9606.ENSP00000333657,9606.ENSP00000339095,9606.ENSP00000346015,9606.ENSP00000357459,9606.ENSP00000360869,9606.ENSP00000379506,9606.ENSP00000386717,9606.ENSP00000393241,9606.ENSP00000405926,9606.ENSP00000416429,9606.ENSP00000464342,9606.ENSP00000464813	IPO5,PML,AKAP8,RPS23,LMAN2,SCAMP4,MX2,RPS7,RPL27A,ADAR,IFIT1,RPL14,RPL31,RPS18,TMED7,RPL32,RPL36,RPL27
GO:0006139	nucleobase-containing compound metabolic process	37	4551	0.27	5.70E-04	9606.ENSP00000228928,9606.ENSP00000233057,9606.ENSP00000256722,9606.ENSP00000267935,9606.ENSP00000268058,9606.ENSP00000269701,9606.ENSP00000290573,9606.ENSP00000293677,9606.ENSP00000296674,9606.ENSP00000300688,9606.ENSP00000315768,9606.ENSP00000325618,9606.ENSP00000339095,9606.ENSP00000346015,9606.ENSP00000347495,9606.ENSP00000354394,9606.ENSP00000357459,9606.ENSP00000359300,9606.ENSP00000367939,9606.ENSP00000374399,9606.ENSP00000377470,9606.ENSP00000379038,9606.ENSP00000379506,9606.ENSP00000386717,9606.ENSP00000388001,9606.ENSP00000393241,9606.ENSP00000398131,9606.ENSP00000399888,9606.ENSP00000400591,9606.ENSP00000410769,9606.ENSP00000416429,9606.ENSP00000417901,9606.ENSP00000418194,9606.ENSP00000420588,9606.ENSP00000464342,9606.ENSP00000464813,9606.ENSP00000479106	OAS3,EIF2AK2,CMPK2,COMMD4,PML,AKAP8,HK2,RAVER1,RPS23,ATP5L,STAT2,PARP10,RPS7,RPL27A,WARS,STAT1,ADAR,CETN2,UQCRQ,TRMT61A,CNP,TYMP,RPL14,RPL31,OAS1,RPS18,GSPT1,HMGA1,SNRPE,CNBP,RPL32,GPHN,PARP14,TFAM,RPL36,RPL27,HIST1H4A
GO:0071705	nitrogen compound transport	20	1690	0.44	5.80E-04	9606.ENSP00000261574,9606.ENSP00000268058,9606.ENSP00000269701,9606.ENSP00000296674,9606.ENSP00000303366,9606.ENSP00000316007,9606.ENSP00000333657,9606.ENSP00000339095,9606.ENSP00000346015,9606.ENSP00000357459,9606.ENSP00000360869,9606.ENSP00000379506,9606.ENSP00000386717,9606.ENSP00000393241,9606.ENSP00000404833,9606.ENSP00000405926,9606.ENSP00000416429,9606.ENSP00000428845,9606.ENSP00000464342,9606.ENSP00000464813	IPO5,PML,AKAP8,RPS23,LMAN2,SCAMP4,MX2,RPS7,RPL27A,ADAR,IFIT1,RPL14,RPL31,RPS18,TAPBP,TMED7,RPL32,VDAC3,RPL36,RPL27

GO:0010033	response to organic substance	27	2815	0.34	7.10E-04	9606.ENSP00000228928,9606.ENSP00000233057,9606.ENSP00000253110,9606.ENSP00000256722,9606.ENSP00000257570,9606.ENSP00000257604,9606.ENSP00000258080,9606.ENSP00000260118,9606.ENSP00000261574,9606.ENSP00000268058,9606.ENSP00000269701,9606.ENSP00000290573,9606.ENSP00000315768,9606.ENSP00000333657,9606.ENSP00000354394,9606.ENSP00000357459,9606.ENSP00000360869,9606.ENSP00000360883,9606.ENSP00000364016,9606.ENSP00000365402,9606.ENSP00000377470,9606.ENSP00000379873,9606.ENSP00000381601,9606.ENSP00000388001,9606.ENSP00000395590,9606.ENSP00000399168,9606.ENSP00000464813	OAS3,EIF2AK2,C19orf66,CMPK2,OASL,TRAFD1,HTRA2,GGH,IPO5,PML,AKAP8,HK2,STAT2,MX2,STAT1,ADAR,IFIT1,IFIT3,PSMB8,HLA-C,CNP,HLA-A,MX1,OAS1,IFI35,HLA-B,RPL27
GO:0051641	cellular localization	23	2180	0.39	7.50E-04	9606.ENSP00000261517,9606.ENSP00000261574,9606.ENSP00000268058,9606.ENSP00000290573,9606.ENSP00000296674,9606.ENSP00000300688,9606.ENSP00000339095,9606.ENSP00000346015,9606.ENSP00000357459,9606.ENSP00000359300,9606.ENSP00000360869,9606.ENSP00000377390,9606.ENSP00000379506,9606.ENSP00000386717,9606.ENSP00000392985,9606.ENSP00000393241,9606.ENSP00000399888,9606.ENSP00000400591,9606.ENSP00000405926,9606.ENSP00000416429,9606.ENSP00000417901,9606.ENSP00000464342,9606.ENSP00000464813	VPS13C,IPO5,PML,HK2,RPS23,ATP5L,RPS7,RPL27A,ADAR,CETN2,IFIT1,PPP6R3,RPL14,RPL31,HTATIP2,RPS18,HMGA1,SNRPE,TMED7,RPL32,GPHN,RPL36,RPL27
GO:0044271	cellular nitrogen compound biosynthetic process	31	3528	0.31	8.10E-04	9606.ENSP00000233057,9606.ENSP00000256722,9606.ENSP00000267935,9606.ENSP00000268058,9606.ENSP00000269701,9606.ENSP00000290573,9606.ENSP00000296674,9606.ENSP00000300688,9606.ENSP00000315768,9606.ENSP00000325618,9606.ENSP00000339095,9606.ENSP00000346015,9606.ENSP00000347495,9606.ENSP00000354394,9606.ENSP00000356978,9606.ENSP00000377470,9606.ENSP00000379038,9606.ENSP00000379506,9606.ENSP00000386717,9606.ENSP00000388001,9606.ENSP00000393241,9606.ENSP00000398131,9606.ENSP00000399888,9606.ENSP00000400591,9606.ENSP00000410769,9606.ENSP00000416429,9606.ENSP00000418194,9606.ENSP00000420588,9606.ENSP00000464342,9606.ENSP00000464813,9606.ENSP00000479106	EIF2AK2,CMPK2,COMMD4,PML,AKAP8,HK2,RPS23,ATP5L,STAT2,PARP10,RPS7,RPL27A,WARS,STAT1,PPOX,CNP,TYMP,RPL14,RPL31,OAS1,RPS18,GSPT1,HMGA1,SNRPE,CNBP,RPL32,PARP14,TFAM,RPL36,RPL27,HIST1H4A
GO:0006886	intracellular protein transport	13	836	0.55	1.20E-03	9606.ENSP00000261574,9606.ENSP00000268058,9606.ENSP00000296674,9606.ENSP00000339095,9606.ENSP00000346015,9606.ENSP00000357459,9606.ENSP00000360869,9606.ENSP00000379506,9606.ENSP00000386717,9606.ENSP00000393241,9606.ENSP00000416429,9606.ENSP00000464342,9606.ENSP00000464813	IPO5,PML,RPS23,RPS7,RPL27A,ADAR,IFIT1,RPL14,RPL31,RPS18,RPL32,RPL36,RPL27
GO:0019882	antigen processing and presentation	6	158	0.94	1.30E-03	9606.ENSP00000289228,9606.ENSP00000364016,9606.ENSP00000365402,9606.ENSP00000379873,9606.ENSP00000399168,9606.ENSP00000404833	ACTR1B,PSMB8,HLA-C,HLA-A,HLA-B,TAPBP

GO:0008104	protein localization	21	1966	0.39	1.40E-03	9606.ENSP00000261574,9606.ENSP00000268058,9606.ENSP00000269701,9606.ENSP00000290573,9606.ENSP00000296674,9606.ENSP00000303366,9606.ENSP00000316007,9606.ENSP00000333657,9606.ENSP00000339095,9606.ENSP00000346015,9606.ENSP00000357459,9606.ENSP00000360869,9606.ENSP00000363624,9606.ENSP00000379506,9606.ENSP00000386717,9606.ENSP00000393241,9606.ENSP00000405926,9606.ENSP00000416429,9606.ENSP00000417901,9606.ENSP00000464342,9606.ENSP00000464813	IPO5,PML,AKAP8,HK2,RPS23,LMAN2,SCAMP4,MX2,RPS7,RPL27A,ADAR,IFIT1,CUTA,RPL14,RPL31,RPS18,TMED7,RPL32,GPHN,RPL36,RPL27
GO:0002831	regulation of response to biotic stimulus	9	426	0.69	1.80E-03	9606.ENSP00000251642,9606.ENSP00000257604,9606.ENSP00000268058,9606.ENSP00000354394,9606.ENSP00000357459,9606.ENSP00000360869,9606.ENSP00000379873,9606.ENSP00000399168,9606.ENSP00000418194	DHX58,TRAFD1,PML,STAT1,ADAR,IFIT1,HLA-A,HLA-B,PARP14
GO:0007005	mitochondrion organization	9	424	0.69	1.80E-03	9606.ENSP00000258080,9606.ENSP00000261517,9606.ENSP00000290573,9606.ENSP00000300688,9606.ENSP00000333657,9606.ENSP00000377470,9606.ENSP00000379038,9606.ENSP00000381601,9606.ENSP00000420588	HTRA2,VPS13C,HK2,ATP5L,MX2,CNP,TYMP,MX1,TFAM
GO:0051179	localization	39	5233	0.23	2.00E-03	9606.ENSP00000249066,9606.ENSP00000260118,9606.ENSP00000261517,9606.ENSP00000261574,9606.ENSP00000262776,9606.ENSP00000268058,9606.ENSP00000269701,9606.ENSP00000289228,9606.ENSP00000290573,9606.ENSP00000296674,9606.ENSP00000300688,9606.ENSP00000303366,9606.ENSP00000316007,9606.ENSP00000333657,9606.ENSP00000339095,9606.ENSP00000346015,9606.ENSP00000355330,9606.ENSP00000357459,9606.ENSP00000359300,9606.ENSP00000360869,9606.ENSP00000363624,9606.ENSP00000365402,9606.ENSP00000377390,9606.ENSP00000377470,9606.ENSP00000379506,9606.ENSP00000379873,9606.ENSP00000386717,9606.ENSP00000392985,9606.ENSP00000393241,9606.ENSP00000399168,9606.ENSP00000399888,9606.ENSP00000400591,9606.ENSP00000404833,9606.ENSP00000405926,9606.ENSP00000416429,9606.ENSP00000417901,9606.ENSP00000428845,9606.ENSP00000464342,9606.ENSP00000464813	APOL2,GGH,VPS13C,IPO5,LGALS3BP,PML,AKAP8,ACTR1B,HK2,RPS23,ATP5L,LMAN2,SCAMP4,MX2,RPS7,RPL27A,TGM2,ADAR,CETN2,IFIT1,CUTA,HLA-C,PPP6R3,CNP,RPL14,HLA-A,RPL31,HTATIP2,RPS18,HLA-B,HMGA1,SNRPE,TAPBP,TMED7,RPL32,GPHN,VDAC3,RPL36,RPL27
GO:1901564	organonitrogen compound metabolic process	39	5281	0.23	2.50E-03	9606.ENSP00000225719,9606.ENSP00000226299,9606.ENSP00000233057,9606.ENSP00000249066,9606.ENSP00000256722,9606.ENSP00000258080,9606.ENSP00000260118,9606.ENSP00000268058,9606.ENSP00000270586,9606.ENSP00000290573,9606.ENSP00000296674,9606.ENSP00000300688,9606.ENSP00000307705,9606.ENSP00000325618,9606.ENSP00000333266,9606.ENSP00000339095,9606.ENSP00000346015,9606.ENSP00000347495,9606.ENSP00000355330,9606.ENSP00000356978,9606.ENSP00000363993,9606.ENSP00000364016,9606.ENSP00000367939,9606.ENSP00000379038,9606.ENSP00000379506,9606.ENSP00000379873,9606.ENSP00000386717,9606.ENSP00000388001,9606.ENSP00000393241,9606.ENSP00000398131,9606.ENSP00000404833,9606.ENSP00000416429,9606.ENSP00000417901,9606.ENSP00000418194,9606.ENSP00000464036,9606.ENSP00000464087,9606.ENSP00000464342,9606.ENSP00000464813,9606.ENSP00000479106	CPD,LAP3,EIF2AK2,APOL2,CMPK2,HTRA2,GGH,PML,PSMB6,HK2,RPS23,ATP5L,HIST1H1E,PARP10,UBA7,RPS7,RPL27A,WARS,TGM2,PPOX,PSMB9,PSMB8,UQCRQ,TYMP,RPL14,HLA-A,RPL31,OAS1,RPS18,GSPT1,TAPBP,RPL32,GPHN,PARP14,CDK11B,RNF213,RPL36,RPL27,HIST1H4A

GO:0071310	cellular response to organic substance	22	2219	0.36	2.60E-03	9606.ENSP00000228928,9606.ENSP00000233057,9606.ENSP00000256722,9606.ENSP00000257570,9606.ENSP00000258080,9606.ENSP00000261574,9606.ENSP00000268058,9606.ENSP00000269701,9606.ENSP00000290573,9606.ENSP00000315768,9606.ENSP00000333657,9606.ENSP00000354394,9606.ENSP00000357459,9606.ENSP00000360869,9606.ENSP00000360883,9606.ENSP00000364016,9606.ENSP00000365402,9606.ENSP00000379873,9606.ENSP00000381601,9606.ENSP00000388001,9606.ENSP00000395590,9606.ENSP00000399168	OAS3,EIF2AK2,CMPK2,OASL,HTRA2,IPO5,PML,AKAP8,HK2,STAT2,MX2,STAT1,ADAR,IFIT1,IFIT3,PSMB8,HLA-C,HLA-A,MX1,OAS1,IFI35,HLA-B
GO:0006807	nitrogen compound metabolic process	53	8349	0.16	3.30E-03	9606.ENSP00000225719,9606.ENSP00000226299,9606.ENSP00000228928,9606.ENSP00000233057,9606.ENSP00000249066,9606.ENSP00000256722,9606.ENSP00000258080,9606.ENSP00000260118,9606.ENSP00000267935,9606.ENSP00000268058,9606.ENSP00000269701,9606.ENSP00000270586,9606.ENSP00000290573,9606.ENSP00000293677,9606.ENSP00000296674,9606.ENSP00000300688,9606.ENSP00000307705,9606.ENSP00000315768,9606.ENSP00000325618,9606.ENSP00000333266,9606.ENSP00000339095,9606.ENSP00000346015,9606.ENSP00000347495,9606.ENSP00000354394,9606.ENSP00000355330,9606.ENSP00000356978,9606.ENSP00000357459,9606.ENSP00000359300,9606.ENSP00000363993,9606.ENSP00000364016,9606.ENSP00000367939,9606.ENSP00000374399,9606.ENSP00000377470,9606.ENSP00000379038,9606.ENSP00000379506,9606.ENSP00000379873,9606.ENSP00000386717,9606.ENSP00000388001,9606.ENSP00000393241,9606.ENSP00000398131,9606.ENSP00000399888,9606.ENSP00000400591,9606.ENSP00000404833,9606.ENSP00000410769,9606.ENSP00000416429,9606.ENSP00000417901,9606.ENSP00000418194,9606.ENSP00000420588,9606.ENSP00000464036,9606.ENSP00000464087,9606.ENSP00000464342,9606.ENSP00000464813,9606.ENSP00000479106	CPD,LAP3,OAS3,EIF2AK2,APOL2,CMPK2,HTRA2,GGH,COMMD4,PML,AKAP8,PSMB6,HK2,RAVER1,RPS23,ATP5L,HIST1H1E,STAT2,PARP10,UBA7,RPS7,RPL27A,WARS,STAT1,TGM2,PPOX,ADAR,CETN2,PSMB9,PSMB8,UQCRQ,TRMT61A,CNP,TYMP,RPL14,HLA-A,RPL31,OAS1,RPS18,GSPT1,HMGA1,SNRPE,TAPBP,CNBP,RPL32,GPHN,PARP14,TFAM,CDK11B,RNF213,RPL36,RPL27,HIST1H4A
GO:0009892	negative regulation of metabolic process	25	2762	0.32	3.40E-03	9606.ENSP00000233057,9606.ENSP00000258080,9606.ENSP00000261574,9606.ENSP00000268058,9606.ENSP00000290573,9606.ENSP00000296674,9606.ENSP00000307705,9606.ENSP00000325618,9606.ENSP00000339095,9606.ENSP00000346015,9606.ENSP00000347495,9606.ENSP00000354394,9606.ENSP00000357459,9606.ENSP00000379506,9606.ENSP00000386717,9606.ENSP00000393241,9606.ENSP00000398131,9606.ENSP00000399888,9606.ENSP00000410769,9606.ENSP00000416429,9606.ENSP00000418194,9606.ENSP00000464342,9606.ENSP00000464813,9606.ENSP00000471242,9606.ENSP00000479106	EIF2AK2,HTRA2,IPO5,PML,HK2,RPS23,HIST1H1E,PARP10,RPS7,RPL27A,WARS,STAT1,ADAR,RPL14,RPL31,RPS18,GSPT1,HMGA1,CNBP,RPL32,PARP14,RPL36,RPL27,FARP1,HIST1H4A
GO:0033036	macromolecule localization	22	2268	0.35	3.50E-03	9606.ENSP00000249066,9606.ENSP00000261574,9606.ENSP00000268058,9606.ENSP00000269701,9606.ENSP00000290573,9606.ENSP00000296674,9606.ENSP00000303366,9606.ENSP00000316007,9606.ENSP00000333657,9606.ENSP00000339095,9606.ENSP00000346015,9606.ENSP00000357459,9606.ENSP00000360869,9606.ENSP00000363624,9606.ENSP00000379506,9606.ENSP00000386717,9606.ENSP00000393241,9606.ENSP00000405926,9606.ENSP00000416429,9606.ENSP00000417901,9606.ENSP00000464342,9606.ENSP00000464813	APOL2,IPO5,PML,AKAP8,HK2,RPS23,LMAN2,SCAMP4,MX2,RPS7,RPL27A,ADAR,IFIT1,CUTA,RPL14,RPL31,RPS18,TMED7,RPL32,GPHN,RPL36,RPL27

GO:0051170	import into nucleus	5	122	0.97	3.50E-03	9606.ENSP00000261574,9606.ENSP00000268058,9606.ENSP00000357459,9606.ENSP00000392985,9606.ENSP00000400591	IPO5,PML,ADAR,HTATIP2,SNRPE
GO:0002478	antigen processing and presentation of exogenous peptide antigen	5	123	0.97	3.60E-03	9606.ENSP00000289228,9606.ENSP00000365402,9606.ENSP00000379873,9606.ENSP00000399168,9606.ENSP00000404833	ACTR1B,HLA-C,HLA-A,HLA-B,TAPBP
GO:0050688	regulation of defense response to virus	4	71	1.11	4.80E-03	9606.ENSP00000251642,9606.ENSP00000268058,9606.ENSP00000354394,9606.ENSP00000360869	DHX58,PML,STAT1,IFIT1
GO:0022607	cellular component assembly	22	2343	0.33	5.20E-03	9606.ENSP00000258080,9606.ENSP00000268058,9606.ENSP00000296674,9606.ENSP00000307705,9606.ENSP00000333657,9606.ENSP00000355330,9606.ENSP00000357459,9606.ENSP00000359300,9606.ENSP00000377390,9606.ENSP00000378563,9606.ENSP00000379873,9606.ENSP00000381601,9606.ENSP00000388001,9606.ENSP00000399888,9606.ENSP00000400591,9606.ENSP00000404833,9606.ENSP00000420588,9606.ENSP00000464087,9606.ENSP00000471242,9606.ENSP00000478061,9606.ENSP00000479106,9606.ENSP00000484121	HTRA2,PML,RPS23,HIST1H1E,MX2,TGM2,ADAR,CETN2,PPP6R3,PFDN6,HLA-A,MX1,OAS1,HMGA1,SNRPE,TAPBP,TFAM,RNF213,FARP1,MARCKS,HIST1H4A,ARFIP2
GO:0042270	protection from natural killer cell mediated cytotoxicity	2	5	1.96	5.70E-03	9606.ENSP00000379873,9606.ENSP00000399168	HLA-A,HLA-B
GO:0070212	protein poly-ADP-ribosylation	2	5	1.96	5.70E-03	9606.ENSP00000325618,9606.ENSP00000418194	PARP10,PARP14
GO:0071702	organic substance transport	20	2040	0.35	5.90E-03	9606.ENSP00000249066,9606.ENSP00000261574,9606.ENSP00000268058,9606.ENSP00000269701,9606.ENSP00000296674,9606.ENSP00000303366,9606.ENSP00000316007,9606.ENSP00000333657,9606.ENSP00000339095,9606.ENSP00000346015,9606.ENSP00000357459,9606.ENSP00000360869,9606.ENSP00000379506,9606.ENSP00000386717,9606.ENSP00000393241,9606.ENSP00000405926,9606.ENSP00000416429,9606.ENSP00000428845,9606.ENSP00000464342,9606.ENSP00000464813	APOL2,IPO5,PML,AKAP8,RPS23,LMAN2,SCAMP4,MX2,RPS7,RPL27A,ADAR,IFIT1,RPL14,RPL31,RPS18,TMED7,RPL32,VDAC3,RPL36,RPL27
GO:0032102	negative regulation of response to external stimulus	7	310	0.72	6.50E-03	9606.ENSP00000251642,9606.ENSP00000257604,9606.ENSP00000357459,9606.ENSP00000360869,9606.ENSP00000379873,9606.ENSP00000399168,9606.ENSP00000418194	DHX58,TRAFD1,ADAR,IFIT1,HLA-A,HLA-B,PARP14
GO:0002683	negative regulation of immune system process	8	425	0.64	8.20E-03	9606.ENSP00000251642,9606.ENSP00000257604,9606.ENSP00000357459,9606.ENSP00000360869,9606.ENSP00000379873,9606.ENSP00000399168,9606.ENSP00000418194,9606.ENSP00000479106	DHX58,TRAFD1,ADAR,IFIT1,HLA-A,HLA-B,PARP14,HIST1H4A
GO:1903146	regulation of autophagy of mitochondrion	3	35	1.3	8.40E-03	9606.ENSP00000258080,9606.ENSP00000261517,9606.ENSP00000290573	HTRA2,VPS13C,HK2
GO:0003374	dynamin family protein polymerization involved in mitochondrial fission	2	7	1.82	9.20E-03	9606.ENSP00000333657,9606.ENSP00000381601	MX2,MX1
GO:0060700	regulation of ribonuclease activity	2	7	1.82	9.20E-03	9606.ENSP00000228928,9606.ENSP00000388001	OAS3,OAS1
GO:0031347	regulation of defense response	10	676	0.53	1.03E-02	9606.ENSP00000251642,9606.ENSP00000257604,9606.ENSP00000268058,9606.ENSP00000354394,9606.ENSP00000355330,9606.ENSP00000357459,9606.ENSP00000360869,9606.ENSP00000379873,9606.ENSP00000399168,9606.ENSP00000418194	DHX58,TRAFD1,PML,STAT1,TGM2,ADAR,IFIT1,HLA-A,HLA-B,PARP14
GO:0034613	cellular protein localization	15	1367	0.4	1.09E-02	9606.ENSP00000261574,9606.ENSP00000268058,9606.ENSP00000290573,9606.ENSP00000296674,9606.ENSP00000339095,9606.ENSP00000346015,9606.ENSP00000357459,9606.ENSP00000360869,9606.ENSP00000379506,9606.ENSP00000386717,9606.ENSP00000393241,9606.ENSP00000416429,9606.ENSP00000417901,9606.ENSP00000464342,9606.ENSP00000464813	IPO5,PML,HK2,RPS23,RPS7,RPL27A,ADAR,IFIT1,RPL14,RPL31,RPS18,RPL32,GPHN,RPL36,RPL27

GO:0050691	regulation of defense response to virus by host	3	39	1.25	1.09E-02	9606.ENSP00000268058,9606.ENSP00000354394,9606.ENSP00000360869	PML,STAT1,IFIT1
GO:0046719	regulation by virus of viral protein levels in host cell	2	8	1.76	1.10E-02	9606.ENSP00000354394,9606.ENSP00000360869	STAT1,IFIT1
GO:0042221	response to chemical	31	4153	0.24	1.25E-02	9606.ENSP00000228928,9606.ENSP00000233057,9606.ENSP00000253110,9606.ENSP00000256722,9606.ENSP00000257570,9606.ENSP00000257604,9606.ENSP00000258080,9606.ENSP00000260118,9606.ENSP00000261574,9606.ENSP00000268058,9606.ENSP00000269701,9606.ENSP00000290573,9606.ENSP00000315768,9606.ENSP00000333657,9606.ENSP00000354394,9606.ENSP00000356978,9606.ENSP00000357459,9606.ENSP00000360869,9606.ENSP00000360883,9606.ENSP00000363624,9606.ENSP00000364016,9606.ENSP00000365402,9606.ENSP00000377470,9606.ENSP00000379038,9606.ENSP00000379873,9606.ENSP00000381601,9606.ENSP00000388001,9606.ENSP00000395590,9606.ENSP00000399168,9606.ENSP00000417901,9606.ENSP00000464813	OAS3,EIF2AK2,C19orf66,CMPK2,OASL,TRAFD1,HTRA2,GGH,IPO5,PML,AKAP8,HK2,STAT2,MX2,STAT1,PPOX,ADAR,IFIT1,IFIT3,CUTA,PSMB8,HLA-C,CNP,TYMP,HLA-A,MX1,OAS1,IFI35,HLA-B,GPHN,RPL27
GO:0045088	regulation of innate immune response	7	361	0.65	1.42E-02	9606.ENSP00000251642,9606.ENSP00000257604,9606.ENSP00000354394,9606.ENSP00000357459,9606.ENSP00000379873,9606.ENSP00000399168,9606.ENSP00000418194	DHX58,TRAFD1,STAT1,ADAR,HLA-A,HLA-B,PARP14
GO:0019538	protein metabolic process	31	4194	0.23	1.45E-02	9606.ENSP00000225719,9606.ENSP00000226299,9606.ENSP00000233057,9606.ENSP00000249066,9606.ENSP00000258080,9606.ENSP00000260118,9606.ENSP00000268058,9606.ENSP00000270586,9606.ENSP00000296674,9606.ENSP00000307705,9606.ENSP00000325618,9606.ENSP00000333266,9606.ENSP00000339095,9606.ENSP00000346015,9606.ENSP00000347495,9606.ENSP00000355330,9606.ENSP00000363993,9606.ENSP00000364016,9606.ENSP00000379506,9606.ENSP00000379873,9606.ENSP00000386717,9606.ENSP00000393241,9606.ENSP00000398131,9606.ENSP00000416429,9606.ENSP00000417901,9606.ENSP00000418194,9606.ENSP00000464036,9606.ENSP00000464087,9606.ENSP00000464342,9606.ENSP00000464813,9606.ENSP00000479106	CPD,LAP3,EIF2AK2,APOL2,HTRA2,GGH,PML,PSMB6,RPS23,HIST1H1E,PARP10,UBA7,RPS7,RPL27A,WARS,TGM2,PSMB9,PSMB8,RPL14,HLA-A,RPL31,RPS18,GSPT1,RPL32,GPHN,PARP14,CDK11B,RNF213,RPL36,RPL27,HIST1H4A
GO:0010605	negative regulation of macromolecule metabolic process	22	2558	0.3	1.50E-02	9606.ENSP00000233057,9606.ENSP00000261574,9606.ENSP00000268058,9606.ENSP00000296674,9606.ENSP00000307705,9606.ENSP00000325618,9606.ENSP00000339095,9606.ENSP00000346015,9606.ENSP00000347495,9606.ENSP00000354394,9606.ENSP00000357459,9606.ENSP00000379506,9606.ENSP00000386717,9606.ENSP00000393241,9606.ENSP00000398131,9606.ENSP00000399888,9606.ENSP00000410769,9606.ENSP00000416429,9606.ENSP00000418194,9606.ENSP00000464342,9606.ENSP00000464813,9606.ENSP00000479106	EIF2AK2,IPO5,PML,RPS23,HIST1H1E,PARP10,RPS7,RPL27A,WARS,STAT1,ADAR,RPL14,RPL31,RPS18,GSPT1,HMGA1,CNBP,RPL32,PARP14,RPL36,RPL27,HIST1H4A
GO:0051169	nuclear transport	6	267	0.71	1.53E-02	9606.ENSP00000261574,9606.ENSP00000268058,9606.ENSP00000357459,9606.ENSP00000392985,9606.ENSP00000399888,9606.ENSP00000400591	IPO5,PML,ADAR,HTATIP2,HMGA1,SNRPE

GO:0080134	regulation of response to stress	14	1299	0.39	1.84E-02	9606.ENSP00000233057,9606.ENSP00000251642,9606.ENSP00000257604,9606.ENSP00000258080,9606.ENSP00000261517,9606.ENSP00000268058,9606.ENSP00000354394,9606.ENSP00000355330,9606.ENSP00000357459,9606.ENSP00000360869,9606.ENSP00000379873,9606.ENSP00000399168,9606.ENSP00000399888,9606.ENSP00000418194	EIF2AK2,DHX58,TRAFD1,HTRA2,VPS13C,PML,STAT1,TGM2,ADAR,IFIT1,HLA-A,HLA-B,HMGA1,PARP14
GO:0035457	cellular response to interferon-alpha	2	12	1.58	2.07E-02	9606.ENSP00000360883,9606.ENSP00000388001	IFIT3,OAS1
GO:0002698	negative regulation of immune effector process	4	113	0.91	2.09E-02	9606.ENSP00000251642,9606.ENSP00000360869,9606.ENSP00000379873,9606.ENSP00000399168	DHX58,IFIT1,HLA-A,HLA-B
GO:0010467	gene expression	28	3733	0.24	2.17E-02	9606.ENSP00000225719,9606.ENSP00000233057,9606.ENSP00000258080,9606.ENSP00000267935,9606.ENSP00000268058,9606.ENSP00000269701,9606.ENSP00000293677,9606.ENSP00000296674,9606.ENSP00000315768,9606.ENSP00000339095,9606.ENSP00000346015,9606.ENSP00000347495,9606.ENSP00000354394,9606.ENSP00000357459,9606.ENSP00000374399,9606.ENSP00000379506,9606.ENSP00000386717,9606.ENSP00000393241,9606.ENSP00000398131,9606.ENSP00000399888,9606.ENSP00000400591,9606.ENSP00000410769,9606.ENSP00000416429,9606.ENSP00000418194,9606.ENSP00000420588,9606.ENSP00000464342,9606.ENSP00000464813,9606.ENSP00000479106	CPD,EIF2AK2,HTRA2,COMMD4,PML,AKAP8,RAVER1,RPS23,STAT2,RPS7,RPL27A,WARS,STAT1,ADAR,TRMT61A,RPL14,RPL31,RPS18,GSPT1,HMGA1,SNRPE,CNBP,RPL32,PARP14,TFAM,RPL36,RPL27,HIST1H4A
GO:1904923	regulation of autophagy of mitochondrion in response to mitochondrial depolarization	2	13	1.55	2.31E-02	9606.ENSP00000258080,9606.ENSP00000290573	HTRA2,HK2
GO:0022613	ribonucleoprotein complex biogenesis	7	409	0.6	2.61E-02	9606.ENSP00000296674,9606.ENSP00000339095,9606.ENSP00000357459,9606.ENSP00000379506,9606.ENSP00000393241,9606.ENSP00000400591,9606.ENSP00000464813	RPS23,RPS7,ADAR,RPL14,RPS18,SNRPE,RPL27
GO:0044249	cellular biosynthetic process	32	4567	0.21	2.65E-02	9606.ENSP00000233057,9606.ENSP00000256722,9606.ENSP00000267935,9606.ENSP00000268058,9606.ENSP00000269701,9606.ENSP00000290573,9606.ENSP00000296674,9606.ENSP00000300688,9606.ENSP00000315768,9606.ENSP00000325618,9606.ENSP00000339095,9606.ENSP00000346015,9606.ENSP00000347495,9606.ENSP00000354394,9606.ENSP00000356978,9606.ENSP00000377470,9606.ENSP00000379038,9606.ENSP00000379506,9606.ENSP00000386717,9606.ENSP00000388001,9606.ENSP00000393241,9606.ENSP00000398131,9606.ENSP00000399888,9606.ENSP00000400591,9606.ENSP00000410769,9606.ENSP00000416429,9606.ENSP00000417901,9606.ENSP00000418194,9606.ENSP00000420588,9606.ENSP00000464342,9606.ENSP00000464813,9606.ENSP00000479106	EIF2AK2,CMPK2,COMMD4,PML,AKAP8,HK2,RPS23,ATP5L,STAT2,PARP10,RPS7,RPL27A,WARS,STAT1,PPOX,CNP,TYMP,RPL14,RPL31,OAS1,RPS18,GSPT1,HMGA1,SNRPE,CNBP,RPL32,GPHN,PARP14,TFAM,RPL36,RPL27,HIST1H4A

GO:0044267	cellular protein metabolic process	27	3603	0.24	2.65E-02	9606.ENSP00000233057,9606.ENSP00000258080,9606.ENSP00000268058,9606.ENSP00000270586,9606.ENSP00000296674,9606.ENSP00000307705,9606.ENSP00000325618,9606.ENSP00000333266,9606.ENSP00000339095,9606.ENSP00000346015,9606.ENSP00000347495,9606.ENSP00000355330,9606.ENSP00000363993,9606.ENSP00000364016,9606.ENSP00000379506,9606.ENSP00000379873,9606.ENSP00000386717,9606.ENSP00000393241,9606.ENSP00000398131,9606.ENSP00000416429,9606.ENSP00000417901,9606.ENSP00000418194,9606.ENSP00000464036,9606.ENSP00000464087,9606.ENSP00000464342,9606.ENSP00000464813,9606.ENSP00000479106	EIF2AK2,HTRA2,PML,PSMB6,RPS23,HIST1H1E,PARP10,UBA7,RPS7,RPL27A,WARS,TGM2,PSMB9,PSMB8,RPL14,HLA-A,RPL31,RPS18,GSPT1,RPL32,GPHN,PARP14,CDK11B,RNF213,RPL36,RPL27,HIST1H4A
GO:0016070	RNA metabolic process	26	3430	0.24	2.77E-02	9606.ENSP00000233057,9606.ENSP00000267935,9606.ENSP00000268058,9606.ENSP00000269701,9606.ENSP00000293677,9606.ENSP00000296674,9606.ENSP00000315768,9606.ENSP00000339095,9606.ENSP00000346015,9606.ENSP00000347495,9606.ENSP00000354394,9606.ENSP00000357459,9606.ENSP00000374399,9606.ENSP00000379506,9606.ENSP00000386717,9606.ENSP00000393241,9606.ENSP00000398131,9606.ENSP00000399888,9606.ENSP00000400591,9606.ENSP00000410769,9606.ENSP00000416429,9606.ENSP00000418194,9606.ENSP00000420588,9606.ENSP00000464342,9606.ENSP00000464813,9606.ENSP00000479106	EIF2AK2,COMMD4,PML,AKAP8,RAVER1,RPS23,STAT2,RPS7,RPL27A,WARS,STAT1,ADAR,TRMT61A,RPL14,RPL31,RPS18,GSPT1,HMGA1,SNRPE,CNBP,RPL32,PARP14,TFAM,RPL36,RPL27,HIST1H4A
GO:0016045	detection of bacterium	2	15	1.49	2.83E-02	9606.ENSP00000379873,9606.ENSP00000399168	HLA-A,HLA-B
GO:0035458	cellular response to interferon-beta	2	15	1.49	2.83E-02	9606.ENSP00000258080,9606.ENSP00000354394	HTRA2,STAT1
GO:0055086	nucleobase-containing small molecule metabolic process	9	662	0.5	2.83E-02	9606.ENSP00000256722,9606.ENSP00000290573,9606.ENSP00000300688,9606.ENSP00000325618,9606.ENSP00000367939,9606.ENSP00000377470,9606.ENSP00000379038,9606.ENSP00000388001,9606.ENSP00000417901	CMPK2,HK2,ATP5L,PARP10,UQCRQ,CNP,TYMP,OAS1,GPHN
GO:1901524	regulation of mitophagy	2	15	1.49	2.83E-02	9606.ENSP00000258080,9606.ENSP00000261517	HTRA2,VPS13C

GO:0071704	organic substance metabolic process	53	9135	0.13	3.09E-02	9606.ENSP00000225719,9606.ENSP00000226299,9606.ENSP00000228928,9606.ENSP00000233057,9606.ENSP00000249066,9606.ENSP00000256722,9606.ENSP00000258080,9606.ENSP00000260118,9606.ENSP00000267935,9606.ENSP00000268058,9606.ENSP00000269701,9606.ENSP00000270586,9606.ENSP00000290573,9606.ENSP00000293677,9606.ENSP00000296674,9606.ENSP00000300688,9606.ENSP00000307705,9606.ENSP00000315768,9606.ENSP00000325618,9606.ENSP00000333266,9606.ENSP00000339095,9606.ENSP00000346015,9606.ENSP00000347495,9606.ENSP00000354394,9606.ENSP00000355330,9606.ENSP00000356978,9606.ENSP00000357459,9606.ENSP00000359300,9606.ENSP00000363993,9606.ENSP00000364016,9606.ENSP00000367939,9606.ENSP00000374399,9606.ENSP00000377470,9606.ENSP00000379038,9606.ENSP00000379506,9606.ENSP00000379873,9606.ENSP00000386717,9606.ENSP00000388001,9606.ENSP00000393241,9606.ENSP00000398131,9606.ENSP00000399888,9606.ENSP00000400591,9606.ENSP00000404833,9606.ENSP00000410769,9606.ENSP00000416429,9606.ENSP00000417901,9606.ENSP00000418194,9606.ENSP00000420588,9606.ENSP00000464036,9606.ENSP00000464087,9606.ENSP00000464342,9606.ENSP00000464813,9606.ENSP00000479106	CPD,LAP3,OAS3,EIF2AK2,APOL2,CMPK2,HTRA2,GGH,COMMD4,PML,AKAP8,PSMB6,HK2,RAVER1,RPS23,ATP5L,HIST1H1E,STAT2,PARP10,UBA7,RPS7,RPL27A,WARS,STAT1,TGM2,PPOX,ADAR,CETN2,PSMB9,PSMB8,UQCRQ,TRMT61A,CNP,TYMP,RPL14,HLA-A,RPL31,OAS1,RPS18,GSPT1,HMGA1,SNRPE,TAPBP,CNBP,RPL32,GPHN,PARP14,TFAM,CDK11B,RNF213,RPL36,RPL27,HIST1H4A
GO:0001916	positive regulation of T cell mediated cytotoxicity	2	17	1.43	3.40E-02	9606.ENSP00000379873,9606.ENSP00000399168	HLA-A,HLA-B
GO:1901576	organic substance biosynthetic process	32	4656	0.2	3.42E-02	9606.ENSP00000233057,9606.ENSP00000256722,9606.ENSP00000267935,9606.ENSP00000268058,9606.ENSP00000269701,9606.ENSP00000290573,9606.ENSP00000296674,9606.ENSP00000300688,9606.ENSP00000315768,9606.ENSP00000325618,9606.ENSP00000339095,9606.ENSP00000346015,9606.ENSP00000347495,9606.ENSP00000354394,9606.ENSP00000356978,9606.ENSP00000377470,9606.ENSP00000379038,9606.ENSP00000379506,9606.ENSP00000386717,9606.ENSP00000388001,9606.ENSP00000393241,9606.ENSP00000398131,9606.ENSP00000399888,9606.ENSP00000400591,9606.ENSP00000410769,9606.ENSP00000416429,9606.ENSP00000417901,9606.ENSP00000418194,9606.ENSP00000420588,9606.ENSP00000464342,9606.ENSP00000464813,9606.ENSP00000479106	EIF2AK2,CMPK2,COMMD4,PML,AKAP8,HK2,RPS23,ATP5L,STAT2,PARP10,RPS7,RPL27A,WARS,STAT1,PPOX,CNP,TYMP,RPL14,RPL31,OAS1,RPS18,GSPT1,HMGA1,SNRPE,CNBP,RPL32,GPHN,PARP14,TFAM,RPL36,RPL27,HIST1H4A
GO:0043555	regulation of translation in response to stress	2	18	1.41	3.70E-02	9606.ENSP00000233057,9606.ENSP00000268058	EIF2AK2,PML
GO:0034622	cellular protein-containing complex assembly	10	832	0.44	3.73E-02	9606.ENSP00000296674,9606.ENSP00000307705,9606.ENSP00000333657,9606.ENSP00000357459,9606.ENSP00000359300,9606.ENSP00000378563,9606.ENSP00000381601,9606.ENSP00000400591,9606.ENSP00000420588,9606.ENSP00000479106	RPS23,HIST1H1E,MX2,ADAR,CETN2,PFDN6,MX1,SNRPE,TFAM,HIST1H4A
GO:0000266	mitochondrial fission	2	19	1.38	4.03E-02	9606.ENSP00000333657,9606.ENSP00000381601	MX2,MX1
GO:0031065	positive regulation of histone deacetylation	2	19	1.38	4.03E-02	9606.ENSP00000268058,9606.ENSP00000269701	PML,AKAP8
GO:0009117	nucleotide metabolic process	8	576	0.5	4.04E-02	9606.ENSP00000256722,9606.ENSP00000290573,9606.ENSP00000300688,9606.ENSP00000325618,9606.ENSP00000367939,9606.ENSP00000377470,9606.ENSP00000388001,9606.ENSP00000417901	CMPK2,HK2,ATP5L,PARP10,UQCRQ,CNP,OAS1,GPHN

GO:0044237	cellular metabolic process	51	8797	0.13	4.09E-02	9606.ENSP00000225719,9606.ENSP00000228928,9606.ENSP00000233057,9606.ENSP00000256722,9606.ENSP00000258080,9606.ENSP00000260118,9606.ENSP00000267935,9606.ENSP00000268058,9606.ENSP00000269701,9606.ENSP00000270586,9606.ENSP00000290573,9606.ENSP00000293677,9606.ENSP00000296674,9606.ENSP00000300688,9606.ENSP00000307705,9606.ENSP00000315768,9606.ENSP00000325618,9606.ENSP00000333266,9606.ENSP00000339095,9606.ENSP00000346015,9606.ENSP00000347495,9606.ENSP00000354394,9606.ENSP00000355330,9606.ENSP00000356978,9606.ENSP00000357459,9606.ENSP00000359300,9606ENSP00000363993,9606.ENSP00000364016,9606.ENSP00000367939,9606.ENSP00000374399,9606.ENSP00000377470,9606.ENSP00000379038,9606.ENSP00000379506,9606.ENSP00000379873,9606.ENSP00000386717,9606.ENSP00000388001,9606.ENSP00000393241,9606.ENSP00000398131,9606.ENSP00000399888,9606.ENSP00000400591,9606.ENSP00000404833,9606.ENSP00000410769,9606.ENSP00000416429,9606.ENSP00000417901,9606.ENSP00000418194,9606.ENSP00000420588,9606.ENSP00000464036,9606.ENSP00000464087,9606.ENSP00000464342,9606.ENSP00000464813,9606.ENSP00000479106	CPD,OAS3,EIF2AK2,CMPK2,HTRA2,GGH,COMMD4,PML,AKAP8,PSMB6,HK2,RAVER1,RPS23,ATP5L,HIST1H1E,STAT2,PARP10,UBA7,RPS7,RPL27A,WARS,STAT1,TGM2,PPOX,ADAR,CETN2,PSMB9,PSMB8,UQCRQ,TRMT61A,CNP,TYMP,RPL14,HLA-A,RPL31,OAS1,RPS18,GSPT1,HMGA1,SNRPE,TAPBP,CNBP,RPL32,GPHN,PARP14,TFAM,CDK11B,RNF213,RPL36,RPL27,HIST1H4A
GO:0044238	primary metabolic process	51	8808	0.12	4.17E-02	9606.ENSP00000225719,9606.ENSP00000226299,9606.ENSP00000228928,9606.ENSP00000233057,9606.ENSP00000249066,9606.ENSP00000256722,9606.ENSP00000258080,9606.ENSP00000260118,9606.ENSP00000267935,9606.ENSP00000268058,9606.ENSP00000269701,9606.ENSP00000270586,9606.ENSP00000290573,9606.ENSP00000293677,9606.ENSP00000296674,9606.ENSP00000300688,9606.ENSP00000307705,9606.ENSP00000315768,9606.ENSP00000325618,9606.ENSP00000333266,9606.ENSP00000339095,9606.ENSP00000346015,9606.ENSP00000347495,9606.ENSP00000354394,9606.ENSP00000355330,9606.ENSP00000357459,9606ENSP00000359300,9606.ENSP00000363993,9606.ENSP00000364016,9606.ENSP00000367939,9606.ENSP00000374399,9606.ENSP00000377470,9606.ENSP00000379038,9606.ENSP00000379506,9606.ENSP00000379873,9606.ENSP00000386717,9606.ENSP00000388001,9606.ENSP00000393241,9606.ENSP00000398131,9606.ENSP00000399888,9606.ENSP00000400591,9606.ENSP00000410769,9606.ENSP00000416429,9606.ENSP00000417901,9606.ENSP00000418194,9606.ENSP00000420588,9606.ENSP00000464036,9606.ENSP00000464087,9606.ENSP00000464342,9606.ENSP00000464813,9606.ENSP00000479106	CPD,LAP3,OAS3,EIF2AK2,APOL2,CMPK2,HTRA2,GGH,COMMD4,PML,AKAP8,PSMB6,HK2,RAVER1,RPS23,ATP5L,HIST1H1E,STAT2,PARP10,UBA7,RPS7,RPL27A,WARS,STAT1,TGM2,ADAR,CETN2,PSMB9,PSMB8,UQCRQ,TRMT61A,CNP,TYMP,RPL14,HLA-A,RPL31,OAS1,RPS18,GSPT1,HMGA1,SNRPE,CNBP,RPL32,GPHN,PARP14,TFAM,CDK11B,RNF213,RPL36,RPL27,HIST1H4A

GO:0071840	cellular component organization or biogenesis	35	5342	0.18	4.21E-02	9606.ENSP00000258080,9606.ENSP00000261517,9606.ENSP00000268058,9606.ENSP00000269701,9606.ENSP00000290573,9606.ENSP00000296674,9606.ENSP00000300688,9606.ENSP00000307705,9606.ENSP00000333657,9606.ENSP00000339095,9606.ENSP00000355330,9606.ENSP00000357459,9606.ENSP00000359300,9606.ENSP00000374566,9606.ENSP00000377390,9606.ENSP00000377470,9606.ENSP00000378563,9606.ENSP00000379038,9606.ENSP00000379506,9606.ENSP00000379873,9606.ENSP00000381601,9606.ENSP00000388001,9606.ENSP00000393241,9606.ENSP00000398131,9606.ENSP00000399888,9606.ENSP00000400591,9606.ENSP00000404833,9606.ENSP00000417901,9606.ENSP00000420588,9606.ENSP00000464087,9606.ENSP00000464813,9606.ENSP00000471242,9606.ENSP00000478061,9606.ENSP00000479106,9606.ENSP00000484121	HTRA2,VPS13C,PML,AKAP8,HK2,RPS23,ATP5L,HIST1H1E,MX2,RPS7,TGM2,ADAR,CETN2,KIF19,PPP6R3,CNP,PFDN6,TYMP,RPL14,HLA-A,MX1,OAS1,RPS18,GSPT1,HMGA1,SNRPE,TAPBP,GPHN,TFAM,RNF213,RPL27,FARP1,MARCKS,HIST1H4A,ARFIP2
GO:0010821	regulation of mitochondrion organization	4	148	0.79	4.43E-02	9606.ENSP00000258080,9606.ENSP00000261517,9606.ENSP00000290573,9606.ENSP00000315768	HTRA2,VPS13C,HK2,STAT2
GO:0009141	nucleoside triphosphate metabolic process	5	246	0.67	4.75E-02	9606.ENSP00000256722,9606.ENSP00000290573,9606.ENSP00000300688,9606.ENSP00000367939,9606.ENSP00000417901	CMPK2,HK2,ATP5L,UQCRQ,GPHN
GO:0008152	metabolic process	54	9569	0.11	4.81E-02	9606.ENSP00000225719,9606.ENSP00000226299,9606.ENSP00000228928,9606.ENSP00000233057,9606.ENSP00000249066,9606.ENSP00000256722,9606.ENSP00000258080,9606.ENSP00000260118,9606.ENSP00000267935,9606.ENSP00000268058,9606.ENSP00000269701,9606.ENSP00000270586,9606.ENSP00000290573,9606.ENSP00000293677,9606.ENSP00000296674,9606.ENSP00000300688,9606.ENSP00000307705,9606.ENSP00000315768,9606.ENSP00000325618,9606.ENSP00000333266,9606.ENSP00000339095,9606.ENSP00000346015,9606.ENSP00000347495,9606.ENSP00000354394,9606.ENSP00000355330,9606.ENSP00000356978,9606.ENSP00000357459,9606.ENSP00000359300,9606.ENSP00000363993,9606.ENSP00000364016,9606.ENSP00000367939,9606.ENSP00000374399,9606.ENSP00000377470,9606.ENSP00000379038,9606.ENSP00000379506,9606.ENSP00000379873,9606.ENSP00000386717,9606.ENSP00000388001,9606.ENSP00000392985,9606.ENSP00000393241,9606.ENSP00000398131,9606.ENSP00000399888,9606.ENSP00000400591,9606.ENSP00000404833,9606.ENSP00000410769,9606.ENSP00000416429,9606.ENSP00000417901,9606.ENSP00000418194,9606.ENSP00000420588,9606.ENSP00000464036,9606.ENSP00000464087,9606.ENSP00000464342,9606.ENSP00000464813,9606.ENSP00000479106	CPD,LAP3,OAS3,EIF2AK2,APOL2,CMPK2,HTRA2,GGH,COMMD4,PML,AKAP8,PSMB6,HK2,RAVER1,RPS23,ATP5L,HIST1H1E,STAT2,PARP10,UBA7,RPS7,RPL27A,WARS,STAT1,TGM2,PPOX,ADAR,CETN2,PSMB9,PSMB8,UQCRQ,TRMT61A,CNP,TYMP,RPL14,HLA-A,RPL31,OAS1,HTATIP2,RPS18,GSPT1,HMGA1,SNRPE,TAPBP,CNBP,RPL32,GPHN,PARP14,TFAM,CDK11B,RNF213,RPL36,RPL27,HIST1H4A

T = 72 h (mock) IFNα5 / Untreated

#Term ID	Term Description	Observed Gene Count	Background Gene Count	Strength	False Discovery Rate	Matching Proteins in Your Input (IDs)	Matching Proteins in Your Input (Labels)
GO:0045087	innate immune response	27	676	1	2.09E-16	9606.ENSP00000225698,9606.ENSP00000233057,9606.ENSP00000253110,9606.ENSP00000257570,9606.ENSP00000268058,9606.ENSP00000296161,9606.ENSP00000323889,9606.ENSP00000342278,9606.ENSP00000343023,9606.ENSP00000354394,9606.ENSP00000357459,9606.ENSP00000359490,9606.ENSP00000360860,9606.ENSP00000360883,9606.ENSP00000368699,9606.ENSP00000369213,9606.ENSP00000377344,9606.ENSP00000378217,9606.ENSP00000378786,9606.ENSP00000379873,9606.ENSP00000381601,9606.ENSP00000382707,9606.ENSP00000395590,9606.ENSP00000399168,9606.ENSP00000416561,9606.ENSP00000433138,9606.ENSP00000452780	C1QBP,EIF2AK2,C19orf66,OASL,PML,DTX3L,TRIM25,OAS2,SP100,STAT1,ADAR,GBP4,IFIT5,IFIT3,ISG15,DDX58,DDX60,PRKCD,HLA-DRA,HLA-A,MX1,IFITM3,IFI35,HLA-B,CFB,CASP1,B2M
GO:0006952	defense response	33	1234	0.82	2.68E-16	9606.ENSP00000225698,9606.ENSP00000233057,9606.ENSP00000249066,9606.ENSP00000253110,9606.ENSP00000257570,9606.ENSP00000259206,9606.ENSP00000262776,9606.ENSP00000268058,9606.ENSP00000296161,9606.ENSP00000323889,9606.ENSP00000342278,9606.ENSP00000343023,9606.ENSP00000354394,9606.ENSP00000357459,9606.ENSP00000359490,9606.ENSP00000359787,9606.ENSP00000360860,9606.ENSP00000360883,9606.ENSP00000368699,9606.ENSP00000369213,9606.ENSP00000377344,9606.ENSP00000378217,9606.ENSP00000378786,9606.ENSP00000379873,9606.ENSP00000381601,9606.ENSP00000382707,9606.ENSP00000395590,9606.ENSP00000399168,9606.ENSP00000416066,9606.ENSP00000416561,9606.ENSP00000428085,9606.ENSP00000433138,9606.ENSP00000452780	C1QBP,EIF2AK2,APOL2,C19orf66,OASL,IL1RN,LGALS3BP,PML,DTX3L,TRIM25,OAS2,SP100,STAT1,ADAR,GBP4,IFI44L,IFIT5,IFIT3,ISG15,DDX58,DDX60,PRKCD,HLA-DRA,HLA-A,MX1,IFITM3,IFI35,HLA-B,SERPINA1,CFB,RPL30,CASP1,B2M
GO:0098542	defense response to other organism	29	859	0.92	2.68E-16	9606.ENSP00000225698,9606.ENSP00000233057,9606.ENSP00000253110,9606.ENSP00000257570,9606.ENSP00000268058,9606.ENSP00000296161,9606.ENSP00000323889,9606.ENSP00000342278,9606.ENSP00000343023,9606.ENSP00000354394,9606.ENSP00000357459,9606.ENSP00000359490,9606.ENSP00000359787,9606.ENSP00000360860,9606.ENSP00000360883,9606.ENSP00000368699,9606.ENSP00000369213,9606.ENSP00000377344,9606.ENSP00000378217,9606.ENSP00000378786,9606.ENSP00000379873,9606.ENSP00000381601,9606.ENSP00000382707,9606.ENSP00000395590,9606.ENSP00000399168,9606.ENSP00000416561,9606.ENSP00000428085,9606.ENSP00000433138,9606.ENSP00000452780	C1QBP,EIF2AK2,C19orf66,OASL,PML,DTX3L,TRIM25,OAS2,SP100,STAT1,ADAR,GBP4,IFI44L,IFIT5,IFIT3,ISG15,DDX58,DDX60,PRKCD,HLA-DRA,HLA-A,MX1,IFITM3,IFI35,HLA-B,CFB,RPL30,CASP1,B2M
GO:0043901	negative regulation of multi-organism process	18	212	1.32	6.95E-16	9606.ENSP00000225698,9606.ENSP00000233057,9606.ENSP00000253110,9606.ENSP00000257570,9606.ENSP00000257604,9606.ENSP00000268058,9606.ENSP00000323889,9606.ENSP00000325618,9606.ENSP00000343023,9606.ENSP00000354394,9606.ENSP00000357459,9606.ENSP00000360860,9606.ENSP00000368699,9606.ENSP00000374455,9606.ENSP00000379873,9606.ENSP00000381601,9606.ENSP00000382707,9606.ENSP00000399168	C1QBP,EIF2AK2,C19orf66,OASL,TRAFD1,PML,TRIM25,PARP10,SP100,STAT1,ADAR,IFIT5,ISG15,SQSTM1,HLA-A,MX1,IFITM3,HLA-B

GO:0051607	defense response to virus	17	181	1.37	1.15E-15	9606.ENSP00000233057,9606.ENSP00000253110,9606.ENSP00000257570,9606.ENSP00000268058,9606.ENSP00000296161,9606.ENSP00000323889,9606.ENSP00000342278,9606.ENSP00000354394,9606.ENSP00000357459,9606.ENSP00000359787,9606.ENSP00000360860,9606.ENSP00000360883,9606.ENSP00000368699,9606.ENSP00000369213,9606.ENSP00000377344,9606.ENSP00000381601,9606.ENSP00000382707	EIF2AK2,C19orf66,OASL,PML,DTX3L,TRIM25,OAS2,STAT1,ADAR,IFI44L,IFIT5,IFIT3,ISG15,DDX58,DDX60,MX1,IFITM3
GO:0034340	response to type I interferon	13	70	1.66	3.22E-15	9606.ENSP00000253110,9606.ENSP00000257570,9606.ENSP00000342278,9606.ENSP00000343023,9606.ENSP00000354394,9606.ENSP00000357459,9606.ENSP00000360883,9606.ENSP00000368699,9606.ENSP00000379873,9606.ENSP00000381601,9606.ENSP00000382707,9606.ENSP00000395590,9606.ENSP00000399168	C19orf66,OASL,OAS2,SP100,STAT1,ADAR,IFIT3,ISG15,HLA-A,MX1,IFITM3,IFI35,HLA-B
GO:0051707	response to other organism	31	1173	0.82	3.22E-15	9606.ENSP00000225698,9606.ENSP00000233057,9606.ENSP00000253110,9606.ENSP00000257570,9606.ENSP00000268058,9606.ENSP00000296161,9606.ENSP00000323889,9606.ENSP00000342278,9606.ENSP00000343023,9606.ENSP00000354394,9606.ENSP00000357459,9606.ENSP00000359490,9606.ENSP00000359783,9606.ENSP00000359787,9606.ENSP00000360860,9606.ENSP00000360883,9606.ENSP00000368699,9606.ENSP00000369213,9606.ENSP00000377344,9606.ENSP00000377470,9606.ENSP00000378217,9606.ENSP00000378786,9606.ENSP00000379873,9606.ENSP00000381601,9606.ENSP00000382707,9606.ENSP00000395590,9606.ENSP00000399168,9606.ENSP00000416561,9606.ENSP00000428085,9606.ENSP00000433138,9606.ENSP00000452780	C1QBP,EIF2AK2,C19orf66,OASL,PML,DTX3L,TRIM25,OAS2,SP100,STAT1,ADAR,GBP4,IFI44,IFI44L,IFIT5,IFIT3,ISG15,DDX58,DDX60,CNP,PRKCD,HLA-DRA,HLA-A,MX1,IFITM3,IFI35,HLA-B,CFB,RPL30,CASP1,B2M
GO:0009615	response to virus	18	270	1.22	1.54E-14	9606.ENSP00000233057,9606.ENSP00000253110,9606.ENSP00000257570,9606.ENSP00000268058,9606.ENSP00000296161,9606.ENSP00000323889,9606.ENSP00000342278,9606.ENSP00000354394,9606.ENSP00000357459,9606.ENSP00000359783,9606.ENSP00000359787,9606.ENSP00000360860,9606.ENSP00000360883,9606.ENSP00000368699,9606.ENSP00000369213,9606.ENSP00000377344,9606.ENSP00000381601,9606.ENSP00000382707	EIF2AK2,C19orf66,OASL,PML,DTX3L,TRIM25,OAS2,STAT1,ADAR,IFI44,IFI44L,IFIT5,IFIT3,ISG15,DDX58,DDX60,MX1,IFITM3
GO:0060337	type I interferon signaling pathway	12	65	1.66	4.41E-14	9606.ENSP00000257570,9606.ENSP00000342278,9606.ENSP00000343023,9606.ENSP00000354394,9606.ENSP00000357459,9606.ENSP00000360883,9606.ENSP00000368699,9606.ENSP00000379873,9606.ENSP00000381601,9606.ENSP00000382707,9606.ENSP00000395590,9606.ENSP00000399168	OASL,OAS2,SP100,STAT1,ADAR,IFIT3,ISG15,HLA-A,MX1,IFITM3,IFI35,HLA-B
GO:0048525	negative regulation of viral process	13	93	1.54	5.06E-14	9606.ENSP00000233057,9606.ENSP00000253110,9606.ENSP00000257570,9606.ENSP00000268058,9606.ENSP00000323889,9606.ENSP00000325618,9606.ENSP00000343023,9606.ENSP00000354394,9606.ENSP00000357459,9606.ENSP00000360860,9606.ENSP00000368699,9606.ENSP00000381601,9606.ENSP00000382707	EIF2AK2,C19orf66,OASL,PML,TRIM25,PARP10,SP100,STAT1,ADAR,IFIT5,ISG15,MX1,IFITM3
GO:0034341	response to interferon-gamma	15	176	1.32	1.83E-13	9606.ENSP00000253110,9606.ENSP00000257570,9606.ENSP00000268058,9606.ENSP00000323889,9606.ENSP00000342278,9606.ENSP00000343023,9606.ENSP00000354394,9606.ENSP00000359490,9606.ENSP00000378217,9606.ENSP00000378786,9606.ENSP00000379873,9606.ENSP00000382707,9606.ENSP00000399168,9606.ENSP00000433138,9606.ENSP00000452780	C19orf66,OASL,PML,TRIM25,OAS2,SP100,STAT1,GBP4,PRKCD,HLA-DRA,HLA-A,IFITM3,HLA-B,CASP1,B2M

GO:0043900	regulation of multi-organism process	23	653	0.94	1.83E-13	9606.ENSP00000225698,9606.ENSP00000233057,9606.ENSP00000253110,9606.ENSP00000257570,9606.ENSP00000257604,9606.ENSP00000268058,9606.ENSP00000296161,9606.ENSP00000296577,9606.ENSP00000323889,9606.ENSP00000325618,9606.ENSP00000343023,9606.ENSP00000354394,9606.ENSP00000357459,9606.ENSP00000360860,9606.ENSP00000368699,9606.ENSP00000369213,9606.ENSP00000374455,9606.ENSP00000377344,9606.ENSP00000378217,9606.ENSP00000379873,9606.ENSP00000381601,9606.ENSP00000382707,9606.ENSP00000399168	C1QBP,EIF2AK2,C19orf66,OASL,TRAFD1,PML,DTX3L,ABCE1,TRIM25,PARP10,SP100,STAT1,ADAR,IFIT5,ISG15,DDX58,SQSTM1,DDX60,PRKCD,HLA-A,MX1,IFITM3,HLA-B
GO:0060333	interferon-gamma-mediated signaling pathway	11	69	1.6	2.46E-12	9606.ENSP00000257570,9606.ENSP00000268058,9606.ENSP00000323889,9606.ENSP00000342278,9606.ENSP00000343023,9606.ENSP00000354394,9606.ENSP00000378217,9606.ENSP00000378786,9606.ENSP00000379873,9606.ENSP00000399168,9606.ENSP00000452780	OASL,PML,TRIM25,OAS2,SP100,STAT1,PRKCD,HLA-DRA,HLA-A,HLA-B,B2M
GO:0006955	immune response	31	1560	0.69	2.65E-12	9606.ENSP00000225698,9606.ENSP00000233057,9606.ENSP00000253110,9606.ENSP00000257570,9606.ENSP00000259206,9606.ENSP00000268058,9606.ENSP00000296161,9606.ENSP00000323889,9606.ENSP00000342278,9606.ENSP00000343023,9606.ENSP00000354394,9606.ENSP00000357459,9606.ENSP00000359490,9606.ENSP00000360860,9606.ENSP00000360883,9606.ENSP00000368699,9606.ENSP00000369213,9606.ENSP00000377344,9606.ENSP00000378217,9606.ENSP00000378786,9606.ENSP00000379873,9606.ENSP00000381601,9606.ENSP00000382707,9606.ENSP00000395590,9606.ENSP00000399168,9606.ENSP00000404833,9606.ENSP00000416066,9606.ENSP00000416561,9606.ENSP00000428085,9606.ENSP00000433138,9606.ENSP00000452780	C1QBP,EIF2AK2,C19orf66,OASL,IL1RN,PML,DTX3L,TRIM25,OAS2,SP100,STAT1,ADAR,GBP4,IFIT5,IFIT3,ISG15,DDX58,DDX60,PRKCD,HLA-DRA,HLA-A,MX1,IFITM3,IFI35,HLA-B,TAPBP,SERPINA1,CFB,RPL30,CASP1,B2M
GO:0002376	immune system process	37	2370	0.59	4.21E-12	9606.ENSP00000225698,9606.ENSP00000233057,9606.ENSP00000253110,9606.ENSP00000257570,9606.ENSP00000259206,9606.ENSP00000268058,9606.ENSP00000296161,9606.ENSP00000317144,9606.ENSP00000323889,9606.ENSP00000333019,9606.ENSP00000342278,9606.ENSP00000343023,9606.ENSP00000354394,9606.ENSP00000357459,9606.ENSP00000359490,9606.ENSP00000359787,9606.ENSP00000360860,9606.ENSP00000360883,9606.ENSP00000363993,9606.ENSP00000368699,9606.ENSP00000369213,9606.ENSP00000372155,9606.ENSP00000374455,9606.ENSP00000377344,9606.ENSP00000378217,9606.ENSP00000378786,9606.ENSP00000379873,9606.ENSP00000381601,9606.ENSP00000382707,9606.ENSP00000395590,9606.ENSP00000399168,9606.ENSP00000404833,9606.ENSP00000416066,9606.ENSP00000416561,9606.ENSP00000428085,9606.ENSP00000433138,9606.ENSP00000452780	C1QBP,EIF2AK2,C19orf66,OASL,IL1RN,PML,DTX3L,PIBF1,TRIM25,ADSSL1,OAS2,SP100,STAT1,ADAR,GBP4,IFI44L,IFIT5,IFIT3,PSMB9,ISG15,DDX58,PSME1,SQSTM1,DDX60,PRKCD,HLA-DRA,HLA-A,MX1,IFITM3,IFI35,HLA-B,TAPBP,SERPINA1,CFB,RPL30,CASP1,B2M

GO:0051704	multi-organism process	38	2514	0.57	4.21E-12	9606.ENSP00000225698,9606.ENSP00000233057,9606.ENSP00000244061,9606.ENSP00000249066,9606.ENSP00000253110,9606.ENSP00000257570,9606.ENSP00000268058,9606.ENSP00000296161,9606.ENSP00000296577,9606.ENSP00000323889,9606.ENSP00000334373,9606.ENSP00000342278,9606.ENSP00000343023,9606.ENSP00000354394,9606.ENSP00000355599,9606.ENSP00000357459,9606.ENSP00000359490,9606.ENSP00000359783,9606.ENSP00000359787,9606.ENSP00000360860,9606.ENSP00000360883,9606.ENSP00000363993,9606.ENSP00000368699,9606.ENSP00000369213,9606.ENSP00000371958,9606.ENSP00000377344,9606.ENSP00000377470,9606.ENSP00000378217,9606.ENSP00000378786,9606.ENSP00000379873,9606.ENSP00000381601,9606.ENSP00000382707,9606.ENSP00000395590,9606.ENSP00000399168,9606.ENSP00000416561,9606.ENSP00000428085,9606.ENSP00000433138,9606.ENSP00000452780	C1QBP,EIF2AK2,RNF114,APOL2,C19orf66,OASL,PML,DTX3L,ABCE1,TRIM25,KPNA4,OAS2,SP100,STAT1,TSNAX,ADAR,GBP4,IFI44,IFI44L,IFIT5,IFIT3,PSMB9,ISG15,DDX58,CD9,DDX60,CNP,PRKCD,HLA-DRA,HLA-A,MX1,IFITM3,IFI35,HLA-B,CFB,RPL30,CASP1,B2M
GO:1903901	negative regulation of viral life cycle	11	77	1.55	5.86E-12	9606.ENSP00000233057,9606.ENSP00000253110,9606.ENSP00000257570,9606.ENSP00000268058,9606.ENSP00000323889,9606.ENSP00000325618,9606.ENSP00000357459,9606.ENSP00000360860,9606.ENSP00000368699,9606.ENSP00000381601,9606.ENSP00000382707	EIF2AK2,C19orf66,OASL,PML,TRIM25,PARP10,ADAR,IFIT5,ISG15,MX1,IFITM3
GO:0071346	cellular response to interferon-gamma	13	155	1.32	1.37E-11	9606.ENSP00000257570,9606.ENSP00000268058,9606.ENSP00000323889,9606.ENSP00000342278,9606.ENSP00000343023,9606.ENSP00000354394,9606.ENSP00000359490,9606.ENSP00000378217,9606.ENSP00000378786,9606.ENSP00000379873,9606.ENSP00000399168,9606.ENSP00000433138,9606.ENSP00000452780	OASL,PML,TRIM25,OAS2,SP100,STAT1,GBP4,PRKCD,HLA-DRA,HLA-A,HLA-B,CASP1,B2M
GO:0019221	cytokine-mediated signaling pathway	21	655	0.9	1.55E-11	9606.ENSP00000231487,9606.ENSP00000257570,9606.ENSP00000259206,9606.ENSP00000268058,9606.ENSP00000323889,9606.ENSP00000342278,9606.ENSP00000343023,9606.ENSP00000354394,9606.ENSP00000357459,9606.ENSP00000360883,9606.ENSP00000368699,9606.ENSP00000374455,9606.ENSP00000378217,9606.ENSP00000378786,9606.ENSP00000379873,9606.ENSP00000381601,9606.ENSP00000382707,9606.ENSP00000395590,9606.ENSP00000399168,9606.ENSP00000433138,9606.ENSP00000452780	SKP1,OASL,IL1RN,PML,TRIM25,OAS2,SP100,STAT1,ADAR,IFIT3,ISG15,SQSTM1,PRKCD,HLA-DRA,HLA-A,MX1,IFITM3,IFI35,HLA-B,CASP1,B2M
GO:0043903	regulation of symbiosis, encompassing mutualism through parasitism	14	203	1.23	1.65E-11	9606.ENSP00000233057,9606.ENSP00000253110,9606.ENSP00000257570,9606.ENSP00000268058,9606.ENSP00000323889,9606.ENSP00000325618,9606.ENSP00000343023,9606.ENSP00000354394,9606.ENSP00000357459,9606.ENSP00000360860,9606.ENSP00000368699,9606.ENSP00000374455,9606.ENSP00000381601,9606.ENSP00000382707	EIF2AK2,C19orf66,OASL,PML,TRIM25,PARP10,SP100,STAT1,ADAR,IFIT5,ISG15,SQSTM1,MX1,IFITM3

GO:0002252	immune effector process	24	927	0.81	1.67E-11	9606.ENSP00000225698,9606.ENSP00000233057,9606.ENSP00000253110,9606.ENSP00000257570,9606.ENSP00000268058,9606.ENSP00000296161,9606.ENSP00000323889,9606.ENSP00000342278,9606.ENSP00000354394,9606.ENSP00000357459,9606.ENSP00000359787,9606.ENSP00000360860,9606.ENSP00000360883,9606.ENSP00000368699,9606.ENSP00000369213,9606.ENSP00000377344,9606.ENSP00000378217,9606.ENSP00000379873,9606.ENSP00000381601,9606.ENSP00000382707,9606.ENSP00000399168,9606.ENSP00000416066,9606.ENSP00000416561,9606.ENSP00000452780	C1QBP,EIF2AK2,C19orf66,OASL,PML,DTX3L,TRIM25,OAS2,STAT1,ADAR,IFI44L,IFIT5,IFIT3,ISG15,DDX58,DDX60,PRKCD,HLA-A,MX1,IFITM3,HLA-B,SERPINA1,CFB,B2M
GO:0034097	response to cytokine	25	1035	0.78	2.00E-11	9606.ENSP00000231487,9606.ENSP00000233057,9606.ENSP00000253110,9606.ENSP00000257570,9606.ENSP00000257604,9606.ENSP00000259206,9606.ENSP00000268058,9606.ENSP00000323889,9606.ENSP00000342278,9606.ENSP00000343023,9606.ENSP00000354394,9606.ENSP00000357459,9606.ENSP00000359490,9606.ENSP00000360883,9606.ENSP00000368699,9606.ENSP00000374455,9606.ENSP00000378217,9606.ENSP00000378786,9606.ENSP00000379873,9606.ENSP00000381601,9606.ENSP00000382707,9606.ENSP00000395590,9606.ENSP00000399168,9606.ENSP00000433138,9606.ENSP00000452780	SKP1,EIF2AK2,C19orf66,OASL,TRAFD1,IL1RN,PML,TRIM25,OAS2,SP100,STAT1,ADAR,GBP4,IFIT3,ISG15,SQSTM1,PRKCD,HLA-DRA,HLA-A,MX1,IFITM3,IFI35,HLA-B,CASP1,B2M
GO:0009605	response to external stimulus	34	2152	0.59	4.02E-11	9606.ENSP00000225698,9606.ENSP00000233057,9606.ENSP00000253110,9606.ENSP00000257570,9606.ENSP00000264108,9606.ENSP00000268058,9606.ENSP00000296161,9606.ENSP00000323889,9606.ENSP00000333019,9606.ENSP00000342278,9606.ENSP00000343023,9606.ENSP00000354394,9606.ENSP00000357459,9606.ENSP00000359490,9606.ENSP00000359783,9606.ENSP00000359787,9606.ENSP00000360860,9606.ENSP00000360883,9606.ENSP00000368699,9606.ENSP00000369213,9606.ENSP00000377344,9606.ENSP00000377470,9606.ENSP00000378217,9606.ENSP00000378786,9606.ENSP00000379038,9606.ENSP00000379873,9606.ENSP00000381601,9606.ENSP00000382707,9606.ENSP00000395590,9606.ENSP00000399168,9606.ENSP00000416561,9606.ENSP00000428085,9606.ENSP00000433138,9606.ENSP00000452780	C1QBP,EIF2AK2,C19orf66,OASL,HAT1,PML,DTX3L,TRIM25,ADSSL1,OAS2,SP100,STAT1,ADAR,GBP4,IFI44,IFI44L,IFIT5,IFIT3,ISG15,DDX58,DDX60,CNP,PRKCD,HLA-DRA,TYMP,HLA-A,MX1,IFITM3,IFI35,HLA-B,CFB,RPL30,CASP1,B2M

GO:0006950	response to stress	41	3267	0.49	7.28E-11	9606.ENSP00000225698,9606.ENSP00000231487,9606.ENSP00000233057,9606.ENSP00000249066,9606.ENSP00000253110,9606.ENSP00000257570,9606.ENSP00000259206,9606.ENSP00000262776,9606.ENSP00000268058,9606.ENSP00000287156,9606.ENSP00000296161,9606.ENSP00000323889,9606.ENSP00000333019,9606.ENSP00000333266,9606.ENSP00000334373,9606.ENSP00000342278,9606.ENSP00000343023,9606.ENSP00000354394,9606.ENSP00000357459,9606.ENSP00000359490,9606.ENSP00000359787,9606.ENSP00000360860,9606.ENSP00000360883,9606.ENSP00000368699,9606.ENSP00000369213,9606.ENSP00000371958,9606.ENSP00000374455,9606.ENSP00000377344,9606.ENSP00000378217,9606.ENSP00000378786,9606.ENSP00000379873,9606.ENSP00000381601,9606.ENSP00000382707,9606.ENSP00000395590,9606.ENSP00000399168,9606.ENSP00000416066,9606.ENSP00000416561,9606.ENSP00000428085,9606.ENSP00000433138,9606.ENSP00000452780,9606.ENSP00000478026	C1QBP,SKP1,EIF2AK2,APOL2,C19orf66,OASL,IL1RN,LGALS3BP,PML,UBE2L6,DTX3L,TRIM25,ADSSL1,UBA7,KPNA4,OAS2,SP100,STAT1,ADAR,GBP4,IFI44L,IFIT5,IFIT3,ISG15,DDX58,CD9,SQSTM1,DDX60,PRKCD,HLA-DRA,HLA-A,MX1,IFITM3,IFI35,HLA-B,SERPINA1,CFB,RPL30,CASP1,B2M,ANXA8
GO:0045071	negative regulation of viral genome replication	9	52	1.63	1.89E-10	9606.ENSP00000233057,9606.ENSP00000253110,9606.ENSP00000257570,9606.ENSP00000325618,9606.ENSP00000357459,9606.ENSP00000360860,9606.ENSP00000368699,9606.ENSP00000381601,9606.ENSP00000382707	EIF2AK2,C19orf66,OASL,PARP10,ADAR,IFIT5,ISG15,MX1,IFITM3
GO:0071345	cellular response to cytokine stimulus	22	953	0.76	1.48E-09	9606.ENSP00000231487,9606.ENSP00000257570,9606.ENSP00000259206,9606.ENSP00000268058,9606.ENSP00000323889,9606.ENSP00000342278,9606.ENSP00000343023,9606.ENSP00000354394,9606.ENSP00000357459,9606.ENSP00000359490,9606.ENSP00000360883,9606.ENSP00000368699,9606.ENSP00000374455,9606.ENSP00000378217,9606.ENSP00000378786,9606.ENSP00000379873,9606.ENSP00000381601,9606.ENSP00000382707,9606.ENSP00000395590,9606.ENSP00000399168,9606.ENSP00000433138,9606.ENSP00000452780	SKP1,OASL,IL1RN,PML,TRIM25,OAS2,SP100,STAT1,ADAR,GBP4,IFIT3,ISG15,SQSTM1,PRKCD,HLA-DRA,HLA-A,MX1,IFITM3,IFI35,HLA-B,CASP1,B2M
GO:0001817	regulation of cytokine production	16	615	0.81	2.31E-07	9606.ENSP00000225698,9606.ENSP00000233057,9606.ENSP00000268058,9606.ENSP00000287156,9606.ENSP00000317144,9606.ENSP00000323889,9606.ENSP00000333266,9606.ENSP00000354394,9606.ENSP00000368699,9606.ENSP00000369213,9606.ENSP00000377344,9606.ENSP00000379873,9606.ENSP00000399168,9606.ENSP00000433138,9606.ENSP00000452780,9606.ENSP00000463188	C1QBP,EIF2AK2,PML,UBE2L6,PIBF1,TRIM25,UBA7,STAT1,ISG15,DDX58,DDX60,HLA-A,HLA-B,CASP1,B2M,TRIM16
GO:0016032	viral process	15	571	0.81	6.53E-07	9606.ENSP00000225698,9606.ENSP00000233057,9606.ENSP00000268058,9606.ENSP00000296577,9606.ENSP00000323889,9606.ENSP00000334373,9606.ENSP00000343023,9606.ENSP00000354394,9606.ENSP00000363993,9606.ENSP00000368699,9606.ENSP00000369213,9606.ENSP00000378786,9606.ENSP00000379873,9606.ENSP00000399168,9606.ENSP00000452780	C1QBP,EIF2AK2,PML,ABCE1,TRIM25,KPNA4,SP100,STAT1,PSMB9,ISG15,DDX58,HLA-DRA,HLA-A,HLA-B,B2M

GO:0010033	response to organic substance	32	2815	0.45	9.00E-07	9606.ENSP00000231487,9606.ENSP00000233057,9606.ENSP00000253110,9606.ENSP00000257570,9606.ENSP00000257604,9606.ENSP00000259206,9606.ENSP00000268058,9606.ENSP00000323889,9606.ENSP00000325677,9606.ENSP00000342278,9606.ENSP00000343023,9606.ENSP00000354394,9606.ENSP00000357459,9606.ENSP00000358327,9606.ENSP00000359490,9606.ENSP00000360883,9606.ENSP00000368678,9606.ENSP00000368699,9606.ENSP00000369213,9606.ENSP00000371958,9606.ENSP00000374455,9606.ENSP00000377470,9606.ENSP00000378217,9606.ENSP00000378786,9606.ENSP00000379873,9606.ENSP00000381601,9606.ENSP00000382707,9606.ENSP00000395590,9606.ENSP00000399168,9606.ENSP00000433138,9606.ENSP00000452780,9606.ENSP00000463188	SKP1,EIF2AK2,C19orf66,OASL,TRAFD1,IL1RN,PML,TRIM25,RNF40,OAS2,SP100,STAT1,ADAR,CASP7,GBP4,IFIT3,AGRN,ISG15,DDX58,CD9,SQSTM1,CNP,PRKCD,HLA-DRA,HLA-A,MX1,IFITM3,IFI35,HLA-B,CASP1,B2M,TRIM16
GO:0044419	interspecies interaction between organisms	16	724	0.74	1.99E-06	9606.ENSP00000225698,9606.ENSP00000233057,9606.ENSP00000268058,9606.ENSP00000296577,9606.ENSP00000323889,9606.ENSP00000334373,9606.ENSP00000343023,9606.ENSP00000354394,9606.ENSP00000363993,9606.ENSP00000368699,9606.ENSP00000369213,9606.ENSP00000378786,9606.ENSP00000379873,9606.ENSP00000399168,9606.ENSP00000428085,9606.ENSP00000452780	C1QBP,EIF2AK2,PML,ABCE1,TRIM25,KPNA4,SP100,STAT1,PSMB9,ISG15,DDX58,HLA-DRA,HLA-A,HLA-B,RPL30,B2M
GO:0019538	protein metabolic process	39	4194	0.36	2.47E-06	9606.ENSP00000161006,9606.ENSP00000225698,9606.ENSP00000226299,9606.ENSP00000231487,9606.ENSP00000233057,9606.ENSP00000244061,9606.ENSP00000249066,9606.ENSP00000264108,9606.ENSP00000268058,9606.ENSP00000287156,9606.ENSP00000296161,9606.ENSP00000296543,9606.ENSP00000296577,9606.ENSP00000306548,9606.ENSP00000317144,9606.ENSP00000323889,9606.ENSP00000325618,9606.ENSP00000325677,9606.ENSP00000333266,9606.ENSP00000347495,9606.ENSP00000355330,9606.ENSP00000358327,9606.ENSP00000363993,9606.ENSP00000366819,9606.ENSP00000368699,9606.ENSP00000369213,9606.ENSP00000372155,9606.ENSP00000374455,9606.ENSP00000378217,9606.ENSP00000379873,9606.ENSP00000381167,9606.ENSP00000416066,9606.ENSP00000416561,9606.ENSP00000428085,9606.ENSP00000433138,9606.ENSP00000452780,9606.ENSP00000463188,9606.ENSP00000464087,9606.ENSP00000464342	PRSS22,C1QBP,LAP3,SKP1,EIF2AK2,RNF114,APOL2,HAT1,PML,UBE2L6,DTX3L,NAA15,ABCE1,MRPL13,PIBF1,TRIM25,PARP10,RNF40,UBA7,WARS,TGM2,CASP7,PSMB9,UCHL3,ISG15,DDX58,PSME1,SQSTM1,PRKCD,HLA-A,DYNC2H1,SERPINA1,CFB,RPL30,CASP1,B2M,TRIM16,RNF213,RPL36
GO:0006508	proteolysis	20	1203	0.61	2.92E-06	9606.ENSP00000161006,9606.ENSP00000226299,9606.ENSP00000231487,9606.ENSP00000268058,9606.ENSP00000287156,9606.ENSP00000296161,9606.ENSP00000323889,9606.ENSP00000325677,9606.ENSP00000333266,9606.ENSP00000358327,9606.ENSP00000363993,9606.ENSP00000366819,9606.ENSP00000368699,9606.ENSP00000369213,9606.ENSP00000372155,9606.ENSP00000374455,9606.ENSP00000381167,9606.ENSP00000416561,9606.ENSP00000433138,9606.ENSP00000464087	PRSS22,LAP3,SKP1,PML,UBE2L6,DTX3L,TRIM25,RNF40,UBA7,CASP7,PSMB9,UCHL3,ISG15,DDX58,PSME1,SQSTM1,DYNC2H1,CFB,CASP1,RNF213

GO:1901564	organonitrogen compound metabolic process	44	5281	0.31	4.11E-06	9606.ENSP00000161006,9606.ENSP00000225698,9606.ENSP00000226299,9606.ENSP00000229379,9606.ENSP00000231487,9606.ENSP00000233057,9606.ENSP00000244061,9606.ENSP00000249066,9606.ENSP00000264108,9606.ENSP00000268058,9606.ENSP00000287156,9606.ENSP00000296161,9606.ENSP00000296543,9606.ENSP00000296577,9606.ENSP00000306548,9606.ENSP00000317144,9606.ENSP00000323889,9606.ENSP00000325618,9606.ENSP00000325677,9606.ENSP00000333019,9606.ENSP00000333266,9606.ENSP00000347495,9606.ENSP00000355330,9606.ENSP00000358327,9606.ENSP00000363993,9606.ENSP00000366819,9606.ENSP00000368678,9606.ENSP00000368699,9606.ENSP00000369213,9606.ENSP00000372155,9606.ENSP00000374455,9606.ENSP00000378217,9606.ENSP00000379038,9606.ENSP00000379873,9606.ENSP00000381167,9606.ENSP00000404833,9606.ENSP00000416066,9606.ENSP00000416561,9606.ENSP00000428085,9606.ENSP00000433138,9606.ENSP00000452780,9606.ENSP00000463188,9606.ENSP00000464087,9606.ENSP00000464342	PRSS22,C1QBP,LAP3,COX6A1,SKP1,EIF2AK2,RNF114,APOL2,HAT1,PML,UBE2L6,DTX3L,NAA15,ABCE1,MRPL13,PIBF1,TRIM25,PARP10,RNF40,ADSSL1,UBA7,WARS,TGM2,CASP7,PSMB9,UCHL3,AGRN,ISG15,DDX58,PSME1,SQSTM1,PRKCD,TYMP,HLA-A,DYNC2H1,TAPBP,SERPINA1,CFB,RPL30,CASP1,B2M,TRIM16,RNF213,RPL36
GO:0031347	regulation of defense response	15	676	0.74	4.75E-06	9606.ENSP00000225698,9606.ENSP00000257604,9606.ENSP00000268058,9606.ENSP00000296161,9606.ENSP00000296577,9606.ENSP00000354394,9606.ENSP00000355330,9606.ENSP00000357459,9606.ENSP00000369213,9606.ENSP00000377344,9606.ENSP00000378217,9606.ENSP00000379873,9606.ENSP00000399168,9606.ENSP00000416561,9606.ENSP00000433138	C1QBP,TRAFD1,PML,DTX3L,ABCE1,STAT1,TGM2,ADAR,DDX58,DDX60,PRKCD,HLA-A,HLA-B,CFB,CASP1
GO:0002831	regulation of response to biotic stimulus	12	426	0.84	8.73E-06	9606.ENSP00000225698,9606.ENSP00000257604,9606.ENSP00000268058,9606.ENSP00000296161,9606.ENSP00000296577,9606.ENSP00000354394,9606.ENSP00000357459,9606.ENSP00000369213,9606.ENSP00000377344,9606.ENSP00000378217,9606.ENSP00000379873,9606.ENSP00000399168	C1QBP,TRAFD1,PML,DTX3L,ABCE1,STAT1,ADAR,DDX58,DDX60,PRKCD,HLA-A,HLA-B
GO:0019885	antigen processing and presentation of endogenous peptide antigen via MHC class I	4	9	2.04	8.73E-06	9606.ENSP00000379873,9606.ENSP00000399168,9606.ENSP00000404833,9606.ENSP00000452780	HLA-A,HLA-B,TAPBP,B2M
GO:0071310	cellular response to organic substance	26	2219	0.46	1.61E-05	9606.ENSP00000231487,9606.ENSP00000233057,9606.ENSP00000257570,9606.ENSP00000259206,9606.ENSP00000268058,9606.ENSP00000323889,9606.ENSP00000342278,9606.ENSP00000343023,9606.ENSP00000354394,9606.ENSP00000357459,9606.ENSP00000358327,9606.ENSP00000359490,9606.ENSP00000360883,9606.ENSP00000368678,9606.ENSP00000368699,9606.ENSP00000369213,9606.ENSP00000374455,9606.ENSP00000378217,9606.ENSP00000378786,9606.ENSP00000379873,9606.ENSP00000381601,9606.ENSP00000382707,9606.ENSP00000395590,9606.ENSP00000399168,9606.ENSP00000433138,9606.ENSP00000452780	SKP1,EIF2AK2,OASL,IL1RN,PML,TRIM25,OAS2,SP100,STAT1,ADAR,CASP7,GBP4,IFIT3,AGRN,ISG15,DDX58,SQSTM1,PRKCD,HLA-DRA,HLA-A,MX1,IFITM3,IFI35,HLA-B,CASP1,B2M

GO:0050896	response to stimulus	54	7824	0.23	1.62E-05	9606.ENSP00000225698,9606.ENSP00000231487,9606.ENSP00000233057,9606.ENSP00000249066,9606.ENSP00000253110,9606.ENSP00000257570,9606.ENSP00000257604,9606.ENSP00000259206,9606.ENSP00000262776,9606.ENSP00000264020,9606.ENSP00000264108,9606.ENSP00000268058,9606.ENSP00000287156,9606.ENSP00000296161,9606.ENSP00000323889,9606.ENSP00000325677,9606.ENSP00000333019,9606.ENSP00000333266,9606.ENSP00000334373,9606.ENSP00000342278,9606.ENSP00000343023,9606.ENSP00000354394,9606.ENSP00000355330,9606.ENSP00000357459,9606.ENSP00000358327,9606.ENSP00000359490,9606.ENSP00000359783,9606.ENSP00000359787,9606.ENSP00000360860,9606.ENSP00000360883,9606.ENSP00000368678,9606.ENSP00000368699,9606.ENSP00000369213,9606.ENSP00000371958,9606.ENSP00000374455,9606.ENSP00000377344,9606.ENSP00000377470,9606.ENSP00000378217,9606.ENSP00000378786,9606.ENSP00000379038,9606.ENSP00000379873,9606.ENSP00000381601,9606.ENSP00000382707,9606.ENSP00000395590,9606.ENSP00000399168,9606.ENSP00000404833,9606.ENSP00000416066,9606.ENSP00000416561,9606.ENSP00000428085,9606.ENSP00000430344,9606.ENSP00000433138,9606.ENSP00000452780,9606.ENSP00000463188,9606.ENSP00000478026	C1QBP,SKP1,EIF2AK2,APOL2,C19orf66,OASL,TRAFD1,IL1RN,LGALS3BP,IFT46,HAT1,PML,UBE2L6,DTX3L,TRIM25,RNF40,ADSSL1,UBA7,KPNA4,OAS2,SP100,STAT1,TGM2,ADAR,CASP7,GBP4,IFI44,IFI44L,IFIT5,IFIT3,AGRN,ISG15,DDX58,CD9,SQSTM1,DDX60,CNP,PRKCD,HLA-DRA,TYMP,HLA-A,MX1,IFITM3,IFI35,HLA-B,TAPBP,SERPINA1,CFB,RPL30,PLCD1,CASP1,B2M,TRIM16,ANXA8
GO:0048519	negative regulation of biological process	41	4953	0.31	1.90E-05	9606.ENSP00000225698,9606.ENSP00000231487,9606.ENSP00000233057,9606.ENSP00000253110,9606.ENSP00000257570,9606.ENSP00000257604,9606.ENSP00000259206,9606.ENSP00000264108,9606.ENSP00000268058,9606.ENSP00000287156,9606.ENSP00000296161,9606.ENSP00000296543,9606.ENSP00000296577,9606.ENSP00000317144,9606.ENSP00000319281,9606.ENSP00000323889,9606.ENSP00000325618,9606.ENSP00000325677,9606.ENSP00000333266,9606.ENSP00000343023,9606.ENSP00000347495,9606.ENSP00000354394,9606.ENSP00000357459,9606.ENSP00000360860,9606.ENSP00000360883,9606.ENSP00000368699,9606.ENSP00000369213,9606.ENSP00000371958,9606.ENSP00000374455,9606.ENSP00000378217,9606.ENSP00000379873,9606.ENSP00000381601,9606.ENSP00000382707,9606.ENSP00000396704,9606.ENSP00000399168,9606.ENSP00000416066,9606.ENSP00000428085,9606.ENSP00000452780,9606.ENSP00000464087,9606.ENSP00000464342,9606.ENSP00000478026	C1QBP,SKP1,EIF2AK2,C19orf66,OASL,TRAFD1,IL1RN,HAT1,PML,UBE2L6,DTX3L,NAA15,ABCE1,PIBF1,BASP1,TRIM25,PARP10,RNF40,UBA7,SP100,WARS,STAT1,ADAR,IFIT5,IFIT3,ISG15,DDX58,CD9,SQSTM1,PRKCD,HLA-A,MX1,IFITM3,TOP2B,HLA-B,SERPINA1,RPL30,B2M,RNF213,RPL36,ANXA8
GO:0050688	regulation of defense response to virus	6	71	1.32	2.93E-05	9606.ENSP00000225698,9606.ENSP00000268058,9606.ENSP00000296161,9606.ENSP00000354394,9606.ENSP00000369213,9606.ENSP00000377344	C1QBP,PML,DTX3L,STAT1,DDX58,DDX60

GO:0042221	response to chemical	36	4153	0.33	5.44E-05	9606.ENSP00000231487,9606.ENSP00000233057,9606.ENSP00000253110,9606.ENSP00000257570,9606.ENSP00000257604,9606.ENSP00000259206,9606.ENSP00000264108,9606.ENSP00000268058,9606.ENSP00000323889,9606.ENSP00000325677,9606.ENSP00000333019,9606.ENSP00000334373,9606.ENSP00000342278,9606.ENSP00000343023,9606.ENSP00000354394,9606.ENSP00000357459,9606.ENSP00000358327,9606.ENSP00000359490,9606.ENSP00000360883,9606.ENSP00000368678,9606.ENSP00000368699,9606.ENSP00000369213,9606.ENSP00000371958,9606.ENSP00000374455,9606.ENSP00000377470,9606.ENSP00000378217,9606.ENSP00000378786,9606.ENSP00000379038,9606.ENSP00000379873,9606.ENSP00000381601,9606.ENSP00000382707,9606.ENSP00000395590,9606.ENSP00000399168,9606.ENSP00000433138,9606.ENSP00000452780,9606.ENSP00000463188	SKP1,EIF2AK2,C19orf66,OASL,TRAFD1,IL1RN,HAT1,PML,TRIM25,RNF40,ADSSL1,KPNA4,OAS2,SP100,STAT1,ADAR,CASP7,GBP4,IFIT3,AGRN,ISG15,DDX58,CD9,SQSTM1,CNP,PRKCD,HLA-DRA,TYMP,HLA-A,MX1,IFITM3,IFI35,HLA-B,CASP1,B2M,TRIM16
GO:0032101	regulation of response to external stimulus	16	955	0.62	5.49E-05	9606.ENSP00000225698,9606.ENSP00000257604,9606.ENSP00000268058,9606.ENSP00000296161,9606.ENSP00000296577,9606.ENSP00000354394,9606.ENSP00000355330,9606.ENSP00000357459,9606.ENSP00000369213,9606.ENSP00000371958,9606.ENSP00000377344,9606.ENSP00000378217,9606.ENSP00000379873,9606.ENSP00000399168,9606.ENSP00000416561,9606.ENSP00000433138	C1QBP,TRAFD1,PML,DTX3L,ABCE1,STAT1,TGM2,ADAR,DDX58,CD9,DDX60,PRKCD,HLA-A,HLA-B,CFB,CASP1
GO:0032480	negative regulation of type I interferon production	5	43	1.46	5.78E-05	9606.ENSP00000287156,9606.ENSP00000323889,9606.ENSP00000333266,9606.ENSP00000368699,9606.ENSP00000369213	UBE2L6,TRIM25,UBA7,ISG15,DDX58
GO:0044265	cellular macromolecule catabolic process	15	842	0.64	5.78E-05	9606.ENSP00000231487,9606.ENSP00000268058,9606.ENSP00000287156,9606.ENSP00000296161,9606.ENSP00000323889,9606.ENSP00000325677,9606.ENSP00000333266,9606.ENSP00000342278,9606.ENSP00000363993,9606.ENSP00000366819,9606.ENSP00000368699,9606.ENSP00000374455,9606.ENSP00000428085,9606.ENSP00000464087,9606.ENSP00000464342	SKP1,PML,UBE2L6,DTX3L,TRIM25,RNF40,UBA7,OAS2,PSMB9,UCHL3,ISG15,SQSTM1,RPL30,RNF213,RPL36
GO:0009057	macromolecule catabolic process	16	970	0.61	6.33E-05	9606.ENSP00000231487,9606.ENSP00000268058,9606.ENSP00000287156,9606.ENSP00000296161,9606.ENSP00000323889,9606.ENSP00000325677,9606.ENSP00000333266,9606.ENSP00000342278,9606.ENSP00000363993,9606.ENSP00000366819,9606.ENSP00000368678,9606.ENSP00000368699,9606.ENSP00000374455,9606.ENSP00000428085,9606.ENSP00000464087,9606.ENSP00000464342	SKP1,PML,UBE2L6,DTX3L,TRIM25,RNF40,UBA7,OAS2,PSMB9,UCHL3,AGRN,ISG15,SQSTM1,RPL30,RNF213,RPL36
GO:0051603	proteolysis involved in cellular protein catabolic process	12	532	0.75	6.75E-05	9606.ENSP00000231487,9606.ENSP00000268058,9606.ENSP00000287156,9606.ENSP00000296161,9606.ENSP00000323889,9606.ENSP00000325677,9606.ENSP00000333266,9606.ENSP00000363993,9606.ENSP00000366819,9606.ENSP00000368699,9606.ENSP00000374455,9606.ENSP00000464087	SKP1,PML,UBE2L6,DTX3L,TRIM25,RNF40,UBA7,PSMB9,UCHL3,ISG15,SQSTM1,RNF213
GO:0045088	regulation of innate immune response	10	361	0.84	8.99E-05	9606.ENSP00000225698,9606.ENSP00000257604,9606.ENSP00000296577,9606.ENSP00000354394,9606.ENSP00000357459,9606.ENSP00000369213,9606.ENSP00000377344,9606.ENSP00000378217,9606.ENSP00000379873,9606.ENSP00000399168	C1QBP,TRAFD1,ABCE1,STAT1,ADAR,DDX58,DDX60,PRKCD,HLA-A,HLA-B
GO:0002697	regulation of immune effector process	10	362	0.84	9.05E-05	9606.ENSP00000225698,9606.ENSP00000268058,9606.ENSP00000296161,9606.ENSP00000354394,9606.ENSP00000369213,9606.ENSP00000377344,9606.ENSP00000379873,9606.ENSP00000399168,9606.ENSP00000416561,9606.ENSP00000452780	C1QBP,PML,DTX3L,STAT1,DDX58,DDX60,HLA-A,HLA-B,CFB,B2M

GO:0019941	modification-dependent protein catabolic process	11	467	0.77	1.20E-04	9606.ENSP00000231487,9606.ENSP00000268058,9606.ENSP00000287156,9606.ENSP00000296161,9606.ENSP00000323889,9606.ENSP00000325677,9606.ENSP00000333266,9606.ENSP00000366819,9606.ENSP00000368699,9606.ENSP00000374455,9606.ENSP00000464087	SKP1,PML,UBE2L6,DTX3L,TRIM25,RNF40,UBA7,UCHL3,ISG15,SQSTM1,RNF213
GO:0035455	response to interferon-alpha	4	22	1.65	1.20E-04	9606.ENSP00000233057,9606.ENSP00000357459,9606.ENSP00000360883,9606.ENSP00000382707	EIF2AK2,ADAR,IFIT3,IFITM3
GO:0070887	cellular response to chemical stimulus	27	2672	0.4	1.20E-04	9606.ENSP00000231487,9606.ENSP00000233057,9606.ENSP00000257570,9606.ENSP00000259206,9606.ENSP00000268058,9606.ENSP00000323889,9606.ENSP00000333019,9606.ENSP00000342278,9606.ENSP00000343023,9606.ENSP00000354394,9606.ENSP00000357459,9606.ENSP00000358327,9606.ENSP00000359490,9606.ENSP00000360883,9606.ENSP00000368678,9606.ENSP00000368699,9606.ENSP00000369213,9606.ENSP00000374455,9606.ENSP00000378217,9606.ENSP00000378786,9606.ENSP00000379873,9606.ENSP00000381601,9606.ENSP00000382707,9606.ENSP00000395590,9606.ENSP00000399168,9606.ENSP00000433138,9606.ENSP00000452780	SKP1,EIF2AK2,OASL,IL1RN,PML,TRIM25,ADSSL1,OAS2,SP100,STAT1,ADAR,CASP7,GBP4,IFIT3,AGRN,ISG15,DDX58,SQSTM1,PRKCD,HLA-DRA,HLA-A,MX1,IFITM3,IFI35,HLA-B,CASP1,B2M
GO:0002479	antigen processing and presentation of exogenous peptide antigen via MHC class I, TAP-dependent	4	25	1.6	1.80E-04	9606.ENSP00000379873,9606.ENSP00000399168,9606.ENSP00000404833,9606.ENSP00000452780	HLA-A,HLA-B,TAPBP,B2M
GO:0032020	ISG15-protein conjugation	3	6	2.09	1.80E-04	9606.ENSP00000287156,9606.ENSP00000333266,9606.ENSP00000368699	UBE2L6,UBA7,ISG15
GO:0002230	positive regulation of defense response to virus by host	4	26	1.58	2.10E-04	9606.ENSP00000268058,9606.ENSP00000296161,9606.ENSP00000354394,9606.ENSP00000369213	PML,DTX3L,STAT1,DDX58
GO:0001818	negative regulation of cytokine production	8	245	0.91	2.60E-04	9606.ENSP00000225698,9606.ENSP00000268058,9606.ENSP00000287156,9606.ENSP00000317144,9606.ENSP00000323889,9606.ENSP00000333266,9606.ENSP00000368699,9606.ENSP00000369213	C1QBP,PML,UBE2L6,PIBF1,TRIM25,UBA7,ISG15,DDX58
GO:0032479	regulation of type I interferon production	6	112	1.12	2.60E-04	9606.ENSP00000287156,9606.ENSP00000323889,9606.ENSP00000333266,9606.ENSP00000354394,9606.ENSP00000368699,9606.ENSP00000369213	UBE2L6,TRIM25,UBA7,STAT1,ISG15,DDX58
GO:0002682	regulation of immune system process	18	1391	0.51	2.90E-04	9606.ENSP00000225698,9606.ENSP00000233057,9606.ENSP00000257604,9606.ENSP00000268058,9606.ENSP00000296161,9606.ENSP00000296577,9606.ENSP00000317144,9606.ENSP00000354394,9606.ENSP00000357459,9606.ENSP00000368699,9606.ENSP00000369213,9606.ENSP00000377344,9606.ENSP00000378217,9606.ENSP00000378786,9606.ENSP00000379873,9606.ENSP00000399168,9606.ENSP00000416561,9606.ENSP00000452780	C1QBP,EIF2AK2,TRAFD1,PML,DTX3L,ABCE1,PIBF1,STAT1,ADAR,ISG15,DDX58,DDX60,PRKCD,HLA-DRA,HLA-A,HLA-B,CFB,B2M
GO:0002480	antigen processing and presentation of exogenous peptide antigen via MHC class I, TAP-independent	3	8	1.97	3.10E-04	9606.ENSP00000379873,9606.ENSP00000399168,9606.ENSP00000452780	HLA-A,HLA-B,B2M

GO:0006807	nitrogen compound metabolic process	53	8349	0.2	3.30E-04	9606.ENSP00000161006,9606.ENSP00000225698,9606.ENSP00000226299,9606.ENSP00000229379,9606.ENSP00000231487,9606.ENSP00000233057,9606.ENSP00000244061,9606.ENSP00000249066,9606.ENSP00000264108,9606.ENSP00000268058,9606.ENSP00000287156,9606.ENSP00000296161,9606.ENSP00000296543,9606.ENSP00000296577,9606.ENSP00000306548,9606.ENSP00000317144,9606.ENSP00000323889,9606.ENSP00000325618,9606.ENSP00000325677,9606.ENSP00000333019,9606.ENSP00000333266,9606.ENSP00000342278,9606.ENSP00000343023,9606.ENSP00000347495,9606.ENSP00000354394,9606.ENSP00000355330,9606.ENSP00000357459,9606.ENSP00000358327,9606.ENSP00000363993,9606.ENSP00000365950,9606.ENSP00000366819,9606.ENSP00000368678,9606.ENSP00000368699,9606.ENSP00000369213,9606.ENSP00000369965,9606.ENSP00000372155,9606.ENSP00000374455,9606.ENSP00000377470,9606.ENSP00000378217,9606.ENSP00000379038,9606.ENSP00000379873,9606.ENSP00000381167,9606.ENSP00000396704,9606.ENSP00000400591,9606.ENSP00000404833,9606.ENSP00000416066,9606.ENSP00000416561,9606.ENSP00000428085,9606.ENSP00000433138,9606.ENSP00000452780,9606.ENSP00000463188,9606.ENSP00000464087,9606.ENSP00000464342	PRSS22,C1QBP,LAP3,COX6A1,SKP1,EIF2AK2,RNF114,APOL2,HAT1,PML,UBE2L6,DTX3L,NAA15,ABCE1,MRPL13,PIBF1,TRIM25,PARP10,RNF40,ADSSL1,UBA7,OAS2,SP100,WARS,STAT1,TGM2,ADAR,CASP7,PSMB9,RBM3,UCHL3,AGRN,ISG15,DDX58,BTF3,PSME1,SQSTM1,CNP,PRKCD,TYMP,HLA-A,DYNC2H1,TOP2B,SNRPE,TAPBP,SERPINA1,CFB,RPL30,CASP1,B2M,TRIM16,RNF213,RPL36
GO:0071704	organic substance metabolic process	56	9135	0.18	3.60E-04	9606.ENSP00000161006,9606.ENSP00000225698,9606.ENSP00000226299,9606.ENSP00000229379,9606.ENSP00000231487,9606.ENSP00000233057,9606.ENSP00000244061,9606.ENSP00000249066,9606.ENSP00000259206,9606.ENSP00000264108,9606.ENSP00000268058,9606.ENSP00000287156,9606.ENSP00000296161,9606.ENSP00000296543,9606.ENSP00000296577,9606.ENSP00000306548,9606.ENSP00000317144,9606.ENSP00000323889,9606.ENSP00000325618,9606.ENSP00000325677,9606.ENSP00000333019,9606.ENSP00000333266,9606.ENSP00000342278,9606.ENSP00000343023,9606.ENSP00000347495,9606.ENSP00000354394,9606.ENSP00000355330,9606.ENSP00000357459,9606.ENSP00000358327,9606.ENSP00000363993,9606.ENSP00000365950,9606.ENSP00000366819,9606.ENSP00000368678,9606.ENSP00000368699,9606.ENSP00000369213,9606.ENSP00000369965,9606.ENSP00000371958,9606.ENSP00000372155,9606.ENSP00000374455,9606.ENSP00000377470,9606.ENSP00000378217,9606.ENSP00000379038,9606.ENSP00000379873,9606.ENSP00000381167,9606.ENSP00000396704,9606.ENSP00000400591,9606.ENSP00000404833,9606.ENSP00000416066,9606.ENSP00000416561,9606.ENSP00000428085,9606.ENSP00000430344,9606.ENSP00000433138,9606.ENSP00000452780,9606.ENSP00000463188,9606.ENSP00000464087,9606.ENSP00000464342	PRSS22,C1QBP,LAP3,COX6A1,SKP1,EIF2AK2,RNF114,APOL2,IL1RN,HAT1,PML,UBE2L6,DTX3L,NAA15,ABCE1,MRPL13,PIBF1,TRIM25,PARP10,RNF40,ADSSL1,UBA7,OAS2,SP100,WARS,STAT1,TGM2,ADAR,CASP7,PSMB9,RBM3,UCHL3,AGRN,ISG15,DDX58,BTF3,CD9,PSME1,SQSTM1,CNP,PRKCD,TYMP,HLA-A,DYNC2H1,TOP2B,SNRPE,TAPBP,SERPINA1,CFB,RPL30,PLCD1,CASP1,B2M,TRIM16,RNF213,RPL36

GO:0044267	cellular protein metabolic process	31	3603	0.33	3.80E-04	9606.ENSP00000231487,9606.ENSP00000233057,9606.ENSP00000244061,9606.ENSP00000264108,9606.ENSP00000268058,9606.ENSP00000287156,9606.ENSP00000296161,9606.ENSP00000296543,9606.ENSP00000296577,9606.ENSP00000306548,9606.ENSP00000317144,9606.ENSP00000323889,9606.ENSP00000325618,9606.ENSP00000325677,9606.ENSP00000333266,9606.ENSP00000347495,9606.ENSP00000355330,9606.ENSP00000363993,9606.ENSP00000366819,9606.ENSP00000368699,9606.ENSP00000369213,9606.ENSP00000372155,9606.ENSP00000374455,9606.ENSP00000378217,9606.ENSP00000379873,9606.ENSP00000416066,9606.ENSP00000428085,9606.ENSP00000452780,9606.ENSP00000463188,9606.ENSP00000464087,9606.ENSP00000464342	SKP1,EIF2AK2,RNF114,HAT1,PML,UBE2L6,DTX3L,NAA15,ABCE1,MRPL13,PIBF1,TRIM25,PARP10,RNF40,UBA7,WARS,TGM2,PSMB9,UCHL3,ISG15,DDX58,PSME1,SQSTM1,PRKCD,HLA-A,SERPINA1,RPL30,B2M,TRIM16,RNF213,RPL36
GO:0007166	cell surface receptor signaling pathway	23	2198	0.41	3.90E-04	9606.ENSP00000231487,9606.ENSP00000257570,9606.ENSP00000259206,9606.ENSP00000264020,9606.ENSP00000268058,9606.ENSP00000323889,9606.ENSP00000342278,9606.ENSP00000343023,9606.ENSP00000354394,9606.ENSP00000357459,9606.ENSP00000360883,9606.ENSP00000368699,9606.ENSP00000371958,9606.ENSP00000374455,9606.ENSP00000378217,9606.ENSP00000378786,9606.ENSP00000379873,9606.ENSP00000381601,9606.ENSP00000382707,9606.ENSP00000395590,9606.ENSP00000399168,9606.ENSP00000433138,9606.ENSP00000452780	SKP1,OASL,IL1RN,IFT46,PML,TRIM25,OAS2,SP100,STAT1,ADAR,IFIT3,ISG15,CD9,SQSTM1,PRKCD,HLA-DRA,HLA-A,MX1,IFITM3,IFI35,HLA-B,CASP1,B2M
GO:0043170	macromolecule metabolic process	49	7453	0.21	4.20E-04	9606.ENSP00000161006,9606.ENSP00000225698,9606.ENSP00000226299,9606.ENSP00000231487,9606.ENSP00000233057,9606.ENSP00000244061,9606.ENSP00000249066,9606.ENSP00000264108,9606.ENSP00000268058,9606.ENSP00000287156,9606.ENSP00000296161,9606.ENSP00000296543,9606.ENSP00000296577,9606.ENSP00000306548,9606.ENSP00000317144,9606.ENSP00000323889,9606.ENSP00000325618,9606.ENSP00000325677,9606.ENSP00000333266,9606.ENSP00000342278,9606.ENSP00000343023,9606.ENSP00000347495,9606.ENSP00000354394,9606.ENSP00000355330,9606.ENSP00000357459,9606.ENSP00000358327,9606.ENSP00000363993,9606.ENSP00000365950,9606.ENSP00000366819,9606.ENSP00000368678,9606.ENSP00000368699,9606.ENSP00000369213,9606.ENSP00000369965,9606.ENSP00000371958,9606.ENSP00000372155,9606.ENSP00000374455,9606.ENSP00000378217,9606.ENSP00000379873,9606.ENSP00000381167,9606.ENSP00000396704,9606.ENSP00000400591,9606.ENSP00000416066,9606.ENSP00000416561,9606.ENSP00000428085,9606.ENSP00000433138,9606.ENSP00000452780,9606.ENSP00000463188,9606.ENSP00000464087,9606.ENSP00000464342	PRSS22,C1QBP,LAP3,SKP1,EIF2AK2,RNF114,APOL2,HAT1,PML,UBE2L6,DTX3L,NAA15,ABCE1,MRPL13,PIBF1,TRIM25,PARP10,RNF40,UBA7,OAS2,SP100,WARS,STAT1,TGM2,ADAR,CASP7,PSMB9,RBM3,UCHL3,AGRN,ISG15,DDX58,BTF3,CD9,PSME1,SQSTM1,PRKCD,HLA-A,DYNC2H1,TOP2B,SNRPE,SERPINA1,CFB,RPL30,CASP1,B2M,TRIM16,RNF213,RPL36
GO:0080134	regulation of response to stress	17	1299	0.51	4.20E-04	9606.ENSP00000225698,9606.ENSP00000233057,9606.ENSP00000257604,9606.ENSP00000268058,9606.ENSP00000296161,9606.ENSP00000296577,9606.ENSP00000354394,9606.ENSP00000355330,9606.ENSP00000357459,9606.ENSP00000369213,9606.ENSP00000371958,9606.ENSP00000377344,9606.ENSP00000378217,9606.ENSP00000379873,9606.ENSP00000399168,9606.ENSP00000416561,9606.ENSP00000433138	C1QBP,EIF2AK2,TRAFD1,PML,DTX3L,ABCE1,STAT1,TGM2,ADAR,DDX58,CD9,DDX60,PRKCD,HLA-A,HLA-B,CFB,CASP1

GO:0019884	antigen processing and presentation of exogenous antigen	6	130	1.06	5.00E-04	9606.ENSP00000372155,9606.ENSP00000378786,9606.ENSP00000379873,9606.ENSP00000399168,9606.ENSP00000404833,9606.ENSP00000452780	PSME1,HLA-DRA,HLA-A,HLA-B,TAPBP,B2M
GO:1901575	organic substance catabolic process	19	1609	0.47	5.00E-04	9606.ENSP00000231487,9606.ENSP00000268058,9606.ENSP00000287156,9606.ENSP00000296161,9606.ENSP00000323889,9606.ENSP00000325677,9606.ENSP00000333266,9606.ENSP00000342278,9606.ENSP00000363993,9606.ENSP00000366819,9606.ENSP00000368678,9606.ENSP00000368699,9606.ENSP00000374455,9606.ENSP00000377470,9606.ENSP00000379038,9606.ENSP00000428085,9606.ENSP00000430344,9606.ENSP00000464087,9606.ENSP00000464342	SKP1,PML,UBE2L6,DTX3L,TRIM25,RNF40,UBA7,OAS2,PSMB9,UCHL3,AGRN,ISG15,SQSTM1,CNP,TYMP,RPL30,PLCD1,RNF213,RPL36
GO:0044238	primary metabolic process	54	8808	0.18	6.80E-04	9606.ENSP00000161006,9606.ENSP00000225698,9606.ENSP00000226299,9606.ENSP00000229379,9606.ENSP00000231487,9606.ENSP00000233057,9606.ENSP00000244061,9606.ENSP00000249066,9606.ENSP00000259206,9606.ENSP00000264108,9606.ENSP00000268058,9606.ENSP00000287156,9606.ENSP00000296161,9606.ENSP00000296543,9606.ENSP00000296577,9606.ENSP00000306548,9606.ENSP00000317144,9606.ENSP00000323889,9606.ENSP00000325618,9606.ENSP00000325677,9606.ENSP00000333019,9606.ENSP00000333266,9606.ENSP00000342278,9606.ENSP00000343023,9606.ENSP00000347495,9606.ENSP00000354394,9606.ENSP00000355330,9606.ENSP00000357459,9606.ENSP00000358327,9606.ENSP00000363993,9606.ENSP00000365950,9606.ENSP00000366819,9606.ENSP00000368678,9606.ENSP00000368699,9606.ENSP00000369213,9606.ENSP00000369965,9606.ENSP00000372155,9606.ENSP00000374455,9606.ENSP00000377470,9606.ENSP00000378217,9606.ENSP00000379038,9606.ENSP00000379873,9606.ENSP00000381167,9606.ENSP00000396704,9606.ENSP00000400591,9606.ENSP00000416066,9606.ENSP00000416561,9606.ENSP00000428085,9606.ENSP00000430344,9606.ENSP00000433138,9606.ENSP00000452780,9606.ENSP00000463188,9606.ENSP00000464087,9606.ENSP00000464342	PRSS22,C1QBP,LAP3,COX6A1,SKP1,EIF2AK2,RNF114,APOL2,IL1RN,HAT1,PML,UBE2L6,DTX3L,NAA15,ABCE1,MRPL13,PIBF1,TRIM25,PARP10,RNF40,ADSSL1,UBA7,OAS2,SP100,WARS,STAT1,TGM2,ADAR,CASP7,PSMB9,RBM3,UCHL3,AGRN,ISG15,DDX58,BTF3,PSME1,SQSTM1,CNP,PRKCD,TYMP,HLA-A,DYNC2H1,TOP2B,SNRPE,SERPINA1,CFB,RPL30,PLCD1,CASP1,B2M,TRIM16,RNF213,RPL36
GO:1901565	organonitrogen compound catabolic process	14	958	0.56	7.80E-04	9606.ENSP00000231487,9606.ENSP00000268058,9606.ENSP00000287156,9606.ENSP00000296161,9606.ENSP00000323889,9606.ENSP00000325677,9606.ENSP00000333266,9606.ENSP00000363993,9606.ENSP00000366819,9606.ENSP00000368678,9606.ENSP00000368699,9606.ENSP00000374455,9606.ENSP00000379038,9606.ENSP00000464087	SKP1,PML,UBE2L6,DTX3L,TRIM25,RNF40,UBA7,PSMB9,UCHL3,AGRN,ISG15,SQSTM1,TYMP,RNF213
GO:0001819	positive regulation of cytokine production	9	390	0.76	8.20E-04	9606.ENSP00000233057,9606.ENSP00000317144,9606.ENSP00000354394,9606.ENSP00000369213,9606.ENSP00000377344,9606.ENSP00000379873,9606.ENSP00000433138,9606.ENSP00000452780,9606.ENSP00000463188	EIF2AK2,PIBF1,STAT1,DDX58,DDX60,HLA-A,CASP1,B2M,TRIM16
GO:0002832	negative regulation of response to biotic stimulus	5	85	1.16	8.20E-04	9606.ENSP00000225698,9606.ENSP00000257604,9606.ENSP00000357459,9606.ENSP00000379873,9606.ENSP00000399168	C1QBP,TRAFD1,ADAR,HLA-A,HLA-B

GO:0050776	regulation of immune response	13	873	0.57	1.20E-03	9606.ENSP00000225698,9606.ENSP00000257604,9606.ENSP00000296577,9606.ENSP00000354394,9606.ENSP00000357459,9606.ENSP00000369213,9606.ENSP00000377344,9606.ENSP00000378217,9606.ENSP00000378786,9606.ENSP00000379873,9606.ENSP00000399168,9606.ENSP00000416561,9606.ENSP00000452780	C1QBP,TRAFD1,ABCE1,STAT1,ADAR,DDX58,DDX60,PRKCD,HLA-DRA,HLA-A,HLA-B,CFB,B2M
GO:0045824	negative regulation of innate immune response	4	49	1.31	1.50E-03	9606.ENSP00000257604,9606.ENSP00000357459,9606.ENSP00000379873,9606.ENSP00000399168	TRAFD1,ADAR,HLA-A,HLA-B
GO:0060759	regulation of response to cytokine stimulus	6	162	0.96	1.50E-03	9606.ENSP00000259206,9606.ENSP00000296577,9606.ENSP00000354394,9606.ENSP00000357459,9606.ENSP00000369213,9606.ENSP00000433138	IL1RN,ABCE1,STAT1,ADAR,DDX58,CASP1
GO:0001916	positive regulation of T cell mediated cytotoxicity	3	17	1.64	1.60E-03	9606.ENSP00000379873,9606.ENSP00000399168,9606.ENSP00000452780	HLA-A,HLA-B,B2M
GO:0043122	regulation of I-kappaB kinase/NF-kappaB signaling	6	167	0.95	1.60E-03	9606.ENSP00000323889,9606.ENSP00000354394,9606.ENSP00000355330,9606.ENSP00000360860,9606.ENSP00000374455,9606.ENSP00000433138	TRIM25,STAT1,TGM2,IFIT5,SQSTM1,CASP1
GO:0051239	regulation of multicellular organismal process	25	2788	0.35	1.60E-03	9606.ENSP00000225698,9606.ENSP00000233057,9606.ENSP00000259206,9606.ENSP00000268058,9606.ENSP00000287156,9606.ENSP00000317144,9606.ENSP00000319281,9606.ENSP00000323889,9606.ENSP00000333266,9606.ENSP00000342278,9606.ENSP00000343023,9606.ENSP00000347495,9606.ENSP00000354394,9606.ENSP00000368678,9606.ENSP00000368699,9606.ENSP00000369213,9606.ENSP00000371958,9606.ENSP00000377344,9606.ENSP00000378217,9606.ENSP00000379038,9606.ENSP00000379873,9606.ENSP00000399168,9606.ENSP00000433138,9606.ENSP00000452780,9606.ENSP00000463188	C1QBP,EIF2AK2,IL1RN,PML,UBE2L6,PIBF1,BASP1,TRIM25,UBA7,OAS2,SP100,WARS,STAT1,AGRN,ISG15,DDX58,CD9,DDX60,PRKCD,TYMP,HLA-A,HLA-B,CASP1,B2M,TRIM16
GO:0009617	response to bacterium	10	555	0.65	2.00E-03	9606.ENSP00000342278,9606.ENSP00000359783,9606.ENSP00000368699,9606.ENSP00000377470,9606.ENSP00000378217,9606.ENSP00000379873,9606.ENSP00000399168,9606.ENSP00000428085,9606.ENSP00000433138,9606.ENSP00000452780	OAS2,IFI44,ISG15,CNP,PRKCD,HLA-A,HLA-B,RPL30,CASP1,B2M
GO:0031348	negative regulation of defense response	6	180	0.92	2.40E-03	9606.ENSP00000225698,9606.ENSP00000257604,9606.ENSP00000357459,9606.ENSP00000378217,9606.ENSP00000379873,9606.ENSP00000399168	C1QBP,TRAFD1,ADAR,PRKCD,HLA-A,HLA-B
GO:0070647	protein modification by small protein conjugation or removal	13	945	0.53	2.40E-03	9606.ENSP00000231487,9606.ENSP00000244061,9606.ENSP00000287156,9606.ENSP00000296161,9606.ENSP00000323889,9606.ENSP00000325677,9606.ENSP00000333266,9606.ENSP00000363993,9606.ENSP00000366819,9606.ENSP00000369213,9606.ENSP00000372155,9606.ENSP00000379873,9606.ENSP00000464087	SKP1,RNF114,UBE2L6,DTX3L,TRIM25,RNF40,UBA7,PSMB9,UCHL3,DDX58,PSME1,HLA-A,RNF213
GO:0006511	ubiquitin-dependent protein catabolic process	9	461	0.68	2.50E-03	9606.ENSP00000231487,9606.ENSP00000268058,9606.ENSP00000296161,9606.ENSP00000323889,9606.ENSP00000325677,9606.ENSP00000333266,9606.ENSP00000366819,9606.ENSP00000374455,9606.ENSP00000464087	SKP1,PML,DTX3L,TRIM25,RNF40,UBA7,UCHL3,SQSTM1,RNF213

GO:0050789	regulation of biological process	61	11116	0.13	2.80E-03	9606.ENSP00000225698,9606.ENSP00000231487,9606.ENSP00000233057,9606.ENSP00000244061,9606.ENSP00000253110,9606.ENSP00000257570,9606.ENSP00000257604,9606.ENSP00000259206,9606.ENSP00000262776,9606.ENSP00000264020,9606.ENSP00000264108,9606.ENSP00000268058,9606.ENSP00000287156,9606.ENSP00000296161,9606.ENSP00000296543,9606.ENSP00000296577,9606.ENSP00000317144,9606.ENSP00000319281,9606.ENSP00000323889,9606.ENSP00000325618,9606.ENSP00000325677,9606.ENSP00000333266,9606.ENSP00000342278,9606.ENSP00000343023,9606.ENSP00000347495,9606.ENSP00000354394,9606.ENSP00000355330,9606.ENSP00000357459,9606.ENSP00000360860,9606.ENSP00000360883,9606.ENSP00000363993,9606.ENSP00000365950,9606.ENSP00000368678,9606.ENSP00000368699,9606.ENSP00000369213,9606.ENSP00000369965,9606.ENSP00000371958,9606.ENSP00000372155,9606.ENSP00000374455,9606.ENSP00000377344,9606.ENSP00000378217,9606.ENSP00000378786,9606.ENSP00000379038,9606.ENSP00000379873,9606.ENSP00000381167,9606.ENSP00000381601,9606.ENSP00000382707,9606.ENSP00000395590,9606.ENSP00000396704,9606.ENSP00000399168,9606.ENSP00000404833,9606.ENSP00000416066,9606.ENSP00000416561,9606.ENSP00000428085,9606.ENSP00000430344,9606.ENSP00000433138,9606.ENSP00000452780,9606.ENSP00000463188,9606.ENSP00000464087,9606.ENSP00000464342,9606.ENSP00000478026	C1QBP,SKP1,EIF2AK2,RNF114,C19orf66,OASL,TRAFD1,IL1RN,LGALS3BP,IFT46,HAT1,PML,UBE2L6,DTX3L,NAA15,ABCE1,PIBF1,BASP1,TRIM25,PARP10,RNF40,UBA7,OAS2,SP100,WARS,STAT1,TGM2,ADAR,IFIT5,IFIT3,PSMB9,RBM3,AGRN,ISG15,DDX58,BTF3,CD9,PSME1,SQSTM1,DDX60,PRKCD,HLA-DRA,TYMP,HLA-A,DYNC2H1,MX1,IFITM3,IFI35,TOP2B,HLA-B,TAPBP,SERPINA1,CFB,RPL30,PLCD1,CASP1,B2M,TRIM16,RNF213,RPL36,ANXA8
GO:0006513	protein monoubiquitination	4	61	1.21	2.90E-03	9606.ENSP00000231487,9606.ENSP00000296161,9606.ENSP00000323889,9606.ENSP00000325677	SKP1,DTX3L,TRIM25,RNF40
GO:0051457	maintenance of protein location in nucleus	3	22	1.53	2.90E-03	9606.ENSP00000231487,9606.ENSP00000268058,9606.ENSP00000343023	SKP1,PML,SP100
GO:0002478	antigen processing and presentation of exogenous peptide antigen	5	123	1	3.40E-03	9606.ENSP00000378786,9606.ENSP00000379873,9606.ENSP00000399168,9606.ENSP00000404833,9606.ENSP00000452780	HLA-DRA,HLA-A,HLA-B,TAPBP,B2M
GO:0002486	antigen processing and presentation of endogenous peptide antigen via MHC class I via ER pathway, TAP-independent	2	3	2.22	3.40E-03	9606.ENSP00000379873,9606.ENSP00000399168	HLA-A,HLA-B

GO:0065007	biological regulation	63	11740	0.12	3.40E-03	9606.ENSP00000225698,9606.ENSP00000229379,9606.ENSP00000231487,9606.ENSP00000233057,9606.ENSP00000244061,9606.ENSP00000253110,9606.ENSP00000257570,9606.ENSP00000257604,9606.ENSP00000259206,9606.ENSP00000262776,9606.ENSP00000264020,9606.ENSP00000264108,9606.ENSP00000268058,9606.ENSP00000287156,9606.ENSP00000296161,9606.ENSP00000296543,9606.ENSP00000296577,9606.ENSP00000317144,9606.ENSP00000319281,9606.ENSP00000323889,9606.ENSP00000325618,9606.ENSP00000325677,9606.ENSP00000333266,9606.ENSP00000342278,9606.ENSP00000343023,9606.ENSP00000347495,9606.ENSP00000354394,9606.ENSP00000355330,9606.ENSP00000357459,9606.ENSP00000360860,9606.ENSP00000360883,9606.ENSP00000363993,9606.ENSP00000365950,9606.ENSP00000368678,9606.ENSP00000368699,9606.ENSP00000369213,9606.ENSP00000369965,9606.ENSP00000371958,9606.ENSP00000372155,9606.ENSP00000374455,9606.ENSP00000377344,9606.ENSP00000377470,9606.ENSP00000378217,9606.ENSP00000378786,9606.ENSP00000379038,9606.ENSP00000379873,9606.ENSP00000381167,9606.ENSP00000381601,9606.ENSP00000382707,9606.ENSP00000395590,9606.ENSP00000396704,9606.ENSP00000399168,9606.ENSP00000404833,9606.ENSP00000416066,9606.ENSP00000416561,9606.ENSP00000428085,9606.ENSP00000430344,9606.ENSP00000433138,9606.ENSP00000452780,9606.ENSP00000463188,9606.ENSP00000464087,9606.ENSP00000464342,9606.ENSP00000478026	C1QBP,COX6A1,SKP1,EIF2AK2,RNF114,C19orf66,OASL,TRAFD1,IL1RN,LGALS3BP,IFT46,HAT1,PML,UBE2L6,DTX3L,NAA15,ABCE1,PIBF1,BASP1,TRIM25,PARP10,RNF40,UBA7,OAS2,SP100,WARS,STAT1,TGM2,ADAR,IFIT5,IFIT3,PSMB9,RBM3,AGRN,ISG15,DDX58,BTF3,CD9,PSME1,SQSTM1,DDX60,CNP,PRKCD,HLA-DRA,TYMP,HLA-A,DYNC2H1,MX1,IFITM3,IFI35,TOP2B,HLA-B,TAPBP,SERPINA1,CFB,RPL30,PLCD1,CASP1,B2M,TRIM16,RNF213,RPL36,ANXA8
GO:0006464	cellular protein modification process	25	2999	0.31	4.50E-03	9606.ENSP00000231487,9606.ENSP00000233057,9606.ENSP00000244061,9606.ENSP00000264108,9606.ENSP00000268058,9606.ENSP00000287156,9606.ENSP00000296161,9606.ENSP00000296543,9606.ENSP00000317144,9606.ENSP00000323889,9606.ENSP00000325618,9606.ENSP00000325677,9606.ENSP00000333266,9606.ENSP00000355330,9606.ENSP00000363993,9606.ENSP00000366819,9606.ENSP00000368699,9606.ENSP00000369213,9606.ENSP00000372155,9606.ENSP00000374455,9606.ENSP00000378217,9606.ENSP00000379873,9606.ENSP00000416066,9606.ENSP00000463188,9606.ENSP00000464087	SKP1,EIF2AK2,RNF114,HAT1,PML,UBE2L6,DTX3L,NAA15,PIBF1,TRIM25,PARP10,RNF40,UBA7,TGM2,PSMB9,UCHL3,ISG15,DDX58,PSME1,SQSTM1,PRKCD,HLA-A,SERPINA1,TRIM16,RNF213
GO:0043412	macromolecule modification	26	3197	0.3	4.60E-03	9606.ENSP00000231487,9606.ENSP00000233057,9606.ENSP00000244061,9606.ENSP00000264108,9606.ENSP00000268058,9606.ENSP00000287156,9606.ENSP00000296161,9606.ENSP00000296543,9606.ENSP00000317144,9606.ENSP00000323889,9606.ENSP00000325618,9606.ENSP00000325677,9606.ENSP00000333266,9606.ENSP00000355330,9606.ENSP00000357459,9606.ENSP00000363993,9606.ENSP00000366819,9606.ENSP00000368699,9606.ENSP00000369213,9606.ENSP00000372155,9606.ENSP00000374455,9606.ENSP00000378217,9606.ENSP00000379873,9606.ENSP00000416066,9606.ENSP00000463188,9606.ENSP00000464087	SKP1,EIF2AK2,RNF114,HAT1,PML,UBE2L6,DTX3L,NAA15,PIBF1,TRIM25,PARP10,RNF40,UBA7,TGM2,ADAR,PSMB9,UCHL3,ISG15,DDX58,PSME1,SQSTM1,PRKCD,HLA-A,SERPINA1,TRIM16,RNF213

GO:0044248	cellular catabolic process	17	1646	0.41	5.40E-03	9606.ENSP00000231487,9606.ENSP00000268058,9606.ENSP00000287156,9606.ENSP00000296161,9606.ENSP00000323889,9606.ENSP00000325677,9606.ENSP00000333266,9606.ENSP00000342278,9606.ENSP00000363993,9606.ENSP00000366819,9606.ENSP00000368699,9606.ENSP00000374455,9606.ENSP00000377470,9606.ENSP00000379038,9606.ENSP00000428085,9606.ENSP00000464087,9606.ENSP00000464342	SKP1,PML,UBE2L6,DTX3L,TRIM25,RNF40,UBA7,OAS2,PSMB9,UCHL3,ISG15,SQSTM1,CNP,TYMP,RPL30,RNF213,RPL36
GO:0032102	negative regulation of response to external stimulus	7	310	0.75	5.60E-03	9606.ENSP00000225698,9606.ENSP00000257604,9606.ENSP00000357459,9606.ENSP00000371958,9606.ENSP00000378217,9606.ENSP00000379873,9606.ENSP00000399168	C1QBP,TRAFD1,ADAR,CD9,PRKCD,HLA-A,HLA-B
GO:0010390	histone monoubiquitination	3	30	1.39	6.00E-03	9606.ENSP00000231487,9606.ENSP00000296161,9606.ENSP00000325677	SKP1,DTX3L,RNF40
GO:0050690	regulation of defense response to virus by virus	3	30	1.39	6.00E-03	9606.ENSP00000233057,9606.ENSP00000379873,9606.ENSP00000452780	EIF2AK2,HLA-A,B2M
GO:0042270	protection from natural killer cell mediated cytotoxicity	2	5	2	6.30E-03	9606.ENSP00000379873,9606.ENSP00000399168	HLA-A,HLA-B
GO:0048583	regulation of response to stimulus	29	3882	0.27	7.20E-03	9606.ENSP00000225698,9606.ENSP00000233057,9606.ENSP00000257604,9606.ENSP00000259206,9606.ENSP00000268058,9606.ENSP00000296161,9606.ENSP00000296577,9606.ENSP00000317144,9606.ENSP00000323889,9606.ENSP00000343023,9606.ENSP00000354394,9606.ENSP00000355330,9606.ENSP00000357459,9606.ENSP00000360860,9606.ENSP00000369213,9606.ENSP00000371958,9606.ENSP00000374455,9606.ENSP00000377344,9606.ENSP00000378217,9606.ENSP00000378786,9606.ENSP00000379038,9606.ENSP00000379873,9606.ENSP00000381167,9606.ENSP00000399168,9606.ENSP00000416561,9606.ENSP00000433138,9606.ENSP00000452780,9606.ENSP00000463188,9606.ENSP00000464087	C1QBP,EIF2AK2,TRAFD1,IL1RN,PML,DTX3L,ABCE1,PIBF1,TRIM25,SP100,STAT1,TGM2,ADAR,IFIT5,DDX58,CD9,SQSTM1,DDX60,PRKCD,HLA-DRA,TYMP,HLA-A,DYNC2H1,HLA-B,CFB,CASP1,B2M,TRIM16,RNF213
GO:0001959	regulation of cytokine-mediated signaling pathway	5	151	0.91	7.70E-03	9606.ENSP00000259206,9606.ENSP00000296577,9606.ENSP00000354394,9606.ENSP00000357459,9606.ENSP00000433138	IL1RN,ABCE1,STAT1,ADAR,CASP1
GO:0048584	positive regulation of response to stimulus	19	2054	0.36	8.30E-03	9606.ENSP00000225698,9606.ENSP00000233057,9606.ENSP00000268058,9606.ENSP00000296161,9606.ENSP00000317144,9606.ENSP00000323889,9606.ENSP00000355330,9606.ENSP00000360860,9606.ENSP00000369213,9606.ENSP00000377344,9606.ENSP00000378217,9606.ENSP00000378786,9606.ENSP00000379873,9606.ENSP00000381167,9606.ENSP00000399168,9606.ENSP00000416561,9606.ENSP00000433138,9606.ENSP00000452780,9606.ENSP00000463188	C1QBP,EIF2AK2,PML,DTX3L,PIBF1,TRIM25,TGM2,IFIT5,DDX58,DDX60,PRKCD,HLA-DRA,HLA-A,DYNC2H1,HLA-B,CFB,CASP1,B2M,TRIM16
GO:0051241	negative regulation of multicellular organismal process	13	1098	0.47	8.40E-03	9606.ENSP00000225698,9606.ENSP00000268058,9606.ENSP00000287156,9606.ENSP00000317144,9606.ENSP00000323889,9606.ENSP00000333266,9606.ENSP00000343023,9606.ENSP00000354394,9606.ENSP00000368699,9606.ENSP00000369213,9606.ENSP00000371958,9606.ENSP00000378217,9606.ENSP00000452780	C1QBP,PML,UBE2L6,PIBF1,TRIM25,UBA7,SP100,STAT1,ISG15,DDX58,CD9,PRKCD,B2M
GO:0039533	regulation of MDA-5 signaling pathway	2	7	1.85	1.01E-02	9606.ENSP00000225698,9606.ENSP00000377344	C1QBP,DDX60
GO:0050706	regulation of interleukin-1 beta secretion	3	37	1.3	1.01E-02	9606.ENSP00000268058,9606.ENSP00000433138,9606.ENSP00000463188	PML,CASP1,TRIM16

GO:0034504	protein localization to nucleus	5	164	0.88	1.05E-02	9606.ENSP00000231487,9606.ENSP00000268058,9606.ENSP00000334373,9606.ENSP00000343023,9606.ENSP00000357459	SKP1,PML,KPNA4,SP100,ADAR
GO:0050778	positive regulation of immune response	9	589	0.58	1.13E-02	9606.ENSP00000225698,9606.ENSP00000369213,9606.ENSP00000377344,9606.ENSP00000378217,9606.ENSP00000378786,9606.ENSP00000379873,9606.ENSP00000399168,9606.ENSP00000416561,9606.ENSP00000452780	C1QBP,DDX58,DDX60,PRKCD,HLA-DRA,HLA-A,HLA-B,CFB,B2M
GO:0019985	translesion synthesis	3	41	1.26	1.26E-02	9606.ENSP00000287156,9606.ENSP00000323889,9606.ENSP00000368699	UBE2L6,TRIM25,ISG15
GO:0051234	establishment of localization	30	4248	0.24	1.29E-02	9606.ENSP00000229379,9606.ENSP00000249066,9606.ENSP00000259206,9606.ENSP00000262776,9606.ENSP00000264020,9606.ENSP00000267113,9606.ENSP00000268058,9606.ENSP00000296161,9606.ENSP00000296577,9606.ENSP00000317144,9606.ENSP00000334373,9606.ENSP00000355330,9606.ENSP00000355599,9606.ENSP00000357459,9606.ENSP00000369965,9606.ENSP00000371958,9606.ENSP00000374455,9606.ENSP00000377470,9606.ENSP00000378217,9606.ENSP00000379873,9606.ENSP00000381167,9606.ENSP00000399168,9606.ENSP00000400591,9606.ENSP00000404833,9606.ENSP00000416066,9606.ENSP00000428085,9606.ENSP00000433138,9606.ENSP00000452780,9606.ENSP00000464342,9606.ENSP00000478026	COX6A1,APOL2,IL1RN,LGALS3BP,IFT46,ESYT1,PML,DTX3L,ABCE1,PIBF1,KPNA4,TGM2,TSNAX,ADAR,BTF3,CD9,SQSTM1,CNP,PRKCD,HLA-A,DYNC2H1,HLA-B,SNRPE,TAPBP,SERPINA1,RPL30,CASP1,B2M,RPL36,ANXA8
GO:0030162	regulation of proteolysis	10	742	0.52	1.40E-02	9606.ENSP00000225698,9606.ENSP00000244061,9606.ENSP00000268058,9606.ENSP00000325677,9606.ENSP00000363993,9606.ENSP00000372155,9606.ENSP00000416066,9606.ENSP00000416561,9606.ENSP00000433138,9606.ENSP00000478026	C1QBP,RNF114,PML,RNF40,PSMB9,PSME1,SERPINA1,CFB,CASP1,ANXA8
GO:0002684	positive regulation of immune system process	11	882	0.49	1.41E-02	9606.ENSP00000225698,9606.ENSP00000354394,9606.ENSP00000368699,9606.ENSP00000369213,9606.ENSP00000377344,9606.ENSP00000378217,9606.ENSP00000378786,9606.ENSP00000379873,9606.ENSP00000399168,9606.ENSP00000416561,9606.ENSP00000452780	C1QBP,STAT1,ISG15,DDX58,DDX60,PRKCD,HLA-DRA,HLA-A,HLA-B,CFB,B2M
GO:0061635	regulation of protein complex stability	2	9	1.74	1.41E-02	9606.ENSP00000374455,9606.ENSP00000404833	SQSTM1,TAPBP
GO:0006953	acute-phase response	3	45	1.22	1.54E-02	9606.ENSP00000249066,9606.ENSP00000259206,9606.ENSP00000416066	APOL2,IL1RN,SERPINA1
GO:0045646	regulation of erythrocyte differentiation	3	46	1.21	1.63E-02	9606.ENSP00000354394,9606.ENSP00000368699,9606.ENSP00000452780	STAT1,ISG15,B2M
GO:0010605	negative regulation of macromolecule metabolic process	21	2558	0.31	1.64E-02	9606.ENSP00000225698,9606.ENSP00000233057,9606.ENSP00000264108,9606.ENSP00000268058,9606.ENSP00000296161,9606.ENSP00000296577,9606.ENSP00000317144,9606.ENSP00000319281,9606.ENSP00000325618,9606.ENSP00000325677,9606.ENSP00000343023,9606.ENSP00000347495,9606.ENSP00000354394,9606.ENSP00000357459,9606.ENSP00000368699,9606.ENSP00000374455,9606.ENSP00000378217,9606.ENSP00000416066,9606.ENSP00000428085,9606.ENSP00000464342,9606.ENSP00000478026	C1QBP,EIF2AK2,HAT1,PML,DTX3L,ABCE1,PIBF1,BASP1,PARP10,RNF40,SP100,WARS,STAT1,ADAR,ISG15,SQSTM1,PRKCD,SERPINA1,RPL30,RPL36,ANXA8
GO:0033523	histone H2B ubiquitination	2	10	1.69	1.64E-02	9606.ENSP00000296161,9606.ENSP00000325677	DTX3L,RNF40

GO:0006810	transport	29	4130	0.24	1.71E-02	9606.ENSP00000229379,9606.ENSP00000249066,9606.ENSP00000259206,9606.ENSP00000262776,9606.ENSP00000264020,9606.ENSP00000267113,9606.ENSP00000268058,9606.ENSP00000296161,9606.ENSP00000296577,9606.ENSP00000334373,9606.ENSP00000355330,9606.ENSP00000355599,9606.ENSP00000357459,9606.ENSP00000369965,9606.ENSP00000371958,9606.ENSP00000374455,9606.ENSP00000377470,9606.ENSP00000378217,9606.ENSP00000379873,9606.ENSP00000381167,9606.ENSP00000399168,9606.ENSP00000400591,9606.ENSP00000404833,9606.ENSP00000416066,9606.ENSP00000428085,9606.ENSP00000433138,9606.ENSP00000452780,9606.ENSP00000464342,9606.ENSP00000478026	COX6A1,APOL2,IL1RN,LGALS3BP,IFT46,ESYT1,PML,DTX3L,ABCE1,KPNA4,TGM2,TSNAX,ADAR,BTF3,CD9,SQSTM1,CNP,PRKCD,HLA-A,DYNC2H1,HLA-B,SNRPE,TAPBP,SERPINA1,RPL30,CASP1,B2M,RPL36,ANXA8
GO:0090331	negative regulation of platelet aggregation	2	11	1.65	1.88E-02	9606.ENSP00000371958,9606.ENSP00000378217	CD9,PRKCD
GO:0016567	protein ubiquitination	9	645	0.54	1.89E-02	9606.ENSP00000231487,9606.ENSP00000244061,9606.ENSP00000287156,9606.ENSP00000296161,9606.ENSP00000323889,9606.ENSP00000325677,9606.ENSP00000333266,9606.ENSP00000379873,9606.ENSP00000464087	SKP1,RNF114,UBE2L6,DTX3L,TRIM25,RNF40,UBA7,HLA-A,RNF213
GO:0033365	protein localization to organelle	9	649	0.54	1.95E-02	9606.ENSP00000231487,9606.ENSP00000268058,9606.ENSP00000317144,9606.ENSP00000334373,9606.ENSP00000343023,9606.ENSP00000357459,9606.ENSP00000381167,9606.ENSP00000428085,9606.ENSP00000464342	SKP1,PML,PIBF1,KPNA4,SP100,ADAR,DYNC2H1,RPL30,RPL36
GO:0051179	localization	34	5233	0.21	1.99E-02	9606.ENSP00000229379,9606.ENSP00000231487,9606.ENSP00000249066,9606.ENSP00000259206,9606.ENSP00000262776,9606.ENSP00000264020,9606.ENSP00000267113,9606.ENSP00000268058,9606.ENSP00000296161,9606.ENSP00000296577,9606.ENSP00000317144,9606.ENSP00000334373,9606.ENSP00000343023,9606.ENSP00000355330,9606.ENSP00000355599,9606.ENSP00000357459,9606.ENSP00000368678,9606.ENSP00000369965,9606.ENSP00000371958,9606.ENSP00000374455,9606.ENSP00000377470,9606.ENSP00000378217,9606.ENSP00000379873,9606.ENSP00000381167,9606.ENSP00000396704,9606.ENSP00000399168,9606.ENSP00000400591,9606.ENSP00000404833,9606.ENSP00000416066,9606.ENSP00000428085,9606.ENSP00000433138,9606.ENSP00000452780,9606.ENSP00000464342,9606.ENSP00000478026	COX6A1,SKP1,APOL2,IL1RN,LGALS3BP,IFT46,ESYT1,PML,DTX3L,ABCE1,PIBF1,KPNA4,SP100,TGM2,TSNAX,ADAR,AGRN,BTF3,CD9,SQSTM1,CNP,PRKCD,HLA-A,DYNC2H1,TOP2B,HLA-B,SNRPE,TAPBP,SERPINA1,RPL30,CASP1,B2M,RPL36,ANXA8
GO:0070498	interleukin-1-mediated signaling pathway	3	51	1.16	2.01E-02	9606.ENSP00000231487,9606.ENSP00000259206,9606.ENSP00000374455	SKP1,IL1RN,SQSTM1
GO:0032655	regulation of interleukin-12 production	3	53	1.15	2.20E-02	9606.ENSP00000225698,9606.ENSP00000317144,9606.ENSP00000399168	C1QBP,PIBF1,HLA-B
GO:0033036	macromolecule localization	19	2268	0.32	2.20E-02	9606.ENSP00000231487,9606.ENSP00000249066,9606.ENSP00000259206,9606.ENSP00000264020,9606.ENSP00000267113,9606.ENSP00000268058,9606.ENSP00000296161,9606.ENSP00000296577,9606.ENSP00000317144,9606.ENSP00000334373,9606.ENSP00000343023,9606.ENSP00000355599,9606.ENSP00000357459,9606.ENSP00000368678,9606.ENSP00000369965,9606.ENSP00000374455,9606.ENSP00000381167,9606.ENSP00000428085,9606.ENSP00000464342	SKP1,APOL2,IL1RN,IFT46,ESYT1,PML,DTX3L,ABCE1,PIBF1,KPNA4,SP100,TSNAX,ADAR,AGRN,BTF3,SQSTM1,DYNC2H1,RPL30,RPL36

GO:0043123	positive regulation of I-kappaB kinase/NF-kappaB signaling	4	122	0.91	2.42E-02	9606.ENSP00000323889,9606.ENSP00000355330,9606.ENSP00000360860,9606.ENSP00000433138	TRIM25,TGM2,IFIT5,CASP1
GO:0044260	cellular macromolecule metabolic process	39	6413	0.18	2.42E-02	9606.ENSP00000225698,9606.ENSP00000231487,9606.ENSP00000233057,9606.ENSP00000244061,9606.ENSP00000264108,9606.ENSP00000268058,9606.ENSP00000287156,9606.ENSP00000296161,9606.ENSP00000296543,9606.ENSP00000296577,9606.ENSP00000306548,9606.ENSP00000317144,9606.ENSP00000323889,9606.ENSP00000325618,9606.ENSP00000325677,9606.ENSP00000333266,9606.ENSP00000342278,9606.ENSP00000343023,9606.ENSP00000347495,9606.ENSP00000354394,9606.ENSP00000355330,9606.ENSP00000363993,9606.ENSP00000366819,9606.ENSP00000368699,9606.ENSP00000369213,9606.ENSP00000369965,9606.ENSP00000371958,9606.ENSP00000372155,9606.ENSP00000374455,9606.ENSP00000378217,9606.ENSP00000379873,9606.ENSP00000396704,9606.ENSP00000400591,9606.ENSP00000416066,9606.ENSP00000428085,9606.ENSP00000452780,9606.ENSP00000463188,9606.ENSP00000464087,9606.ENSP00000464342	C1QBP,SKP1,EIF2AK2,RNF114,HAT1,PML,UBE2L6,DTX3L,NAA15,ABCE1,MRPL13,PIBF1,TRIM25,PARP10,RNF40,UBA7,OAS2,SP100,WARS,STAT1,TGM2,PSMB9,UCHL3,ISG15,DDX58,BTF3,CD9,PSME1,SQSTM1,PRKCD,HLA-A,TOP2B,SNRPE,SERPINA1,RPL30,B2M,TRIM16,RNF213,RPL36
GO:0051170	import into nucleus	4	122	0.91	2.42E-02	9606.ENSP00000268058,9606.ENSP00000334373,9606.ENSP00000357459,9606.ENSP00000400591	PML,KPNA4,ADAR,SNRPE
GO:0043933	protein-containing complex subunit organization	16	1770	0.35	2.51E-02	9606.ENSP00000225698,9606.ENSP00000231487,9606.ENSP00000264108,9606.ENSP00000268058,9606.ENSP00000296577,9606.ENSP00000306548,9606.ENSP00000355330,9606.ENSP00000357459,9606.ENSP00000374455,9606.ENSP00000378786,9606.ENSP00000379873,9606.ENSP00000381601,9606.ENSP00000400591,9606.ENSP00000404833,9606.ENSP00000416066,9606.ENSP00000464087	C1QBP,SKP1,HAT1,PML,ABCE1,MRPL13,TGM2,ADAR,SQSTM1,HLA-DRA,HLA-A,MX1,SNRPE,TAPBP,SERPINA1,RNF213
GO:0044092	negative regulation of molecular function	12	1119	0.42	2.53E-02	9606.ENSP00000259206,9606.ENSP00000268058,9606.ENSP00000296161,9606.ENSP00000296577,9606.ENSP00000325618,9606.ENSP00000343023,9606.ENSP00000347495,9606.ENSP00000357459,9606.ENSP00000378217,9606.ENSP00000416066,9606.ENSP00000452780,9606.ENSP00000478026	IL1RN,PML,DTX3L,ABCE1,PARP10,SP100,WARS,ADAR,PRKCD,SERPINA1,B2M,ANXA8
GO:0051172	negative regulation of nitrogen compound metabolic process	19	2307	0.31	2.56E-02	9606.ENSP00000225698,9606.ENSP00000233057,9606.ENSP00000264108,9606.ENSP00000268058,9606.ENSP00000296161,9606.ENSP00000296577,9606.ENSP00000317144,9606.ENSP00000319281,9606.ENSP00000325618,9606.ENSP00000325677,9606.ENSP00000343023,9606.ENSP00000347495,9606.ENSP00000354394,9606.ENSP00000357459,9606.ENSP00000368699,9606.ENSP00000374455,9606.ENSP00000378217,9606.ENSP00000416066,9606.ENSP00000478026	C1QBP,EIF2AK2,HAT1,PML,DTX3L,ABCE1,PIBF1,BASP1,PARP10,RNF40,SP100,WARS,STAT1,ADAR,ISG15,SQSTM1,PRKCD,SERPINA1,ANXA8
GO:0072075	metanephric mesenchyme development	2	14	1.55	2.58E-02	9606.ENSP00000319281,9606.ENSP00000354394	BASP1,STAT1
GO:0008104	protein localization	17	1966	0.33	2.68E-02	9606.ENSP00000231487,9606.ENSP00000259206,9606.ENSP00000264020,9606.ENSP00000268058,9606.ENSP00000296161,9606.ENSP00000296577,9606.ENSP00000317144,9606.ENSP00000334373,9606.ENSP00000343023,9606.ENSP00000355599,9606.ENSP00000357459,9606.ENSP00000368678,9606.ENSP00000369965,9606.ENSP00000374455,9606.ENSP00000381167,9606.ENSP00000428085,9606.ENSP00000464342	SKP1,IL1RN,IFT46,PML,DTX3L,ABCE1,PIBF1,KPNA4,SP100,TSNAX,ADAR,AGRN,BTF3,SQSTM1,DYNC2H1,RPL30,RPL36

GO:0016045	detection of bacterium	2	15	1.52	2.79E-02	9606.ENSP00000379873,9606.ENSP00000399168	HLA-A,HLA-B
GO:1901698	response to nitrogen compound	11	988	0.44	2.79E-02	9606.ENSP00000323889,9606.ENSP00000325677,9606.ENSP00000354394,9606.ENSP00000357459,9606.ENSP00000358327,9606.ENSP00000368678,9606.ENSP00000369213,9606.ENSP00000371958,9606.ENSP00000378217,9606.ENSP00000433138,9606.ENSP00000463188	TRIM25,RNF40,STAT1,ADAR,CASP7,AGRN,DDX58,CD9,PRKCD,CASP1,TRIM16
GO:0032695	negative regulation of interleukin-12 production	2	16	1.49	3.07E-02	9606.ENSP00000225698,9606.ENSP00000317144	C1QBP,PIBF1
GO:0051641	cellular localization	18	2180	0.31	3.10E-02	9606.ENSP00000231487,9606.ENSP00000264020,9606.ENSP00000267113,9606.ENSP00000268058,9606.ENSP00000296161,9606.ENSP00000296577,9606.ENSP00000317144,9606.ENSP00000334373,9606.ENSP00000343023,9606.ENSP00000357459,9606.ENSP00000368678,9606.ENSP00000374455,9606.ENSP00000381167,9606.ENSP00000400591,9606.ENSP00000416066,9606.ENSP00000428085,9606.ENSP00000464342,9606.ENSP00000478026	SKP1,IFT46,ESYT1,PML,DTX3L,ABCE1,PIBF1,KPNA4,SP100,ADAR,AGRN,SQSTM1,DYNC2H1,SNRPE,SERPINA1,RPL30,RPL36,ANXA8
GO:0032269	negative regulation of cellular protein metabolic process	11	1014	0.43	3.28E-02	9606.ENSP00000233057,9606.ENSP00000268058,9606.ENSP00000296161,9606.ENSP00000317144,9606.ENSP00000325618,9606.ENSP00000347495,9606.ENSP00000357459,9606.ENSP00000368699,9606.ENSP00000378217,9606.ENSP00000416066,9606.ENSP00000478026	EIF2AK2,PML,DTX3L,PIBF1,PARP10,WARS,ADAR,ISG15,PRKCD,SERPINA1,ANXA8
GO:0008631	intrinsic apoptotic signaling pathway in response to oxidative stress	2	17	1.46	3.30E-02	9606.ENSP00000268058,9606.ENSP00000378217	PML,PRKCD
GO:0048384	retinoic acid receptor signaling pathway	2	17	1.46	3.30E-02	9606.ENSP00000268058,9606.ENSP00000343023	PML,SP100
GO:1902187	negative regulation of viral release from host cell	2	17	1.46	3.30E-02	9606.ENSP00000268058,9606.ENSP00000323889	PML,TRIM25
GO:0016570	histone modification	6	347	0.63	3.53E-02	9606.ENSP00000231487,9606.ENSP00000264108,9606.ENSP00000296161,9606.ENSP00000325677,9606.ENSP00000378217,9606.ENSP00000463188	SKP1,HAT1,DTX3L,RNF40,PRKCD,TRIM16
GO:0031397	negative regulation of protein ubiquitination	3	68	1.04	3.53E-02	9606.ENSP00000296161,9606.ENSP00000325618,9606.ENSP00000368699	DTX3L,PARP10,ISG15
GO:0039535	regulation of RIG-I signaling pathway	2	18	1.44	3.53E-02	9606.ENSP00000225698,9606.ENSP00000377344	C1QBP,DDX60
GO:0043555	regulation of translation in response to stress	2	18	1.44	3.53E-02	9606.ENSP00000233057,9606.ENSP00000268058	EIF2AK2,PML
GO:0046597	negative regulation of viral entry into host cell	2	18	1.44	3.53E-02	9606.ENSP00000323889,9606.ENSP00000382707	TRIM25,IFITM3
GO:0065003	protein-containing complex assembly	14	1514	0.36	3.53E-02	9606.ENSP00000225698,9606.ENSP00000231487,9606.ENSP00000264108,9606.ENSP00000268058,9606.ENSP00000355330,9606.ENSP00000357459,9606.ENSP00000374455,9606.ENSP00000378786,9606.ENSP00000379873,9606.ENSP00000381601,9606.ENSP00000400591,9606.ENSP00000404833,9606.ENSP00000416066,9606.ENSP00000464087	C1QBP,SKP1,HAT1,PML,TGM2,ADAR,SQSTM1,HLA-DRA,HLA-A,MX1,SNRPE,TAPBP,SERPINA1,RNF213
GO:0002726	positive regulation of T cell cytokine production	2	19	1.42	3.72E-02	9606.ENSP00000379873,9606.ENSP00000452780	HLA-A,B2M

GO:1902914	regulation of protein polyubiquitination	2	19	1.42	3.72E-02	9606.ENSP00000325618,9606.ENSP00000325677	PARP10,RNF40
GO:0032069	regulation of nuclease activity	2	20	1.39	4.00E-02	9606.ENSP00000296577,9606.ENSP00000378217	ABCE1,PRKCD
GO:0044237	cellular metabolic process	48	8797	0.13	4.00E-02	9606.ENSP00000225698,9606.ENSP00000229379,9606.ENSP00000231487,9606.ENSP00000233057,9606.ENSP00000244061,9606.ENSP00000264108,9606.ENSP00000268058,9606.ENSP00000287156,9606.ENSP00000296161,9606.ENSP00000296543,9606.ENSP00000296577,9606.ENSP00000306548,9606.ENSP00000317144,9606.ENSP00000323889,9606.ENSP00000325618,9606.ENSP00000325677,9606.ENSP00000333019,9606.ENSP00000333266,9606.ENSP00000342278,9606.ENSP00000343023,9606.ENSP00000347495,9606.ENSP00000354394,9606.ENSP00000355330,9606.ENSP00000357459,9606.ENSP00000363993,9606.ENSP00000365950,9606.ENSP00000366819,9606.ENSP00000368678,9606.ENSP00000368699,9606.ENSP00000369213,9606.ENSP00000369965,9606.ENSP00000371958,9606.ENSP00000372155,9606.ENSP00000374455,9606.ENSP00000377470,9606.ENSP00000378217,9606.ENSP00000379038,9606.ENSP00000379873,9606.ENSP00000396704,9606.ENSP00000400591,9606.ENSP00000404833,9606.ENSP00000416066,9606.ENSP00000428085,9606.ENSP00000430344,9606.ENSP00000452780,9606.ENSP00000463188,9606.ENSP00000464087,9606.ENSP00000464342	C1QBP,COX6A1,SKP1,EIF2AK2,RNF114,HAT1,PML,UBE2L6,DTX3L,NAA15,ABCE1,MRPL13,PIBF1,TRIM25,PARP10,RNF40,ADSSL1,UBA7,OAS2,SP100,WARS,STAT1,TGM2,ADAR,PSMB9,RBM3,UCHL3,AGRN,ISG15,DDX58,BTF3,CD9,PSME1,SQSTM1,CNP,PRKCD,TYMP,HLA-A,TOP2B,SNRPE,TAPBP,SERPINA1,RPL30,PLCD1,B2M,TRIM16,RNF213,RPL36
GO:0051240	positive regulation of multicellular organismal process	14	1551	0.35	4.02E-02	9606.ENSP00000225698,9606.ENSP00000233057,9606.ENSP00000268058,9606.ENSP00000317144,9606.ENSP00000319281,9606.ENSP00000354394,9606.ENSP00000368678,9606.ENSP00000368699,9606.ENSP00000369213,9606.ENSP00000377344,9606.ENSP00000379873,9606.ENSP00000433138,9606.ENSP00000452780,9606.ENSP00000463188	C1QBP,EIF2AK2,PML,PIBF1,BASP1,STAT1,AGRN,ISG15,DDX58,DDX60,HLA-A,CASP1,B2M,TRIM16
GO:0006412	translation	6	362	0.61	4.06E-02	9606.ENSP00000233057,9606.ENSP00000296577,9606.ENSP00000306548,9606.ENSP00000347495,9606.ENSP00000428085,9606.ENSP00000464342	EIF2AK2,ABCE1,MRPL13,WARS,RPL30,RPL36
GO:0042742	defense response to bacterium	5	250	0.69	4.06E-02	9606.ENSP00000368699,9606.ENSP00000378217,9606.ENSP00000379873,9606.ENSP00000428085,9606.ENSP00000452780	ISG15,PRKCD,HLA-A,RPL30,B2M
GO:0006959	humoral immune response	5	252	0.69	4.13E-02	9606.ENSP00000225698,9606.ENSP00000379873,9606.ENSP00000416561,9606.ENSP00000428085,9606.ENSP00000452780	C1QBP,HLA-A,CFB,RPL30,B2M
GO:0046907	intracellular transport	13	1390	0.36	4.13E-02	9606.ENSP00000264020,9606.ENSP00000268058,9606.ENSP00000296161,9606.ENSP00000296577,9606.ENSP00000334373,9606.ENSP00000357459,9606.ENSP00000374455,9606.ENSP00000381167,9606.ENSP00000400591,9606.ENSP00000416066,9606.ENSP00000428085,9606.ENSP00000464342,9606.ENSP00000478026	IFT46,PML,DTX3L,ABCE1,KPNA4,ADAR,SQSTM1,DYNC2H1,SNRPE,SERPINA1,RPL30,RPL36,ANXA8
GO:0031324	negative regulation of cellular metabolic process	19	2463	0.28	4.16E-02	9606.ENSP00000225698,9606.ENSP00000233057,9606.ENSP00000264108,9606.ENSP00000268058,9606.ENSP00000296161,9606.ENSP00000296577,9606.ENSP00000317144,9606.ENSP00000319281,9606.ENSP00000325618,9606.ENSP00000325677,9606.ENSP00000343023,9606.ENSP00000347495,9606.ENSP00000354394,9606.ENSP00000357459,9606.ENSP00000368699,9606.ENSP00000374455,9606.ENSP00000378217,9606.ENSP00000416066,9606.ENSP00000478026	C1QBP,EIF2AK2,HAT1,PML,DTX3L,ABCE1,PIBF1,BASP1,PARP10,RNF40,SP100,WARS,STAT1,ADAR,ISG15,SQSTM1,PRKCD,SERPINA1,ANXA8

GO:0051246	regulation of protein metabolic process	20	2668	0.27	4.33E-02	9606.ENSP00000225698,9606.ENSP00000233057,9606.ENSP00000244061,9606.ENSP00000268058,9606.ENSP00000296161,9606.ENSP00000317144,9606.ENSP00000325618,9606.ENSP00000325677,9606.ENSP00000347495,9606.ENSP00000357459,9606.ENSP00000363993,9606.ENSP00000365950,9606.ENSP00000368699,9606.ENSP00000372155,9606.ENSP00000374455,9606.ENSP00000378217,9606.ENSP00000416066,9606.ENSP00000416561,9606.ENSP00000433138,9606.ENSP00000478026	C1QBP,EIF2AK2,RNF114,PML,DTX3L,PIBF1,PARP10,RNF40,WARS,ADAR,PSMB9,RBM3,ISG15,PSME1,SQSTM1,PRKCD,SERPINA1,CFB,CASP1,ANXA8
GO:0006518	peptide metabolic process	7	497	0.54	4.38E-02	9606.ENSP00000233057,9606.ENSP00000296577,9606.ENSP00000306548,9606.ENSP00000347495,9606.ENSP00000404833,9606.ENSP00000428085,9606.ENSP00000464342	EIF2AK2,ABCE1,MRPL13,WARS,TAPBP,RPL30,RPL36
GO:0006913	nucleocytoplasmic transport	5	263	0.67	4.74E-02	9606.ENSP00000268058,9606.ENSP00000296577,9606.ENSP00000334373,9606.ENSP00000357459,9606.ENSP00000400591	PML,ABCE1,KPNA4,ADAR,SNRPE
GO:0007165	signal transduction	30	4738	0.2	4.74E-02	9606.ENSP00000225698,9606.ENSP00000231487,9606.ENSP00000233057,9606.ENSP00000257570,9606.ENSP00000259206,9606.ENSP00000262776,9606.ENSP00000264020,9606.ENSP00000268058,9606.ENSP00000323889,9606.ENSP00000342278,9606.ENSP00000343023,9606.ENSP00000354394,9606.ENSP00000355330,9606.ENSP00000357459,9606.ENSP00000360883,9606.ENSP00000368678,9606.ENSP00000368699,9606.ENSP00000369213,9606.ENSP00000371958,9606.ENSP00000374455,9606.ENSP00000378217,9606.ENSP00000378786,9606.ENSP00000379873,9606.ENSP00000381601,9606.ENSP00000382707,9606.ENSP00000395590,9606.ENSP00000399168,9606.ENSP00000430344,9606.ENSP00000433138,9606.ENSP00000452780	C1QBP,SKP1,EIF2AK2,OASL,IL1RN,LGALS3BP,IFT46,PML,TRIM25,OAS2,SP100,STAT1,TGM2,ADAR,IFIT3,AGRN,ISG15,DDX58,CD9,SQSTM1,PRKCD,HLA-DRA,HLA-A,MX1,IFITM3,IFI35,HLA-B,PLCD1,CASP1,B2M
GO:0032469	endoplasmic reticulum calcium ion homeostasis	2	23	1.33	4.74E-02	9606.ENSP00000268058,9606.ENSP00000355330	PML,TGM2
GO:0032727	positive regulation of interferon-alpha production	2	23	1.33	4.74E-02	9606.ENSP00000354394,9606.ENSP00000369213	STAT1,DDX58
GO:0033522	histone H2A ubiquitination	2	23	1.33	4.74E-02	9606.ENSP00000231487,9606.ENSP00000296161	SKP1,DTX3L
GO:0035456	response to interferon-beta	2	23	1.33	4.74E-02	9606.ENSP00000354394,9606.ENSP00000382707	STAT1,IFITM3
GO:0072215	regulation of metanephros development	2	23	1.33	4.74E-02	9606.ENSP00000319281,9606.ENSP00000354394	BASP1,STAT1
GO:0032897	negative regulation of viral transcription	2	24	1.31	4.91E-02	9606.ENSP00000343023,9606.ENSP00000382707	SP100,IFITM3
GO:0050821	protein stabilization	4	166	0.78	4.91E-02	9606.ENSP00000264020,9606.ENSP00000268058,9606.ENSP00000296543,9606.ENSP00000378217	IFT46,PML,NAA15,PRKCD
GO:0042886	amide transport	13	1440	0.35	4.96E-02	9606.ENSP00000259206,9606.ENSP00000264020,9606.ENSP00000268058,9606.ENSP00000296161,9606.ENSP00000296577,9606.ENSP00000334373,9606.ENSP00000355599,9606.ENSP00000357459,9606.ENSP00000369965,9606.ENSP00000381167,9606.ENSP00000404833,9606.ENSP00000428085,9606.ENSP00000464342	IL1RN,IFT46,PML,DTX3L,ABCE1,KPNA4,TSNAX,ADAR,BTF3,DYNC2H1,TAPBP,RPL30,RPL36

T = 72 h (mock) IFNα7 / Untreated

#Term ID	Term Description	Observed Gene Count	Background Gene Count	Strength	False Discovery Rate	Matching Proteins in Your Input (IDs)	Matching Proteins in Your Input (Labels)
GO:0034340	response to type I interferon	9	70	1.6	5.91E-09	9606.ENSP00000253110,9606.ENSP00000342278,9606.ENSP00000354394,9606.ENSP00000360883,9606.ENSP00000365402,9606.ENSP00000368699,9606.ENSP00000381601,9606.ENSP00000395590,9606.ENSP00000399168	C19orf66,OAS2,STAT1,IFIT3,HLA-C,ISG15,MX1,IFI35,HLA-B
GO:0009615	response to virus	12	270	1.14	6.97E-08	9606.ENSP00000233057,9606.ENSP00000253110,9606.ENSP00000268058,9606.ENSP00000342278,9606.ENSP00000354394,9606.ENSP00000359783,9606.ENSP00000359787,9606.ENSP00000360883,9606.ENSP00000368699,9606.ENSP00000369213,9606.ENSP00000381601,9606.ENSP00000399888	EIF2AK2,C19orf66,PML,OAS2,STAT1,IFI44,IFI44L,IFIT3,ISG15,DDX58,MX1,HMGA1
GO:0060337	type I interferon signaling pathway	8	65	1.58	6.97E-08	9606.ENSP00000342278,9606.ENSP00000354394,9606.ENSP00000360883,9606.ENSP00000365402,9606.ENSP00000368699,9606.ENSP00000381601,9606.ENSP00000395590,9606.ENSP00000399168	OAS2,STAT1,IFIT3,HLA-C,ISG15,MX1,IFI35,HLA-B
GO:0051607	defense response to virus	10	181	1.23	1.69E-07	9606.ENSP00000233057,9606.ENSP00000253110,9606.ENSP00000268058,9606.ENSP00000342278,9606.ENSP00000354394,9606.ENSP00000359787,9606.ENSP00000360883,9606.ENSP00000368699,9606.ENSP00000369213,9606.ENSP00000381601	EIF2AK2,C19orf66,PML,OAS2,STAT1,IFI44L,IFIT3,ISG15,DDX58,MX1
GO:0006952	defense response	20	1234	0.7	3.03E-07	9606.ENSP00000233057,9606.ENSP00000253110,9606.ENSP00000259206,9606.ENSP00000263636,9606.ENSP00000268058,9606.ENSP00000342278,9606.ENSP00000354394,9606.ENSP00000358045,9606.ENSP00000359490,9606.ENSP00000359787,9606.ENSP00000360883,9606.ENSP00000365402,9606.ENSP00000368699,9606.ENSP00000369213,9606.ENSP00000381601,9606.ENSP00000384886,9606.ENSP00000395590,9606.ENSP00000399168,9606.ENSP00000428085,9606.ENSP00000478874	EIF2AK2,C19orf66,IL1RN,LY75,PML,OAS2,STAT1,ECM1,GBP4,IFI44L,IFIT3,HLA-C,ISG15,DDX58,MX1,IFI30,IFI35,HLA-B,RPL30,CAMK1D
GO:0051707	response to other organism	19	1173	0.7	7.72E-07	9606.ENSP00000233057,9606.ENSP00000253110,9606.ENSP00000268058,9606.ENSP00000312988,9606.ENSP00000342278,9606.ENSP00000354394,9606.ENSP00000359490,9606.ENSP00000359783,9606.ENSP00000359787,9606.ENSP00000360883,9606.ENSP00000365402,9606.ENSP00000368699,9606.ENSP00000369213,9606.ENSP00000381601,9606.ENSP00000384886,9606.ENSP00000395590,9606.ENSP00000399168,9606.ENSP00000399888,9606.ENSP00000428085	EIF2AK2,C19orf66,PML,NFKBIB,OAS2,STAT1,GBP4,IFI44,IFI44L,IFIT3,HLA-C,ISG15,DDX58,MX1,IFI30,IFI35,HLA-B,HMGA1,RPL30
GO:0098542	defense response to other organism	16	859	0.76	1.86E-06	9606.ENSP00000233057,9606.ENSP00000253110,9606.ENSP00000268058,9606.ENSP00000342278,9606.ENSP00000354394,9606.ENSP00000359490,9606.ENSP00000359787,9606.ENSP00000360883,9606.ENSP00000365402,9606.ENSP00000368699,9606.ENSP00000369213,9606.ENSP00000381601,9606.ENSP00000384886,9606.ENSP00000395590,9606.ENSP00000399168,9606.ENSP00000428085	EIF2AK2,C19orf66,PML,OAS2,STAT1,GBP4,IFI44L,IFIT3,HLA-C,ISG15,DDX58,MX1,IFI30,IFI35,HLA-B,RPL30

GO:0045087	innate immune response	14	676	0.81	4.57E-06	9606.ENSP00000233057,9606.ENSP00000253110,9606.ENSP00000268058,9606.ENSP00000342278,9606.ENSP00000354394,9606.ENSP00000359490,9606.ENSP00000360883,9606.ENSP00000365402,9606.ENSP00000368699,9606.ENSP00000369213,9606.ENSP00000381601,9606.ENSP00000384886,9606.ENSP00000395590,9606.ENSP00000399168	EIF2AK2,C19orf66,PML,OAS2,STAT1,GBP4,IFIT3,HLA-C,ISG15,DDX58,MX1,IFI30,IFI35,HLA-B
GO:0048525	negative regulation of viral process	7	93	1.37	4.57E-06	9606.ENSP00000233057,9606.ENSP00000253110,9606.ENSP00000268058,9606.ENSP00000325618,9606.ENSP00000354394,9606.ENSP00000368699,9606.ENSP00000381601	EIF2AK2,C19orf66,PML,PARP10,STAT1,ISG15,MX1
GO:0034341	response to interferon-gamma	8	176	1.15	1.69E-05	9606.ENSP00000253110,9606.ENSP00000268058,9606.ENSP00000342278,9606.ENSP00000354394,9606.ENSP00000359490,9606.ENSP00000365402,9606.ENSP00000384886,9606.ENSP00000399168	C19orf66,PML,OAS2,STAT1,GBP4,HLA-C,IFI30,HLA-B
GO:0060333	interferon-gamma-mediated signaling pathway	6	69	1.43	1.78E-05	9606.ENSP00000268058,9606.ENSP00000342278,9606.ENSP00000354394,9606.ENSP00000365402,9606.ENSP00000384886,9606.ENSP00000399168	PML,OAS2,STAT1,HLA-C,IFI30,HLA-B
GO:0006955	immune response	19	1560	0.58	3.05E-05	9606.ENSP00000233057,9606.ENSP00000253110,9606.ENSP00000259206,9606.ENSP00000261811,9606.ENSP00000263636,9606.ENSP00000268058,9606.ENSP00000342278,9606.ENSP00000354394,9606.ENSP00000359490,9606.ENSP00000360883,9606.ENSP00000365402,9606.ENSP00000366525,9606.ENSP00000368699,9606.ENSP00000369213,9606.ENSP00000381601,9606.ENSP00000384886,9606.ENSP00000395590,9606.ENSP00000399168,9606.ENSP00000428085	EIF2AK2,C19orf66,IL1RN,CYSTM1,LY75,PML,OAS2,STAT1,GBP4,IFIT3,HLA-C,FTL,ISG15,DDX58,MX1,IFI30,IFI35,HLA-B,RPL30
GO:1903901	negative regulation of viral life cycle	6	77	1.38	3.05E-05	9606.ENSP00000233057,9606.ENSP00000253110,9606.ENSP00000268058,9606.ENSP00000325618,9606.ENSP00000368699,9606.ENSP00000381601	EIF2AK2,C19orf66,PML,PARP10,ISG15,MX1
GO:0043901	negative regulation of multi-organism process	8	212	1.07	5.05E-05	9606.ENSP00000233057,9606.ENSP00000253110,9606.ENSP00000268058,9606.ENSP00000325618,9606.ENSP00000354394,9606.ENSP00000368699,9606.ENSP00000381601,9606.ENSP00000399168	EIF2AK2,C19orf66,PML,PARP10,STAT1,ISG15,MX1,HLA-B
GO:0071346	cellular response to interferon-gamma	7	155	1.15	8.20E-05	9606.ENSP00000268058,9606.ENSP00000342278,9606.ENSP00000354394,9606.ENSP00000359490,9606.ENSP00000365402,9606.ENSP00000384886,9606.ENSP00000399168	PML,OAS2,STAT1,GBP4,HLA-C,IFI30,HLA-B
GO:0045071	negative regulation of viral genome replication	5	52	1.48	9.65E-05	9606.ENSP00000233057,9606.ENSP00000253110,9606.ENSP00000325618,9606.ENSP00000368699,9606.ENSP00000381601	EIF2AK2,C19orf66,PARP10,ISG15,MX1
GO:0002252	immune effector process	14	927	0.67	1.10E-04	9606.ENSP00000233057,9606.ENSP00000253110,9606.ENSP00000261811,9606.ENSP00000268058,9606.ENSP00000342278,9606.ENSP00000354394,9606.ENSP00000359787,9606.ENSP00000360883,9606.ENSP00000365402,9606.ENSP00000366525,9606.ENSP00000368699,9606.ENSP00000369213,9606.ENSP00000381601,9606.ENSP00000399168	EIF2AK2,C19orf66,CYSTM1,PML,OAS2,STAT1,IFI44L,IFIT3,HLA-C,FTL,ISG15,DDX58,MX1,HLA-B

GO:0009605	response to external stimulus	21	2152	0.48	1.60E-04	9606.ENSP00000220676,9606.ENSP00000233057,9606.ENSP00000253110,9606.ENSP00000268058,9606.ENSP00000312988,9606.ENSP00000342278,9606.ENSP00000354394,9606.ENSP00000359490,9606.ENSP00000359783,9606.ENSP00000359787,9606.ENSP00000360883,9606.ENSP00000365402,9606.ENSP00000368699,9606.ENSP00000369213,9606.ENSP00000379038,9606.ENSP00000381601,9606.ENSP00000384886,9606.ENSP00000395590,9606.ENSP00000399168,9606.ENSP00000399888,9606.ENSP00000428085	RP1,EIF2AK2,C19orf66,PML,NFKBIB,OAS2,STAT1,GBP4,IFI44,IFI44L,IFIT3,HLA-C,ISG15,DDX58,TYMP,MX1,IFI30,IFI35,HLA-B,HMGA1,RPL30
GO:0034097	response to cytokine	14	1035	0.62	3.40E-04	9606.ENSP00000233057,9606.ENSP00000253110,9606.ENSP00000259206,9606.ENSP00000268058,9606.ENSP00000342278,9606.ENSP00000354394,9606.ENSP00000359490,9606.ENSP00000360883,9606.ENSP00000365402,9606.ENSP00000368699,9606.ENSP00000381601,9606.ENSP00000384886,9606.ENSP00000395590,9606.ENSP00000399168	EIF2AK2,C19orf66,IL1RN,PML,OAS2,STAT1,GBP4,IFIT3,HLA-C,ISG15,MX1,IFI30,IFI35,HLA-B
GO:0051704	multi-organism process	22	2514	0.43	4.30E-04	9606.ENSP00000233057,9606.ENSP00000253110,9606.ENSP00000268058,9606.ENSP00000296577,9606.ENSP00000312988,9606.ENSP00000342278,9606.ENSP00000354394,9606.ENSP00000359490,9606.ENSP00000359783,9606.ENSP00000359787,9606.ENSP00000360883,9606.ENSP00000361930,9606.ENSP00000365402,9606.ENSP00000368699,9606.ENSP00000369213,9606.ENSP00000373450,9606.ENSP00000381601,9606.ENSP00000384886,9606.ENSP00000395590,9606.ENSP00000399168,9606.ENSP00000399888,9606.ENSP00000428085	EIF2AK2,C19orf66,PML,ABCE1,NFKBIB,OAS2,STAT1,GBP4,IFI44,IFI44L,IFIT3,YWHAB,HLA-C,ISG15,DDX58,SPAG1,MX1,IFI30,IFI35,HLA-B,HMGA1,RPL30
GO:0019221	cytokine-mediated signaling pathway	11	655	0.72	5.00E-04	9606.ENSP00000259206,9606.ENSP00000268058,9606.ENSP00000342278,9606.ENSP00000354394,9606.ENSP00000360883,9606.ENSP00000365402,9606.ENSP00000368699,9606.ENSP00000381601,9606.ENSP00000384886,9606.ENSP00000395590,9606.ENSP00000399168	IL1RN,PML,OAS2,STAT1,IFIT3,HLA-C,ISG15,MX1,IFI30,IFI35,HLA-B
GO:0002376	immune system process	21	2370	0.44	5.90E-04	9606.ENSP00000233057,9606.ENSP00000253110,9606.ENSP00000259206,9606.ENSP00000261811,9606.ENSP00000263636,9606.ENSP00000268058,9606.ENSP00000342278,9606.ENSP00000354394,9606.ENSP00000359490,9606.ENSP00000359787,9606.ENSP00000360883,9606.ENSP00000365402,9606.ENSP00000366525,9606.ENSP00000368699,9606.ENSP00000369213,9606.ENSP00000372155,9606.ENSP00000381601,9606.ENSP00000384886,9606.ENSP00000395590,9606.ENSP00000399168,9606.ENSP00000428085	EIF2AK2,C19orf66,IL1RN,CYSTM1,LY75,PML,OAS2,STAT1,GBP4,IFI44L,IFIT3,HLA-C,FTL,ISG15,DDX58,PSME1,MX1,IFI30,IFI35,HLA-B,RPL30
GO:0016032	viral process	10	571	0.74	8.50E-04	9606.ENSP00000233057,9606.ENSP00000268058,9606.ENSP00000296577,9606.ENSP00000354394,9606.ENSP00000361930,9606.ENSP00000365402,9606.ENSP00000368699,9606.ENSP00000369213,9606.ENSP00000399168,9606.ENSP00000399888	EIF2AK2,PML,ABCE1,STAT1,YWHAB,HLA-C,ISG15,DDX58,HLA-B,HMGA1
GO:0044419	interspecies interaction between organisms	11	724	0.67	1.10E-03	9606.ENSP00000233057,9606.ENSP00000268058,9606.ENSP00000296577,9606.ENSP00000354394,9606.ENSP00000361930,9606.ENSP00000365402,9606.ENSP00000368699,9606.ENSP00000369213,9606.ENSP00000399168,9606.ENSP00000399888,9606.ENSP00000428085	EIF2AK2,PML,ABCE1,STAT1,YWHAB,HLA-C,ISG15,DDX58,HLA-B,HMGA1,RPL30

GO:0006950	response to stress	24	3267	0.36	2.20E-03	9606.ENSP00000233057,9606.ENSP00000253110,9606.ENSP00000259206,9606.ENSP00000263636,9606.ENSP00000268058,9606.ENSP00000305480,9606.ENSP00000342278,9606.ENSP00000348278,9606.ENSP00000354394,9606.ENSP00000358045,9606.ENSP00000359490,9606.ENSP00000359787,9606.ENSP00000360883,9606.ENSP00000365402,9606.ENSP00000368699,9606.ENSP00000369213,9606.ENSP00000381601,9606.ENSP00000384886,9606.ENSP00000395590,9606.ENSP00000399168,9606.ENSP00000399888,9606.ENSP00000428085,9606.ENSP00000478874,9606.ENSP00000478927	EIF2AK2,C19orf66,IL1RN,LY75,PML,FEN1,OAS2,STK39,STAT1,ECM1,GBP4,IFI44L,IFIT3,HLA-C,ISG15,DDX58,MX1,IFI30,IFI35,HLA-B,HMGA1,RPL30,CAMK1D,YAP1
GO:0043900	regulation of multi-organism process	10	653	0.68	2.30E-03	9606.ENSP00000233057,9606.ENSP00000253110,9606.ENSP00000268058,9606.ENSP00000296577,9606.ENSP00000325618,9606.ENSP00000354394,9606.ENSP00000368699,9606.ENSP00000369213,9606.ENSP00000381601,9606.ENSP00000399168	EIF2AK2,C19orf66,PML,ABCE1,PARP10,STAT1,ISG15,DDX58,MX1,HLA-B
GO:0071345	cellular response to cytokine stimulus	12	953	0.59	2.40E-03	9606.ENSP00000259206,9606.ENSP00000268058,9606.ENSP00000342278,9606.ENSP00000354394,9606.ENSP00000359490,9606.ENSP00000360883,9606.ENSP00000365402,9606.ENSP00000368699,9606.ENSP00000381601,9606.ENSP00000384886,9606.ENSP00000395590,9606.ENSP00000399168	IL1RN,PML,OAS2,STAT1,GBP4,IFIT3,HLA-C,ISG15,MX1,IFI30,IFI35,HLA-B
GO:0002486	antigen processing and presentation of endogenous peptide antigen via MHC class I via ER pathway, TAP-independent	2	3	2.32	4.80E-03	9606.ENSP00000365402,9606.ENSP00000399168	HLA-C,HLA-B
GO:0002230	positive regulation of defense response to virus by host	3	26	1.55	4.90E-03	9606.ENSP00000268058,9606.ENSP00000354394,9606.ENSP00000369213	PML,STAT1,DDX58
GO:0042590	antigen processing and presentation of exogenous peptide antigen via MHC class I	3	29	1.51	6.40E-03	9606.ENSP00000365402,9606.ENSP00000384886,9606.ENSP00000399168	HLA-C,IFI30,HLA-B
GO:0060759	regulation of response to cytokine stimulus	5	162	0.98	8.50E-03	9606.ENSP00000259206,9606.ENSP00000296577,9606.ENSP00000354394,9606.ENSP00000358045,9606.ENSP00000369213	IL1RN,ABCE1,STAT1,ECM1,DDX58
GO:0001578	microtubule bundle formation	4	87	1.15	8.80E-03	9606.ENSP00000220676,9606.ENSP00000298292,9606.ENSP00000353785,9606.ENSP00000373450	RP1,DNAAF2,TPPP,SPAG1
GO:0072307	regulation of metanephric nephron tubule epithelial cell differentiation	2	7	1.95	1.44E-02	9606.ENSP00000354394,9606.ENSP00000478927	STAT1,YAP1
GO:0002480	antigen processing and presentation of exogenous peptide antigen via MHC class I, TAP-independent	2	8	1.89	1.71E-02	9606.ENSP00000365402,9606.ENSP00000399168	HLA-C,HLA-B
GO:0045646	regulation of erythrocyte differentiation	3	46	1.31	1.95E-02	9606.ENSP00000341422,9606.ENSP00000354394,9606.ENSP00000368699	P4HTM,STAT1,ISG15
GO:0019884	antigen processing and presentation of exogenous antigen	4	130	0.98	3.18E-02	9606.ENSP00000365402,9606.ENSP00000372155,9606.ENSP00000384886,9606.ENSP00000399168	HLA-C,PSME1,IFI30,HLA-B
GO:0035082	axoneme assembly	3	59	1.2	3.56E-02	9606.ENSP00000220676,9606.ENSP00000298292,9606.ENSP00000373450	RP1,DNAAF2,SPAG1

GO:0002682	regulation of immune system process	12	1391	0.43	4.82E-02	9606.ENSP00000233057,9606.ENSP00000268058,9606.ENSP00000296577,9606.ENSP00000341422,9606.ENSP00000354394,9606.ENSP00000358045,9606.ENSP00000365402,9606.ENSP00000368699,9606.ENSP00000369213,9606.ENSP00000399168,9606.ENSP00000478874,9606.ENSP00000478927	EIF2AK2,PML,ABCE1,P4HTM,STAT1,ECM1,HLA-C,ISG15,DDX58,HLA-B,CAMK1D,YAP1
GO:0071310	cellular response to organic substance	16	2219	0.35	4.82E-02	9606.ENSP00000233057,9606.ENSP00000259206,9606.ENSP00000268058,9606.ENSP00000312988,9606.ENSP00000342278,9606.ENSP00000354394,9606.ENSP00000359490,9606.ENSP00000360883,9606.ENSP00000365402,9606.ENSP00000368699,9606.ENSP00000369213,9606.ENSP00000381601,9606.ENSP00000384886,9606.ENSP00000395590,9606.ENSP00000399168,9606.ENSP00000478927	EIF2AK2,IL1RN,PML,NFKBIB,OAS2,STAT1,GBP4,IFIT3,HLA-C,ISG15,DDX58,MX1,IFI30,IFI35,HLA-B,YAP1
GO:0001959	regulation of cytokine-mediated signaling pathway	4	151	0.92	4.85E-02	9606.ENSP00000259206,9606.ENSP00000296577,9606.ENSP00000354394,9606.ENSP00000358045	IL1RN,ABCE1,STAT1,ECM1

T = 72 h (mock) IFNα16 / Untreated

No significantly enriched GO categories

T = 72 h (mock) IFNλ3 / Untreated

#Term ID	Term Description	Observed Gene Count	Background Gene Count	Strength	False Discovery Rate	Matching Proteins in Your Input (IDs)	Matching Proteins in Your Input (Labels)
GO:0060337	type I interferon signaling pathway	11	65	1.67	6.04E-12	9606.ENSP00000257570,9606.ENSP00000342278,9606.ENSP00000343023,9606.ENSP00000354394,9606.ENSP00000360883,9606.ENSP00000368699,9606.ENSP00000379873,9606.ENSP00000381601,9606.ENSP00000382707,9606.ENSP00000395590,9606.ENSP00000399168	OASL,OAS2,SP100,STAT1,IFIT3,ISG15,HLA-A,MX1,IFITM3,IFI35,HLA-B
GO:0009615	response to virus	15	270	1.18	4.22E-11	9606.ENSP00000233057,9606.ENSP00000257570,9606.ENSP00000268058,9606.ENSP00000323889,9606.ENSP00000342278,9606.ENSP00000354394,9606.ENSP00000359783,9606.ENSP00000359787,9606.ENSP00000360883,9606.ENSP00000368699,9606.ENSP00000369213,9606.ENSP00000377344,9606.ENSP00000381601,9606.ENSP00000382707,9606.ENSP00000399888	EIF2AK2,OASL,PML,TRIM25,OAS2,STAT1,IFI44,IFI44L,IFIT3,ISG15,DDX58,DDX60,MX1,IFITM3,HMGA1
GO:0051607	defense response to virus	13	181	1.3	8.29E-11	9606.ENSP00000233057,9606.ENSP00000257570,9606.ENSP00000268058,9606.ENSP00000323889,9606.ENSP00000342278,9606.ENSP00000354394,9606.ENSP00000359787,9606.ENSP00000360883,9606.ENSP00000368699,9606.ENSP00000369213,9606.ENSP00000377344,9606.ENSP00000381601,9606.ENSP00000382707	EIF2AK2,OASL,PML,TRIM25,OAS2,STAT1,IFI44L,IFIT3,ISG15,DDX58,DDX60,MX1,IFITM3
GO:0045087	innate immune response	20	676	0.91	1.06E-10	9606.ENSP00000233057,9606.ENSP00000243253,9606.ENSP00000257570,9606.ENSP00000268058,9606.ENSP00000323889,9606.ENSP00000342278,9606.ENSP00000343023,9606.ENSP00000354394,9606.ENSP00000357722,9606.ENSP00000359490,9606.ENSP00000360883,9606.ENSP00000368699,9606.ENSP00000369213,9606.ENSP00000377344,9606.ENSP00000378217,9606.ENSP00000379873,9606.ENSP00000381601,9606.ENSP00000382707,9606.ENSP00000395590,9606.ENSP00000399168	EIF2AK2,SEC61A1,OASL,PML,TRIM25,OAS2,SP100,STAT1,S100A8,GBP4,IFIT3,ISG15,DDX58,DDX60,PRKCD,HLA-A,MX1,IFITM3,IFI35,HLA-B
GO:0051707	response to other organism	24	1173	0.75	5.43E-10	9606.ENSP00000233057,9606.ENSP00000243253,9606.ENSP00000257570,9606.ENSP00000264346,9606.ENSP00000268058,9606.ENSP00000323889,9606.ENSP00000342278,9606.ENSP00000343023,9606.ENSP00000354394,9606.ENSP00000357722,9606.ENSP00000359490,9606.ENSP00000359783,9606.ENSP00000359787,9606.ENSP00000360883,9606.ENSP00000368699,9606.ENSP00000369213,9606.ENSP00000377344,9606.ENSP00000378217,9606.ENSP00000379873,9606.ENSP00000381601,9606.ENSP00000382707,9606.ENSP00000395590,9606.ENSP00000399168,9606.ENSP00000399888	EIF2AK2,SEC61A1,OASL,HERC6,PML,TRIM25,OAS2,SP100,STAT1,S100A8,GBP4,IFI44,IFI44L,IFIT3,ISG15,DDX58,DDX60,PRKCD,HLA-A,MX1,IFITM3,IFI35,HLA-B,HMGA1
GO:0098542	defense response to other organism	21	859	0.83	5.67E-10	9606.ENSP00000233057,9606.ENSP00000243253,9606.ENSP00000257570,9606.ENSP00000268058,9606.ENSP00000323889,9606.ENSP00000342278,9606.ENSP00000343023,9606.ENSP00000354394,9606.ENSP00000357722,9606.ENSP00000359490,9606.ENSP00000359787,9606.ENSP00000360883,9606.ENSP00000368699,9606.ENSP00000369213,9606.ENSP00000377344,9606.ENSP00000378217,9606.ENSP00000379873,9606.ENSP00000381601,9606.ENSP00000382707,9606.ENSP00000395590,9606.ENSP00000399168	EIF2AK2,SEC61A1,OASL,PML,TRIM25,OAS2,SP100,STAT1,S100A8,GBP4,IFI44L,IFIT3,ISG15,DDX58,DDX60,PRKCD,HLA-A,MX1,IFITM3,IFI35,HLA-B

GO:0034341	response to interferon-gamma	12	176	1.27	6.74E-10	9606.ENSP00000243253,9606.ENSP00000257570,9606.ENSP00000268058,9606.ENSP00000323889,9606.ENSP00000342278,9606.ENSP00000343023,9606.ENSP00000354394,9606.ENSP00000359490,9606.ENSP00000378217,9606.ENSP00000379873,9606.ENSP00000382707,9606.ENSP00000399168	SEC61A1,OASL,PML,TRIM25,OAS2,SP100,STAT1,GBP4,PRKCD,HLA-A,IFITM3,HLA-B
GO:0048525	negative regulation of viral process	10	93	1.47	6.74E-10	9606.ENSP00000233057,9606.ENSP00000257570,9606.ENSP00000268058,9606.ENSP00000323889,9606.ENSP00000325618,9606.ENSP00000343023,9606.ENSP00000354394,9606.ENSP00000368699,9606.ENSP00000381601,9606.ENSP00000382707	EIF2AK2,OASL,PML,TRIM25,PARP10,SP100,STAT1,ISG15,MX1,IFITM3
GO:0060333	interferon-gamma-mediated signaling pathway	9	69	1.56	1.49E-09	9606.ENSP00000257570,9606.ENSP00000268058,9606.ENSP00000323889,9606.ENSP00000342278,9606.ENSP00000343023,9606.ENSP00000354394,9606.ENSP00000378217,9606.ENSP00000379873,9606.ENSP00000399168	OASL,PML,TRIM25,OAS2,SP100,STAT1,PRKCD,HLA-A,HLA-B
GO:0043901	negative regulation of multi-organism process	12	212	1.19	3.78E-09	9606.ENSP00000233057,9606.ENSP00000257570,9606.ENSP00000268058,9606.ENSP00000323889,9606.ENSP00000325618,9606.ENSP00000343023,9606.ENSP00000354394,9606.ENSP00000368699,9606.ENSP00000379873,9606.ENSP00000381601,9606.ENSP00000382707,9606.ENSP00000399168	EIF2AK2,OASL,PML,TRIM25,PARP10,SP100,STAT1,ISG15,HLA-A,MX1,IFITM3,HLA-B
GO:0006952	defense response	23	1234	0.71	5.37E-09	9606.ENSP00000233057,9606.ENSP00000243253,9606.ENSP00000249066,9606.ENSP00000257570,9606.ENSP00000259206,9606.ENSP00000268058,9606.ENSP00000323889,9606.ENSP00000342278,9606.ENSP00000343023,9606.ENSP00000354394,9606.ENSP00000357722,9606.ENSP00000359490,9606.ENSP00000359787,9606.ENSP00000360883,9606.ENSP00000368699,9606.ENSP00000369213,9606.ENSP00000377344,9606.ENSP00000378217,9606.ENSP00000379873,9606.ENSP00000381601,9606.ENSP00000382707,9606.ENSP00000395590,9606.ENSP00000399168	EIF2AK2,SEC61A1,APOL2,OASL,IL1RN,PML,TRIM25,OAS2,SP100,STAT1,S100A8,GBP4,IFI44L,IFIT3,ISG15,DDX58,DDX60,PRKCD,HLA-A,MX1,IFITM3,IFI35,HLA-B
GO:0002252	immune effector process	20	927	0.77	1.07E-08	9606.ENSP00000233057,9606.ENSP00000257570,9606.ENSP00000263946,9606.ENSP00000268058,9606.ENSP00000323889,9606.ENSP00000342278,9606.ENSP00000349687,9606.ENSP00000354394,9606.ENSP00000357722,9606.ENSP00000359787,9606.ENSP00000360883,9606.ENSP00000368699,9606.ENSP00000369213,9606.ENSP00000377344,9606.ENSP00000378217,9606.ENSP00000379873,9606.ENSP00000381601,9606.ENSP00000382707,9606.ENSP00000399168,9606.ENSP00000408411	EIF2AK2,OASL,PKP1,PML,TRIM25,OAS2,GM2A,STAT1,S100A8,IFI44L,IFIT3,ISG15,DDX58,DDX60,PRKCD,HLA-A,MX1,IFITM3,HLA-B,LAMP2
GO:0006955	immune response	25	1560	0.65	1.24E-08	9606.ENSP00000233057,9606.ENSP00000243253,9606.ENSP00000257570,9606.ENSP00000259206,9606.ENSP00000263946,9606.ENSP00000268058,9606.ENSP00000323889,9606.ENSP00000342278,9606.ENSP00000343023,9606.ENSP00000349687,9606.ENSP00000354394,9606.ENSP00000357722,9606.ENSP00000359490,9606.ENSP00000360883,9606.ENSP00000368699,9606.ENSP00000369213,9606.ENSP00000377344,9606.ENSP00000378217,9606.ENSP00000379873,9606.ENSP00000381601,9606.ENSP00000382707,9606.ENSP00000395590,9606.ENSP00000399168,9606.ENSP00000404833,9606.ENSP00000408411	EIF2AK2,SEC61A1,OASL,IL1RN,PKP1,PML,TRIM25,OAS2,SP100,GM2A,STAT1,S100A8,GBP4,IFIT3,ISG15,DDX58,DDX60,PRKCD,HLA-A,MX1,IFITM3,IFI35,HLA-B,TAPBP,LAMP2

GO:0009605	response to external stimulus	29	2152	0.57	1.27E-08	9606.ENSP00000233057,9606.ENSP00000243253,9606.ENSP00000257570,9606.ENSP00000264108,9606.ENSP00000264346,9606.ENSP00000268058,9606.ENSP00000309539,9606.ENSP00000323889,9606.ENSP00000342278,9606.ENSP00000343023,9606.ENSP00000354394,9606.ENSP00000357722,9606.ENSP00000359490,9606.ENSP00000359783,9606.ENSP00000359787,9606.ENSP00000360883,9606.ENSP00000368699,9606.ENSP00000369213,9606.ENSP00000377284,9606.ENSP00000377344,9606.ENSP00000378217,9606.ENSP00000379038,9606.ENSP00000379873,9606.ENSP00000381601,9606.ENSP00000382707,9606.ENSP00000395590,9606.ENSP00000399168,9606.ENSP00000399888,9606.ENSP00000408411	EIF2AK2,SEC61A1,OASL,HAT1,HERC6,PML,DPYSL2,TRIM25,OAS2,SP100,STAT1,S100A8,GBP4,IFI44,IFI44L,IFIT3,ISG15,DDX58,FOLR1,DDX60,PRKCD,TYMP,HLA-A,MX1,IFITM3,IFI35,HLA-B,HMGA1,LAMP2
GO:0043900	regulation of multi-organism process	17	653	0.86	1.83E-08	9606.ENSP00000233057,9606.ENSP00000257570,9606.ENSP00000268058,9606.ENSP00000296577,9606.ENSP00000323889,9606.ENSP00000325618,9606.ENSP00000343023,9606.ENSP00000354394,9606.ENSP00000357722,9606.ENSP00000368699,9606.ENSP00000369213,9606.ENSP00000377344,9606.ENSP00000378217,9606.ENSP00000379873,9606.ENSP00000381601,9606.ENSP00000382707,9606.ENSP00000399168	EIF2AK2,OASL,PML,ABCE1,TRIM25,PARP10,SP100,STAT1,S100A8,ISG15,DDX58,DDX60,PRKCD,HLA-A,MX1,IFITM3,HLA-B
GO:0051704	multi-organism process	31	2514	0.53	1.83E-08	9606.ENSP00000233057,9606.ENSP00000243253,9606.ENSP00000244061,9606.ENSP00000249066,9606.ENSP00000257570,9606.ENSP00000264346,9606.ENSP00000268058,9606.ENSP00000270586,9606.ENSP00000296577,9606.ENSP00000323889,9606.ENSP00000342278,9606.ENSP00000343023,9606.ENSP00000354394,9606.ENSP00000357722,9606.ENSP00000359490,9606.ENSP00000359783,9606.ENSP00000359787,9606.ENSP00000360883,9606.ENSP00000363993,9606.ENSP00000364119,9606.ENSP00000368699,9606.ENSP00000369213,9606.ENSP00000371958,9606.ENSP00000377344,9606.ENSP00000378217,9606.ENSP00000379873,9606.ENSP00000381601,9606.ENSP00000382707,9606.ENSP00000395590,9606.ENSP00000399168,9606.ENSP00000399888	EIF2AK2,SEC61A1,RNF114,APOL2,OASL,HERC6,PML,PSMB6,ABCE1,TRIM25,OAS2,SP100,STAT1,S100A8,GBP4,IFI44,IFI44L,IFIT3,PSMB9,EIF2S2,ISG15,DDX58,CD9,DDX60,PRKCD,HLA-A,MX1,IFITM3,IFI35,HLA-B,HMGA1
GO:0071346	cellular response to interferon-gamma	10	155	1.25	3.90E-08	9606.ENSP00000257570,9606.ENSP00000268058,9606.ENSP00000323889,9606.ENSP00000342278,9606.ENSP00000343023,9606.ENSP00000354394,9606.ENSP00000359490,9606.ENSP00000378217,9606.ENSP00000379873,9606.ENSP00000399168	OASL,PML,TRIM25,OAS2,SP100,STAT1,GBP4,PRKCD,HLA-A,HLA-B
GO:1903901	negative regulation of viral life cycle	8	77	1.46	7.05E-08	9606.ENSP00000233057,9606.ENSP00000257570,9606.ENSP00000268058,9606.ENSP00000323889,9606.ENSP00000325618,9606.ENSP00000368699,9606.ENSP00000381601,9606.ENSP00000382707	EIF2AK2,OASL,PML,TRIM25,PARP10,ISG15,MX1,IFITM3
GO:0002376	immune system process	29	2370	0.53	9.67E-08	9606.ENSP00000233057,9606.ENSP00000243253,9606.ENSP00000257570,9606.ENSP00000259206,9606.ENSP00000263946,9606.ENSP00000264346,9606.ENSP00000268058,9606.ENSP00000323889,9606.ENSP00000342278,9606.ENSP00000343023,9606.ENSP00000349687,9606.ENSP00000354394,9606.ENSP00000357722,9606.ENSP00000359490,9606.ENSP00000359787,9606.ENSP00000360883,9606.ENSP00000363993,9606.ENSP00000368699,9606.ENSP00000369213,9606.ENSP00000372155,9606.ENSP00000377344,9606.ENSP00000378217,9606.ENSP00000379873,9606.ENSP00000381601,9606.ENSP00000382707,9606.ENSP00000395590,9606.ENSP00000399168,9606.ENSP00000404833,9606.ENSP00000408411	EIF2AK2,SEC61A1,OASL,IL1RN,PKP1,HERC6,PML,TRIM25,OAS2,SP100,GM2A,STAT1,S100A8,GBP4,IFI44L,IFIT3,PSMB9,ISG15,DDX58,PSME1,DDX60,PRKCD,HLA-A,MX1,IFITM3,IFI35,HLA-B,TAPBP,LAMP2

GO:0019221	cytokine-mediated signaling pathway	15	655	0.8	1.05E-06	9606.ENSP00000257570,9606.ENSP00000259206,9606.ENSP00000268058,9606.ENSP00000323889,9606.ENSP00000342278,9606.ENSP00000343023,9606.ENSP00000354394,9606.ENSP00000360883,9606.ENSP00000368699,9606.ENSP00000378217,9606.ENSP00000379873,9606.ENSP00000381601,9606.ENSP00000382707,9606.ENSP00000395590,9606.ENSP00000399168	OASL,IL1RN,PML,TRIM25,OAS2,SP100,STAT1,IFIT3,ISG15,PRKCD,HLA-A,MX1,IFITM3,IFI35,HLA-B
GO:0034097	response to cytokine	18	1035	0.68	1.85E-06	9606.ENSP00000233057,9606.ENSP00000243253,9606.ENSP00000257570,9606.ENSP00000259206,9606.ENSP00000268058,9606.ENSP00000323889,9606.ENSP00000342278,9606.ENSP00000343023,9606.ENSP00000354394,9606.ENSP00000359490,9606.ENSP00000360883,9606.ENSP00000368699,9606.ENSP00000378217,9606.ENSP00000379873,9606.ENSP00000381601,9606.ENSP00000382707,9606.ENSP00000395590,9606.ENSP00000399168	EIF2AK2,SEC61A1,OASL,IL1RN,PML,TRIM25,OAS2,SP100,STAT1,GBP4,IFIT3,ISG15,PRKCD,HLA-A,MX1,IFITM3,IFI35,HLA-B
GO:0045071	negative regulation of viral genome replication	6	52	1.5	4.48E-06	9606.ENSP00000233057,9606.ENSP00000257570,9606.ENSP00000325618,9606.ENSP00000368699,9606.ENSP00000381601,9606.ENSP00000382707	EIF2AK2,OASL,PARP10,ISG15,MX1,IFITM3
GO:0016032	viral process	13	571	0.8	1.03E-05	9606.ENSP00000233057,9606.ENSP00000268058,9606.ENSP00000270586,9606.ENSP00000296577,9606.ENSP00000323889,9606.ENSP00000343023,9606.ENSP00000354394,9606.ENSP00000363993,9606.ENSP00000368699,9606.ENSP00000369213,9606.ENSP00000379873,9606.ENSP00000399168,9606.ENSP00000399888	EIF2AK2,PML,PSMB6,ABCE1,TRIM25,SP100,STAT1,PSMB9,ISG15,DDX58,HLA-A,HLA-B,HMGA1
GO:0071345	cellular response to cytokine stimulus	16	953	0.67	1.81E-05	9606.ENSP00000257570,9606.ENSP00000259206,9606.ENSP00000268058,9606.ENSP00000323889,9606.ENSP00000342278,9606.ENSP00000343023,9606.ENSP00000354394,9606.ENSP00000359490,9606.ENSP00000360883,9606.ENSP00000368699,9606.ENSP00000378217,9606.ENSP00000379873,9606.ENSP00000381601,9606.ENSP00000382707,9606.ENSP00000395590,9606.ENSP00000399168	OASL,IL1RN,PML,TRIM25,OAS2,SP100,STAT1,GBP4,IFIT3,ISG15,PRKCD,HLA-A,MX1,IFITM3,IFI35,HLA-B
GO:0006950	response to stress	30	3267	0.4	2.38E-05	9606.ENSP00000216479,9606.ENSP00000233057,9606.ENSP00000243253,9606.ENSP00000249066,9606.ENSP00000257570,9606.ENSP00000259206,9606.ENSP00000268058,9606.ENSP00000287156,9606.ENSP00000323889,9606.ENSP00000333266,9606.ENSP00000342278,9606.ENSP00000343023,9606.ENSP00000354394,9606.ENSP00000357722,9606.ENSP00000359490,9606.ENSP00000359787,9606.ENSP00000360883,9606.ENSP00000368699,9606.ENSP00000369213,9606.ENSP00000371958,9606.ENSP00000377284,9606.ENSP00000377344,9606.ENSP00000378217,9606.ENSP00000379873,9606.ENSP00000381601,9606.ENSP00000382707,9606.ENSP00000395590,9606.ENSP00000399168,9606.ENSP00000399888,9606.ENSP00000408411	AHSA1,EIF2AK2,SEC61A1,APOL2,OASL,IL1RN,PML,UBE2L6,TRIM25,UBA7,OAS2,SP100,STAT1,S100A8,GBP4,IFI44L,IFIT3,ISG15,DDX58,CD9,FOLR1,DDX60,PRKCD,HLA-A,MX1,IFITM3,IFI35,HLA-B,HMGA1,LAMP2
GO:0032480	negative regulation of type I interferon production	5	43	1.51	4.94E-05	9606.ENSP00000287156,9606.ENSP00000323889,9606.ENSP00000333266,9606.ENSP00000368699,9606.ENSP00000369213	UBE2L6,TRIM25,UBA7,ISG15,DDX58
GO:0032020	ISG15-protein conjugation	3	6	2.14	2.10E-04	9606.ENSP00000287156,9606.ENSP00000333266,9606.ENSP00000368699	UBE2L6,UBA7,ISG15
GO:0032479	regulation of type I interferon production	6	112	1.17	2.30E-04	9606.ENSP00000287156,9606.ENSP00000323889,9606.ENSP00000333266,9606.ENSP00000354394,9606.ENSP00000368699,9606.ENSP00000369213	UBE2L6,TRIM25,UBA7,STAT1,ISG15,DDX58
GO:0019885	antigen processing and presentation of endogenous peptide antigen via MHC class I	3	9	1.96	5.20E-04	9606.ENSP00000379873,9606.ENSP00000399168,9606.ENSP00000404833	HLA-A,HLA-B,TAPBP

GO:0001817	regulation of cytokine production	11	615	0.69	7.20E-04	9606.ENSP00000233057,9606.ENSP00000268058,9606.ENSP00000287156,9606.ENSP00000323889,9606.ENSP00000333266,9606.ENSP00000354394,9606.ENSP00000368699,9606.ENSP00000369213,9606.ENSP00000377344,9606.ENSP00000379873,9606.ENSP00000399168	EIF2AK2,PML,UBE2L6,TRIM25,UBA7,STAT1,ISG15,DDX58,DDX60,HLA-A,HLA-B
GO:0002831	regulation of response to biotic stimulus	9	426	0.77	1.30E-03	9606.ENSP00000268058,9606.ENSP00000296577,9606.ENSP00000354394,9606.ENSP00000357722,9606.ENSP00000369213,9606.ENSP00000377344,9606.ENSP00000378217,9606.ENSP00000379873,9606.ENSP00000399168	PML,ABCE1,STAT1,S100A8,DDX58,DDX60,PRKCD,HLA-A,HLA-B
GO:0010033	response to organic substance	24	2815	0.37	1.60E-03	9606.ENSP00000233057,9606.ENSP00000243253,9606.ENSP00000257570,9606.ENSP00000259206,9606.ENSP00000268058,9606.ENSP00000323889,9606.ENSP00000325677,9606.ENSP00000342278,9606.ENSP00000343023,9606.ENSP00000354394,9606.ENSP00000357722,9606.ENSP00000359490,9606.ENSP00000360883,9606.ENSP00000368678,9606.ENSP00000368699,9606.ENSP00000369213,9606.ENSP00000371958,9606.ENSP00000377284,9606.ENSP00000378217,9606.ENSP00000379873,9606.ENSP00000381601,9606.ENSP00000382707,9606.ENSP00000395590,9606.ENSP00000399168	EIF2AK2,SEC61A1,OASL,IL1RN,PML,TRIM25,RNF40,OAS2,SP100,STAT1,S100A8,GBP4,IFIT3,AGRN,ISG15,DDX58,CD9,FOLR1,PRKCD,HLA-A,MX1,IFITM3,IFI35,HLA-B
GO:0044257	cellular protein catabolic process	10	562	0.69	1.70E-03	9606.ENSP00000268058,9606.ENSP00000270586,9606.ENSP00000287156,9606.ENSP00000323889,9606.ENSP00000325677,9606.ENSP00000333266,9606.ENSP00000363993,9606.ENSP00000368699,9606.ENSP00000408411,9606.ENSP00000464087	PML,PSMB6,UBE2L6,TRIM25,RNF40,UBA7,PSMB9,ISG15,LAMP2,RNF213
GO:1901565	organonitrogen compound catabolic process	13	958	0.57	1.80E-03	9606.ENSP00000268058,9606.ENSP00000270586,9606.ENSP00000287156,9606.ENSP00000323889,9606.ENSP00000325677,9606.ENSP00000333266,9606.ENSP00000349687,9606.ENSP00000363993,9606.ENSP00000368678,9606.ENSP00000368699,9606.ENSP00000379038,9606.ENSP00000408411,9606.ENSP00000464087	PML,PSMB6,UBE2L6,TRIM25,RNF40,UBA7,GM2A,PSMB9,AGRN,ISG15,TYMP,LAMP2,RNF213
GO:0009057	macromolecule catabolic process	13	970	0.57	2.00E-03	9606.ENSP00000268058,9606.ENSP00000270586,9606.ENSP00000287156,9606.ENSP00000323889,9606.ENSP00000325677,9606.ENSP00000333266,9606.ENSP00000342278,9606.ENSP00000363993,9606.ENSP00000368678,9606.ENSP00000368699,9606.ENSP00000408411,9606.ENSP00000464087,9606.ENSP00000464342	PML,PSMB6,UBE2L6,TRIM25,RNF40,UBA7,OAS2,PSMB9,AGRN,ISG15,LAMP2,RNF213,RPL36
GO:0044265	cellular macromolecule catabolic process	12	842	0.59	2.20E-03	9606.ENSP00000268058,9606.ENSP00000270586,9606.ENSP00000287156,9606.ENSP00000323889,9606.ENSP00000325677,9606.ENSP00000333266,9606.ENSP00000342278,9606.ENSP00000363993,9606.ENSP00000368699,9606.ENSP00000408411,9606.ENSP00000464087,9606.ENSP00000464342	PML,PSMB6,UBE2L6,TRIM25,RNF40,UBA7,OAS2,PSMB9,ISG15,LAMP2,RNF213,RPL36
GO:0045088	regulation of innate immune response	8	361	0.79	2.30E-03	9606.ENSP00000296577,9606.ENSP00000354394,9606.ENSP00000357722,9606.ENSP00000369213,9606.ENSP00000377344,9606.ENSP00000378217,9606.ENSP00000379873,9606.ENSP00000399168	ABCE1,STAT1,S100A8,DDX58,DDX60,PRKCD,HLA-A,HLA-B

GO:1901564	organonitrogen compound metabolic process	35	5281	0.26	2.30E-03	9606.ENSP00000226299,9606.ENSP00000233057,9606.ENSP00000244061,9606.ENSP00000247665,9606.ENSP00000249066,9606.ENSP00000264108,9606.ENSP00000264346,9606.ENSP00000268058,9606.ENSP00000270586,9606.ENSP00000287156,9606.ENSP00000296577,9606.ENSP00000323889,9606.ENSP00000325618,9606.ENSP00000325677,9606.ENSP00000327436,9606.ENSP00000333266,9606.ENSP00000347495,9606.ENSP00000349687,9606.ENSP00000355330,9606.ENSP00000357722,9606.ENSP00000363993,9606.ENSP00000364119,9606.ENSP00000367923,9606.ENSP00000368678,9606.ENSP00000368699,9606.ENSP00000369213,9606.ENSP00000372155,9606.ENSP00000377284,9606.ENSP00000378217,9606.ENSP00000379038,9606.ENSP00000379873,9606.ENSP00000404833,9606.ENSP00000408411,9606.ENSP00000464087,9606.ENSP00000464342	LAP3,EIF2AK2,RNF114,PHPT1,APOL2,HAT1,HERC6,PML,PSMB6,UBE2L6,ABCE1,TRIM25,PARP10,RNF40,SETD3,UBA7,WARS,GM2A,TGM2,S100A8,PSMB9,EIF2S2,SUCLA2,AGRN,ISG15,DDX58,PSME1,FOLR1,PRKCD,TYMP,HLA-A,TAPBP,LAMP2,RNF213,RPL36
GO:0050896	response to stimulus	45	7824	0.2	2.40E-03	9606.ENSP00000216479,9606.ENSP00000233057,9606.ENSP00000243253,9606.ENSP00000249066,9606.ENSP00000257570,9606.ENSP00000259206,9606.ENSP00000263946,9606.ENSP00000264108,9606.ENSP00000264346,9606.ENSP00000268058,9606.ENSP00000287156,9606.ENSP00000302037,9606.ENSP00000309539,9606.ENSP00000323889,9606.ENSP00000325677,9606.ENSP00000333266,9606.ENSP00000337641,9606.ENSP00000342278,9606.ENSP00000343023,9606.ENSP00000349687,9606.ENSP00000354394,9606.ENSP00000355330,9606.ENSP00000357722,9606.ENSP00000359490,9606.ENSP00000359783,9606.ENSP00000359787,9606.ENSP00000360883,9606.ENSP00000364585,9606.ENSP00000368678,9606.ENSP00000368699,9606.ENSP00000369213,9606.ENSP00000371958,9606.ENSP00000377284,9606.ENSP00000377344,9606.ENSP00000378217,9606.ENSP00000379038,9606.ENSP00000379873,9606.ENSP00000381601,9606.ENSP00000382707,9606.ENSP00000395590,9606.ENSP00000399168,9606.ENSP00000399888,9606.ENSP00000404833,9606.ENSP00000408411,9606.ENSP00000482445	AHSA1,EIF2AK2,SEC61A1,APOL2,OASL,IL1RN,PKP1,HAT1,HERC6,PML,UBE2L6,MFF,DPYSL2,TRIM25,RNF40,UBA7,PPP2R5E,OAS2,SP100,GM2A,STAT1,TGM2,S100A8,GBP4,IFI44,IFI44L,IFIT3,RCC2,AGRN,ISG15,DDX58,CD9,FOLR1,DDX60,PRKCD,TYMP,HLA-A,MX1,IFITM3,IFI35,HLA-B,HMGA1,TAPBP,LAMP2,FAM213A
GO:0044267	cellular protein metabolic process	27	3603	0.31	3.40E-03	9606.ENSP00000233057,9606.ENSP00000244061,9606.ENSP00000247665,9606.ENSP00000264108,9606.ENSP00000264346,9606.ENSP00000268058,9606.ENSP00000270586,9606.ENSP00000287156,9606.ENSP00000296577,9606.ENSP00000323889,9606.ENSP00000325618,9606.ENSP00000325677,9606.ENSP00000327436,9606.ENSP00000333266,9606.ENSP00000347495,9606.ENSP00000355330,9606.ENSP00000357722,9606.ENSP00000363993,9606.ENSP00000364119,9606.ENSP00000368699,9606.ENSP00000369213,9606.ENSP00000372155,9606.ENSP00000378217,9606.ENSP00000379873,9606.ENSP00000408411,9606.ENSP00000464087,9606.ENSP00000464342	EIF2AK2,RNF114,PHPT1,HAT1,HERC6,PML,PSMB6,UBE2L6,ABCE1,TRIM25,PARP10,RNF40,SETD3,UBA7,WARS,TGM2,S100A8,PSMB9,EIF2S2,ISG15,DDX58,PSME1,PRKCD,HLA-A,LAMP2,RNF213,RPL36
GO:0071310	cellular response to organic substance	20	2219	0.4	3.40E-03	9606.ENSP00000233057,9606.ENSP00000257570,9606.ENSP00000259206,9606.ENSP00000268058,9606.ENSP00000323889,9606.ENSP00000342278,9606.ENSP00000343023,9606.ENSP00000354394,9606.ENSP00000359490,9606.ENSP00000360883,9606.ENSP00000368678,9606.ENSP00000368699,9606.ENSP00000369213,9606.ENSP00000377284,9606.ENSP00000378217,9606.ENSP00000379873,9606.ENSP00000381601,9606.ENSP00000382707,9606.ENSP00000395590,9606.ENSP00000399168	EIF2AK2,OASL,IL1RN,PML,TRIM25,OAS2,SP100,STAT1,GBP4,IFIT3,AGRN,ISG15,DDX58,FOLR1,PRKCD,HLA-A,MX1,IFITM3,IFI35,HLA-B

GO:0035455	response to interferon-alpha	3	22	1.57	3.70E-03	9606.ENSP00000233057,9606.ENSP00000360883,9606.ENSP00000382707	EIF2AK2,IFIT3,IFITM3
GO:0002486	antigen processing and presentation of endogenous peptide antigen via MHC class I via ER pathway, TAP-independent	2	3	2.26	4.70E-03	9606.ENSP00000379873,9606.ENSP00000399168	HLA-A,HLA-B
GO:0002479	antigen processing and presentation of exogenous peptide antigen via MHC class I, TAP-dependent	3	25	1.52	4.90E-03	9606.ENSP00000379873,9606.ENSP00000399168,9606.ENSP00000404833	HLA-A,HLA-B,TAPBP
GO:0051603	proteolysis involved in cellular protein catabolic process	9	532	0.67	4.90E-03	9606.ENSP00000268058,9606.ENSP00000270586,9606.ENSP00000287156,9606.ENSP00000323889,9606.ENSP00000325677,9606.ENSP00000333266,9606.ENSP00000363993,9606.ENSP00000368699,9606.ENSP00000464087	PML,PSMB6,UBE2L6,TRIM25,RNF40,UBA7,PSMB9,ISG15,RNF213
GO:0070887	cellular response to chemical stimulus	22	2672	0.36	4.90E-03	9606.ENSP00000233057,9606.ENSP00000257570,9606.ENSP00000259206,9606.ENSP00000268058,9606.ENSP00000323889,9606.ENSP00000342278,9606.ENSP00000343023,9606.ENSP00000354394,9606.ENSP00000357722,9606.ENSP00000359490,9606.ENSP00000360883,9606.ENSP00000368678,9606.ENSP00000368699,9606.ENSP00000369213,9606.ENSP00000377284,9606.ENSP00000378217,9606.ENSP00000379873,9606.ENSP00000381601,9606.ENSP00000382707,9606.ENSP00000395590,9606.ENSP00000399168,9606.ENSP00000482445	EIF2AK2,OASL,IL1RN,PML,TRIM25,OAS2,SP100,STAT1,S100A8,GBP4,IFIT3,AGRN,ISG15,DDX58,FOLR1,PRKCD,HLA-A,MX1,IFITM3,IFI35,HLA-B,FAM213A
GO:0070647	protein modification by small protein conjugation or removal	12	945	0.54	5.10E-03	9606.ENSP00000244061,9606.ENSP00000264346,9606.ENSP00000270586,9606.ENSP00000287156,9606.ENSP00000323889,9606.ENSP00000325677,9606.ENSP00000333266,9606.ENSP00000363993,9606.ENSP00000369213,9606.ENSP00000372155,9606.ENSP00000379873,9606.ENSP00000464087	RNF114,HERC6,PSMB6,UBE2L6,TRIM25,RNF40,UBA7,PSMB9,DDX58,PSME1,HLA-A,RNF213
GO:0002230	positive regulation of defense response to virus by host	3	26	1.5	5.20E-03	9606.ENSP00000268058,9606.ENSP00000354394,9606.ENSP00000369213	PML,STAT1,DDX58
GO:0050688	regulation of defense response to virus	4	71	1.19	5.20E-03	9606.ENSP00000268058,9606.ENSP00000354394,9606.ENSP00000369213,9606.ENSP00000377344	PML,STAT1,DDX58,DDX60
GO:0031347	regulation of defense response	10	676	0.61	5.50E-03	9606.ENSP00000268058,9606.ENSP00000296577,9606.ENSP00000354394,9606.ENSP00000355330,9606.ENSP00000357722,9606.ENSP00000369213,9606.ENSP00000377344,9606.ENSP00000378217,9606.ENSP00000379873,9606.ENSP00000399168	PML,ABCE1,STAT1,TGM2,S100A8,DDX58,DDX60,PRKCD,HLA-A,HLA-B
GO:0019538	protein metabolic process	29	4194	0.28	5.70E-03	9606.ENSP00000226299,9606.ENSP00000233057,9606.ENSP00000244061,9606.ENSP00000247665,9606.ENSP00000249066,9606.ENSP00000264108,9606.ENSP00000264346,9606.ENSP00000268058,9606.ENSP00000270586,9606.ENSP00000287156,9606.ENSP00000296577,9606.ENSP00000323889,9606.ENSP00000325618,9606.ENSP00000325677,9606.ENSP00000327436,9606.ENSP00000333266,9606.ENSP00000347495,9606.ENSP00000355330,9606.ENSP00000357722,9606.ENSP00000363993,9606.ENSP00000364119,9606.ENSP00000368699,9606.ENSP00000369213,9606.ENSP00000372155,9606.ENSP00000378217,9606.ENSP00000379873,9606.ENSP00000408411,9606.ENSP00000464087,9606.ENSP00000464342	LAP3,EIF2AK2,RNF114,PHPT1,APOL2,HAT1,HERC6,PML,PSMB6,UBE2L6,ABCE1,TRIM25,PARP10,RNF40,SETD3,UBA7,WARS,TGM2,S100A8,PSMB9,EIF2S2,ISG15,DDX58,PSME1,PRKCD,HLA-A,LAMP2,RNF213,RPL36

GO:0080134	regulation of response to stress	14	1299	0.47	6.80E-03	9606.ENSP00000233057,9606.ENSP00000261517,9606.ENSP00000268058,9606.ENSP00000296577,9606.ENSP00000354394,9606.ENSP00000355330,9606.ENSP00000357722,9606.ENSP00000369213,9606.ENSP00000371958,9606.ENSP00000377344,9606.ENSP00000378217,9606.ENSP00000379873,9606.ENSP00000399168,9606.ENSP00000399888	EIF2AK2,VPS13C,PML,ABCE1,STAT1,TGM2,S100A8,DDX58,CD9,DDX60,PRKCD,HLA-A,HLA-B,HMGA1
GO:0044248	cellular catabolic process	16	1646	0.43	7.10E-03	9606.ENSP00000268058,9606.ENSP00000270586,9606.ENSP00000279281,9606.ENSP00000287156,9606.ENSP00000323889,9606.ENSP00000325677,9606.ENSP00000333266,9606.ENSP00000342278,9606.ENSP00000349687,9606.ENSP00000357722,9606.ENSP00000363993,9606.ENSP00000368699,9606.ENSP00000379038,9606.ENSP00000408411,9606.ENSP00000464087,9606.ENSP00000464342	PML,PSMB6,VPS51,UBE2L6,TRIM25,RNF40,UBA7,OAS2,GM2A,S100A8,PSMB9,ISG15,TYMP,LAMP2,RNF213,RPL36
GO:0048519	negative regulation of biological process	32	4953	0.25	7.40E-03	9606.ENSP00000233057,9606.ENSP00000247665,9606.ENSP00000257570,9606.ENSP00000259206,9606.ENSP00000261517,9606.ENSP00000263946,9606.ENSP00000264108,9606.ENSP00000268058,9606.ENSP00000287156,9606.ENSP00000296577,9606.ENSP00000305699,9606.ENSP00000323889,9606.ENSP00000325618,9606.ENSP00000325677,9606.ENSP00000333266,9606.ENSP00000343023,9606.ENSP00000347495,9606.ENSP00000354394,9606.ENSP00000360883,9606.ENSP00000364585,9606.ENSP00000368699,9606.ENSP00000369213,9606.ENSP00000371958,9606.ENSP00000378217,9606.ENSP00000379873,9606.ENSP00000381601,9606.ENSP00000382707,9606.ENSP00000399168,9606.ENSP00000399888,9606.ENSP00000408411,9606.ENSP00000464087,9606.ENSP00000464342	EIF2AK2,PHPT1,OASL,IL1RN,VPS13C,PKP1,HAT1,PML,UBE2L6,ABCE1,HOOK3,TRIM25,PARP10,RNF40,UBA7,SP100,WARS,STAT1,IFIT3,RCC2,ISG15,DDX58,CD9,PRKCD,HLA-A,MX1,IFITM3,HLA-B,HMGA1,LAMP2,RNF213,RPL36
GO:0051239	regulation of multicellular organismal process	22	2788	0.34	7.50E-03	9606.ENSP00000233057,9606.ENSP00000259206,9606.ENSP00000268058,9606.ENSP00000287156,9606.ENSP00000305699,9606.ENSP00000309539,9606.ENSP00000323889,9606.ENSP00000333266,9606.ENSP00000342278,9606.ENSP00000343023,9606.ENSP00000347495,9606.ENSP00000354394,9606.ENSP00000368678,9606.ENSP00000368699,9606.ENSP00000369213,9606.ENSP00000371958,9606.ENSP00000377344,9606.ENSP00000378217,9606.ENSP00000379038,9606.ENSP00000379873,9606.ENSP00000399168,9606.ENSP00000482445	EIF2AK2,IL1RN,PML,UBE2L6,HOOK3,DPYSL2,TRIM25,UBA7,OAS2,SP100,WARS,STAT1,AGRN,ISG15,DDX58,CD9,DDX60,PRKCD,TYMP,HLA-A,HLA-B,FAM213A
GO:0042270	protection from natural killer cell mediated cytotoxicity	2	5	2.04	7.80E-03	9606.ENSP00000379873,9606.ENSP00000399168	HLA-A,HLA-B
GO:0090398	cellular senescence	3	32	1.41	7.80E-03	9606.ENSP00000268058,9606.ENSP00000378217,9606.ENSP00000399888	PML,PRKCD,HMGA1
GO:0001818	negative regulation of cytokine production	6	245	0.83	8.30E-03	9606.ENSP00000268058,9606.ENSP00000287156,9606.ENSP00000323889,9606.ENSP00000333266,9606.ENSP00000368699,9606.ENSP00000369213	PML,UBE2L6,TRIM25,UBA7,ISG15,DDX58
GO:0009056	catabolic process	17	1859	0.4	8.30E-03	9606.ENSP00000268058,9606.ENSP00000270586,9606.ENSP00000279281,9606.ENSP00000287156,9606.ENSP00000323889,9606.ENSP00000325677,9606.ENSP00000333266,9606.ENSP00000342278,9606.ENSP00000349687,9606.ENSP00000357722,9606.ENSP00000363993,9606.ENSP00000368678,9606.ENSP00000368699,9606.ENSP00000379038,9606.ENSP00000408411,9606.ENSP00000464087,9606.ENSP00000464342	PML,PSMB6,VPS51,UBE2L6,TRIM25,RNF40,UBA7,OAS2,GM2A,S100A8,PSMB9,AGRN,ISG15,TYMP,LAMP2,RNF213,RPL36

GO:0043603	cellular amide metabolic process	10	732	0.58	8.80E-03	9606.ENSP00000233057,9606.ENSP00000296577,9606.ENSP00000347495,9606.ENSP00000349687,9606.ENSP00000364119,9606.ENSP00000367923,9606.ENSP00000377284,9606.ENSP00000404833,9606.ENSP00000408411,9606.ENSP00000464342	EIF2AK2,ABCE1,WARS,GM2A,EIF2S2,SUCLA2,FOLR1,TAPBP,LAMP2,RPL36
GO:0016192	vesicle-mediated transport	16	1699	0.41	9.10E-03	9606.ENSP00000261517,9606.ENSP00000263946,9606.ENSP00000279281,9606.ENSP00000305699,9606.ENSP00000309539,9606.ENSP00000349687,9606.ENSP00000355330,9606.ENSP00000357722,9606.ENSP00000371958,9606.ENSP00000377284,9606.ENSP00000377566,9606.ENSP00000378217,9606.ENSP00000379873,9606.ENSP00000399168,9606.ENSP00000404833,9606.ENSP00000408411	VPS13C,PKP1,VPS51,HOOK3,DPYSL2,GM2A,TGM2,S100A8,CD9,FOLR1,ELMO3,PRKCD,HLA-A,HLA-B,TAPBP,LAMP2
GO:1902275	regulation of chromatin organization	5	162	0.93	9.30E-03	9606.ENSP00000268058,9606.ENSP00000325618,9606.ENSP00000325677,9606.ENSP00000380557,9606.ENSP00000399888	PML,PARP10,RNF40,AKAP8L,HMGA1
GO:0042221	response to chemical	28	4153	0.27	1.02E-02	9606.ENSP00000233057,9606.ENSP00000243253,9606.ENSP00000257570,9606.ENSP00000259206,9606.ENSP00000264108,9606.ENSP00000268058,9606.ENSP00000309539,9606.ENSP00000323889,9606.ENSP00000325677,9606.ENSP00000342278,9606.ENSP00000343023,9606.ENSP00000354394,9606.ENSP00000357722,9606.ENSP00000359490,9606.ENSP00000360883,9606.ENSP00000368678,9606.ENSP00000368699,9606.ENSP00000369213,9606.ENSP00000371958,9606.ENSP00000377284,9606.ENSP00000378217,9606.ENSP00000379038,9606.ENSP00000379873,9606.ENSP00000381601,9606.ENSP00000382707,9606.ENSP00000395590,9606.ENSP00000399168,9606.ENSP00000482445	EIF2AK2,SEC61A1,OASL,IL1RN,HAT1,PML,DPYSL2,TRIM25,RNF40,OAS2,SP100,STAT1,S100A8,GBP4,IFIT3,AGRN,ISG15,DDX58,CD9,FOLR1,PRKCD,TYMP,HLA-A,MX1,IFITM3,IFI35,HLA-B,FAM213A
GO:0019985	translesion synthesis	3	41	1.3	1.39E-02	9606.ENSP00000287156,9606.ENSP00000323889,9606.ENSP00000368699	UBE2L6,TRIM25,ISG15
GO:0002480	antigen processing and presentation of exogenous peptide antigen via MHC class I, TAP-independent	2	8	1.84	1.45E-02	9606.ENSP00000379873,9606.ENSP00000399168	HLA-A,HLA-B
GO:1901575	organic substance catabolic process	15	1609	0.41	1.49E-02	9606.ENSP00000268058,9606.ENSP00000270586,9606.ENSP00000287156,9606.ENSP00000323889,9606.ENSP00000325677,9606.ENSP00000333266,9606.ENSP00000342278,9606.ENSP00000349687,9606.ENSP00000363993,9606.ENSP00000368678,9606.ENSP00000368699,9606.ENSP00000379038,9606.ENSP00000408411,9606.ENSP00000464087,9606.ENSP00000464342	PML,PSMB6,UBE2L6,TRIM25,RNF40,UBA7,OAS2,GM2A,PSMB9,AGRN,ISG15,TYMP,LAMP2,RNF213,RPL36
GO:0032101	regulation of response to external stimulus	11	955	0.5	1.62E-02	9606.ENSP00000268058,9606.ENSP00000296577,9606.ENSP00000354394,9606.ENSP00000355330,9606.ENSP00000357722,9606.ENSP00000369213,9606.ENSP00000371958,9606.ENSP00000377344,9606.ENSP00000378217,9606.ENSP00000379873,9606.ENSP00000399168	PML,ABCE1,STAT1,TGM2,S100A8,DDX58,CD9,DDX60,PRKCD,HLA-A,HLA-B
GO:0006464	cellular protein modification process	22	2999	0.31	1.72E-02	9606.ENSP00000233057,9606.ENSP00000244061,9606.ENSP00000247665,9606.ENSP00000264108,9606.ENSP00000264346,9606.ENSP00000268058,9606.ENSP00000270586,9606.ENSP00000287156,9606.ENSP00000323889,9606.ENSP00000325618,9606.ENSP00000325677,9606.ENSP00000327436,9606.ENSP00000333266,9606.ENSP00000355330,9606.ENSP00000357722,9606.ENSP00000363993,9606.ENSP00000368699,9606.ENSP00000369213,9606.ENSP00000372155,9606.ENSP00000378217,9606.ENSP00000379873,9606.ENSP00000464087	EIF2AK2,RNF114,PHPT1,HAT1,HERC6,PML,PSMB6,UBE2L6,TRIM25,PARP10,RNF40,SETD3,UBA7,TGM2,S100A8,PSMB9,ISG15,DDX58,PSME1,PRKCD,HLA-A,RNF213

GO:0009617	response to bacterium	8	555	0.6	2.22E-02	9606.ENSP00000264346,9606.ENSP00000342278,9606.ENSP00000357722,9606.ENSP00000359783,9606.ENSP00000368699,9606.ENSP00000378217,9606.ENSP00000379873,9606.ENSP00000399168	HERC6,OAS2,S100A8,IFI44,ISG15,PRKCD,HLA-A,HLA-B
GO:0090331	negative regulation of platelet aggregation	2	11	1.7	2.28E-02	9606.ENSP00000371958,9606.ENSP00000378217	CD9,PRKCD
GO:0008152	metabolic process	48	9569	0.14	2.60E-02	9606.ENSP00000226299,9606.ENSP00000233057,9606.ENSP00000244061,9606.ENSP00000247665,9606.ENSP00000249066,9606.ENSP00000259206,9606.ENSP00000264108,9606.ENSP00000264346,9606.ENSP00000268058,9606.ENSP00000270586,9606.ENSP00000279281,9606.ENSP00000287156,9606.ENSP00000296577,9606.ENSP00000309539,9606.ENSP00000313432,9606.ENSP00000323889,9606.ENSP00000325618,9606.ENSP00000325677,9606.ENSP00000327436,9606.ENSP00000333266,9606.ENSP00000342278,9606.ENSP00000343023,9606.ENSP00000347495,9606.ENSP00000349687,9606.ENSP00000354394,9606.ENSP00000355330,9606.ENSP00000357722,9606.ENSP00000363993,9606.ENSP00000364119,9606.ENSP00000367923,9606.ENSP00000368678,9606.ENSP00000368699,9606.ENSP00000369213,9606.ENSP00000371958,9606.ENSP00000372155,9606.ENSP00000377284,9606.ENSP00000378217,9606.ENSP00000379038,9606.ENSP00000379873,9606.ENSP00000380557,9606.ENSP00000380837,9606.ENSP00000399888,9606.ENSP00000400591,9606.ENSP00000404833,9606.ENSP00000408411,9606.ENSP00000464087,9606.ENSP00000464342,9606.ENSP00000482445	LAP3,EIF2AK2,RNF114,PHPT1,APOL2,IL1RN,HAT1,HERC6,PML,PSMB6,VPS51,UBE2L6,ABCE1,DPYSL2,GRHPR,TRIM25,PARP10,RNF40,SETD3,UBA7,OAS2,SP100,WARS,GM2A,STAT1,TGM2,S100A8,PSMB9,EIF2S2,SUCLA2,AGRN,ISG15,DDX58,CD9,PSME1,FOLR1,PRKCD,TYMP,HLA-A,AKAP8L,LSS,HMGA1,SNRPE,TAPBP,LAMP2,RNF213,RPL36,FAM213A
GO:0006508	proteolysis	12	1203	0.44	2.88E-02	9606.ENSP00000226299,9606.ENSP00000268058,9606.ENSP00000270586,9606.ENSP00000287156,9606.ENSP00000323889,9606.ENSP00000325677,9606.ENSP00000333266,9606.ENSP00000363993,9606.ENSP00000368699,9606.ENSP00000369213,9606.ENSP00000372155,9606.ENSP00000464087	LAP3,PML,PSMB6,UBE2L6,TRIM25,RNF40,UBA7,PSMB9,ISG15,DDX58,PSME1,RNF213
GO:0051234	establishment of localization	27	4248	0.24	2.95E-02	9606.ENSP00000243253,9606.ENSP00000249066,9606.ENSP00000259206,9606.ENSP00000261517,9606.ENSP00000263946,9606.ENSP00000268058,9606.ENSP00000279281,9606.ENSP00000296577,9606.ENSP00000302037,9606.ENSP00000305699,9606.ENSP00000309539,9606.ENSP00000349687,9606.ENSP00000355330,9606.ENSP00000357722,9606.ENSP00000364119,9606.ENSP00000364585,9606.ENSP00000371958,9606.ENSP00000377284,9606.ENSP00000377566,9606.ENSP00000378217,9606.ENSP00000379873,9606.ENSP00000399168,9606.ENSP00000399888,9606.ENSP00000400591,9606.ENSP00000404833,9606.ENSP00000408411,9606.ENSP00000464342	SEC61A1,APOL2,IL1RN,VPS13C,PKP1,PML,VPS51,ABCE1,MFF,HOOK3,DPYSL2,GM2A,TGM2,S100A8,EIF2S2,RCC2,CD9,FOLR1,ELMO3,PRKCD,HLA-A,HLA-B,HMGA1,SNRPE,TAPBP,LAMP2,RPL36
GO:0002682	regulation of immune system process	13	1391	0.41	3.08E-02	9606.ENSP00000233057,9606.ENSP00000247665,9606.ENSP00000268058,9606.ENSP00000296577,9606.ENSP00000354394,9606.ENSP00000357722,9606.ENSP00000368699,9606.ENSP00000369213,9606.ENSP00000377344,9606.ENSP00000378217,9606.ENSP00000379873,9606.ENSP00000399168,9606.ENSP00000482445	EIF2AK2,PHPT1,PML,ABCE1,STAT1,S100A8,ISG15,DDX58,DDX60,PRKCD,HLA-A,HLA-B,FAM213A
GO:0019884	antigen processing and presentation of exogenous antigen	4	130	0.93	3.08E-02	9606.ENSP00000372155,9606.ENSP00000379873,9606.ENSP00000399168,9606.ENSP00000404833	PSME1,HLA-A,HLA-B,TAPBP

GO:0019941	modification-dependent protein catabolic process	7	467	0.62	3.35E-02	9606.ENSP00000268058,9606.ENSP00000287156,9606.ENSP00000323889,9606.ENSP00000325677,9606.ENSP00000333266,9606.ENSP00000368699,9606.ENSP00000464087	PML,UBE2L6,TRIM25,RNF40,UBA7,ISG15,RNF213
GO:0051336	regulation of hydrolase activity	12	1238	0.43	3.37E-02	9606.ENSP00000216479,9606.ENSP00000233057,9606.ENSP00000268058,9606.ENSP00000296577,9606.ENSP00000337641,9606.ENSP00000349687,9606.ENSP00000357722,9606.ENSP00000363993,9606.ENSP00000364585,9606.ENSP00000368678,9606.ENSP00000372155,9606.ENSP00000378217	AHSA1,EIF2AK2,PML,ABCE1,PPP2R5E,GM2A,S100A8,PSMB9,RCC2,AGRN,PSME1,PRKCD
GO:0071704	organic substance metabolic process	46	9135	0.14	3.37E-02	9606.ENSP00000226299,9606.ENSP00000233057,9606.ENSP00000244061,9606.ENSP00000247665,9606.ENSP00000249066,9606.ENSP00000259206,9606.ENSP00000264108,9606.ENSP00000264346,9606.ENSP00000268058,9606.ENSP00000270586,9606.ENSP00000287156,9606.ENSP00000296577,9606.ENSP00000309539,9606.ENSP00000313432,9606.ENSP00000323889,9606.ENSP00000325618,9606.ENSP00000325677,9606.ENSP00000327436,9606.ENSP00000333266,9606.ENSP00000342278,9606.ENSP00000343023,9606.ENSP00000347495,9606.ENSP00000349687,9606.ENSP00000354394,9606.ENSP00000355330,9606.ENSP00000357722,9606.ENSP00000363993,9606.ENSP00000364119,9606.ENSP00000367923,9606.ENSP00000368678,9606.ENSP00000368699,9606.ENSP00000369213,9606.ENSP00000371958,9606.ENSP00000372155,9606.ENSP00000377284,9606.ENSP00000378217,9606.ENSP00000379038,9606.ENSP00000379873,9606.ENSP00000380557,9606.ENSP00000380837,9606.ENSP00000399888,9606.ENSP00000400591,9606.ENSP00000404833,9606.ENSP00000408411,9606.ENSP00000464087,9606.ENSP00000464342	LAP3,EIF2AK2,RNF114,PHPT1,APOL2,IL1RN,HAT1,HERC6,PML,PSMB6,UBE2L6,ABCE1,DPYSL2,GRHPR,TRIM25,PARP10,RNF40,SETD3,UBA7,OAS2,SP100,WARS,GM2A,STAT1,TGM2,S100A8,PSMB9,EIF2S2,SUCLA2,AGRN,ISG15,DDX58,CD9,PSME1,FOLR1,PRKCD,TYMP,HLA-A,AKAP8L,LSS,HMGA1,SNRPE,TAPBP,LAMP2,RNF213,RPL36
GO:0016045	detection of bacterium	2	15	1.57	3.40E-02	9606.ENSP00000379873,9606.ENSP00000399168	HLA-A,HLA-B
GO:0006807	nitrogen compound metabolic process	43	8349	0.15	3.62E-02	9606.ENSP00000226299,9606.ENSP00000233057,9606.ENSP00000244061,9606.ENSP00000247665,9606.ENSP00000249066,9606.ENSP00000264108,9606.ENSP00000264346,9606.ENSP00000268058,9606.ENSP00000270586,9606.ENSP00000287156,9606.ENSP00000296577,9606.ENSP00000309539,9606.ENSP00000313432,9606.ENSP00000323889,9606.ENSP00000325618,9606.ENSP00000325677,9606.ENSP00000327436,9606.ENSP00000333266,9606.ENSP00000342278,9606.ENSP00000343023,9606.ENSP00000347495,9606.ENSP00000349687,9606.ENSP00000354394,9606.ENSP00000355330,9606.ENSP00000357722,9606.ENSP00000363993,9606.ENSP00000364119,9606.ENSP00000367923,9606.ENSP00000368678,9606.ENSP00000368699,9606.ENSP00000369213,9606.ENSP00000372155,9606.ENSP00000377284,9606.ENSP00000378217,9606.ENSP00000379038,9606.ENSP00000379873,9606.ENSP00000380557,9606.ENSP00000399888,9606.ENSP00000400591,9606.ENSP00000404833,9606.ENSP00000408411,9606.ENSP00000464087,9606.ENSP00000464342	LAP3,EIF2AK2,RNF114,PHPT1,APOL2,HAT1,HERC6,PML,PSMB6,UBE2L6,ABCE1,DPYSL2,GRHPR,TRIM25,PARP10,RNF40,SETD3,UBA7,OAS2,SP100,WARS,GM2A,STAT1,TGM2,S100A8,PSMB9,EIF2S2,SUCLA2,AGRN,ISG15,DDX58,PSME1,FOLR1,PRKCD,TYMP,HLA-A,AKAP8L,HMGA1,SNRPE,TAPBP,LAMP2,RNF213,RPL36
GO:0007166	cell surface receptor signaling pathway	17	2198	0.33	3.75E-02	9606.ENSP00000257570,9606.ENSP00000259206,9606.ENSP00000268058,9606.ENSP00000323889,9606.ENSP00000342278,9606.ENSP00000343023,9606.ENSP00000354394,9606.ENSP00000360883,9606.ENSP00000364585,9606.ENSP00000368699,9606.ENSP00000371958,9606.ENSP00000378217,9606.ENSP00000379873,9606.ENSP00000381601,9606.ENSP00000382707,9606.ENSP00000395590,9606.ENSP00000399168	OASL,IL1RN,PML,TRIM25,OAS2,SP100,STAT1,IFIT3,RCC2,ISG15,CD9,PRKCD,HLA-A,MX1,IFITM3,IFI35,HLA-B

GO:0006810	transport	26	4130	0.24	3.79E-02	9606.ENSP00000243253,9606.ENSP00000249066,9606.ENSP00000259206,9606.ENSP00000261517,9606.ENSP00000263946,9606.ENSP00000268058,9606.ENSP00000279281,9606.ENSP00000296577,9606.ENSP00000302037,9606.ENSP00000305699,9606.ENSP00000309539,9606.ENSP00000349687,9606.ENSP00000355330,9606.ENSP00000357722,9606.ENSP00000364119,9606.ENSP00000371958,9606.ENSP00000377284,9606.ENSP00000377566,9606.ENSP00000378217,9606.ENSP00000379873,9606.ENSP00000399168,9606.ENSP00000399888,9606.ENSP00000400591,9606.ENSP00000404833,9606.ENSP00000408411,9606.ENSP00000464342	SEC61A1,APOL2,IL1RN,VPS13C,PKP1,PML,VPS51,ABCE1,MFF,HOOK3,DPYSL2,GM2A,TGM2,S100A8,EIF2S2,CD9,FOLR1,ELMO3,PRKCD,HLA-A,HLA-B,HMGA1,SNRPE,TAPBP,LAMP2,RPL36
GO:0043312	neutrophil degranulation	7	485	0.6	3.79E-02	9606.ENSP00000263946,9606.ENSP00000349687,9606.ENSP00000357722,9606.ENSP00000378217,9606.ENSP00000379873,9606.ENSP00000399168,9606.ENSP00000408411	PKP1,GM2A,S100A8,PRKCD,HLA-A,HLA-B,LAMP2
GO:0051241	negative regulation of multicellular organismal process	11	1098	0.44	3.79E-02	9606.ENSP00000268058,9606.ENSP00000287156,9606.ENSP00000305699,9606.ENSP00000323889,9606.ENSP00000333266,9606.ENSP00000343023,9606.ENSP00000354394,9606.ENSP00000368699,9606.ENSP00000369213,9606.ENSP00000371958,9606.ENSP00000378217	PML,UBE2L6,HOOK3,TRIM25,UBA7,SP100,STAT1,ISG15,DDX58,CD9,PRKCD
GO:0001916	positive regulation of T cell mediated cytotoxicity	2	17	1.51	3.86E-02	9606.ENSP00000379873,9606.ENSP00000399168	HLA-A,HLA-B
GO:0002697	regulation of immune effector process	6	362	0.66	3.86E-02	9606.ENSP00000268058,9606.ENSP00000354394,9606.ENSP00000369213,9606.ENSP00000377344,9606.ENSP00000379873,9606.ENSP00000399168	PML,STAT1,DDX58,DDX60,HLA-A,HLA-B
GO:0008631	intrinsic apoptotic signaling pathway in response to oxidative stress	2	17	1.51	3.86E-02	9606.ENSP00000268058,9606.ENSP00000378217	PML,PRKCD
GO:0048384	retinoic acid receptor signaling pathway	2	17	1.51	3.86E-02	9606.ENSP00000268058,9606.ENSP00000343023	PML,SP100
GO:0050793	regulation of developmental process	18	2416	0.31	3.86E-02	9606.ENSP00000233057,9606.ENSP00000259206,9606.ENSP00000268058,9606.ENSP00000302037,9606.ENSP00000305699,9606.ENSP00000309539,9606.ENSP00000327436,9606.ENSP00000342278,9606.ENSP00000343023,9606.ENSP00000347495,9606.ENSP00000354394,9606.ENSP00000364585,9606.ENSP00000368678,9606.ENSP00000368699,9606.ENSP00000379038,9606.ENSP00000399168,9606.ENSP00000399888,9606.ENSP00000482445	EIF2AK2,IL1RN,PML,MFF,HOOK3,DPYSL2,SETD3,OAS2,SP100,WARS,STAT1,RCC2,AGRN,ISG15,TYMP,HLA-B,HMGA1,FAM213A
GO:1902187	negative regulation of viral release from host cell	2	17	1.51	3.86E-02	9606.ENSP00000268058,9606.ENSP00000323889	PML,TRIM25
GO:0006518	peptide metabolic process	7	497	0.59	3.88E-02	9606.ENSP00000233057,9606.ENSP00000296577,9606.ENSP00000347495,9606.ENSP00000364119,9606.ENSP00000404833,9606.ENSP00000408411,9606.ENSP00000464342	EIF2AK2,ABCE1,WARS,EIF2S2,TAPBP,LAMP2,RPL36
GO:0016567	protein ubiquitination	8	645	0.53	3.88E-02	9606.ENSP00000244061,9606.ENSP00000264346,9606.ENSP00000287156,9606.ENSP00000323889,9606.ENSP00000325677,9606.ENSP00000333266,9606.ENSP00000379873,9606.ENSP00000464087	RNF114,HERC6,UBE2L6,TRIM25,RNF40,UBA7,HLA-A,RNF213
GO:0043555	regulation of translation in response to stress	2	18	1.49	3.88E-02	9606.ENSP00000233057,9606.ENSP00000268058	EIF2AK2,PML
GO:0046597	negative regulation of viral entry into host cell	2	18	1.49	3.88E-02	9606.ENSP00000323889,9606.ENSP00000382707	TRIM25,IFITM3
GO:0051091	positive regulation of DNA-binding transcription factor activity	5	250	0.74	3.88E-02	9606.ENSP00000233057,9606.ENSP00000323889,9606.ENSP00000343023,9606.ENSP00000357722,9606.ENSP00000369213	EIF2AK2,TRIM25,SP100,S100A8,DDX58

GO:0051235	maintenance of location	4	150	0.87	3.88E-02	9606.ENSP00000268058,9606.ENSP00000343023,9606.ENSP00000349687,9606.ENSP00000357722	PML,SP100,GM2A,S100A8
GO:0033365	protein localization to organelle	8	649	0.53	3.95E-02	9606.ENSP00000243253,9606.ENSP00000268058,9606.ENSP00000302037,9606.ENSP00000305699,9606.ENSP00000343023,9606.ENSP00000364585,9606.ENSP00000408411,9606.ENSP00000464342	SEC61A1,PML,MFF,HOOK3,SP100,RCC2,LAMP2,RPL36
GO:0000266	mitochondrial fission	2	19	1.46	4.03E-02	9606.ENSP00000302037,9606.ENSP00000381601	MFF,MX1
GO:0002526	acute inflammatory response	3	73	1.05	4.03E-02	9606.ENSP00000249066,9606.ENSP00000259206,9606.ENSP00000357722	APOL2,IL1RN,S100A8
GO:0031065	positive regulation of histone deacetylation	2	19	1.46	4.03E-02	9606.ENSP00000268058,9606.ENSP00000380557	PML,AKAP8L
GO:0090087	regulation of peptide transport	8	654	0.53	4.03E-02	9606.ENSP00000247665,9606.ENSP00000268058,9606.ENSP00000302037,9606.ENSP00000343023,9606.ENSP00000357722,9606.ENSP00000369213,9606.ENSP00000378217,9606.ENSP00000380557	PHPT1,PML,MFF,SP100,S100A8,DDX58,PRKCD,AKAP8L
GO:1902914	regulation of protein polyubiquitination	2	19	1.46	4.03E-02	9606.ENSP00000325618,9606.ENSP00000325677	PARP10,RNF40
GO:0032069	regulation of nuclease activity	2	20	1.44	4.23E-02	9606.ENSP00000296577,9606.ENSP00000378217	ABCE1,PRKCD
GO:0008285	negative regulation of cell population proliferation	8	669	0.52	4.35E-02	9606.ENSP00000233057,9606.ENSP00000268058,9606.ENSP00000325618,9606.ENSP00000347495,9606.ENSP00000354394,9606.ENSP00000360883,9606.ENSP00000371958,9606.ENSP00000399888	EIF2AK2,PML,PARP10,WARS,STAT1,IFIT3,CD9,HMGA1
GO:0060759	regulation of response to cytokine stimulus	4	162	0.83	4.43E-02	9606.ENSP00000259206,9606.ENSP00000296577,9606.ENSP00000354394,9606.ENSP00000369213	IL1RN,ABCE1,STAT1,DDX58
GO:0051457	maintenance of protein location in nucleus	2	22	1.4	4.75E-02	9606.ENSP00000268058,9606.ENSP00000343023	PML,SP100

T = 72 h, Untreated: CoV-2 / mock

#Term ID	Term Description	Genes Mapped	Enrichment Score	Direction	False Discovery Rate	Method	Matching Proteins in Your Input (IDs)	Matching Proteins in Your Input (Labels)
GO:0016266	O-glycan processing	9	4.81179	top	0.00062	afc	9606.ENSP00000169298,9606.ENSP00000287590,9606.ENSP00000355632,9606.ENSP00000360776,9606.ENSP00000381008,9606.ENSP00000414350,9606.ENSP00000417498,9606.ENSP00000436812,9606.ENSP00000484824	ST6GAL1,B3GNT7,GALNT2,B4GALT5,MUC16,MUC20,MUC4,MUC5B,MUC1
GO:0002673	regulation of acute inflammatory response	9	4.30565	top	0.0027	afc	9606.ENSP00000225698,9606.ENSP00000245907,9606.ENSP00000315130,9606.ENSP00000379191,9606.ENSP00000381607,9606.ENSP00000415941,9606.ENSP00000416561,9606.ENSP00000418770,9606.ENSP00000479488	C1QBP,C3,CLU,CD59,GSTP1,C4B,CFB,PARK7,PHB
GO:0006956	complement activation	5	6.05752	top	0.0027	afc	9606.ENSP00000225698,9606.ENSP00000245907,9606.ENSP00000315130,9606.ENSP00000415941,9606.ENSP00000416561	C1QBP,C3,CLU,C4B,CFB
GO:0030198	extracellular matrix organization	32	1.7626	top	0.0027	ks	9606.ENSP00000007722,9606.ENSP00000200181,9606.ENSP00000218388,9606.ENSP00000260227,9606.ENSP00000260408,9606.ENSP00000261769,9606.ENSP00000264144,9606.ENSP00000265131,9606.ENSP00000279441,9606.ENSP00000288235,9606.ENSP00000296585,9606.ENSP00000324532,9606.ENSP00000325941,9606.ENSP00000342098,9606.ENSP00000345344,9606.ENSP00000346032,9606.ENSP00000355361,9606.ENSP00000357980,9606.ENSP00000357981,9606.ENSP00000358983,9606.ENSP00000361405,9606.ENSP00000363827,9606.ENSP00000365569,9606.ENSP00000368646,9606.ENSP00000368678,9606.ENSP00000369055,9606.ENSP00000372221,9606.ENSP00000375778,9606.ENSP00000386896,9606.ENSP00000416330,9606.ENSP00000427552,9606.ENSP00000434412	ITGA3,ITGB4,TIMP1,MMP7,ADAM10,CDH1,LAMC2,TNC,MMP10,MYO1E,ITGA2,LAMA3,RIC8A,SPINT1,CTSL,ANXA2,CD47,HTRA1,CTSS,NFKB2,MMP9,HSPG2,FLOT1,PRDX4,AGRN,B4GALT1,SERPINB5,LAMB3,ITGA6,TGFBI,DDR1,SERPINH1
GO:0030449	regulation of complement activation	7	5.02589	top	0.0027	afc	9606.ENSP00000225698,9606.ENSP00000245907,9606.ENSP00000315130,9606.ENSP00000379191,9606.ENSP00000415941,9606.ENSP00000416561,9606.ENSP00000479488	C1QBP,C3,CLU,CD59,C4B,CFB,PHB
GO:2000257	regulation of protein activation cascade	7	5.02589	top	0.0027	afc	9606.ENSP00000225698,9606.ENSP00000245907,9606.ENSP00000315130,9606.ENSP00000379191,9606.ENSP00000415941,9606.ENSP00000416561,9606.ENSP00000479488	C1QBP,C3,CLU,CD59,C4B,CFB,PHB
GO:0009593	detection of chemical stimulus	3	8.21595	top	0.0029	afc	9606.ENSP00000291009,9606.ENSP00000348888,9606.ENSP00000415941	PIP,PIGR,C4B

GO:0043062	extracellular structure organization	35	1.82148	top	0.003	ks	9606.ENSP00000007722,9606.ENSP00000200181,9606.ENSP00000218388,9606.ENSP00000260227,9606.ENSP00000260408,9606.ENSP00000261769,9606.ENSP00000264144,9606.ENSP00000265131,9606.ENSP00000279441,9606.ENSP00000288235,9606.ENSP00000296585,9606.ENSP00000309591,9606.ENSP00000324532,9606.ENSP00000325941,9606.ENSP00000327801,9606.ENSP00000342098,9606.ENSP00000345344,9606.ENSP00000346032,9606.ENSP00000355361,9606.ENSP00000357980,9606.ENSP00000357981,9606.ENSP00000358983,9606.ENSP00000361405,9606.ENSP00000363827,9606.ENSP00000365569,9606.ENSP00000368646,9606.ENSP00000368678,9606.ENSP00000369055,9606.ENSP00000372221,9606.ENSP00000375778,9606.ENSP00000386896,9606.ENSP00000416330,9606.ENSP00000417138,9606.ENSP00000427552,9606.ENSP00000434412	ITGA3,ITGB4,TIMP1,MMP7,ADAM10,CDH1,LAMC2,TNC,MMP10,MYO1E,ITGA2,PRKACA,LAMA3,RIC8A,P4HB,SPINT1,CTSL,ANXA2,CD47,HTRA1,CTSS,NFKB2,MMP9,HSPG2,FLOT1,PRDX4,AGRN,B4GALT1,SERPINB5,LAMB3,ITGA6,TGFBI,PLTP,DDR1,SERPINH1
GO:0072376	protein activation cascade	6	5.21514	top	0.003	afc	9606.ENSP00000225698,9606.ENSP00000245907,9606.ENSP00000315130,9606.ENSP00000377055,9606.ENSP00000415941,9606.ENSP00000416561	C1QBP,C3,CLU,PRCP,C4B,CFB
GO:0002920	regulation of humoral immune response	8	4.35141	top	0.0045	afc	9606.ENSP00000225698,9606.ENSP00000245907,9606.ENSP00000315130,9606.ENSP00000379191,9606.ENSP00000391592,9606.ENSP00000415941,9606.ENSP00000416561,9606.ENSP00000479488	C1QBP,C3,CLU,CD59,PTPN6,C4B,CFB,PHB
GO:0006493	protein O-linked glycosylation	11	3.57649	top	0.0045	afc	9606.ENSP00000169298,9606.ENSP00000247020,9606.ENSP00000287590,9606.ENSP00000355632,9606.ENSP00000360776,9606.ENSP00000381008,9606.ENSP00000414350,9606.ENSP00000417498,9606.ENSP00000425809,9606.ENSP00000436812,9606.ENSP00000484824	ST6GAL1,SDF2,B3GNT7,GALNT2,B4GALT5,MUC16,MUC20,MUC4,PGM3,MUC5B,MUC1

GO:0006397	mRNA processing	158	0.316183	bottom	0.0061	ks	9606.ENSP00000215793,9606.ENSP00000215829,9606.ENSP00000216252,9606.ENSP00000216727,9606.ENSP00000217109,9606.ENSP00000221419,9606.ENSP00000221494,9606.ENSP00000225698,9606.ENSP00000225792,9606.ENSP00000227524,9606.ENSP00000228284,9606.ENSP00000229390,9606.ENSP00000238112,9606.ENSP00000240185,9606.ENSP00000242776,9606.ENSP00000243563,9606.ENSP00000244020,9606.ENSP00000246194,9606.ENSP00000249299,9606.ENSP00000253363,9606.ENSP00000254108,9606.ENSP00000254193,9606.ENSP00000254301,9606.ENSP00000258962,9606.ENSP00000261167,9606.ENSP00000261531,9606.ENSP00000261973,9606.ENSP00000262710,9606.ENSP00000263579,9606.ENSP00000263694,9606.ENSP00000265866,9606.ENSP00000265872,9606.ENSP00000266079,9606.ENSP00000266679,9606.ENSP00000269349,9606.ENSP00000276079,9606.ENSP00000283179,9606.ENSP00000291552,9606.ENSP00000293677,9606.ENSP00000297071,9606.ENSP00000300291,9606.ENSP00000300413,9606.ENSP00000301740,9606.ENSP00000302160,9606.ENSP00000304903,9606.ENSP00000305556,9606.ENSP00000305790,9606.ENSP00000307863,9606.ENSP00000309591,9606.ENSP00000310670,9606.ENSP00000310723,9606.ENSP00000311677,9606.ENSP00000313007,9606.ENSP00000313199,9606.ENSP00000313829,9606.ENSP00000316042,9606.ENSP00000317123,9606.ENSP00000318861,9606.ENSP00000319169,9606.ENSP00000324122,9606.ENSP00000325376,9606.ENSP00000325377,9606.ENSP00000325905,9606.ENSP00000326806,9606.ENSP00000328848,9606.ENSP00000335321,9606.ENSP00000336741,9	SF3A1,SNRPD3,PHF5A,PABPN1,CSTF1,HNRNPL,SF3A2,C1QBP,DDX5,PRPF19,SART3,SRSF9,CPSF3,TARDBP,DDX39A,SNRPA,SRSF6,RALY,LSM8,RBM39,FUS,SNRPA1,LGALS3,SRSF1,WBP11,SNW1,RBM25,ACIN1,DCPS,SNRNP40,HNRNPH3,CCAR1,PRPF6,CPSF6,EIF4A3,NONO,HNRNPU,U2AFBP,RAVER1,TRA2A,NUDT21,SNRPD1,SRRM2,LSM3,CD2BP2,PCBP1,SF3B3,U2AF2,PRKACA,CCAR2,DDX23,PPP1R8,PABPC1,HNRNPD,KHDRBS1,HNRNPA0,SNRNP200,SF3B2,PRMT5,PRPF31,HNRNPM,WDR33,SRSF7,NCBP2,RBM10,SF3B1,DHX15,FIP1L1,PRPF4B,SARNP,ZNF326,GTF2F2,HNRNPA1,SNRPD2,PSPC1,CPSF7,THRAP3,HNRNPA2B1,CGI-74,SON,HNRNPH1,PTBP1,SFPQ,WTAP,PCBP2,CTNNBL1,CDC73,DHX9,ADAR,SYNCRIP,RBM15,AHCYL1,FMR1,RBMX,SRSF11,CDC5L,YBX1,HNRNPH2,CSTF2,SF3A3,SRSF3,PRPF4,HNRNPR,NCBP1,LSM2,HNRNPK,XRN2,SF1,POLR2B,DDX1,RNMT,HNRNPUL1,SRSF2,HNRNPA3,GTF2F1,DDX39B,DDX17,SMU1,KHSRP,KDM1A,NHP2L1,ELAVL1,PRPF40A,RBM4,RBM17,EFTUD2,STRAP,POLDIP3,HNRNPF,SNRPE,PNPT1,ESRP1,SNRPB,PTBP3,DDX46,TRA2B,U2SURP,SRSF10,ALYREF,PLRG1,LUC7L3,HSPA8,CLNS1A,PUF60,CHERP,HTATSF1,HNRNPC,SRSF5,RNPS1,PRPF8,RBM8A,DDX42,LSM4,SUPT5H,SNRNP70,POLR2A,SRRT,CPSF1

GO:0016999	antibiotic metabolic process	49	1.00873	top	0.0061	ks	9606.ENSP00000169298,9606.ENSP00000216254,9606.ENSP00000222673,9606.ENSP00000229329,9606.ENSP00000235835,9606.ENSP00000241052,9606.ENSP00000253382,9606.ENSP00000253792,9606.ENSP00000261733,9606.ENSP00000262746,9606.ENSP00000264932,9606.ENSP00000265462,9606.ENSP00000270142,9606.ENSP00000280346,9606.ENSP00000285930,9606.ENSP00000296412,9606.ENSP00000297785,9606.ENSP00000298510,9606.ENSP00000299518,9606.ENSP00000301522,9606.ENSP00000307241,9606.ENSP00000309477,9606.ENSP00000317997,9606.ENSP00000321070,9606.ENSP00000327070,9606.ENSP00000331897,9606.ENSP00000335304,9606.ENSP00000342026,9606.ENSP00000342056,9606.ENSP00000352584,9606.ENSP00000355518,9606.ENSP00000364649,9606.ENSP00000367992,9606.ENSP00000369134,9606.ENSP00000369927,9606.ENSP00000370129,9606.ENSP00000370223,9606.ENSP00000377446,9606.ENSP00000378972,9606.ENSP00000390265,9606.ENSP00000391596,9606.ENSP00000407375,9606.ENSP00000418770,9606.ENSP00000419325,9606.ENSP00000420269,9606.ENSP00000426083,9606.ENSP00000438144,9606.ENSP00000473990,9606.ENSP00000477639	ST6GAL1,ACO2,OGDH,CMAS,AKR7A2,CAT,ACSS2,ACLY,ALDH2,PRDX1,SDHA,PRDX5,SOD1,DLAT,AKR1B1,ADH5,ALDH1A1,PRDX3,IDH3A,PRDX2,PDHB,ACO1,DUOX1,ME2,MDH2,IDH2,DLST,PRDX6,CS,AKR1B10,FH,SDHB,ESD,PDHA1,AKR1C3,AKR1C2,IDH3B,SUCLG1,SULT1A1,IDH1,AMDHD2,GPX1,PARK7,SUCLG2,ADH7,ADH1C,MDH1,ALDH3B1,NAGK
GO:0097237	cellular response to toxic substance	50	1.04019	top	0.0061	ks	9606.ENSP00000012443,9606.ENSP00000216465,9606.ENSP00000216714,9606.ENSP00000217515,9606.ENSP00000221130,9606.ENSP00000223136,9606.ENSP00000226574,9606.ENSP00000241052,9606.ENSP00000250101,9606.ENSP00000262746,9606.ENSP00000265462,9606.ENSP00000270142,9606.ENSP00000278572,9606.ENSP00000287295,9606.ENSP00000296412,9606.ENSP00000298510,9606.ENSP00000300134,9606.ENSP00000301522,9606.ENSP00000317997,9606.ENSP00000319788,9606.ENSP00000335620,9606.ENSP00000337443,9606.ENSP00000341826,9606.ENSP00000342026,9606.ENSP00000346103,9606.ENSP00000346148,9606.ENSP00000349960,9606.ENSP00000356864,9606.ENSP00000357727,9606.ENSP00000358727,9606.ENSP00000363641,9606.ENSP00000366109,9606.ENSP00000367992,9606.ENSP00000368458,9606.ENSP00000368646,9606.ENSP00000373905,9606.ENSP00000376506,9606.ENSP00000378040,9606.ENSP00000379512,9606.ENSP00000381448,9606.ENSP00000381607,9606.ENSP00000384273,9606.ENSP00000407375,9606.ENSP00000413218,9606.ENSP00000418770,9606.ENSP00000430432,9606.ENSP00000431049,9606.ENSP00000434516,9606.ENSP00000446252,9606.ENSP00000482445	PPP5C,GSTZ1,APEX1,TXNL1,GSR,FIS1,NFKB1,CAT,TXNDC17,PRDX1,PRDX5,SOD1,RPS3,AIFM1,ADH5,PRDX3,STAT6,PRDX2,DUOX1,NQO1,GSTA1,NXN,HNRNPA1,PRDX6,GPX4,PRKAA1,ACTB,MGST3,S100A9,GSTO1,TXN,ANXA1,ESD,PCNA,PRDX4,DNM2,PDCD10,BAD,MGST1,CST3,GSTP1,RELA,GPX1,FAM213B,PARK7,HDAC2,GSTK1,TXNRD1,SOD2,FAM213A

GO:0008380	RNA splicing	151	0.311357	bottom	0.0062	ks	9606.ENSP00000215793,9606.ENSP00000215829,9606.ENSP00000216038,9606.ENSP00000216252,9606.ENSP00000216727,9606.ENSP00000217109,9606.ENSP00000221419,9606.ENSP00000221494,9606.ENSP00000225698,9606.ENSP00000225792,9606.ENSP00000227524,9606.ENSP00000228284,9606.ENSP00000229390,9606.ENSP00000238112,9606.ENSP00000240185,9606.ENSP00000242776,9606.ENSP00000243563,9606.ENSP00000244020,9606.ENSP00000246194,9606.ENSP00000249299,9606.ENSP00000253363,9606.ENSP00000254108,9606.ENSP00000254193,9606.ENSP00000254301,9606.ENSP00000258962,9606.ENSP00000261167,9606.ENSP00000261531,9606.ENSP00000261700,9606.ENSP00000261973,9606.ENSP00000262710,9606.ENSP00000263579,9606.ENSP00000263694,9606.ENSP00000265866,9606.ENSP00000265872,9606.ENSP00000266079,9606.ENSP00000269349,9606.ENSP00000276079,9606.ENSP00000283179,9606.ENSP00000291552,9606.ENSP00000293677,9606.ENSP00000297071,9606.ENSP00000300291,9606.ENSP00000300413,9606.ENSP00000301740,9606.ENSP00000302160,9606.ENSP00000304903,9606.ENSP00000305556,9606.ENSP00000305790,9606.ENSP00000306117,9606.ENSP00000307863,9606.ENSP00000310670,9606.ENSP00000310723,9606.ENSP00000311677,9606.ENSP00000313007,9606.ENSP00000313199,9606.ENSP00000316042,9606.ENSP00000317123,9606.ENSP00000318861,9606.ENSP00000319169,9606.ENSP00000324122,9606.ENSP00000324804,9606.ENSP00000325376,9606.ENSP00000325377,9606.ENSP00000325905,9606.ENSP00000326806,9606.ENSP00000328848,9606.ENSP00000335321,9	SF3A1,SNRPD3,RTCB,PHF5A,PABPN1,CSTF1,HNRNPL,SF3A2,C1QBP,DDX5,PRPF19,SART3,SRSF9,CPSF3,TARDBP,DDX39A,SNRPA,SRSF6,RALY,LSM8,RBM39,FUS,SNRPA1,LGALS3,SRSF1,WBP11,SNW1,C14orf166,RBM25,ACIN1,DCPS,SNRNP40,HNRNPH3,CCAR1,PRPF6,EIF4A3,NONO,HNRNPU,U2AFBP,RAVER1,TRA2A,NUDT21,SNRPD1,SRRM2,LSM3,CD2BP2,PCBP1,SF3B3,DDX19A,U2AF2,CCAR2,DDX23,PPP1R8,PABPC1,HNRNPD,HNRNPA0,SNRNP200,SF3B2,PRMT5,PRPF31,PPP2R1A,HNRNPM,WDR33,SRSF7,NCBP2,RBM10,SF3B1,DHX15,FIP1L1,PRPF4B,ZNF326,GTF2F2,HNRNPA1,SNRPD2,PSPC1,CPSF7,THRAP3,HNRNPA2B1,CGI-74,SON,HNRNPH1,PTBP1,SFPQ,WTAP,PCBP2,CTNNBL1,DHX9,SYNCRIP,RBM15,FMR1,RBMX,SRSF11,CDC5L,YBX1,HNRNPH2,CSTF2,SF3A3,SRSF3,PRPF4,HNRNPR,NCBP1,LSM2,HNRNPK,SF1,POLR2B,DDX1,HNRNPUL1,SRSF2,HNRNPA3,GTF2F1,DDX39B,DDX17,SMU1,KHSRP,KDM1A,NHP2L1,ELAVL1,PRPF40A,RBM4,RBM17,EFTUD2,STRAP,HNRNPF,SNRPE,ESRP1,SNRPB,PTBP3,DDX46,TRA2B,PPP2CA,U2SURP,SRSF10,ALYREF,PLRG1,LUC7L3,HSPA8,CLNS1A,PUF60,CHERP,HTATSF1,HNRNPC,SRSF5,RNPS1,PRPF8,RBM8A,DDX42,LSM4,SNRNP70,POLR2A,SRRT,CPSF1
GO:0019730	antimicrobial humoral response	16	2.69954	top	0.0062	afc	9606.ENSP00000229239,9606.ENSP00000253354,9606.ENSP00000254301,9606.ENSP00000315130,9606.ENSP00000342082,9606.ENSP00000346251,9606.ENSP00000355315,9606.ENSP00000357722,9606.ENSP00000357727,9606.ENSP00000357905,9606.ENSP00000362108,9606.ENSP00000379873,9606.ENSP00000428085,9606.ENSP00000435370,9606.ENSP00000452780,9606.ENSP00000470972	GAPDH,BPIFB1,LGALS3,CLU,SLPI,BPIFA1,RPL39,S100A8,S100A9,DMBT1,LCN2,HLA-A,RPL30,FAU,B2M,RPS19

GO:1990748	cellular detoxification	32	1.35613	top	0.0062	ks	9606.ENSP00000216465,9606.ENSP00000217515,9606.ENSP00000221130,9606.ENSP00000241052,9606.ENSP00000250101,9606.ENSP00000262746,9606.ENSP00000265462,9606.ENSP00000270142,9606.ENSP00000296412,9606.ENSP00000298510,9606.ENSP00000301522,9606.ENSP00000317997,9606.ENSP00000319788,9606.ENSP00000335620,9606.ENSP00000337443,9606.ENSP00000342026,9606.ENSP00000346103,9606.ENSP00000356864,9606.ENSP00000357727,9606.ENSP00000358727,9606.ENSP00000363641,9606.ENSP00000367992,9606.ENSP00000368646,9606.ENSP00000379512,9606.ENSP00000381607,9606.ENSP00000407375,9606.ENSP00000413218,9606.ENSP00000418770,9606.ENSP00000431049,9606.ENSP00000434516,9606.ENSP00000446252,9606.ENSP00000482445	GSTZ1,TXNL1,GSR,CAT,TXNDC17,PRDX1,PRDX5,SOD1,ADH5,PRDX3,PRDX2,DUOX1,NQO1,GSTA1,NXN,PRDX6,GPX4,MGST3,S100A9,GSTO1,TXN,ESD,PRDX4,MGST1,GSTP1,GPX1,FAM213B,PARK7,GSTK1,TXNRD1,SOD2,FAM213A
GO:0060627	regulation of vesicle-mediated transport	92	0.959664	top	0.0077	ks	9606.ENSP00000161559,9606.ENSP00000215832,9606.ENSP00000220325,9606.ENSP00000230085,9606.ENSP00000238497,9606.ENSP00000239940,9606.ENSP00000245907,9606.ENSP00000247470,9606.ENSP00000250559,9606.ENSP00000252699,9606.ENSP00000253083,9606.ENSP00000254301,9606.ENSP00000260130,9606.ENSP00000261263,9606.ENSP00000262948,9606.ENSP00000263025,9606.ENSP00000263246,9606.ENSP00000263277,9606.ENSP00000263864,9606.ENSP00000264710,9606.ENSP00000265062,9606.ENSP00000265562,9606.ENSP00000268150,9606.ENSP00000269886,9606.ENSP00000296585,9606.ENSP00000303366,9606.ENSP00000304236,9606.ENSP00000307078,9606.ENSP00000310491,9606.ENSP00000312999,9606.ENSP00000314615,9606.ENSP00000315130,9606.ENSP00000320043,9606.ENSP00000320866,9606.ENSP00000331201,9606.ENSP00000333298,9606.ENSP00000333547,9606.ENSP00000337691,9606.ENSP00000345195,9606.ENSP00000345347,9606.ENSP00000346032,9606.ENSP00000346148,9606.ENSP00000348461,9606.ENSP00000351926,9606.ENSP00000352264,9606.ENSP00000353408,9606.ENSP00000354376,9606.ENSP00000355361,9606.ENSP00000356042,9606.ENSP00000359506,9606.ENSP00000361072,9606.ENSP00000362946,9606.ENSP00000365439,9606.ENSP00000365569,9606.ENSP00000366109,9606.ENSP00000366565,9606.ENSP00000366843,9606.ENSP00000370542,9606.ENSP00000373905,9606.ENSP00000376333,9606.ENSP00000377015,9606.ENSP00000377148,9606.ENSP00000378323,9606.ENSP00000378856,9606.ENSP00000381293,9606.ENSP00000384136,9606.ENSP00000390783,9	CEACAM1,MAPK1,EHD4,SNX3,VPS4B,PFN2,C3,PYCARD,RAP1B,ACTN4,HIP1R,LGALS3,SDCBP,RAB21,MAP2K2,MAPK3,PACSIN2,EHD2,VAMP8,RAB10,RAB7A,PTPN23,MFGE8,SH3GL1,ITGA2,LMAN2,CD14,KIF5B,ARHGAP1,GNAI2,ARFGAP1,CLU,LYPLA1,CALR,HGS,LAMP1,RAB11B,MAPK15,UBQLN2,HMGB1,ANXA2,PRKAA1,RAC1,AP2A1,CD2AP,MSN,RAB25,CD47,EZR,FMR1,TSPAN1,RAB14,HNRNPK,FLOT1,ANXA1,VPS28,ATXN2,SDC1,DNM2,LLGL2,PICALM,AP1G1,PPP3CA,LGALS9,NSF,RDX,SCFD1,PPT1,CHMP3,PDCD6IP,STXBP2,C4B,CDK5,GNB2L1,SEC24B,LYN,MYO18A,ARF1,ATP2A2,SYTL1,PACSIN3,RAB5C,CD63,DNM1L,B2M,ALOX15,ANKFY1,CHMP2A,CAMK1D,EHD1,SNX12,AP2B1
GO:0007606	sensory perception of chemical stimulus	4	5.90005	top	0.009	afc	9606.ENSP00000291009,9606.ENSP00000348888,9606.ENSP00000360141,9606.ENSP00000481878	PIP,PIGR,GNAS,GNB1

T = 72 h, IFNα5: CoV-2 / mock

#Term ID	Term Description	Genes Mapped	Enrichment Score	Direction	False Discovery Rate	Method	Matching Proteins in Your Input (IDs)	Matching Proteins in Your Input (Labels)
GO:0002673	regulation of acute inflammatory response	10	5.47022	top	0.0000261	afc	9606.ENSP00000225698,9606.ENSP00000245907,9606.ENSP00000315130,9606.ENSP00000356399,9606.ENSP00000379191,9606.ENSP00000381607,9606.ENSP00000415941,9606.ENSP00000416561,9606.ENSP00000418770,9606.ENSP00000479488	C1QBP,C3,CLU,CFH,CD59,GSTP1,C4B,CFB,PARK7,PHB
GO:0002920	regulation of humoral immune response	9	5.57206	top	0.0000261	afc	9606.ENSP00000225698,9606.ENSP00000245907,9606.ENSP00000315130,9606.ENSP00000356399,9606.ENSP00000379191,9606.ENSP00000391592,9606.ENSP00000415941,9606.ENSP00000416561,9606.ENSP00000479488	C1QBP,C3,CLU,CFH,CD59,PTPN6,C4B,CFB,PHB
GO:0006956	complement activation	6	7.14134	top	0.0000261	afc	9606.ENSP00000225698,9606.ENSP00000245907,9606.ENSP00000315130,9606.ENSP00000356399,9606.ENSP00000415941,9606.ENSP00000416561	C1QBP,C3,CLU,CFH,C4B,CFB
GO:0030449	regulation of complement activation	8	6.2269	top	0.0000261	afc	9606.ENSP00000225698,9606.ENSP00000245907,9606.ENSP00000315130,9606.ENSP00000356399,9606.ENSP00000379191,9606.ENSP00000415941,9606.ENSP00000416561,9606.ENSP00000479488	C1QBP,C3,CLU,CFH,CD59,C4B,CFB,PHB
GO:0072376	protein activation cascade	7	6.11034	top	0.0000261	afc	9606.ENSP00000225698,9606.ENSP00000245907,9606.ENSP00000315130,9606.ENSP00000356399,9606.ENSP00000377055,9606.ENSP00000415941,9606.ENSP00000416561	C1QBP,C3,CLU,CFH,PRCP,C4B,CFB
GO:2000257	regulation of protein activation cascade	8	6.2269	top	0.0000261	afc	9606.ENSP00000225698,9606.ENSP00000245907,9606.ENSP00000315130,9606.ENSP00000356399,9606.ENSP00000379191,9606.ENSP00000415941,9606.ENSP00000416561,9606.ENSP00000479488	C1QBP,C3,CLU,CFH,CD59,C4B,CFB,PHB
GO:0016266	O-glycan processing	10	4.7641	top	0.00013	afc	9606.ENSP00000169298,9606.ENSP00000287590,9606.ENSP00000321874,9606.ENSP00000355632,9606.ENSP00000360776,9606.ENSP00000381008,9606.ENSP00000414350,9606.ENSP00000417498,9606.ENSP00000436812,9606.ENSP00000484824	ST6GAL1,B3GNT7,B3GNT3,GALNT2,B4GALT5,MUC16,MUC20,MUC4,MUC5B,MUC1
GO:0006959	humoral immune response	26	3.66066	top	0.00031	ks	9606.ENSP00000169298,9606.ENSP00000225698,9606.ENSP00000229239,9606.ENSP00000245907,9606.ENSP00000253354,9606.ENSP00000254301,9606.ENSP00000315130,9606.ENSP00000342082,9606.ENSP00000346251,9606.ENSP00000351314,9606.ENSP00000353099,9606.ENSP00000355315,9606.ENSP00000356399,9606.ENSP00000357722,9606.ENSP00000357727,9606.ENSP00000357905,9606.ENSP00000362108,9606.ENSP00000379873,9606.ENSP00000405573,9606.ENSP00000415941,9606.ENSP00000416561,9606.ENSP00000428085,9606.ENSP00000435370,9606.ENSP00000452780,9606.ENSP00000470972,9606.ENSP00000483599	ST6GAL1,C1QBP,GAPDH,C3,BPIFB1,LGALS3,CLU,SLPI,BPIFA1,PSMB10,HLA-DRB1,RPL39,CFH,S100A8,S100A9,DMBT1,LCN2,HLA-A,GPI,C4B,CFB,RPL30,FAU,B2M,RPS19,SPON2
GO:0019730	antimicrobial humoral response	17	2.22974	top	0.00031	afc	9606.ENSP00000229239,9606.ENSP00000253354,9606.ENSP00000254301,9606.ENSP00000315130,9606.ENSP00000342082,9606.ENSP00000346251,9606.ENSP00000355315,9606.ENSP00000357722,9606.ENSP00000357727,9606.ENSP00000357905,9606.ENSP00000362108,9606.ENSP00000379873,9606.ENSP00000428085,9606.ENSP00000435370,9606.ENSP00000452780,9606.ENSP00000470972,9606.ENSP00000483599	GAPDH,BPIFB1,LGALS3,CLU,SLPI,BPIFA1,RPL39,S100A8,S100A9,DMBT1,LCN2,HLA-A,RPL30,FAU,B2M,RPS19,SPON2

GO:0006412	translation	161	0.689132	bottom	0.00083	ks	9606.ENSP00000177742,9606.ENSP00000216190,9606.ENSP00000216554,9606.ENSP00000220849,9606.ENSP00000222247,9606.ENSP00000223029,9606.ENSP00000225430,9606.ENSP00000230050,9606.ENSP00000231572,9606.ENSP00000233057,9606.ENSP00000234677,9606.ENSP00000248342,9606.ENSP00000251453,9606.ENSP00000253039,9606.ENSP00000253099,9606.ENSP00000253108,9606.ENSP00000256383,9606.ENSP00000256854,9606.ENSP00000259469,9606.ENSP00000261772,9606.ENSP00000261868,9606.ENSP00000262027,9606.ENSP00000262584,9606.ENSP00000264161,9606.ENSP00000265753,9606.ENSP00000266126,9606.ENSP00000270625,9606.ENSP00000272317,9606.ENSP00000278572,9606.ENSP00000281828,9606.ENSP00000288937,9606.ENSP00000289371,9606.ENSP00000293831,9606.ENSP00000296456,9606.ENSP00000296577,9606.ENSP00000296674,9606.ENSP00000300648,9606.ENSP00000301457,9606.ENSP00000302896,9606.ENSP00000307567,9606.ENSP00000307889,9606.ENSP00000307940,9606.ENSP00000308275,9606.ENSP00000309334,9606.ENSP00000309830,9606.ENSP00000311430,9606.ENSP00000312311,9606.ENSP00000313007,9606.ENSP00000318646,9606.ENSP00000320309,9606.ENSP00000322419,9606.ENSP00000322439,9606.ENSP00000325448,9606.ENSP00000326381,9606.ENSP00000331901,9606.ENSP00000332604,9606.ENSP00000336702,9606.ENSP00000336721,9606.ENSP00000339063,9606.ENSP00000339095,9606.ENSP00000339795,9606.ENSP00000341885,9606.ENSP00000343885,9606.ENSP00000345957,9606.ENSP00000346001,9606.ENSP00000346015,9606.ENSP00000346027,9606.ENSP00000346037,9606.ENSP00000346046,9606.ENSP00000346050,9606.ENSP00000346067,9606.ENSP00000346088,9606.ENSP00000347495,9606.ENSP00000348849,9606.ENSP00000350512,9606.ENSP000003535	MRPS34,EIF3D,EIF5,EIF3E,RPL18A,AIMP2,RPL19,RPS12,RARS,EIF2AK2,SARS,EIF3K,RPS16,EIF2S3,MRPL4,EIF3G,EIF2S1,NARS,RPL35,AARS,EIF3J,MARS,RPL8,DARS,EIF4H,EIF2B2,RPS11,RPS27A,RPS3,FARSB,MRPL17,EIF5B,EIF4A1,APEH,ABCE1,RPS23,GCN1L1,NDUFA7,RPS9,QARS,RPL13,EEF2,MRPL38,RPL15,RPL38,RPL4,MRPL46,PABPC1,RPS15A,FARSA,RPLP2,TUFM,KARS,EIF4A2,EEF1G,EIF3C,EIF5A,LARP1,EEF1A1,RPS7,RPL7,RPS2,PPA2,RPS21,RPL3,RPL27A,RPL21,RPLP1,RPS17,RPS3A,RPSA,RPL22,WARS,RPS26,COPS5,EIF2B3,ETF1,EIF3B,RPL12,RPL39,IARS2,EPRS,RPS27,EIF3A,RPL5,MCTS1,MRPS2,RPL7A,MRPL14,MRPS18A,PABPC4,PPA1,YARS,EIF3I,RPS4X,RPL10A,EIF6,RPL11,EIF2S2,IARS,VARS,RRBP1,EEF1E1,RPS6,CARS,GARS,RPL13A,EEF1B2,RPL24,LARS,RPL34,AIMP1,RPL14,RPS8,RPL23L,LARP4,DDX3X,RPS14,RPL31,RBM4,TARS,EIF4B,RPL23A,RPS18,EIF2B4,RPL17,GSPT1,RPL9,AARSD1,RPL6,EEF1D,RPL10,RPS24,EIF4G1,RPL32,EIF2A,RPL29,RPL35A,RPL23,EIF4E,HARS,GNB2L1,RPL30,RPS20,EIF3F,EIF4G2,RPS25,FAU,RPS13,RPL18,NACA,RPLP0,RPL28,RPL26,RPL36,RPL27,RPS19,RPS28,RPS5,RPS10,EIF3L
GO:0070613	regulation of protein processing	15	3.38208	top	0.00085	afc	9606.ENSP00000216181,9606.ENSP00000217131,9606.ENSP00000225698,9606.ENSP00000234590,9606.ENSP00000245907,9606.ENSP00000315130,9606.ENSP00000356399,9606.ENSP00000362924,9606.ENSP00000379157,9606.ENSP00000379191,9606.ENSP00000415941,9606.ENSP00000416561,9606.ENSP00000457243,9606.ENSP00000479488,9606.ENSP00000481314	MYH9,CTSZ,C1QBP,ENO1,C3,CLU,CFH,GSN,CAST,CD59,C4B,CFB,NOL3,PHB,SNX12
GO:0002385	mucosal immune response	5	4.46358	top	0.0012	afc	9606.ENSP00000253354,9606.ENSP00000346251,9606.ENSP00000348888,9606.ENSP00000355315,9606.ENSP00000435370	BPIFB1,BPIFA1,PIGR,RPL39,FAU
GO:0006493	protein O-linked glycosylation	12	3.79932	top	0.0012	afc	9606.ENSP00000169298,9606.ENSP00000247020,9606.ENSP00000287590,9606.ENSP00000321874,9606.ENSP00000355632,9606.ENSP00000360776,9606.ENSP00000381008,9606.ENSP00000414350,9606.ENSP00000417498,9606.ENSP00000425809,9606.ENSP00000436812,9606.ENSP00000484824	ST6GAL1,SDF2,B3GNT7,B3GNT3,GALNT2,B4GALT5,MUC16,MUC20,MUC4,PGM3,MUC5B,MUC1
GO:0009593	detection of chemical stimulus	3	8.27984	top	0.0012	afc	9606.ENSP00000291009,9606.ENSP00000348888,9606.ENSP00000415941	PIP,PIGR,C4B
GO:0019731	antibacterial humoral response	8	3.15967	top	0.0014	afc	9606.ENSP00000342082,9606.ENSP00000346251,9606.ENSP00000355315,9606.ENSP00000357905,9606.ENSP00000379873,9606.ENSP00000435370,9606.ENSP00000452780,9606.ENSP00000483599	SLPI,BPIFA1,RPL39,DMBT1,HLA-A,FAU,B2M,SPON2
GO:0006957	complement activation, alternative pathway	3	8.18558	top	0.0015	afc	9606.ENSP00000245907,9606.ENSP00000356399,9606.ENSP00000416561	C3,CFH,CFB

GO:0006413	translational initiation	107	0.849951	bottom	0.0019	ks	9606.ENSP00000216190,9606.ENSP00000216554,9606.ENSP00000220849,9606.ENSP00000222247,9606.ENSP00000225430,9606.ENSP00000230050,9606.ENSP00000248342,9606.ENSP00000251453,9606.ENSP00000253039,9606.ENSP00000253108,9606.ENSP00000256383,9606.ENSP00000259469,9606.ENSP00000261868,9606.ENSP00000262584,9606.ENSP00000265753,9606.ENSP00000266126,9606.ENSP00000270625,9606.ENSP00000272317,9606.ENSP00000278572,9606.ENSP00000289371,9606.ENSP00000293831,9606.ENSP00000296577,9606.ENSP00000296674,9606.ENSP00000302896,9606.ENSP00000307889,9606.ENSP00000309334,9606.ENSP00000309830,9606.ENSP00000311430,9606.ENSP00000313007,9606.ENSP00000318646,9606.ENSP00000322419,9606.ENSP00000326381,9606.ENSP00000332604,9606.ENSP00000336721,9606.ENSP00000339095,9606.ENSP00000339795,9606.ENSP00000341885,9606.ENSP00000345957,9606.ENSP00000346001,9606.ENSP00000346015,9606.ENSP00000346027,9606.ENSP00000346037,9606.ENSP00000346046,9606.ENSP00000346050,9606.ENSP00000346067,9606.ENSP00000346088,9606.ENSP00000348849,9606.ENSP00000350512,9606.ENSP00000353575,9606.ENSP00000354125,9606.ENSP00000354739,9606.ENSP00000355315,9606.ENSP00000357555,9606.ENSP00000358140,9606.ENSP00000359345,9606.ENSP00000360365,9606.ENSP00000361076,9606.ENSP00000362688,9606.ENSP00000362744,9606.ENSP00000363018,9606.ENSP00000363574,9606.ENSP00000363676,9606.ENSP00000364119,9606.ENSP00000369757,9606.ENSP00000375730,9606.ENSP00000377640,9606.ENSP00000378163,9606.ENSP00000379506,9606.ENSP00000379888,9606.ENSP00000382840,9606.ENSP00000385958,9606.ENSP00000386717,9606.ENSP00000386894,9606.ENSP00000388806,9606.ENSP00000389103,9606.ENSP000003932	EIF3D,EIF5,EIF3E,RPL18A,RPL19,RPS12,EIF3K,RPS16,EIF2S3,EIF3G,EIF2S1,RPL35,EIF3J,RPL8,EIF4H,EIF2B2,RPS11,RPS27A,RPS3,EIF5B,EIF4A1,ABCE1,RPS23,RPS9,RPL13,RPL15,RPL38,RPL4,PABPC1,RPS15A,RPLP2,EIF4A2,EIF3C,LARP1,RPS7,RPL7,RPS2,RPS21,RPL3,RPL27A,RPL21,RPLP1,RPS17,RPS3A,RPSA,RPL22,RPS26,COPS5,EIF2B3,EIF3B,RPL12,RPL39,RPS27,EIF3A,RPL5,MCTS1,RPL7A,EIF3I,RPS4X,RPL10A,EIF6,RPL11,EIF2S2,RPS6,RPL13A,RPL24,RPL34,RPL14,RPS8,DDX3X,RPS14,RPL31,RBM4,EIF4B,RPL23A,RPS18,EIF2B4,RPL17,RPL9,RPL6,RPL10,RPS24,EIF4G1,RPL32,EIF2A,RPL29,RPL35A,RPL23,EIF4E,RPL30,RPS20,EIF3F,EIF4G2,RPS25,FAU,RPS13,RPL18,RPLP0,RPL28,RPL26,RPL36,RPL27,RPS19,RPS28,RPS5,RPS10,EIF3L
GO:0006958	complement activation, classical pathway	4	6.62481	top	0.003	afc	9606.ENSP00000225698,9606.ENSP00000245907,9606.ENSP00000315130,9606.ENSP00000415941	C1QBP,C3,CLU,C4B

GO:0043043	peptide biosynthetic process	169	0.652961	bottom	0.0033	ks	9606.ENSP00000177742,9606.ENSP00000216190,9606.ENSP00000216554,9606.ENSP00000216951,9606.ENSP00000220849,9606.ENSP00000222247,9606.ENSP00000223029,9606.ENSP00000225430,9606.ENSP00000229416,9606.ENSP00000230050,9606.ENSP00000231572,9606.ENSP00000233057,9606.ENSP00000234677,9606.ENSP00000248342,9606.ENSP00000251453,9606.ENSP00000253039,9606.ENSP00000253099,9606.ENSP00000253108,9606.ENSP00000256383,9606.ENSP00000256854,9606.ENSP00000259469,9606.ENSP00000261772,9606.ENSP00000261868,9606.ENSP00000262027,9606.ENSP00000262584,9606.ENSP00000264161,9606.ENSP00000265753,9606.ENSP00000266126,9606.ENSP00000270625,9606.ENSP00000272317,9606.ENSP00000275428,9606.ENSP00000278572,9606.ENSP00000281828,9606.ENSP00000288937,9606.ENSP00000289371,9606.ENSP00000293831,9606.ENSP00000296424,9606.ENSP00000296456,9606.ENSP00000296577,9606.ENSP00000296674,9606.ENSP00000300648,9606.ENSP00000301457,9606.ENSP00000302896,9606.ENSP00000307567,9606.ENSP00000307889,9606.ENSP00000307940,9606.ENSP00000308275,9606.ENSP00000309334,9606.ENSP00000309830,9606.ENSP00000311430,9606.ENSP00000312311,9606.ENSP00000313007,9606.ENSP00000318646,9606.ENSP00000320309,9606.ENSP00000322419,9606.ENSP00000322439,9606.ENSP00000325448,9606.ENSP00000325548,9606.ENSP00000326381,9606.ENSP00000331901,9606.ENSP00000332604,9606.ENSP00000336702,9606.ENSP00000336721,9606.ENSP00000339063,9606.ENSP00000339095,9606.ENSP00000339795,9606.ENSP00000341885,9606.ENSP00000343885,9606.ENSP00000345957,9606.ENSP00000346001,9606.ENSP00000346015,9606.ENSP00000346027,9606.ENSP00000346037,9606.ENSP00000346046,9606.ENSP00000346050,9606.ENSP000003460	MRPS34,EIF3D,EIF5,GSS,EIF3E,RPL18A,AIMP2,RPL19,GCLC,RPS12,RARS,EIF2AK2,SARS,EIF3K,RPS16,EIF2S3,MRPL4,EIF3G,EIF2S1,NARS,RPL35,AARS,EIF3J,MARS,RPL8,DARS,EIF4H,EIF2B2,RPS11,RPS27A,GGCT,RPS3,FARSB,MRPL17,EIF5B,EIF4A1,BDH2,APEH,ABCE1,RPS23,GCN1L1,NDUFA7,RPS9,QARS,RPL13,EEF2,MRPL38,RPL15,RPL38,RPL4,MRPL46,PABPC1,RPS15A,FARSA,RPLP2,TUFM,KARS,CNDP2,EIF4A2,EEF1G,EIF3C,EIF5A,LARP1,EEF1A1,RPS7,RPL7,RPS2,PPA2,RPS21,RPL3,RPL27A,RPL21,RPLP1,RPS17,RPS3A,RPSA,RPL22,WARS,RPS26,COPS5,EIF2B3,ETF1,EIF3B,RPL12,RPL39,IARS2,EPRS,RPS27,EIF3A,GCLM,RPL5,MCTS1,MRPS2,RPL7A,MRPL14,MRPS18A,PABPC4,PPA1,YARS,EIF3I,RPS4X,RPL10A,EIF6,RPL11,EIF2S2,IARS,VARS,RRBP1,EEF1E1,RPS6,CARS,GARS,RPL13A,EEF1B2,RPL24,LARS,RPL34,AIMP1,RPL14,RPS8,RPL23L,HAGH,LARP4,DDX3X,RPS14,RPL31,RBM4,TARS,EIF4B,RPL23A,RPS18,EIF2B4,RPL17,GSPT1,RPL9,AARSD1,RPL6,EEF1D,RPL10,RPS24,EIF4G1,RPL32,EIF2A,RPL29,RPL35A,RPL23,EIF4E,HARS,GNB2L1,RPL30,RPS20,EIF3F,EIF4G2,RPS25,FAU,RPS13,RPL18,NACA,RPLP0,RPL28,RPL26,RPL36,RPL27,RPS19,RPS28,RPS5,OPLAH,RPS10,EIF3L

GO:0043604	amide biosynthetic process	188	0.63863	bottom	0.0067	ks	9606.ENSP00000175506,9606.ENSP00000177742,9606.ENSP00000205402,9606.ENSP00000215567,9606.ENSP00000215882,9606.ENSP00000216190,9606.ENSP00000216554,9606.ENSP00000216605,9606.ENSP00000216951,9606.ENSP00000220849,9606.ENSP00000222247,9606.ENSP00000223029,9606.ENSP00000225430,9606.ENSP00000229416,9606.ENSP00000230050,9606.ENSP00000231572,9606.ENSP00000233057,9606.ENSP00000234677,9606.ENSP00000236959,9606.ENSP00000248342,9606.ENSP00000251453,9606.ENSP00000253039,9606.ENSP00000253099,9606.ENSP00000253108,9606.ENSP00000253382,9606.ENSP00000253792,9606.ENSP00000256383,9606.ENSP00000256854,9606.ENSP00000259469,9606.ENSP00000261772,9606.ENSP00000261868,9606.ENSP00000262027,9606.ENSP00000262584,9606.ENSP00000264161,9606.ENSP00000265753,9606.ENSP00000265838,9606.ENSP00000266126,9606.ENSP00000270625,9606.ENSP00000272317,9606.ENSP00000275428,9606.ENSP00000278572,9606.ENSP00000280346,9606.ENSP00000281828,9606.ENSP00000288937,9606.ENSP00000289371,9606.ENSP00000293831,9606.ENSP00000296424,9606.ENSP00000296456,9606.ENSP00000296577,9606.ENSP00000296674,9606.ENSP00000300648,9606.ENSP00000301457,9606.ENSP00000302896,9606.ENSP00000304592,9606.ENSP00000307188,9606.ENSP00000307241,9606.ENSP00000307567,9606.ENSP00000307889,9606.ENSP00000307940,9606.ENSP00000308275,9606.ENSP00000309334,9606.ENSP00000309830,9606.ENSP00000311430,9606.ENSP00000312311,9606.ENSP00000313007,9606.ENSP00000314508,9606.ENSP00000318646,9606.ENSP00000320309,9606.ENSP00000322419,9606.ENSP00000322439,9606.ENSP00000325448,9606.ENSP00000325548,9606.ENSP00000326381,9606.ENSP00000331901,9606.ENSP00000332604,9606.ENSP000003367	ASNS,MRPS34,DLD,TECR,SLC25A1,EIF3D,EIF5,MTHFD1,GSS,EIF3E,RPL18A,AIMP2,RPL19,GCLC,RPS12,RARS,EIF2AK2,SARS,ATIC,EIF3K,RPS16,EIF2S3,MRPL4,EIF3G,ACSS2,ACLY,EIF2S1,NARS,RPL35,AARS,EIF3J,MARS,RPL8,DARS,EIF4H,ACAT1,EIF2B2,RPS11,RPS27A,GGCT,RPS3,DLAT,FARSB,MRPL17,EIF5B,EIF4A1,BDH2,APEH,ABCE1,RPS23,GCN1L1,NDUFA7,RPS9,FASN,ASL,PDHB,QARS,RPL13,EEF2,MRPL38,RPL15,RPL38,RPL4,MRPL46,PABPC1,GBA,RPS15A,FARSA,RPLP2,TUFM,KARS,CNDP2,EIF4A2,EEF1G,EIF3C,EIF5A,LARP1,EEF1A1,RPS7,RPL7,RPS2,PPA2,RPS21,RPL3,RPL27A,RPL21,RPLP1,RPS17,RPS3A,RPSA,RPL22,WARS,RPS26,COPS5,EIF2B3,ETF1,EIF3B,RPL12,RPL39,IARS2,EPRS,RPS27,EIF3A,GCLM,RPL5,MCTS1,MRPS2,RPL7A,MRPL14,MRPS18A,ASS1,PABPC4,PPA1,YARS,EIF3I,RPS4X,RPL10A,EIF6,RPL11,EIF2S2,IARS,VARS,RRBP1,EEF1E1,PDHA1,RPS6,CARS,GART,GARS,RPL13A,EEF1B2,RPL24,LARS,RPL34,AIMP1,RPL14,RPS8,RPL23L,HAGH,LARP4,DDX3X,RPS14,RPL31,RBM4,TARS,EIF4B,RPL23A,RPS18,PPT1,EIF2B4,RPL17,GSPT1,RPL9,AARSD1,RPL6,EEF1D,RPL10,RPS24,EIF4G1,RPL32,EIF2A,RPL29,RPL35A,RPL23,ACSL1,EIF4E,HARS,GNB2L1,RPL30,RPS20,EIF3F,EIF4G2,RPS25,FAU,RPS13,RPL18,NACA,RPLP0,RPL28,RPL26,RPL36,RPL27,RPS19,RPS28,RPS5,OPLAH,RPS10,EIF3L

T = 72 h, IFNα7: CoV-2 / mock

#Term ID	Term Description	Genes Mapped	Enrichment Score	Direction	False Discovery Rate	Method	Matching Proteins in Your Input (IDs)	Matching Proteins in Your Input (Labels)			
GO:0006956	complement activation	5	6.85201	top	0.00016	afc	9606.ENSP00000225698,9606.ENSP00000245907,9606.ENSP00000315130,9606.ENSP00000356399,9606.ENSP00000416561	C1QBP,C3,CLU,CFH,CFB			
GO:0016266	O-glycan processing	7	5.98512	top	0.00016	afc	9606.ENSP00000287590,9606.ENSP00000321874,9606.ENSP00000360776,9606.ENSP00000381008,9606.ENSP00000417498,9606.ENSP00000436812,9606.ENSP00000484824	B3GNT7,B3GNT3,B4GALT5,MUC16,MUC4,MUC5B,MUC1			
GO:0030449	regulation of complement activation	7	5.56086	top	0.00016	afc	9606.ENSP00000225698,9606.ENSP00000245907,9606.ENSP00000315130,9606.ENSP00000356399,9606.ENSP00000379191,9606.ENSP00000416561,9606.ENSP00000479488	C1QBP,C3,CLU,CFH,CD59,CFB,PHB			
GO:2000257	regulation of protein activation cascade	7	5.56086	top	0.00016	afc	9606.ENSP00000225698,9606.ENSP00000245907,9606.ENSP00000315130,9606.ENSP00000356399,9606.ENSP00000379191,9606.ENSP00000416561,9606.ENSP00000479488	C1QBP,C3,CLU,CFH,CD59,CFB,PHB			
GO:0002920	regulation of humoral immune response	8	4.9906	top	0.00021	afc	9606.ENSP00000225698,9606.ENSP00000245907,9606.ENSP00000315130,9606.ENSP00000356399,9606.ENSP00000379191,9606.ENSP00000391592,9606.ENSP00000416561,9606.ENSP00000479488	C1QBP,C3,CLU,CFH,CD59,PTPN6,CFB,PHB			
GO:0006397	mRNA processing	160	0.395219	bottom	0.00021	ks	9606.ENSP00000215793,9606.ENSP00000215829,9606.ENSP00000216252,9606.ENSP00000216727,9606.ENSP00000217109,9606.ENSP00000221419,9606.ENSP00000225698,9606.ENSP00000225792,9606.ENSP00000227524,9606.ENSP00000228284,9606.ENSP00000229390,9606.ENSP00000238112,9606.ENSP00000240185,9606.ENSP00000242776,9606.ENSP00000243563,9606.ENSP00000244020,9606.ENSP00000246071,9606.ENSP00000246194,9606.ENSP00000249299,9606.ENSP00000253363,9606.ENSP00000254108,9606.ENSP00000254193,9606.ENSP00000254301,9606.ENSP00000258962,9606.ENSP00000261167,9606.ENSP00000261531,9606.ENSP00000261973,9606.ENSP00000262710,9606.ENSP00000263579,9606.ENSP00000263694,9606.ENSP00000265866,9606.ENSP00000265872,9606.ENSP00000266079,9606.ENSP00000266679,9606.ENSP00000269349,9606.ENSP00000276079,9606.ENSP00000282007,9606.ENSP00000283179,9606.ENSP00000291552,9606.ENSP00000293677,9606.ENSP00000297071,9606.ENSP00000300291,9606.ENSP00000300413,9606.ENSP00000301740,9606.ENSP00000302160,9606.ENSP00000304903,9606.ENSP00000305556,9606.ENSP00000305790,9606.ENSP00000307863,9606.ENSP00000310448,9606.ENSP00000310670,9606.ENSP00000310723,9606.ENSP00000311677,9606.ENSP00000313007,9606.ENSP00000313199,9606.ENSP00000313829,9606.ENSP00000316042,9606.ENSP00000317123,9606.ENSP00000318861,9606.ENSP00000319169,9606.ENSP00000319240,9606.ENSP00000324122,9606.ENSP00000325376,9606.ENSP00000325377,9606.ENSP00000325905,9606.ENSP00000326531,9606.ENSP00000326806,9606.ENSP00000328848,9606.ENSP00000335321,9606.ENSP00000336741,9606.ENSP00000336752,9606.ENSP00000337632,9606.ENSP00000340796,9606.ENSP00000341826,9606.ENSP00000342374,9606.ENSP00000343966,9606.ENSP00000346634,9606.ENSP00000346694,9606.ENSP00000347005,9606.ENSP00000348984,9606.ENSP00000349168,9606.ENSP00000349428,9606.ENSP00000349748,9606.ENSP00000352438,[illegible]	SF3A1,SNRPD3,PHF5A,PABPN1,CSTF1,HNRNPL,C1QBP,DDX5,PRPF19,SART3,SRSF9,CPSF3,TARDBP,DDX39A,SNRPA,SRSF6,SNRPB2,RALY,LSM8,RBM39,FUS,SNRPA1,LGALS3,SRSF1,WBP11,SNW1,RBM25,ACIN1,DCPS,SNRNP40,HNRNPH3,CCAR1,PRPF6,CPSF6,EIF4A3,NONO,ZC3H13,HNRNPU,U2AFBP,RAVER1,TRA2A,NUDT21,SNRPD1,SRRM2,LSM3,CD2BP2,PCBP1,SF3B3,U2AF2,SART1,CCAR2,DDX23,PPP1R8,PABPC1,HNRNPD,KHDRBS1,HNRNPA0,SNRNP200,SF3B2,PRMT5,MAGOHB,PRPF31,HNRNPM,WDR33,SRSF7,THOC6,NCBP2,RBM10,SF3B1,DHX15,FIP1L1,SARNP,ZNF326,HNRNPA1,SNRPD2,PSPC1,THRAP3,HNRNPA2B1,CGI-74,SON,HNRNPH1,PTBP1,SFPQ,PCBP2,CTNNBL1,CDC73,DHX9,RNASEL,ADAR,SYNCRIP,RBM15,AHCYL1,FMR1,RBMX,SRSF11,CDC5L,YBX1,HNRNPH2,CSTF2,SF3A3,SRSF3,PRPF4,HNRNPR,NCBP1,LSM2,HNRNPK,XRN2,SF1,POLR2B,DDX1,RNMT,HNRNPUL1,SRSF2,HNRNPA3,GTF2F1,DDX39B,DDX17,SMU1,KHSRP,NHP2L1,ELAVL1,PRPF40A,RBM4,HNRNPLL,EFTUD2,STRAP,POLDIP3,HNRNPF,SNRPE,PNPT1,ESRP1,SNRPB,PTBP3,DDX46,TRA2B,U2SURP,SRSF10,ALYREF,PLRG1,LUC7L3,HSPA8,CLNS1A,PUF60,CELF1,CHERP,HTATSF1,HNRNPC,SRSF5,NOL3,RNPS1,PRPF8,RBM8A,DDX42,LSM4,SUPT5H,SNRNP70,POLR2A,SRRT,SAP18,CPSF1			

GO:0000375	RNA splicing, via transesterification reactions	128	0.402529	bottom	0.00022	ks	9606.ENSP00000215793,9606.ENSP00000215829,9606.ENSP00000216252,9606.ENSP00000216727,9606.ENSP00000217109,9606.ENSP00000221419,9606.ENSP00000225792,9606.ENSP00000227524,9606.ENSP00000228284,9606.ENSP00000229390,9606.ENSP00000238112,9606.ENSP00000242776,9606.ENSP00000243563,9606.ENSP00000244020,9606.ENSP00000246071,9606.ENSP00000246194,9606.ENSP00000249299,9606.ENSP00000254108,9606.ENSP00000254193,9606.ENSP00000258962,9606.ENSP00000261167,9606.ENSP00000261531,9606.ENSP00000263579,9606.ENSP00000263694,9606.ENSP00000265866,9606.ENSP00000265872,9606.ENSP00000266079,9606.ENSP00000269349,9606.ENSP00000276079,9606.ENSP00000283179,9606.ENSP00000291552,9606.ENSP00000293677,9606.ENSP00000297071,9606.ENSP00000300291,9606.ENSP00000300413,9606.ENSP00000301740,9606.ENSP00000302160,9606.ENSP00000304903,9606.ENSP00000305556,9606.ENSP00000305790,9606.ENSP00000307863,9606.ENSP00000310448,9606.ENSP00000310723,9606.ENSP00000313007,9606.ENSP00000313199,9606.ENSP00000316042,9606.ENSP00000317123,9606.ENSP00000318861,9606.ENSP00000319169,9606.ENSP00000319240,9606.ENSP00000324122,9606.ENSP00000325376,9606.ENSP00000325377,9606.ENSP00000325905,9606.ENSP00000326806,9606.ENSP00000335321,9606.ENSP00000336741,9606.ENSP00000336752,9606.ENSP00000341826,9606.ENSP00000342374,9606.ENSP00000343966,9606.ENSP00000346694,9606.ENSP00000347005,9606.ENSP00000349168,9606.ENSP00000349428,9606.ENSP00000349748,9606.ENSP00000352438,9606.ENSP00000355050,9606.ENSP00000356520,9606.ENSP00000358635,9606.ENSP00000358799,9606.ENSP00000359645,9606.ENSP00000359988,9606.ENSP00000360532,9606.ENSP00000361626,9606.ENSP00000361927,9606.ENSP00000362063,9606.ENSP00000362110,9606.ENSP00000362820,9606.ENSP00000363313,9606.ENSP00000363745,9606.ENSP00000364289,9606.ENSP00000364813,9606.ENSP00000365439,[illegible]	SF3A1,SNRPD3,PHF5A,PABPN1,CSTF1,HNRNPL,DDX5,PRPF19,SART3,SRSF9,CPSF3,DDX39A,SNRPA,SRSF6,SNRPB2,RALY,LSM8,FUS,SNRPA1,SRSF1,WBP11,SNW1,DCPS,SNRNP40,HNRNPH3,CCAR1,PRPF6,EIF4A3,NONO,HNRNPU,U2AFBP,RAVER1,TRA2A,NUDT21,SNRPD1,SRRM2,LSM3,CD2BP2,PCBP1,SF3B3,U2AF2,SART1,DDX23,PABPC1,HNRNPD,HNRNPA0,SNRNP200,SF3B2,PRMT5,MAGOHB,PRPF31,HNRNPM,WDR33,SRSF7,NCBP2,SF3B1,DHX15,FIP1L1,HNRNPA1,SNRPD2,PSPC1,HNRNPA2B1,CGI-74,HNRNPH1,PTBP1,SFPQ,PCBP2,CTNNBL1,DHX9,SYNCRIP,RBM15,RBMX,SRSF11,CDC5L,YBX1,HNRNPH2,CSTF2,SF3A3,SRSF3,PRPF4,HNRNPR,NCBP1,LSM2,HNRNPK,SF1,POLR2B,DDX1,HNRNPUL1,SRSF2,HNRNPA3,GTF2F1,DDX39B,DDX17,SMU1,KHSRP,NHP2L1,ELAVL1,PRPF40A,EFTUD2,STRAP,HNRNPF,SNRPE,SNRPB,DDX46,TRA2B,U2SURP,SRSF10,ALYREF,PLRG1,LUC7L3,HSPA8,CLNS1A,PUF60,CELF1,CHERP,HTATSF1,HNRNPC,SRSF5,NOL3,RNPS1,PRPF8,RBM8A,DDX42,LSM4,SNRNP70,POLR2A,SRRT,CPSF1			
GO:0002673	regulation of acute inflammatory response	9	4.55841	top	0.00022	afc	9606.ENSP00000225698,9606.ENSP00000245907,9606.ENSP00000315130,9606.ENSP00000356399,9606.ENSP00000379191,9606.ENSP00000381607,9606.ENSP00000416561,9606.ENSP00000418770,9606.ENSP00000479488	C1QBP,C3,CLU,CFH,CD59,GSTP1,CFB,PARK7,PHB			
GO:0043604	amide biosynthetic process	197	0.392157	bottom	0.00022	ks	9606.ENSP00000175506,9606.ENSP00000177742,9606.ENSP00000199706,9606.ENSP00000205402,9606.ENSP00000215567,9606.ENSP00000215882,9606.ENSP00000216190,9606.ENSP00000216554,9606.ENSP00000216605,9606.ENSP00000216951,9606.ENSP00000220849,9606.ENSP00000222247,9606.ENSP00000223029,9606.ENSP00000225430,9606.ENSP00000229416,9606.ENSP00000230050,9606.ENSP00000231572,9606.ENSP00000233057,9606.ENSP00000234677,9606.ENSP00000236959,9606.ENSP00000248342,9606.ENSP00000251453,9606.ENSP00000253039,9606.ENSP00000253099,9606.ENSP00000253108,9606.ENSP00000253382,9606.ENSP00000253792,9606.ENSP00000254630,9606.ENSP00000256383,9606.ENSP00000256854,9606.ENSP00000259469,9606.ENSP00000261772,9606.ENSP00000261868,9606.ENSP00000262027,9606.ENSP00000262584,9606.ENSP00000264161,9606.ENSP00000264705,9606.ENSP00000265753,9606.ENSP00000265838,9606.ENSP00000266126,9606.ENSP00000270625,9606.ENSP00000272317,9606.ENSP00000274606,9606.ENSP00000275428,9606.ENSP00000278572,9606.ENSP00000280346,9606.ENSP00000280557,9606.ENSP00000281828,9606.ENSP00000288937,9606.ENSP00000289371,9606.ENSP00000293831,9606.ENSP00000296424,9606.ENSP00000296456,9606.ENSP00000296577,9606.ENSP00000296674,9606.ENSP00000296805,9606.ENSP00000300648,9606.ENSP00000301457,9606.ENSP00000302896,9606.ENSP00000304592,9606.ENSP00000306548,9606.ENSP00000307188,9606.ENSP00000307241,9606.ENSP00000307567,9606.ENSP00000307889,9606.ENSP00000307940,9606.ENSP00000308275,9606.ENSP00000309334,9606.ENSP00000309830,9606.ENSP00000311430,9606.ENSP00000312311,9606.ENSP00000313007,9606.ENSP00000314508,9606.ENSP00000318646,9606.ENSP00000320309,9606.ENSP00000322419,9606.ENSP00000322439,9606.ENSP00000325448,9606.ENSP00000325548,9606.ENSP00000326381,9606.ENSP00000331901,9606.ENSP00000332604,9606.ENSP00000336702,9606.ENSP00000336721,[illegible]	ASNS,MRPS34,MRPL28,DLD,TECR,SLC25A1,EIF3D,EIF5,MTHFD1,GSS,EIF3E,RPL18A,AIMP2,RPL19,GCLC,RPS12,RARS,EIF2AK2,SARS,ATIC,EIF3K,RPS16,EIF2S3,MRPL4,EIF3G,ACSS2,ACLY,PTCD3,EIF2S1,NARS,RPL35,AARS,EIF3J,MARS,RPL8,DARS,CAD,EIF4H,ACAT1,EIF2B2,RPS11,RPS27A,NHP2,GGCT,RPS3,DLAT,DENR,FARSB,MRPL17,EIF5B,EIF4A1,BDH2,APEH,ABCE1,RPS23,GFM2,GCN1L1,NDUFA7,RPS9,FASN,MRPL13,ASL,PDHB,QARS,RPL13,EEF2,MRPL38,RPL15,RPL38,RPL4,MRPL46,PABPC1,GBA,RPS15A,FARSA,RPLP2,TUFM,KARS,CNDP2,EIF4A2,EEF1G,EIF3C,EIF5A,LARP1,EEF1A1,RPS7,RPL7,RPS2,PPA2,RPS21,RPL3,RPL27A,RPL21,RPLP1,RPS17,RPS3A,RPSA,RPL22,WARS,RPS26,COPS5,EIF2B3,ETF1,EIF3B,RPL12,RPL39,IARS2,EPRS,RPS27,EIF3A,GCLM,RPL5,MCTS1,MRPS2,RPL7A,MRPL14,MRPS18A,ASS1,PABPC4,PPA1,YARS,EIF3I,RPS4X,RPL10A,EIF6,RPL11,EIF2S2,IARS,VARS,RRBP1,EEF1E1,PDHA1,RPS6,CARS,GART,GARS,RPL13A,EEF1B2,RPL24,LARS,RPL34,AIMP1,RPL14,RPS8,RPL23L,HAGH,LARP4,DDX3X,RPS14,RPL31,RBM4,TARS,EIF4B,RPL23A,RPS18,EIF2B4,RPL17,GSPT1,RPL9,AARSD1,RPL6,MRPL55,EEF1D,RPL10,RPS24,EIF4G1,RPL32,EIF2A,RPL29,RPL35A,EIF1,RPL23,ACSL1,EIF4E,HARS,GNB2L1,RPL30,RPS20,EIF3F,EIF4G2,RPS25,FAU,RPS13,EIF3M,RPL18,NACA,RPLP0,RPL28,RPL26,RPL36,RPL27,RPS19,RPS28,RPS5,OPLAH,RPS10,EIF3L			
GO:0072376	protein activation cascade	6	5.913	top	0.00022	afc	9606.ENSP00000225698,9606.ENSP00000245907,9606.ENSP00000315130,9606.ENSP00000356399,9606.ENSP00000377055,9606.ENSP00000416561	C1QBP,C3,CLU,CFH,PRCP,CFB			

GO:0000398	mRNA splicing, via spliceosome	127	0.399947	bottom	0.00029	ks	9606.ENSP00000215793,9606.ENSP00000215829,9606.ENSP00000216252,9606.ENSP00000216727,9606.ENSP00000217109,9606.ENSP00000221419,9606.ENSP00000225792,9606.ENSP00000227524,9606.ENSP00000228284,9606.ENSP00000229390,9606.ENSP00000238112,9606.ENSP00000242776,9606.ENSP00000243563,9606.ENSP00000244020,9606.ENSP00000246071,9606.ENSP00000246194,9606.ENSP00000249299,9606.ENSP00000254108,9606.ENSP00000254193,9606.ENSP00000258962,9606.ENSP00000261167,9606.ENSP00000261531,9606.ENSP00000263579,9606.ENSP00000263694,9606.ENSP00000265866,9606.ENSP00000265872,9606.ENSP00000266079,9606.ENSP00000269349,9606.ENSP00000276079,9606.ENSP00000283179,9606.ENSP00000291552,9606.ENSP00000293677,9606.ENSP00000297071,9606.ENSP00000300291,9606.ENSP00000300413,9606.ENSP00000301740,9606.ENSP00000302160,9606.ENSP00000304903,9606.ENSP00000305556,9606.ENSP00000305790,9606.ENSP00000307863,9606.ENSP00000310448,9606.ENSP00000310723,9606.ENSP00000313007,9606.ENSP00000313199,9606.ENSP00000316042,9606.ENSP00000317123,9606.ENSP00000318861,9606.ENSP00000319169,9606.ENSP00000319240,9606.ENSP00000324122,9606.ENSP00000325376,9606.ENSP00000325377,9606.ENSP00000325905,9606.ENSP00000326806,9606.ENSP00000335321,9606.ENSP00000336741,9606.ENSP00000336752,9606.ENSP00000341826,9606.ENSP00000342374,9606.ENSP00000343966,9606.ENSP00000346694,9606.ENSP00000347005,9606.ENSP00000349168,9606.ENSP00000349428,9606.ENSP00000349748,9606.ENSP00000352438,9606.ENSP00000355050,9606.ENSP00000356520,9606.ENSP00000358635,9606.ENSP00000358799,9606.ENSP00000359645,9606.ENSP00000359988,9606.ENSP00000360532,9606.ENSP00000361626,9606.ENSP00000361927,9606.ENSP00000362063,9606.ENSP00000362110,9606.ENSP00000362820,9606.ENSP00000363313,9606.ENSP00000363745,9606.ENSP00000364289,9606.ENSP00000364813,9606.ENSP00000365439,[illegible]	SF3A1,SNRPD3,PHF5A,PABPN1,CSTF1,HNRNPL,DDX5,PRPF19,SART3,SRSF9,CPSF3,DDX39A,SNRPA,SRSF6,SNRPB2,RALY,LSM8,FUS,SNRPA1,SRSF1,WBP11,SNW1,DCPS,SNRNP40,HNRNPH3,CCAR1,PRPF6,EIF4A3,NONO,HNRNPU,U2AFBP,RAVER1,TRA2A,NUDT21,SNRPD1,SRRM2,LSM3,CD2BP2,PCBP1,SF3B3,U2AF2,SART1,DDX23,PABPC1,HNRNPD,HNRNPA0,SNRNP200,SF3B2,PRMT5,MAGOHB,PRPF31,HNRNPM,WDR33,SRSF7,NCBP2,SF3B1,DHX15,FIP1L1,HNRNPA1,SNRPD2,PSPC1,HNRNPA2B1,CGI-74,HNRNPH1,PTBP1,SFPQ,PCBP2,CTNNBL1,DHX9,SYNCRIP,RBM15,RBMX,SRSF11,CDC5L,YBX1,HNRNPH2,CSTF2,SF3A3,SRSF3,PRPF4,HNRNPR,NCBP1,LSM2,HNRNPK,SF1,POLR2B,DDX1,HNRNPUL1,SRSF2,HNRNPA3,GTF2F1,DDX39B,DDX17,SMU1,NHP2L1,ELAVL1,PRPF40A,EFTUD2,STRAP,HNRNPF,SNRPE,SNRPB,DDX46,TRA2B,U2SURP,SRSF10,ALYREF,PLRG1,LUC7L3,HSPA8,CLNS1A,PUF60,CELF1,CHERP,HTATSF1,HNRNPC,SRSF5,NOL3,RNPS1,PRPF8,RBM8A,DDX42,LSM4,SNRNP70,POLR2A,SRRT,CPSF1			
GO:0008380	RNA splicing	152	0.368846	bottom	0.00032	ks	9606.ENSP00000215793,9606.ENSP00000215829,9606.ENSP00000216038,9606.ENSP00000216252,9606.ENSP00000216727,9606.ENSP00000217109,9606.ENSP00000221419,9606.ENSP00000225698,9606.ENSP00000225792,9606.ENSP00000227524,9606.ENSP00000228284,9606.ENSP00000229390,9606.ENSP00000238112,9606.ENSP00000240185,9606.ENSP00000242776,9606.ENSP00000243563,9606.ENSP00000244020,9606.ENSP00000246071,9606.ENSP00000246194,9606.ENSP00000249299,9606.ENSP00000253363,9606.ENSP00000254108,9606.ENSP00000254193,9606.ENSP00000254301,9606.ENSP00000258962,9606.ENSP00000261167,9606.ENSP00000261531,9606.ENSP00000261700,9606.ENSP00000261973,9606.ENSP00000262710,9606.ENSP00000263579,9606.ENSP00000263694,9606.ENSP00000265866,9606.ENSP00000265872,9606.ENSP00000266079,9606.ENSP00000269349,9606.ENSP00000276079,9606.ENSP00000282007,9606.ENSP00000283179,9606.ENSP00000291552,9606.ENSP00000293677,9606.ENSP00000297071,9606.ENSP00000300291,9606.ENSP00000300413,9606.ENSP00000301740,9606.ENSP00000302160,9606.ENSP00000304903,9606.ENSP00000305556,9606.ENSP00000305790,9606.ENSP00000306117,9606.ENSP00000307863,9606.ENSP00000310448,9606.ENSP00000310670,9606.ENSP00000310723,9606.ENSP00000311677,9606.ENSP00000313007,9606.ENSP00000313199,9606.ENSP00000316042,9606.ENSP00000317123,9606.ENSP00000318861,9606.ENSP00000319169,9606.ENSP00000319240,9606.ENSP00000324122,9606.ENSP00000324804,9606.ENSP00000325376,9606.ENSP00000325377,9606.ENSP00000325905,9606.ENSP00000326531,9606.ENSP00000326806,9606.ENSP00000328848,9606.ENSP00000335321,9606.ENSP00000336741,9606.ENSP00000336752,9606.ENSP00000340796,9606.ENSP00000341826,9606.ENSP00000342374,9606.ENSP00000343966,9606.ENSP00000346634,9606.ENSP00000346694,9606.ENSP00000347005,9606.ENSP00000348984,9606.ENSP00000349168,9606.ENSP00000349428,9606.ENSP00000349748,[illegible]	SF3A1,SNRPD3,RTCB,PHF5A,PABPN1,CSTF1,HNRNPL,C1QBP,DDX5,PRPF19,SART3,SRSF9,CPSF3,TARDBP,DDX39A,SNRPA,SRSF6,SNRPB2,RALY,LSM8,RBM39,FUS,SNRPA1,LGALS3,SRSF1,WBP11,SNW1,C14orf166,RBM25,ACIN1,DCPS,SNRNP40,HNRNPH3,CCAR1,PRPF6,EIF4A3,NONO,ZC3H13,HNRNPU,U2AFBP,RAVER1,TRA2A,NUDT21,SNRPD1,SRRM2,LSM3,CD2BP2,PCBP1,SF3B3,DDX19A,U2AF2,SART1,CCAR2,DDX23,PPP1R8,PABPC1,HNRNPD,HNRNPA0,SNRNP200,SF3B2,PRMT5,MAGOHB,PRPF31,PPP2R1A,HNRNPM,WDR33,SRSF7,THOC6,NCBP2,RBM10,SF3B1,DHX15,FIP1L1,ZNF326,HNRNPA1,SNRPD2,PSPC1,THRAP3,HNRNPA2B1,CGI-74,SON,HNRNPH1,PTBP1,SFPQ,PCBP2,CTNNBL1,DHX9,SYNCRIP,RBM15,FMR1,RBMX,SRSF11,CDC5L,YBX1,HNRNPH2,CSTF2,SF3A3,SRSF3,PRPF4,HNRNPR,NCBP1,LSM2,HNRNPK,SF1,POLR2B,DDX1,HNRNPUL1,SRSF2,HNRNPA3,GTF2F1,DDX39B,DDX17,SMU1,KHSRP,NHP2L1,ELAVL1,PRPF40A,RBM4,EFTUD2,STRAP,HNRNPF,SNRPE,ESRP1,SNRPB,PTBP3,DDX46,TRA2B,PPP2CA,U2SURP,SRSF10,ALYREF,PLRG1,LUC7L3,HSPA8,CLNS1A,PUF60,CELF1,CHERP,HTATSF1,HNRNPC,SRSF5,NOL3,RNPS1,PRPF8,RBM8A,DDX42,LSM4,SNRNP70,POLR2A,SRRT,SAP18,CPSF1			
GO:0006493	protein O-linked glycosylation	10	4.11632	top	0.0004	afc	9606.ENSP00000287590,9606.ENSP00000321874,9606.ENSP00000360776,9606.ENSP00000362824,9606.ENSP00000373206,9606.ENSP00000381008,9606.ENSP00000417498,9606.ENSP00000425809,9606.ENSP00000436812,9606.ENSP00000484824	B3GNT7,B3GNT3,B4GALT5,OGT,EOGT,MUC16,MUC4,PGM3,MUC5B,MUC1			

GO:0043043	peptide biosynthetic process	178	0.392472	bottom	0.0006	ks	9606.ENSP00000177742,9606.ENSP00000199706,9606.ENSP00000216190,9606.ENSP00000216554,9606.ENSP00000216951,9606.ENSP00000220849,9606.ENSP00000222247,9606.ENSP00000223029,9606.ENSP00000225430,9606.ENSP00000229416,9606.ENSP00000230050,9606.ENSP00000231572,9606.ENSP00000233057,9606.ENSP00000234677,9606.ENSP00000248342,9606.ENSP00000251453,9606.ENSP00000253039,9606.ENSP00000253099,9606.ENSP00000253108,9606.ENSP00000254630,9606.ENSP00000256383,9606.ENSP00000256854,9606.ENSP00000259469,9606.ENSP00000261772,9606.ENSP00000261868,9606.ENSP00000262027,9606.ENSP00000262584,9606.ENSP00000264161,9606.ENSP00000265753,9606.ENSP00000266126,9606.ENSP00000270625,9606.ENSP00000272317,9606.ENSP00000274606,9606.ENSP00000275428,9606.ENSP00000278572,9606.ENSP00000280557,9606.ENSP00000281828,9606.ENSP00000288937,9606.ENSP00000289371,9606.ENSP00000293831,9606.ENSP00000296424,9606.ENSP00000296456,9606.ENSP00000296577,9606.ENSP00000296674,9606.ENSP00000296805,9606.ENSP00000300648,9606.ENSP00000301457,9606.ENSP00000302896,9606.ENSP00000306548,9606.ENSP00000307567,9606.ENSP00000307889,9606.ENSP00000307940,9606.ENSP00000308275,9606.ENSP00000309334,9606.ENSP00000309830,9606.ENSP00000311430,9606.ENSP00000312311,9606.ENSP00000313007,9606.ENSP00000318646,9606.ENSP00000320309,9606.ENSP00000322419,9606.ENSP00000322439,9606.ENSP00000325448,9606.ENSP00000325548,9606.ENSP00000326381,9606.ENSP00000331901,9606.ENSP00000332604,9606.ENSP00000336702,9606.ENSP00000336721,9606.ENSP00000339063,9606.ENSP00000339095,9606.ENSP00000339795,9606.ENSP00000341885,9606.ENSP00000343885,9606.ENSP00000345957,9606.ENSP00000346001,9606.ENSP00000346015,9606.ENSP00000346027,9606.ENSP00000346037,9606.ENSP00000346046,9606.ENSP00000346050,9606.ENSP00000346067,9606.ENSP00000346088,9606.ENSP00000347495,[illegible]	MRPS34,MRPL28,EIF3D,EIF5,GSS,EIF3E,RPL18A,AIMP2,RPL19,GCLC,RPS12,RARS,EIF2AK2,SARS,EIF3K,RPS16,EIF2S3,MRPL4,EIF3G,PTCD3,EIF2S1,NARS,RPL35,AARS,EIF3J,MARS,RPL8,DARS,EIF4H,EIF2B2,RPS11,RPS27A,NHP2,GGCT,RPS3,DENR,FARSB,MRPL17,EIF5B,EIF4A1,BDH2,APEH,ABCE1,RPS23,GFM2,GCN1L1,NDUFA7,RPS9,MRPL13,QARS,RPL13,EEF2,MRPL38,RPL15,RPL38,RPL4,MRPL46,PABPC1,RPS15A,FARSA,RPLP2,TUFM,KARS,CNDP2,EIF4A2,EEF1G,EIF3C,EIF5A,LARP1,EEF1A1,RPS7,RPL7,RPS2,PPA2,RPS21,RPL3,RPL27A,RPL21,RPLP1,RPS17,RPS3A,RPSA,RPL22,WARS,RPS26,COPS5,EIF2B3,ETF1,EIF3B,RPL12,RPL39,IARS2,EPRS,RPS27,EIF3A,GCLM,RPL5,MCTS1,MRPS2,RPL7A,MRPL14,MRPS18A,PABPC4,PPA1,YARS,EIF3I,RPS4X,RPL10A,EIF6,RPL11,EIF2S2,IARS,VARS,RRBP1,EEF1E1,RPS6,CARS,GARS,RPL13A,EEF1B2,RPL24,LARS,RPL34,AIMP1,RPL14,RPS8,RPL23L,HAGH,LARP4,DDX3X,RPS14,RPL31,RBM4,TARS,EIF4B,RPL23A,RPS18,EIF2B4,RPL17,GSPT1,RPL9,AARSD1,RPL6,MRPL55,EEF1D,RPL10,RPS24,EIF4G1,RPL32,EIF2A,RPL29,RPL35A,EIF1,RPL23,EIF4E,HARS,GNB2L1,RPL30,RPS20,EIF3F,EIF4G2,RPS25,FAU,RPS13,EIF3M,RPL18,NACA,RPLP0,RPL28,RPL26,RPL36,RPL27,RPS19,RPS28,RPS5,OPLAH,RPS10,EIF3L			
GO:0006957	complement activation, alternative pathway	3	8.45774	top	0.00065	afc	9606.ENSP00000245907,9606.ENSP00000356399,9606.ENSP00000416561	C3,CFH,CFB			
GO:0006412	translation	170	0.409808	bottom	0.0011	ks	9606.ENSP00000177742,9606.ENSP00000199706,9606.ENSP00000216190,9606.ENSP00000216554,9606.ENSP00000220849,9606.ENSP00000222247,9606.ENSP00000223029,9606.ENSP00000225430,9606.ENSP00000230050,9606.ENSP00000231572,9606.ENSP00000233057,9606.ENSP00000234677,9606.ENSP00000248342,9606.ENSP00000251453,9606.ENSP00000253039,9606.ENSP00000253099,9606.ENSP00000253108,9606.ENSP00000254630,9606.ENSP00000256383,9606.ENSP00000256854,9606.ENSP00000259469,9606.ENSP00000261772,9606.ENSP00000261868,9606.ENSP00000262027,9606.ENSP00000262584,9606.ENSP00000264161,9606.ENSP00000265753,9606.ENSP00000266126,9606.ENSP00000270625,9606.ENSP00000272317,9606.ENSP00000274606,9606.ENSP00000278572,9606.ENSP00000280557,9606.ENSP00000281828,9606.ENSP00000288937,9606.ENSP00000289371,9606.ENSP00000293831,9606.ENSP00000296456,9606.ENSP00000296577,9606.ENSP00000296674,9606.ENSP00000296805,9606.ENSP00000300648,9606.ENSP00000301457,9606.ENSP00000302896,9606.ENSP00000306548,9606.ENSP00000307567,9606.ENSP00000307889,9606.ENSP00000307940,9606.ENSP00000308275,9606.ENSP00000309334,9606.ENSP00000309830,9606.ENSP00000311430,9606.ENSP00000312311,9606.ENSP00000313007,9606.ENSP00000318646,9606.ENSP00000320309,9606.ENSP00000322419,9606.ENSP00000322439,9606.ENSP00000325448,9606.ENSP00000326381,9606.ENSP00000331901,9606.ENSP00000332604,9606.ENSP00000336702,9606.ENSP00000336721,9606.ENSP00000339063,9606.ENSP00000339095,9606.ENSP00000339795,9606.ENSP00000341885,9606.ENSP00000343885,9606.ENSP00000345957,9606.ENSP00000346001,9606.ENSP00000346015,9606.ENSP00000346027,9606.ENSP00000346037,9606.ENSP00000346046,9606.ENSP00000346050,9606.ENSP00000346067,9606.ENSP00000346088,9606.ENSP00000347495,9606.ENSP00000348849,9606.ENSP00000350512,9606.ENSP00000353575,9606.ENSP00000353741,9606.ENSP00000354125,[illegible]	MRPS34,MRPL28,EIF3D,EIF5,EIF3E,RPL18A,AIMP2,RPL19,RPS12,RARS,EIF2AK2,SARS,EIF3K,RPS16,EIF2S3,MRPL4,EIF3G,PTCD3,EIF2S1,NARS,RPL35,AARS,EIF3J,MARS,RPL8,DARS,EIF4H,EIF2B2,RPS11,RPS27A,NHP2,RPS3,DENR,FARSB,MRPL17,EIF5B,EIF4A1,APEH,ABCE1,RPS23,GFM2,GCN1L1,NDUFA7,RPS9,MRPL13,QARS,RPL13,EEF2,MRPL38,RPL15,RPL38,RPL4,MRPL46,PABPC1,RPS15A,FARSA,RPLP2,TUFM,KARS,EIF4A2,EEF1G,EIF3C,EIF5A,LARP1,EEF1A1,RPS7,RPL7,RPS2,PPA2,RPS21,RPL3,RPL27A,RPL21,RPLP1,RPS17,RPS3A,RPSA,RPL22,WARS,RPS26,COPS5,EIF2B3,ETF1,EIF3B,RPL12,RPL39,IARS2,EPRS,RPS27,EIF3A,RPL5,MCTS1,MRPS2,RPL7A,MRPL14,MRPS18A,PABPC4,PPA1,YARS,EIF3I,RPS4X,RPL10A,EIF6,RPL11,EIF2S2,IARS,VARS,RRBP1,EEF1E1,RPS6,CARS,GARS,RPL13A,EEF1B2,RPL24,LARS,RPL34,AIMP1,RPL14,RPS8,RPL23L,LARP4,DDX3X,RPS14,RPL31,RBM4,TARS,EIF4B,RPL23A,RPS18,EIF2B4,RPL17,GSPT1,RPL9,AARSD1,RPL6,MRPL55,EEF1D,RPL10,RPS24,EIF4G1,RPL32,EIF2A,RPL29,RPL35A,EIF1,RPL23,EIF4E,HARS,GNB2L1,RPL30,RPS20,EIF3F,EIF4G2,RPS25,FAU,RPS13,EIF3M,RPL18,NACA,RPLP0,RPL28,RPL26,RPL36,RPL27,RPS19,RPS28,RPS5,RPS10,EIF3L			

GO:0000184	nuclear-transcribed mRNA catabolic process, nonsense-mediated decay	88	0.607222	bottom	0.0021	ks	9606.ENSP00000220849,9606.ENSP00000222247,9606.ENSP00000225430,9606.ENSP00000230050,9606.ENSP00000251453,9606.ENSP00000259469,9606.ENSP00000262584,9606.ENSP00000269349,9606.ENSP00000270625,9606.ENSP00000272317,9606.ENSP00000278572,9606.ENSP00000296674,9606.ENSP00000302896,9606.ENSP00000307889,9606.ENSP00000309334,9606.ENSP00000309830,9606.ENSP00000311430,9606.ENSP00000313007,9606.ENSP00000318646,9606.ENSP00000319240,9606.ENSP00000322419,9606.ENSP00000324804,9606.ENSP00000325074,9606.ENSP00000326806,9606.ENSP00000339095,9606.ENSP00000339795,9606.ENSP00000341885,9606.ENSP00000345957,9606.ENSP00000346001,9606.ENSP00000346015,9606.ENSP00000346027,9606.ENSP00000346037,9606.ENSP00000346046,9606.ENSP00000346050,9606.ENSP00000346067,9606.ENSP00000346088,9606.ENSP00000348849,9606.ENSP00000353741,9606.ENSP00000354739,9606.ENSP00000355315,9606.ENSP00000357555,9606.ENSP00000359345,9606.ENSP00000361076,9606.ENSP00000362744,9606.ENSP00000363018,9606.ENSP00000363676,9606.ENSP00000364289,9606.ENSP00000364543,9606.ENSP00000369757,9606.ENSP00000375730,9606.ENSP00000377640,9606.ENSP00000378163,9606.ENSP00000379506,9606.ENSP00000379888,9606.ENSP00000385958,9606.ENSP00000386717,9606.ENSP00000389103,9606.ENSP00000393241,9606.ENSP00000397798,9606.ENSP00000398131,9606.ENSP00000400467,9606.ENSP00000403172,9606.ENSP00000413436,9606.ENSP00000414321,9606.ENSP00000416255,9606.ENSP00000416429,9606.ENSP00000418447,9606.ENSP00000418868,9606.ENSP00000419117,9606.ENSP00000420311,9606.ENSP00000428085,9606.ENSP00000429374,9606.ENSP00000435096,9606.ENSP00000435370,9606.ENSP00000435777,9606.ENSP00000447001,9606.ENSP00000449328,9606.ENSP00000452909,9606.ENSP00000457723,9606.ENSP00000463058,9606.ENSP00000463784,9606.ENSP00000464342,9606.ENSP00000464813,9606.ENSP00000470142,[illegible]	EIF3E,RPL18A,RPL19,RPS12,RPS16,RPL35,RPL8,EIF4A3,RPS11,RPS27A,RPS3,RPS23,RPS9,RPL13,RPL15,RPL38,RPL4,PABPC1,RPS15A,MAGOHB,RPLP2,PPP2R1A,PPP2R2A,NCBP2,RPS7,RPL7,RPS2,RPS21,RPL3,RPL27A,RPL21,RPLP1,RPS17,RPS3A,RPSA,RPL22,RPS26,ETF1,RPL12,RPL39,RPS27,RPL5,RPL7A,RPS4X,RPL10A,RPL11,NCBP1,SKIV2L,RPS6,RPL13A,RPL24,RPL34,RPL14,RPS8,RPS14,RPL31,RPL23A,RPS18,RPL17,GSPT1,RPL9,RPL6,RPL10,RPS24,EIF4G1,RPL32,PPP2CA,RPL29,RPL35A,RPL23,RPL30,RPS20,RPS25,FAU,RPS13,RPL18,RPLP0,RPL28,RNPS1,RBM8A,RPL26,RPL36,RPL27,UPF1,RPS19,RPS28,RPS5,RPS10			
GO:0070613	regulation of protein processing	14	2.89355	top	0.0046	afc	9606.ENSP00000216181,9606.ENSP00000217131,9606.ENSP00000225698,9606.ENSP00000234590,9606.ENSP00000245907,9606.ENSP00000315130,9606.ENSP00000356399,9606.ENSP00000362924,9606.ENSP00000379157,9606.ENSP00000379191,9606.ENSP00000416561,9606.ENSP00000457243,9606.ENSP00000479488,9606.ENSP00000481314	MYH9,CTSZ,C1QBP,ENO1,C3,CLU,CFH,GSN,CAST,CD59,CFB,NOL3,PHB,SNX12			
GO:0034660	ncRNA metabolic process	100	0.473094	bottom	0.005	ks	9606.ENSP00000168216,9606.ENSP00000206542,9606.ENSP00000216038,9606.ENSP00000221801,9606.ENSP00000223029,9606.ENSP00000231572,9606.ENSP00000234677,9606.ENSP00000244051,9606.ENSP00000246868,9606.ENSP00000251453,9606.ENSP00000256854,9606.ENSP00000259469,9606.ENSP00000261167,9606.ENSP00000261601,9606.ENSP00000261700,9606.ENSP00000261772,9606.ENSP00000262027,9606.ENSP00000262160,9606.ENSP00000264161,9606.ENSP00000264279,9606.ENSP00000264670,9606.ENSP00000269349,9606.ENSP00000274606,9606.ENSP00000281828,9606.ENSP00000301396,9606.ENSP00000302886,9606.ENSP00000302896,9606.ENSP00000306565,9606.ENSP00000307567,9606.ENSP00000310448,9606.ENSP00000318176,9606.ENSP00000320309,9606.ENSP00000325448,9606.ENSP00000326806,9606.ENSP00000339095,9606.ENSP00000339795,9606.ENSP00000341885,9606.ENSP00000343885,9606.ENSP00000345957,9606.ENSP00000346046,9606.ENSP00000346067,9606.ENSP00000346694,9606.ENSP00000347495,9606.ENSP00000349541,9606.ENSP00000355889,9606.ENSP00000355890,9606.ENSP00000356476,9606.ENSP00000356530,9606.ENSP00000357459,9606.ENSP00000357555,9606.ENSP00000358563,9606.ENSP00000359345,9606.ENSP00000361076,9606.ENSP00000361186,9606.ENSP00000362329,9606.ENSP00000362532,9606.ENSP00000362576,9606.ENSP00000363018,9606.ENSP00000363574,9606.ENSP00000363676,9606.ENSP00000364289,9606.ENSP00000364794,9606.ENSP00000364815,9606.ENSP00000365522,9606.ENSP00000366396,9606.ENSP00000366934,9606.ENSP00000369038,9606.ENSP00000369757,9606.ENSP00000369897,9606.ENSP00000370589,9606.ENSP00000370625,9606.ENSP00000370745,9606.ENSP00000373918,9606.ENSP00000377954,9606.ENSP00000377969,9606.ENSP00000378191,9606.ENSP00000379506,9606.ENSP00000379888,9606.ENSP00000380033,9606.ENSP00000381216,9606.ENSP00000383949,9606.ENSP00000385958,9606.ENSP00000386636,9606.ENSP00000387710,[illegible]	HSD17B10,OSGEP,RTCB,FBL,AIMP2,RARS,SARS,MOCS3,SBDS,RPS16,NARS,RPL35,WBP11,USP14,C14orf166,AARS,MARS,SMAD2,DARS,NOP58,NSUN2,EIF4A3,NHP2,FARSB,PELP1,PA2G4,RPS9,ISG20,QARS,SART1,PRKRA,FARSA,KARS,NCBP2,RPS7,RPL7,RPS2,PPA2,RPS21,RPS17,RPSA,HNRNPA2B1,WARS,PTRF,IARS2,EPRS,TRMT1L,RNASEL,ADAR,RPS27,DKC1,RPL5,RPL7A,TP53RK,PPA1,RPRD1B,YARS,RPL10A,EIF6,RPL11,NCBP1,IARS,VARS,C9orf64,XRN2,NOL9,EEF1E1,RPS6,CARS,NOP56,POLR2B,DDX1,GARS,LARS,GTF2F1,AIMP1,RPL14,RPS8,DDX17,KHSRP,NHP2L1,RPS14,SSB,TARS,EXOSC6,AARSD1,EBNA1BP2,RPS24,CDKN2A,RPL35A,NUDT16,HARS,RAN,RPL26,RPL27,RPS19,RPS28,WDR18,POLR2A,SRRT			

GO:0043062	extracellular structure organization	34	1.41803	top	0.0053	ks	9606.ENSP00000007722,9606.ENSP00000200181,9606.ENSP00000218388,9606.ENSP00000260408,9606.ENSP00000261769,9606.ENSP00000264144,9606.ENSP00000265131,9606.ENSP00000279441,9606.ENSP00000288235,9606.ENSP00000296585,9606.ENSP00000324532,9606.ENSP00000325941,9606.ENSP00000327801,9606.ENSP00000342098,9606.ENSP00000345344,9606.ENSP00000346032,9606.ENSP00000349436,9606.ENSP00000352673,9606.ENSP00000355361,9606.ENSP00000356954,9606.ENSP00000357980,9606.ENSP00000357981,9606.ENSP00000358983,9606.ENSP00000361405,9606.ENSP00000363827,9606.ENSP00000365569,9606.ENSP00000368646,9606.ENSP00000368678,9606.ENSP00000372221,9606.ENSP00000375778,9606.ENSP00000386896,9606.ENSP00000416330,9606.ENSP00000417138,9606.ENSP00000434412	ITGA3,ITGB4,TIMP1,ADAM10,CDH1,LAMC2,TNC,MMP10,MYO1E,ITGA2,LAMA3,RIC8A,P4HB,SPINT1,CTSL,ANXA2,ADAM15,ELF3,CD47,CTGF,HTRA1,CTSS,NFKB2,MMP9,HSPG2,FLOT1,PRDX4,AGRN,SERPINB5,LAMB3,ITGA6,TGFBI,PLTP,SERPINH1			
GO:0006413	translational initiation	110	0.529674	bottom	0.0063	ks	9606.ENSP00000216190,9606.ENSP00000216554,9606.ENSP00000220849,9606.ENSP00000222247,9606.ENSP00000225430,9606.ENSP00000230050,9606.ENSP00000248342,9606.ENSP00000251453,9606.ENSP00000253039,9606.ENSP00000253108,9606.ENSP00000256383,9606.ENSP00000259469,9606.ENSP00000261868,9606.ENSP00000262584,9606.ENSP00000265753,9606.ENSP00000266126,9606.ENSP00000270625,9606.ENSP00000272317,9606.ENSP00000278572,9606.ENSP00000280557,9606.ENSP00000289371,9606.ENSP00000293831,9606.ENSP00000296577,9606.ENSP00000296674,9606.ENSP00000302896,9606.ENSP00000307889,9606.ENSP00000309334,9606.ENSP00000309830,9606.ENSP00000311430,9606.ENSP00000313007,9606.ENSP00000318646,9606.ENSP00000322419,9606.ENSP00000326381,9606.ENSP00000332604,9606.ENSP00000336721,9606.ENSP00000339095,9606.ENSP00000339795,9606.ENSP00000341885,9606.ENSP00000345957,9606.ENSP00000346001,9606.ENSP00000346015,9606.ENSP00000346027,9606.ENSP00000346037,9606.ENSP00000346046,9606.ENSP00000346050,9606.ENSP00000346067,9606.ENSP00000346088,9606.ENSP00000348849,9606.ENSP00000350512,9606.ENSP00000353575,9606.ENSP00000354125,9606.ENSP00000354739,9606.ENSP00000355315,9606.ENSP00000357555,9606.ENSP00000358140,9606.ENSP00000359345,9606.ENSP00000360365,9606.ENSP00000361076,9606.ENSP00000362688,9606.ENSP00000362744,9606.ENSP00000363018,9606.ENSP00000363574,9606.ENSP00000363676,9606.ENSP00000364119,9606.ENSP00000369757,9606.ENSP00000375730,9606.ENSP00000377640,9606.ENSP00000378163,9606.ENSP00000379506,9606.ENSP00000379888,9606.ENSP00000382840,9606.ENSP00000385958,9606.ENSP00000386717,9606.ENSP00000386894,9606.ENSP00000388806,9606.ENSP00000389103,9606.ENSP00000393241,9606.ENSP00000394869,9606.ENSP00000397798,9606.ENSP00000400467,9606.ENSP00000403172,9606.ENSP00000413436,9606.ENSP00000414321,9606.ENSP00000416255,[illegible]	EIF3D,EIF5,EIF3E,RPL18A,RPL19,RPS12,EIF3K,RPS16,EIF2S3,EIF3G,EIF2S1,RPL35,EIF3J,RPL8,EIF4H,EIF2B2,RPS11,RPS27A,RPS3,DENR,EIF5B,EIF4A1,ABCE1,RPS23,RPS9,RPL13,RPL15,RPL38,RPL4,PABPC1,RPS15A,RPLP2,EIF4A2,EIF3C,LARP1,RPS7,RPL7,RPS2,RPS21,RPL3,RPL27A,RPL21,RPLP1,RPS17,RPS3A,RPSA,RPL22,RPS26,COPS5,EIF2B3,EIF3B,RPL12,RPL39,RPS27,EIF3A,RPL5,MCTS1,RPL7A,EIF3I,RPS4X,RPL10A,EIF6,RPL11,EIF2S2,RPS6,RPL13A,RPL24,RPL34,RPL14,RPS8,DDX3X,RPS14,RPL31,RBM4,EIF4B,RPL23A,RPS18,EIF2B4,RPL17,RPL9,RPL6,RPL10,RPS24,EIF4G1,RPL32,EIF2A,RPL29,RPL35A,EIF1,RPL23,EIF4E,RPL30,RPS20,EIF3F,EIF4G2,RPS25,FAU,RPS13,EIF3M,RPL18,RPLP0,RPL28,RPL26,RPL36,RPL27,RPS19,RPS28,RPS5,RPS10,EIF3L			
GO:0042060	wound healing	62	1.19471	top	0.0065	ks	9606.ENSP00000078429,9606.ENSP00000161559,9606.ENSP00000215832,9606.ENSP00000218388,9606.ENSP00000220763,9606.ENSP00000220809,9606.ENSP00000225698,9606.ENSP00000229795,9606.ENSP00000255194,9606.ENSP00000262160,9606.ENSP00000263025,9606.ENSP00000263168,9606.ENSP00000263277,9606.ENSP00000265131,9606.ENSP00000265563,9606.ENSP00000278568,9606.ENSP00000286548,9606.ENSP00000296511,9606.ENSP00000296585,9606.ENSP00000311032,9606.ENSP00000320171,9606.ENSP00000328405,9606.ENSP00000348461,9606.ENSP00000349436,9606.ENSP00000352208,9606.ENSP00000352252,9606.ENSP00000353590,9606.ENSP00000354573,9606.ENSP00000354947,9606.ENSP00000357722,9606.ENSP00000358866,9606.ENSP00000360141,9606.ENSP00000361850,9606.ENSP00000361949,9606.ENSP00000362924,9606.ENSP00000366109,9606.ENSP00000369129,9606.ENSP00000370542,9606.ENSP00000371958,9606.ENSP00000376475,9606.ENSP00000376506,9606.ENSP00000376800,9606.ENSP00000377055,9606.ENSP00000377471,9606.ENSP00000379191,9606.ENSP00000379287,9606.ENSP00000383118,9606.ENSP00000391592,9606.ENSP00000393840,9606.ENSP00000400175,9606.ENSP00000400717,9606.ENSP00000401010,9606.ENSP00000401645,9606.ENSP00000407375,9606.ENSP00000428924,9606.ENSP00000430432,9606.ENSP00000448035,9606.ENSP00000458832,9606.ENSP00000478026,9606.ENSP00000479153,9606.ENSP00000481878,9606.ENSP00000484472	GNA11,CEACAM1,MAPK1,TIMP1,CPQ,PLAT,C1QBP,MAPK14,AP3B1,SMAD2,MAPK3,CAPZA1,EHD2,TNC,PRKAR2A,PAK1,GNAQ,ANXA5,ITGA2,CASP3,PKM,DGKA,RAC1,ADAM15,MYOF,HIST1H3J,MYH10,MACF1,CAPZA2,S100A8,FLNA,GNAS,PLAU,PABPC4,GSN,ANXA1,DSP,SDC1,CD9,PRKAR1A,PDCD10,MTPN,PRCP,CORO1B,CD59,YWHAZ,CDC42,PTPN6,BLOC1S3,RHOA,GNA13,CAPZB,SERPINB2,GPX1,LYN,HDAC2,NACA,ALOX15,ANXA8,EHD1,GNB1,EPPK1			

GO:0090150	establishment of protein localization to membrane	104	0.467158	bottom	0.0099	ks	9606.ENSP00000054666,9606.ENSP00000216034,9606.ENSP00000217402,9606.ENSP00000222247,9606.ENSP00000225430,9606.ENSP00000230050,9606.ENSP00000251453,9606.ENSP00000253861,9606.ENSP00000259469,9606.ENSP00000260130,9606.ENSP00000262584,9606.ENSP00000264710,9606.ENSP00000267884,9606.ENSP00000269228,9606.ENSP00000270625,9606.ENSP00000272317,9606.ENSP00000278572,9606.ENSP00000280772,9606.ENSP00000293288,9606.ENSP00000295797,9606.ENSP00000296674,9606.ENSP00000299138,9606.ENSP00000302896,9606.ENSP00000302961,9606.ENSP00000307889,9606.ENSP00000309334,9606.ENSP00000309830,9606.ENSP00000311430,9606.ENSP00000312066,9606.ENSP00000318646,9606.ENSP00000318822,9606.ENSP00000322419,9606.ENSP00000333547,9606.ENSP00000335153,9606.ENSP00000339095,9606.ENSP00000339795,9606.ENSP00000341885,9606.ENSP00000342181,9606.ENSP00000345445,9606.ENSP00000345957,9606.ENSP00000346001,9606.ENSP00000346015,9606.ENSP00000346027,9606.ENSP00000346037,9606.ENSP00000346046,9606.ENSP00000346050,9606.ENSP00000346067,9606.ENSP00000346088,9606.ENSP00000348849,9606.ENSP00000349259,9606.ENSP00000352834,9606.ENSP00000354573,9606.ENSP00000354739,9606.ENSP00000355315,9606.ENSP00000356790,9606.ENSP00000357555,9606.ENSP00000359345,9606.ENSP00000361076,9606.ENSP00000362744,9606.ENSP00000363018,9606.ENSP00000363676,9606.ENSP00000363921,9606.ENSP00000365131,9606.ENSP00000365606,9606.ENSP00000368022,9606.ENSP00000369757,9606.ENSP00000373487,9606.ENSP00000375730,9606.ENSP00000377640,9606.ENSP00000378040,9606.ENSP00000378163,9606.ENSP00000379506,9606.ENSP00000379888,9606.ENSP00000385958,9606.ENSP00000386717,9606.ENSP00000389103,9606.ENSP00000393241,9606.ENSP00000397798,9606.ENSP00000400467,9606.ENSP00000403172,9606.ENSP00000413436,9606.ENSP00000414321,9606.ENSP00000416429,9606.ENSP00000418868,[illegible]	VAMP3,TOMM22,CHMP4B,RPL18A,RPL19,RPS12,RPS16,EXOC4,RPL35,SDCBP,RPL8,RAB10,SRP14,NPC1,RPS11,RPS27A,RPS3,ANK3,BAX,PRKCI,RPS23,VPS35,RPS9,HSPA4,RPL13,RPL15,RPL38,RPL4,SRP68,RPS15A,BID,RPLP2,RAB11B,HSP90AA1,RPS7,RPL7,RPS2,SRP72,SAMM50,RPS21,RPL3,RPL27A,RPL21,RPLP1,RPS17,RPS3A,RPSA,RPL22,RPS26,SPTBN1,MYO1C,MACF1,RPL12,RPL39,ATP1B1,RPS27,RPL5,RPL7A,RPS4X,RPL10A,RPL11,PARD3,BAG6,GRIPAP1,OPTN,RPS6,KRT18,RPL13A,RPL24,BAD,RPL34,RPL14,RPS8,RPS14,RPL31,RPL23A,RPS18,RPL17,RPL9,RPL6,RPL10,RPS24,RPL32,RPL29,RPL35A,RPL23,NDUFA13,RPL30,RPS20,RPS25,FAU,RPS13,RPL18,RPLP0,RPL28,RPL26,RPL36,RPL27,ASNA1,RPS19,RPS28,RPS5,CLIP1,RPS10			

T = 72 h, IFNα16: CoV-2 / mock

#Term ID	Term Description	Genes Mapped	Enrichment Score	Direction	False Discovery Rate	Method	Matching Proteins in Your Input (IDs)	Matching Proteins in Your Input (Labels)
GO:0002673	regulation of acute inflammatory response	10	4.99106	top	0.0000398	afc	9606.ENSP00000225698,9606.ENSP00000245907,9606.ENSP00000315130,9606.ENSP00000356399,9606.ENSP00000379191,9606.ENSP00000381607,9606.ENSP00000415941,9606.ENSP00000416561,9606.ENSP00000418770,9606.ENSP00000479488	C1QBP,C3,CLU,CFH,CD59,GSTP1,C4B,CFB,PARK7,PHB
GO:0016266	O-glycan processing	8	5.4561	top	0.0000398	afc	9606.ENSP00000287590,9606.ENSP00000321874,9606.ENSP00000360776,9606.ENSP00000381008,9606.ENSP00000417498,9606.ENSP00000436812,9606.ENSP00000484824,9606.ENSP00000485028	B3GNT7,B3GNT3,B4GALT5,MUC16,MUC4,MUC5B,MUC1,MUC13
GO:0030449	regulation of complement activation	8	5.49766	top	0.0000398	afc	9606.ENSP00000225698,9606.ENSP00000245907,9606.ENSP00000315130,9606.ENSP00000356399,9606.ENSP00000379191,9606.ENSP00000415941,9606.ENSP00000416561,9606.ENSP00000479488	C1QBP,C3,CLU,CFH,CD59,C4B,CFB,PHB
GO:2000257	regulation of protein activation cascade	8	5.49766	top	0.0000398	afc	9606.ENSP00000225698,9606.ENSP00000245907,9606.ENSP00000315130,9606.ENSP00000356399,9606.ENSP00000379191,9606.ENSP00000415941,9606.ENSP00000416561,9606.ENSP00000479488	C1QBP,C3,CLU,CFH,CD59,C4B,CFB,PHB
GO:0006956	complement activation	6	6.45766	top	0.0000531	afc	9606.ENSP00000225698,9606.ENSP00000245907,9606.ENSP00000315130,9606.ENSP00000356399,9606.ENSP00000415941,9606.ENSP00000416561	C1QBP,C3,CLU,CFH,C4B,CFB
GO:0019730	antimicrobial humoral response	17	3.4345	top	0.0000531	afc	9606.ENSP00000229239,9606.ENSP00000253354,9606.ENSP00000254301,9606.ENSP00000315130,9606.ENSP00000342082,9606.ENSP00000346251,9606.ENSP00000355315,9606.ENSP00000357722,9606.ENSP00000357727,9606.ENSP00000357905,9606.ENSP00000362108,9606.ENSP00000379873,9606.ENSP00000428085,9606.ENSP00000435370,9606.ENSP00000452780,9606.ENSP00000470972,9606.ENSP00000483599	GAPDH,BPIFB1,LGALS3,CLU,SLPI,BPIFA1,RPL39,S100A8,S100A9,DMBT1,LCN2,HLA-A,RPL30,FAU,B2M,RPS19,SPON2
GO:0002920	regulation of humoral immune response	9	4.91396	top	0.0000682	afc	9606.ENSP00000225698,9606.ENSP00000245907,9606.ENSP00000315130,9606.ENSP00000356399,9606.ENSP00000379191,9606.ENSP00000391592,9606.ENSP00000415941,9606.ENSP00000416561,9606.ENSP00000479488	C1QBP,C3,CLU,CFH,CD59,PTPN6,C4B,CFB,PHB
GO:0072376	protein activation cascade	7	5.27166	top	0.00034	afc	9606.ENSP00000225698,9606.ENSP00000245907,9606.ENSP00000315130,9606.ENSP00000356399,9606.ENSP00000377055,9606.ENSP00000415941,9606.ENSP00000416561	C1QBP,C3,CLU,CFH,PRCP,C4B,CFB

GO:0006397	mRNA processing	158	0.947642	bottom	0.00036	ks	9606.ENSP00000215793,9606.ENSP00000215829,9606.ENSP00000216727,9606.ENSP00000217109,9606.ENSP00000221419,9606.ENSP00000221494,9606.ENSP00000225698,9606.ENSP00000225792,9606.ENSP00000227524,9606.ENSP00000228284,9606.ENSP00000229390,9606.ENSP00000238112,9606.ENSP00000240185,9606.ENSP00000243563,9606.ENSP00000244020,9606.ENSP00000246194,9606.ENSP00000249299,9606.ENSP00000253363,9606.ENSP00000254108,9606.ENSP00000254193,9606.ENSP00000254301,9606.ENSP00000258962,9606.ENSP00000261167,9606.ENSP00000261531,9606.ENSP00000261973,9606.ENSP00000262710,9606.ENSP00000263579,9606.ENSP00000263694,9606.ENSP00000265866,9606.ENSP00000265872,9606.ENSP00000266079,9606.ENSP00000266679,9606.ENSP00000269349,9606.ENSP00000276079,9606.ENSP00000282007,9606.ENSP00000283179,9606.ENSP00000291552,9606.ENSP00000293677,9606.ENSP00000297071,9606.ENSP00000300291,9606.ENSP00000300413,9606.ENSP00000301740,9606.ENSP00000302160,9606.ENSP00000304903,9606.ENSP00000305556,9606.ENSP00000305790,9606.ENSP00000307863,9606.ENSP00000310448,9606.ENSP00000310670,9606.ENSP00000310723,9606.ENSP00000311677,9606.ENSP00000313007,9606.ENSP00000313199,9606.ENSP00000313829,9606.ENSP00000316042,9606.ENSP00000317123,9606.ENSP00000318861,9606.ENSP00000319169,9606.ENSP00000324122,9606.ENSP00000325376,9606.ENSP00000325377,9606.ENSP00000325905,9606.ENSP00000326806,9606.ENSP00000328848,9606.ENSP00000335321,9606.ENSP00000336741,9606.ENSP00000336752,9606.ENSP00000337632,9606.ENSP00000339245,9606.ENSP00000340796,9606.ENSP00000340823,9606.ENSP00000341826,9606.ENSP00000342374,9606.ENSP00000343966,9606.ENSP00000346634,9	SF3A1,SNRPD3,PABPN1,CSTF1,HNRNPL,SF3A2,C1QBP,DDX5,PRPF19,SART3,SRSF9,CPSF3,TARDBP,SNRPA,SRSF6,RALY,LSM8,RBM39,FUS,SNRPA1,LGALS3,SRSF1,WBP11,SNW1,RBM25,ACIN1,DCPS,SNRNP40,HNRNPH3,CCAR1,PRPF6,CPSF6,EIF4A3,NONO,ZC3H13,HNRNPU,U2AFBP,RAVER1,TRA2A,NUDT21,SNRPD1,SRRM2,LSM3,CD2BP2,PCBP1,SF3B3,U2AF2,SART1,CCAR2,DDX23,PPP1R8,PABPC1,HNRNPD,KHDRBS1,HNRNPA0,SNRNP200,SF3B2,PRMT5,PRPF31,HNRNPM,WDR33,SRSF7,NCBP2,RBM10,SF3B1,DHX15,FIP1L1,SARNP,YTHDC1,ZNF326,GTF2F2,HNRNPA1,SNRPD2,PSPC1,THRAP3,HNRNPA2B1,CGI-74,SON,HNRNPH1,PTBP1,SFPQ,WTAP,PCBP2,CTNNBL1,CDC73,DHX9,RNASEL,ADAR,SYNCRIP,RBM15,AHCYL1,FMR1,RBMX,SRSF11,CDC5L,YBX1,HNRNPH2,CSTF2,SF3A3,SRSF3,HNRNPR,NCBP1,LSM2,HNRNPK,XRN2,SF1,POLR2B,DDX1,RNMT,HNRNPUL1,SRSF2,HNRNPA3,GTF2F1,DDX39B,DDX17,SMU1,KHSRP,KDM1A,NHP2L1,ELAVL1,PRPF40A,RBM4,EFTUD2,STRAP,POLDIP3,HNRNPF,SNRPE,PNPT1,ESRP1,SNRPB,PTBP3,DDX46,TRA2B,U2SURP,SRSF10,ALYREF,LSM6,PLRG1,LUC7L3,HSPA8,CLNS1A,PUF60,CELF1,CHERP,HNRNPC,SRSF5,NOL3,RNPS1,PRPF8,RBM8A,DDX42,LSM4,SUPT5H,SNRNP70,POLR2A,SRRT,SAP18,CPSF1
GO:0006957	complement activation, alternative pathway	3	8.45935	top	0.00055	afc	9606.ENSP00000245907,9606.ENSP00000356399,9606.ENSP00000416561	C3,CFH,CFB

GO:0008380	RNA splicing	151	0.983547	bottom	0.00055	ks	9606.ENSP00000215793,9606.ENSP00000215829,9606.ENSP00000216038,9606.ENSP00000216727,9606.ENSP00000217109,9606.ENSP00000221419,9606.ENSP00000221494,9606.ENSP00000225698,9606.ENSP00000225792,9606.ENSP00000227524,9606.ENSP00000228284,9606.ENSP00000229390,9606.ENSP00000238112,9606.ENSP00000240185,9606.ENSP00000243563,9606.ENSP00000244020,9606.ENSP00000246194,9606.ENSP00000249299,9606.ENSP00000253363,9606.ENSP00000254108,9606.ENSP00000254193,9606.ENSP00000254301,9606.ENSP00000258962,9606.ENSP00000261167,9606.ENSP00000261531,9606.ENSP00000261700,9606.ENSP00000261973,9606.ENSP00000262710,9606.ENSP00000263579,9606.ENSP00000263694,9606.ENSP00000265866,9606.ENSP00000265872,9606.ENSP00000266079,9606.ENSP00000269349,9606.ENSP00000276079,9606.ENSP00000282007,9606.ENSP00000283179,9606.ENSP00000291552,9606.ENSP00000293677,9606.ENSP00000297071,9606.ENSP00000300291,9606.ENSP00000300413,9606.ENSP00000301740,9606.ENSP00000302160,9606.ENSP00000304903,9606.ENSP00000305556,9606.ENSP00000305790,9606.ENSP00000306117,9606.ENSP00000307863,9606.ENSP00000310448,9606.ENSP00000310670,9606.ENSP00000310723,9606.ENSP00000311677,9606.ENSP00000313007,9606.ENSP00000313199,9606.ENSP00000316042,9606.ENSP00000317123,9606.ENSP00000318861,9606.ENSP00000319169,9606.ENSP00000324122,9606.ENSP00000324804,9606.ENSP00000325376,9606.ENSP00000325377,9606.ENSP00000325905,9606.ENSP00000326806,9606.ENSP00000328848,9606.ENSP00000335321,9606.ENSP00000336741,9606.ENSP00000336752,9606.ENSP00000339245,9606.ENSP00000340796,9606.ENSP00000340823,9606.ENSP00000341826,9606.ENSP00000342374,9606.ENSP00000343966,9	SF3A1,SNRPD3,RTCB,PABPN1,CSTF1,HNRNPL,SF3A2,C1QBP,DDX5,PRPF19,SART3,SRSF9,CPSF3,TARDBP,SNRPA,SRSF6,RALY,LSM8,RBM39,FUS,SNRPA1,LGALS3,SRSF1,WBP11,SNW1,C14orf166,RBM25,ACIN1,DCPS,SNRNP40,HNRNPH3,CCAR1,PRPF6,EIF4A3,NONO,ZC3H13,HNRNPU,U2AFBP,RAVER1,TRA2A,NUDT21,SNRPD1,SRRM2,LSM3,CD2BP2,PCBP1,SF3B3,DDX19A,U2AF2,SART1,CCAR2,DDX23,PPP1R8,PABPC1,HNRNPD,HNRNPA0,SNRNP200,SF3B2,PRMT5,PRPF31,PPP2R1A,HNRNPM,WDR33,SRSF7,NCBP2,RBM10,SF3B1,DHX15,FIP1L1,YTHDC1,ZNF326,GTF2F2,HNRNPA1,SNRPD2,PSPC1,THRAP3,HNRNPA2B1,CGI-74,SON,HNRNPH1,PTBP1,SFPQ,WTAP,PCBP2,CTNNBL1,DHX9,SYNCRIP,RBM15,FMR1,RBMX,SRSF11,CDC5L,YBX1,HNRNPH2,CSTF2,SF3A3,SRSF3,HNRNPR,NCBP1,LSM2,HNRNPK,SF1,POLR2B,DDX1,HNRNPUL1,SRSF2,HNRNPA3,GTF2F1,DDX39B,DDX17,SMU1,KHSRP,KDM1A,NHP2L1,ELAVL1,PRPF40A,RBM4,EFTUD2,STRAP,HNRNPF,SNRPE,ESRP1,SNRPB,PTBP3,DDX46,TRA2B,PPP2CA,U2SURP,SRSF10,ALYREF,LSM6,PLRG1,LUC7L3,HSPA8,CLNS1A,PUF60,CELF1,CHERP,HNRNPC,SRSF5,NOL3,RNPS1,PRPF8,RBM8A,DDX42,LSM4,SNRNP70,POLR2A,SRRT,SAP18,CPSF1
GO:0006493	protein O-linked glycosylation	10	3.94918	top	0.0014	afc	9606.ENSP00000247020,9606.ENSP00000287590,9606.ENSP00000321874,9606.ENSP00000360776,9606.ENSP00000381008,9606.ENSP00000417498,9606.ENSP00000425809,9606.ENSP00000436812,9606.ENSP00000484824,9606.ENSP00000485028	SDF2,B3GNT7,B3GNT3,B4GALT5,MUC16,MUC4,PGM3,MUC5B,MUC1,MUC13
GO:0051181	cofactor transport	7	4.62921	top	0.0029	afc	9606.ENSP00000225665,9606.ENSP00000229729,9606.ENSP00000257264,9606.ENSP00000262613,9606.ENSP00000362108,9606.ENSP00000377284,9606.ENSP00000394863	SLC25A11,SLC44A4,TCN1,SLC9A3R1,LCN2,FOLR1,PPT1

GO:0006351	transcription, DNA-templated	192	0.816408	bottom	0.003	ks	9606.ENSP00000012443,9606.ENSP00000200457,9606.ENSP00000215829,9606.ENSP00000215832,9606.ENSP00000216297,9606.ENSP00000216714,9606.ENSP00000216727,9606.ENSP00000217109,9606.ENSP00000217244,9606.ENSP00000225698,9606.ENSP00000225792,9606.ENSP00000226574,9606.ENSP00000228251,9606.ENSP00000229795,9606.ENSP00000233057,9606.ENSP00000234590,9606.ENSP00000234677,9606.ENSP00000238112,9606.ENSP00000240185,9606.ENSP00000244537,9606.ENSP00000246194,9606.ENSP00000252542,9606.ENSP00000253024,9606.ENSP00000253363,9606.ENSP00000253794,9606.ENSP00000260665,9606.ENSP00000261531,9606.ENSP00000261700,9606.ENSP00000262027,9606.ENSP00000262160,9606.ENSP00000262306,9606.ENSP00000262506,9606.ENSP00000262570,9606.ENSP00000263025,9606.ENSP00000264538,9606.ENSP00000264657,9606.ENSP00000265073,9606.ENSP00000265872,9606.ENSP00000267064,9606.ENSP00000267973,9606.ENSP00000268058,9606.ENSP00000269701,9606.ENSP00000274458,9606.ENSP00000276079,9606.ENSP00000278412,9606.ENSP00000278572,9606.ENSP00000283179,9606.ENSP00000290231,9606.ENSP00000290921,9606.ENSP00000292431,9606.ENSP00000293288,9606.ENSP00000295025,9606.ENSP00000296543,9606.ENSP00000296702,9606.ENSP00000297338,9606.ENSP00000299259,9606.ENSP00000299299,9606.ENSP00000300134,9606.ENSP00000300291,9606.ENSP00000300305,9606.ENSP00000301396,9606.ENSP00000302640,9606.ENSP00000302886,9606.ENSP00000304229,9606.ENSP00000309555,9606.ENSP00000310670,9606.ENSP00000311677,9606.ENSP00000311747,9606.ENSP00000311825,9606.ENSP00000312988,9606.ENSP00000313199,9606.ENSP00000313601,9606.ENSP00000313829,9606.ENSP00000315768,9606.ENSP00000316950,9	PPP5C,TRIP6,SNRPD3,MAPK1,SUPT16H,APEX1,PABPN1,CSTF1,CSNK2A1,C1QBP,DDX5,NFKB1,YBX3,MAPK14,EIF2AK2,ENO1,SARS,CPSF3,TARDBP,HIST1H4F,RALY,SAFB2,TRIM28,RBM39,VPS25,LRPPRC,SNW1,C14orf166,MARS,SMAD2,TCEB2,CSNK2A2,CHCHD3,MAPK3,IFT57,STAT3,SUB1,CCAR1,SMARCC2,WDR61,PML,AKAP8,COMMD10,NONO,SSRP1,RPS3,HNRNPU,NCOA5,CTBP1,NACC1,BAX,REL,NAA15,TCERG1,RAD21,COPS2,PCBD1,STAT6,NUDT21,RUNX1,PELP1,UBTF,PA2G4,HINT1,HCFC1,CCAR2,PPP1R8,RBM14,CTBP2,NFKBIB,HNRNPD,CC2D1A,KHDRBS1,STAT2,MYEF2,CPNE1,FUBP3,RUVBL1,PRMT5,CNOT1,YLPM1,FLII,WDR33,NCBP2,PURA,CBX3,FIP1L1,SARNP,EEF1A1,ZNF326,GTF2F2,SP100,TRIM29,PSPC1,CTNNB1,PRKAA1,THRAP3,SND1,CHD4,PTRF,SFPQ,COPS5,XRCC6,STAT1,ILF2,IRF2BP2,CDC73,TROVE2,UCHL5,DHX9,CTGF,IFI16,HDGF,FLNA,NFKB2,NPM3,PKN2,RBMX,FUBP1,MCTS1,CDC5L,POLR1C,YBX1,CSTF2,RPRD1B,RBBP4,TXN,NCBP1,NELFE,HNRNPK,XRN2,SF1,TRIM22,RBBP7,PIR,SLTM,POLR2B,DDX1,HMG20A,TP53BP1,RNMT,LRRFIP1,HNRNPUL1,XRCC5,DYNLL1,MLF1,NME1-NME2,GTF2F1,CHCHD2,PURB,ETV6,DDX17,IRF9,DEK,KHSRP,CTNND1,DDX3X,KDM1A,RELA,EWSR1,SNRPE,PPP1R13L,ILF3,TBL1XR1,EEF1D,SNRPB,CBFB,ANP32A,PARP14,CDKN2A,TCEA1,HDAC2,HSPA8,PUF60,BCLAF1,PHB2,NACA,SLIRP,RNPS1,CIC,GTF2I,SAFB,ZSCAN18,SUPT5H,RUVBL2,TAF15,TCEB1,POLR2A,SRRT,SAP18,HNRNPDL,CPSF1

GO:0000375	RNA splicing, via transesterification reactions	127	0.971936	bottom	0.0042	ks	9606.ENSP00000215793,9606.ENSP00000215829,9606.ENSP00000216727,9606.ENSP00000217109,9606.ENSP00000221419,9606.ENSP00000221494,9606.ENSP00000225792,9606.ENSP00000227524,9606.ENSP00000228284,9606.ENSP00000229390,9606.ENSP00000238112,9606.ENSP00000243563,9606.ENSP00000244020,9606.ENSP00000246194,9606.ENSP00000249299,9606.ENSP00000254108,9606.ENSP00000254193,9606.ENSP00000258962,9606.ENSP00000261167,9606.ENSP00000261531,9606.ENSP00000263579,9606.ENSP00000263694,9606.ENSP00000265866,9606.ENSP00000265872,9606.ENSP00000266079,9606.ENSP00000269349,9606.ENSP00000276079,9606.ENSP00000283179,9606.ENSP00000291552,9606.ENSP00000293677,9606.ENSP00000297071,9606.ENSP00000300291,9606.ENSP00000300413,9606.ENSP00000301740,9606.ENSP00000302160,9606.ENSP00000304903,9606.ENSP00000305556,9606.ENSP00000305790,9606.ENSP00000307863,9606.ENSP00000310448,9606.ENSP00000310723,9606.ENSP00000313007,9606.ENSP00000313199,9606.ENSP00000316042,9606.ENSP00000317123,9606.ENSP00000318861,9606.ENSP00000319169,9606.ENSP00000324122,9606.ENSP00000325376,9606.ENSP00000325377,9606.ENSP00000325905,9606.ENSP00000326806,9606.ENSP00000335321,9606.ENSP00000336741,9606.ENSP00000336752,9606.ENSP00000339245,9606.ENSP00000340823,9606.ENSP00000341826,9606.ENSP00000342374,9606.ENSP00000343966,9606.ENSP00000346694,9606.ENSP00000347005,9606.ENSP00000349168,9606.ENSP00000349428,9606.ENSP00000349748,9606.ENSP00000352438,9606.ENSP00000355050,9606.ENSP00000356520,9606.ENSP00000358635,9606.ENSP00000358799,9606.ENSP00000359645,9606.ENSP00000359988,9606.ENSP00000360532,9606.ENSP00000361626,9606.ENSP00000361927,9	SF3A1,SNRPD3,PABPN1,CSTF1,HNRNPL,SF3A2,DDX5,PRPF19,SART3,SRSF9,CPSF3,SNRPA,SRSF6,RALY,LSM8,FUS,SNRPA1,SRSF1,WBP11,SNW1,DCPS,SNRNP40,HNRNPH3,CCAR1,PRPF6,EIF4A3,NONO,HNRNPU,U2AFBP,RAVER1,TRA2A,NUDT21,SNRPD1,SRRM2,LSM3,CD2BP2,PCBP1,SF3B3,U2AF2,SART1,DDX23,PABPC1,HNRNPD,HNRNPA0,SNRNP200,SF3B2,PRMT5,PRPF31,HNRNPM,WDR33,SRSF7,NCBP2,SF3B1,DHX15,FIP1L1,YTHDC1,GTF2F2,HNRNPA1,SNRPD2,PSPC1,HNRNPA2B1,CGI-74,HNRNPH1,PTBP1,SFPQ,PCBP2,CTNNBL1,DHX9,SYNCRIP,RBM15,RBMX,SRSF11,CDC5L,YBX1,HNRNPH2,CSTF2,SF3A3,SRSF3,HNRNPR,NCBP1,LSM2,HNRNPK,SF1,POLR2B,DDX1,HNRNPUL1,SRSF2,HNRNPA3,GTF2F1,DDX39B,DDX17,SMU1,KHSRP,KDM1A,NHP2L1,ELAVL1,PRPF40A,EFTUD2,STRAP,HNRNPF,SNRPE,SNRPB,DDX46,TRA2B,U2SURP,SRSF10,ALYREF,LSM6,PLRG1,LUC7L3,HSPA8,CLNS1A,PUF60,CELF1,CHERP,HNRNPC,SRSF5,NOL3,RNPS1,PRPF8,RBM8A,DDX42,LSM4,SNRNP70,POLR2A,SRRT,CPSF1
GO:0006959	humoral immune response	25	3.83141	top	0.0042	ks	9606.ENSP00000225698,9606.ENSP00000229239,9606.ENSP00000245907,9606.ENSP00000253354,9606.ENSP00000254301,9606.ENSP00000315130,9606.ENSP00000342082,9606.ENSP00000346251,9606.ENSP00000351314,9606.ENSP00000353099,9606.ENSP00000355315,9606.ENSP00000356399,9606.ENSP00000357722,9606.ENSP00000357727,9606.ENSP00000357905,9606.ENSP00000362108,9606.ENSP00000379873,9606.ENSP00000405573,9606.ENSP00000415941,9606.ENSP00000416561,9606.ENSP00000428085,9606.ENSP00000435370,9606.ENSP00000452780,9606.ENSP00000470972,9606.ENSP00000483599	C1QBP,GAPDH,C3,BPIFB1,LGALS3,CLU,SLPI,BPIFA1,PSMB10,HLA-DRB1,RPL39,CFH,S100A8,S100A9,DMBT1,LCN2,HLA-A,GPI,C4B,CFB,RPL30,FAU,B2M,RPS19,SPON2

GO:0000398	mRNA splicing, via spliceosome	126	0.966932	bottom	0.0054	ks	9606.ENSP00000215793,9606.ENSP00000215829,9606.ENSP00000216727,9606.ENSP00000217109,9606.ENSP00000221419,9606.ENSP00000221494,9606.ENSP00000225792,9606.ENSP00000227524,9606.ENSP00000228284,9606.ENSP00000229390,9606.ENSP00000238112,9606.ENSP00000243563,9606.ENSP00000244020,9606.ENSP00000246194,9606.ENSP00000249299,9606.ENSP00000254108,9606.ENSP00000254193,9606.ENSP00000258962,9606.ENSP00000261167,9606.ENSP00000261531,9606.ENSP00000263579,9606.ENSP00000263694,9606.ENSP00000265866,9606.ENSP00000265872,9606.ENSP00000266079,9606.ENSP00000269349,9606.ENSP00000276079,9606.ENSP00000283179,9606.ENSP00000291552,9606.ENSP00000293677,9606.ENSP00000297071,9606.ENSP00000300291,9606.ENSP00000300413,9606.ENSP00000301740,9606.ENSP00000302160,9606.ENSP00000304903,9606.ENSP00000305556,9606.ENSP00000305790,9606.ENSP00000307863,9606.ENSP00000310448,9606.ENSP00000310723,9606.ENSP00000313007,9606.ENSP00000313199,9606.ENSP00000316042,9606.ENSP00000317123,9606.ENSP00000318861,9606.ENSP00000319169,9606.ENSP00000324122,9606.ENSP00000325376,9606.ENSP00000325377,9606.ENSP00000325905,9606.ENSP00000326806,9606.ENSP00000335321,9606.ENSP00000336741,9606.ENSP00000336752,9606.ENSP00000339245,9606.ENSP00000340823,9606.ENSP00000341826,9606.ENSP00000342374,9606.ENSP00000343966,9606.ENSP00000346694,9606.ENSP00000347005,9606.ENSP00000349168,9606.ENSP00000349428,9606.ENSP00000349748,9606.ENSP00000352438,9606.ENSP00000355050,9606.ENSP00000356520,9606.ENSP00000358635,9606.ENSP00000358799,9606.ENSP00000359645,9606.ENSP00000359988,9606.ENSP00000360532,9606.ENSP00000361626,9606.ENSP00000361927,9	SF3A1,SNRPD3,PABPN1,CSTF1,HNRNPL,SF3A2,DDX5,PRPF19,SART3,SRSF9,CPSF3,SNRPA,SRSF6,RALY,LSM8,FUS,SNRPA1,SRSF1,WBP11,SNW1,DCPS,SNRNP40,HNRNPH3,CCAR1,PRPF6,EIF4A3,NONO,HNRNPU,U2AFBP,RAVER1,TRA2A,NUDT21,SNRPD1,SRRM2,LSM3,CD2BP2,PCBP1,SF3B3,U2AF2,SART1,DDX23,PABPC1,HNRNPD,HNRNPA0,SNRNP200,SF3B2,PRMT5,PRPF31,HNRNPM,WDR33,SRSF7,NCBP2,SF3B1,DHX15,FIP1L1,YTHDC1,GTF2F2,HNRNPA1,SNRPD2,PSPC1,HNRNPA2B1,CGI-74,HNRNPH1,PTBP1,SFPQ,PCBP2,CTNNBL1,DHX9,SYNCRIP,RBM15,RBMX,SRSF11,CDC5L,YBX1,HNRNPH2,CSTF2,SF3A3,SRSF3,HNRNPR,NCBP1,LSM2,HNRNPK,SF1,POLR2B,DDX1,HNRNPUL1,SRSF2,HNRNPA3,GTF2F1,DDX39B,DDX17,SMU1,KDM1A,NHP2L1,ELAVL1,PRPF40A,EFTUD2,STRAP,HNRNPF,SNRPE,SNRPB,DDX46,TRA2B,U2SURP,SRSF10,ALYREF,LSM6,PLRG1,LUC7L3,HSPA8,CLNS1A,PUF60,CELF1,CHERP,HNRNPC,SRSF5,NOL3,RNPS1,PRPF8,RBM8A,DDX42,LSM4,SNRNP70,POLR2A,SRRT,CPSF1
GO:0019731	antibacterial humoral response	8	3.96728	top	0.0079	afc	9606.ENSP00000342082,9606.ENSP00000346251,9606.ENSP00000355315,9606.ENSP00000357905,9606.ENSP00000379873,9606.ENSP00000435370,9606.ENSP00000452780,9606.ENSP00000483599	SLPI,BPIFA1,RPL39,DMBT1,HLA-A,FAU,B2M,SPON2

T = 72 h, IFNλ3: CoV-2 / mock

#Term ID	Term Description	Genes Mapped	Enrichment Score	Direction	False Discovery Rate	Method	Matching Proteins in Your Input (IDs)	Matching Proteins in Your Input (Labels)
GO:0016266	O-glycan processing	6	6.63864	top	0.00016	afc	9606.ENSP00000287590,9606.ENSP00000360776,9606.ENSP00000381008,9606.ENSP00000417498,9606.ENSP00000436812,9606.ENSP00000484824	B3GNT7,B4GALT5,MUC16,MUC4,MUC5B,MUC1
GO:0006493	protein O-linked glycosylation	7	5.44046	top	0.00082	afc	9606.ENSP00000287590,9606.ENSP00000360776,9606.ENSP00000381008,9606.ENSP00000417498,9606.ENSP00000425809,9606.ENSP00000436812,9606.ENSP00000484824	B3GNT7,B4GALT5,MUC16,MUC4,PGM3,MUC5B,MUC1
GO:0030449	regulation of complement activation	7	5.34925	top	0.00082	afc	9606.ENSP00000225698,9606.ENSP00000245907,9606.ENSP00000315130,9606.ENSP00000356399,9606.ENSP00000379191,9606.ENSP00000416561,9606.ENSP00000479488	C1QBP,C3,CLU,CFH,CD59,CFB,PHB
GO:2000257	regulation of protein activation cascade	7	5.34925	top	0.00082	afc	9606.ENSP00000225698,9606.ENSP00000245907,9606.ENSP00000315130,9606.ENSP00000356399,9606.ENSP00000379191,9606.ENSP00000416561,9606.ENSP00000479488	C1QBP,C3,CLU,CFH,CD59,CFB,PHB
GO:0006957	complement activation, alternative pathway	3	8.57137	top	0.0013	afc	9606.ENSP00000245907,9606.ENSP00000356399,9606.ENSP00000416561	C3,CFH,CFB
GO:0002673	regulation of acute inflammatory response	9	4.48606	top	0.0025	afc	9606.ENSP00000225698,9606.ENSP00000245907,9606.ENSP00000315130,9606.ENSP00000356399,9606.ENSP00000379191,9606.ENSP00000381607,9606.ENSP00000416561,9606.ENSP00000418770,9606.ENSP00000479488	C1QBP,C3,CLU,CFH,CD59,GSTP1,CFB,PARK7,PHB
GO:0006956	complement activation	5	6.08964	top	0.0025	afc	9606.ENSP00000225698,9606.ENSP00000245907,9606.ENSP00000315130,9606.ENSP00000356399,9606.ENSP00000416561	C1QBP,C3,CLU,CFH,CFB
GO:0002920	regulation of humoral immune response	8	4.56308	top	0.0039	afc	9606.ENSP00000225698,9606.ENSP00000245907,9606.ENSP00000315130,9606.ENSP00000356399,9606.ENSP00000379191,9606.ENSP00000391592,9606.ENSP00000416561,9606.ENSP00000479488	C1QBP,C3,CLU,CFH,CD59,PTPN6,CFB,PHB
GO:0072376	protein activation cascade	6	5.18805	top	0.0066	afc	9606.ENSP00000225698,9606.ENSP00000245907,9606.ENSP00000315130,9606.ENSP00000356399,9606.ENSP00000377055,9606.ENSP00000416561	C1QBP,C3,CLU,CFH,PRCP,CFB

Supplementary Table 4: Primer sequences for Taqman PCR

Primer	Sequence (5´-3´)
GAPDH fwd	ccatcttccaggagcgaga
GAPDH rev	caggaggcattgctgatgatc
GAPDH probe	YAK-agtggggcgatgctggcgctgag-BBQ
β-Actin fwd	cacccgccgccagctcaccat
β-Actin rev	caccatcacgccctggtgcct
β-Actin probe	Cy5-gatatcgccgcgctcgtcgtcgacaacggc-BBQ
M-gene fwd	tgtgacatcaaggacctgcc
M-gene rev	ctgagtcacctgctacacgc
M-gene probe (PMID 32575728)	FAM-tgttgctacatcacgaacgc-BHQ1
N-gene fwd (pWhSF-N-F9)	acattggcacccgcaatc
N-gene rev (pWhSF-N-R10)	cgagaagaggcttgactgcc
N-gene probe (pWhSF-N-P11lna)	FAM-cct+caaggaa+caa+catt-BHQ1

(+ = LNA (locked nucleic acid) modified)

Supplementary Table 5: Primer sequences for Sybr Green PCR

Gene	Forward Primer Sequence (5´-3´)	Reverse Primer Sequence (5´-3´)
GAPDH	caaggtcatccatgacaactttg	gtccaccaccctgttgctgtag
APOBEC3G	ggttacctgcttcacctcct	atcatagatgcgggcagtga
IDO1	caaaggtcatggagatgtcc	ccaccaatagagagaccagg
IFITM3	ggtcttcgctggacaccat	tgtccctagacttcacggagta
ISG15	gagaggcagcgaactcatct	agggacacctggaattcgtt
Tetherin	aagggctttcaggatgtgga	tttgtccttgggccttctct
